# Combined proteomics and transcriptomics reveal the genetic basis underlying the differentiation of skin appendages and immunity in pangolin

**DOI:** 10.1038/s41598-020-71513-w

**Published:** 2020-09-03

**Authors:** Hui-Ming Li, Ping Liu, Xiu-Juan Zhang, Lin-Miao Li, Hai-Ying Jiang, Hua Yan, Fang-Hui Hou, Jin-Ping Chen

**Affiliations:** 1 Guangdong Key Laboratory of Animal Conservation and Resource Utilization, Guangdong Public Laboratory of Wild Animal Conservation and Utilization, Institute of Zoology, Guangdong Academy of Science, Guangzhou, 510260 China; 2 Guangdong Provincial Key Laboratory of Silviculture, Protection and Utilization, Guangdong Academy of Forestry, Guangzhou, Guangdong Province China; 3 Guangdong Provincial Wildlife Rescue Centre, Guangzhou, Guangdong Province China

**Keywords:** Differentiation, Developmental biology, Molecular biology, Proteomics, Transcriptomics

## Abstract

Pangolin (*Mains javanica*) is an interesting endangered mammal with special morphological characteristics. Here, we applied proteomics and transcriptomics to explore the differentiation of pangolin skin appendages at two developmental stages and to compare gene expression profiles between abdomen hair and dorsal scale tissues. We identified 4,311 genes and 91 proteins differentially expressed between scale-type and hair-type tissue, of which 6 genes were shared by the transcriptome and proteome. Differentiation altered the abundance of hundreds of proteins and mRNA in the two types of skin appendages, many of which are involved in keratinocyte differentiation, epidermal cell differentiation, and multicellular organism development based on GO enrichment analysis, and FoxO, MAPK, and p53 signalling pathways based on KEGG enrichment analysis. DEGs in scale-type tissues were also significantly enriched in immune-related terms and pathways compared with that in hair-type tissues. Thus, we propose that pangolins have a normal skin innate immune system. Compared with the abdomen, the back skin of pangolins had more genes involved in the regulation of immune function, which may be an adaptive adjustment for the vulnerability of scaly skin to infection and injury. This investigation provides a scientific basis for the study of development and immunity of pangolin skin, which may be helpful in the protection of wild pangolin in China.

## Introduction

The skin is the largest organ of the body, strategically located at the barrier between the interior and exterior^1^. It performs important physiological functions and is widely involved in life processes including growth and development, defence and protection, and tumorigenesis^2^. Skin appendages are formed by keratinisation of surface cells^3,4^. In the process of long-term contact with the environment, the reptilian ancestor’s skin forms an anatomical placode as the origin, and animal skin gives rise to various appendages, such as feathers, thorns, scales, and so on^5^. Skin appendages are important to an organism not only for its physical defence, but also for its ability to renew itself and perform immune protection. Therefore, key signalling molecules and pathways in the development, differentiation, and subsequent circulation of skin appendages are crucial to understanding the diversity, skin pathologies, and immune functions of skin appendages.

Pangolins, (*Mains javanica*), unlike other placental mammals, have a unique skin covered by large overlapping keratinised scales^6^, no teeth, poor vision, and comprise a placental order (Pholidota)^7^. Perceiving and responding to life-threatening signals and regulating their own morphological characteristics constitute a fundamental challenge for these troglobites, who live in holes. Pangolin scales, which are soft on newborn pangolins but harden as the animals mature, are formed by keratins^8^, guaranteeing a high abrasive wear resistance^9^. Apparently, the scales of pangolins are better able to help organisms in their physical defence, protecting them from predators^10^. Springer and Gatesy (2018) found that the mutation or deletion of *MC5R* gene related to sebaceous gland development was an important molecular affecting the unusual skin characteristics of pangolin^11^, and even the evolution of scales on their backs is thought to have compensated for the lower body immunity^7^. Although the selective forces behind the origin of this unique mammalian trait remain a mystery, pangolin scales and hair are natural ‘mutants’ for studying the development and differentiation of skin appendages. In addition to keratin genes, numerous genes and signalling pathways have been reported to be involved in the regulation of animal hair cell dynamics, growth activities, and changes in different stages of the growth cycle, including bone morphogenetic protein (BMP)^12,13^, notch signalling pathway, Wnt signalling pathway^14,15^, Hedgehog signalling pathway^16^, fiber growth factor (FGF)^17,18^, and transforming growth factor (TGF)^19^.

The abovementioned studies primarily focused on mammalian hair, little is known about changes in gene expression during scale differentiation. This investigation provides a scientific basis for the study of the development and differentiation of pangolin skin and will help protect wild pangolin in China. The results will also provide a benchmark for comparative studies of other mammalian hair, including that of humans, and provide important reference information for the prevention of hair, scale, and other diseases.

## Results

### RNA-seq transcriptomic and label-free quantitative proteomic analysis of *M. javanica* skin tissues

**Figure 1:**
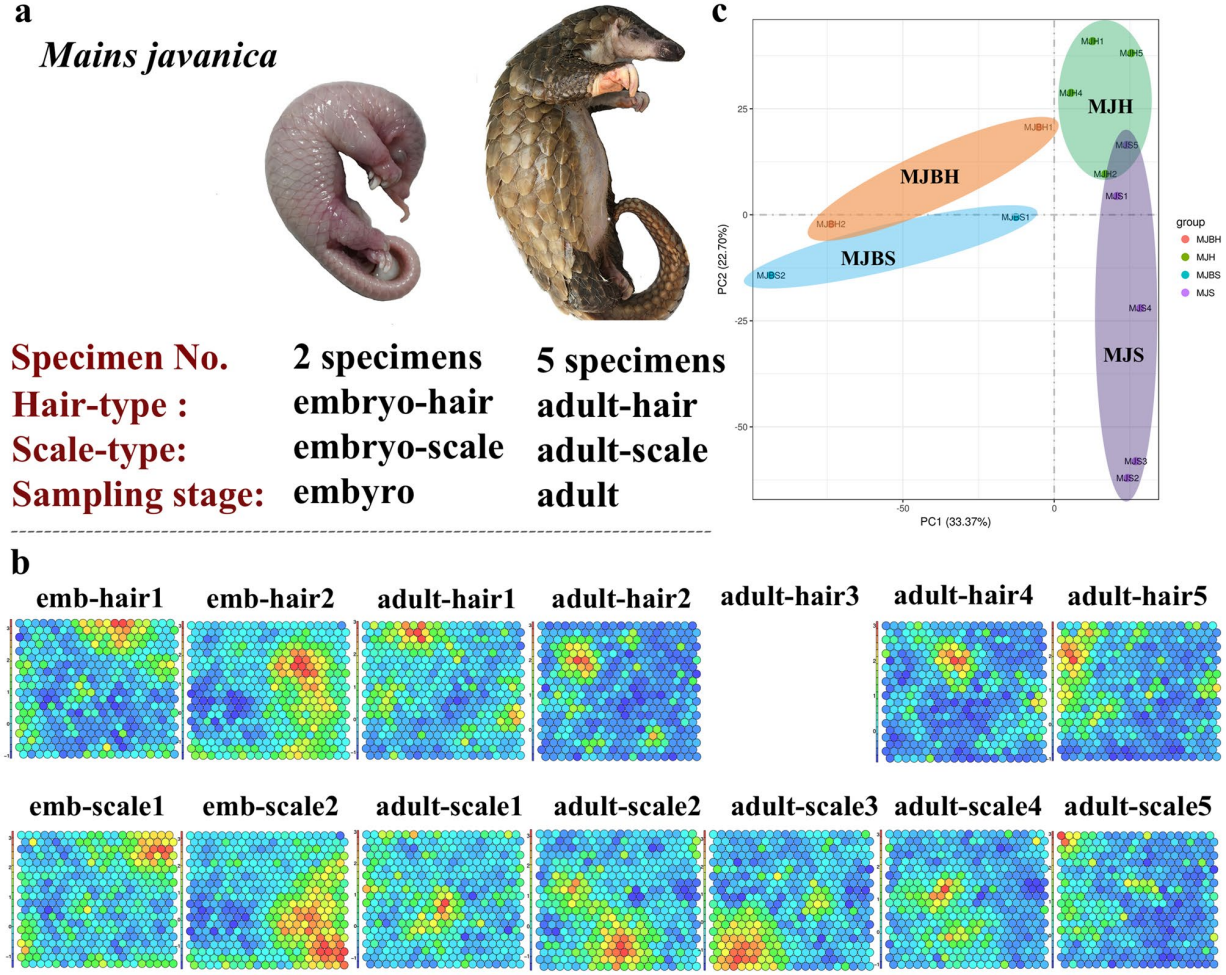
Sample information, SOM module, and PCA analysis. (**a**) Different phenotypes from two different development stages of *Manis javanica*.; (**b**) Gene expression-specific and phenotype-specific gene-trait correlation analysis based on self-organizing feature map module analysis (SOM module), colour scale of SOM component plane represented the mean ratio in each map node, and red indicates high expression, blue indicates low expression; (**c**) Principal components analysis (PCA) displaying biological variation between scale-type and hair-type samples in pangolin skin appendages. (The adult-hair3 sample in the sequencing results was of poor quality so this sample was deleted).

To identify the molecular mechanisms governing the development and differentiation of skin appendages, we harvested skin samples from *M. javanica* in biological replicates with 7 specimens including 2 embryos and 5 adult specimens at two developmental stages for two skin appendage types: embryo/adult-hair and embryo/adult-scale group (Fig. 1a). The 14 samples were divided into identical pools and subjected to label-free MS-based proteomics and RNA-seq-based transcriptomics. The adult-hair3 sample in the sequencing results was of poor data quality; thus, this sample was deleted from our transcriptome data.

The Illumina sequencing reads were deposited in the Short Read Archive as accession number PRJNA610466 for *M. javanica*. For the two types of pangolin skin, a total of 84,182,093 clean reads were obtained, corresponding to 90.54% of all high-quality reference genes of the *M. javanica* genome (NCBI BioProjects: PRJNA335369; Table 1). A total of 36,043 annotated RNAs were obtained, of which 21,302 were protein-encoding. A neural network graph based on self-organizing map (SOM) analysis^20^ revealed dynamic changes of gene expression occurring at the individual developmental stages (Fig. 1b). We evaluated biological reproducibility by comparing the biological replicates individually by principle component analysis (PCA) with all skin appendages (Fig. 1c). As expected, a similar expression pattern was observed for some of the genes in both hair- and scale-type samples.

**Table 1:** Statistics of sequencing data quality and reference genome mapping.

Sample type	Sample ID	Total map %	Clean reads	Clean bases (G)	Q20	Q30	GC (%)
Embryo (embryo-hair/scale)	Embryo-hair1	92.61	93,690,874	14.05	97.89	94.03	51.46
	Embryo-hair2	91.97	81,022,760	12.15	97.48	93.13	51.96
	Embryo-scale1	92.41	79,894,162	11.98	97.79	93.8	53.05
	Embryo-scale2	91.48	78,930,270	11.84	97.57	93.32	51.53
Adult (adult-hair/scale)	Adult-hair1	92.16	82,382,356	12.36	97.28	92.63	49.58
	Adult-hair2	93.24	78,570,822	11.79	97.53	93.09	50.92
	Adult-hair4	91.97	77,617,244	11.64	97.66	93.55	50.89
	Adult-hair5	91.03	76,474,746	11.47	97.68	93.66	51.94
	Adult-scale1	91.71	80,214,986	12.03	97.76	93.81	52.88
	Adult-scale2	92.68	77,279,612	11.59	97.87	93.92	50.84
	Adult-scale3	71.8	126,485,454	18.97	97.26	92.84	49.28
	Adult-scale4	93.28	82,736,446	12.41	97.74	93.65	50.47
	Adult-scale5	90.64	79,067,482	11.86	97.59	93.42	50.86

Based on these results, a total of 8,854 new genes were discovered in the transcriptomic datasets (Supplementary Table 1), of which 7,800 were annotated by GO and KEGG database, 38 new genes were identified as being involved in KEGG pathways related to skin appendage development and differentiation, such as apoptosis, cell cycle, Hedgehog signalling pathway, etc. The new genes provide new information for studying the differentiation mechanisms of pangolins.

To complement the transcriptomic analyses, we carried out a systematic label-free quantitative proteomic analysis. Following MaxQuant analysis, peptide sequences were searched against the transcriptome reference dataset. In total, 2,904 proteins containing 26,472 peptides were successfully identified from 175,115 matched spectra, of which 2,643 proteins were shared by the transcriptome and proteome data.

### DEGs related to abdominal hair development of *M. javanica*

**Figure 2:**
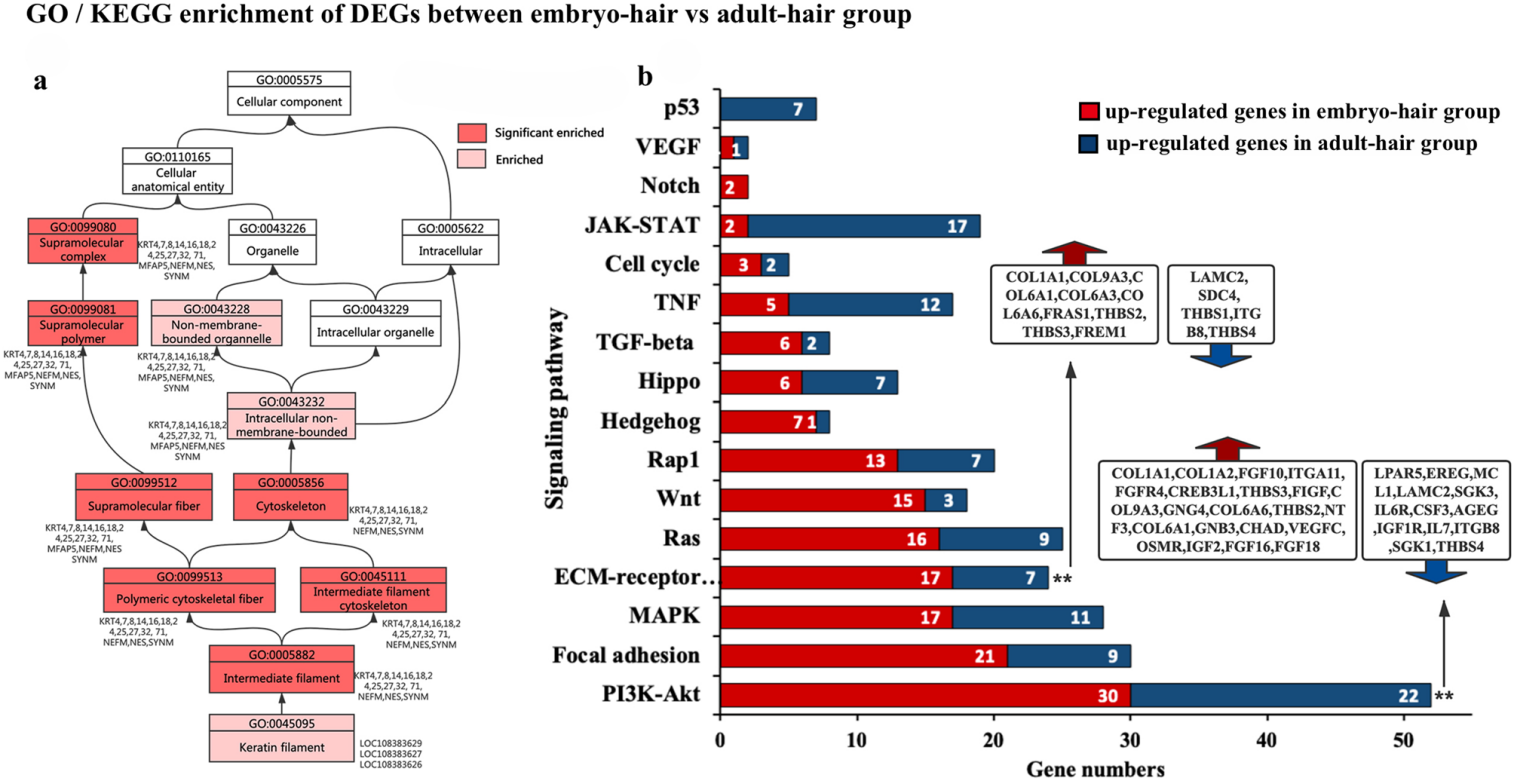
GO and KEGG enrichment analysis of DEGs between embryo-hair vs adult-hair group. (**a**) Directed acyclic graph (DAG) associated with keratin filament term based on GO enrichment analysis; (**b**) Genes and signalling pathways related to hair development.

To further understand the genetic basis of abdominal hair development of pangolin, we conducted DEGs analysis before (embryo-hair) and after (adult-hair) occurrence of abdominal hair. In total, 2,248 DEGs were identified between embryo-hair vs adult-hair group; among them, 862 genes were up-regulated in adult-hair group and the remaining 1,386 genes were up-regulated in embryo-hair group (Supplementary Table 2). Based on the annotation of the GO database, the up-regulated DEGs in embryo-hair group were significantly enriched in the component categories related to transporter activity, including transmembrane transporter activity, substrate-specific transporter activity, ion transport, channel activity, etc. However, the up-regulated DEGs in adult-hair group were significantly enriched in categories related to filaments, such as intermediate filaments, intermediate filament cytoskeleton, keratin filaments, etc. (Supplementary Table 3, 4). And its directed acyclic graph of enriched GO terms associated with the keratin filament term, genes related to the supramolecular complex, supramolecular polymer, supramolecular fiber, intermediate filament, intermediate filament cytoskeleton, polymeric cytoskeletal fiber, and cytoskeleton were significantly enriched. The associated genes KRT14, KRT25, KRT32, KRT71, and SYNM may play an important role in the development of abdominal hair in pangolin (Fig. 2a).

For the KEGG enrichment analysis, a total of 161 up-regulated and 117 down-regulated genes in embryo-hair group of 16 unique KEGG pathways related to the hair development process were identified. Among these genes, PI3K-Akt and the ECM-receptor interaction signalling pathway were significantly enriched, and several enriched pathways related to hair development such as focal adhesion, hedgehog, Wnt pathways, etc., were identified (Fig. 2b). It is noteworthy that in embryo-hair group, the up-regulated genes FGF10, VEGFC, FGF16, FGF18, FGFR4, THBS4, EREG, MCL1, and AREG do not only play an important regulatory role in the development of abdominal hair in pangolin, but they also participate in various processes including cellular apoptosis, proliferation, adhesion and attachment, and the inflammatory response. Furthermore, they are involved in epidermal growth, animal organ morphogenesis, and keratinocyte proliferation in GO annotation.

We conducted distinct expressed proteins (DEPs) analysis before and after hair development based on our proteome data. In total, 67 DEPs (embryo-hair vs adult-hair group) were identified, of which 34 proteins were shared with DEGs in the transcriptome (Supplementary Table 5). Among these DEPs, were proteins involved in cell attachment, controlling growth, and intermediate filaments in GO annotation, including 2 up-regulated genes in embryo-hair group: COL11A1 and POSTN, and 5 up-regulated genes in adult-hair group: FCGR2B, PTBP3, DSC1, CDSN, and CSTA.

### DEGs related to dorsal scale development of *M. javanica*

**Figure 3:**
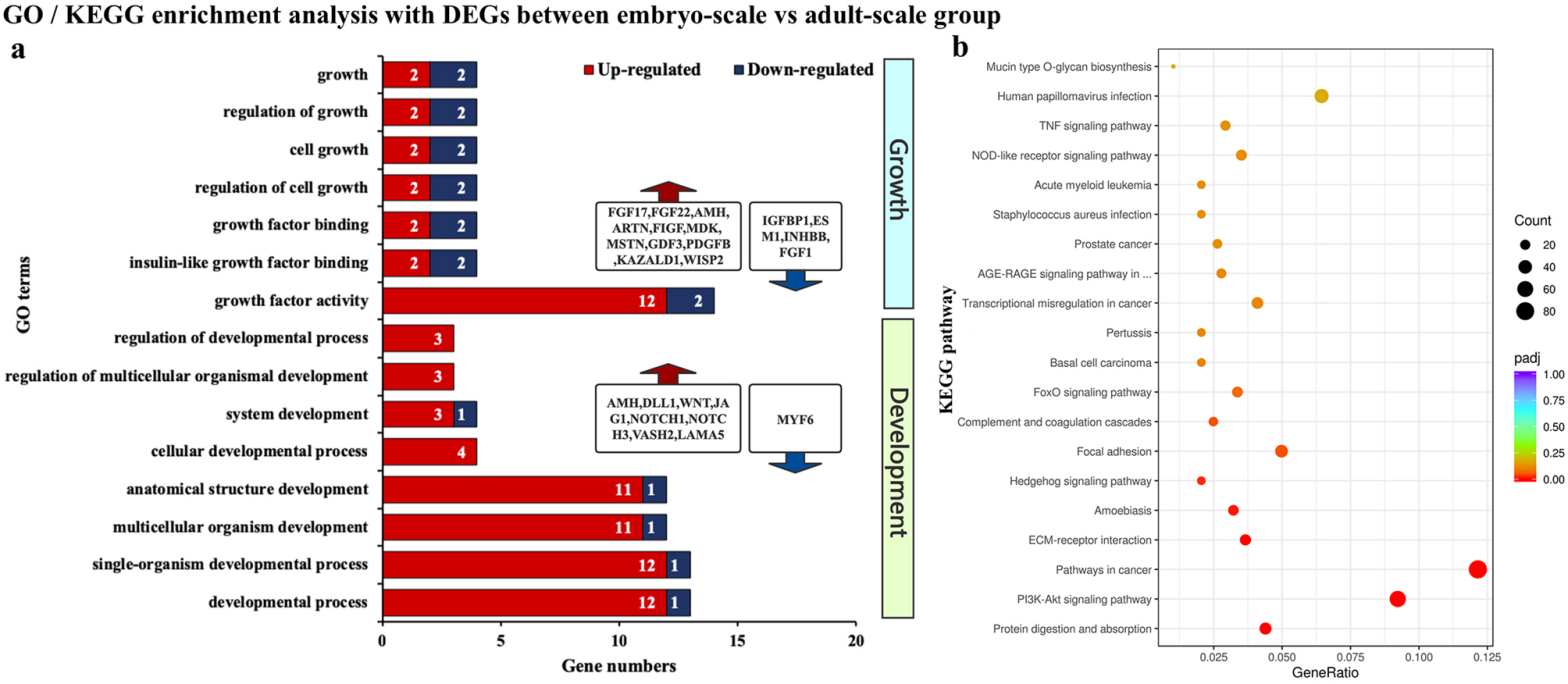
GO and KEGG enrichment analysis of DEGs between embryo-scale vs adult-scale group. (**a**) GO terms related to growth and development of skin appendages; (**b**) Bubble diagram of KEGG enrichment analysis.

To identify genes that activate the development of pangolin scales, we analysed DEGs before (embryo-scale) and after (adult-scale) the occurrence of dorsal scale. A total of 2,556 DEGs were identified between embryo-scale vs adult-scale group, of which 875 genes were up-regulated in adult-scale group, and the remaining 1,681 genes were up-regulated in embryo-scale group. In the GO enrichment analysis with all DEGs, 7 terms related to the extracellular region and metallopeptidase activity were significantly enriched. In addition, 15 terms related to development and growth were mainly enriched in growth factor activity, regulation of cell growth, multicellular organism development, anatomical structure development, etc. (Fig. 3a). In the KEGG enrichment analysis, genes associated with PI3-Akt, ECM-receptor interaction, Hedgehog, and focal adhesion signalling pathways were significantly enriched (Fig. 3b). In addition, there were 16 signalling pathways related to scale development (Table 2). Notably, FGF1, SGK1, IGFBP3, TEAD4, SERPINE1, and KRAS genes were highly expressed in adult-scale group. We speculate that these genes play an important role in activating the development of pangolin scales.

**Table 2:** KEGG signalling pathways related to skin appendage differentiation with DEGs between embryo-scale and adult-scale (embryo-scale vs adult-scale group).

KEGG ID	KEGG pathway	padj	Up-regulated gene in embryo-scale group	Up-regulated gene in adult-scale group
mmu04151	PI3K-Akt signaling pathway	< 0.001	FGFR4, LPAR6, CREB3L1, PDGFB, LAMA5, LAMB2, NTF3, FGF22, FGFR1, CCND1, FGF17, NGF, ITGB6, FGFR2, CDK2	MCL1, OSMR, GNG3, CSF3R, CSF3, FGF1, KRAS, CHUK, AKT3, SGK1, THBS4, ANGPT2, NFKB1, TLR4, SGK3
mmu04512	ECM-receptor interaction	< 0.001	FRAS1, THBS3, HMMR, ITGB7, AGRN, NPNT, LAMA5, LAMB2, SV2A, ITGB6, ITGA11, CHAD, ITGA5	SDC4, THBS4
mmu04340	Hedgehog signaling pathway	0.013	PTCH2, BOC, GLI2, SMO, KIF7, GLI1, CCND1, GLI3, MEGF8, HHIP	SPOPL, PRKACB
mmu04510	Focal adhesion	0.042	MYL7, THBS3, ITGB7, MYL10, VAV2, PDGFB, LAMA5, LAMB2, CCND1, ITGB6, ITGA11, MYL9	BIRC3, IGF1R, AKT3, THBS4
mmu04068	FoxO signaling pathway	0.074	CCNB1, PLK1, CCND1, CDK2	SOD2, FBXO32, STAT3, FOXO1, IGF1R, KRAS, CHUK, S1PR1, AKT3, SGK1, S1PR4, SGK3
mmu04668	TNF signaling pathway	0.126	CREB3L1, FIGF, RIPK3, PIK3R2, JAG1	CEBPB, SOCS3, IL6, CCL20, BIRC3, MMP9, TNFAIP3, CHUK, TNFRSF1B, AKT3, MAP3K8, NFKB1
mmu04514	Cell adhesion molecules (CAMs)	0.380	NLGN2, ITGB7, CLDN7, CNTNAP1, VCAN, CLDN8, CNTN2, CD34, LRRC4B, L1CAM	SELP, CD2, LOC108391351, PTPRC, SELL, ITGAM, SDC4, PECAM1, CDH2
mmu04115	p53 signaling pathway	0.380	CHEK2, CCNB1, CCND1, TSC2, CDK2	PMAIP1, RPRM, SERPINE1, GADD45A, SESN1, AIFM2, IGFBP3
mmu04330	Notch signaling pathway	0.380	DTX4, JAG2, KAT2A, DLL1, NOTCH1, NOTCH3, DTX1, JAG1	KAT2B, ATXN1L
mmu04630	JAK-STAT signaling pathway	0.420	PDGFB, CCND1	MCL1, STAT3, SOCS3, OSMR, CSF3R, CSF3, CSF2RB, AOX1, AKT3
mmu03320	PPAR signaling pathway	0.460	RXRG, PLIN5, HMGCS2, ILK	ACSL4, MMP1, SORBS1, PLTP, PLIN2
mmu04210	Apoptosis	0.760	SEPT4, NGF, SPTAN1, LMNB2	PMAIP1, MCL1, BIRC3, CTSS, KRAS, CHUK, AKT3, NFKB1
mmu04010	MAPK signaling pathway	0.773	FGFR4, MAP3K6, PDGFB, NTF3, MAP4K2, FGF22, IGF2, FGFR1, FGF17, NGF, FGFR2	CD14, SRF, PRKACB, FGF1, KRAS, CHUK, AKT3, ELK4, DUSP3, ANGPT2, NFKB1
mmu04310	Wnt signaling pathway	0.936	SFRP2, TCF7, ROR2, LEF1, PRICKLE2, SFRP4, WNT8B, NOTUM, AXIN2, RSPO3, LGR5, CCND1, WNT10B, WNT4	FOSL1, PRKACB
mmu04530	Tight junction	1.000	CLDN7, TJP3, CLDN8, CCND1, MYH7B, MICALL2, ARHGEF2, MYL9	PRKACB, ACTR2, YBX3, PRKAB2, SYNPO
mmu04110	Cell cycle	1.000	CHEK2, ESPL1, CCNB1, PLK1, CDC45, CCND1, PTTG1, CDK2	GADD45A
mmu04350	TGF-beta signaling pathway	1.000	FMOD, RGMB, AMH, FBN3, ID3, TGIF2	INHBB
mmu04012	ErbB signaling pathway	1.000	PLCG1, PIK3R2	KRAS, AKT3
mmu04390	Hippo signaling pathway	1.000	TCF7, GLI2, LEF1, AMH, WNT8B, AXIN2, CCND1, WNT10B, WNT4, CRB2,	SERPINE1, FGF1, TEAD4

We conducted DEPs analysis before and after scale development (embryo-scale vs adult-scale group) based on our proteome data. In total, 54 DEPs were identified, of which 17 proteins were shared with DEGs in the transcriptome (Supplementary Table 6). According to the gene description, 3 proteins (COL16A1, RAB21, and MAPRE1) were related to cell spreading, alterations in cell morphology, and cell adhesion, respectively.

### DEGs related to skin appendage differentiation of *M. javanica*

To explore genes controlling the differentiation of skin appendages in pangolin, we compared the gene expression profiles in skin appendages between adult-hair and adult-scale group. Differential expression analysis identified 1,825 up-regulated genes in adult-hair group and 2,486 up-regulated genes in adult-scale group (Supplementary Table 7). According to the gene description, we screened 8 genes related to cell differentiation (MYOD1, PPDPF, COPRS, MYADML2, GDAP1, MYADML2, MAL, and MALL), 7 genes related to cell proliferation (PPDPF, MTCP1, SIPA1L2, SIPA1L3, BTG1, BTG3, and MKI67), 8 genes related to cell apoptosis (AIFM1, AIFM2, BFAR, BCL2, CCAR1, DDIAS, LOC108396282, and LOC108384891), 5 genes related to cell development (NDE1, LMBR1, DRG1, MESDC2, and novel.887), and 17 genes related to keratin (KRT8, 14, 32, 36, 38, etc.). These genes might be indicative of pangolin transcriptome involvement in scale and hair development and differentiation.

**Figure 4:**
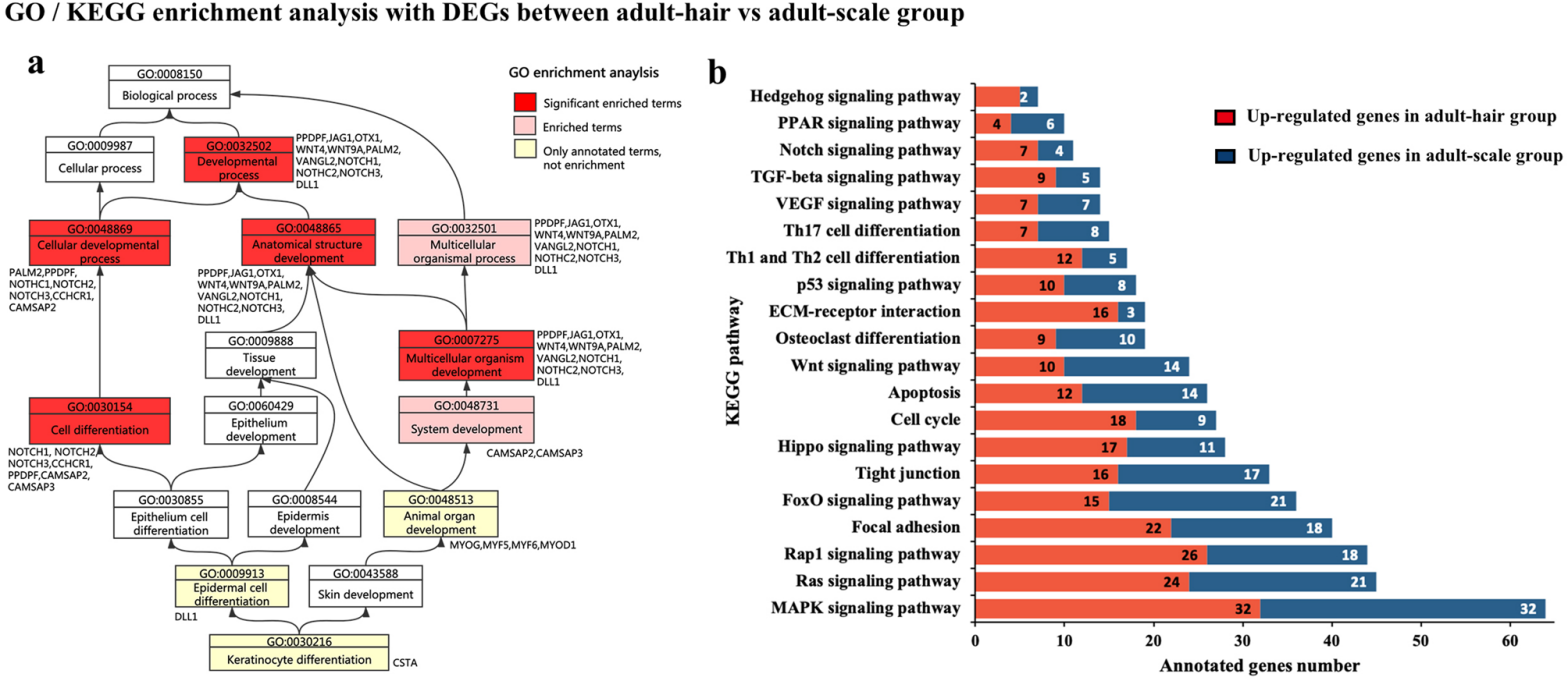
GO and KEGG enrichment analysis of DEGs between adult-hair vs adult-scale group. **(a**) Directed acyclic graph of GO enrichment analysis associated with cell differentiation; (**b**) Signalling pathways related to development and differentiation of skin appendages in KEGG enrichment analysis.

Annotation of the *M. javanica* transcripts with the GO database revealed that the up-regulated DEGs in adult-hair group were significantly enriched in terms related to skin appendage differentiation such as cell adhesion, cell differentiation, biological adhesion, etc. However, the up-regulated DEGs in adult-scale group were significantly enriched in terms related to protein complex, protein catabolic process, transferase activity, etc. In one directed acyclic graph of GO enrichment, the terms cell differentiation, developmental process, anatomical structure development, and multicellular organism development were significantly enriched; the main DEGs involved in these processes included PPDPF, JAG1, CSTA, DLL1, NOTCH1, etc. (Fig. 4a). The KEGG enrichment analysis revealed a total of 35 up-regulated and 79 down-regulated genes in adult-hair group of 20 unique KEGG pathways related to hair development and the differentiation process, including ECM-receptor interaction, proteasome, FoxO, MAPK, NOTCH, P53, HIPPO pathway, etc. (Fig. 4b; Supplementary Table 8). It is noteworthy that the Notch signalling pathway and PPDPF, DLL1, JAG1, VANGL2, WNT9A, LOC108401680, NOTCH1, and NOTCH3 gene were found in both annotation results, and are closely related to the differentiation of keratinocytes and epidermal cells.

**Figure 5:**
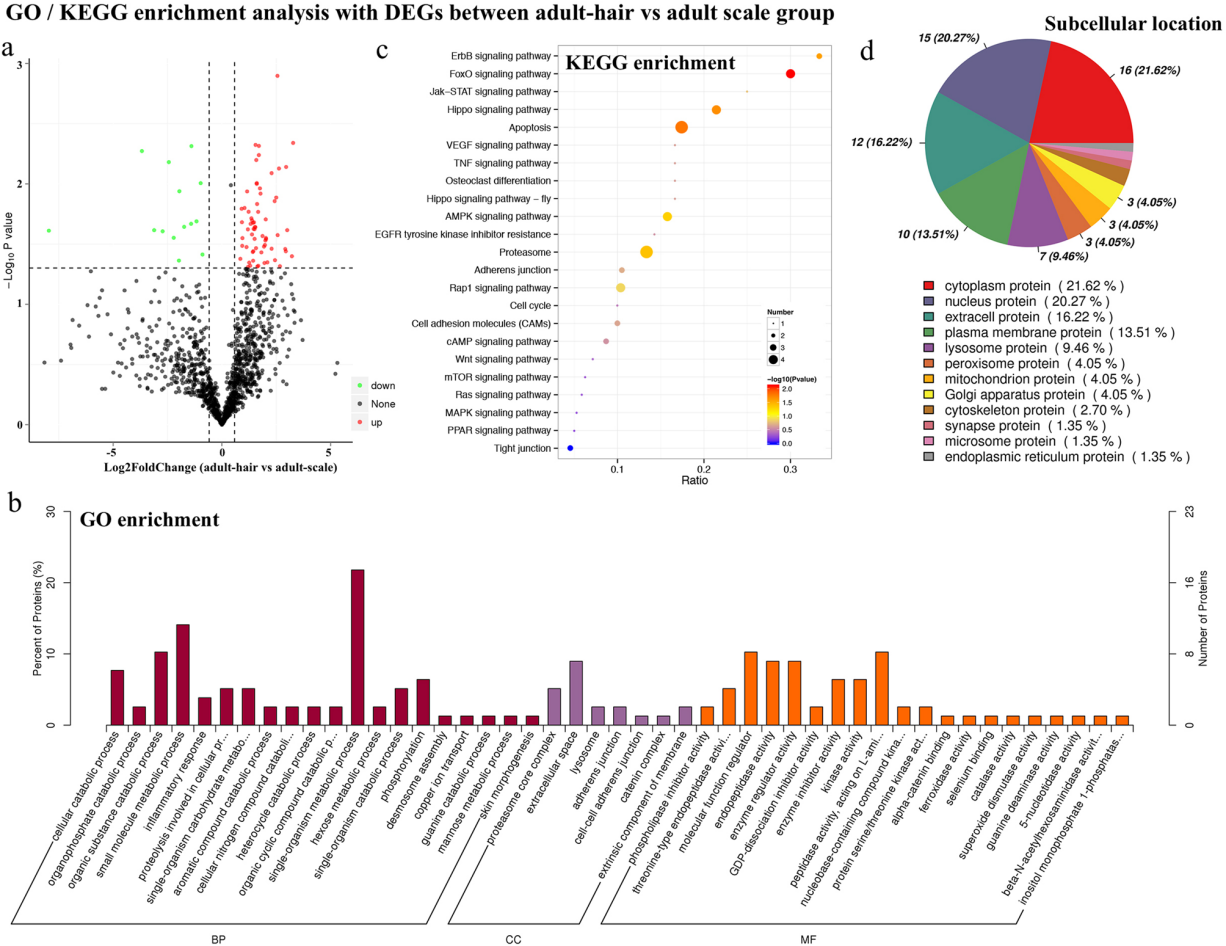
Distinct expressed proteins (DEPs) analysis between adult-hair vs adult-scale group. (**a**) Volcano map of DEPs showing the threshold line of the screening criteria; (**b**) GO enrichment analysis, *BP* biological process, *CC* cellular component, *MF* molecular function.; (**c**) Pathway related to skin appendage development and differentiation of KEGG enrichment analysis; (**d**) Differential protein subcellular localisation analysis.

According to our proteome data, differential proteome analysis identified 91 distinct significant proteins, including 73 up-regulated proteins in adult-hair group and 18 up-regulated proteins in adult-scale tissues (Fig. 5a). In the GO enrichment analysis with all DEPs, the CDSN gene was enriched in the skin morphogenesis term, which is also involved in biological processes related to skin development and keratinocyte differentiation. JUP, DSC1, CDH1, and TLN2 genes were enriched in cell adhesion and adherens junction terms, which were also involved in cell migration and regulation of cell population proliferation (Fig. 5b). In the KEGG enrichment analysis with all DEPs, 41 proteins in 21 pathways were related to the development and differentiation of skin appendages (Fig. 5c). We also analysed the subcellular localisation of all distinct proteins and found that the proportion of extracellular proteins was the highest (26.5%), followed by cytoplasmic proteins (17.7%) (Fig. 5d).

**Figure 6:**
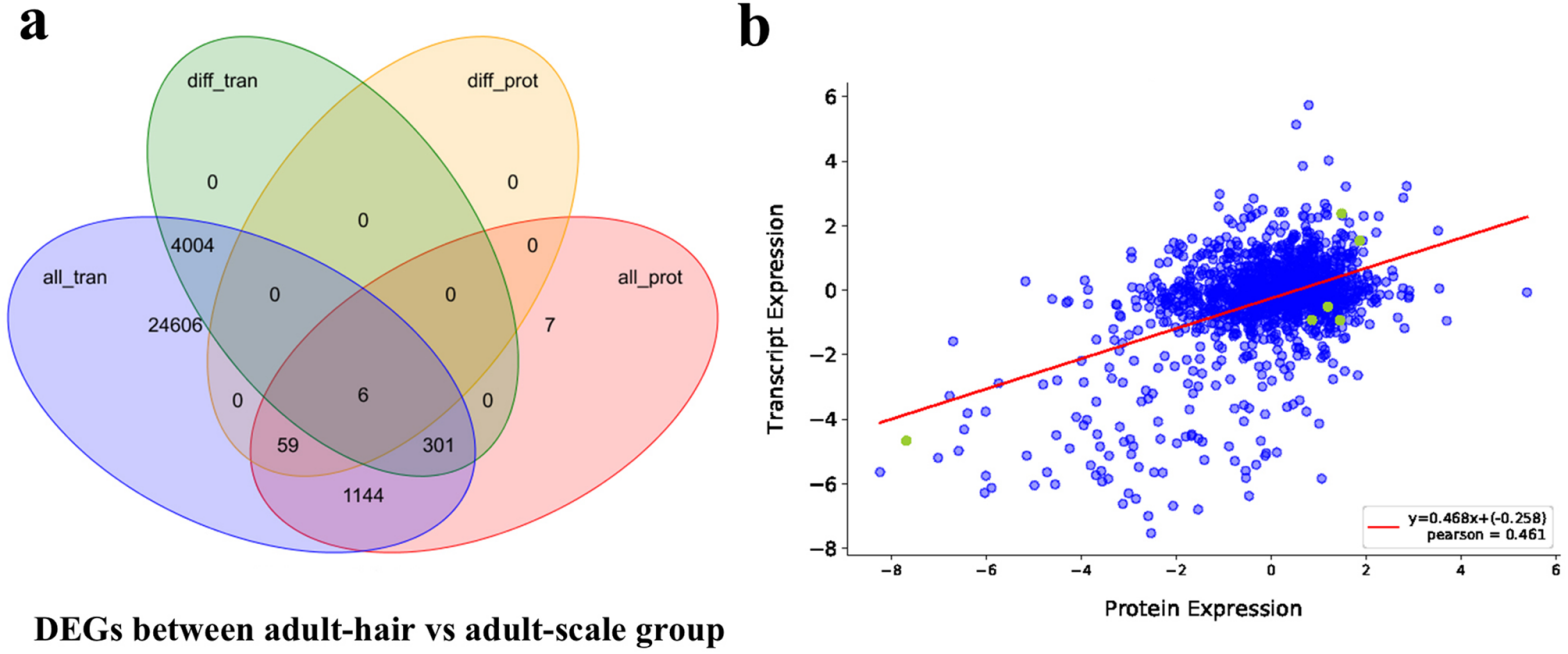
Combined analysis of transcriptome and proteome. (**a**) Expression of transcriptome and proteome regulates the Venn diagram, all_tran, all the transcripts in our transcriptome data; diff_tran, differentially expressed transcripts between adult-hair and adult-scale; diff_prot, distinct expressed proteins between adult-hair and adult-scale; all_prot, all the proteins in our proteome data; (**b**) Correlation analysis of transcriptome and proteome expression levels.

The mRNA information obtained from the transcriptome was integrated with the protein information identified by the proteome to identify any corresponding relationships. A total of 1,144 IDs were identified by both RNA-seq and proteomics (Fig. 6a). There were 6 distinct proteins corresponding to transcriptome DEGs: CSTA, XDH, PSMC5, KLK10, VCP, and MYOZ1. The correlation between the different multiples of genes (proteins) identified by transcriptome and proteome analysis in the two groups was analysed (Fig. 6b). The Pearson correlation coefficient between mRNA and the corresponding protein was positive (Pearson = 0.461). Thus, we conclude that it is important to determine the protein level to understand phenotypic changes and to not rely solely on mRNA levels.

### Immune-related genes in the epidermis of *M. javanica*

**Figure 7:**
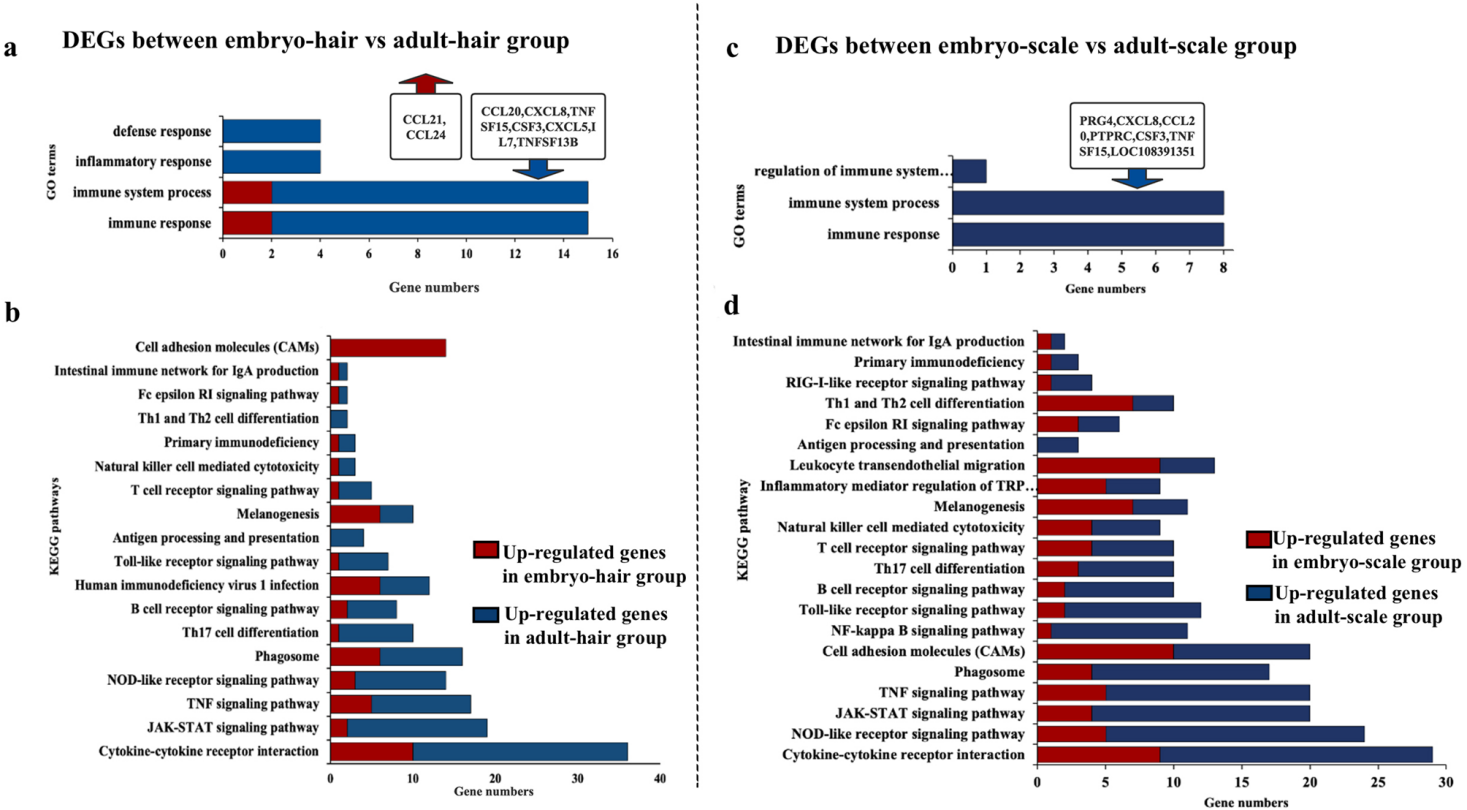
GO and KEGG enrichment analysis of DEGs related to immunity between embryo and adult skin tissue. (**a**) GO terms DEGs between embryo-hair vs adult-hair group in GO enrichment; (**b**) KEGG signalling pathways related to immunity with DEGs between embryo-hair vs adult-hair group; (**c**) GO terms DEGs between embryo-scale vs adult-scale group in GO enrichment; (**d**) KEGG signalling pathways related to immunity with DEGs between embryo-scale vs adult-scale group.

In this study, many immune-related genes were also identified in tissues of skin appendages from different stages of development in pangolin. Through GO enrichment analysis, the immune-related DEGs before and after hair growth (embryo-hair vs. adult-hair group), 34 up-regulated genes and 4 immune-related biological processes were identified as enriched in adult-hair group. These included defence response, inflammatory response, immune system, and immune response, while only 4 immune-related up-regulated genes and two terms were enriched in embryo-hair group (Fig. 7a). The results obtained from KEGG enrichment analysis were similar to those of the GO enrichment analysis. Compared with the embryonic samples (embryo-hair: 15 signalling pathways and 61 immune genes), more immune-related pathways and genes (adult-hair: 17 signalling pathways and 123 immune genes) were enriched in the adult-hair group (Fig. 7b).

Through GO enrichment analysis with all DEGs, before and after scale growth (embryo-scale vs. adult-scale group), we found that 17 up-regulated genes and 3 immune-related biological processes were enriched in adult-scale group. These included regulation of the immune system process, immune system, and immune response, while there was no enrichment of terms related to the immune response in embryo-scale group (Fig. 7c). The results obtained from KEGG enrichment analysis were similar to those of the GO enrichment analysis. Compared with the embryonic samples (embryo-scale: 15 signalling pathways and 61 immune genes), more immune-related pathways and genes (adult-scale: 17 signalling pathways and 123 immune genes) were enriched in the adult-scale tissue (Fig. 7d).

**Figure 8:**
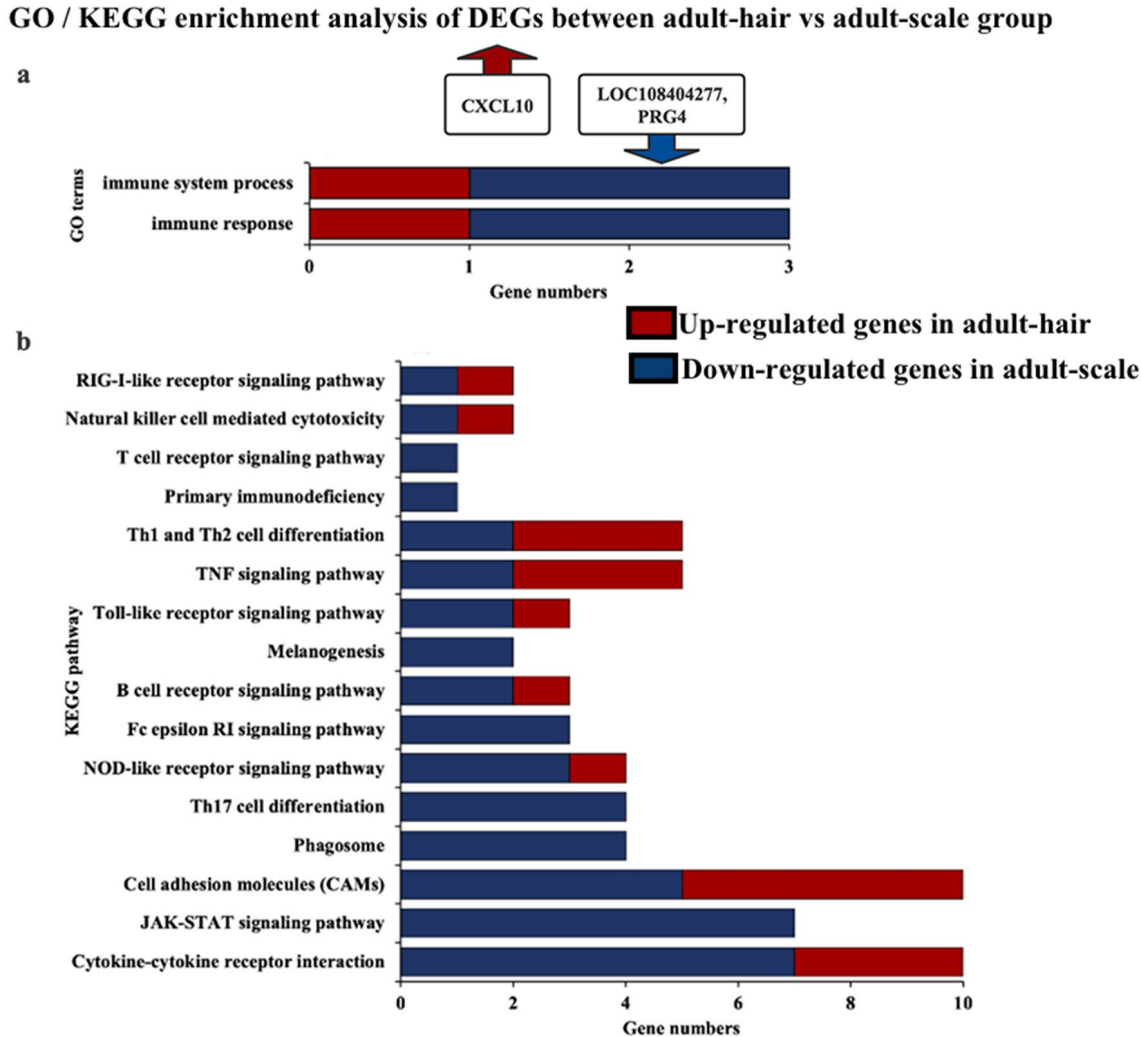
GO and KEGG enrichment analysis of DEGs related to immunity between hair-type and spine-type tissue. (**a**) GO terms related to immunity DEGs between adult-hair vs adult-scale group in GO enrichment; (**b**) KEGG signalling pathways related to immunity with DEGs between adult-hair vs adult-scale group.

We also compared and analysed the DEGs related to immunity in the dorsal and abdominal skin samples (adult-hair vs. adult-scale group) of adult pangolins. After GO enrichment analysis with all DEGs, both tissue types were found to be enriched in 2 terms related to immunity (immune system process and immune response), while the number of genes related to immunity and enriched in scale-type tissues (4 genes) was higher than that in hair-type tissues (2 genes) (Fig. 8a). In addition, we observed similar KEGG enrichment analysis results. Scale-type tissues had more immune-related pathways and genes than that found in hair-type tissues (Fig. 8b). Scale-type tissues exhibited 16 enriched immune-related pathways and 47 immune-related genes. The number of terms and genes enriched in hair-type tissues was significantly fewer than in scale-type tissues.

## Discussion

This study provides the most detailed poly-omic analysis of pangolin skin to date, characterising the developmental and differential gene expression in a special mammal. Thus, we have applied both label-free quantitative proteomics and RNA-seq analyses to globally monitor changes in protein and mRNA levels in response to differentiation in both scale-type and hair-type skin. Consistent with previous findings^21^, we also found only a moderate correlation between protein and corresponding mRNA levels. This means that the protein expression of pangolins is determined by the level of transcription and is also influenced by post-transcription conditions. The result of these responsible multiple regulatory pathways is that mRNA and protein levels are negatively correlated to the response of some proteins to differentiation.

During the development of mammalian skin appendages, a large number of regulatory genes and molecular signalling pathways are involved, affecting the establishment of complex morphologies between the epidermis and the mesenchymal^22^. Specifically, different species of organisms can evolve a wide variety of hair types because certain regulatory genes and signalling pathways perform specific functions in different space and time^23^. Interactions between epithelial and mesenchymal tissues determine the morphological characteristics, size, and polarity of different skin appendages^24^.

Pangolin scales, which are soft on newborn pangolins but harden as the animal matures, are formed by keratins^8^, guaranteeing a high abrasive wear resistance^9^. Among the differentially expressed genes in the transcriptome, we found 17 keratin-related genes, and an up-regulated KRT2 protein was found in the differentially expressed proteins in the proteome. This is consistent with Cadieu et al. (2009) who reported that a combination of RSPO2 and keratin genes causes hair to become coarse and stiff in the developmental stage^13^.

In the KEGG enrichment analysis, 36 DEGs (adult-hair vs adult-scale group) were significantly enriched in the FoxO signalling pathway. The fork head box O (FOXO) family of transcription factors regulates the expression of genes in cellular physiological events including apoptosis, cell-cycle control, glucose metabolism, oxidative stress resistance, and longevity. In our data, TGFB2, TGFB3, MAPK11, EGFR, CNND1, BCL6, and TNFSF10 genes were identified as up-regulated DEGs in scale-type tissue. We hypothesise that these genes are involved in skin appendage development and follicle size. Jackowska et al. (2012) found that an increased TGFB1, TGFB2, and TGFB3 protein pattern and expression level in oocytes of large compared to small follicles^25^. These findings are consistent with our results here.

In total, 89 DEGs (adult-hair vs adult-scale group) were enriched in MAPK and the cell cycle signalling pathway, which often work together in forming signalling loops in organogenesis^26^. They induce the most important fundamental biological process, the formation of periodic patterns. FGF, DUSP, VEGFA, EGF, and CDK6 have been identified as up-regulated genes in scale-type tissue. Hebert et al. (1994) demonstrated that the FGF gene plays an important role in hair cycle growth regulation, which functions as an inhibitor of hair elongation by promoting progression from anagen (the growth phase of the hair follicle) to catagen (the apoptosis-induced regression phase)^18^. Because of this, we believe that these genes may be important regulatory genes that affect the development and differentiation of scales.

Many of the genes we detected that vary in abundance in different skin types are involved in the Notch signalling pathway. These include CSTA, CDSN, PPDPF, DLL1, JAG1, VANGL2, WNT9A, LOC108401680, NOTCH1, and NOTCH3 genes, which were found in both KEGG and GO annotation results and are closely related to the differentiation of keratinocytes and epidermal cells. In particular, the up-regulated CSTA gene in adult-scale group was one of 6 DEGs in the transcriptome and proteome in the multi-omics analysis.

Through gene annotation, we not only found many genes related to the differentiation of skin appendages, but we also identified many genes and pathways related to diseases and immunity. Firstly, in the embryonic stage, a large number of genes and proteins related to immune function were detected in different skin regions, indicating that pangolins have a sound skin innate immune system. With the development and growth of an organism, its skin immune system tends to gradually improve, and the signalling pathways and regulatory genes involved in immune function become more complex. The immune function of the adult is more perfect than that of the embryo. Secondly, more immune-related genes and pathways were enriched in the back skin of the growing scales, indicating that the body may have some regulatory differences in the immune function of different skin regions. Thus, since the skin with the scales on the back looks smoother and thinner and more vulnerable to bacterial infection or other invasions, it appears that the body has differentially adapted to specifically protect this area.

Previous studies have found that some key genes related to immune function in the innate immune system of pangolins are pseudogenized or mutated, resulting in the loss of their immune pathways and functions involved in resistance to viral or bacterial invasion, and it is speculated that pangolins may have some undiscovered immune replacement compensation mechanisms^27,28^. However, we do not agree with some scholars who suggest that the evolution of pangolin scales is an innovative compensation for low immune function of the back skin^7^. This is because our data, to date, show that although a few immune genes (interferon) have been pseudogenised, a large number of complex immune-related signalling pathways and their gene participants normally performing immune functions can still be detected in the skin samples of pangolins. Therefore, we are more inclined to speculate that scale evolution of pangolin is more of an adaptive response to its living environment, enabling it to better adapt to burrowing, especially burrowing in other habits. Furthermore, scale evolution enables pangolin to walk better and more freely in caves and better protect the body from the wear of sand and stones. The gradual decline of the pangolin population is not due to its own low immunity, but it is more likely due to the irresistible hunting and habitat destruction by humans.

In summary, this study combined proteomics and transcriptomics to provide a comprehensive analysis of skin scale development and differentiation in pangolins. Our data highlight the correlation between the combined analysis of protein and transcript levels to understand the mechanisms that regulate gene expression in complex phenotypic responses. We expect that the data set reported in this study will provide a valuable resource for the scientific community and will facilitate future experimental design to study the development, differentiation, and even evolution of pangolin skin appendages as an adaptation to their environment.

## Conclusions

In this study, we applied transcriptome and proteome analysis of a pangolin species to explore the genetic basis of the development and differentiation of skin appendages. We have shown that the skin of pangolins has a healthy immune function. Our overall transcriptome and proteome analysis provided a rich list of genes and proteins expressed in hair-type and scale-type tissue of *M. javanica*. In total, 4,311 DEGs and 91 DEPs were screened from two types of skin appendages. Candidate genes related to 20 key signalling pathways are likely involved in regulating the growth and differentiation of the hair and scales. These molecular and signalling pathways greatly contribute to current genetic resources for pangolin and certain mammalian species with shaggy appendages and may help to understand human disease related to skin.

## Materials and methods

### Biological samples

Fourteen skin samples from the dorsolateral (hair-type) and the abdominal (scale-type) regions of *M. javanica* were collected from Guangdong Provincial Wildlife Rescue Centre. These individuals came from a wild population intercepted by customs and died of natural causes in the process of rescue. Pangolin hair and scale tissues were collected from 2 embryos (embryo-hair/scale) and 5 adult (adult-hair/scale) specimens, these samples included a small amount of epidermis and its attached skin appendages. 14 skin tissues representing the two types of appendages (7 hair-type and 7 scale-type) were rapidly excised, immediately snap-frozen on dry ice, and stored at − 80 °C until RNA extraction.

### RNA extraction and RNA-seq

Total RNA from each tissue sample was extracted using the RNeasy Kit (Qiagen, Germany). RNA purity was checked using a NanoPhotometer spectrophotometer (IMPLEN, CA, USA). RNA integrity was assessed using the RNA Nano 6000 assay kit of the Bioanalyzer 2,100 system (Agilent Technologies, CA, USA). RNA concentration (ng/μl) was determined using the Qubit RNA assay kit and Qubit 2.0 Fluorometer (Life Technologies, CA, USA). Qualified RNAs were used for cDNA library construction and sequencing^29^. To examine the similarity between the different organ transcriptomes, the expression levels of the transcripts (FPKM) in the transcriptomes of each tissue were manipulated using the tool ‘RSEM-calculate-expression’ in the RSEM pipeline (https://deweylab.biostat.wisc.edu/rsem/README.html), which performs accurate transcript quantification from RNA-Seq data with a reference genome^30–32^. All of the sequencing stages and methods were conducted by Novogene Sequencing Company (Beijing, China).

### Total protein extraction

Tissue samples were ground individually in liquid nitrogen and lysed with lysis buffer containing 100 mM NH_4_HCO_3_ (pH 8), 6 M urea and 0.2% SDS, followed by 5 min of ultrasonication on ice. The lysate was centrifuged at 12,000 g for 15 min at 4 °C and the supernatant was transferred to a clean tube. Extracts from each sample were reduced with 10 mM DTT for 1 h at 56 °C, and subsequently alkylated with sufficient iodoacetamide for 1 h at room temperature in the dark. Samples were subsequently mixed with 4-times volume of precooled acetone by vortexing before incubating at − 20 °C for at least 2 h. Samples were then centrifuged and the precipitate was collected. After washing twice with cold acetone, the pellet was dissolved in dissolution buffer containing 0.1 M triethylammonium bicarbonate (TEAB, pH 8.5) and 6 M urea^33–35^.

### Label-free quantitative protein analysis

The resulting spectra from each fraction were searched separately against X101SC19091078-Z01.fasta (41,843 sequences) database using the search engine Proteome Discoverer 2.2 (PD 2.2, Thermo). The search parameters were set as follows: mass tolerance for precursor ion was 10 ppm and mass tolerance for product ion was 0.02 Da. Carbamidomethyl was specified in PD 2.2 as a fixed modification. Oxidation of methionine (M) and acetylation of the N-terminus were specified in PD 2.2 as variable modifications. A maximum of two missed cleavage sites was allowed.

Identified proteins contained at least one unique peptide with FDR no more than 1.0%. Proteins containing similar peptides that could not be distinguished by mass spectrometry (MS)/MS analysis were identified as the same protein group. Precursor ion was quantified by the label-free method based on intensity and was used for label-free quantification. The protein quantitation results were statistically analysed by the Mann–Whitney Test. Proteins whose quantitation significantly differed between experimental and control groups (p < 0.05 and |log2FC|> 1.5 were up-regulated proteins p < 0.05 and |log2FC|≤ 0.67 were down-regulated proteins) were defined as differentially expressed proteins (DEPs).

Gene Ontology (GO) and InterPro (IPR) analysis were conducted using the InterProScan-5 program against the non-redundant protein database (including Pfam, PRINTS, ProDom, SMART, ProSiteProfiles, PANTHER)^36^, and the databases COG (Clusters of Orthologous Groups) and KEGG (Kyoto Encyclopedia of Genes and Genomes) were used to analyse the protein family and pathway. The probable protein–protein interactions were predicted using the STRING-db server^37^ (https://string.embl.de/). The enrichment pipeline^38^ was used for enrichment analysis of GO, IPR, and KEGG. All sequencing stages were conducted at Novogene Sequencing Company (Beijing, China).

### Ethics statement

All animal procedures in this study were approved by the ethics committee for animal experiments at the Guangdong Institute of Applied Biological Resources (reference number GIABR20170523) and followed basic principles. We confirm that all methods were performed in accordance with the relevant guidelines and regulations.

### Ethics approval and consent to participate

All animal procedures were approved by the ethics committee for animal experiments at the Guangdong Institute of Applied Biological Resources (reference number GIABR20170523).

## Supplementary information

Supplementary information

## Data Availability

Data analyzed in the current study are included within the article and its supplementary material. All unigene sequences from *M. javanica* have been deposited in the GenBank Sequence Read Archive (SRA) under accession number PRJNA610466 for SUB7099543. We have uploaded supplemental material to figshare via the GSA Portal.

## Abbreviations

*M. javanica*: *Mains javanica*
Hiseq: High-throughput sequencing
PCA: Principal component analysis
KRT: Keratin
FGF: Fibroblast growth factor
BMP: Bone morphogenetic protein
DEGs: Differentially expressed genes
DEPs: Distinct expressed proteins
SOM: Self-organizing feature map
