# Supplementary material for: Combined proteomics and transcriptomics reveal the genetic basis underlying the differentiation of skin appendages and immunity in pangolin
Source: Sci Rep. 2020 Sep 3;10:14566. doi: 10.1038/s41598-020-71513-w (PMC7471334; doi:10.1038/s41598-020-71513-w)
Supplement: Supplementary file 1 — Supplementary information [file 41598_2020_71513_MOESM1_ESM.pdf]

Combined proteomics and transcriptomics reveal the genetic basis underlying the differentiation of skin appendages and immunity in pangolin

Hui-Ming Li[1], Ping-Liu[1], Xiu-Juan Zhang[1], Lin-Miao Li[1], Hai-Ying Jiang[1], Hua Yan[2], Fanghui Hou[3] & Jin-Ping Chen[1*]

[1]Guangdong Key Laboratory of Animal Conservation and Resource Utilization, Guangdong Public Laboratory of Wild Animal Conservation and Utilization, Guangdong Institute of Applied Biological Resources, Guangdong Academy of Science, Guangzhou 510260, China

[2]Guangdong Provincial Key Laboratory of Silviculture, Protection and Utilization, Guangdong Academy of Forestry, Guangzhou, Guangdong Province

[3]Provincial Wildlife Rescue Center, Guangzhou, Guangdong Province, China

*Corresponding author: chenjp@giabr.gd.cn

Supplementary Table 1. The information of identified new genes in this study

Gene ID	Gene_chr	Gene_start	Gene_end	Gene_strand	Gene_length
novel.3049	NW_016535409.1	248456	248864	+	360
novel.3048	NW_016535409.1	215795	221340	+	309
novel.5445	NW_016545110.1	734390	736394	+	1153
novel.3043	NW_016535392.1	156711	159636	+	206
novel.3042	NW_016535389.1	103717	104583	-	234
novel.3041	NW_016535389.1	88871	93739	-	1454
novel.3040	NW_016535389.1	33251	33787	+	537
novel.3047	NW_016535407.1	39478	40876	+	980
novel.3046	NW_016535406.1	82418	83688	+	261
novel.3045	NW_016535405.1	69046	72330	+	578
novel.3044	NW_016535393.1	23575	23931	-	308
novel.1637	NW_016531014.1	43936	44253	-	275
novel.4235	NW_016539521.1	63372	63997	-	569
novel.4234	NW_016539521.1	90904	93334	+	806
novel.4237	NW_016539521.1	90661	90969	-	256
novel.4236	NW_016539521.1	64297	64776	-	364
novel.4231	NW_016539516.1	4174	4575	+	318
novel.4230	NW_016539510.1	200527	201654	+	293
novel.4233	NW_016539521.1	51685	52337	+	596
novel.4232	NW_016539518.1	279320	288720	+	416
novel.8745	NW_016606035.1	3389	4162	-	458
novel.8744	NW_016606034.1	1017	2400	-	773
novel.8747	NW_016606131.1	1	696	-	696
novel.8746	NW_016606108.1	192	3046	-	292
novel.8741	NW_016605918.1	590	4942	+	1262
novel.4238	NW_016539521.1	92476	93885	-	1212
novel.8743	NW_016606019.1	229	1592	-	1364
novel.8742	NW_016605924.1	1	477	-	477
novel.3959	NW_016538363.1	147173	147689	-	517
novel.3958	NW_016538363.1	26872	262167	+	2953
novel.3229	NW_016535878.1	3998	7460	-	2303
novel.3228	NW_016535877.1	1535	5360	+	3775
novel.3227	NW_016535874.1	30837	48216	+	658
novel.3226	NW_016535866.1	123669	160835	+	800
novel.3225	NW_016535864.1	1673	46191	-	335
novel.3224	NW_016535864.1	473319	473944	+	203
novel.3223	NW_016535859.1	38068	38797	-	521
novel.3222	NW_016535859.1	32697	34089	-	455
novel.3221	NW_016535854.1	6524	6815	-	236
novel.3220	NW_016535849.1	141451	143027	-	1555
novel.2798	NW_016534388.1	94353	94922	-	517

novel.2799	NW_016534392.1	8202	8647	+	381
novel.2790	NW_016534323.1	276804	277136	+	333
novel.2791	NW_016534350.1	383691	384348	+	603
novel.2792	NW_016534367.1	233816	234039	+	224
novel.2793	NW_016534368.1	7423	12771	-	614
novel.2794	NW_016534369.1	263004	263298	+	295
novel.2795	NW_016534369.1	28604	40054	-	4146
novel.2796	NW_016534370.1	381501	381787	+	287
novel.2797	NW_016534378.1	7569	17338	-	2373
novel.1781	NW_016531420.1	388155	389415	+	811
novel.2578	NW_016533775.1	208293	209818	+	1045
novel.2579	NW_016533775.1	340021	341127	+	401
novel.2574	NW_016533767.1	276819	278770	-	1882
novel.2575	NW_016533773.1	196273	197087	+	768
novel.2576	NW_016533775.1	82364	89620	+	228
novel.2577	NW_016533775.1	153882	155876	+	1995
novel.2570	NW_016533766.1	13575	22126	-	1978
novel.2571	NW_016533766.1	78120	79782	-	1608
novel.2572	NW_016533767.1	276817	277300	+	463
novel.2573	NW_016533767.1	258432	258740	-	259
novel.8235	NW_016586873.1	4199	7423	-	3170
novel.3476	NW_016536734.1	420190	420490	-	247
novel.3477	NW_016536752.1	75449	75890	+	392
novel.3474	NW_016536733.1	119256	129199	-	435
novel.3475	NW_016536734.1	502669	502998	+	276
novel.3472	NW_016536723.1	687	1152	-	248
novel.3473	NW_016536726.1	83161	86217	+	586
novel.3470	NW_016536720.1	182825	189040	-	608
novel.3471	NW_016536723.1	408	1652	-	268
novel.1179	NW_016529900.1	40802	48866	+	727
novel.1178	NW_016529899.1	4954	6071	-	299
novel.1175	NW_016529894.1	344488	375495	-	320
novel.1174	NW_016529889.1	119608	143974	+	1701
novel.1177	NW_016529898.1	5357	6063	-	554
novel.1176	NW_016529898.1	5753	6092	+	307
novel.1171	NW_016529888.1	732301	733843	+	533
novel.1170	NW_016529888.1	466595	466858	+	207
novel.1173	NW_016529888.1	445425	463600	-	2633
novel.1172	NW_016529888.1	751217	752091	+	875
novel.4420	NW_016540298.1	148662	148932	-	215
novel.4421	NW_016540300.1	10615	11159	+	294
novel.4422	NW_016540312.1	7729	9314	-	292
novel.4423	NW_016540318.1	2140	2901	+	463
novel.4424	NW_016540319.1	156104	157233	+	1089

novel.4425	NW_016540323.1	6848	12078	-	481
novel.4426	NW_016540328.1	17599	18659	+	1061
novel.4427	NW_016540329.1	128394	130839	+	1788
novel.4428	NW_016540329.1	128374	129598	-	926
novel.4429	NW_016540332.1	836733	839120	+	890
novel.3479	NW_016536760.1	182967	183323	+	357
novel.7038	NW_016558074.1	6770	8588	+	1241
novel.6682	NW_016554424.1	91860	92865	+	952
novel.6683	NW_016554424.1	90588	91699	-	1058
novel.6680	NW_016554403.1	54804	63368	+	328
novel.6681	NW_016554409.1	18383	19014	+	578
novel.6686	NW_016554482.1	17980	18205	+	226
novel.6687	NW_016554520.1	20881	22353	-	1319
novel.6684	NW_016554433.1	33186	55212	+	3967
novel.6685	NW_016554465.1	249	1122	-	641
novel.6688	NW_016554525.1	32418	32658	-	205
novel.6689	NW_016554548.1	16875	17522	+	430
novel.575	NW_016528495.1	1	426	+	426
novel.574	NW_016528491.1	361003	460175	-	349
novel.577	NW_016528500.1	682700	712773	-	270
novel.576	NW_016528496.1	10943	11260	+	262
novel.571	NW_016528488.1	224608	225347	-	246
novel.570	NW_016528488.1	243730	243961	+	202
novel.573	NW_016528491.1	378040	378367	+	272
novel.572	NW_016528489.1	1021	1863	-	843
novel.579	NW_016528501.1	467413	470204	+	273
novel.578	NW_016528500.1	816600	818181	-	1526
novel.1799	NW_016531455.1	102553	240902	-	5915
novel.1798	NW_016531455.1	63193	64626	-	1434
novel.1620	NW_016530968.1	150931	152136	+	660
novel.1791	NW_016531448.1	27162	29024	+	1829
novel.1790	NW_016531445.1	10756	12163	-	350
novel.1793	NW_016531448.1	35976	38988	-	3013
novel.1792	NW_016531448.1	57467	58125	+	302
novel.1795	NW_016531455.1	184312	186200	+	1833
novel.1794	NW_016531448.1	39041	40195	-	994
novel.1797	NW_016531455.1	240455	241455	+	822
novel.1796	NW_016531455.1	229222	230370	+	957
novel.5089	NW_016542996.1	212	804	+	378
novel.5088	NW_016542988.1	205986	207409	-	817
novel.1622	NW_016530993.1	616414	619651	+	3123
novel.5081	NW_016542931.1	290802	292743	-	1390
novel.5080	NW_016542931.1	289096	290711	-	1616
novel.5083	NW_016542943.1	150165	158427	+	682

novel.5082	NW_016542943.1	3426	37367	+	1651
novel.5085	NW_016542969.1	250373	260318	+	249
novel.5084	NW_016542956.1	78287	80087	+	1801
novel.5087	NW_016542983.1	43503	44313	-	761
novel.5086	NW_016542983.1	30616	33388	-	538
novel.1627	NW_016530999.1	77044	77437	-	341
novel.1626	NW_016530996.1	1249	1484	+	216
novel.7021	NW_016557922.1	41560	42016	+	290
novel.7020	NW_016557922.1	11287	20211	+	2379
novel.7023	NW_016557949.1	1338	1717	+	323
novel.7790	NW_016572367.1	23414	27011	+	3598
novel.7025	NW_016557956.1	734	1517	-	747
novel.7024	NW_016557951.1	252483	252864	+	346
novel.7027	NW_016557963.1	1912	7165	-	1242
novel.7794	NW_016572565.1	38855	49977	+	414
novel.7029	NW_016557970.1	14467	14732	+	266
novel.7028	NW_016557970.1	13400	14192	+	301
novel.7799	NW_016572684.1	11749	14158	+	1981
novel.7798	NW_016572678.1	133263	134999	-	1737
novel.8088	NW_016581847.1	817	1266	-	424
novel.8089	NW_016581855.1	1	874	+	852
novel.4849	NW_016541940.1	6240	9257	-	2501
novel.5333	NW_016544419.1	36380	36868	+	270
novel.5332	NW_016544417.1	6204	7546	+	1286
novel.5331	NW_016544400.1	66787	67011	-	225
novel.5330	NW_016544400.1	82802	85603	+	230
novel.5337	NW_016544478.1	16711	18759	+	1159
novel.5336	NW_016544463.1	58628	58927	+	218
novel.5335	NW_016544460.1	25615	25844	+	230
novel.5334	NW_016544444.1	21404	42958	+	422
novel.5328	NW_016544387.1	47712	48333	-	622
novel.5339	NW_016544479.1	7280	9572	+	328
novel.5338	NW_016544478.1	20384	20664	+	233
novel.6922	NW_016556864.1	33868	34937	-	1013
novel.8558	NW_016601041.1	239	1939	-	215
novel.8559	NW_016601061.1	1	1962	-	1962
novel.8228	NW_016586825.1	2800	3329	+	476
novel.8229	NW_016586825.1	1850	2096	-	247
novel.8222	NW_016586655.1	2475	2730	-	200
novel.8223	NW_016586715.1	1	202	+	202
novel.8220	NW_016586416.1	12273	12781	+	403
novel.8553	NW_016600802.1	1	1906	-	1906
novel.8554	NW_016600834.1	715	1881	+	597
novel.8555	NW_016600835.1	3	1809	-	1807

novel.8224	NW_016586726.1	5406	9255	+	553
novel.8557	NW_016600931.1	1020	1931	+	205
novel.7245	NW_016561462.1	1	670	+	613
novel.7244	NW_016561420.1	53	438	-	354
novel.7247	NW_016561462.1	13492	15714	-	210
novel.7246	NW_016561462.1	11	945	-	781
novel.7241	NW_016561303.1	125663	125967	+	258
novel.7240	NW_016561279.1	1074	1663	-	308
novel.7243	NW_016561409.1	1306	1897	+	564
novel.7242	NW_016561308.1	14851	16482	+	682
novel.4841	NW_016541896.1	9456	24716	-	642
novel.7249	NW_016561472.1	81832	82391	+	418
novel.7248	NW_016561468.1	2612	7091	-	2306
novel.5823	NW_016547542.1	489730	519024	-	1332
novel.5822	NW_016547538.1	126940	137068	-	896
novel.4239	NW_016539530.1	93671	94353	-	626
novel.5821	NW_016547512.1	4661	5073	-	368
novel.8740	NW_016605909.1	2900	3499	-	600
novel.5820	NW_016547486.1	56090	100526	-	1417
novel.5322	NW_016544336.1	21799	22089	-	235
novel.5827	NW_016547550.1	11665	15424	-	3760
novel.5323	NW_016544342.1	17920	19311	+	564
novel.5826	NW_016547550.1	438438	439220	+	783
novel.993	NW_016529538.1	4704	5103	+	400
novel.992	NW_016529537.1	152540	153942	+	458
novel.991	NW_016529537.1	130116	130644	+	529
novel.990	NW_016529535.1	101037	101756	-	513
novel.997	NW_016529551.1	17330	18820	+	1359
novel.996	NW_016529550.1	44734	48211	+	440
novel.995	NW_016529550.1	741	2551	+	1811
novel.994	NW_016529549.1	16251	22092	+	1912
novel.999	NW_016529553.1	10544	10894	-	351
novel.998	NW_016529551.1	17330	18820	-	1294
novel.157	NW_016527584.1	56617	58854	-	1577
novel.156	NW_016527584.1	46696	54350	-	254
novel.155	NW_016527577.1	12533	17423	-	4891
novel.3951	NW_016538343.1	18701	22301	-	3601
novel.154	NW_016527577.1	4	4318	-	600
novel.3950	NW_016538343.1	9999	22303	+	1480
novel.153	NW_016527575.1	78951	79283	-	205
novel.3953	NW_016538348.1	18293	18574	-	208
novel.152	NW_016527574.1	201219	253658	-	562
novel.3952	NW_016538348.1	14024	15263	+	1209
novel.151	NW_016527574.1	72992	73446	-	455

novel.3955	NW_016538353.1	3430	4757	+	1328
novel.150	NW_016527574.1	187489	253658	+	328
novel.3954	NW_016538349.1	37720	38130	-	365
novel.3568	NW_016537131.1	11530	12522	-	732
novel.3569	NW_016537131.1	84408	85879	-	230
novel.3957	NW_016538361.1	25189	25676	-	303
novel.3560	NW_016537125.1	456	1515	-	258
novel.3561	NW_016537126.1	77306	89985	+	220
novel.3562	NW_016537129.1	20171	24392	+	1066
novel.3956	NW_016538361.1	24954	25312	+	239
novel.6044	NW_016549187.1	2509	2953	-	445
novel.3565	NW_016537129.1	26897	27154	-	258
novel.3566	NW_016537131.1	151161	151744	+	302
novel.3567	NW_016537131.1	459107	459707	+	601
novel.7256	NW_016561577.1	6220	7701	-	1392
novel.7257	NW_016561577.1	8799	9501	-	295
novel.7254	NW_016561507.1	22696	30431	-	471
novel.7255	NW_016561522.1	26565	30892	-	506
novel.2969	NW_016535041.1	15167	15436	+	215
novel.2968	NW_016535039.1	402607	406627	-	245
novel.2967	NW_016535039.1	388091	393720	-	572
novel.2966	NW_016535039.1	150248	151505	-	1236
novel.2965	NW_016535039.1	457681	471387	+	2783
novel.2964	NW_016535039.1	421876	429165	+	250
novel.2963	NW_016535039.1	264918	271976	+	6177
novel.2962	NW_016535026.1	11591	12097	+	381
novel.2961	NW_016535014.1	29884	31410	+	226
novel.2960	NW_016535004.1	8840	9871	-	850
novel.7250	NW_016561472.1	87310	87913	-	422
novel.8562	NW_016601156.1	543	1959	-	364
novel.6307	NW_016550833.1	57526	58119	-	268
novel.6306	NW_016550833.1	57014	57382	-	369
novel.6305	NW_016550833.1	57526	57829	+	249
novel.6304	NW_016550832.1	2260	3760	-	735
novel.6303	NW_016550830.1	31416	32313	-	300
novel.6302	NW_016550811.1	4872	23528	-	591
novel.6301	NW_016550804.1	147263	148401	+	1088
novel.6300	NW_016550804.1	102667	103381	+	537
novel.6309	NW_016550883.1	8774	9515	-	742
novel.6308	NW_016550876.1	31319	31867	-	495
novel.3335	NW_016536252.1	355795	356748	-	954
novel.3334	NW_016536252.1	4979	6790	-	1782
novel.5539	NW_016545598.1	59284	59807	+	488
novel.5538	NW_016545591.1	323520	385202	-	952

novel.4371	NW_016540109.1	7192	7486	-	270
novel.2211	NW_016532634.1	17948	18512	+	565
novel.2210	NW_016532624.1	33981	34566	-	586
novel.2213	NW_016532634.1	64035	64580	-	495
novel.2212	NW_016532634.1	65832	66657	+	576
novel.2215	NW_016532639.1	65822	66071	+	202
novel.2214	NW_016532634.1	76202	76519	-	318
novel.2217	NW_016532640.1	20045	20564	+	520
novel.2216	NW_016532640.1	1	281	+	253
novel.1446	NW_016530524.1	354805	387105	+	1063
novel.1096	NW_016529748.1	13102	14519	-	980
novel.1097	NW_016529749.1	30446	39229	+	281
novel.1094	NW_016529733.1	333822	334972	+	226
novel.1095	NW_016529737.1	264063	264381	-	266
novel.1092	NW_016529723.1	285774	286522	+	451
novel.1093	NW_016529727.1	246377	247760	-	385
novel.1090	NW_016529719.1	510	1131	-	487
novel.1091	NW_016529719.1	120873	125399	-	4527
novel.1548	NW_016530752.1	1710	2326	-	593
novel.1549	NW_016530752.1	172813	175483	-	1711
novel.1098	NW_016529749.1	26577	29920	-	509
novel.1099	NW_016529749.1	86432	88134	-	809
novel.5654	NW_016546305.1	186683	187040	-	358
novel.5655	NW_016546326.1	71972	72623	+	507
novel.5656	NW_016546337.1	24059	25509	+	493
novel.5657	NW_016546337.1	25222	25495	-	254
novel.5650	NW_016546293.1	283131	283631	-	501
novel.5651	NW_016546305.1	59597	60600	+	954
novel.5652	NW_016546305.1	59718	61975	-	1167
novel.5653	NW_016546305.1	62392	63222	-	249
novel.1449	NW_016530529.1	569866	573984	-	247
novel.5658	NW_016546362.1	19431	23641	-	3268
novel.5659	NW_016546371.1	147302	148520	-	1199
novel.1448	NW_016530529.1	510177	541724	-	542
novel.7876	NW_016574679.1	5440	5741	+	245
novel.7877	NW_016574778.1	294	1030	-	224
novel.7874	NW_016574638.1	15265	16320	-	269
novel.7875	NW_016574638.1	17747	20591	-	2153
novel.7872	NW_016574638.1	9024	18899	+	2324
novel.7873	NW_016574638.1	49917	51631	+	1543
novel.7870	NW_016574554.1	5454	10060	+	920
novel.7871	NW_016574592.1	1527	1880	+	222
novel.7878	NW_016574817.1	1784	2659	+	842
novel.7879	NW_016574853.1	1	1154	-	1091

novel.1328	NW_016530196.1	178822	179199	-	358
novel.1329	NW_016530198.1	3085	50738	-	707
novel.1898	NW_016531704.1	683540	684332	-	739
novel.1899	NW_016531704.1	1075692	1076099	-	408
novel.1320	NW_016530196.1	78426	79020	+	565
novel.1321	NW_016530196.1	84166	85304	+	1089
novel.1322	NW_016530196.1	136690	137542	+	815
novel.1323	NW_016530196.1	137629	141667	+	3801
novel.1324	NW_016530196.1	84267	85303	-	992
novel.1325	NW_016530196.1	131203	154341	-	1193
novel.1326	NW_016530196.1	136591	137549	-	873
novel.1327	NW_016530196.1	137756	169936	-	1829
novel.748	NW_016528914.1	401971	438208	+	2291
novel.749	NW_016528914.1	514328	522688	+	520
novel.746	NW_016528893.1	139334	139757	+	424
novel.747	NW_016528904.1	161790	168163	+	259
novel.744	NW_016528885.1	4310	16856	-	419
novel.745	NW_016528892.1	26813	27164	+	295
novel.742	NW_016528884.1	540569	541896	-	1284
novel.743	NW_016528884.1	706047	712084	-	674
novel.740	NW_016528879.1	48452	53194	-	617
novel.741	NW_016528883.1	640465	641816	+	1352
novel.5470	NW_016545291.1	11468	27210	+	738
novel.5471	NW_016545291.1	28031	34758	+	258
novel.8352	NW_016591146.1	32350	36092	+	939
novel.3029	NW_016535316.1	2767	3089	+	272
novel.3028	NW_016535309.1	130774	131743	-	970
novel.3025	NW_016535293.1	3566	4175	-	561
novel.3024	NW_016535290.1	15307	15582	-	220
novel.3027	NW_016535294.1	6357	6802	-	446
novel.3026	NW_016535294.1	75693	76094	+	221
novel.3021	NW_016535287.1	5971	6569	+	599
novel.3020	NW_016535275.1	23399	23783	+	328
novel.3023	NW_016535287.1	10605	17493	+	3145
novel.3022	NW_016535287.1	8918	10475	+	1558
novel.4219	NW_016539387.1	61120	61418	-	244
novel.4218	NW_016539387.1	3514	3848	-	291
novel.4217	NW_016539384.1	147962	148354	-	249
novel.4216	NW_016539384.1	50288	51367	-	1033
novel.4215	NW_016539384.1	136430	136760	+	331
novel.4214	NW_016539382.1	25476	29398	+	1498
novel.4213	NW_016539371.1	5687	6029	+	287
novel.4212	NW_016539367.1	3856	4238	-	379
novel.4211	NW_016539367.1	125458	140526	+	293

novel.4210	NW_016539365.1	43366	43866	+	261
novel.7490	NW_016565913.1	11680	12328	-	531
novel.7491	NW_016565942.1	77457	78017	+	476
novel.7492	NW_016565942.1	16529	29745	-	515
novel.7493	NW_016565946.1	28548	28875	+	328
novel.7494	NW_016565946.1	2908	25881	-	7908
novel.7495	NW_016565979.1	15396	16494	+	1048
novel.7496	NW_016565989.1	3893	4341	+	397
novel.7497	NW_016566034.1	2304	2795	-	444
novel.7498	NW_016566069.1	10264	10980	+	717
novel.7499	NW_016566077.1	9203	27511	-	326
novel.8769	NW_016606633.1	5770	6432	+	301
novel.8768	NW_016606616.1	99	5418	-	451
novel.3973	NW_016538397.1	15391	25483	+	523
novel.3796	NW_016537794.1	35133	35427	-	250
novel.3795	NW_016537790.1	4966	5396	-	239
novel.3794	NW_016537786.1	370088	370614	+	320
novel.3977	NW_016538414.1	18288	19545	+	1201
novel.3792	NW_016537781.1	165072	169430	+	390
novel.3975	NW_016538412.1	423047	426195	-	500
novel.3790	NW_016537779.1	488818	493534	-	2037
novel.3979	NW_016538416.1	65339	67531	+	225
novel.3978	NW_016538416.1	55378	57967	+	2590
novel.3799	NW_016537806.1	95890	96258	-	319
novel.3798	NW_016537806.1	429889	430136	+	221
novel.8351	NW_016591106.1	105	1060	-	923
novel.2772	NW_016534296.1	8291	9906	+	1616
novel.2773	NW_016534296.1	44983	53400	+	1073
novel.2770	NW_016534292.1	344197	347431	-	1620
novel.2771	NW_016534295.1	97107	97527	+	421
novel.2776	NW_016534299.1	102446	105938	-	883
novel.2777	NW_016534300.1	494537	497619	+	499
novel.2774	NW_016534296.1	8518	9906	-	1389
novel.2775	NW_016534299.1	93769	101560	-	3220
novel.2778	NW_016534300.1	390846	392148	-	437
novel.2779	NW_016534300.1	407342	408335	-	994
novel.2516	NW_016533626.1	1	1178	-	1151
novel.2517	NW_016533628.1	147262	147596	+	335
novel.2514	NW_016533620.1	515213	521946	-	236
novel.2515	NW_016533626.1	313	2319	+	1873
novel.2512	NW_016533620.1	544434	545296	+	863
novel.2513	NW_016533620.1	511910	515101	-	1000
novel.2510	NW_016533617.1	31678	32004	-	280
novel.2511	NW_016533620.1	496432	539460	+	430

novel.2518	NW_016533630.1	27894	44060	+	1483
novel.2519	NW_016533630.1	139805	140901	+	235
novel.3332	NW_016536251.1	537	1055	-	519
novel.2431	NW_016533355.1	292563	292774	+	212
novel.4484	NW_016540573.1	989160	989728	+	290
novel.4485	NW_016540573.1	237403	238601	-	1199
novel.2430	NW_016533342.1	19042	19988	+	772
novel.4480	NW_016540549.1	549238	550956	+	868
novel.4481	NW_016540549.1	273603	275912	-	358
novel.7663	NW_016569085.1	2933	3294	-	362
novel.2433	NW_016533366.1	63212	66105	+	2894
novel.7662	NW_016569080.1	14652	18215	-	3425
novel.7661	NW_016569080.1	15133	18215	+	3026
novel.6512	NW_016552797.1	2425	5889	-	304
novel.6513	NW_016552804.1	55566	56022	+	353
novel.6510	NW_016552787.1	3948	5643	-	1639
novel.6511	NW_016552789.1	39727	40337	+	556
novel.6516	NW_016552898.1	10239	31933	+	937
novel.6517	NW_016552907.1	19294	19609	-	281
novel.6514	NW_016552843.1	154710	178039	+	2909
novel.6515	NW_016552843.1	3085	11999	-	1922
novel.7667	NW_016569234.1	80410	83892	-	2507
novel.6518	NW_016552922.1	155510	157001	+	1387
novel.6519	NW_016552922.1	175765	176095	-	275
novel.7666	NW_016569218.1	406	1134	-	695
novel.1157	NW_016529867.1	322981	323601	-	431
novel.1156	NW_016529865.1	28257	28540	-	284
novel.1155	NW_016529865.1	28049	28563	+	269
novel.1154	NW_016529858.1	2216	2681	+	247
novel.1153	NW_016529856.1	230824	233300	-	1874
novel.1152	NW_016529856.1	174570	175784	+	1158
novel.1151	NW_016529855.1	1932132	1968269	-	972
novel.1150	NW_016529855.1	440789	441039	-	251
novel.8131	NW_016583471.1	5140	6276	+	465
novel.1159	NW_016529868.1	10087	10643	-	557
novel.1158	NW_016529868.1	10504	11254	+	676
novel.2435	NW_016533367.1	22295	50550	+	497
novel.3555	NW_016537101.1	94706	96295	+	1541
novel.6293	NW_016550763.1	63308	65157	-	1360
novel.3554	NW_016537098.1	10652	27842	+	543
novel.6292	NW_016550763.1	980	5595	-	4616
novel.3557	NW_016537108.1	157097	181775	+	1321
novel.3556	NW_016537108.1	2765	5062	+	607
novel.3551	NW_016537083.1	2685	3166	+	250

novel.3252	NW_016535943.1	30272	37469	-	6955
novel.3550	NW_016537078.1	10223	10699	+	273
novel.7443	NW_016564953.1	10454	24775	+	2233
novel.3553	NW_016537090.1	154558	155578	-	1000
novel.7442	NW_016564913.1	11771	14270	+	2244
novel.2436	NW_016533368.1	25258	25510	-	217
novel.6299	NW_016550804.1	77652	145949	+	1257
novel.3552	NW_016537083.1	5892	6263	+	372
novel.7441	NW_016564859.1	1	610	+	549
novel.6298	NW_016550788.1	44351	45072	-	669
novel.7440	NW_016564790.1	832	1548	-	669
novel.7447	NW_016565075.1	441	877	-	397
novel.7446	NW_016565075.1	2898	4067	+	1170
novel.7009	NW_016557801.1	45299	47655	-	974
novel.7008	NW_016557787.1	23361	105180	-	2505
novel.7117	NW_016559215.1	8202	12819	+	2577
novel.7003	NW_016557727.1	209750	216371	-	2484
novel.7002	NW_016557727.1	82279	83277	+	942
novel.7001	NW_016557712.1	102136	102668	-	504
novel.7000	NW_016557712.1	100715	102037	-	1323
novel.7007	NW_016557758.1	130399	130668	+	244
novel.7006	NW_016557757.1	42636	46307	+	1075
novel.7005	NW_016557744.1	1093	1396	+	247
novel.7004	NW_016557727.1	277317	277768	-	452
novel.229	NW_016527737.1	66286	67407	+	1067
novel.228	NW_016527735.1	178783	179219	+	386
novel.559	NW_016528468.1	53804	54520	-	717
novel.558	NW_016528466.1	1016	2156	-	1090
novel.5351	NW_016544547.1	230	868	+	586
novel.5350	NW_016544542.1	9817	13730	+	287
novel.5353	NW_016544576.1	202199	202703	+	347
novel.5352	NW_016544548.1	13483	13813	-	275
novel.553	NW_016528460.1	55340	57136	-	1338
novel.552	NW_016528460.1	66540	69496	+	229
novel.551	NW_016528460.1	55218	55828	+	514
novel.222	NW_016527730.1	6484	11367	+	200
novel.557	NW_016528466.1	1016	2617	+	1573
novel.224	NW_016527730.1	121811	198200	+	266
novel.555	NW_016528462.1	104669	104910	-	204
novel.554	NW_016528461.1	491269	493369	+	2101
novel.8208	NW_016586202.1	269630	271141	-	403
novel.8209	NW_016586208.1	6918	8188	+	1231
novel.7269	NW_016561913.1	3512	4433	+	787
novel.7268	NW_016561899.1	800	1791	-	591

novel.7267	NW_016561899.1	116217	116684	+	261
novel.7266	NW_016561872.1	19702	20852	-	537
novel.7265	NW_016561786.1	3906	16674	-	1581
novel.7264	NW_016561772.1	3555	5668	-	1214
novel.7263	NW_016561755.1	4409	5023	-	567
novel.7262	NW_016561673.1	16214	18677	+	241
novel.7261	NW_016561673.1	15671	18467	+	471
novel.7260	NW_016561628.1	90508	94453	-	3946
novel.4259	NW_016539625.1	26160	28397	+	2238
novel.1724	NW_016531302.1	151594	152218	-	414
novel.1725	NW_016531306.1	5247	5805	+	534
novel.1726	NW_016531306.1	5492	6120	-	431
novel.1727	NW_016531308.1	164622	168450	+	550
novel.1720	NW_016531281.1	115386	116149	+	655
novel.1721	NW_016531289.1	480970	485649	+	4531
novel.4153	NW_016539172.1	3309	3762	+	400
novel.1722	NW_016531301.1	196	997	+	593
novel.1723	NW_016531302.1	14913	40340	+	298
novel.4155	NW_016539183.1	33	292	+	232
novel.1729	NW_016531310.1	161259	202038	-	436
novel.4008	NW_016538502.1	10364	49403	+	512
novel.4009	NW_016538502.1	49467	49860	+	221
novel.4002	NW_016538484.1	20848	24290	-	3443
novel.4003	NW_016538487.1	22604	23705	+	983
novel.4000	NW_016538482.1	1	806	+	806
novel.4001	NW_016538483.1	380208	380427	+	220
novel.4006	NW_016538487.1	50653	50938	-	230
novel.4007	NW_016538500.1	2652	13704	+	507
novel.4004	NW_016538487.1	22273	24608	-	2258
novel.4005	NW_016538487.1	27391	38982	-	1997
novel.3582	NW_016537144.1	20476	27378	-	1724
novel.3583	NW_016537145.1	22341	30225	+	3670
novel.3580	NW_016537138.1	67686	68241	-	516
novel.3581	NW_016537144.1	25245	25579	+	243
novel.3586	NW_016537152.1	6994	8660	+	1644
novel.3587	NW_016537152.1	13434	14059	+	606
novel.3584	NW_016537151.1	31289	79433	-	419
novel.8689	NW_016605051.1	56	960	+	419
novel.3588	NW_016537152.1	15366	22113	+	6460
novel.3589	NW_016537152.1	925	14059	-	1259
novel.2981	NW_016535137.1	508455	536926	+	491
novel.2980	NW_016535137.1	507784	508389	+	606
novel.2983	NW_016535137.1	494881	501566	-	664
novel.2982	NW_016535137.1	383635	387067	-	3251

novel.2985	NW_016535143.1	8346	14540	-	622
novel.2984	NW_016535141.1	4840	5141	-	302
novel.2987	NW_016535165.1	3690	5368	+	1640
novel.2986	NW_016535160.1	296600	299287	+	1984
novel.2989	NW_016535165.1	16658	32793	+	1344
novel.2988	NW_016535165.1	15238	15505	+	216
novel.5039	NW_016542722.1	181600	181943	+	344
novel.2189	NW_016532560.1	8143	9230	+	1088
novel.2188	NW_016532558.1	720	1000	-	242
novel.2181	NW_016532543.1	391813	392534	+	367
novel.2180	NW_016532542.1	276815	277131	+	291
novel.2183	NW_016532545.1	17635	19021	+	255
novel.2182	NW_016532543.1	623392	624237	+	605
novel.2185	NW_016532552.1	92874	93199	+	326
novel.2184	NW_016532549.1	2263	3230	+	811
novel.2187	NW_016532552.1	158745	174576	+	494
novel.2186	NW_016532552.1	147768	148029	+	218
novel.8767	NW_016606612.1	3248	5797	+	454
novel.2233	NW_016532697.1	192245	192563	+	267
novel.2232	NW_016532692.1	20800	21635	-	836
novel.2231	NW_016532692.1	19735	20383	+	244
novel.2230	NW_016532678.1	6440	7384	-	880
novel.2237	NW_016532707.1	29320	29717	+	341
novel.2236	NW_016532707.1	14119	15075	+	862
novel.2235	NW_016532702.1	10749	13475	+	399
novel.2234	NW_016532701.1	41062	88458	+	411
novel.2239	NW_016532708.1	8877	9961	+	1085
novel.5518	NW_016545517.1	14709	15713	-	963
novel.1528	NW_016530703.1	157271	157958	-	407
novel.1529	NW_016530703.1	158079	158912	-	657
novel.1526	NW_016530703.1	222835	223724	+	255
novel.1527	NW_016530703.1	95401	96283	-	723
novel.1524	NW_016530698.1	9979	27490	-	218
novel.1525	NW_016530702.1	311739	329766	-	421
novel.1522	NW_016530684.1	42469	44291	+	986
novel.1523	NW_016530684.1	1	2737	-	365
novel.1520	NW_016530683.1	81930	82297	-	324
novel.1521	NW_016530684.1	1	478	+	478
novel.5638	NW_016546240.1	606456	607087	+	204
novel.5639	NW_016546240.1	18163	108415	-	5271
novel.5636	NW_016546240.1	577958	578892	+	935
novel.5637	NW_016546240.1	596482	617918	+	2635
novel.5634	NW_016546240.1	552015	553227	+	1213
novel.5635	NW_016546240.1	558850	563097	+	2814

novel.5632	NW_016546240.1	24436	26282	+	1660
novel.5633	NW_016546240.1	460995	463111	+	609
novel.5630	NW_016546211.1	151594	152787	-	1138
novel.5631	NW_016546236.1	92996	93376	-	328
novel.7858	NW_016574325.1	716	2062	-	694
novel.7859	NW_016574333.1	3699	12119	+	704
novel.8764	NW_016606596.1	1	1452	+	1452
novel.7850	NW_016574136.1	909	1186	-	221
novel.7851	NW_016574146.1	57687	58071	+	385
novel.7852	NW_016574210.1	1	5025	+	1599
novel.7853	NW_016574210.1	1	1380	-	1255
novel.7854	NW_016574210.1	8398	10742	-	243
novel.7855	NW_016574229.1	63688	77942	+	855
novel.7856	NW_016574256.1	964	2628	-	1618
novel.7857	NW_016574325.1	1366	2121	+	722
novel.8763	NW_016606579.1	1	6299	-	594
novel.5180	NW_016543555.1	421537	423648	-	2112
novel.5181	NW_016543562.1	56339	56886	-	270
novel.5182	NW_016543580.1	18951	21287	-	2299
novel.5183	NW_016543583.1	64167	64609	+	244
novel.5184	NW_016543590.1	93055	97971	+	1932
novel.5185	NW_016543590.1	93068	94594	-	1527
novel.5186	NW_016543590.1	94909	101724	-	6280
novel.5187	NW_016543604.1	19367	20799	-	1433
novel.5188	NW_016543608.1	19355	31568	+	5647
novel.5189	NW_016543608.1	25468	31568	-	4187
novel.764	NW_016528977.1	75042	75337	-	247
novel.765	NW_016528977.1	155621	157261	-	1584
novel.766	NW_016528977.1	206845	226974	-	651
novel.767	NW_016528977.1	353010	355843	-	2521
novel.760	NW_016528967.1	7848	8489	+	585
novel.761	NW_016528967.1	7671	8142	-	418
novel.762	NW_016528969.1	195272	197332	+	300
novel.763	NW_016528969.1	236230	236571	+	285
novel.768	NW_016528978.1	364893	365180	+	230
novel.769	NW_016528996.1	226805	227710	-	906
novel.4589	NW_016540923.1	42924	57130	-	312
novel.4588	NW_016540923.1	90382	95029	+	244
novel.4587	NW_016540919.1	14938	16086	-	317
novel.4586	NW_016540917.1	5716	6545	+	671
novel.4585	NW_016540902.1	62488	70812	-	6767
novel.4584	NW_016540902.1	8868	33132	+	6959
novel.4583	NW_016540899.1	76774	79946	-	3139
novel.4582	NW_016540899.1	76753	79946	+	3042

novel.4581	NW_016540897.1	42604	45570	+	2754
novel.4580	NW_016540893.1	4407	22871	+	1309
novel.6729	NW_016554786.1	13975	15095	+	325
novel.6728	NW_016554786.1	8404	8775	+	325
novel.6721	NW_016554723.1	788	2213	+	1384
novel.6720	NW_016554722.1	34726	35278	-	415
novel.6723	NW_016554736.1	4578	5791	+	1185
novel.6722	NW_016554723.1	788	2677	-	1848
novel.6725	NW_016554776.1	14453	27238	+	341
novel.6724	NW_016554736.1	2722	3963	-	1217
novel.6727	NW_016554780.1	37032	38261	+	1230
novel.6726	NW_016554776.1	60125	85785	+	221
novel.3007	NW_016535220.1	138356	139102	+	232
novel.3006	NW_016535197.1	2	6040	-	210
novel.3005	NW_016535189.1	24621	25280	-	564
novel.3004	NW_016535189.1	31045	35477	+	829
novel.3003	NW_016535189.1	30099	30535	+	238
novel.3002	NW_016535189.1	25049	25794	+	555
novel.3001	NW_016535187.1	458460	459241	-	782
novel.3000	NW_016535187.1	457383	464456	+	761
novel.3009	NW_016535235.1	11818	12438	+	419
novel.3008	NW_016535232.1	175359	176413	-	684
novel.259	NW_016527844.1	29458	36336	+	347
novel.256	NW_016527838.1	127005	127275	-	223
novel.3915	NW_016538237.1	35533	37493	+	1961
novel.3914	NW_016538223.1	3689	7915	+	805
novel.3917	NW_016538245.1	4872	5545	+	674
novel.3916	NW_016538245.1	3977	4689	+	662
novel.3911	NW_016538211.1	413790	418465	-	232
novel.3910	NW_016538211.1	23181	24481	-	1301
novel.3913	NW_016538222.1	6371	10936	+	1859
novel.3912	NW_016538218.1	114071	136921	+	425
novel.3919	NW_016538248.1	8100	8389	+	235
novel.3918	NW_016538245.1	3627	4910	-	1119
novel.251	NW_016527824.1	259698	261951	+	2150
novel.2208	NW_016532622.1	142187	160289	+	617
novel.2754	NW_016534231.1	13728	14124	-	397
novel.2755	NW_016534234.1	2967	3345	+	323
novel.2756	NW_016534243.1	587113	601740	+	1960
novel.5529	NW_016545566.1	209246	211281	-	991
novel.2750	NW_016534220.1	13054	13770	+	667
novel.2751	NW_016534220.1	13148	13777	-	575
novel.2752	NW_016534220.1	14914	15123	-	210
novel.2753	NW_016534220.1	15347	15629	-	228

novel.2758	NW_016534261.1	173830	178788	+	1435
novel.2759	NW_016534274.1	16241	27549	+	1879
novel.2202	NW_016532583.1	101863	102563	+	340
novel.2203	NW_016532590.1	54806	56948	+	2143
novel.2200	NW_016532580.1	60141	62622	-	2425
novel.2538	NW_016533693.1	113960	114568	+	562
novel.2539	NW_016533693.1	116475	117078	+	551
novel.2201	NW_016532581.1	71965	81619	-	235
novel.2530	NW_016533666.1	14926	15159	-	234
novel.5526	NW_016545566.1	303756	304075	+	266
novel.2532	NW_016533684.1	70340	106277	+	221
novel.2533	NW_016533688.1	212367	213047	-	480
novel.2534	NW_016533691.1	11904	92099	+	1657
novel.2535	NW_016533692.1	94046	97105	+	2946
novel.2536	NW_016533692.1	98986	99263	+	221
novel.2537	NW_016533692.1	94052	98803	-	2850
novel.2204	NW_016532591.1	142302	143775	-	1328
novel.2205	NW_016532593.1	138092	139711	+	762
novel.2556	NW_016533723.1	154348	155294	-	947
novel.2554	NW_016533721.1	371261	385390	-	1346
novel.2555	NW_016533723.1	159364	160997	+	990
novel.5483	NW_016545368.1	59102	60198	+	1048
novel.8107	NW_016582586.1	89882	116610	-	1140
novel.5430	NW_016545039.1	1	1888	-	1888
novel.6534	NW_016553112.1	184802	185570	-	576
novel.6535	NW_016553116.1	1578	1985	+	408
novel.6536	NW_016553119.1	9542	10062	+	477
novel.6537	NW_016553119.1	8761	9551	-	595
novel.6530	NW_016553100.1	65604	72449	-	6811
novel.6531	NW_016553107.1	256	903	+	595
novel.6532	NW_016553107.1	724	1838	-	388
novel.6533	NW_016553112.1	199096	202649	+	455
novel.6538	NW_016553122.1	8304	11242	+	1424
novel.6539	NW_016553122.1	9389	11345	-	210
novel.7766	NW_016571716.1	11356	19944	+	313
novel.7767	NW_016571845.1	2884	5109	-	1184
novel.7764	NW_016571700.1	225	1219	+	469
novel.7765	NW_016571706.1	8668	10222	+	1508
novel.7762	NW_016571592.1	4315	37827	+	849
novel.7763	NW_016571671.1	1258	1578	-	321
novel.5431	NW_016545049.1	10656	13151	-	407
novel.7760	NW_016571560.1	94702	95828	-	394
novel.8012	NW_016579122.1	755	7302	+	6548
novel.8042	NW_016580044.1	652	6313	+	355

novel.8043	NW_016580107.1	4	2470	+	345
novel.8040	NW_016580040.1	11789	12199	+	388
novel.8041	NW_016580044.1	1	582	+	582
novel.7069	NW_016558502.1	3985	4876	+	232
novel.7068	NW_016558463.1	7237	7559	-	296
novel.8044	NW_016580107.1	331	3251	+	370
novel.8045	NW_016580107.1	4102	4533	-	409
novel.7065	NW_016558361.1	26446	27105	-	473
novel.7064	NW_016558353.1	383119	384372	-	915
novel.7067	NW_016558438.1	51202	51473	+	234
novel.8049	NW_016580477.1	1	492	-	492
novel.7061	NW_016558303.1	403189	404534	-	375
novel.7060	NW_016558300.1	1	1157	+	577
novel.7063	NW_016558353.1	377064	381257	-	2518
novel.7062	NW_016558353.1	383105	384372	+	929
novel.5379	NW_016544712.1	2069	2871	-	803
novel.5378	NW_016544701.1	51301	56768	-	3266
novel.5377	NW_016544701.1	18435	23135	-	625
novel.5376	NW_016544692.1	3791	4391	-	544
novel.5375	NW_016544692.1	1695	3628	-	1934
novel.5374	NW_016544692.1	1134	3148	+	338
novel.5373	NW_016544688.1	13611	15887	+	569
novel.5372	NW_016544685.1	51447	59116	+	251
novel.5371	NW_016544680.1	53707	54674	+	432
novel.5370	NW_016544680.1	13437	15119	+	1440
novel.3861	NW_016538049.1	131	672	+	542
novel.209	NW_016527690.1	13738	14876	-	1085
novel.208	NW_016527681.1	37032	37358	-	298
novel.207	NW_016527675.1	147941	148334	-	339
novel.206	NW_016527675.1	154633	156164	+	1532
novel.205	NW_016527675.1	148179	152645	+	4224
novel.204	NW_016527675.1	53713	66092	+	3546
novel.203	NW_016527674.1	487116	487632	-	493
novel.202	NW_016527674.1	350774	353566	+	1700
novel.201	NW_016527671.1	29264	30689	-	1010
novel.200	NW_016527669.1	864250	865165	+	339
novel.7281	NW_016562083.1	2355	2888	-	484
novel.8595	NW_016602473.1	9	544	-	401
novel.8596	NW_016602473.1	1849	2350	-	222
novel.8597	NW_016602505.1	1	2361	-	1686
novel.7285	NW_016562183.1	10215	12682	-	1054
novel.8591	NW_016602371.1	1119	2222	-	1019
novel.8592	NW_016602388.1	852	1505	+	558
novel.8593	NW_016602390.1	163	1824	-	252

novel.7289	NW_016562232.1	3466	3900	-	346
novel.7288	NW_016562219.1	1800	4307	-	2508
novel.8598	NW_016602569.1	20	918	-	529
novel.8599	NW_016602604.1	603	1526	+	303
novel.8268	NW_016587563.1	1	637	+	580
novel.8269	NW_016587615.1	485	1044	+	512
novel.936	NW_016529408.1	117356	117962	+	607
novel.3867	NW_016538055.1	3163	7712	-	1191
novel.3866	NW_016538055.1	1553	1983	-	342
novel.8635	NW_016603604.1	78	1429	-	570
novel.8634	NW_016603561.1	1	1007	+	1007
novel.932	NW_016529400.1	1143257	1143890	-	259
novel.8637	NW_016603733.1	1	2235	-	1625
novel.4398	NW_016540195.1	57467	70409	+	2026
novel.4399	NW_016540195.1	56700	57647	-	948
novel.4392	NW_016540186.1	9093	9377	-	253
novel.4393	NW_016540186.1	10104	10440	-	337
novel.4390	NW_016540174.1	384579	389860	-	468
novel.4391	NW_016540181.1	258991	270565	+	1564
novel.4396	NW_016540192.1	29127	32249	+	2345
novel.4397	NW_016540192.1	4989	6935	-	305
novel.4394	NW_016540186.1	10650	14978	-	818
novel.4395	NW_016540191.1	14058	14987	-	475
novel.8633	NW_016603542.1	1	2787	+	478
novel.8632	NW_016603503.1	1	2420	-	492
novel.3612	NW_016537221.1	77364	79361	-	1998
novel.3613	NW_016537221.1	87493	97133	-	1479
novel.3610	NW_016537221.1	8398	22508	+	848
novel.3611	NW_016537221.1	7599	8213	-	370
novel.6888	NW_016556555.1	5064	6144	-	817
novel.6889	NW_016556582.1	7955	21516	+	2969
novel.3614	NW_016537240.1	1	1169	-	1128
novel.3615	NW_016537251.1	131835	133473	+	482
novel.6884	NW_016556518.1	1603	2153	-	504
novel.6885	NW_016556545.1	26089	26419	+	274
novel.3618	NW_016537251.1	85188	86220	-	1033
novel.3619	NW_016537256.1	109523	110520	+	581
novel.6880	NW_016556499.1	10823	12608	+	610
novel.6881	NW_016556499.1	4887	5231	-	246
novel.6882	NW_016556510.1	38	4258	-	274
novel.6883	NW_016556512.1	9114	10307	-	1194
novel.8161	NW_016584385.1	14	682	+	387
novel.4028	NW_016538580.1	10158	14150	+	307
novel.4029	NW_016538580.1	23600	24813	-	1161

novel.4024	NW_016538573.1	356	986	-	245
novel.4025	NW_016538574.1	78174	83859	+	5535
novel.4026	NW_016538574.1	93768	94248	+	457
novel.4027	NW_016538574.1	78728	83859	-	5078
novel.4020	NW_016538545.1	2174	14229	-	675
novel.4021	NW_016538557.1	3704	7108	+	903
novel.4022	NW_016538557.1	16519	17082	+	508
novel.4023	NW_016538557.1	41907	44949	-	645
novel.8160	NW_016584368.1	412	1133	+	668
novel.6084	NW_016549431.1	1729	2250	-	494
novel.6085	NW_016549435.1	58837	76014	+	1558
novel.6086	NW_016549452.1	3067	4059	+	937
novel.6087	NW_016549457.1	49935	51086	-	337
novel.6080	NW_016549422.1	17442	17809	-	340
novel.6081	NW_016549428.1	157865	165299	+	1204
novel.6082	NW_016549428.1	305789	307697	-	1909
novel.6083	NW_016549428.1	318949	320107	-	1159
novel.6088	NW_016549459.1	78698	79617	+	721
novel.6089	NW_016549459.1	37421	39059	-	704
novel.5433	NW_016545053.1	3369	55665	+	292
novel.2321	NW_016533039.1	403597	403968	+	342
novel.849	NW_016529226.1	43286	43908	-	273
novel.848	NW_016529223.1	388750	390008	+	224
novel.5577	NW_016545890.1	32458	32695	-	200
novel.5576	NW_016545881.1	23569	23959	+	335
novel.2251	NW_016532769.1	31642	32128	-	321
novel.2250	NW_016532767.1	698584	711250	-	802
novel.2253	NW_016532782.1	24619	47488	-	1021
novel.2252	NW_016532771.1	16	3056	+	3041
novel.841	NW_016529207.1	36443	37473	-	332
novel.840	NW_016529198.1	10858	11338	-	437
novel.843	NW_016529213.1	14439	20624	-	605
novel.842	NW_016529207.1	56055	61835	-	515
novel.845	NW_016529217.1	16238	19170	+	446
novel.844	NW_016529216.1	83508	85738	+	1299
novel.847	NW_016529220.1	12515	12881	-	319
novel.846	NW_016529219.1	103872	105256	-	254
novel.8029	NW_016579875.1	12593	13395	-	746
novel.1052	NW_016529639.1	79503	84391	-	4889
novel.1053	NW_016529640.1	161209	162200	+	308
novel.1502	NW_016530649.1	203746	218094	-	1811
novel.1051	NW_016529638.1	78389	78769	+	381
novel.1056	NW_016529644.1	24209	24531	-	323
novel.1057	NW_016529647.1	41567	91110	-	460

novel.1054	NW_016529640.1	244171	244912	+	669
novel.1507	NW_016530659.1	19147	19700	-	554
novel.1508	NW_016530659.1	21357	22036	-	680
novel.1509	NW_016530662.1	7784	20059	-	1050
novel.1058	NW_016529657.1	530371	533647	-	1198
novel.1059	NW_016529663.1	228709	228961	-	221
novel.8000	NW_016578669.1	4196	5132	+	484
novel.5610	NW_016546092.1	2939	3421	-	433
novel.5611	NW_016546099.1	69631	71434	+	1804
novel.5612	NW_016546103.1	301765	317529	+	549
novel.5613	NW_016546105.1	29314	31843	+	450
novel.5614	NW_016546111.1	61922	96005	+	923
novel.5615	NW_016546146.1	486710	487112	-	215
novel.5616	NW_016546151.1	570253	570701	+	233
novel.5617	NW_016546152.1	1	1085	+	459
novel.5618	NW_016546152.1	422	685	-	210
novel.5619	NW_016546159.1	14078	18026	-	344
novel.4628	NW_016541041.1	19199	21616	-	2266
novel.4629	NW_016541043.1	47507	49821	-	473
novel.4622	NW_016541014.1	138	1054	+	629
novel.4623	NW_016541018.1	46625	64352	-	505
novel.4620	NW_016541008.1	28115	32288	-	380
novel.4621	NW_016541008.1	73214	95837	-	527
novel.4626	NW_016541039.1	16436	16953	+	518
novel.4627	NW_016541040.1	13636	50511	+	412
novel.4624	NW_016541019.1	9626	20176	-	267
novel.4625	NW_016541032.1	18529	19586	-	699
novel.8544	NW_016600345.1	1	1005	-	392
novel.4561	NW_016540770.1	18788	19116	+	295
novel.4560	NW_016540765.1	35916	37047	-	1042
novel.4563	NW_016540773.1	178625	180301	-	1170
novel.4562	NW_016540770.1	19748	20081	-	286
novel.4565	NW_016540777.1	32340	33169	-	647
novel.4564	NW_016540777.1	32552	33137	+	455
novel.4567	NW_016540796.1	70948	71325	-	378
novel.4566	NW_016540787.1	36691	37059	+	248
novel.4569	NW_016540833.1	7262	8590	+	1303
novel.4568	NW_016540809.1	10361	10773	-	413
novel.6703	NW_016554652.1	124547	136695	-	2299
novel.6702	NW_016554652.1	313929	314275	+	290
novel.6701	NW_016554652.1	293730	315027	+	1235
novel.6700	NW_016554642.1	269515	270525	-	1011
novel.6707	NW_016554667.1	20622	21078	+	248
novel.6706	NW_016554663.1	8398	57666	+	955

novel.6705	NW_016554662.1	971	1253	-	283
novel.6704	NW_016554652.1	273217	273478	-	219
novel.6709	NW_016554672.1	15661	20224	+	419
novel.6708	NW_016554671.1	38319	39885	+	311
novel.8748	NW_016606133.1	1	712	+	712
novel.8541	NW_016600248.1	855	1796	-	607
novel.8540	NW_016600195.1	1	1786	-	1786
novel.7181	NW_016559917.1	1	1167	+	1143
novel.2888	NW_016534753.1	87126	87409	-	233
novel.2889	NW_016534754.1	5	20999	+	749
novel.2738	NW_016534206.1	19968	20289	+	322
novel.2739	NW_016534207.1	70057	72141	+	2060
novel.2736	NW_016534205.1	234882	235692	+	400
novel.2737	NW_016534205.1	43033	45118	-	2086
novel.2734	NW_016534197.1	141607	142016	-	410
novel.2735	NW_016534204.1	189535	189845	+	259
novel.2732	NW_016534189.1	141161	146603	-	856
novel.2733	NW_016534190.1	34328	35453	+	216
novel.2730	NW_016534188.1	23589	24130	-	520
novel.2731	NW_016534189.1	140304	147331	+	283
novel.8679	NW_016604870.1	1	1513	-	630
novel.8678	NW_016604845.1	503	927	-	425
novel.7548	NW_016567037.1	206	1322	-	1060
novel.7549	NW_016567037.1	4001	5206	-	459
novel.7542	NW_016566978.1	1	3040	-	704
novel.7543	NW_016566978.1	4149	4594	-	446
novel.7540	NW_016566838.1	1145	1346	+	202
novel.7541	NW_016566960.1	49400	50490	-	1021
novel.7546	NW_016567037.1	2856	3758	+	847
novel.7547	NW_016567037.1	4001	6339	+	1592
novel.7544	NW_016566978.1	4704	5021	-	295
novel.7545	NW_016567017.1	45297	54775	+	1857
novel.3289	NW_016536080.1	5949	77446	-	249
novel.3288	NW_016536076.1	41502	56231	-	2800
novel.3939	NW_016538328.1	407002	407875	-	277
novel.3938	NW_016538328.1	296682	297035	-	261
novel.3937	NW_016538328.1	204654	205035	+	330
novel.3284	NW_016536065.1	496991	497857	-	514
novel.3287	NW_016536075.1	22147	22530	-	329
novel.3286	NW_016536075.1	114	3105	-	401
novel.3933	NW_016538308.1	20549	20866	-	265
novel.3932	NW_016538303.1	586	1511	+	810
novel.3283	NW_016536058.1	86236	100107	-	1245
novel.3282	NW_016536055.1	54897	55782	-	563

novel.2080	NW_016532217.1	387820	388290	+	286
novel.2081	NW_016532217.1	67938	68717	-	780
novel.2082	NW_016532220.1	126213	128172	+	269
novel.2083	NW_016532224.1	35326	35705	+	380
novel.2084	NW_016532224.1	1778	35726	-	353
novel.2085	NW_016532227.1	59379	59878	+	324
novel.2086	NW_016532227.1	247913	254652	+	662
novel.2087	NW_016532227.1	426853	428727	+	1849
novel.2088	NW_016532227.1	60154	61109	-	759
novel.2089	NW_016532227.1	395680	433976	-	615
novel.3429	NW_016536614.1	221673	222446	+	774
novel.3428	NW_016536607.1	5193	5784	-	592
novel.6141	NW_016549770.1	48069	49329	+	1208
novel.6140	NW_016549765.1	73022	74743	-	560
novel.3423	NW_016536565.1	11443	12651	-	851
novel.3422	NW_016536557.1	12793	13098	-	263
novel.3425	NW_016536594.1	7300	10595	+	472
novel.3424	NW_016536580.1	44083	44871	+	732
novel.3427	NW_016536597.1	1101	2337	+	1237
novel.3426	NW_016536594.1	22083	22921	+	789
novel.2116	NW_016532274.1	132718	133321	-	352
novel.4811	NW_016541810.1	17057	17616	+	560
novel.4810	NW_016541805.1	368434	375620	-	204
novel.4813	NW_016541811.1	155629	157421	+	1066
novel.4812	NW_016541810.1	12089	17616	-	597
novel.4815	NW_016541827.1	19166	19472	+	284
novel.4814	NW_016541813.1	18414	20738	+	2230
novel.4817	NW_016541827.1	26260	27790	-	1531
novel.4816	NW_016541827.1	24757	25334	-	542
novel.4819	NW_016541840.1	47873	49081	+	726
novel.4818	NW_016541830.1	177233	178707	+	1475
novel.3447	NW_016536661.1	8579	9946	+	1277
novel.6105	NW_016549575.1	20127	22049	+	244
novel.6104	NW_016549574.1	353	1319	+	913
novel.6107	NW_016549578.1	22539	23432	-	894
novel.6106	NW_016549578.1	21881	23432	+	1497
novel.6558	NW_016553283.1	40114	40584	-	426
novel.6559	NW_016553284.1	1195	2093	+	469
novel.6556	NW_016553251.1	1	514	+	514
novel.6101	NW_016549552.1	5287	6131	-	804
novel.6554	NW_016553241.1	8167	9664	+	1467
novel.6555	NW_016553241.1	8167	10602	-	1375
novel.6552	NW_016553225.1	4941	17940	-	473
novel.6553	NW_016553238.1	9379	9742	-	364

novel.6550	NW_016553218.1	488	1668	+	1181
novel.6100	NW_016549532.1	607922	608786	+	239
novel.7793	NW_016572504.1	5100	5476	-	326
novel.6103	NW_016549573.1	130516	131815	-	342
novel.7792	NW_016572399.1	8904	11350	+	215
novel.6102	NW_016549567.1	4184	5528	+	266
novel.7791	NW_016572395.1	5213	5498	+	237
novel.3381	NW_016536405.1	58958	59975	+	1018
novel.7022	NW_016557922.1	17956	20211	-	2152
novel.3445	NW_016536657.1	4415	4658	-	244
novel.7797	NW_016572662.1	228123	228898	+	720
novel.7796	NW_016572662.1	103407	107141	+	2232
novel.7795	NW_016572607.1	14571	21050	-	502
novel.6109	NW_016549605.1	998	1292	+	245
novel.7026	NW_016557962.1	23825	25274	+	229
novel.6108	NW_016549603.1	5425	5800	+	343
novel.1269	NW_016530093.1	34262	34521	-	223
novel.1268	NW_016530092.1	118110	119170	-	774
novel.1261	NW_016530086.1	3837	10489	+	1035
novel.1260	NW_016530082.1	198724	205088	-	337
novel.1263	NW_016530087.1	49671	52240	+	342
novel.1262	NW_016530086.1	14148	14696	+	549
novel.1265	NW_016530092.1	91984	92736	+	471
novel.1264	NW_016530087.1	79441	79959	-	482
novel.1267	NW_016530092.1	92384	92736	-	304
novel.1266	NW_016530092.1	118114	118453	+	292
novel.3441	NW_016536640.1	9599	33224	+	595
novel.8064	NW_016581275.1	4350	5768	+	265
novel.8065	NW_016581355.1	447	961	-	322
novel.7045	NW_016558140.1	4614	5826	+	567
novel.8067	NW_016581395.1	1	1047	-	1000
novel.7043	NW_016558126.1	9273	9739	-	416
novel.7042	NW_016558122.1	73534	86631	+	665
novel.7041	NW_016558091.1	7652	8018	+	367
novel.7040	NW_016558084.1	13628	14569	-	354
novel.7739	NW_016570606.1	56992	59674	-	2081
novel.7738	NW_016570606.1	119014	127618	+	8591
novel.3440	NW_016536638.1	326123	327067	-	337
novel.8068	NW_016581407.1	1222	3623	+	484
novel.8069	NW_016581471.1	731	1998	-	1239
novel.7049	NW_016558167.1	7786	8425	+	640
novel.7048	NW_016558167.1	6413	7014	+	545
novel.5391	NW_016544765.1	24726	31859	+	292
novel.5390	NW_016544759.1	218	688	+	415

novel.5393	NW_016544765.1	71006	71477	+	419
novel.5392	NW_016544765.1	55991	56428	+	385
novel.5395	NW_016544791.1	91302	91614	-	313
novel.5394	NW_016544766.1	88625	88989	+	365
novel.5397	NW_016544816.1	5081	5682	+	523
novel.5396	NW_016544795.1	58754	58993	+	240
novel.5399	NW_016544828.1	143788	151565	+	2162
novel.5398	NW_016544816.1	5081	5635	-	401
novel.597	NW_016528567.1	81498	90544	+	411
novel.596	NW_016528564.1	20234	21370	+	570
novel.595	NW_016528560.1	101086	112958	-	8165
novel.594	NW_016528559.1	287612	289374	-	528
novel.593	NW_016528555.1	105902	145070	+	1401
novel.592	NW_016528552.1	49567	49845	-	279
novel.591	NW_016528550.1	89243	111996	+	708
novel.590	NW_016528550.1	35409	41211	+	1312
novel.269	NW_016527890.1	19329	22290	+	2859
novel.268	NW_016527887.1	39896	40545	-	622
novel.599	NW_016528572.1	5107	5745	+	581
novel.598	NW_016528569.1	45132	45697	-	566
novel.5764	NW_016547064.1	15672	18903	+	3232
novel.5765	NW_016547064.1	60072	60534	+	311
novel.5584	NW_016545910.1	204348	204765	-	278
novel.5585	NW_016545922.1	236840	237138	+	248
novel.5586	NW_016545926.1	42848	46449	-	3457
novel.5587	NW_016545967.1	5117	39987	-	483
novel.6416	NW_016551858.1	40622	43242	-	564
novel.5580	NW_016545902.1	1526	27984	+	274
novel.5853	NW_016547698.1	10237	11133	-	241
novel.5582	NW_016545906.1	1	1370	+	1313
novel.7731	NW_016570513.1	8394	8714	-	266
novel.144	NW_016527558.1	467	1066	-	544
novel.5583	NW_016545910.1	1245	38732	-	518
novel.5769	NW_016547085.1	112685	116923	-	524
novel.3634	NW_016537299.1	26814	27663	+	797
novel.3635	NW_016537300.1	202675	207581	-	4853
novel.3636	NW_016537307.1	65061	65710	-	594
novel.3637	NW_016537307.1	72691	89891	-	695
novel.3630	NW_016537285.1	452164	462162	+	548
novel.3631	NW_016537288.1	650	1742	+	787
novel.3632	NW_016537296.1	23	985	-	565
novel.3633	NW_016537298.1	25425	25718	+	238
novel.3784	NW_016537773.1	133486	134593	+	760
novel.6410	NW_016551788.1	11961	12902	-	942

novel.3638	NW_016537309.1	63156	73540	+	808
novel.3639	NW_016537317.1	128296	128663	+	316
novel.3785	NW_016537775.1	39243	40162	-	865
novel.7730	NW_016570513.1	1338	9104	-	439
novel.4046	NW_016538657.1	25138	27135	+	1591
novel.3788	NW_016537779.1	509898	510791	+	206
novel.4044	NW_016538655.1	16966	17308	+	306
novel.4045	NW_016538655.1	5322	10653	-	1871
novel.4042	NW_016538655.1	5322	5660	+	292
novel.4043	NW_016538655.1	8933	10653	+	1671
novel.4040	NW_016538650.1	88155	89099	+	945
novel.8551	NW_016600524.1	26	1708	-	1502
novel.8240	NW_016587020.1	205	1368	+	328
novel.8241	NW_016587038.1	2049	2751	-	219
novel.8242	NW_016587039.1	1	1059	-	214
novel.8243	NW_016587082.1	1055	1529	-	310
novel.8244	NW_016587088.1	1080	1589	+	510
novel.8245	NW_016587119.1	6707	17968	-	377
novel.4048	NW_016538657.1	25138	25701	-	431
novel.4049	NW_016538657.1	78320	79118	-	755
novel.7733	NW_016570526.1	4982	6633	-	1652
novel.8221	NW_016586511.1	620	893	+	217
novel.8226	NW_016586750.1	1	1022	+	965
novel.8227	NW_016586750.1	1	1022	-	967
novel.8556	NW_016600916.1	1	1929	-	544
novel.8225	NW_016586736.1	35	508	+	228
novel.7732	NW_016570526.1	4982	5988	+	1007
novel.5319	NW_016544320.1	1	377	+	330
novel.7735	NW_016570539.1	40861	60168	-	414
novel.8120	NW_016583146.1	1837	2125	-	268
novel.7734	NW_016570539.1	5109	7828	-	216
novel.7980	NW_016578321.1	436	794	-	359
novel.7737	NW_016570552.1	6439	9541	+	506
novel.5542	NW_016545602.1	1	876	-	828
novel.5809	NW_016547415.1	5444	5710	+	267
novel.5808	NW_016547412.1	69844	77102	-	6988
novel.2279	NW_016532879.1	97970	98866	+	851
novel.2278	NW_016532868.1	209158	209751	+	399
novel.2277	NW_016532866.1	106516	107846	-	1331
novel.2276	NW_016532863.1	78374	103484	+	610
novel.5803	NW_016547376.1	1	1078	-	1057
novel.2274	NW_016532859.1	138538	142694	-	4101
novel.2273	NW_016532853.1	12292	15221	-	255
novel.2272	NW_016532853.1	9259	16566	+	250

novel.2271	NW_016532849.1	677980	678282	-	303
novel.2270	NW_016532849.1	607557	608353	-	562
novel.869	NW_016529255.1	77087	77894	+	654
novel.868	NW_016529252.1	243313	244278	-	262
novel.867	NW_016529252.1	307679	309394	+	1068
novel.866	NW_016529250.1	145028	145425	-	309
novel.865	NW_016529250.1	144188	144850	-	663
novel.864	NW_016529250.1	175773	176268	+	439
novel.863	NW_016529250.1	145659	146195	+	482
novel.862	NW_016529246.1	2696	3425	-	730
novel.861	NW_016529242.1	53367	55380	-	1957
novel.860	NW_016529242.1	15716	16248	-	533
novel.7986	NW_016578484.1	7797	8069	-	217
novel.7522	NW_016566506.1	145495	166272	+	3135
novel.8394	NW_016591899.1	3578	6225	+	405
novel.1074	NW_016529692.1	6933	17827	-	1104
novel.1075	NW_016529695.1	6601	22780	-	631
novel.1076	NW_016529696.1	137287	315244	+	519
novel.1077	NW_016529696.1	150758	151089	+	276
novel.1070	NW_016529668.1	408304	408993	+	444
novel.1071	NW_016529668.1	370675	377457	-	560
novel.1072	NW_016529678.1	215331	238090	+	473
novel.1073	NW_016529685.1	74701	75140	-	440
novel.5318	NW_016544319.1	1	518	+	518
novel.1078	NW_016529696.1	95213	95924	-	582
novel.1079	NW_016529698.1	98064	101151	+	225
novel.7987	NW_016578487.1	1881	2213	+	282
novel.7894	NW_016575413.1	1	975	-	637
novel.7895	NW_016575487.1	1	10248	-	320
novel.7896	NW_016575497.1	25625	27987	+	2158
novel.7897	NW_016575523.1	845	1375	+	484
novel.7890	NW_016575371.1	855	1113	+	219
novel.7891	NW_016575378.1	18751	20423	+	1161
novel.7892	NW_016575378.1	18751	19604	-	854
novel.7893	NW_016575378.1	19970	20423	-	284
novel.7898	NW_016575554.1	8317	8946	+	630
novel.7899	NW_016575554.1	10282	10596	-	266
novel.8007	NW_016578920.1	89958	124303	+	908
novel.4608	NW_016540978.1	536759	537962	+	439
novel.4609	NW_016540978.1	704247	714774	+	834
novel.4604	NW_016540958.1	20812	22712	-	324
novel.4605	NW_016540970.1	111527	111852	-	294
novel.4606	NW_016540978.1	69014	69359	+	221
novel.4607	NW_016540978.1	69242	69646	+	255

novel.4600	NW_016540952.1	5505	7734	+	2230
novel.4601	NW_016540952.1	103378	106314	+	2937
novel.4602	NW_016540952.1	5505	7734	-	2230
novel.4603	NW_016540958.1	48918	49945	+	863
novel.2423	NW_016533334.1	49219	62600	+	1288
novel.7599	NW_016568031.1	97044	107516	+	792
novel.1212	NW_016529952.1	93752	94108	-	309
novel.4549	NW_016540738.1	1706	2482	-	723
novel.4548	NW_016540735.1	114753	116033	+	463
novel.4543	NW_016540716.1	344988	347527	+	494
novel.4542	NW_016540708.1	1423	1819	-	341
novel.4541	NW_016540703.1	6779	14251	-	682
novel.4540	NW_016540703.1	7787	8974	+	1144
novel.4547	NW_016540735.1	92855	93295	+	394
novel.4546	NW_016540732.1	405454	406234	-	582
novel.4545	NW_016540726.1	290801	293515	+	354
novel.4544	NW_016540720.1	39398	39837	+	387
novel.6765	NW_016555230.1	9100	9764	-	261
novel.6764	NW_016555201.1	9245	9725	-	424
novel.6767	NW_016555265.1	92888	96871	-	2749
novel.6766	NW_016555265.1	92888	94944	+	2057
novel.6761	NW_016555198.1	10874	11253	+	355
novel.6760	NW_016555179.1	9687	10503	+	817
novel.1388	NW_016530347.1	2992	3468	-	477
novel.1389	NW_016530348.1	87808	88632	-	769
novel.1386	NW_016530347.1	4113	7011	+	1879
novel.1387	NW_016530347.1	7126	9612	+	2487
novel.1384	NW_016530345.1	226746	253585	-	2985
novel.1385	NW_016530347.1	2415	3482	+	965
novel.1382	NW_016530344.1	1070	3852	-	1202
novel.1383	NW_016530345.1	251127	256019	+	2273
novel.1380	NW_016530343.1	365811	367382	-	323
novel.1381	NW_016530344.1	1070	2995	+	270
novel.678	NW_016528768.1	59537	77274	+	2879
novel.679	NW_016528776.1	3646	10394	+	2451
novel.672	NW_016528748.1	181015	181543	+	283
novel.673	NW_016528755.1	384225	390692	-	410
novel.670	NW_016528742.1	107275	109684	-	510
novel.671	NW_016528748.1	108177	110968	+	2576
novel.676	NW_016528762.1	237403	237761	-	228
novel.677	NW_016528765.1	1759	2461	+	330
novel.674	NW_016528756.1	5007	5381	-	250
novel.675	NW_016528762.1	50690	101555	-	527
novel.2339	NW_016533092.1	225415	225674	+	260

novel.2332	NW_016533069.1	8338	9585	-	741
novel.7595	NW_016567874.1	10189	10991	+	747
novel.5453	NW_016545158.1	5313	6238	-	879
novel.2330	NW_016533069.1	2892	3913	+	998
novel.2331	NW_016533069.1	8338	9435	+	712
novel.2336	NW_016533086.1	386	853	-	429
novel.5457	NW_016545210.1	5852	7979	-	321
novel.7388	NW_016563869.1	1	699	+	673
novel.7389	NW_016563869.1	959	1586	-	522
novel.2334	NW_016533075.1	103962	113332	+	499
novel.7380	NW_016563638.1	20356	22359	+	1238
novel.7381	NW_016563669.1	2011	3593	-	312
novel.7382	NW_016563724.1	1	660	-	632
novel.2335	NW_016533082.1	45056	45440	-	255
novel.7384	NW_016563800.1	9592	10440	+	792
novel.7385	NW_016563811.1	44	442	-	352
novel.7386	NW_016563855.1	29695	30039	+	345
novel.7387	NW_016563863.1	5705	6022	+	294
novel.1928	NW_016531792.1	9176	10204	+	311
novel.1929	NW_016531795.1	966	1399	-	410
novel.1922	NW_016531775.1	36327	36685	-	314
novel.1923	NW_016531777.1	44796	45000	+	205
novel.1920	NW_016531775.1	27513	30721	+	3153
novel.1921	NW_016531775.1	30654	36293	-	2984
novel.1926	NW_016531779.1	62056	63754	-	236
novel.1927	NW_016531782.1	123723	124379	-	254
novel.1924	NW_016531777.1	102972	103966	-	612
novel.1925	NW_016531779.1	163336	163867	+	292
novel.5238	NW_016543814.1	16448	21834	+	5387
novel.5239	NW_016543814.1	32966	33562	-	552
novel.5232	NW_016543805.1	46868	50793	+	3473
novel.5233	NW_016543805.1	53348	54535	+	1166
novel.5230	NW_016543798.1	3864	10254	-	586
novel.5231	NW_016543798.1	16875	19256	-	399
novel.5236	NW_016543805.1	5594	13708	-	1774
novel.5237	NW_016543805.1	119208	121524	-	2078
novel.5234	NW_016543805.1	69284	74804	+	697
novel.5235	NW_016543805.1	118978	121090	+	2113
novel.2710	NW_016534147.1	13522	13807	+	229
novel.2863	NW_016534656.1	16667	16958	-	292
novel.2860	NW_016534648.1	21911	62659	+	431
novel.2861	NW_016534648.1	37697	38880	+	600
novel.2866	NW_016534660.1	216938	217720	+	783
novel.2867	NW_016534668.1	127659	132646	+	355

novel.2864	NW_016534656.1	25718	29063	-	1353
novel.2865	NW_016534656.1	138351	139727	-	1322
novel.2718	NW_016534159.1	18407	18723	-	265
novel.2719	NW_016534159.1	36388	36849	-	229
novel.2868	NW_016534670.1	8643	26938	-	298
novel.2869	NW_016534670.1	135223	135678	-	402
novel.8653	NW_016604243.1	2159	2529	+	250
novel.8652	NW_016604210.1	1558	3162	+	1605
novel.8651	NW_016604178.1	2114	2986	+	276
novel.8650	NW_016604086.1	64	1826	-	689
novel.7568	NW_016567405.1	105326	109102	-	1304
novel.7569	NW_016567453.1	2601	35373	+	642
novel.8655	NW_016604355.1	548	3265	+	2718
novel.8654	NW_016604323.1	1	1159	-	1049
novel.7564	NW_016567382.1	887	1499	+	561
novel.7565	NW_016567396.1	18074	19168	-	494
novel.7566	NW_016567401.1	8037	27017	+	401
novel.7567	NW_016567405.1	105326	106538	+	849
novel.7560	NW_016567291.1	8373	10185	+	1813
novel.7561	NW_016567291.1	7575	8007	-	283
novel.7562	NW_016567318.1	1	299	+	261
novel.7563	NW_016567351.1	1	516	-	464
novel.2340	NW_016533092.1	225415	225685	-	271
novel.3701	NW_016537534.1	38497	51585	-	1716
novel.5545	NW_016545607.1	19533	22872	-	2065
novel.3403	NW_016536481.1	106753	113511	+	529
novel.3402	NW_016536475.1	107120	108807	+	248
novel.3401	NW_016536473.1	32898	33478	-	529
novel.3400	NW_016536466.1	98245	99227	-	207
novel.3407	NW_016536494.1	213411	214428	-	236
novel.3406	NW_016536494.1	212325	213204	-	880
novel.3405	NW_016536490.1	257060	257383	+	289
novel.3404	NW_016536490.1	38130	43037	+	275
novel.3409	NW_016536509.1	361016	362711	+	276
novel.3408	NW_016536509.1	48292	58305	+	946
novel.4839	NW_016541879.1	13904	16139	+	203
novel.4838	NW_016541877.1	17515	18854	+	856
novel.4833	NW_016541850.1	37269	37476	+	208
novel.4832	NW_016541849.1	340852	349849	-	1274
novel.4831	NW_016541849.1	281772	282823	-	1007
novel.4830	NW_016541849.1	306463	307004	+	262
novel.4837	NW_016541876.1	8971	44606	-	1294
novel.4836	NW_016541861.1	69771	70264	-	447
novel.4835	NW_016541853.1	467579	488845	-	1472

novel.4834	NW_016541853.1	238584	239727	-	906
novel.614	NW_016528606.1	260670	261269	+	257
novel.2649	NW_016533982.1	120987	121234	+	222
novel.7638	NW_016568861.1	1847	2657	+	754
novel.3707	NW_016537535.1	187795	188787	-	993
novel.615	NW_016528617.1	27317	31333	-	3862
novel.4491	NW_016540573.1	978399	978681	-	283
novel.4490	NW_016540573.1	723975	724508	-	234
novel.4493	NW_016540573.1	986241	987362	-	1122
novel.4492	NW_016540573.1	983045	989736	-	677
novel.6570	NW_016553425.1	6238	6560	+	268
novel.6571	NW_016553434.1	842	1203	-	312
novel.6572	NW_016553436.1	81917	89205	+	1184
novel.6573	NW_016553450.1	4585	4887	-	272
novel.6574	NW_016553453.1	10778	11478	+	701
novel.6575	NW_016553467.1	47130	47937	+	622
novel.6576	NW_016553467.1	46453	47423	-	922
novel.6577	NW_016553467.1	47584	47935	-	296
novel.6578	NW_016553468.1	4492	6462	-	1971
novel.6579	NW_016553470.1	42319	44243	+	1870
novel.7614	NW_016568406.1	40703	41571	-	257
novel.6048	NW_016549226.1	21494	24028	-	750
novel.7615	NW_016568466.1	5048	5414	-	296
novel.6049	NW_016549261.1	16114	16644	-	327
novel.8121	NW_016583219.1	10	1318	+	215
novel.7613	NW_016568354.1	3815	4600	+	733
novel.7610	NW_016568251.1	708	13447	-	1494
novel.7611	NW_016568307.1	502	6782	+	226
novel.6040	NW_016549125.1	12538	13221	-	458
novel.6041	NW_016549128.1	15878	17695	+	1818
novel.6042	NW_016549128.1	3638	7179	-	411
novel.3563	NW_016537129.1	23852	25313	-	270
novel.3564	NW_016537129.1	24100	24392	-	293
novel.7278	NW_016562059.1	118487	119487	+	502
novel.6045	NW_016549213.1	12600	13052	+	413
novel.5547	NW_016545607.1	25898	26131	-	234
novel.6046	NW_016549217.1	1742	23035	-	668
novel.6047	NW_016549226.1	3273	4949	-	1677
novel.1243	NW_016530046.1	150115	165120	-	1217
novel.1242	NW_016530046.1	9257	9725	-	469
novel.1241	NW_016530046.1	79909	85583	+	1085
novel.1240	NW_016530039.1	403806	407216	-	3411
novel.1247	NW_016530059.1	95687	96061	+	322
novel.1246	NW_016530057.1	147661	150174	+	949

novel.1245	NW_016530055.1	119066	122327	-	458
novel.1244	NW_016530047.1	64926	66328	-	815
novel.1719	NW_016531279.1	469202	476156	-	485
novel.1718	NW_016531279.1	82176	102314	+	598
novel.1249	NW_016530065.1	10868	12152	-	346
novel.1248	NW_016530063.1	13131	13510	-	380
novel.5001	NW_016542575.1	314270	356325	-	534
novel.5000	NW_016542575.1	361368	365100	+	594
novel.5003	NW_016542581.1	3522	3818	-	297
novel.5002	NW_016542581.1	24923	26080	+	592
novel.5005	NW_016542584.1	6823	7438	+	532
novel.5004	NW_016542581.1	18421	20431	-	537
novel.5007	NW_016542589.1	122415	123590	+	1176
novel.5006	NW_016542589.1	19306	28136	+	1875
novel.5009	NW_016542603.1	8252	9053	+	779
novel.5008	NW_016542589.1	58141	69120	-	5956
novel.7126	NW_016559303.1	29397	29657	+	209
novel.7127	NW_016559317.1	628	1006	+	335
novel.7120	NW_016559227.1	3139	31261	-	3565
novel.7121	NW_016559229.1	4844	7568	-	1646
novel.7122	NW_016559241.1	1306	1710	+	405
novel.7123	NW_016559247.1	271877	273257	+	1226
novel.249	NW_016527812.1	691338	713072	+	536
novel.248	NW_016527811.1	3125	3334	-	210
novel.243	NW_016527801.1	155604	156283	-	454
novel.242	NW_016527799.1	18078	18576	-	445
novel.241	NW_016527790.1	220679	222064	+	1386
novel.240	NW_016527782.1	151430	151646	+	217
novel.247	NW_016527811.1	32004	32459	+	317
novel.246	NW_016527808.1	48886	50012	-	759
novel.245	NW_016527808.1	27088	27510	-	397
novel.244	NW_016527807.1	380156	383647	+	367
novel.7657	NW_016569002.1	5969	7495	-	1527
novel.7654	NW_016569002.1	1	611	+	414
novel.7775	NW_016572001.1	29584	29898	+	277
novel.5029	NW_016542700.1	68150	71610	+	3272
novel.7719	NW_016570306.1	1	203	-	203
novel.1226	NW_016530013.1	216660	217007	-	293
novel.8008	NW_016578920.1	190945	191715	+	771
novel.8009	NW_016578976.1	18512	19064	-	204
novel.7713	NW_016570280.1	123182	125412	-	2180
novel.7712	NW_016570272.1	2394	3545	-	1095
novel.7711	NW_016570234.1	750	1354	+	605
novel.7710	NW_016570199.1	4445	5636	-	1192

novel.7717	NW_016570301.1	3665	3951	+	243
novel.7716	NW_016570298.1	4899	12722	-	210
novel.7715	NW_016570295.1	20971	43622	-	565
novel.7714	NW_016570280.1	126308	127144	-	787
novel.3878	NW_016538107.1	8217	16782	+	880
novel.3879	NW_016538107.1	8217	11600	-	3232
novel.3658	NW_016537382.1	52896	56807	-	3748
novel.3659	NW_016537382.1	51319	52313	-	943
novel.3872	NW_016538063.1	154997	161390	+	227
novel.3873	NW_016538093.1	82643	83154	-	458
novel.3870	NW_016538058.1	41431	43433	-	1952
novel.3655	NW_016537378.1	118674	119498	+	535
novel.3876	NW_016538106.1	82216	87492	-	3015
novel.3877	NW_016538107.1	4812	7334	+	2392
novel.3874	NW_016538095.1	41016	41673	+	386
novel.3875	NW_016538103.1	13812	15079	+	625
novel.4068	NW_016538759.1	260320	270849	+	2004
novel.4069	NW_016538772.1	9394	10962	-	423
novel.4060	NW_016538742.1	40381	118071	+	226
novel.1229	NW_016530028.1	93731	99675	+	5749
novel.4062	NW_016538754.1	31813	32077	-	212
novel.4063	NW_016538755.1	119061	119835	-	733
novel.4064	NW_016538755.1	286142	286567	-	370
novel.4065	NW_016538756.1	12298	21827	-	439
novel.4066	NW_016538757.1	93234	93512	+	227
novel.1228	NW_016530016.1	147882	150016	-	2023
novel.7271	NW_016561927.1	57031	57379	-	349
novel.8766	NW_016606611.1	1	5834	-	1289
novel.7272	NW_016561951.1	1376	1765	-	335
novel.556	NW_016528463.1	341935	383602	-	297
novel.6931	NW_016556935.1	44129	46997	-	507
novel.2291	NW_016532943.1	344274	344525	+	232
novel.2290	NW_016532933.1	214073	227819	+	1048
novel.2293	NW_016532946.1	2553	21201	-	664
novel.2292	NW_016532943.1	398335	399310	+	884
novel.2295	NW_016532951.1	22545	23145	+	601
novel.2294	NW_016532950.1	4317	9974	+	599
novel.2297	NW_016532953.1	10590	10890	-	278
novel.2296	NW_016532952.1	110124	110393	-	237
novel.2299	NW_016532973.1	25917	27489	+	255
novel.2298	NW_016532955.1	4870	5408	-	498
novel.5829	NW_016547554.1	19	1397	-	1358
novel.5828	NW_016547550.1	429833	435789	-	5957
novel.805	NW_016529116.1	373020	374918	-	208

novel.804	NW_016529116.1	234863	236319	+	883
novel.807	NW_016529119.1	1	1181	+	1181
novel.806	NW_016529116.1	577556	635558	-	5286
novel.801	NW_016529111.1	45268	46065	+	435
novel.800	NW_016529109.1	173481	188961	+	1712
novel.803	NW_016529111.1	9830	45049	-	974
novel.802	NW_016529111.1	132456	134844	+	358
novel.809	NW_016529133.1	166776	168322	-	241
novel.808	NW_016529119.1	547	1081	-	320
novel.4998	NW_016542575.1	204774	250422	+	858
novel.4999	NW_016542575.1	287846	289440	+	767
novel.4994	NW_016542559.1	733298	733766	-	301
novel.4995	NW_016542560.1	32730	36393	-	347
novel.4996	NW_016542562.1	3197	8073	+	4877
novel.4997	NW_016542574.1	113491	115381	+	268
novel.4990	NW_016542559.1	400015	401972	+	1958
novel.4991	NW_016542559.1	457118	460699	+	3221
novel.4992	NW_016542559.1	330044	332457	-	633
novel.4993	NW_016542559.1	453407	457286	-	1554
novel.3546	NW_016537069.1	2444	12352	-	555
novel.3547	NW_016537069.1	129477	131973	-	719
novel.4666	NW_016541231.1	528	1461	+	887
novel.4667	NW_016541231.1	6515	8687	-	429
novel.4664	NW_016541225.1	29406	46923	+	618
novel.4665	NW_016541227.1	61007	62298	-	1144
novel.4662	NW_016541195.1	60605	62184	-	1400
novel.4663	NW_016541219.1	6558	10662	-	515
novel.4660	NW_016541193.1	9	2260	+	2252
novel.4661	NW_016541194.1	9044	38731	+	716
novel.4668	NW_016541241.1	270205	273608	-	1511
novel.4669	NW_016541250.1	71850	72284	-	255
novel.6295	NW_016550788.1	42867	44480	+	1558
novel.6294	NW_016550788.1	16480	22767	+	6176
novel.6297	NW_016550788.1	12863	33314	-	775
novel.6296	NW_016550788.1	44652	46659	+	1853
novel.6291	NW_016550763.1	63231	65157	+	1871
novel.6290	NW_016550732.1	11640	12212	-	376
novel.1018	NW_016529592.1	297374	312281	+	217
novel.1019	NW_016529592.1	191386	192254	-	461
novel.1016	NW_016529584.1	377201	377545	+	345
novel.1017	NW_016529592.1	16074	25324	+	1356
novel.1014	NW_016529581.1	538544	541201	+	201
novel.1015	NW_016529581.1	566129	568006	-	1669
novel.1012	NW_016529574.1	57834	58541	-	653

novel.1013	NW_016529581.1	516349	521542	+	1361
novel.1010	NW_016529571.1	146339	263867	+	1067
novel.1011	NW_016529571.1	144411	146634	-	623
novel.4529	NW_016540675.1	68182	70693	+	255
novel.4528	NW_016540664.1	12888	13794	-	634
novel.4525	NW_016540662.1	353089	356893	+	409
novel.4524	NW_016540656.1	61780	62046	-	244
novel.4527	NW_016540664.1	19130	19850	+	664
novel.4526	NW_016540664.1	17088	17756	+	615
novel.4521	NW_016540656.1	10000	10600	+	430
novel.4520	NW_016540643.1	56365	58827	+	1913
novel.4523	NW_016540656.1	30040	30639	-	600
novel.4522	NW_016540656.1	63395	63850	+	432
novel.6749	NW_016555084.1	607	1114	-	508
novel.6748	NW_016555063.1	4955	5569	-	615
novel.6747	NW_016555055.1	1	1899	-	1899
novel.6746	NW_016555053.1	60053	61752	+	218
novel.6745	NW_016555050.1	163068	165347	+	207
novel.6744	NW_016555034.1	9873	10519	-	591
novel.6743	NW_016555026.1	110931	115723	+	782
novel.6742	NW_016555009.1	248912	249712	-	562
novel.6741	NW_016554977.1	1	469	-	335
novel.6740	NW_016554961.1	1	1287	+	1239
novel.650	NW_016528687.1	1578	2030	+	453
novel.651	NW_016528689.1	13082	13339	-	208
novel.652	NW_016528690.1	1	1142	-	1142
novel.653	NW_016528691.1	159223	178587	+	817
novel.654	NW_016528702.1	248211	251206	-	480
novel.655	NW_016528706.1	89469	120042	+	1095
novel.656	NW_016528710.1	144226	144704	+	479
novel.657	NW_016528711.1	334154	340687	-	311
novel.658	NW_016528711.1	416511	417431	-	558
novel.659	NW_016528712.1	293213	293417	+	205
novel.4297	NW_016539808.1	127728	129592	-	319
novel.4296	NW_016539808.1	51805	53421	-	1617
novel.4295	NW_016539793.1	18625	30377	-	301
novel.4294	NW_016539787.1	4	659	+	656
novel.4293	NW_016539779.1	11098	11663	-	512
novel.4292	NW_016539774.1	73314	91107	+	3258
novel.4291	NW_016539766.1	8576	9914	-	891
novel.4290	NW_016539748.1	23930	24240	-	256
novel.4299	NW_016539817.1	16352	20608	+	686
novel.4298	NW_016539808.1	156935	158348	-	871
novel.7362	NW_016563357.1	425	1079	-	598

novel.7363	NW_016563365.1	82	38687	+	939
novel.7360	NW_016563317.1	1	1023	-	941
novel.7361	NW_016563337.1	2415	4860	-	617
novel.7366	NW_016563402.1	12	748	+	737
novel.7367	NW_016563411.1	843	1597	+	288
novel.7364	NW_016563365.1	2214	2486	+	273
novel.7365	NW_016563373.1	3120	3517	+	398
novel.7368	NW_016563437.1	1686	1968	-	283
novel.7369	NW_016563437.1	2970	4953	-	289
novel.7963	NW_016577782.1	4618	4883	-	244
novel.1908	NW_016531733.1	165206	166888	-	1683
novel.1909	NW_016531733.1	319937	322196	-	415
novel.1904	NW_016531721.1	64980	67567	+	1060
novel.1905	NW_016531721.1	33748	34119	-	336
novel.1906	NW_016531728.1	2622	7603	+	4961
novel.1907	NW_016531728.1	15522	17092	-	1519
novel.1900	NW_016531709.1	49461	52158	+	1026
novel.1901	NW_016531709.1	49637	50428	-	792
novel.1902	NW_016531712.1	64324	64716	+	313
novel.1903	NW_016531713.1	69797	70255	-	343
novel.5218	NW_016543701.1	13700	17089	+	3390
novel.5219	NW_016543714.1	130687	132442	-	1756
novel.5214	NW_016543690.1	311820	314850	+	869
novel.5215	NW_016543690.1	297564	304499	-	5501
novel.5216	NW_016543698.1	9650	37841	+	881
novel.5217	NW_016543698.1	34858	35251	-	281
novel.5210	NW_016543679.1	28415	29720	-	454
novel.5211	NW_016543679.1	105510	106725	-	1216
novel.5212	NW_016543690.1	134860	135444	+	293
novel.5213	NW_016543690.1	297564	304499	+	6159
novel.2844	NW_016534591.1	228955	230493	+	1539
novel.2845	NW_016534591.1	419709	422043	+	1958
novel.2846	NW_016534591.1	636149	637490	+	264
novel.2847	NW_016534591.1	29944	30931	-	988
novel.2840	NW_016534588.1	68869	69315	+	391
novel.2841	NW_016534588.1	72529	72849	+	264
novel.2842	NW_016534588.1	95211	95991	+	739
novel.2843	NW_016534591.1	181117	181989	+	873
novel.2848	NW_016534593.1	25515	31043	+	2719
novel.2849	NW_016534611.1	19004	21312	-	224
novel.7506	NW_016566125.1	29778	30130	-	298
novel.7507	NW_016566178.1	71870	72677	+	612
novel.7504	NW_016566114.1	1619	3084	-	1425
novel.8636	NW_016603723.1	994	2797	-	527

novel.8631	NW_016603460.1	20	2499	-	2480
novel.7503	NW_016566114.1	15	1125	+	1111
novel.7500	NW_016566084.1	454	915	+	406
novel.7501	NW_016566093.1	258769	260708	+	1883
novel.8639	NW_016603784.1	408	1380	-	311
novel.8638	NW_016603772.1	1305	2910	-	1224
novel.7508	NW_016566178.1	71356	71752	-	301
novel.7509	NW_016566200.1	2675	4019	+	1296
novel.2642	NW_016533949.1	117152	117963	+	767
novel.2643	NW_016533949.1	50113	50445	-	333
novel.2640	NW_016533949.1	49259	50445	+	387
novel.5531	NW_016545566.1	339470	341869	-	2400
novel.2641	NW_016533949.1	114555	117039	+	2456
novel.5530	NW_016545566.1	303756	304438	-	627
novel.2646	NW_016533973.1	153088	159897	-	1375
novel.5533	NW_016545568.1	22167	23081	+	858
novel.2647	NW_016533982.1	59976	60452	+	349
novel.5532	NW_016545566.1	410649	411851	-	289
novel.2644	NW_016533962.1	3766	4829	+	873
novel.5535	NW_016545572.1	381	881	+	501
novel.2645	NW_016533973.1	134440	135427	-	762
novel.5534	NW_016545568.1	24253	27268	-	1165
novel.5537	NW_016545580.1	189226	189628	+	403
novel.5536	NW_016545572.1	10912	17928	+	580
novel.2648	NW_016533982.1	87119	89730	+	253
novel.5969	NW_016548567.1	88184	90631	-	1265
novel.4258	NW_016539620.1	41788	42628	+	310
novel.3465	NW_016536712.1	259014	259800	+	787
novel.3464	NW_016536704.1	307807	308230	-	246
novel.3467	NW_016536712.1	311323	327041	-	733
novel.3466	NW_016536712.1	189702	190197	-	451
novel.3461	NW_016536696.1	315771	316207	-	266
novel.3460	NW_016536689.1	43551	54946	-	748
novel.3463	NW_016536704.1	231079	231759	+	368
novel.3462	NW_016536703.1	76967	77237	+	221
novel.3469	NW_016536720.1	101177	102030	-	242
novel.3468	NW_016536720.1	72101	72631	-	248
novel.2598	NW_016533826.1	285691	288588	-	455
novel.2599	NW_016533827.1	237241	237447	+	207
novel.2596	NW_016533826.1	293892	294541	+	621
novel.2597	NW_016533826.1	921534	922036	+	286
novel.2594	NW_016533823.1	35030	35337	-	252
novel.2595	NW_016533826.1	64697	94088	+	4270
novel.2592	NW_016533818.1	371649	372622	-	851

novel.2593	NW_016533822.1	78890	79348	+	288
novel.2590	NW_016533818.1	316620	317338	+	719
novel.2591	NW_016533818.1	369185	372622	+	913
novel.4859	NW_016541988.1	26742	27492	-	696
novel.4858	NW_016541978.1	60140	61357	+	833
novel.4855	NW_016541973.1	118166	118876	-	563
novel.4854	NW_016541973.1	82876	83149	-	222
novel.4857	NW_016541976.1	7174	7598	-	319
novel.4856	NW_016541973.1	320938	321957	-	820
novel.4851	NW_016541959.1	57480	57832	-	353
novel.4850	NW_016541940.1	12968	13357	-	333
novel.4853	NW_016541973.1	81792	82722	-	931
novel.4852	NW_016541973.1	283245	284260	+	993
novel.2367	NW_016533169.1	1949	2177	+	229
novel.2366	NW_016533163.1	7616	8160	-	262
novel.1544	NW_016530746.1	270086	270345	-	208
novel.5458	NW_016545217.1	15861	16783	+	923
novel.5459	NW_016545225.1	105195	105512	-	261
novel.1545	NW_016530750.1	113677	117098	+	3158
novel.5452	NW_016545133.1	5322	16783	+	242
novel.2333	NW_016533070.1	62925	64075	+	603
novel.5450	NW_016545123.1	806	1212	+	328
novel.5451	NW_016545123.1	33575	60111	-	1151
novel.5456	NW_016545207.1	4898	5214	-	317
novel.1546	NW_016530750.1	116157	117183	-	974
novel.5454	NW_016545158.1	32404	33564	-	1105
novel.5455	NW_016545196.1	60763	61127	-	308
novel.1547	NW_016530751.1	41939	42448	-	510
novel.1540	NW_016530732.1	498350	500925	-	201
novel.1541	NW_016530733.1	66259	67385	+	1127
novel.1542	NW_016530733.1	518184	519688	-	922
novel.1543	NW_016530742.1	9141	17442	-	826
novel.7617	NW_016568520.1	2792	3149	-	336
novel.5202	NW_016543637.1	1	1100	-	784
novel.8022	NW_016579562.1	821	1092	+	239
novel.8024	NW_016579614.1	20721	25334	-	4614
novel.8025	NW_016579699.1	4	7328	+	6347
novel.1739	NW_016531357.1	617517	618172	+	302
novel.1738	NW_016531356.1	4722	9362	-	249
novel.1733	NW_016531326.1	58318	58614	+	297
novel.1732	NW_016531317.1	6371	6711	+	220
novel.1731	NW_016531314.1	26700	28151	+	1452
novel.1730	NW_016531310.1	424580	426271	-	406
novel.1737	NW_016531351.1	168958	180870	+	284

novel.1736	NW_016531346.1	251152	251678	-	527
novel.1735	NW_016531346.1	251152	251678	+	527
novel.1734	NW_016531331.1	29952	40695	-	721
novel.1225	NW_016530013.1	216396	216597	-	202
novel.1224	NW_016529995.1	22043	22289	-	204
novel.1227	NW_016530016.1	178323	178607	+	230
novel.5028	NW_016542700.1	63873	66384	+	1944
novel.1221	NW_016529986.1	132811	133077	+	267
novel.1220	NW_016529980.1	3526	4341	+	776
novel.1223	NW_016529994.1	7924	10609	+	1739
novel.1222	NW_016529989.1	25624	25952	-	329
novel.5023	NW_016542687.1	65161	72819	-	354
novel.5022	NW_016542679.1	15540	28552	-	2563
novel.5021	NW_016542669.1	199141	199701	+	509
novel.5020	NW_016542659.1	38226	38515	-	290
novel.5027	NW_016542695.1	1	1838	-	1838
novel.5026	NW_016542695.1	1	1839	+	1839
novel.5025	NW_016542693.1	7466	13106	-	5641
novel.5024	NW_016542688.1	90423	90862	+	283
novel.2394	NW_016533236.1	83137	83737	-	544
novel.2395	NW_016533239.1	15550	15845	-	263
novel.2396	NW_016533241.1	73480	73968	+	328
novel.2397	NW_016533243.1	155934	157269	+	297
novel.2390	NW_016533234.1	100451	100743	+	245
novel.8086	NW_016581784.1	111570	112324	+	703
novel.2391	NW_016533235.1	9260	12801	-	3011
novel.6822	NW_016555795.1	11166	13152	-	1987
novel.2392	NW_016533236.1	7615	23927	+	3780
novel.6823	NW_016555795.1	16428	17499	-	1072
novel.2393	NW_016533236.1	3542	3868	-	284
novel.6820	NW_016555795.1	4951	5853	-	728
novel.3673	NW_016537445.1	102206	102886	-	681
novel.8087	NW_016581785.1	87	506	+	370
novel.3674	NW_016537446.1	24861	29813	-	547
novel.3675	NW_016537451.1	27483	32004	+	353
novel.6824	NW_016555819.1	5530	6344	-	765
novel.6825	NW_016555835.1	950	1378	-	381
novel.3678	NW_016537456.1	355408	363400	-	1171
novel.8084	NW_016581781.1	485	785	+	277
novel.8601	NW_016602645.1	631	2409	+	1779
novel.6828	NW_016555841.1	47489	47862	+	344
novel.6829	NW_016555841.1	47493	48057	-	370
novel.8356	NW_016591194.1	610	2461	+	222
novel.8357	NW_016591201.1	7306	8333	-	718

novel.8085	NW_016581784.1	109844	111367	+	842
novel.8354	NW_016591154.1	11918	12252	+	280
novel.8607	NW_016602744.1	582	1760	-	771
novel.8020	NW_016579494.1	1041	1401	-	331
novel.8021	NW_016579528.1	1	629	+	603
novel.8082	NW_016581765.1	1	786	-	753
novel.8023	NW_016579563.1	5573	6010	+	400
novel.7779	NW_016572144.1	15939	18745	+	230
novel.7778	NW_016572070.1	4403	5729	-	297
novel.8026	NW_016579773.1	768	1282	+	438
novel.8027	NW_016579801.1	4001	4245	-	245
novel.8028	NW_016579801.1	4339	4855	-	477
novel.7774	NW_016571991.1	11340	11758	-	224
novel.7777	NW_016572057.1	18524	19738	-	1215
novel.7776	NW_016572019.1	573	845	-	237
novel.7771	NW_016571985.1	1	811	-	811
novel.7770	NW_016571985.1	1	811	+	811
novel.7773	NW_016571990.1	10766	11256	-	469
novel.7772	NW_016571987.1	380	1577	+	1176
novel.3670	NW_016537435.1	83205	89824	-	1614
novel.3671	NW_016537435.1	123405	144075	-	4340
novel.3672	NW_016537440.1	120195	122438	+	844
novel.6821	NW_016555795.1	8654	9263	-	610
novel.3858	NW_016538043.1	96778	97020	+	207
novel.3859	NW_016538048.1	6687	8138	-	1452
novel.3676	NW_016537451.1	27688	28265	+	578
novel.3677	NW_016537456.1	311301	311512	+	212
novel.3854	NW_016538003.1	140192	144396	-	248
novel.3855	NW_016538006.1	45505	46334	+	773
novel.3856	NW_016538006.1	45208	45624	-	291
novel.3857	NW_016538014.1	14293	16300	-	2008
novel.3850	NW_016537974.1	80344	80785	-	387
novel.3851	NW_016537978.1	324	978	-	607
novel.3852	NW_016537986.1	11626	11928	+	269
novel.3853	NW_016537988.1	3435	21628	+	751
novel.8083	NW_016581765.1	1443	1918	-	425
novel.4082	NW_016538840.1	74470	75163	+	521
novel.4083	NW_016538856.1	75346	76812	+	1467
novel.4080	NW_016538822.1	85213	87100	+	297
novel.4081	NW_016538822.1	185001	187552	+	1276
novel.4086	NW_016538861.1	113045	162315	+	222
novel.4087	NW_016538862.1	253195	255169	-	385
novel.4084	NW_016538856.1	233326	233927	+	602
novel.4085	NW_016538856.1	141958	149386	-	549

novel.4088	NW_016538865.1	94110	94509	+	346
novel.4089	NW_016538865.1	94044	94384	-	289
novel.8080	NW_016581750.1	4265	5200	+	879
novel.8284	NW_016588272.1	8200	9810	-	1519
novel.1896	NW_016531704.1	368801	369080	-	224
novel.8286	NW_016588496.1	1	4960	-	220
novel.8287	NW_016588525.1	1	421	-	387
novel.8280	NW_016587983.1	2813	3658	+	800
novel.8281	NW_016588069.1	22	5595	+	414
novel.8282	NW_016588248.1	2862	3794	+	272
novel.8283	NW_016588256.1	5263	5838	+	549
novel.8288	NW_016588648.1	4127	12481	+	1800
novel.1894	NW_016531699.1	222246	222494	+	249
novel.1895	NW_016531704.1	671368	671751	+	384
novel.1892	NW_016531693.1	19747	20045	-	273
novel.8081	NW_016581765.1	4101	8723	+	2508
novel.1893	NW_016531693.1	197623	204254	-	372
novel.1890	NW_016531688.1	145929	151469	+	1176
novel.1891	NW_016531693.1	68732	71140	+	366
novel.7199	NW_016560441.1	4347	4782	+	436
novel.7198	NW_016560441.1	1	5411	+	600
novel.7191	NW_016560284.1	5245	6523	+	1279
novel.7190	NW_016560230.1	11210	30088	+	239
novel.7193	NW_016560336.1	27933	28973	+	996
novel.7192	NW_016560309.1	18081	19275	+	1147
novel.7195	NW_016560387.1	27731	28276	+	512
novel.7194	NW_016560336.1	207119	211495	+	1550
novel.7197	NW_016560439.1	5147	6224	-	1023
novel.7196	NW_016560387.1	27731	28276	-	415
novel.4430	NW_016540332.1	842075	843106	-	629
novel.2101	NW_016532265.1	250187	251448	+	286
novel.2100	NW_016532265.1	159346	161092	+	1747
novel.2103	NW_016532265.1	292378	298303	+	400
novel.2102	NW_016532265.1	287148	292241	+	1203
novel.2105	NW_016532265.1	482660	484172	+	436
novel.2104	NW_016532265.1	336490	338673	+	1615
novel.2107	NW_016532265.1	478483	479237	-	519
novel.2106	NW_016532265.1	468034	469180	-	1147
novel.2109	NW_016532265.1	562151	564778	-	2220
novel.2108	NW_016532265.1	559196	560017	-	822
novel.3544	NW_016537061.1	16514	75299	+	1480
novel.8285	NW_016588280.1	326	3197	+	2664
novel.5599	NW_016546017.1	117957	118559	-	603
novel.5598	NW_016546017.1	398505	412191	+	210

novel.5593	NW_016545998.1	5964	40633	-	7310
novel.5592	NW_016545998.1	22416	25418	+	2804
novel.5591	NW_016545978.1	2852	15966	+	232
novel.5590	NW_016545972.1	64020	65562	-	1370
novel.5597	NW_016546017.1	195289	202670	+	914
novel.5596	NW_016546013.1	162755	177228	-	4128
novel.5595	NW_016546013.1	161310	163259	+	1894
novel.5594	NW_016546002.1	22676	23294	-	238
novel.5845	NW_016547635.1	4320	5255	-	936
novel.5844	NW_016547623.1	28823	29233	-	411
novel.829	NW_016529180.1	46941	48224	+	1284
novel.828	NW_016529179.1	403587	404675	+	442
novel.5841	NW_016547612.1	120702	121848	-	305
novel.5840	NW_016547594.1	29169	38262	-	814
novel.5843	NW_016547623.1	28762	29233	+	472
novel.5842	NW_016547619.1	5420	5623	+	204
novel.823	NW_016529161.1	220859	222049	-	552
novel.822	NW_016529161.1	134205	134855	+	594
novel.821	NW_016529161.1	52916	53671	+	244
novel.820	NW_016529152.1	465149	483291	-	309
novel.827	NW_016529179.1	85272	85472	+	201
novel.826	NW_016529175.1	6214	6553	+	340
novel.825	NW_016529167.1	31686	32127	-	385
novel.824	NW_016529167.1	102024	102436	+	277
novel.8289	NW_016588648.1	1892	5148	-	1113
novel.3525	NW_016537007.1	20967	21176	+	210
novel.2675	NW_016534053.1	209474	234331	-	8151
novel.1880	NW_016531672.1	10313	11200	+	888
novel.4648	NW_016541147.1	312834	322207	+	201
novel.4649	NW_016541147.1	323428	323891	+	410
novel.4640	NW_016541109.1	223400	231001	+	2863
novel.4641	NW_016541115.1	91647	93473	-	1827
novel.4642	NW_016541121.1	28548	29146	-	493
novel.4643	NW_016541132.1	65	1578	-	1514
novel.4644	NW_016541134.1	44971	45261	-	291
novel.4645	NW_016541136.1	7205	15987	+	345
novel.4646	NW_016541136.1	169685	267611	+	263
novel.4647	NW_016541138.1	10995	29757	-	1061
novel.1030	NW_016529605.1	88516	89483	+	728
novel.1031	NW_016529605.1	247698	248767	-	248
novel.1032	NW_016529605.1	297758	306852	-	490
novel.1033	NW_016529605.1	336672	360734	-	212
novel.1034	NW_016529607.1	9085	12292	-	2786
novel.1035	NW_016529611.1	10998	13924	-	2866

novel.1036	NW_016529617.1	41420	41820	+	401
novel.1037	NW_016529617.1	41901	42618	+	694
novel.1038	NW_016529617.1	45824	46167	-	288
novel.1039	NW_016529619.1	284195	284874	-	680
novel.4507	NW_016540599.1	135413	150710	-	496
novel.4506	NW_016540594.1	268445	269692	-	1060
novel.4505	NW_016540593.1	66151	66483	-	333
novel.4504	NW_016540593.1	58239	58459	-	221
novel.4503	NW_016540593.1	54891	55924	+	592
novel.4502	NW_016540591.1	112479	113566	+	455
novel.4501	NW_016540591.1	103070	103284	+	215
novel.4500	NW_016540582.1	217437	217842	-	213
novel.4509	NW_016540604.1	28723	29147	-	425
novel.4508	NW_016540603.1	286766	287987	-	280
novel.636	NW_016528661.1	411942	412967	-	1004
novel.637	NW_016528663.1	26487	31128	-	4642
novel.634	NW_016528658.1	2079	3626	-	274
novel.635	NW_016528661.1	261573	278826	-	571
novel.632	NW_016528653.1	69374	70247	+	874
novel.633	NW_016528653.1	197681	207359	-	528
novel.630	NW_016528648.1	6347	6783	-	328
novel.631	NW_016528653.1	992	2351	+	1360
novel.638	NW_016528676.1	609279	609770	+	492
novel.639	NW_016528677.1	24660	31264	+	755
novel.7252	NW_016561476.1	10091	10695	+	569
novel.1698	NW_016531244.1	22179	22749	-	571
novel.1699	NW_016531248.1	78884	80386	-	1278
novel.1694	NW_016531226.1	33641	34962	-	1269
novel.1695	NW_016531236.1	43084	43772	+	632
novel.1696	NW_016531244.1	11210	14783	+	696
novel.1697	NW_016531244.1	14312	15254	-	839
novel.1690	NW_016531226.1	27175	36113	+	1994
novel.1691	NW_016531226.1	27912	30874	+	779
novel.1692	NW_016531226.1	29855	33518	+	266
novel.1693	NW_016531226.1	26619	36113	-	315
novel.7253	NW_016561476.1	5074	6065	-	828
novel.7696	NW_016569945.1	1147	1849	+	512
novel.7697	NW_016569951.1	1217	3504	-	2231
novel.7694	NW_016569924.1	40305	40600	-	240
novel.7695	NW_016569941.1	1078	1744	+	667
novel.7692	NW_016569833.1	6697	14009	+	332
novel.7693	NW_016569902.1	3437	7593	+	511
novel.7690	NW_016569820.1	1	1120	+	1064
novel.7691	NW_016569820.1	1	1101	-	1101

novel.7348	NW_016563173.1	27681	28260	-	580
novel.7349	NW_016563179.1	5932	13763	+	7775
novel.7698	NW_016569951.1	3978	5347	-	1286
novel.7699	NW_016570068.1	1015	1360	+	292
novel.1966	NW_016531924.1	12299	12789	-	469
novel.1967	NW_016531925.1	147747	165006	-	1090
novel.1964	NW_016531924.1	5654	6894	+	1013
novel.1965	NW_016531924.1	5654	16980	-	1937
novel.1962	NW_016531917.1	32902	39226	-	640
novel.1963	NW_016531917.1	51958	52758	-	801
novel.1960	NW_016531916.1	77596	109687	+	589
novel.1961	NW_016531917.1	157142	157557	+	416
novel.1968	NW_016531931.1	12842	15603	+	262
novel.1969	NW_016531931.1	36361	41855	+	242
novel.5276	NW_016544009.1	80815	84155	-	266
novel.5277	NW_016544033.1	138410	141650	+	1363
novel.5274	NW_016544006.1	63253	64905	+	206
novel.5275	NW_016544009.1	110637	111035	+	301
novel.5272	NW_016543992.1	159876	165724	-	498
novel.5273	NW_016543997.1	36349	38123	-	580
novel.5270	NW_016543991.1	10877	11378	-	502
novel.5271	NW_016543992.1	159747	160293	+	479
novel.5278	NW_016544033.1	126093	126442	-	291
novel.5279	NW_016544053.1	110760	112649	+	455
novel.8349	NW_016591011.1	21478	75630	+	272
novel.8348	NW_016590898.1	42	611	-	544
novel.8619	NW_016603142.1	170	2355	+	314
novel.8618	NW_016603085.1	1440	2582	+	1123
novel.8341	NW_016590635.1	22660	23508	+	758
novel.8340	NW_016590585.1	352	2106	+	316
novel.8343	NW_016590667.1	128	1013	+	829
novel.8342	NW_016590635.1	21665	22857	-	1059
novel.8613	NW_016602835.1	332	1395	+	1064
novel.8344	NW_016590710.1	1081	1441	-	305
novel.8347	NW_016590840.1	399	754	+	299
novel.8610	NW_016602763.1	1	861	-	861
novel.3999	NW_016538476.1	24949	25502	-	413
novel.3998	NW_016538476.1	15621	16880	-	676
novel.3995	NW_016538473.1	449873	450373	+	501
novel.3994	NW_016538463.1	9955	10507	+	553
novel.3997	NW_016538473.1	473160	473762	-	392
novel.3996	NW_016538473.1	466571	466873	+	248
novel.3991	NW_016538461.1	16196	16856	-	605
novel.3990	NW_016538461.1	8811	9083	-	216

novel.3993	NW_016538463.1	9138	9386	+	249
novel.3992	NW_016538461.1	117521	119162	-	1530
novel.3195	NW_016535749.1	19454	20970	-	1464
novel.3194	NW_016535749.1	17374	19404	-	2031
novel.3197	NW_016535783.1	494	777	+	284
novel.3196	NW_016535777.1	71697	72559	+	200
novel.3191	NW_016535749.1	25036	26489	+	812
novel.3190	NW_016535745.1	87608	88470	-	421
novel.3193	NW_016535749.1	12296	15787	-	2765
novel.3192	NW_016535749.1	28729	29034	+	253
novel.3199	NW_016535795.1	7556	9619	+	1857
novel.3198	NW_016535794.1	86651	92101	+	1698
novel.2828	NW_016534502.1	242494	243176	+	483
novel.2829	NW_016534502.1	242071	242590	-	264
novel.2826	NW_016534501.1	783	8586	-	1066
novel.2827	NW_016534502.1	241666	242297	+	368
novel.2824	NW_016534501.1	287214	287989	+	238
novel.2825	NW_016534501.1	378404	414172	+	1164
novel.2822	NW_016534491.1	14082	14888	+	705
novel.2823	NW_016534495.1	10487	12726	-	2130
novel.2820	NW_016534488.1	186351	192822	-	3888
novel.2821	NW_016534488.1	374375	376249	-	1731
novel.4705	NW_016541341.1	3956	6464	+	2509
novel.3526	NW_016537015.1	715504	715710	+	207
novel.4704	NW_016541337.1	28881	141183	-	747
novel.3339	NW_016536275.1	9177	15806	-	206
novel.3338	NW_016536275.1	16268	16777	+	482
novel.6129	NW_016549683.1	30265	32110	-	717
novel.6128	NW_016549653.1	5074	5336	-	227
novel.3331	NW_016536251.1	537	1055	+	519
novel.3330	NW_016536248.1	142988	144401	-	1414
novel.3333	NW_016536252.1	4979	8649	+	3557
novel.3444	NW_016536651.1	461	755	-	295
novel.3443	NW_016536648.1	9306	9951	-	562
novel.3442	NW_016536645.1	113018	138953	-	275
novel.3337	NW_016536264.1	61975	62230	-	201
novel.3336	NW_016536263.1	4030	5286	-	1213
novel.3458	NW_016536689.1	8	937	-	930
novel.3459	NW_016536689.1	3953	5268	-	1316
novel.4877	NW_016542069.1	232	701	+	470
novel.4876	NW_016542064.1	10103	52526	-	452
novel.4875	NW_016542057.1	4186	4413	-	228
novel.4874	NW_016542056.1	1529	4311	+	598
novel.4873	NW_016542054.1	24515	26939	+	2322

novel.4872	NW_016542039.1	2218	2809	+	571
novel.4871	NW_016542035.1	21713	21976	+	212
novel.4870	NW_016542031.1	10016	23902	+	1320
novel.4709	NW_016541342.1	29331	32945	-	3615
novel.4879	NW_016542070.1	24165	25425	+	950
novel.4878	NW_016542069.1	1207	1886	+	624
novel.3450	NW_016536661.1	6935	7551	-	576
novel.3451	NW_016536661.1	7698	8350	-	575
novel.3452	NW_016536661.1	8579	10073	-	1384
novel.3321	NW_016536215.1	16016	26168	-	1142
novel.3326	NW_016536227.1	31533	35333	-	535
novel.3327	NW_016536231.1	399451	409115	-	503
novel.3324	NW_016536225.1	533151	533930	-	546
novel.3325	NW_016536227.1	30791	31372	-	582
novel.3522	NW_016536986.1	522333	524033	-	1647
novel.2303	NW_016532998.1	56971	57669	-	229
novel.3523	NW_016536987.1	74450	80506	+	264
novel.7014	NW_016557850.1	1819	2680	+	832
novel.5478	NW_016545347.1	1	1103	-	1103
novel.5479	NW_016545355.1	208448	210086	+	1451
novel.7015	NW_016557869.1	2065	3157	+	446
novel.5474	NW_016545335.1	24979	25470	+	464
novel.5475	NW_016545335.1	38277	72738	+	543
novel.5476	NW_016545343.1	13687	15567	+	318
novel.5477	NW_016545346.1	19055	44864	-	278
novel.2310	NW_016533016.1	177762	178757	+	560
novel.2311	NW_016533020.1	165458	173431	+	233
novel.5472	NW_016545315.1	5752	6482	+	397
novel.5473	NW_016545323.1	290263	291513	-	402
novel.8096	NW_016582153.1	1040	2434	-	928
novel.7010	NW_016557812.1	12471	13288	-	527
novel.7011	NW_016557830.1	11270	13799	-	1352
novel.7012	NW_016557840.1	1	835	+	835
novel.7013	NW_016557840.1	1	5527	-	337
novel.7018	NW_016557892.1	4864	6012	+	1149
novel.7019	NW_016557911.1	47512	48125	-	614
novel.1759	NW_016531376.1	4459	5451	-	247
novel.1758	NW_016531376.1	1712	3974	-	594
novel.1209	NW_016529952.1	44307	45019	+	669
novel.1208	NW_016529952.1	15897	35717	+	575
novel.1207	NW_016529950.1	244373	247132	-	2720
novel.1754	NW_016531376.1	4263	5436	+	428
novel.1205	NW_016529949.1	521862	522160	-	212
novel.1204	NW_016529949.1	449484	451893	-	219

novel.1203	NW_016529949.1	296539	306180	-	725
novel.1202	NW_016529947.1	12009	12240	+	207
novel.1201	NW_016529938.1	477259	478587	-	1329
novel.1200	NW_016529938.1	477259	478587	+	1098
novel.5049	NW_016542797.1	1845	2297	-	409
novel.5048	NW_016542797.1	1507	1747	-	241
novel.5045	NW_016542765.1	113881	116590	+	874
novel.5044	NW_016542761.1	75451	100131	+	451
novel.5047	NW_016542781.1	109857	110117	+	261
novel.5046	NW_016542766.1	65838	66971	+	721
novel.5041	NW_016542734.1	35362	35777	-	361
novel.5040	NW_016542728.1	235948	238568	-	2621
novel.5043	NW_016542759.1	20636	20955	+	320
novel.5042	NW_016542759.1	19909	20323	+	366
novel.2304	NW_016533003.1	43955	44935	+	744
novel.287	NW_016527918.1	180454	180865	-	412
novel.286	NW_016527914.1	817552	818074	+	523
novel.285	NW_016527914.1	488299	512250	+	413
novel.284	NW_016527913.1	11	1059	+	805
novel.283	NW_016527909.1	6512	7849	+	1293
novel.282	NW_016527907.1	267182	267501	-	213
novel.281	NW_016527907.1	266819	267062	-	244
novel.280	NW_016527907.1	191002	192386	-	824
novel.8765	NW_016606606.1	4349	6373	+	2025
novel.289	NW_016527920.1	370862	377092	-	897
novel.288	NW_016527919.1	260086	263100	+	266
novel.6468	NW_016552400.1	17650	18812	+	496
novel.6469	NW_016552420.1	1373	5822	+	531
novel.6462	NW_016552317.1	150332	151310	-	848
novel.6463	NW_016552324.1	2498	2773	+	219
novel.6460	NW_016552311.1	5365	6525	-	265
novel.6461	NW_016552317.1	31361	107619	-	1066
novel.6466	NW_016552353.1	21179	32579	+	453
novel.6467	NW_016552355.1	8269	13189	+	2156
novel.6464	NW_016552340.1	67064	67275	-	212
novel.6465	NW_016552345.1	120714	130402	-	1037
novel.8761	NW_016606442.1	1037	5970	-	1011
novel.8760	NW_016606413.1	1	4582	+	4582
novel.8523	NW_016599364.1	1	1650	-	1121
novel.4312	NW_016539892.1	1274	6864	+	957
novel.4313	NW_016539892.1	1274	6864	-	696
novel.4310	NW_016539882.1	4748	5455	-	660
novel.4311	NW_016539888.1	90060	95700	-	470
novel.4316	NW_016539904.1	433464	433763	+	300

novel.4317	NW_016539904.1	348927	350031	-	543
novel.4314	NW_016539902.1	30691	41190	+	662
novel.4315	NW_016539904.1	265259	291372	+	222
novel.4318	NW_016539910.1	5334	9594	-	552
novel.4319	NW_016539921.1	64736	65383	+	602
novel.5847	NW_016547666.1	11991	12212	-	222
novel.7757	NW_016571377.1	32456	35265	-	2393
novel.7756	NW_016571371.1	13037	28639	-	320
novel.7755	NW_016571366.1	5964	6203	+	204
novel.7754	NW_016571366.1	5112	5909	+	798
novel.7753	NW_016571325.1	12875	13142	-	237
novel.7752	NW_016571325.1	11575	13021	+	910
novel.7751	NW_016571172.1	1	2386	-	1262
novel.7750	NW_016571133.1	2288	2591	+	269
novel.7759	NW_016571555.1	1	237	-	237
novel.7758	NW_016571434.1	492	1007	-	486
novel.6804	NW_016555645.1	145969	150886	-	1135
novel.6805	NW_016555646.1	1	375	+	331
novel.6806	NW_016555646.1	1166	3923	-	501
novel.6807	NW_016555656.1	30368	34010	+	1286
novel.6800	NW_016555614.1	4813	11226	-	229
novel.6801	NW_016555635.1	1116	92688	+	613
novel.6802	NW_016555635.1	48827	51979	-	636
novel.3972	NW_016538394.1	4077	6861	+	2785
novel.6808	NW_016555656.1	56524	58088	+	293
novel.6809	NW_016555656.1	85362	86943	+	1582
novel.3838	NW_016537928.1	23350	23786	-	285
novel.3839	NW_016537932.1	5023	5818	+	796
novel.3970	NW_016538387.1	90232	94857	-	4626
novel.7163	NW_016559738.1	8577	9430	-	854
novel.3793	NW_016537782.1	339721	354790	-	1765
novel.3976	NW_016538413.1	199224	202366	+	289
novel.3791	NW_016537779.1	510107	510731	-	281
novel.5849	NW_016547670.1	125847	126392	-	546
novel.3974	NW_016538404.1	439631	440318	-	643
novel.5848	NW_016547670.1	125847	126392	+	546
novel.7270	NW_016561927.1	42162	57426	+	526
novel.7164	NW_016559738.1	9575	12561	-	2879
novel.8546	NW_016600397.1	654	1824	+	416
novel.8545	NW_016600397.1	8	568	+	561
novel.7273	NW_016561960.1	42282	42782	-	458
novel.7274	NW_016561993.1	48939	52701	+	930
novel.7275	NW_016561993.1	72520	73784	+	306
novel.7276	NW_016562033.1	44922	47747	-	2088

novel.7277	NW_016562046.1	390	1138	-	204
novel.1743	NW_016531358.1	1443	1740	+	242
novel.2129	NW_016532349.1	31214	33144	-	268
novel.2128	NW_016532346.1	222199	223669	+	1471
novel.2123	NW_016532310.1	66524	76476	+	1288
novel.2122	NW_016532305.1	1842	7796	-	230
novel.2121	NW_016532305.1	137912	139498	+	1560
novel.2120	NW_016532296.1	18473	18677	-	205
novel.2127	NW_016532341.1	46223	52459	-	280
novel.2126	NW_016532327.1	165065	166356	-	546
novel.2125	NW_016532327.1	163894	177514	-	200
novel.2124	NW_016532325.1	57592	69956	+	367
novel.8408	NW_016592842.1	1	1046	-	1046
novel.8409	NW_016592853.1	61	401	-	240
novel.8402	NW_016592627.1	359	1025	+	546
novel.8403	NW_016592637.1	1	508	+	295
novel.8400	NW_016591943.1	212	1000	+	514
novel.8401	NW_016592393.1	1	1021	-	711
novel.8406	NW_016592784.1	1	1043	-	1009
novel.8407	NW_016592786.1	479	984	+	506
novel.8404	NW_016592684.1	1	557	+	276
novel.8405	NW_016592691.1	1	814	-	814
novel.1350	NW_016530274.1	230701	283530	+	1153
novel.5869	NW_016547846.1	151561	153795	-	220
novel.5868	NW_016547846.1	169396	180067	+	2780
novel.5867	NW_016547846.1	159878	161352	+	461
novel.5866	NW_016547836.1	22159	22798	+	472
novel.5865	NW_016547834.1	11560	16110	+	3429
novel.5864	NW_016547809.1	237	14504	+	1311
novel.5863	NW_016547790.1	7615	8756	-	920
novel.5862	NW_016547790.1	7615	8746	+	920
novel.5861	NW_016547789.1	1369	2180	+	207
novel.5860	NW_016547764.1	217639	223854	+	328
novel.6251	NW_016550459.1	5203	5421	+	219
novel.6250	NW_016550450.1	11188	11701	-	367
novel.6253	NW_016550459.1	44804	46494	-	1636
novel.6252	NW_016550459.1	12583	19229	+	1025
novel.6255	NW_016550459.1	48988	51743	-	1684
novel.6254	NW_016550459.1	46573	46827	-	213
novel.6257	NW_016550472.1	3754	4504	+	241
novel.6256	NW_016550459.1	54893	55837	-	666
novel.6259	NW_016550497.1	8964	10477	+	1514
novel.6258	NW_016550472.1	28802	31190	+	1810
novel.6789	NW_016555462.1	3396	4235	+	793

novel.987	NW_016529531.1	416688	417558	+	674
novel.6783	NW_016555418.1	138143	141507	+	1427
novel.6782	NW_016555415.1	12095	12468	-	346
novel.6781	NW_016555386.1	41643	42027	-	362
novel.2222	NW_016532658.1	33243	33987	+	717
novel.6787	NW_016555448.1	2784	4176	-	1139
novel.6786	NW_016555429.1	5964	8367	-	802
novel.6785	NW_016555429.1	7140	9891	+	2326
novel.6784	NW_016555423.1	7620	7881	+	226
novel.2329	NW_016533059.1	35180	62735	+	336
novel.2328	NW_016533055.1	69839	70303	-	408
novel.5786	NW_016547155.1	68124	68431	+	252
novel.5787	NW_016547168.1	11836	35757	+	1926
novel.5784	NW_016547135.1	786111	787536	-	501
novel.5785	NW_016547147.1	11129	12512	+	280
novel.5782	NW_016547135.1	81278	81521	-	220
novel.5783	NW_016547135.1	184743	185698	-	956
novel.5780	NW_016547135.1	463731	463935	+	205
novel.5440	NW_016545086.1	286940	287833	+	712
novel.5443	NW_016545096.1	58975	59619	-	417
novel.5788	NW_016547168.1	297844	308602	+	655
novel.5789	NW_016547174.1	7074	7899	+	826
novel.5442	NW_016545096.1	58338	58737	-	400
novel.7328	NW_016562772.1	14561	16149	-	1532
novel.8188	NW_016585484.1	1085	2097	+	956
novel.7326	NW_016562737.1	27956	28741	+	760
novel.8186	NW_016585438.1	659	982	+	324
novel.7324	NW_016562729.1	4370	5209	+	811
novel.7325	NW_016562730.1	3449	3939	-	462
novel.7322	NW_016562707.1	15960	44526	-	444
novel.8182	NW_016585406.1	5443	5907	-	408
novel.7320	NW_016562689.1	2207	2512	+	206
novel.7321	NW_016562689.1	1	901	-	501
novel.1948	NW_016531871.1	13	3156	-	389
novel.1949	NW_016531885.1	323834	325894	+	2061
novel.1418	NW_016530419.1	1	3090	-	3055
novel.1419	NW_016530420.1	2220	27490	+	1726
novel.5447	NW_016545110.1	543141	543573	-	433
novel.1412	NW_016530409.1	2738	3091	+	354
novel.1413	NW_016530415.1	188633	189399	+	742
novel.1410	NW_016530398.1	8510	44914	+	1006
novel.1411	NW_016530398.1	48165	50427	+	597
novel.1416	NW_016530416.1	58267	58564	+	246
novel.1417	NW_016530417.1	162463	162860	+	219

novel.1414	NW_016530415.1	220729	221920	-	1164
novel.1415	NW_016530415.1	328532	370398	-	203
novel.5258	NW_016543951.1	1174	2981	-	1076
novel.5259	NW_016543951.1	5180	5913	-	625
novel.5250	NW_016543870.1	4701	22301	-	1424
novel.5251	NW_016543883.1	15504	16360	+	828
novel.5252	NW_016543883.1	8432	12871	-	314
novel.5253	NW_016543899.1	3963	13991	-	322
novel.5254	NW_016543918.1	26147	26493	-	307
novel.5255	NW_016543947.1	6613	6984	-	315
novel.5256	NW_016543951.1	308	624	+	265
novel.5257	NW_016543951.1	2587	4548	+	1911
novel.4067	NW_016538757.1	28889	66791	-	728
novel.348	NW_016528037.1	181790	182100	+	259
novel.349	NW_016528037.1	186834	187703	+	255
novel.618	NW_016528630.1	33163	33894	+	702
novel.619	NW_016528630.1	63051	63682	+	632
novel.342	NW_016528029.1	83528	85643	+	2023
novel.343	NW_016528030.1	345751	346134	+	328
novel.340	NW_016528023.1	19106	19567	+	462
novel.341	NW_016528023.1	17350	17870	-	521
novel.346	NW_016528034.1	461695	464306	-	675
novel.347	NW_016528036.1	5664	6643	-	270
novel.344	NW_016528034.1	25077	25700	+	624
novel.345	NW_016528034.1	133266	134432	-	1167
novel.8363	NW_016591329.1	460	888	+	429
novel.8362	NW_016591328.1	5952	7365	-	1414
novel.8361	NW_016591305.1	1530	4922	+	292
novel.8360	NW_016591276.1	4232	4502	-	271
novel.8367	NW_016591459.1	1792	4267	+	453
novel.8366	NW_016591457.1	1816	5574	+	276
novel.8365	NW_016591412.1	307	1177	-	839
novel.8364	NW_016591380.1	567	1095	+	529
novel.8369	NW_016591532.1	1	1828	+	1669
novel.8368	NW_016591472.1	2588	4306	-	744
novel.3509	NW_016536907.1	180326	180648	+	285
novel.7303	NW_016562388.1	8711	9433	-	661
novel.7304	NW_016562420.1	64296	65787	+	1437
novel.2800	NW_016534392.1	10157	10912	+	706
novel.2801	NW_016534399.1	117907	119087	+	375
novel.2802	NW_016534400.1	5	6486	+	225
novel.2803	NW_016534402.1	51413	53907	-	2462
novel.2804	NW_016534404.1	27460	45680	+	276
novel.2805	NW_016534408.1	677504	680366	-	2863

novel.2806	NW_016534411.1	59194	59520	+	219
novel.2807	NW_016534412.1	103028	103283	-	204
novel.2808	NW_016534417.1	98849	103007	+	276
novel.2809	NW_016534417.1	85810	87457	-	695
novel.4123	NW_016539012.1	130314	134010	-	1064
novel.4122	NW_016539005.1	174467	174698	+	203
novel.4125	NW_016539022.1	42752	43210	-	405
novel.4124	NW_016539018.1	435	10207	+	431
novel.4127	NW_016539053.1	2308	3353	-	994
novel.4126	NW_016539052.1	381245	385868	+	2956
novel.3313	NW_016536171.1	46116	46553	-	385
novel.3312	NW_016536171.1	22472	24300	-	787
novel.3311	NW_016536170.1	69048	69846	-	440
novel.3310	NW_016536170.1	43463	44140	-	343
novel.3317	NW_016536199.1	5104	6563	-	1360
novel.3316	NW_016536190.1	2034	2334	+	301
novel.3315	NW_016536187.1	210327	213431	-	3105
novel.3314	NW_016536180.1	2624	46404	-	521
novel.3319	NW_016536203.1	159816	160162	-	291
novel.3318	NW_016536203.1	306057	311797	+	250
novel.6608	NW_016553690.1	1	2833	-	2343
novel.6609	NW_016553691.1	36957	55945	+	1561
novel.7609	NW_016568237.1	2991	18325	-	2056
novel.7608	NW_016568232.1	42046	42525	-	281
novel.8118	NW_016583082.1	1	697	+	583
novel.8119	NW_016583122.1	1406	2183	-	730
novel.6600	NW_016553627.1	6282	15138	-	277
novel.2376	NW_016533193.1	43330	43751	+	255
novel.2377	NW_016533194.1	6253	6854	+	550
novel.5414	NW_016544944.1	4866	5271	+	214
novel.2375	NW_016533187.1	3249	4075	+	775
novel.2372	NW_016533173.1	90269	92846	-	425
novel.2373	NW_016533179.1	443961	463459	+	639
novel.2370	NW_016533169.1	101063	105810	-	1871
novel.2371	NW_016533173.1	252266	252574	+	253
novel.6606	NW_016553672.1	209721	210120	+	352
novel.2378	NW_016533195.1	3384	3694	+	286
novel.5419	NW_016544970.1	61191	64150	-	647
novel.6604	NW_016553654.1	3826	7241	-	2975
novel.6605	NW_016553666.1	5675	5965	+	268
novel.3120	NW_016535584.1	5244	6475	+	1232
novel.3579	NW_016537136.1	3101	6767	-	1145
novel.3121	NW_016535586.1	61141	63301	+	1978
novel.1987	NW_016531972.1	85518	87301	+	1784

novel.3578	NW_016537136.1	5458	6542	+	1085
novel.3122	NW_016535586.1	59540	61028	-	1014
novel.6057	NW_016549290.1	34994	37663	+	2461
novel.3123	NW_016535586.1	152508	153030	-	317
novel.6056	NW_016549290.1	26754	28733	+	1940
novel.3124	NW_016535592.1	3113	79557	-	896
novel.6055	NW_016549284.1	149952	150208	+	201
novel.3125	NW_016535599.1	125019	127073	+	2055
novel.3574	NW_016537133.1	220224	235465	+	204
novel.3126	NW_016535599.1	116045	120768	-	2118
novel.3573	NW_016537131.1	459107	459707	-	601
novel.3127	NW_016535600.1	3138	10200	-	423
novel.3572	NW_016537131.1	366075	367571	-	214
novel.3571	NW_016537131.1	147500	149765	-	249
novel.6050	NW_016549262.1	25267	28615	+	210
novel.1777	NW_016531412.1	9127	24838	-	283
novel.1776	NW_016531410.1	30153	31281	+	322
novel.1775	NW_016531404.1	17195	18142	-	948
novel.1774	NW_016531404.1	7330	8749	-	906
novel.1773	NW_016531404.1	16018	16978	+	930
novel.1772	NW_016531402.1	69708	75567	-	1107
novel.1771	NW_016531402.1	417597	435333	+	887
novel.1770	NW_016531402.1	16249	23710	+	2557
novel.3831	NW_016537885.1	14063	15325	-	751
novel.1779	NW_016531414.1	213048	213734	-	396
novel.1778	NW_016531412.1	213082	213626	-	352
novel.5067	NW_016542877.1	490968	492996	-	205
novel.5066	NW_016542877.1	666969	667343	+	320
novel.5065	NW_016542877.1	290299	291091	+	568
novel.58	NW_016527373.1	100046	100519	-	259
novel.5063	NW_016542856.1	10302	11348	-	943
novel.5062	NW_016542856.1	9671	10235	-	539
novel.5061	NW_016542856.1	8482	9033	-	507
novel.5060	NW_016542856.1	4719	8336	-	3458
novel.53	NW_016527363.1	155942	158360	-	948
novel.52	NW_016527363.1	143909	144277	-	250
novel.51	NW_016527363.1	29764	30270	-	332
novel.50	NW_016527363.1	23668	28071	+	451
novel.57	NW_016527373.1	96864	97768	-	466
novel.56	NW_016527373.1	96861	97200	+	293
novel.55	NW_016527369.1	115219	118332	+	383
novel.54	NW_016527363.1	161607	164861	-	2652
novel.1219	NW_016529977.1	329849	401062	-	794
novel.1749	NW_016531373.1	190810	192962	-	2153

novel.1746	NW_016531369.1	22895	27006	-	4112
novel.6448	NW_016552224.1	18831	20406	-	222
novel.6449	NW_016552224.1	69527	90269	-	241
novel.1747	NW_016531369.1	27248	30321	-	2876
novel.6444	NW_016552208.1	486	894	-	270
novel.6445	NW_016552212.1	579	1033	-	251
novel.6446	NW_016552219.1	23354	33900	-	2445
novel.6447	NW_016552224.1	177287	177556	+	270
novel.6440	NW_016552138.1	108454	109134	+	627
novel.6441	NW_016552138.1	108456	110368	-	732
novel.6442	NW_016552175.1	4933	6013	-	334
novel.6443	NW_016552190.1	3834	9444	-	1259
novel.1213	NW_016529955.1	239713	240411	+	296
novel.1742	NW_016531357.1	312915	314298	-	1384
novel.1215	NW_016529955.1	48766	53488	-	1173
novel.1740	NW_016531357.1	630974	631409	+	385
novel.1217	NW_016529955.1	263336	263915	-	400
novel.4334	NW_016539980.1	631923	639014	+	281
novel.4335	NW_016539980.1	664760	668198	-	2104
novel.4336	NW_016539980.1	670810	672325	-	820
novel.4337	NW_016539990.1	1	531	+	219
novel.4330	NW_016539973.1	421435	443166	+	283
novel.4331	NW_016539973.1	445116	446041	+	926
novel.4332	NW_016539979.1	24137	24732	+	391
novel.4333	NW_016539980.1	164380	178576	+	525
novel.3971	NW_016538392.1	61730	62209	+	440
novel.4338	NW_016539991.1	12803	19775	-	2199
novel.4339	NW_016539998.1	223113	223841	-	729
novel.5269	NW_016543988.1	9007	9550	+	283
novel.409	NW_016528146.1	25809	33123	+	343
novel.408	NW_016528144.1	524071	527205	-	918
novel.401	NW_016528118.1	98686	99781	-	949
novel.400	NW_016528118.1	98014	98615	-	557
novel.403	NW_016528124.1	22873	24133	+	1053
novel.402	NW_016528123.1	8216	9147	-	932
novel.405	NW_016528125.1	3159	9532	-	221
novel.404	NW_016528124.1	22873	23960	-	883
novel.407	NW_016528137.1	70284	70838	+	377
novel.406	NW_016528130.1	30813	31223	-	360
novel.5057	NW_016542842.1	106151	113555	-	292
novel.5268	NW_016543982.1	8921	9254	-	287
novel.2320	NW_016533037.1	133659	136338	-	2239
novel.5051	NW_016542809.1	10039	11008	-	887
novel.8798	NW_016607223.1	3027	6511	+	1943

novel.8799	NW_016607227.1	3731	6874	-	443
novel.8792	NW_016607125.1	1929	4398	+	372
novel.8793	NW_016607165.1	1	3172	-	1457
novel.8790	NW_016607068.1	4329	8179	-	3851
novel.8791	NW_016607119.1	1455	8222	+	402
novel.8796	NW_016607183.1	8	3656	+	3649
novel.8797	NW_016607188.1	2223	8822	+	743
novel.8794	NW_016607179.1	1609	1886	+	278
novel.8795	NW_016607179.1	2093	3817	+	1418
novel.3818	NW_016537849.1	814185	816739	+	832
novel.3819	NW_016537849.1	760503	774793	+	378
novel.6868	NW_016556333.1	43649	43956	-	252
novel.6869	NW_016556334.1	7953	39026	-	518
novel.3810	NW_016537830.1	22077	79320	-	296
novel.3811	NW_016537832.1	112981	117132	+	516
novel.3812	NW_016537832.1	338342	375779	+	1311
novel.3813	NW_016537832.1	345652	348235	+	266
novel.3814	NW_016537832.1	348286	348915	+	573
novel.3815	NW_016537832.1	294987	301440	-	5275
novel.3816	NW_016537832.1	348278	348800	-	466
novel.3817	NW_016537843.1	343753	344428	+	508
novel.2149	NW_016532417.1	114175	116212	-	777
novel.2148	NW_016532417.1	23517	24118	-	567
novel.3385	NW_016536411.1	18	2684	+	2667
novel.2145	NW_016532409.1	58955	59419	-	224
novel.2144	NW_016532409.1	58385	58885	-	501
novel.2147	NW_016532416.1	3	841	+	839
novel.2146	NW_016532409.1	167524	168297	-	749
novel.2141	NW_016532398.1	30514	35936	-	516
novel.2140	NW_016532398.1	5253	5692	-	383
novel.2143	NW_016532409.1	37786	42235	+	562
novel.2142	NW_016532398.1	51491	52229	-	431
novel.8428	NW_016594427.1	1	1098	-	662
novel.8429	NW_016594554.1	522	1150	-	285
novel.8424	NW_016594168.1	1	902	-	902
novel.8425	NW_016594169.1	211	1124	-	914
novel.8426	NW_016594288.1	1	1050	+	546
novel.8427	NW_016594382.1	1	1139	+	1139
novel.8420	NW_016593994.1	17	1114	-	400
novel.8421	NW_016594006.1	471	1114	-	644
novel.8422	NW_016594090.1	1	1119	+	237
novel.8423	NW_016594098.1	1	1120	-	873
novel.6993	NW_016557685.1	8831	9071	-	241
novel.5881	NW_016547966.1	52439	54453	+	2015

novel.5880	NW_016547965.1	7610	17786	+	278
novel.5883	NW_016547979.1	775	1345	-	571
novel.5882	NW_016547979.1	1	1348	+	1326
novel.5885	NW_016547995.1	8146	13109	-	2342
novel.5884	NW_016547995.1	63684	65037	+	804
novel.5887	NW_016548000.1	262645	265597	-	2653
novel.5886	NW_016548000.1	262645	265216	+	2450
novel.5889	NW_016548002.1	4545	5906	-	496
novel.5888	NW_016548001.1	20094	20941	+	294
novel.3389	NW_016536414.1	14170	19174	-	3850
novel.2326	NW_016533055.1	20773	26810	+	225
novel.3768	NW_016537722.1	1270	1646	-	320
novel.3769	NW_016537730.1	3676	7356	+	2166
novel.3762	NW_016537703.1	3037	3247	+	211
novel.3763	NW_016537703.1	6932	7232	-	245
novel.3760	NW_016537690.1	18964	19322	+	306
novel.3761	NW_016537695.1	4709	4967	-	213
novel.3766	NW_016537721.1	245433	245867	+	379
novel.3767	NW_016537721.1	245433	246012	-	580
novel.3764	NW_016537715.1	4550	4886	+	287
novel.3765	NW_016537715.1	4058	4394	-	337
novel.4932	NW_016542320.1	83412	117283	-	242
novel.4933	NW_016542322.1	149547	156503	+	2142
novel.4930	NW_016542308.1	4168	4729	-	562
novel.4931	NW_016542309.1	61615	123074	+	1533
novel.4936	NW_016542328.1	58974	97735	-	926
novel.4937	NW_016542333.1	5485	6786	+	1250
novel.4934	NW_016542328.1	56630	57421	+	792
novel.4935	NW_016542328.1	57513	58384	+	802
novel.4938	NW_016542355.1	68050	69063	+	828
novel.4939	NW_016542356.1	25620	34344	+	610
novel.8542	NW_016600323.1	1	1812	+	867
novel.4684	NW_016541300.1	160724	169512	+	2079
novel.4685	NW_016541305.1	6640	7000	+	307
novel.4686	NW_016541307.1	612	1022	-	221
novel.4687	NW_016541309.1	3515	20960	+	2169
novel.4680	NW_016541291.1	250399	250749	+	351
novel.4681	NW_016541291.1	250399	250749	-	351
novel.4682	NW_016541291.1	256189	319092	-	294
novel.4683	NW_016541299.1	22467	23985	-	636
novel.6273	NW_016550600.1	1910	2659	-	216
novel.6272	NW_016550600.1	311625	316934	+	4325
novel.6271	NW_016550599.1	409	954	+	311
novel.6270	NW_016550597.1	11824	12414	-	568

novel.4688	NW_016541311.1	36879	38161	-	1233
novel.4689	NW_016541314.1	6201	11388	+	1759
novel.6275	NW_016550600.1	12069	12842	-	774
novel.6274	NW_016550600.1	10104	11372	-	1269
novel.7142	NW_016559604.1	31668	33537	+	357
novel.7143	NW_016559623.1	77364	79567	+	1971
novel.7908	NW_016575758.1	5720	7670	-	229
novel.7909	NW_016575766.1	1	344	+	304
novel.188	NW_016527644.1	42110	42415	+	250
novel.189	NW_016527650.1	590	864	+	275
novel.184	NW_016527632.1	1	2718	+	2666
novel.185	NW_016527632.1	1	2718	-	2644
novel.186	NW_016527637.1	409185	416341	-	1565
novel.187	NW_016527640.1	121024	124849	-	689
novel.180	NW_016527622.1	25195	25608	+	414
novel.181	NW_016527624.1	298855	299179	+	325
novel.182	NW_016527629.1	150416	153890	+	1542
novel.183	NW_016527629.1	71667	74496	-	2773
novel.7652	NW_016568999.1	13016	13548	+	491
novel.7653	NW_016568999.1	13102	13548	-	447
novel.7302	NW_016562369.1	3864	4075	+	212
novel.8162	NW_016584494.1	1098	2541	-	398
novel.8165	NW_016584539.1	3408	6296	+	2283
novel.7305	NW_016562433.1	44098	45899	-	298
novel.8167	NW_016584712.1	31	1174	+	697
novel.8166	NW_016584618.1	1	5146	+	324
novel.7308	NW_016562473.1	1203	4018	-	2724
novel.7309	NW_016562484.1	5783	6389	-	575
novel.7658	NW_016569002.1	142198	142703	-	320
novel.7659	NW_016569061.1	1	1173	-	1173
novel.1438	NW_016530488.1	287765	288056	-	242
novel.1439	NW_016530500.1	1624	2355	+	712
novel.1434	NW_016530471.1	250254	258012	-	3686
novel.1435	NW_016530483.1	168637	170211	+	1472
novel.1436	NW_016530483.1	168449	169521	-	1034
novel.1437	NW_016530488.1	394003	394376	+	326
novel.1430	NW_016530454.1	42159	44189	-	714
novel.1431	NW_016530466.1	45186	46863	+	1570
novel.1432	NW_016530466.1	41867	44630	-	2718
novel.1433	NW_016530470.1	43358	44853	-	751
novel.6285	NW_016550679.1	1997	2474	-	434
novel.7642	NW_016568897.1	11322	13392	+	2071
novel.360	NW_016528052.1	332687	333252	-	329
novel.361	NW_016528053.1	29929	32413	+	2212

novel.362	NW_016528054.1	84473	105475	+	1115
novel.363	NW_016528054.1	234659	239209	+	633
novel.364	NW_016528054.1	158663	216766	-	916
novel.365	NW_016528054.1	344348	344665	-	318
novel.366	NW_016528058.1	233827	234684	+	206
novel.367	NW_016528058.1	356654	359338	-	1158
novel.368	NW_016528059.1	4827	5133	+	307
novel.369	NW_016528060.1	23867	24750	+	229
novel.8305	NW_016589288.1	748	1030	-	250
novel.8304	NW_016589252.1	984	1320	-	292
novel.8307	NW_016589404.1	1	1089	-	992
novel.8306	NW_016589374.1	1	916	-	916
novel.8301	NW_016589099.1	2836	5743	+	353
novel.8300	NW_016589064.1	1	1746	-	1718
novel.8303	NW_016589235.1	467	3144	+	375
novel.8302	NW_016589113.1	106	570	+	445
novel.8309	NW_016589475.1	32460	32727	+	268
novel.8308	NW_016589462.1	3896	5439	+	444
novel.5508	NW_016545496.1	13017	18233	-	2695
novel.5509	NW_016545496.1	69385	85406	-	951
novel.2651	NW_016533984.1	23025	24325	-	1301
novel.2650	NW_016533982.1	16064	17202	-	563
novel.2653	NW_016533987.1	96450	100222	-	3773
novel.2224	NW_016532659.1	32632	33349	+	672
novel.2652	NW_016533987.1	5067	6604	+	1202
novel.2225	NW_016532659.1	1514	2005	-	444
novel.2655	NW_016533990.1	14986	15266	-	281
novel.5506	NW_016545496.1	85465	96896	+	817
novel.2654	NW_016533988.1	163743	164541	+	760
novel.2227	NW_016532667.1	18235	19151	-	917
novel.2657	NW_016533999.1	489772	495402	+	402
novel.2220	NW_016532642.1	635273	645435	-	742
novel.2656	NW_016533993.1	42872	43499	+	596
novel.2221	NW_016532652.1	201818	202867	-	1023
novel.3151	NW_016535646.1	129245	131361	+	1960
novel.3150	NW_016535639.1	6943	9285	+	264
novel.3153	NW_016535648.1	102407	102705	+	299
novel.3152	NW_016535646.1	499	752	-	254
novel.3155	NW_016535650.1	28467	31165	-	534
novel.3154	NW_016535649.1	47268	50377	-	2802
novel.3157	NW_016535652.1	83772	84343	+	376
novel.3156	NW_016535650.1	40791	42608	-	1818
novel.3159	NW_016535656.1	216819	221297	-	738
novel.3158	NW_016535653.1	3807	4084	+	278

novel.5503	NW_016545473.1	4521	7143	+	535
novel.3702	NW_016537535.1	63681	65115	+	1435
novel.3703	NW_016537535.1	187795	188787	+	993
novel.3704	NW_016537535.1	189142	192188	+	2993
novel.3257	NW_016535960.1	112007	112673	+	610
novel.4103	NW_016538921.1	79292	80030	+	489
novel.4102	NW_016538916.1	7598	19977	+	566
novel.4101	NW_016538911.1	18880	20696	+	1785
novel.4100	NW_016538911.1	12308	12948	+	641
novel.4107	NW_016538926.1	14870	15974	-	1070
novel.3254	NW_016535959.1	4868	6000	+	1079
novel.4105	NW_016538926.1	872	2257	+	392
novel.4104	NW_016538921.1	85541	86067	-	494
novel.4109	NW_016538932.1	133058	135914	+	1465
novel.4108	NW_016538930.1	93870	99038	-	3782
novel.3489	NW_016536810.1	235681	240353	-	444
novel.3488	NW_016536810.1	189191	190818	-	1327
novel.3483	NW_016536769.1	11386	26661	-	251
novel.3482	NW_016536768.1	423616	426093	-	2425
novel.3481	NW_016536768.1	22544	22819	+	276
novel.3480	NW_016536765.1	2983	3238	+	256
novel.3487	NW_016536803.1	75252	75607	+	299
novel.3486	NW_016536797.1	9726	10062	-	285
novel.3485	NW_016536789.1	76083	76558	+	323
novel.3484	NW_016536785.1	526674	527067	-	340
novel.3375	NW_016536397.1	102529	108113	+	2324
novel.3374	NW_016536397.1	99171	100462	+	872
novel.3377	NW_016536400.1	6972	259535	+	495
novel.3376	NW_016536397.1	111982	112898	+	871
novel.3371	NW_016536393.1	25293	26012	-	673
novel.3370	NW_016536393.1	61998	62314	+	262
novel.3373	NW_016536397.1	93764	94603	+	751
novel.3372	NW_016536396.1	16953	17279	-	277
novel.3379	NW_016536403.1	5026	5626	-	571
novel.3378	NW_016536403.1	3881	4304	+	325
novel.7344	NW_016563084.1	1	1900	+	896
novel.7345	NW_016563102.1	2778	3205	-	428
novel.7346	NW_016563109.1	1785	2324	-	513
novel.7347	NW_016563166.1	66843	68668	+	1097
novel.4168	NW_016539231.1	16513	22649	+	6137
novel.7341	NW_016563064.1	991	1298	+	282
novel.7342	NW_016563064.1	1666	1957	+	235
novel.7343	NW_016563078.1	1093	1563	-	471
novel.2686	NW_016534081.1	41811	66045	-	591

novel.2687	NW_016534091.1	163389	164663	+	1228
novel.2684	NW_016534079.1	194353	195664	+	744
novel.2685	NW_016534079.1	377247	381695	-	1572
novel.2682	NW_016534067.1	31215	35724	-	223
novel.2683	NW_016534075.1	139892	140565	+	674
novel.2680	NW_016534064.1	19381	27684	-	858
novel.2681	NW_016534064.1	103275	105674	-	1286
novel.2688	NW_016534096.1	46026	48176	-	889
novel.2689	NW_016534099.1	192991	206675	+	483
novel.2358	NW_016533148.1	249371	252987	+	291
novel.2359	NW_016533148.1	190708	191990	-	1228
novel.2428	NW_016533340.1	33166	34107	-	661
novel.2429	NW_016533342.1	18130	18934	+	805
novel.2422	NW_016533330.1	30398	31816	-	1375
novel.2351	NW_016533134.1	5261	5921	+	247
novel.2352	NW_016533134.1	101697	101981	+	230
novel.2421	NW_016533319.1	771833	775589	-	3757
novel.2426	NW_016533337.1	371875	380287	+	684
novel.2427	NW_016533340.1	30237	32560	+	626
novel.2424	NW_016533336.1	14431	15038	+	608
novel.2357	NW_016533147.1	29308	38140	-	550
novel.968	NW_016529462.1	280212	280961	+	262
novel.969	NW_016529462.1	8768	9131	-	315
novel.962	NW_016529455.1	25945	26280	+	284
novel.963	NW_016529458.1	469390	470173	+	527
novel.960	NW_016529438.1	87311	87917	+	607
novel.961	NW_016529447.1	82568	82832	-	232
novel.966	NW_016529461.1	183315	183727	+	413
novel.967	NW_016529462.1	219910	220120	+	211
novel.964	NW_016529460.1	561338	561892	-	505
novel.965	NW_016529461.1	168045	169820	+	913
novel.1086	NW_016529713.1	9125	10033	-	909
novel.2388	NW_016533220.1	319380	322529	-	3102
novel.7740	NW_016570739.1	3788	4129	+	321
novel.7741	NW_016570748.1	4462	48205	-	654
novel.1089	NW_016529719.1	221263	222967	+	1003
novel.7742	NW_016570813.1	1	743	-	711
novel.1088	NW_016529719.1	214447	217317	+	2871
novel.7743	NW_016570980.1	8	5495	+	320
novel.7744	NW_016571062.1	5776	10310	+	1644
novel.7745	NW_016571081.1	2532	3643	-	668
novel.7746	NW_016571127.1	6812	9128	+	443
novel.7747	NW_016571129.1	4797	6232	+	1409
novel.71	NW_016527385.1	250838	251509	-	325

novel.70	NW_016527385.1	383724	384173	+	286
novel.73	NW_016527398.1	234956	238665	-	3710
novel.72	NW_016527388.1	9989	12816	-	381
novel.75	NW_016527406.1	161936	162332	+	397
novel.74	NW_016527402.1	25796	26134	-	283
novel.77	NW_016527407.1	357009	362032	-	4506
novel.76	NW_016527407.1	22532	25381	+	1271
novel.79	NW_016527415.1	63189	368871	+	659
novel.78	NW_016527415.1	28676	29541	+	866
novel.5904	NW_016548131.1	138	412	-	251
novel.4749	NW_016541510.1	132592	135463	+	950
novel.4748	NW_016541497.1	246930	248635	-	712
novel.4741	NW_016541458.1	14397	14732	-	336
novel.4740	NW_016541456.1	123426	146211	+	804
novel.4743	NW_016541469.1	2932	4611	-	1637
novel.4742	NW_016541469.1	1	4611	+	2264
novel.4745	NW_016541491.1	138126	139487	-	1257
novel.4744	NW_016541481.1	18837	19116	-	258
novel.4747	NW_016541497.1	269004	270922	+	300
novel.4746	NW_016541493.1	310933	311402	+	279
novel.918	NW_016529388.1	15025	132087	+	3277
novel.2470	NW_016533467.1	27574	44164	-	237
novel.6831	NW_016555916.1	23237	25134	+	1898
novel.7529	NW_016566718.1	33451	40647	+	755
novel.6833	NW_016555922.1	5083	6613	-	418
novel.6832	NW_016555920.1	2224	2533	+	265
novel.6835	NW_016555951.1	13019	16246	+	346
novel.6834	NW_016555927.1	23058	24896	+	207
novel.3661	NW_016537401.1	156883	158991	+	279
novel.2479	NW_016533499.1	1	2727	+	2671
novel.3660	NW_016537382.1	96169	98202	-	973
novel.2478	NW_016533495.1	247400	247824	-	226
novel.6426	NW_016551974.1	10725	11386	-	282
novel.6427	NW_016552018.1	5855	6269	-	210
novel.6424	NW_016551968.1	27206	29623	+	1830
novel.6425	NW_016551968.1	37572	46978	+	3595
novel.6422	NW_016551920.1	88886	93677	+	203
novel.6423	NW_016551931.1	122822	124807	-	1938
novel.6420	NW_016551895.1	1468	13334	-	3562
novel.6421	NW_016551905.1	16683	17198	+	307
novel.8615	NW_016602978.1	81	2495	-	535
novel.6428	NW_016552058.1	4158	4969	+	784
novel.6429	NW_016552072.1	1814	6103	+	321
novel.799	NW_016529109.1	12359	192911	+	3742

novel.798	NW_016529108.1	385116	390018	+	817
novel.8614	NW_016602859.1	305	2493	+	2189
novel.3935	NW_016538313.1	105316	108769	+	1014
novel.791	NW_016529091.1	230508	231067	+	507
novel.790	NW_016529089.1	352935	354165	+	1231
novel.793	NW_016529102.1	345245	352942	+	274
novel.792	NW_016529093.1	78183	79447	+	225
novel.795	NW_016529103.1	18797	21424	-	433
novel.794	NW_016529103.1	191116	191419	+	210
novel.797	NW_016529105.1	237835	254556	-	2206
novel.796	NW_016529103.1	147244	148062	-	702
novel.8611	NW_016602792.1	587	2456	-	1367
novel.4358	NW_016540068.1	420029	425135	+	375
novel.4359	NW_016540071.1	19032	19325	-	294
novel.4356	NW_016540049.1	186524	187002	+	320
novel.4357	NW_016540056.1	76591	88707	-	564
novel.4354	NW_016540047.1	5560	6704	+	1041
novel.8346	NW_016590816.1	194	539	-	297
novel.4352	NW_016540039.1	80687	81015	+	280
novel.4353	NW_016540044.1	79954	81980	+	655
novel.4350	NW_016540038.1	3945	68077	-	341
novel.4351	NW_016540039.1	25110	138421	+	8815
novel.3934	NW_016538313.1	102341	103246	+	256
novel.429	NW_016528184.1	69182	162567	+	423
novel.428	NW_016528183.1	108159	109193	+	1003
novel.427	NW_016528180.1	87623	88584	-	401
novel.426	NW_016528180.1	90475	94214	+	754
novel.425	NW_016528174.1	544150	558157	+	293
novel.424	NW_016528162.1	7461	19494	-	4115
novel.423	NW_016528162.1	6400	6776	-	227
novel.422	NW_016528162.1	12321	13310	+	783
novel.421	NW_016528160.1	3528	4192	+	535
novel.420	NW_016528156.1	600971	602364	-	478
novel.1337	NW_016530240.1	98643	101899	+	1392
novel.1336	NW_016530239.1	23590	213042	+	346
novel.1335	NW_016530236.1	10836	39456	-	1237
novel.1621	NW_016530979.1	333132	334303	+	1122
novel.1334	NW_016530235.1	69927	70371	-	416
novel.1881	NW_016531672.1	49740	55434	+	1169
novel.4093	NW_016538874.1	14896	20622	+	835
novel.1332	NW_016530202.1	20515	21345	-	831
novel.7469	NW_016565613.1	18394	19668	-	1218
novel.7468	NW_016565612.1	3200	8889	-	696
novel.4092	NW_016538868.1	23802	32176	-	474

novel.1331	NW_016530202.1	38798	40009	+	421
novel.7461	NW_016565388.1	65113	75733	-	2291
novel.7460	NW_016565374.1	1	1159	+	1105
novel.7463	NW_016565474.1	55001	61082	+	667
novel.1330	NW_016530201.1	79454	79993	+	333
novel.7465	NW_016565542.1	64	872	+	809
novel.7464	NW_016565508.1	1396	2031	+	476
novel.7467	NW_016565600.1	49048	51277	-	823
novel.7466	NW_016565584.1	12994	13682	+	274
novel.6840	NW_016555954.1	51153	52225	-	1073
novel.6841	NW_016555959.1	1	865	+	833
novel.6842	NW_016555977.1	567	854	+	231
novel.6843	NW_016555978.1	515	910	+	368
novel.6844	NW_016556009.1	14600	17012	+	379
novel.6845	NW_016556033.1	15910	20635	+	200
novel.6846	NW_016556066.1	1	335	+	335
novel.6847	NW_016556066.1	501	760	+	220
novel.6848	NW_016556069.1	8627	19105	-	562
novel.6849	NW_016556114.1	4737	5026	+	234
novel.1623	NW_016530993.1	621604	622050	+	394
novel.2167	NW_016532496.1	141539	142064	-	481
novel.2166	NW_016532496.1	51794	53100	-	913
novel.2165	NW_016532496.1	29981	30299	-	262
novel.2164	NW_016532496.1	148382	151073	+	788
novel.2163	NW_016532496.1	140470	142077	+	1530
novel.2162	NW_016532488.1	15792	20255	-	403
novel.2161	NW_016532480.1	72118	72648	-	200
novel.2160	NW_016532480.1	72218	73194	+	660
novel.2169	NW_016532502.1	41453	42956	-	1504
novel.2168	NW_016532496.1	160993	162490	-	1403
novel.8446	NW_016595679.1	1	455	+	455
novel.8447	NW_016595718.1	1	1151	-	1151
novel.8444	NW_016595517.1	1	1198	-	755
novel.8445	NW_016595661.1	1	1231	+	1231
novel.8442	NW_016595416.1	184	1213	-	1030
novel.8443	NW_016595497.1	1	817	+	269
novel.8440	NW_016595327.1	1	976	+	397
novel.8441	NW_016595382.1	345	761	-	417
novel.8448	NW_016595735.1	1	502	-	386
novel.8449	NW_016595737.1	583	833	+	251
novel.1625	NW_016530993.1	621604	622517	-	775
novel.889	NW_016529295.1	93710	94122	+	379
novel.888	NW_016529295.1	56758	106302	+	282
novel.885	NW_016529290.1	163146	165064	+	1497

novel.884	NW_016529288.1	109150	110730	-	1492
novel.887	NW_016529290.1	197658	199067	+	1410
novel.886	NW_016529290.1	189691	190456	+	346
novel.881	NW_016529276.1	262814	264474	+	1661
novel.880	NW_016529275.1	10545	11766	+	297
novel.883	NW_016529288.1	108944	112078	+	840
novel.882	NW_016529278.1	324667	327433	-	2767
novel.1624	NW_016530993.1	624023	624373	+	294
novel.3748	NW_016537672.1	327593	327898	+	306
novel.3749	NW_016537672.1	276048	283420	-	204
novel.3744	NW_016537661.1	14261	14572	-	268
novel.3745	NW_016537664.1	11622	12538	+	866
novel.3746	NW_016537664.1	14791	16296	-	1506
novel.3747	NW_016537672.1	310547	311120	+	574
novel.3740	NW_016537651.1	161414	290231	+	4855
novel.3741	NW_016537657.1	44758	46201	+	1424
novel.3742	NW_016537657.1	44758	65956	-	1324
novel.3743	NW_016537660.1	10943	12441	+	555
novel.778	NW_016529041.1	9322	9737	+	416
novel.4914	NW_016542256.1	2067	2498	-	432
novel.4915	NW_016542264.1	18133	18658	+	481
novel.4916	NW_016542264.1	18806	19183	-	342
novel.4917	NW_016542272.1	9202	9927	-	706
novel.4910	NW_016542215.1	41602	43066	-	889
novel.4911	NW_016542222.1	50646	54547	+	3802
novel.4912	NW_016542222.1	63223	63762	-	422
novel.4913	NW_016542248.1	103200	104793	-	1504
novel.4918	NW_016542273.1	25749	26807	-	1010
novel.4919	NW_016542276.1	28862	31590	+	2634
novel.7470	NW_016565622.1	4165	4857	-	665
novel.6219	NW_016550251.1	228324	229500	+	1078
novel.6218	NW_016550251.1	219118	225859	+	504
novel.6215	NW_016550243.1	21523	22397	-	486
novel.6214	NW_016550241.1	94020	96528	-	722
novel.6217	NW_016550251.1	183369	186184	+	1186
novel.6216	NW_016550248.1	1	433	-	400
novel.6211	NW_016550241.1	91248	91888	+	490
novel.6210	NW_016550241.1	38144	38522	+	336
novel.6213	NW_016550241.1	38244	40384	-	2090
novel.6212	NW_016550241.1	45394	64811	-	1407
novel.1632	NW_016531014.1	8163	17354	+	1013
novel.1633	NW_016531014.1	33	337	-	273
novel.1630	NW_016531014.1	1157	2206	+	993
novel.1631	NW_016531014.1	2625	3341	+	717

novel.1188	NW_016529909.1	318203	319331	-	773
novel.1189	NW_016529913.1	28407	80409	-	243
novel.1634	NW_016531014.1	929	1352	-	398
novel.1635	NW_016531014.1	11004	12085	-	442
novel.1184	NW_016529908.1	2747	3163	-	306
novel.1185	NW_016529909.1	75573	81647	+	647
novel.1186	NW_016529909.1	156287	163295	+	306
novel.1187	NW_016529909.1	161319	167010	-	392
novel.1180	NW_016529902.1	65844	82218	+	523
novel.1181	NW_016529902.1	42885	66273	-	1124
novel.1182	NW_016529904.1	1187	1986	-	771
novel.1183	NW_016529908.1	886	1427	+	487
novel.5742	NW_016546974.1	5654	6057	+	375
novel.5743	NW_016546976.1	27970	30350	-	1258
novel.5740	NW_016546948.1	375638	376912	+	1211
novel.5741	NW_016546969.1	322336	323964	-	1021
novel.5746	NW_016546994.1	10536	11547	+	906
novel.5747	NW_016546994.1	11929	12569	+	589
novel.5744	NW_016546984.1	91349	91599	-	251
novel.5745	NW_016546989.1	1622	2314	+	665
novel.5748	NW_016546994.1	9724	10377	-	597
novel.5749	NW_016546994.1	10536	11459	-	878
novel.162	NW_016527596.1	203581	283576	+	312
novel.163	NW_016527596.1	177196	181994	-	1733
novel.160	NW_016527593.1	178	416	+	239
novel.161	NW_016527593.1	734	972	+	239
novel.166	NW_016527599.1	82758	85411	+	2631
novel.167	NW_016527599.1	347298	350975	+	3678
novel.164	NW_016527599.1	63218	66276	+	1897
novel.165	NW_016527599.1	66747	93294	+	2083
novel.7924	NW_016575997.1	3891	4282	+	362
novel.7925	NW_016576039.1	322	896	-	519
novel.168	NW_016527599.1	81630	82602	-	917
novel.7927	NW_016576097.1	385	678	+	245
novel.7920	NW_016575983.1	27979	29084	+	1076
novel.7921	NW_016575983.1	22179	25226	-	2991
novel.7922	NW_016575983.1	28009	29084	-	1029
novel.7923	NW_016575989.1	3787	15352	+	393
novel.7674	NW_016569353.1	876	1349	-	389
novel.7675	NW_016569355.1	6907	9145	+	2239
novel.7676	NW_016569355.1	749	2102	-	1331
novel.7677	NW_016569425.1	6619	13860	+	3306
novel.7670	NW_016569292.1	4323	10132	-	825
novel.7671	NW_016569323.1	215399	227721	-	326

novel.7672	NW_016569336.1	10680	11345	-	580
novel.7673	NW_016569336.1	175907	179442	-	3536
novel.7678	NW_016569458.1	16722	21812	-	4461
novel.7679	NW_016569493.1	4796	5404	+	552
novel.1984	NW_016531960.1	58872	62401	-	920
novel.1985	NW_016531961.1	84147	84581	+	392
novel.1986	NW_016531961.1	197339	197745	-	407
novel.1455	NW_016530547.1	197798	198157	+	216
novel.1980	NW_016531955.1	931	1843	-	871
novel.1453	NW_016530533.1	56284	61824	-	1860
novel.1450	NW_016530529.1	1025616	1026227	-	494
novel.1451	NW_016530533.1	58913	61073	+	2161
novel.1988	NW_016531972.1	234565	235653	+	544
novel.1989	NW_016531972.1	191269	203946	-	1864
novel.1458	NW_016530552.1	38136	40520	-	1410
novel.1459	NW_016530552.1	96859	98193	-	320
novel.5294	NW_016544151.1	64019	75704	+	5446
novel.5295	NW_016544174.1	23026	23960	+	366
novel.5296	NW_016544175.1	335898	336379	+	482
novel.5297	NW_016544177.1	8921	9200	+	240
novel.5290	NW_016544110.1	357	1637	-	1281
novel.5291	NW_016544110.1	884	1620	-	716
novel.5292	NW_016544113.1	224274	224848	+	520
novel.5293	NW_016544137.1	4457	6034	-	424
novel.5298	NW_016544182.1	40738	58678	+	3922
novel.5299	NW_016544182.1	48392	55832	-	4905
novel.306	NW_016527952.1	5486	22036	+	1136
novel.307	NW_016527953.1	384098	384635	+	454
novel.304	NW_016527948.1	778436	778808	+	293
novel.305	NW_016527952.1	1036	1347	+	265
novel.302	NW_016527946.1	253630	255473	-	1634
novel.303	NW_016527946.1	701286	703997	-	1318
novel.300	NW_016527946.1	661010	664529	+	1355
novel.301	NW_016527946.1	711993	713799	+	547
novel.308	NW_016527953.1	443327	447612	-	963
novel.309	NW_016527954.1	270364	273967	+	299
novel.7124	NW_016559258.1	466	1034	+	547
novel.2342	NW_016533108.1	317337	321886	-	4550
novel.8149	NW_016584263.1	1	5426	+	403
novel.8148	NW_016584213.1	55	519	-	254
novel.7125	NW_016559290.1	195134	195465	-	304
novel.8143	NW_016583934.1	1093	1485	-	337
novel.8142	NW_016583919.1	3951	4838	-	888
novel.8141	NW_016583763.1	54891	55582	-	576

novel.8140	NW_016583761.1	2731	3000	-	217
novel.8147	NW_016584199.1	2540	3045	-	466
novel.8146	NW_016584199.1	2449	7845	+	1169
novel.8145	NW_016584055.1	121	1380	-	324
novel.8144	NW_016584043.1	2601	4389	+	1737
novel.3179	NW_016535730.1	162946	163862	+	893
novel.3178	NW_016535730.1	94014	95822	+	1809
novel.3173	NW_016535695.1	121159	124843	+	406
novel.3172	NW_016535689.1	4325	17080	-	289
novel.3171	NW_016535689.1	2797	3064	-	224
novel.3170	NW_016535687.1	88591	90575	-	426
novel.3177	NW_016535727.1	60015	61064	+	424
novel.3176	NW_016535715.1	177503	178029	+	386
novel.3175	NW_016535703.1	224148	235176	-	431
novel.3174	NW_016535697.1	74874	75792	+	919
novel.6985	NW_016557629.1	5597	25332	-	3056
novel.6984	NW_016557617.1	105722	106973	-	1015
novel.6987	NW_016557652.1	1939	12994	+	1355
novel.6986	NW_016557637.1	3908	4391	-	275
novel.6981	NW_016557499.1	1	965	-	965
novel.6980	NW_016557489.1	8376	8594	+	219
novel.6983	NW_016557577.1	1282	5819	-	271
novel.6982	NW_016557510.1	11910	12201	-	235
novel.6989	NW_016557666.1	17300	18344	+	991
novel.6988	NW_016557652.1	21327	23949	-	1193
novel.4165	NW_016539221.1	240658	255689	-	200
novel.4164	NW_016539216.1	59032	59579	-	220
novel.4167	NW_016539229.1	52666	56501	+	215
novel.4166	NW_016539226.1	96809	105073	-	285
novel.4161	NW_016539206.1	3794	4185	-	339
novel.4160	NW_016539203.1	3509	4859	+	227
novel.4163	NW_016539216.1	40775	51207	+	580
novel.8328	NW_016590173.1	862	1557	-	334
novel.7586	NW_016567711.1	3881	13805	-	290
novel.7587	NW_016567768.1	61082	61538	-	410
novel.7584	NW_016567690.1	1580	2460	+	385
novel.7585	NW_016567690.1	113	1365	-	490
novel.8323	NW_016590068.1	105	757	-	552
novel.8322	NW_016590023.1	1	2071	-	780
novel.7580	NW_016567646.1	517	1043	+	502
novel.7581	NW_016567646.1	816	1473	-	400
novel.6189	NW_016550105.1	36266	39087	+	2592
novel.6188	NW_016550087.1	4184	5292	+	1053
novel.3359	NW_016536343.1	2673	4472	+	277

novel.3358	NW_016536341.1	89071	89331	+	224
novel.3357	NW_016536334.1	22128	22670	+	277
novel.3356	NW_016536322.1	100268	101030	-	659
novel.3355	NW_016536319.1	68503	69006	-	468
novel.3354	NW_016536319.1	137493	137788	+	296
novel.3353	NW_016536319.1	68503	69333	+	630
novel.3352	NW_016536312.1	32166	32370	+	205
novel.3351	NW_016536311.1	12846	14791	+	1812
novel.3350	NW_016536309.1	427656	428805	-	247
novel.3449	NW_016536661.1	76493	82069	+	2600
novel.3448	NW_016536661.1	65741	68149	+	1314
novel.5988	NW_016548786.1	35236	35578	-	293
novel.6127	NW_016549638.1	130134	132461	-	2328
novel.6126	NW_016549638.1	29486	30603	-	1061
novel.5980	NW_016548741.1	42055	47685	-	231
novel.5981	NW_016548747.1	59956	60478	+	473
novel.5982	NW_016548761.1	54116	59853	-	569
novel.5983	NW_016548764.1	55787	56737	+	951
novel.5984	NW_016548764.1	31341	55365	-	2357
novel.6125	NW_016549638.1	35707	36326	+	620
novel.5986	NW_016548768.1	13	2220	+	2159
novel.5987	NW_016548768.1	1	5431	-	401
novel.6124	NW_016549626.1	33592	34385	-	410
novel.6123	NW_016549622.1	45607	118749	+	1147
novel.6122	NW_016549619.1	263922	264357	-	266
novel.6121	NW_016549619.1	147025	147985	-	795
novel.6120	NW_016549619.1	146615	146905	-	217
novel.2408	NW_016533272.1	139723	140110	+	388
novel.2409	NW_016533273.1	171473	172086	+	434
novel.2404	NW_016533259.1	167538	168704	-	1138
novel.2405	NW_016533259.1	171347	173225	-	1822
novel.2406	NW_016533266.1	83048	93571	+	1566
novel.2407	NW_016533266.1	96270	97911	+	995
novel.2400	NW_016533257.1	5614	8013	+	2400
novel.2401	NW_016533257.1	5612	9191	-	2510
novel.2402	NW_016533257.1	22211	23479	-	243
novel.2403	NW_016533259.1	167538	168383	+	236
novel.940	NW_016529410.1	444860	449692	-	428
novel.941	NW_016529414.1	67298	68799	+	466
novel.942	NW_016529414.1	405276	406149	+	874
novel.943	NW_016529414.1	3322	3576	-	229
novel.944	NW_016529414.1	405276	406150	-	875
novel.945	NW_016529416.1	89993	93559	+	698
novel.946	NW_016529416.1	56175	62351	-	603

novel.947	NW_016529416.1	89955	95695	-	376
novel.948	NW_016529422.1	9285	10728	-	1444
novel.949	NW_016529426.1	203056	203768	+	713
novel.6601	NW_016553627.1	23698	24131	-	388
novel.6607	NW_016553687.1	5327	5962	-	579
novel.17	NW_016527297.1	92587	94743	-	2157
novel.16	NW_016527297.1	659948	664072	+	404
novel.15	NW_016527296.1	25255	25579	+	304
novel.14	NW_016527296.1	10906	17575	+	796
novel.13	NW_016527291.1	171439	171961	+	523
novel.12	NW_016527290.1	26881	27201	+	298
novel.11	NW_016527284.1	493612	505342	-	569
novel.10	NW_016527282.1	57343	59863	+	373
novel.19	NW_016527301.1	126016	130307	+	242
novel.18	NW_016527297.1	655513	658435	-	960
novel.4763	NW_016541560.1	31579	31917	+	282
novel.4762	NW_016541546.1	80892	81848	+	752
novel.4761	NW_016541540.1	120803	121053	-	206
novel.4760	NW_016541537.1	30709	38676	-	254
novel.4767	NW_016541589.1	21827	22381	-	431
novel.4766	NW_016541574.1	19414	20559	-	1068
novel.4765	NW_016541574.1	82708	86132	+	3313
novel.4764	NW_016541574.1	554	1150	+	444
novel.4769	NW_016541599.1	39120	40762	-	222
novel.4768	NW_016541590.1	49067	88802	+	626
novel.6408	NW_016551768.1	5725	6956	-	507
novel.6409	NW_016551788.1	6877	12923	+	833
novel.5354	NW_016544576.1	216449	223491	-	218
novel.6400	NW_016551703.1	43	372	+	296
novel.6401	NW_016551708.1	174064	177218	-	1058
novel.6402	NW_016551719.1	140176	235080	-	3893
novel.6403	NW_016551724.1	17576	17845	-	213
novel.6404	NW_016551741.1	4776	5323	+	493
novel.6405	NW_016551741.1	1	4620	-	4464
novel.6406	NW_016551753.1	15146	15710	+	565
novel.6407	NW_016551768.1	4536	5351	-	745
novel.5356	NW_016544596.1	11054	13914	+	1544
novel.4370	NW_016540109.1	1	659	-	524
novel.221	NW_016527727.1	24410	76961	+	1927
novel.4372	NW_016540111.1	70847	80353	+	6188
novel.4373	NW_016540111.1	79315	80355	-	671
novel.4374	NW_016540129.1	2473	3216	+	724
novel.4375	NW_016540134.1	251345	252068	-	724
novel.4376	NW_016540136.1	33490	34295	-	806

novel.4377	NW_016540138.1	4039	4953	-	915
novel.4378	NW_016540141.1	62118	62533	+	236
novel.4379	NW_016540147.1	6915	12127	-	3040
novel.223	NW_016527730.1	90195	90582	+	210
novel.550	NW_016528460.1	45478	47012	+	252
novel.5359	NW_016544608.1	102732	110842	+	8048
novel.5358	NW_016544603.1	28643	29078	+	323
novel.7960	NW_016577526.1	406	5851	-	209
novel.227	NW_016527735.1	7858	16346	+	779
novel.7961	NW_016577555.1	1	551	-	551
novel.226	NW_016527730.1	84038	122697	-	300
novel.7962	NW_016577743.1	4684	8080	+	2391
novel.445	NW_016528238.1	128221	128629	+	356
novel.444	NW_016528234.1	91700	91994	-	295
novel.447	NW_016528238.1	1059	1612	-	519
novel.446	NW_016528238.1	374768	377574	+	2751
novel.441	NW_016528220.1	149746	150022	-	232
novel.440	NW_016528220.1	166491	174109	+	474
novel.443	NW_016528228.1	176166	185681	-	240
novel.442	NW_016528224.1	17551	18725	+	462
novel.7964	NW_016577853.1	32	3658	+	530
novel.449	NW_016528240.1	164806	165187	-	265
novel.448	NW_016528240.1	64927	144122	+	1792
novel.7965	NW_016577885.1	1	1036	+	989
novel.834	NW_016529190.1	353205	354268	-	856
novel.7966	NW_016577896.1	22542	22887	+	346
novel.7967	NW_016577896.1	20344	21047	-	528
novel.1849	NW_016531593.1	5999	6701	-	587
novel.1848	NW_016531593.1	3198	4350	+	1153
novel.4488	NW_016540573.1	412560	415665	-	1698
novel.1841	NW_016531566.1	609	902	+	237
novel.1840	NW_016531561.1	419252	424562	-	5311
novel.1843	NW_016531575.1	54313	54663	-	351
novel.1842	NW_016531570.1	7572	7940	-	314
novel.1845	NW_016531578.1	10563	12051	-	663
novel.1844	NW_016531578.1	342046	342249	+	204
novel.1847	NW_016531591.1	71214	142648	-	253
novel.1846	NW_016531580.1	101075	101633	-	442
novel.5179	NW_016543555.1	480435	482278	+	760
novel.5178	NW_016543542.1	63804	64264	-	441
novel.8205	NW_016586163.1	5316	6607	-	815
novel.5171	NW_016543494.1	110843	113175	+	258
novel.5170	NW_016543478.1	499	1222	+	707
novel.5173	NW_016543499.1	54312	54874	+	563

novel.5172	NW_016543494.1	114606	115511	-	906
novel.5175	NW_016543529.1	28694	98165	+	1422
novel.5174	NW_016543519.1	25360	27604	+	1774
novel.5177	NW_016543542.1	63368	63699	-	279
novel.5176	NW_016543539.1	80760	82278	-	1468
novel.8207	NW_016586202.1	163644	163979	-	310
novel.4489	NW_016540573.1	399698	408419	-	1139
novel.8200	NW_016586120.1	5882	6472	+	535
novel.8201	NW_016586148.1	614	992	+	344
novel.7111	NW_016559120.1	17622	18107	-	486
novel.7110	NW_016559120.1	16142	16709	-	536
novel.7113	NW_016559150.1	2132	3022	+	799
novel.7112	NW_016559120.1	20770	21292	-	246
novel.7115	NW_016559211.1	3004	3801	+	754
novel.7114	NW_016559157.1	1970	2318	-	349
novel.1940	NW_016531837.1	8727	9306	-	437
novel.7116	NW_016559211.1	3004	9422	-	649
novel.7119	NW_016559224.1	10302	32557	-	328
novel.7118	NW_016559215.1	12181	12815	-	587
novel.7449	NW_016565114.1	3052	8975	-	1667
novel.7448	NW_016565114.1	2087	3971	+	1798
novel.1941	NW_016531838.1	56217	75480	+	444
novel.3871	NW_016538062.1	57614	60660	+	2981
novel.8468	NW_016596859.1	372	732	-	361
novel.8469	NW_016596882.1	1	1190	-	482
novel.8460	NW_016596450.1	1	1221	-	1221
novel.8461	NW_016596545.1	1	1311	+	476
novel.8462	NW_016596555.1	1	1299	-	780
novel.8463	NW_016596566.1	1	1313	-	1313
novel.8464	NW_016596658.1	1	784	+	784
novel.8465	NW_016596662.1	1	1284	-	1284
novel.8466	NW_016596779.1	1	1332	+	1332
novel.8467	NW_016596803.1	1	1334	+	416
novel.2912	NW_016534818.1	53302	53967	+	275
novel.1942	NW_016531838.1	86343	86906	+	305
novel.7340	NW_016563029.1	55602	56010	-	409
novel.7736	NW_016570550.1	1135	1647	+	513
novel.4847	NW_016541930.1	10890	15482	+	273
novel.3726	NW_016537599.1	174385	191032	-	524
novel.3727	NW_016537601.1	209719	210474	+	756
novel.3724	NW_016537593.1	127094	134945	-	6519
novel.3725	NW_016537599.1	25531	28681	-	3151
novel.3722	NW_016537593.1	109398	110114	+	482
novel.3723	NW_016537593.1	127095	133699	+	6605

novel.3720	NW_016537581.1	6559	9776	-	3189
novel.3721	NW_016537588.1	157926	161317	-	3117
novel.3728	NW_016537601.1	210537	211330	+	743
novel.3729	NW_016537605.1	125332	125689	-	323
novel.4978	NW_016542494.1	4287	4643	-	298
novel.4979	NW_016542503.1	9308	10196	-	323
novel.4976	NW_016542492.1	18847	20321	-	1363
novel.4977	NW_016542494.1	4466	5127	+	589
novel.4974	NW_016542482.1	6183	6984	+	304
novel.4975	NW_016542488.1	25095	26354	-	1203
novel.4972	NW_016542454.1	104927	105512	-	365
novel.4973	NW_016542460.1	15557	16367	+	811
novel.4970	NW_016542454.1	41107	46169	-	571
novel.4971	NW_016542454.1	53087	62125	-	516
novel.6151	NW_016549867.1	9624	10858	+	1188
novel.6237	NW_016550332.1	32044	32655	+	612
novel.6236	NW_016550330.1	11532	11862	+	278
novel.6235	NW_016550330.1	10635	10896	+	236
novel.6234	NW_016550305.1	2950	9287	-	269
novel.6233	NW_016550299.1	36481	87344	-	451
novel.6232	NW_016550290.1	7831	8690	-	803
novel.6231	NW_016550290.1	16370	19323	+	2931
novel.6230	NW_016550280.1	2420	4281	+	1862
novel.6239	NW_016550334.1	7383	7712	+	295
novel.6238	NW_016550332.1	35565	35858	+	236
novel.1447	NW_016530529.1	97263	98338	+	542
novel.5581	NW_016545904.1	12790	20452	+	242
novel.1614	NW_016530938.1	10678	14245	-	3521
novel.1615	NW_016530938.1	153557	153851	-	244
novel.1616	NW_016530944.1	38577	39132	+	502
novel.1617	NW_016530955.1	29874	30205	-	283
novel.1610	NW_016530935.1	3275	3576	+	246
novel.1611	NW_016530938.1	2326	9351	+	3727
novel.1612	NW_016530938.1	10678	14245	+	3517
novel.1613	NW_016530938.1	4892	9351	-	4460
novel.2318	NW_016533037.1	15694	16171	+	428
novel.1618	NW_016530961.1	96858	99385	+	864
novel.1619	NW_016530966.1	106741	107270	-	494
novel.2319	NW_016533037.1	133659	149034	+	1641
novel.148	NW_016527569.1	212506	213653	+	238
novel.149	NW_016527570.1	32041	32331	+	220
novel.5766	NW_016547064.1	15672	18903	-	3232
novel.5767	NW_016547069.1	1339	2034	-	670
novel.5760	NW_016547047.1	34117	34391	-	218

novel.5761	NW_016547048.1	13285	13915	+	631
novel.5762	NW_016547052.1	192905	201643	+	315
novel.5763	NW_016547052.1	224326	225722	-	507
novel.140	NW_016527548.1	46687	50380	-	1726
novel.141	NW_016527551.1	36920	38209	-	1290
novel.142	NW_016527553.1	13411	32472	+	511
novel.143	NW_016527557.1	4323	7225	-	527
novel.5768	NW_016547085.1	105067	117589	-	314
novel.145	NW_016527561.1	63429	79910	+	991
novel.146	NW_016527564.1	493430	496861	+	262
novel.147	NW_016527564.1	478328	481301	-	458
novel.7946	NW_016576615.1	115	2234	-	1186
novel.7947	NW_016576625.1	3042	3953	+	857
novel.7944	NW_016576559.1	3435	3861	+	251
novel.7945	NW_016576595.1	1031	1255	+	204
novel.7942	NW_016576479.1	2682	4696	+	1175
novel.7943	NW_016576508.1	3999	4707	+	217
novel.7940	NW_016576381.1	14744	16398	-	307
novel.7941	NW_016576407.1	757	1019	-	235
novel.2316	NW_016533034.1	145161	179934	+	1849
novel.7948	NW_016576625.1	2975	3783	-	809
novel.7949	NW_016576709.1	556	1057	+	470
novel.1478	NW_016530591.1	18458	19653	-	630
novel.1479	NW_016530591.1	21169	22562	-	1206
novel.2317	NW_016533035.1	304135	315405	-	3106
novel.1470	NW_016530581.1	158632	209234	+	622
novel.1471	NW_016530581.1	302810	308040	-	254
novel.1472	NW_016530581.1	513931	524949	-	248
novel.1473	NW_016530591.1	3992	4298	+	276
novel.1474	NW_016530591.1	13220	13890	+	623
novel.1475	NW_016530591.1	18299	22561	+	1606
novel.1476	NW_016530591.1	14778	14983	-	206
novel.1477	NW_016530591.1	15143	17472	-	285
novel.2312	NW_016533020.1	167179	167922	+	630
novel.2313	NW_016533022.1	46960	53703	-	852
novel.328	NW_016527997.1	208670	209127	+	418
novel.329	NW_016528000.1	18081	38918	-	462
novel.324	NW_016527989.1	80156	82459	+	2265
novel.325	NW_016527989.1	79194	80045	-	806
novel.326	NW_016527994.1	73959	111309	-	1245
novel.327	NW_016527996.1	278091	278660	+	375
novel.320	NW_016527967.1	3318	4120	-	761
novel.321	NW_016527972.1	153394	157607	-	224
novel.322	NW_016527984.1	180756	217807	-	262

novel.323	NW_016527987.1	146176	150843	-	1193
novel.4495	NW_016540577.1	29008	34219	+	394
novel.4494	NW_016540573.1	987481	990253	-	548
novel.4497	NW_016540577.1	829	1136	-	308
novel.4496	NW_016540577.1	29476	29984	+	481
novel.8129	NW_016583405.1	4177	5564	+	349
novel.8128	NW_016583367.1	1361	4677	-	3155
novel.7618	NW_016568540.1	1480	1806	+	270
novel.7619	NW_016568542.1	7985	8829	+	666
novel.7616	NW_016568515.1	66814	69234	+	1067
novel.8124	NW_016583288.1	39	290	+	212
novel.8127	NW_016583367.1	1368	3403	+	1909
novel.8126	NW_016583290.1	14041	14650	+	610
novel.7612	NW_016568340.1	7900	10462	+	581
novel.4498	NW_016540577.1	3279	5113	-	218
novel.8123	NW_016583238.1	772	2525	-	1171
novel.8122	NW_016583221.1	77	667	+	545
novel.3119	NW_016535582.1	30886	77303	+	2505
novel.3118	NW_016535580.1	27985	29125	-	927
novel.3115	NW_016535565.1	128918	129592	-	621
novel.3114	NW_016535565.1	50978	51313	-	281
novel.3117	NW_016535567.1	516175	516771	+	277
novel.3116	NW_016535566.1	8309	8762	-	454
novel.3111	NW_016535561.1	8820	55864	+	778
novel.3110	NW_016535558.1	617576	636918	-	733
novel.3113	NW_016535565.1	75921	77206	+	621
novel.3112	NW_016535563.1	113405	125661	+	511
novel.4149	NW_016539161.1	151137	152045	-	204
novel.4148	NW_016539161.1	5038	104036	-	907
novel.4147	NW_016539155.1	15389	180150	+	6916
novel.4146	NW_016539148.1	90473	91283	-	785
novel.4145	NW_016539146.1	73406	73674	+	214
novel.4144	NW_016539143.1	118155	121886	-	860
novel.4143	NW_016539136.1	1	101008	-	2376
novel.4142	NW_016539131.1	33135	50530	-	2070
novel.4141	NW_016539131.1	17607	25354	+	770
novel.4140	NW_016539110.1	121719	124584	+	279
novel.8697	NW_016605296.1	1	1042	-	465
novel.8696	NW_016605249.1	1	2952	+	315
novel.8695	NW_016605226.1	1	4022	-	3458
novel.8694	NW_016605197.1	1	3990	+	1377
novel.8693	NW_016605118.1	511	3918	-	445
novel.8692	NW_016605097.1	4	3896	+	1226
novel.8691	NW_016605089.1	1202	1677	-	476

novel.8690	NW_016605053.1	1	1750	-	351
novel.8699	NW_016605354.1	1003	4173	+	3171
novel.8698	NW_016605330.1	1870	3066	-	271
novel.5962	NW_016548518.1	20479	30920	+	6990
novel.5963	NW_016548518.1	25370	30635	-	5023
novel.5960	NW_016548505.1	146424	147582	-	742
novel.5961	NW_016548512.1	13543	20536	-	464
novel.5966	NW_016548555.1	16	650	-	595
novel.5967	NW_016548560.1	113875	119071	+	484
novel.5964	NW_016548532.1	2427	2844	+	361
novel.5965	NW_016548552.1	241741	245677	+	777
novel.5968	NW_016548560.1	100862	114263	-	233
novel.4121	NW_016538996.1	63499	66468	+	2970
novel.7630	NW_016568698.1	46493	49256	+	414
novel.8106	NW_016582580.1	5449	10544	-	285
novel.8105	NW_016582580.1	8102	9386	+	317
novel.8104	NW_016582580.1	5449	10795	+	1318
novel.8103	NW_016582491.1	1031	5601	-	2391
novel.8102	NW_016582386.1	4952	10511	-	540
novel.8101	NW_016582386.1	10190	10691	+	448
novel.7637	NW_016568861.1	404	1751	+	1348
novel.2466	NW_016533436.1	50953	54525	-	3485
novel.2467	NW_016533461.1	211	580	+	370
novel.2464	NW_016533435.1	63507	69320	-	325
novel.2465	NW_016533436.1	54644	56245	+	1602
novel.2462	NW_016533427.1	22668	27336	-	4669
novel.2463	NW_016533427.1	27393	28643	-	1049
novel.2460	NW_016533426.1	89380	126164	-	798
novel.2461	NW_016533427.1	19770	21289	+	963
novel.2398	NW_016533255.1	3373	9704	+	1900
novel.2399	NW_016533255.1	9956	10700	-	304
novel.2468	NW_016533465.1	308848	327954	+	4818
novel.2469	NW_016533466.1	270727	272949	+	484
novel.926	NW_016529395.1	365149	367679	+	1123
novel.927	NW_016529395.1	655853	661022	+	724
novel.924	NW_016529393.1	1	4126	-	1091
novel.925	NW_016529393.1	751105	751382	-	278
novel.922	NW_016529392.1	54164	54558	+	395
novel.923	NW_016529393.1	87017	88436	+	264
novel.920	NW_016529388.1	87709	88559	+	456
novel.921	NW_016529388.1	134523	137620	+	226
novel.928	NW_016529395.1	364436	369039	-	4504
novel.929	NW_016529398.1	24098	26258	-	320
novel.8109	NW_016582842.1	1668	2101	+	401

novel.8108	NW_016582688.1	6667	11948	-	2266
novel.6941	NW_016557015.1	1	1216	+	1193
novel.6940	NW_016556981.1	40181	42024	-	1560
novel.3585	NW_016537152.1	2613	5580	+	2801
novel.6943	NW_016557030.1	8419	14338	+	356
novel.6942	NW_016557019.1	14130	21907	+	2617
novel.3133	NW_016535625.1	286009	288012	+	2004
novel.3132	NW_016535621.1	3171	3979	-	280
novel.6947	NW_016557088.1	10502	13263	+	321
novel.6946	NW_016557083.1	68468	68819	-	306
novel.7146	NW_016559629.1	313	3416	+	3049
novel.7147	NW_016559630.1	11230	23434	-	288
novel.7144	NW_016559623.1	77364	78898	-	1472
novel.7145	NW_016559627.1	46553	47652	+	1017
novel.39	NW_016527349.1	149960	150283	+	272
novel.38	NW_016527349.1	105447	106933	+	1431
novel.35	NW_016527342.1	51837	254974	+	300
novel.34	NW_016527341.1	18323	19718	-	674
novel.37	NW_016527349.1	95083	95449	+	314
novel.36	NW_016527344.1	147003	147310	+	253
novel.31	NW_016527329.1	377095	382670	+	938
novel.30	NW_016527329.1	214356	216437	+	439
novel.33	NW_016527331.1	51325	58066	-	4510
novel.32	NW_016527329.1	393564	393817	-	201
novel.7140	NW_016559578.1	10634	11107	+	326
novel.7141	NW_016559578.1	9780	10582	-	760
novel.6398	NW_016551658.1	224644	234717	+	1587
novel.6399	NW_016551686.1	216859	218473	-	1615
novel.4707	NW_016541341.1	3956	8927	-	4965
novel.4706	NW_016541341.1	14424	18209	+	1215
novel.4701	NW_016541333.1	11102	11472	+	371
novel.4700	NW_016541330.1	14332	18221	+	3682
novel.4703	NW_016541333.1	231343	233144	-	1338
novel.4702	NW_016541333.1	203982	204397	+	416
novel.6390	NW_016551581.1	10856	11084	+	229
novel.6391	NW_016551581.1	75482	76670	+	1146
novel.6392	NW_016551587.1	82953	83264	+	264
novel.6393	NW_016551595.1	507914	509299	-	260
novel.6394	NW_016551595.1	528931	529825	-	206
novel.4708	NW_016541341.1	16012	17404	-	1340
novel.6396	NW_016551605.1	10181	71084	-	229
novel.6397	NW_016551646.1	28344	36400	-	1927
novel.1287	NW_016530121.1	236191	238125	-	1891
novel.1286	NW_016530121.1	236069	238125	+	1999

novel.1285	NW_016530118.1	59332	150624	-	2831
novel.1284	NW_016530118.1	149069	157890	+	1847
novel.1283	NW_016530115.1	143083	150207	-	223
novel.1282	NW_016530114.1	167545	169426	-	1187
novel.1281	NW_016530107.1	25562	26099	-	406
novel.1280	NW_016530106.1	269465	270628	-	283
novel.1757	NW_016531376.1	8	549	-	266
novel.1289	NW_016530123.1	8508	8791	+	284
novel.1288	NW_016530121.1	238206	238590	-	329
novel.1756	NW_016531376.1	10993	12114	+	1065
novel.1751	NW_016531374.1	123477	127640	-	1146
novel.1750	NW_016531374.1	123583	124488	+	871
novel.1753	NW_016531376.1	3250	8999	+	457
novel.1752	NW_016531376.1	1123	1802	+	504
novel.1863	NW_016531624.1	177	683	+	507
novel.1862	NW_016531622.1	187621	189689	+	243
novel.1861	NW_016531621.1	75605	75850	+	224
novel.1860	NW_016531616.1	5738	80581	-	2591
novel.1867	NW_016531638.1	15865	33397	+	513
novel.1866	NW_016531632.1	49801	51130	-	1330
novel.1865	NW_016531630.1	46922	48231	+	524
novel.1864	NW_016531629.1	92227	93181	+	740
novel.1869	NW_016531644.1	26605	26863	+	215
novel.1868	NW_016531641.1	8525	8940	+	416
novel.5153	NW_016543358.1	77467	88383	+	4277
novel.5152	NW_016543338.1	115829	116779	+	563
novel.5151	NW_016543330.1	178191	191491	-	655
novel.5150	NW_016543313.1	63104	63623	-	406
novel.5157	NW_016543383.1	8213	9019	+	588
novel.5156	NW_016543381.1	71920	80429	-	886
novel.5155	NW_016543358.1	74492	76940	-	2449
novel.5154	NW_016543358.1	257435	260059	+	1068
novel.5159	NW_016543387.1	3076	3852	+	777
novel.5158	NW_016543384.1	14383	15254	+	320
novel.7139	NW_016559578.1	8868	10577	+	1247
novel.7138	NW_016559575.1	6002	6857	-	815
novel.7133	NW_016559476.1	4971	10706	-	781
novel.7132	NW_016559446.1	28130	31211	-	1618
novel.7131	NW_016559419.1	5019	5906	-	223
novel.7130	NW_016559377.1	4524	5059	+	536
novel.7137	NW_016559542.1	772	1013	-	218
novel.7136	NW_016559505.1	42409	46011	-	3603
novel.7135	NW_016559505.1	2624	4829	-	2152
novel.7134	NW_016559503.1	30222	32700	-	501

novel.7425	NW_016564419.1	983	1228	-	205
novel.7424	NW_016564418.1	2150	2504	-	323
novel.7427	NW_016564513.1	5330	5716	+	352
novel.7426	NW_016564497.1	16938	19819	-	548
novel.7421	NW_016564337.1	9894	10633	+	293
novel.7420	NW_016564331.1	32441	52706	+	315
novel.7423	NW_016564409.1	8727	10637	+	1042
novel.7422	NW_016564338.1	10389	13854	-	848
novel.7429	NW_016564589.1	1021	1304	+	202
novel.7428	NW_016564527.1	2406	4016	-	1286
novel.469	NW_016528295.1	150645	150978	-	280
novel.468	NW_016528287.1	15468	15731	-	219
novel.463	NW_016528266.1	564503	564763	+	261
novel.462	NW_016528266.1	307165	307489	+	270
novel.461	NW_016528264.1	157918	158829	-	598
novel.460	NW_016528264.1	141442	149340	-	369
novel.467	NW_016528277.1	12751	86558	-	1167
novel.466	NW_016528272.1	2774	15026	-	869
novel.465	NW_016528267.1	125804	126147	-	287
novel.464	NW_016528266.1	564503	564763	-	261
novel.8482	NW_016597411.1	1	982	-	982
novel.8483	NW_016597414.1	41	1052	+	915
novel.8480	NW_016597064.1	28	1194	+	1167
novel.8481	NW_016597303.1	1	1373	-	427
novel.8486	NW_016597550.1	1	831	-	337
novel.8487	NW_016597568.1	1	1415	-	607
novel.8484	NW_016597420.1	1	1399	+	1399
novel.8485	NW_016597517.1	1	904	+	904
novel.8488	NW_016597587.1	1	556	-	302
novel.8489	NW_016597847.1	17	1088	+	1072
novel.3708	NW_016537535.1	189142	192304	-	3163
novel.3709	NW_016537549.1	18799	20087	-	960
novel.3258	NW_016535960.1	112741	113068	+	276
novel.3259	NW_016535960.1	115277	116595	+	1220
novel.3700	NW_016537529.1	40504	42145	+	1642
novel.3253	NW_016535945.1	5672	6096	+	230
novel.3250	NW_016535942.1	647	870	-	224
novel.3251	NW_016535943.1	26825	30267	-	3443
novel.3256	NW_016535959.1	20325	20776	+	405
novel.3705	NW_016537535.1	208223	211484	+	1160
novel.3706	NW_016537535.1	178673	185106	-	6434
novel.3255	NW_016535959.1	7069	8236	+	640
novel.4950	NW_016542386.1	667105	673199	+	3160
novel.4951	NW_016542386.1	608404	610012	-	1001

novel.4952	NW_016542386.1	616991	618163	-	1089
novel.4953	NW_016542386.1	659673	660796	-	232
novel.4954	NW_016542397.1	56605	59565	+	347
novel.4955	NW_016542397.1	82921	106905	+	216
novel.4956	NW_016542412.1	198925	201415	-	2387
novel.4957	NW_016542414.1	463	1141	+	622
novel.4958	NW_016542428.1	65482	100710	+	230
novel.4959	NW_016542428.1	115086	122999	+	766
novel.2486	NW_016533521.1	96652	117914	-	242
novel.2079	NW_016532211.1	34276	48885	-	420
novel.2078	NW_016532203.1	100233	101024	-	738
novel.2071	NW_016532197.1	76498	77836	-	857
novel.2070	NW_016532197.1	74751	75269	+	239
novel.2073	NW_016532198.1	404154	406232	+	1750
novel.2072	NW_016532198.1	27550	31082	+	873
novel.2075	NW_016532201.1	1281	1874	+	566
novel.2074	NW_016532199.1	28	47227	+	922
novel.2077	NW_016532203.1	96777	97704	-	904
novel.2076	NW_016532203.1	95805	99409	+	1828
novel.1678	NW_016531181.1	43999	44244	-	217
novel.1679	NW_016531198.1	42575	44327	+	825
novel.1676	NW_016531176.1	3886	20209	-	843
novel.1677	NW_016531177.1	261893	262380	+	458
novel.1674	NW_016531176.1	4199	10256	+	995
novel.1675	NW_016531176.1	19679	20713	+	985
novel.1672	NW_016531169.1	234641	247321	+	1604
novel.1673	NW_016531170.1	12717	12980	-	208
novel.1670	NW_016531157.1	311382	334694	-	1153
novel.1671	NW_016531165.1	1074	2215	+	1142
novel.5708	NW_016546678.1	71019	71705	+	687
novel.5709	NW_016546678.1	61083	84508	-	1694
novel.5706	NW_016546666.1	12338	14377	-	517
novel.5707	NW_016546678.1	3527	4117	+	591
novel.5704	NW_016546650.1	844	1059	-	216
novel.5705	NW_016546650.1	2282	4850	-	466
novel.5702	NW_016546644.1	5990	8306	-	731
novel.5703	NW_016546650.1	13938	14213	+	276
novel.5700	NW_016546641.1	248990	250297	+	221
novel.5701	NW_016546644.1	7206	7559	+	314
novel.7968	NW_016578038.1	227	1275	+	712
novel.7969	NW_016578041.1	45758	49749	-	3992
novel.128	NW_016527518.1	177634	178374	-	375
novel.129	NW_016527521.1	69489	70951	-	538
novel.126	NW_016527516.1	254173	367394	+	926

novel.127	NW_016527517.1	2690	24652	-	698
novel.124	NW_016527509.1	295897	297256	+	440
novel.125	NW_016527513.1	233872	234493	-	593
novel.122	NW_016527507.1	21677	23324	-	678
novel.123	NW_016527507.1	231725	231954	-	230
novel.120	NW_016527503.1	1679073	1680974	-	237
novel.121	NW_016527507.1	236736	238116	+	989
novel.5940	NW_016548356.1	2472	2850	-	344
novel.1492	NW_016530612.1	250450	252900	+	2451
novel.1493	NW_016530612.1	334439	334948	+	313
novel.1490	NW_016530612.1	11192	13133	+	1482
novel.1491	NW_016530612.1	232149	234956	+	762
novel.1496	NW_016530623.1	1	300	-	248
novel.1497	NW_016530633.1	140342	144345	+	1616
novel.1494	NW_016530612.1	11404	14275	-	2466
novel.1495	NW_016530612.1	145302	146244	-	633
novel.1498	NW_016530637.1	197865	201037	-	3033
novel.1499	NW_016530643.1	340860	346063	+	371
novel.5941	NW_016548357.1	5534	5806	+	273
novel.694	NW_016528799.1	215778	217217	-	911
novel.695	NW_016528799.1	329521	330215	-	402
novel.696	NW_016528799.1	660153	662586	-	2393
novel.697	NW_016528814.1	80911	83702	+	229
novel.690	NW_016528799.1	511308	517467	+	1536
novel.691	NW_016528799.1	657841	666483	+	7408
novel.692	NW_016528799.1	667271	667515	+	245
novel.693	NW_016528799.1	762479	764829	+	546
novel.698	NW_016528819.1	112561	113473	+	539
novel.699	NW_016528823.1	26451	41386	+	1029
novel.5942	NW_016548357.1	6862	7068	+	207
novel.5513	NW_016545516.1	5016	5636	+	565
novel.5512	NW_016545503.1	49230	58505	+	336
novel.5511	NW_016545501.1	63736	64029	-	260
novel.5510	NW_016545498.1	296	497	+	202
novel.5517	NW_016545517.1	10462	14644	-	1124
novel.5516	NW_016545517.1	10030	13708	-	572
novel.5944	NW_016548357.1	5694	6331	-	613
novel.5515	NW_016545517.1	10039	13687	+	524
novel.5945	NW_016548358.1	30602	30846	-	245
novel.5514	NW_016545516.1	12314	15815	-	2478
novel.5946	NW_016548366.1	58286	208801	-	554
novel.6619	NW_016553847.1	799	2106	-	240
novel.7631	NW_016568698.1	8987	10788	-	589
novel.7632	NW_016568775.1	14429	14663	+	235

novel.7633	NW_016568794.1	47057	49316	+	2015
novel.7634	NW_016568807.1	2925	3660	-	700
novel.7635	NW_016568830.1	5689	6918	+	1230
novel.7636	NW_016568830.1	5689	7433	-	1689
novel.8100	NW_016582353.1	6	318	-	259
novel.6611	NW_016553787.1	13652	13904	-	253
novel.7639	NW_016568863.1	71501	72795	+	660
novel.6613	NW_016553803.1	6956	7407	+	272
novel.6612	NW_016553803.1	6270	6868	+	599
novel.6615	NW_016553820.1	2730	3214	+	460
novel.5519	NW_016545524.1	53937	54739	+	211
novel.6617	NW_016553820.1	14400	15183	-	738
novel.6616	NW_016553820.1	14430	15165	+	688
novel.3137	NW_016535627.1	104436	104716	+	281
novel.3136	NW_016535625.1	286009	288012	-	2004
novel.3135	NW_016535625.1	78988	80234	-	1192
novel.2238	NW_016532708.1	242	720	+	430
novel.6945	NW_016557064.1	213717	225676	+	622
novel.6944	NW_016557058.1	4614	4988	+	318
novel.3131	NW_016535619.1	25690	25967	+	227
novel.3130	NW_016535618.1	13799	14028	-	230
novel.6949	NW_016557099.1	13503	15964	-	372
novel.6948	NW_016557090.1	50827	52419	+	1544
novel.2663	NW_016534015.1	66905	67232	+	328
novel.3139	NW_016535627.1	108051	108386	+	336
novel.3138	NW_016535627.1	104908	106198	+	322
novel.3717	NW_016537571.1	15830	16358	+	529
novel.3716	NW_016537564.1	669039	678558	-	737
novel.3715	NW_016537559.1	68176	70192	-	1058
novel.3714	NW_016537555.1	43031	43452	-	221
novel.2668	NW_016534036.1	6920	8577	-	1200
novel.5949	NW_016548431.1	7293	7610	-	318
novel.8815	NW_016607497.1	1	5536	-	1481
novel.8814	NW_016607493.1	4	3831	-	3521
novel.8817	NW_016607502.1	3907	7360	-	3454
novel.8816	NW_016607502.1	3907	7360	+	3454
novel.8811	NW_016607437.1	9079	9850	+	393
novel.8810	NW_016607436.1	3433	7239	+	490
novel.8813	NW_016607480.1	8380	11522	+	318
novel.8812	NW_016607471.1	6619	7816	+	1198
novel.3246	NW_016535932.1	6231	7908	-	238
novel.8819	NW_016607523.1	386	735	+	350
novel.8818	NW_016607513.1	2667	9826	-	328
novel.7350	NW_016563197.1	8075	11550	-	2290

novel.7357	NW_016563311.1	2594	4470	+	1844
novel.7356	NW_016563311.1	53	1048	+	996
novel.7355	NW_016563291.1	9817	10366	+	278
novel.7354	NW_016563289.1	68696	69530	+	835
novel.7098	NW_016559030.1	42157	69093	-	515
novel.7099	NW_016559099.1	55733	56727	+	878
novel.2481	NW_016533508.1	34033	34433	+	345
novel.7094	NW_016558884.1	152432	154240	-	1087
novel.7095	NW_016558886.1	44620	45349	-	730
novel.7096	NW_016558895.1	16700	20213	-	2140
novel.7097	NW_016558956.1	520	1212	-	657
novel.7090	NW_016558806.1	168672	168997	+	269
novel.7091	NW_016558842.1	2803	3137	+	295
novel.7092	NW_016558882.1	4599	5044	+	320
novel.7093	NW_016558884.1	39064	43847	-	1225
novel.3281	NW_016536053.1	18916	19624	-	709
novel.2664	NW_016534022.1	13621	14096	+	314
novel.2665	NW_016534024.1	5448	6056	+	609
novel.2666	NW_016534024.1	6166	9212	+	3001
novel.2667	NW_016534036.1	6920	7728	+	763
novel.2660	NW_016534004.1	1094	8225	+	459
novel.2661	NW_016534008.1	24592	25300	+	517
novel.2662	NW_016534013.1	4505	5042	+	253
novel.5943	NW_016548357.1	7220	7897	+	623
novel.5948	NW_016548411.1	8617	9298	+	682
novel.2669	NW_016534039.1	250220	254080	+	549
novel.3280	NW_016536053.1	1009	12405	+	1001
novel.3399	NW_016536465.1	660	943	+	229
novel.3398	NW_016536452.1	13166	20777	-	585
novel.3393	NW_016536422.1	469472	469680	-	209
novel.3392	NW_016536422.1	458523	469119	+	1865
novel.3391	NW_016536419.1	8136	8595	+	421
novel.3390	NW_016536414.1	21729	22258	-	304
novel.3397	NW_016536447.1	134670	135245	-	524
novel.3396	NW_016536435.1	17385	17721	+	280
novel.3395	NW_016536429.1	101529	102060	+	479
novel.3394	NW_016536422.1	469776	470108	-	277
novel.2448	NW_016533391.1	333455	339713	-	555
novel.2449	NW_016533391.1	340010	341520	-	999
novel.2918	NW_016534857.1	27383	31493	+	361
novel.2919	NW_016534857.1	471904	475104	-	3201
novel.5498	NW_016545451.1	17788	19286	-	1442
novel.5499	NW_016545452.1	14890	16216	+	428
novel.5496	NW_016545416.1	115906	116896	+	991

novel.2913	NW_016534818.1	369291	376367	-	6175
novel.2442	NW_016533387.1	183330	183674	-	345
novel.2443	NW_016533387.1	263471	267394	-	2693
novel.2444	NW_016533388.1	114383	121426	-	384
novel.2445	NW_016533388.1	194317	195047	-	711
novel.2914	NW_016534821.1	102459	103996	-	635
novel.2447	NW_016533391.1	255050	316993	+	594
novel.908	NW_016529356.1	152928	153559	-	597
novel.909	NW_016529361.1	403651	403933	+	226
novel.904	NW_016529330.1	23166	25098	+	1933
novel.905	NW_016529337.1	27649	28594	+	331
novel.906	NW_016529344.1	58633	167064	+	277
novel.907	NW_016529344.1	129289	165146	-	453
novel.900	NW_016529316.1	186707	188468	-	1457
novel.901	NW_016529318.1	141	6229	+	218
novel.902	NW_016529325.1	6848	15721	-	324
novel.903	NW_016529326.1	25193	32142	+	203
novel.5265	NW_016543970.1	1042	1568	-	527
novel.358	NW_016528048.1	867322	868996	+	1675
novel.5267	NW_016543981.1	69585	70684	+	414
novel.2440	NW_016533387.1	1005743	1013008	+	7266
novel.6992	NW_016557685.1	4874	5191	+	264
novel.5266	NW_016543975.1	108560	109644	+	734
novel.2441	NW_016533387.1	1019139	1019975	+	837
novel.2910	NW_016534808.1	29860	58904	-	3134
novel.2911	NW_016534818.1	20844	22241	+	1398
novel.2916	NW_016534828.1	52928	54331	-	1404
novel.2917	NW_016534833.1	152486	153826	-	1341
novel.603	NW_016528579.1	49527	54621	+	220
novel.2446	NW_016533390.1	56791	57093	+	276
novel.4729	NW_016541421.1	22873	23447	-	575
novel.4728	NW_016541421.1	22935	23448	+	455
novel.4727	NW_016541416.1	55613	56309	+	435
novel.4726	NW_016541416.1	18171	18647	+	429
novel.4725	NW_016541402.1	48332	48680	-	292
novel.2915	NW_016534828.1	45994	46723	-	699
novel.4723	NW_016541401.1	48424	49800	-	275
novel.4722	NW_016541401.1	371135	408484	+	5420
novel.4721	NW_016541395.1	16664	22849	-	237
novel.4720	NW_016541392.1	8773	45456	+	3057
novel.6372	NW_016551348.1	4624	6349	-	1586
novel.6373	NW_016551352.1	38358	41704	-	953
novel.6370	NW_016551338.1	2756	19779	-	516
novel.6371	NW_016551348.1	5495	6354	+	837

novel.6376	NW_016551375.1	18285	20893	+	765
novel.6377	NW_016551378.1	4246	4572	+	285
novel.6374	NW_016551354.1	43388	43828	-	324
novel.6375	NW_016551361.1	14977	15275	+	299
novel.607	NW_016528580.1	34128	36819	+	2520
novel.6378	NW_016551435.1	12171	15715	-	209
novel.6379	NW_016551437.1	1168	1652	-	438
novel.606	NW_016528579.1	200373	201343	-	771
novel.357	NW_016528048.1	212320	219080	+	800
novel.356	NW_016528048.1	73156	74219	+	1064
novel.3836	NW_016537910.1	150048	151619	+	1139
novel.3837	NW_016537928.1	23038	23786	-	749
novel.3834	NW_016537892.1	3401	3659	+	202
novel.3835	NW_016537895.1	72769	74954	-	2130
novel.3832	NW_016537890.1	109894	110116	-	223
novel.3833	NW_016537890.1	110338	112715	-	828
novel.3830	NW_016537885.1	15298	15732	+	395
novel.6803	NW_016555645.1	145968	151559	+	429
novel.8374	NW_016591651.1	1	1051	-	1001
novel.7536	NW_016566801.1	1	5493	+	316
novel.7535	NW_016566766.1	65640	67066	-	866
novel.7534	NW_016566766.1	13432	16584	-	1587
novel.7533	NW_016566764.1	148680	149824	+	236
novel.7532	NW_016566754.1	787	2265	+	709
novel.7531	NW_016566719.1	4097	4928	-	576
novel.7530	NW_016566718.1	91327	97091	+	233
novel.1805	NW_016531468.1	1994	2572	+	546
novel.1804	NW_016531467.1	17880	18220	+	285
novel.1807	NW_016531478.1	738121	739077	-	392
novel.1806	NW_016531478.1	326748	327286	-	490
novel.1801	NW_016531465.1	6186	9311	+	283
novel.1800	NW_016531464.1	24369	24711	-	291
novel.1803	NW_016531466.1	32343	32834	-	297
novel.1802	NW_016531465.1	40303	48565	+	1431
novel.1809	NW_016531483.1	122510	123735	+	535
novel.1808	NW_016531479.1	39587	39893	-	250
novel.5135	NW_016543225.1	448827	449296	-	213
novel.5134	NW_016543211.1	1482	3565	-	2051
novel.5137	NW_016543244.1	162562	163404	-	752
novel.5136	NW_016543235.1	7530	8668	-	1139
novel.5131	NW_016543165.1	55626	90070	+	467
novel.5130	NW_016543158.1	522129	522558	-	395
novel.5133	NW_016543211.1	1	1269	-	1239
novel.5132	NW_016543173.1	5615	6671	+	1025

novel.5139	NW_016543258.1	40625	41585	+	881
novel.5138	NW_016543248.1	10375	25293	-	300
novel.8712	NW_016605535.1	1	4397	-	4397
novel.8713	NW_016605563.1	1	3364	-	3364
novel.8710	NW_016605463.1	437	3967	+	311
novel.8711	NW_016605525.1	8	4304	+	223
novel.8716	NW_016605582.1	1	1466	-	334
novel.8717	NW_016605608.1	430	3728	+	362
novel.8714	NW_016605567.1	1	4432	+	4432
novel.8715	NW_016605567.1	1	4432	-	4432
novel.7155	NW_016559733.1	20906	21375	+	219
novel.7154	NW_016559733.1	2643	2916	+	274
novel.8718	NW_016605630.1	1679	4444	-	499
novel.8719	NW_016605644.1	1387	4530	+	2215
novel.7151	NW_016559713.1	702	3331	-	2586
novel.7150	NW_016559700.1	11067	25603	+	1186
novel.7153	NW_016559728.1	14513	14879	-	310
novel.7152	NW_016559715.1	3286	3558	-	273
novel.3890	NW_016538137.1	138979	139384	+	355
novel.3891	NW_016538137.1	154927	157266	+	2301
novel.3892	NW_016538137.1	369237	369763	+	421
novel.3893	NW_016538137.1	154927	156555	-	1573
novel.3894	NW_016538137.1	170661	181118	-	412
novel.3895	NW_016538146.1	28518	33964	+	5320
novel.3896	NW_016538146.1	121415	139399	+	855
novel.3897	NW_016538149.1	275910	280336	-	283
novel.3898	NW_016538154.1	14153	14355	-	203
novel.3899	NW_016538159.1	167847	168767	+	921
novel.481	NW_016528318.1	171997	172647	+	256
novel.480	NW_016528315.1	370774	373906	+	3133
novel.483	NW_016528326.1	171638	259354	+	623
novel.482	NW_016528323.1	180151	181991	-	323
novel.485	NW_016528326.1	507616	508446	-	228
novel.484	NW_016528326.1	466413	466991	+	524
novel.487	NW_016528329.1	36251	38585	+	422
novel.486	NW_016528328.1	19274	19981	-	651
novel.489	NW_016528351.1	117411	117630	+	220
novel.488	NW_016528337.1	7886	8132	+	247
novel.3098	NW_016535544.1	154413	156007	-	1414
novel.3099	NW_016535545.1	6672	9381	-	2710
novel.3090	NW_016535527.1	218421	219023	+	603
novel.3091	NW_016535527.1	218047	218687	-	307
novel.3092	NW_016535531.1	450575	450937	+	309
novel.3093	NW_016535531.1	424710	438706	-	504

novel.3094	NW_016535536.1	165333	171501	+	6169
novel.3095	NW_016535539.1	260553	261532	-	980
novel.3096	NW_016535540.1	21552	168195	-	375
novel.3097	NW_016535540.1	116782	117249	-	279
novel.4268	NW_016539654.1	68824	71688	-	822
novel.4269	NW_016539660.1	286747	287026	+	235
novel.4262	NW_016539628.1	31316	36801	+	674
novel.4263	NW_016539628.1	59705	60019	-	258
novel.4260	NW_016539625.1	171839	172516	-	319
novel.4261	NW_016539628.1	14850	70591	+	1875
novel.4266	NW_016539630.1	103812	114513	+	267
novel.4267	NW_016539630.1	103812	107650	-	3839
novel.4264	NW_016539628.1	60916	65017	-	254
novel.4265	NW_016539628.1	65789	66630	-	747
novel.3278	NW_016536044.1	393772	395444	-	289
novel.3279	NW_016536046.1	207580	208538	+	959
novel.3274	NW_016536022.1	457249	457514	-	223
novel.3275	NW_016536034.1	160085	199075	+	982
novel.3276	NW_016536039.1	33623	34213	+	282
novel.3277	NW_016536039.1	33346	33844	-	337
novel.3270	NW_016536006.1	130771	132543	-	1773
novel.3271	NW_016536011.1	312411	313177	+	710
novel.3272	NW_016536022.1	452459	452705	+	247
novel.3273	NW_016536022.1	456533	456887	-	300
novel.8854	NW_016607931.1	47695	55040	-	7346
novel.2053	NW_016532173.1	164333	164646	+	314
novel.2052	NW_016532171.1	1211	1743	+	323
novel.2051	NW_016532163.1	67367	68131	-	709
novel.2050	NW_016532159.1	116516	117989	+	1474
novel.2057	NW_016532177.1	13849	15260	-	1360
novel.2056	NW_016532177.1	6125	8818	+	2662
novel.2055	NW_016532175.1	40423	45926	+	278
novel.2054	NW_016532173.1	182449	188168	+	379
novel.2059	NW_016532182.1	164605	172809	-	537
novel.2058	NW_016532178.1	6682	7021	-	291
novel.1122	NW_016529789.1	5738	21633	-	921
novel.1123	NW_016529790.1	4201	6223	+	1748
novel.1120	NW_016529784.1	398842	399560	-	412
novel.1121	NW_016529785.1	341964	342450	+	487
novel.1654	NW_016531105.1	12653	12872	+	220
novel.1655	NW_016531121.1	82857	83358	+	464
novel.1124	NW_016529790.1	6709	9377	+	463
novel.1125	NW_016529790.1	9542	10448	-	887
novel.1658	NW_016531127.1	46020	47998	+	1979

novel.1659	NW_016531127.1	178536	179167	+	486
novel.1128	NW_016529799.1	87218	93354	-	431
novel.1129	NW_016529800.1	267398	267787	+	390
novel.5720	NW_016546800.1	91690	92569	-	852
novel.5721	NW_016546815.1	5709	7069	+	1161
novel.5722	NW_016546815.1	7188	7766	+	492
novel.5723	NW_016546815.1	9044	13096	+	4053
novel.5724	NW_016546815.1	15266	16078	+	813
novel.5725	NW_016546815.1	16282	16561	+	223
novel.5726	NW_016546815.1	7071	7766	-	639
novel.5727	NW_016546823.1	26359	55253	+	1583
novel.5728	NW_016546828.1	352678	355834	-	486
novel.5729	NW_016546841.1	28912	31838	-	2716
novel.104	NW_016527472.1	405981	431555	+	302
novel.105	NW_016527473.1	101732	107660	+	5929
novel.106	NW_016527473.1	184580	185059	-	480
novel.7981	NW_016578367.1	17391	18685	-	1295
novel.100	NW_016527465.1	37989	39966	+	1165
novel.101	NW_016527465.1	201418	204835	-	1111
novel.102	NW_016527466.1	29314	29947	-	350
novel.103	NW_016527470.1	27476	27832	-	312
novel.7988	NW_016578487.1	10088	10526	+	384
novel.7989	NW_016578487.1	10961	11767	+	807
novel.108	NW_016527479.1	189271	240407	+	449
novel.109	NW_016527482.1	91860	94676	+	2817
novel.5424	NW_016545022.1	146122	153142	+	1982
novel.3446	NW_016536661.1	6935	8344	+	1317
novel.6589	NW_016553543.1	3542	4343	-	462
novel.6588	NW_016553543.1	21	471	+	230
novel.6581	NW_016553470.1	45596	46109	-	263
novel.6580	NW_016553470.1	35321	47882	-	556
novel.6583	NW_016553500.1	15050	15322	-	218
novel.6582	NW_016553477.1	5191	7108	+	1219
novel.6585	NW_016553529.1	4721	14673	+	4902
novel.6584	NW_016553504.1	41257	41549	-	247
novel.6587	NW_016553530.1	1568	67470	-	741
novel.6586	NW_016553530.1	104638	104879	+	242
novel.5421	NW_016544993.1	639	906	-	211
novel.4132	NW_016539069.1	78473	79991	+	1020
novel.4133	NW_016539072.1	19784	20478	+	695
novel.4130	NW_016539067.1	101436	102579	-	1099
novel.4451	NW_016540385.1	300225	324380	-	320
novel.4450	NW_016540377.1	5142	7337	-	900
novel.4453	NW_016540398.1	119131	119514	+	216

novel.4452	NW_016540387.1	5628	45928	+	225
novel.4455	NW_016540398.1	22133	23498	-	918
novel.4454	NW_016540398.1	19406	24883	-	570
novel.4457	NW_016540415.1	114325	115199	-	720
novel.4456	NW_016540398.1	119574	121361	-	1788
novel.4459	NW_016540418.1	359946	360828	-	300
novel.4458	NW_016540417.1	10719	12767	+	2049
novel.4137	NW_016539101.1	345044	345923	-	200
novel.6633	NW_016554032.1	319	1110	-	766
novel.6632	NW_016554032.1	319	1110	+	767
novel.6631	NW_016554020.1	779	2117	+	1317
novel.6630	NW_016554016.1	38857	41756	-	2900
novel.6637	NW_016554051.1	359	4870	-	464
novel.6636	NW_016554047.1	4387	5100	+	599
novel.6635	NW_016554043.1	1	1434	+	1408
novel.6634	NW_016554035.1	10027	10860	-	834
novel.6639	NW_016554069.1	1	474	+	426
novel.6638	NW_016554062.1	5451	6827	-	561
novel.4189	NW_016539304.1	30461	31102	+	519
novel.4188	NW_016539303.1	1671	3382	-	1655
novel.4183	NW_016539288.1	86112	119607	+	587
novel.4182	NW_016539285.1	45533	56824	-	339
novel.4181	NW_016539285.1	28643	30143	-	1053
novel.4180	NW_016539284.1	29355	74092	-	667
novel.4187	NW_016539303.1	3787	4301	+	515
novel.4186	NW_016539303.1	1	3386	+	2399
novel.4185	NW_016539302.1	238439	394093	+	498
novel.4184	NW_016539298.1	102254	105130	-	2654
novel.4724	NW_016541402.1	30660	64798	-	2079
novel.8389	NW_016591831.1	25150	32175	-	226
novel.8388	NW_016591820.1	74087	85218	+	1105
novel.8839	NW_016607828.1	8301	18827	+	622
novel.8838	NW_016607825.1	1	4357	-	4357
novel.8385	NW_016591734.1	1	568	-	568
novel.8836	NW_016607809.1	1958	9006	+	1076
novel.8835	NW_016607780.1	1	737	-	279
novel.8386	NW_016591734.1	798	2859	-	2037
novel.8381	NW_016591729.1	1	2154	+	1806
novel.8832	NW_016607672.1	1	5446	-	5446
novel.8383	NW_016591729.1	2055	2442	-	344
novel.8830	NW_016607666.1	1	5133	-	2062
novel.5540	NW_016545598.1	57914	58827	-	858
novel.5541	NW_016545602.1	1	2692	+	2345
novel.6969	NW_016557425.1	2781	3029	-	249

novel.6968	NW_016557425.1	1522	2482	+	903
novel.6963	NW_016557345.1	26165	26852	+	550
novel.6962	NW_016557266.1	16433	21152	-	372
novel.6961	NW_016557248.1	34915	35548	+	555
novel.6960	NW_016557219.1	31936	32286	-	351
novel.6967	NW_016557409.1	48266	53929	-	3782
novel.6966	NW_016557402.1	9280	9608	+	284
novel.6965	NW_016557402.1	8354	8636	+	230
novel.6964	NW_016557345.1	25736	26376	-	312
novel.5928	NW_016548322.1	135763	137009	+	1198
novel.5929	NW_016548322.1	229478	231854	+	583
novel.5926	NW_016548285.1	2554	10301	+	1353
novel.2607	NW_016533842.1	225501	227692	-	2192
novel.5924	NW_016548264.1	32885	33316	-	380
novel.5925	NW_016548283.1	248405	289161	-	1667
novel.5922	NW_016548264.1	20961	33641	+	574
novel.5923	NW_016548264.1	20553	21089	-	492
novel.5920	NW_016548227.1	151278	153648	-	933
novel.5921	NW_016548263.1	1747	2447	+	657
novel.8529	NW_016599514.1	1	503	+	426
novel.8528	NW_016599446.1	778	1448	-	671
novel.7218	NW_016560844.1	79919	80243	+	268
novel.7219	NW_016560863.1	7620	10684	+	507
novel.8521	NW_016599346.1	311	1648	-	1338
novel.8520	NW_016599326.1	474	1443	-	474
novel.7210	NW_016560643.1	91771	114618	-	420
novel.7211	NW_016560652.1	476	1767	+	430
novel.7216	NW_016560723.1	4092	5633	-	1514
novel.7217	NW_016560833.1	1470	1760	+	252
novel.7214	NW_016560721.1	173	4132	+	2694
novel.8526	NW_016599411.1	1	1658	-	1658
novel.5543	NW_016545602.1	1425	2692	-	1224
novel.2938	NW_016534937.1	65591	66247	-	657
novel.2939	NW_016534937.1	219544	220399	-	782
novel.5544	NW_016545607.1	7778	8723	-	644
novel.2934	NW_016534935.1	30629	31815	-	452
novel.2935	NW_016534936.1	26498	64638	+	547
novel.2936	NW_016534937.1	220120	221088	+	580
novel.2937	NW_016534937.1	242582	309686	+	1054
novel.2930	NW_016534915.1	58517	62228	-	1870
novel.2931	NW_016534920.1	49347	50164	-	254
novel.2932	NW_016534933.1	227698	228427	-	544
novel.2933	NW_016534935.1	22025	22787	-	707
novel.3	NW_016527274.1	94462	95258	+	797

novel.2	NW_016527272.1	19443	106169	-	409
novel.1	NW_016527272.1	88415	88912	+	498
novel.7	NW_016527277.1	361287	363814	+	1880
novel.6	NW_016527277.1	293416	318221	+	324
novel.5	NW_016527275.1	11447	12576	-	1041
novel.4	NW_016527274.1	94458	95261	-	804
novel.9	NW_016527278.1	314168	314485	+	287
novel.8	NW_016527277.1	361287	416999	-	3068
novel.5546	NW_016545607.1	24125	27053	-	367
novel.3519	NW_016536977.1	53894	92899	-	1950
novel.6038	NW_016549112.1	940	3433	+	2494
novel.3511	NW_016536913.1	9755	14807	+	1042
novel.3510	NW_016536907.1	14444	15421	-	448
novel.3513	NW_016536931.1	161699	167350	-	1526
novel.3512	NW_016536930.1	23946	56817	+	442
novel.3515	NW_016536955.1	78166	95324	+	1116
novel.3514	NW_016536938.1	6278	7051	+	673
novel.3517	NW_016536955.1	102569	103024	-	330
novel.3516	NW_016536955.1	491631	501519	+	390
novel.6354	NW_016551284.1	1345	2648	-	1096
novel.6355	NW_016551284.1	2735	3211	-	273
novel.6356	NW_016551289.1	16154	28066	-	208
novel.6357	NW_016551295.1	40086	43087	+	3002
novel.6350	NW_016551243.1	28380	28731	+	209
novel.6351	NW_016551276.1	19678	21211	+	354
novel.6352	NW_016551281.1	19662	20186	-	392
novel.6353	NW_016551284.1	1	1494	+	1253
novel.934	NW_016529407.1	88268	90728	-	2425
novel.6358	NW_016551295.1	282745	284390	+	1646
novel.6359	NW_016551295.1	283483	284293	+	405
novel.544	NW_016528451.1	17530	19217	+	390
novel.233	NW_016527741.1	2747	3040	+	241
novel.546	NW_016528456.1	11616	12982	+	793
novel.231	NW_016527737.1	408878	409554	-	366
novel.8266	NW_016587541.1	864	1709	+	846
novel.115	NW_016527497.1	150130	151909	+	1780
novel.236	NW_016527754.1	205898	208872	+	781
novel.114	NW_016527491.1	142319	151556	-	5251
novel.237	NW_016527759.1	106961	107467	-	416
novel.542	NW_016528448.1	231176	238447	-	247
novel.543	NW_016528451.1	17155	17419	+	265
novel.119	NW_016527503.1	1612905	1622818	-	1465
novel.8213	NW_016586292.1	4090	4787	+	641
novel.8267	NW_016587541.1	1	645	-	612

novel.118	NW_016527503.1	1652461	1658345	+	1093
novel.8212	NW_016586256.1	2040	2443	-	362
novel.8211	NW_016586213.1	75	1067	+	295
novel.1359	NW_016530292.1	243219	249120	+	323
novel.1358	NW_016530292.1	237158	237759	+	236
novel.8210	NW_016586212.1	10544	11768	-	1122
novel.1829	NW_016531541.1	25600	26366	+	425
novel.1828	NW_016531539.1	200326	201072	+	747
novel.1827	NW_016531533.1	144385	144721	-	282
novel.1826	NW_016531532.1	202387	203711	-	1325
novel.1353	NW_016530274.1	1324959	1369769	-	259
novel.1824	NW_016531531.1	227568	228537	-	252
novel.1823	NW_016531530.1	16936	17937	+	953
novel.1822	NW_016531525.1	3467	9048	-	5582
novel.1821	NW_016531525.1	35536	37491	+	1922
novel.1820	NW_016531523.1	281501	323298	-	509
novel.8264	NW_016587520.1	1	474	-	418
novel.8215	NW_016586333.1	5322	8871	-	690
novel.5119	NW_016543119.1	99617	101425	+	301
novel.5118	NW_016543112.1	64981	81569	-	283
novel.5117	NW_016543109.1	3903	4891	-	735
novel.5116	NW_016543109.1	4257	4891	+	635
novel.5115	NW_016543106.1	41108	42492	-	1385
novel.5114	NW_016543092.1	8457	8769	+	313
novel.5113	NW_016543083.1	4476	5232	+	408
novel.5112	NW_016543074.1	106170	146225	+	783
novel.5111	NW_016543068.1	133430	134128	+	252
novel.5110	NW_016543065.1	83856	85282	+	1427
novel.711	NW_016528834.1	25589	33567	-	7979
novel.710	NW_016528828.1	18665	49382	-	760
novel.713	NW_016528837.1	1839	3955	+	951
novel.712	NW_016528834.1	90009	90818	-	585
novel.715	NW_016528837.1	289432	290959	-	872
novel.714	NW_016528837.1	278358	279666	+	893
novel.717	NW_016528839.1	139056	139315	-	217
novel.716	NW_016528838.1	4492	12543	-	1353
novel.719	NW_016528846.1	393047	436385	+	1071
novel.718	NW_016528841.1	3441	53524	-	328
novel.8219	NW_016586392.1	2031	2331	+	245
novel.8265	NW_016587536.1	503	2741	-	2218
novel.7177	NW_016559847.1	6067	7170	-	1068
novel.7176	NW_016559847.1	5588	5837	-	250
novel.7175	NW_016559841.1	154601	157049	-	228
novel.7174	NW_016559841.1	52690	54860	-	286

novel.7173	NW_016559841.1	156935	174332	+	1895
novel.7172	NW_016559835.1	12370	14852	-	2009
novel.7171	NW_016559816.1	4138	8802	-	365
novel.8733	NW_016605837.1	3106	4364	-	570
novel.8738	NW_016605900.1	1	1920	+	1437
novel.8739	NW_016605900.1	1	3425	-	924
novel.7179	NW_016559891.1	953	1729	+	717
novel.7178	NW_016559864.1	785	991	-	207
novel.8262	NW_016587496.1	655	2601	-	1892
novel.1480	NW_016530591.1	280379	283242	-	2864
novel.3072	NW_016535468.1	10872	11319	+	448
novel.3073	NW_016535481.1	112910	113510	+	362
novel.3070	NW_016535449.1	869	16196	-	786
novel.3071	NW_016535457.1	31262	31597	+	336
novel.3076	NW_016535487.1	28529	30529	-	1398
novel.3077	NW_016535487.1	46436	47584	-	233
novel.3074	NW_016535481.1	36679	108424	-	507
novel.3075	NW_016535484.1	1116	2645	+	310
novel.8260	NW_016587429.1	442	766	-	325
novel.3078	NW_016535487.1	225447	226693	-	1216
novel.3079	NW_016535487.1	260199	261662	-	492
novel.1486	NW_016530597.1	396128	401086	+	323
novel.4248	NW_016539564.1	48112	48695	-	496
novel.4249	NW_016539566.1	98950	99578	+	266
novel.8261	NW_016587449.1	5097	5497	-	374
novel.4244	NW_016539563.1	1	827	-	669
novel.4245	NW_016539564.1	23603	25778	+	2105
novel.4246	NW_016539564.1	43753	44330	+	521
novel.4247	NW_016539564.1	42739	48948	-	247
novel.4240	NW_016539534.1	128753	186514	+	933
novel.4241	NW_016539549.1	132447	137848	-	2811
novel.4242	NW_016539550.1	122122	124378	-	655
novel.4243	NW_016539559.1	322011	326109	+	308
novel.3216	NW_016535849.1	106173	107484	+	1268
novel.3217	NW_016535849.1	113394	114355	+	889
novel.3214	NW_016535847.1	31340	33082	+	1498
novel.3215	NW_016535847.1	92024	98479	-	1889
novel.3212	NW_016535843.1	5295	7763	+	252
novel.3213	NW_016535846.1	139592	139819	-	207
novel.3210	NW_016535836.1	1	1130	+	1079
novel.3211	NW_016535836.1	1	1130	-	1053
novel.3218	NW_016535849.1	165	1088	-	924
novel.3219	NW_016535849.1	116412	120210	-	3094
novel.8831	NW_016607672.1	1	5446	+	5446

novel.3797	NW_016537806.1	123505	146195	+	632
novel.2035	NW_016532127.1	53295	53674	-	380
novel.2034	NW_016532127.1	46213	48011	+	1623
novel.2037	NW_016532134.1	11750	12467	+	331
novel.2036	NW_016532131.1	14266	16904	+	1108
novel.2031	NW_016532118.1	60609	62560	+	1852
novel.2030	NW_016532116.1	58027	58947	+	896
novel.2033	NW_016532127.1	43479	46090	+	2612
novel.2032	NW_016532127.1	270	12466	+	1618
novel.2039	NW_016532135.1	162487	163143	+	637
novel.2038	NW_016532134.1	7548	8333	-	786
novel.1104	NW_016529770.1	20634	21898	+	252
novel.1105	NW_016529770.1	27649	28018	+	282
novel.1106	NW_016529770.1	51684	52321	+	551
novel.1107	NW_016529770.1	25827	27429	-	1131
novel.1100	NW_016529750.1	17928	20027	-	696
novel.1101	NW_016529752.1	128572	129223	-	595
novel.1102	NW_016529768.1	181013	182171	+	1038
novel.1103	NW_016529768.1	181735	182171	-	437
novel.1108	NW_016529770.1	48216	49278	-	503
novel.1109	NW_016529772.1	74810	75446	+	637
novel.5460	NW_016545226.1	17600	18203	-	558
novel.2307	NW_016533005.1	103797	105229	-	1234
novel.2306	NW_016533005.1	58200	75648	-	406
novel.4888	NW_016542094.1	263714	264593	+	544
novel.4889	NW_016542094.1	272107	273689	-	1022
novel.2305	NW_016533005.1	204840	205152	+	261
novel.4882	NW_016542073.1	62991	64171	+	855
novel.4883	NW_016542073.1	64235	150326	+	479
novel.4880	NW_016542070.1	123524	131787	+	315
novel.4881	NW_016542070.1	129222	129977	+	708
novel.4886	NW_016542087.1	1879	8226	-	213
novel.4887	NW_016542093.1	29022	30318	-	1168
novel.4884	NW_016542073.1	18310	65068	-	1289
novel.4885	NW_016542074.1	3251	3652	+	353
novel.2757	NW_016534256.1	2684	5819	-	229
novel.2309	NW_016533016.1	113919	116095	+	1931
novel.2308	NW_016533016.1	4255	6769	+	2149
novel.388	NW_016528100.1	12805	13458	-	477
novel.389	NW_016528100.1	13241	13884	-	451
novel.386	NW_016528100.1	12687	13884	+	425
novel.387	NW_016528100.1	19042	54781	+	314
novel.384	NW_016528096.1	44659	45016	-	301
novel.385	NW_016528096.1	82934	83763	-	260

novel.382	NW_016528096.1	448	1409	+	910
novel.383	NW_016528096.1	37859	84360	-	1004
novel.380	NW_016528087.1	54790	60001	-	4051
novel.381	NW_016528092.1	78634	90072	-	251
novel.3364	NW_016536379.1	62820	67727	-	745
novel.4479	NW_016540541.1	9188	11625	-	2364
novel.4478	NW_016540541.1	6	12781	-	525
novel.4473	NW_016540509.1	400	779	-	380
novel.4472	NW_016540503.1	77804	79779	-	1976
novel.4471	NW_016540503.1	77804	79779	+	1976
novel.4470	NW_016540495.1	41420	41764	-	289
novel.4477	NW_016540540.1	1	4270	-	390
novel.4476	NW_016540535.1	655	27107	-	1254
novel.4475	NW_016540512.1	194934	195365	-	432
novel.4474	NW_016540509.1	3044	4066	-	335
novel.3653	NW_016537372.1	20026	81117	+	832
novel.6655	NW_016554153.1	8573	9178	-	606
novel.6654	NW_016554153.1	12112	14257	+	2146
novel.6657	NW_016554187.1	15793	16118	-	326
novel.6656	NW_016554159.1	169588	172011	-	2398
novel.6651	NW_016554135.1	9113	13595	+	1734
novel.6650	NW_016554135.1	105	536	+	392
novel.6653	NW_016554153.1	8574	9114	+	424
novel.6652	NW_016554153.1	7915	8308	+	348
novel.6659	NW_016554219.1	8277	8516	-	217
novel.6658	NW_016554219.1	9829	10106	+	246
novel.528	NW_016528420.1	420979	421399	-	312
novel.529	NW_016528422.1	6639	13713	+	207
novel.522	NW_016528416.1	10718	18613	+	1427
novel.523	NW_016528420.1	68513	69714	+	458
novel.520	NW_016528413.1	866	1994	+	805
novel.521	NW_016528413.1	13119	14595	+	1422
novel.526	NW_016528420.1	81291	408059	-	1791
novel.527	NW_016528420.1	292796	293817	-	694
novel.524	NW_016528420.1	114412	120021	+	344
novel.525	NW_016528420.1	239817	241801	+	244
novel.3363	NW_016536379.1	62820	63896	+	1045
novel.5308	NW_016544259.1	38555	39871	-	388
novel.5309	NW_016544294.1	15065	17093	-	983
novel.6909	NW_016556797.1	650	2470	-	822
novel.6908	NW_016556797.1	2825	4439	+	1615
novel.5302	NW_016544215.1	35609	36486	+	763
novel.5303	NW_016544215.1	35739	36495	-	699
novel.5300	NW_016544182.1	56534	65067	-	2166

novel.5301	NW_016544200.1	112416	114288	-	1461
novel.5306	NW_016544232.1	53893	64102	-	333
novel.5307	NW_016544259.1	7778	9213	+	1335
novel.5304	NW_016544229.1	38605	39129	-	525
novel.5305	NW_016544232.1	2960	3701	-	243
novel.2620	NW_016533880.1	25369	26207	+	783
novel.5901	NW_016548093.1	69386	69754	+	319
novel.2622	NW_016533897.1	30106	31380	-	1275
novel.2623	NW_016533910.1	222779	223143	-	342
novel.2624	NW_016533910.1	234306	252690	-	3458
novel.2625	NW_016533913.1	245211	306223	+	329
novel.2626	NW_016533917.1	87801	106215	+	285
novel.2627	NW_016533917.1	100708	102194	-	1348
novel.2628	NW_016533926.1	37	353	-	282
novel.2629	NW_016533935.1	205023	208897	+	837
novel.8503	NW_016598407.1	14	1497	+	1167
novel.8502	NW_016598306.1	1	1506	-	1506
novel.8501	NW_016598290.1	9	1009	-	882
novel.8500	NW_016598281.1	21	1488	+	1468
novel.7238	NW_016561229.1	7107	13321	+	506
novel.7239	NW_016561264.1	252	57927	-	1071
novel.8505	NW_016598614.1	1	1513	-	296
novel.8504	NW_016598548.1	451	793	+	224
novel.7234	NW_016561193.1	11974	14757	+	720
novel.7235	NW_016561213.1	101172	101886	+	413
novel.7236	NW_016561221.1	49530	49967	-	213
novel.7237	NW_016561225.1	7717	8155	+	210
novel.7230	NW_016561125.1	22811	23086	-	206
novel.7231	NW_016561125.1	35568	59847	-	8852
novel.7232	NW_016561127.1	121791	122219	-	305
novel.7233	NW_016561133.1	1443	1812	+	318
novel.2531	NW_016533675.1	242965	243728	+	764
novel.6628	NW_016553999.1	1	1001	+	961
novel.6629	NW_016553999.1	1	972	-	569
novel.2956	NW_016534990.1	8038	8315	-	222
novel.2957	NW_016534993.1	8919	9479	-	507
novel.2954	NW_016534971.1	654347	655785	-	1403
novel.2955	NW_016534989.1	22549	30222	+	760
novel.2952	NW_016534971.1	452666	459008	+	2246
novel.2953	NW_016534971.1	289181	290072	-	892
novel.2950	NW_016534971.1	308520	310322	+	384
novel.2483	NW_016533512.1	521	987	+	200
novel.2488	NW_016533527.1	677036	701241	-	216
novel.2489	NW_016533528.1	33761	40261	+	229

novel.2958	NW_016534998.1	18055	57487	-	537
novel.2959	NW_016535004.1	10999	11573	+	523
novel.3689	NW_016537494.1	253861	254957	+	270
novel.3688	NW_016537491.1	212243	212601	+	359
novel.3681	NW_016537466.1	85133	85847	+	506
novel.3680	NW_016537462.1	15463	17632	+	1020
novel.3683	NW_016537470.1	251833	265939	-	659
novel.3682	NW_016537469.1	3348	11937	-	507
novel.3685	NW_016537477.1	80305	81079	-	573
novel.3684	NW_016537471.1	76801	79392	-	2592
novel.3687	NW_016537481.1	6775	7269	+	495
novel.3686	NW_016537478.1	50547	56203	+	300
novel.6622	NW_016553886.1	400	3918	+	3126
novel.6623	NW_016553886.1	172	741	-	217
novel.7159	NW_016559733.1	104966	105491	-	526
novel.7158	NW_016559733.1	177271	178268	+	998
novel.3533	NW_016537023.1	24872	25149	-	252
novel.3532	NW_016537023.1	18780	22135	-	3301
novel.3531	NW_016537023.1	13962	15041	-	1031
novel.3530	NW_016537023.1	23844	24102	+	234
novel.3537	NW_016537049.1	105825	108781	+	1284
novel.7409	NW_016564073.1	129396	130129	-	453
novel.3535	NW_016537024.1	244583	279216	+	504
novel.3534	NW_016537023.1	31642	32606	-	916
novel.3539	NW_016537050.1	22110	22824	-	337
novel.7408	NW_016564073.1	27050	27509	-	413
novel.7407	NW_016564073.1	26374	26978	-	605
novel.7406	NW_016564073.1	95806	107202	+	1757
novel.7157	NW_016559733.1	163488	164634	+	1147
novel.7156	NW_016559733.1	104966	105491	+	526
novel.7403	NW_016564039.1	82241	85989	-	1964
novel.7402	NW_016564039.1	27965	31981	-	3766
novel.7401	NW_016564039.1	26101	27073	-	916
novel.6338	NW_016551113.1	236751	241748	-	2760
novel.6339	NW_016551128.1	5960	23793	-	292
novel.6336	NW_016551108.1	72377	73948	+	455
novel.6337	NW_016551113.1	236751	241014	+	2894
novel.6334	NW_016551103.1	8001	9338	+	1294
novel.6335	NW_016551103.1	7595	7902	-	267
novel.6332	NW_016551084.1	12537	36187	-	241
novel.6333	NW_016551092.1	58462	58720	-	204
novel.6330	NW_016551043.1	5009	7496	+	1747
novel.6331	NW_016551052.1	74036	92713	-	863
novel.3134	NW_016535625.1	8	1133	-	898

novel.6480	NW_016552482.1	180109	182180	+	584
novel.6481	NW_016552482.1	102889	103348	-	460
novel.6482	NW_016552493.1	11850	13680	-	622
novel.6483	NW_016552509.1	36563	36872	+	262
novel.6484	NW_016552527.1	12681	13455	-	775
novel.6485	NW_016552556.1	79747	91901	+	459
novel.6486	NW_016552595.1	13911	43069	-	1129
novel.6487	NW_016552604.1	77585	78356	-	715
novel.6488	NW_016552617.1	3888	5848	-	268
novel.6489	NW_016552630.1	682	19281	+	815
novel.8350	NW_016591097.1	13091	17874	+	859
novel.99	NW_016527462.1	26726	27650	-	387
novel.98	NW_016527460.1	16955	17286	-	332
novel.97	NW_016527454.1	71094	72424	+	1331
novel.96	NW_016527453.1	38	1328	-	1265
novel.95	NW_016527451.1	22457	41629	-	257
novel.94	NW_016527451.1	43984	44711	+	728
novel.93	NW_016527450.1	55766	57196	+	1374
novel.92	NW_016527435.1	261847	263080	+	288
novel.91	NW_016527432.1	254276	254709	-	382
novel.90	NW_016527432.1	252920	253231	-	256
novel.4194	NW_016539325.1	48814	54906	-	413
novel.4195	NW_016539332.1	262896	263282	+	387
novel.1599	NW_016530902.1	128638	149535	-	767
novel.1598	NW_016530902.1	63342	63719	-	323
novel.1597	NW_016530901.1	1	712	+	672
novel.1596	NW_016530893.1	49220	50336	+	1117
novel.1595	NW_016530893.1	33882	34439	+	227
novel.1594	NW_016530862.1	566207	569061	-	812
novel.1593	NW_016530862.1	327930	328620	-	635
novel.1592	NW_016530862.1	142943	152802	-	2547
novel.1591	NW_016530862.1	537000	537991	+	554
novel.1590	NW_016530862.1	534584	536910	+	788
novel.5070	NW_016542891.1	121745	124355	+	2549
novel.4192	NW_016539320.1	363334	363680	+	347
novel.5689	NW_016546582.1	8951	9568	+	618
novel.5071	NW_016542909.1	20759	25354	+	1208
novel.5687	NW_016546508.1	257	3182	-	2926
novel.5686	NW_016546508.1	91933	111735	+	1129
novel.5685	NW_016546508.1	47339	49279	+	1941
novel.5684	NW_016546506.1	330937	335728	-	2726
novel.5683	NW_016546483.1	54866	56571	-	556
novel.5682	NW_016546479.1	9519	10343	-	768
novel.5681	NW_016546479.1	8681	9506	-	826

novel.5680	NW_016546479.1	2556	4036	-	1118
novel.7829	NW_016573294.1	1	2661	-	831
novel.7828	NW_016573294.1	11289	11649	+	309
novel.6788	NW_016555462.1	797	1424	+	574
novel.8397	NW_016591916.1	15822	22950	-	907
novel.7821	NW_016573151.1	3580	4460	+	881
novel.7820	NW_016573124.1	1	5772	+	771
novel.7823	NW_016573178.1	90894	92059	-	619
novel.7822	NW_016573151.1	3451	4852	-	1235
novel.7825	NW_016573203.1	24	11106	-	467
novel.7824	NW_016573203.1	2575	11150	+	8538
novel.7827	NW_016573294.1	7244	7686	+	443
novel.7826	NW_016573203.1	2098	2439	-	285
novel.8395	NW_016591904.1	3601	4940	+	223
novel.8392	NW_016591868.1	1	1270	-	341
novel.8393	NW_016591888.1	45452	47647	-	874
novel.1373	NW_016530338.1	2480	31671	-	992
novel.1372	NW_016530334.1	256062	257786	-	417
novel.1371	NW_016530334.1	251095	252667	-	471
novel.1370	NW_016530334.1	100578	104451	+	1749
novel.1377	NW_016530343.1	144359	152397	+	2393
novel.1376	NW_016530341.1	1	303	+	303
novel.1375	NW_016530340.1	6245	76488	-	971
novel.1374	NW_016530338.1	117830	121039	-	377
novel.1379	NW_016530343.1	1031654	1033661	+	560
novel.1378	NW_016530343.1	368198	369943	+	839
novel.6780	NW_016555353.1	8120	8393	+	220
novel.737	NW_016528877.1	98809	99341	+	323
novel.736	NW_016528869.1	182496	184176	-	354
novel.735	NW_016528869.1	88889	93642	-	4514
novel.734	NW_016528867.1	443170	443538	+	369
novel.733	NW_016528864.1	211267	211903	+	242
novel.732	NW_016528864.1	74476	76727	+	2145
novel.731	NW_016528861.1	77923	78316	-	255
novel.730	NW_016528861.1	226695	237587	+	741
novel.739	NW_016528879.1	29721	30275	+	260
novel.738	NW_016528877.1	98809	99341	-	266
novel.1717	NW_016531278.1	220246	221489	-	392
novel.3054	NW_016535421.1	40243	42131	-	1836
novel.3055	NW_016535424.1	84174	92464	+	301
novel.3056	NW_016535429.1	152175	152630	+	325
novel.3057	NW_016535429.1	46325	46723	-	399
novel.3050	NW_016535412.1	40202	41095	+	894
novel.3051	NW_016535414.1	66969	70214	+	3246

novel.3052	NW_016535415.1	26471	30133	+	1921
novel.3053	NW_016535421.1	28616	29135	+	477
novel.3058	NW_016535429.1	489816	491014	-	249
novel.3059	NW_016535429.1	500125	500958	-	798
novel.4226	NW_016539474.1	17518	20477	+	336
novel.4227	NW_016539474.1	16977	19807	-	240
novel.4224	NW_016539449.1	29289	29785	-	440
novel.4225	NW_016539464.1	93201	116995	+	956
novel.4222	NW_016539438.1	492264	493627	+	1252
novel.4223	NW_016539449.1	24964	28432	+	382
novel.4220	NW_016539417.1	4821	5066	+	221
novel.4221	NW_016539435.1	171762	173999	-	1828
novel.8756	NW_016606334.1	567	2889	-	386
novel.8757	NW_016606342.1	3657	5071	-	497
novel.8754	NW_016606292.1	294	3202	+	206
novel.8755	NW_016606331.1	1	1269	+	1269
novel.8752	NW_016606235.1	4419	5500	-	768
novel.8753	NW_016606240.1	2454	3836	-	463
novel.4228	NW_016539498.1	349133	350083	-	516
novel.4229	NW_016539503.1	96574	98355	-	1782
novel.3238	NW_016535914.1	7296	7799	+	420
novel.3239	NW_016535914.1	119287	120311	-	927
novel.3948	NW_016538342.1	258440	260062	-	401
novel.3949	NW_016538342.1	425882	426878	-	997
novel.3942	NW_016538337.1	13089	13690	-	602
novel.3943	NW_016538337.1	17527	18814	-	1288
novel.3940	NW_016538335.1	185569	186471	-	705
novel.3941	NW_016538337.1	13089	14664	+	1157
novel.3946	NW_016538342.1	327218	330774	+	2952
novel.3947	NW_016538342.1	134712	136546	-	1835
novel.3944	NW_016538342.1	218497	220261	+	228
novel.3945	NW_016538342.1	310665	313194	+	271
novel.8204	NW_016586151.1	936	1876	-	941
novel.2549	NW_016533717.1	11614	12414	-	666
novel.2548	NW_016533715.1	111966	112168	-	203
novel.2019	NW_016532068.1	106217	106617	+	368
novel.2018	NW_016532067.1	55181	60220	+	263
novel.2541	NW_016533696.1	71081	74573	-	1119
novel.2540	NW_016533693.1	114805	116403	-	1599
novel.2543	NW_016533706.1	26585	27256	-	218
novel.2014	NW_016532061.1	9426	10315	-	272
novel.2545	NW_016533711.1	171006	177611	+	629
novel.2544	NW_016533711.1	139131	140586	+	827
novel.2547	NW_016533714.1	82740	86003	-	3210

novel.2546	NW_016533711.1	134599	141748	-	786
novel.3679	NW_016537458.1	63742	66336	-	520
novel.8206	NW_016586202.1	269629	271169	+	331
novel.6169	NW_016550000.1	4616	6117	-	1447
novel.297	NW_016527945.1	107306	107721	+	416
novel.8202	NW_016586149.1	15	997	-	941
novel.2637	NW_016533948.1	50931	51507	+	550
novel.8203	NW_016586151.1	5	3984	+	2906
novel.2679	NW_016534060.1	88382	88953	+	519
novel.220	NW_016527726.1	131177	278168	-	396
novel.2678	NW_016534060.1	36	549	+	461
novel.2636	NW_016533946.1	14749	15077	+	329
novel.2673	NW_016534046.1	17727	18388	-	519
novel.2635	NW_016533945.1	44789	45318	-	475
novel.1168	NW_016529877.1	97282	97880	+	377
novel.1169	NW_016529888.1	365303	365855	+	407
novel.1166	NW_016529874.1	580620	585598	+	228
novel.1167	NW_016529874.1	589093	595798	+	381
novel.1164	NW_016529871.1	123407	124342	-	830
novel.1165	NW_016529874.1	544454	548308	+	947
novel.1162	NW_016529870.1	73780	74415	-	636
novel.1163	NW_016529870.1	74708	74984	-	277
novel.1160	NW_016529869.1	617	1346	-	675
novel.1161	NW_016529869.1	6701	7009	-	309
novel.2246	NW_016532740.1	120664	123354	-	2640
novel.2670	NW_016534039.1	253019	254396	-	1186
novel.2247	NW_016532753.1	44490	49140	+	306
novel.2677	NW_016534057.1	10435	11023	+	532
novel.5781	NW_016547135.1	464039	467088	+	670
novel.5564	NW_016545768.1	102815	111210	-	337
novel.2676	NW_016534053.1	219082	219881	-	335
novel.5565	NW_016545787.1	81497	92213	-	447
novel.4419	NW_016540298.1	140712	140976	+	235
novel.4418	NW_016540291.1	39472	40194	+	388
novel.4415	NW_016540280.1	95445	96695	+	1251
novel.2242	NW_016532713.1	5323	8801	-	3367
novel.4417	NW_016540286.1	1	1366	-	1342
novel.4416	NW_016540283.1	207771	222158	+	1114
novel.4411	NW_016540237.1	20778	21767	+	947
novel.4410	NW_016540216.1	32451	33273	-	704
novel.4413	NW_016540266.1	73431	84156	-	404
novel.2243	NW_016532733.1	12729	13089	+	336
novel.2240	NW_016532708.1	8365	10390	-	504
novel.6679	NW_016554389.1	4446	4994	-	493

novel.6678	NW_016554383.1	4941	5284	+	288
novel.6677	NW_016554375.1	31276	32213	-	705
novel.6676	NW_016554375.1	30373	31240	-	868
novel.6675	NW_016554342.1	22000	25962	-	1464
novel.2241	NW_016532713.1	5331	8788	+	3401
novel.6673	NW_016554342.1	1505	3093	+	699
novel.6672	NW_016554341.1	117689	118081	-	393
novel.6671	NW_016554321.1	1217	1702	+	430
novel.6670	NW_016554319.1	1	288	+	288
novel.500	NW_016528366.1	488093	489403	-	1311
novel.501	NW_016528368.1	110854	112312	+	384
novel.502	NW_016528368.1	355715	356995	+	1037
novel.503	NW_016528368.1	258343	259938	-	1474
novel.504	NW_016528370.1	86404	87065	-	468
novel.505	NW_016528372.1	73669	74668	+	299
novel.506	NW_016528382.1	289218	289685	+	468
novel.507	NW_016528382.1	586632	587433	+	406
novel.508	NW_016528382.1	611442	613361	+	597
novel.509	NW_016528382.1	613462	620048	+	461
novel.5568	NW_016545820.1	7361	7701	+	303
novel.2249	NW_016532767.1	506894	508670	-	978
novel.1788	NW_016531444.1	27612	28009	-	398
novel.1789	NW_016531444.1	44240	45064	-	212
novel.8189	NW_016585484.1	2409	4322	+	1862
novel.1782	NW_016531420.1	40633	41589	-	444
novel.1783	NW_016531420.1	147613	150998	-	3114
novel.1780	NW_016531418.1	12295	64617	-	849
novel.7329	NW_016562807.1	54068	54957	+	852
novel.1786	NW_016531441.1	98692	99569	+	523
novel.1787	NW_016531444.1	173410	174181	+	723
novel.1784	NW_016531420.1	378934	388852	-	1000
novel.1785	NW_016531429.1	41186	42015	-	318
novel.8187	NW_016585438.1	1	596	-	546
novel.5098	NW_016543003.1	165395	167130	-	1690
novel.5099	NW_016543015.1	279851	281024	-	680
novel.7327	NW_016562737.1	29824	31134	+	230
novel.5092	NW_016542996.1	54269	62314	+	680
novel.5093	NW_016542998.1	22057	22372	-	263
novel.5090	NW_016542996.1	13676	13992	+	265
novel.5091	NW_016542996.1	34010	35176	+	1143
novel.5096	NW_016543002.1	311883	315150	-	648
novel.5097	NW_016543003.1	164521	168213	+	1853
novel.5094	NW_016543000.1	421377	421579	+	203
novel.5095	NW_016543002.1	218444	228075	+	614

novel.8184	NW_016585408.1	19055	22639	-	929
novel.8183	NW_016585408.1	5082	18187	-	574
novel.7323	NW_016562707.1	58165	58664	-	448
novel.7032	NW_016557978.1	1	733	+	733
novel.7033	NW_016557980.1	30807	32087	-	962
novel.7030	NW_016557970.1	18146	19693	+	885
novel.7031	NW_016557970.1	8358	10287	-	1877
novel.7036	NW_016558067.1	40873	41340	-	468
novel.7037	NW_016558071.1	1815	2241	+	222
novel.7034	NW_016558050.1	18930	19426	+	287
novel.7035	NW_016558063.1	4687	5133	-	390
novel.7784	NW_016572217.1	1	231	+	231
novel.7785	NW_016572220.1	4089	4369	+	228
novel.7786	NW_016572304.1	18077	23871	-	5795
novel.7039	NW_016558083.1	3756	4315	+	509
novel.7780	NW_016572144.1	52051	56772	-	2093
novel.7781	NW_016572193.1	5821	14214	+	2095
novel.7782	NW_016572193.1	12254	14214	-	1961
novel.7783	NW_016572204.1	3485	3850	-	366
novel.6927	NW_016556918.1	6308	7772	+	1021
novel.6926	NW_016556917.1	24938	26076	-	1029
novel.6925	NW_016556892.1	26328	26810	-	212
novel.6924	NW_016556884.1	5233	5554	-	297
novel.6923	NW_016556864.1	35669	36060	-	336
novel.5329	NW_016544397.1	1	675	-	618
novel.6921	NW_016556851.1	21560	26954	+	288
novel.6920	NW_016556848.1	4536	6071	-	947
novel.5324	NW_016544383.1	173718	174577	+	312
novel.5325	NW_016544387.1	51499	52364	+	866
novel.5326	NW_016544387.1	8020	8294	-	220
novel.5327	NW_016544387.1	43259	44686	-	1428
novel.5320	NW_016544326.1	47543	48561	+	299
novel.5321	NW_016544329.1	15682	21290	-	274
novel.6929	NW_016556926.1	32049	39115	+	7067
novel.6928	NW_016556918.1	83505	85640	+	1258
novel.8565	NW_016601221.1	178	1947	+	1770
novel.8564	NW_016601206.1	73	2002	-	1146
novel.8567	NW_016601301.1	1	2027	+	2027
novel.8566	NW_016601238.1	1	2011	+	534
novel.8561	NW_016601139.1	83	1927	+	354
novel.8560	NW_016601109.1	266	1975	-	1130
novel.8563	NW_016601176.1	1	1995	-	265
novel.7251	NW_016561476.1	4514	6062	+	1488
novel.8569	NW_016601367.1	1	2038	-	318

novel.8568	NW_016601366.1	219	2037	+	1819
novel.7258	NW_016561585.1	98874	100303	-	536
novel.7259	NW_016561588.1	10651	18906	+	777
novel.1943	NW_016531840.1	261440	261897	+	297
novel.1944	NW_016531847.1	147079	147485	-	317
novel.1945	NW_016531849.1	621901	626725	+	2823
novel.1946	NW_016531858.1	19365	31529	+	695
novel.1947	NW_016531868.1	26505	27210	-	706
novel.5462	NW_016545255.1	4225	5910	+	1635
novel.4304	NW_016539832.1	134175	134509	+	335
novel.8327	NW_016590167.1	7718	8240	-	255
novel.5461	NW_016545241.1	31686	99370	-	224
novel.8326	NW_016590167.1	7000	7612	+	613
novel.2639	NW_016533948.1	114227	114884	+	321
novel.6075	NW_016549407.1	3618	4333	+	667
novel.6074	NW_016549402.1	39420	39799	+	330
novel.6077	NW_016549422.1	11232	12105	-	874
novel.6076	NW_016549417.1	95677	97792	-	2116
novel.6071	NW_016549396.1	49286	50488	+	1203
novel.6070	NW_016549396.1	38811	48860	+	669
novel.6073	NW_016549402.1	39014	39365	+	352
novel.6072	NW_016549396.1	198051	202522	+	806
novel.6079	NW_016549422.1	17026	17306	-	281
novel.6078	NW_016549422.1	12156	16739	-	3464
novel.2978	NW_016535121.1	144436	144745	+	310
novel.2979	NW_016535133.1	73672	77198	-	294
novel.5484	NW_016545368.1	57838	59026	-	1082
novel.2970	NW_016535044.1	7734	57187	+	939
novel.2971	NW_016535063.1	4632	20454	-	1437
novel.2972	NW_016535084.1	178236	182883	+	3081
novel.2973	NW_016535085.1	300109	300549	-	331
novel.2974	NW_016535093.1	44684	55812	-	4620
novel.2975	NW_016535093.1	56703	60570	-	244
novel.2976	NW_016535093.1	63637	74345	-	834
novel.2977	NW_016535103.1	133737	134098	+	311
novel.2454	NW_016533421.1	218	806	+	589
novel.4789	NW_016541717.1	15063	16225	+	1163
novel.4788	NW_016541691.1	135	1081	-	947
novel.4785	NW_016541685.1	8767	10070	-	245
novel.4784	NW_016541681.1	23450	23665	+	216
novel.4787	NW_016541691.1	901	1335	+	296
novel.4786	NW_016541690.1	15273	15666	-	239
novel.4781	NW_016541675.1	8841	10228	+	681
novel.4780	NW_016541648.1	120985	122244	-	297

novel.4783	NW_016541679.1	6635	7162	+	528
novel.4782	NW_016541678.1	13363	14108	-	414
novel.6310	NW_016550901.1	55394	56101	+	708
novel.6311	NW_016550901.1	56334	56654	+	273
novel.6312	NW_016550901.1	60821	61724	+	793
novel.6313	NW_016550901.1	13563	28929	-	263
novel.6314	NW_016550901.1	58777	59082	-	250
novel.6315	NW_016550908.1	131399	132954	+	217
novel.6316	NW_016550933.1	5678	8202	-	532
novel.6317	NW_016550942.1	34792	47443	-	556
novel.6318	NW_016550958.1	177	2631	+	799
novel.6319	NW_016550959.1	143167	146009	+	967
novel.5482	NW_016545364.1	58017	58545	+	249
novel.616	NW_016528620.1	24067	24849	+	581
novel.617	NW_016528627.1	120443	121037	-	595
novel.610	NW_016528581.1	1	1045	+	992
novel.611	NW_016528583.1	69411	73324	+	927
novel.612	NW_016528596.1	37687	38255	+	569
novel.613	NW_016528601.1	356818	357611	-	428
novel.7842	NW_016573779.1	1	1361	-	1316
novel.5528	NW_016545566.1	453431	454033	+	422
novel.2209	NW_016532623.1	181995	182769	+	243
novel.5522	NW_016545551.1	62182	62470	+	245
novel.5523	NW_016545565.1	64278	65516	+	1171
novel.5520	NW_016545535.1	661	3349	-	600
novel.5521	NW_016545541.1	5200	6056	-	814
novel.2206	NW_016532605.1	125236	125541	+	250
novel.2207	NW_016532607.1	199881	200177	-	275
novel.5524	NW_016545566.1	17632	18541	+	910
novel.5525	NW_016545566.1	73792	84183	+	387
novel.1571	NW_016530815.1	41237	43830	-	2311
novel.1570	NW_016530807.1	74488	76945	+	618
novel.1573	NW_016530821.1	75000	75670	+	589
novel.1572	NW_016530817.1	109596	111560	-	1923
novel.1575	NW_016530824.1	31134	34736	-	3603
novel.1574	NW_016530824.1	15542	34736	+	3738
novel.1577	NW_016530830.1	77512	78005	+	258
novel.1576	NW_016530828.1	544325	544538	-	214
novel.1579	NW_016530832.1	147423	147974	+	552
novel.1578	NW_016530832.1	28864	172729	+	3586
novel.5661	NW_016546378.1	5339	13804	+	6148
novel.5660	NW_016546374.1	20616	62838	+	1067
novel.5663	NW_016546387.1	38080	66829	-	502
novel.5662	NW_016546387.1	22965	33911	-	684

novel.5665	NW_016546410.1	23668	38853	+	1321
novel.5664	NW_016546401.1	24265	25945	+	1575
novel.5667	NW_016546410.1	632319	633048	-	474
novel.5666	NW_016546410.1	137024	138662	-	437
novel.5669	NW_016546414.1	173509	178252	-	1587
novel.5668	NW_016546414.1	178272	197769	+	506
novel.7803	NW_016572752.1	7285	8812	-	1188
novel.7802	NW_016572752.1	7839	8812	+	974
novel.7801	NW_016572713.1	3408	5193	+	254
novel.7800	NW_016572704.1	45607	109187	+	5692
novel.7807	NW_016572822.1	1	279	-	237
novel.7806	NW_016572800.1	5287	5558	+	221
novel.7805	NW_016572791.1	7414	12675	-	744
novel.7804	NW_016572781.1	4005	24908	-	1308
novel.7809	NW_016572863.1	83861	86433	+	515
novel.7808	NW_016572863.1	58378	59657	+	1223
novel.1315	NW_016530185.1	47592	47870	+	279
novel.1314	NW_016530184.1	681645	682086	-	388
novel.1317	NW_016530193.1	7969	55640	-	406
novel.1316	NW_016530185.1	181968	182694	+	372
novel.1311	NW_016530181.1	239685	253185	+	490
novel.1310	NW_016530169.1	154386	155098	+	540
novel.1313	NW_016530182.1	1026797	1030615	+	409
novel.1312	NW_016530181.1	221837	222920	-	494
novel.1319	NW_016530196.1	77928	84092	+	827
novel.1318	NW_016530195.1	91518	100367	+	740
novel.759	NW_016528952.1	4297	4579	-	227
novel.758	NW_016528952.1	1116	1674	-	374
novel.755	NW_016528949.1	168766	177209	+	6494
novel.754	NW_016528941.1	12623	13755	+	1133
novel.757	NW_016528950.1	556731	557528	-	743
novel.756	NW_016528949.1	170518	175835	-	5318
novel.751	NW_016528919.1	75571	79746	-	328
novel.750	NW_016528914.1	272338	274923	-	1345
novel.753	NW_016528932.1	104736	106496	+	314
novel.752	NW_016528921.1	172060	172765	-	490
novel.3038	NW_016535388.1	27106	29695	-	2503
novel.3039	NW_016535388.1	37463	38493	-	997
novel.3036	NW_016535381.1	44211	44980	-	663
novel.3037	NW_016535388.1	27106	28618	+	1483
novel.3034	NW_016535366.1	97042	118156	+	521
novel.3035	NW_016535380.1	152018	153321	-	1108
novel.3032	NW_016535347.1	37351	38526	-	1031
novel.3033	NW_016535364.1	79834	84427	+	4594

novel.3030	NW_016535321.1	1677	1927	+	251
novel.3031	NW_016535329.1	48137	55768	+	233
novel.6602	NW_016553636.1	27899	33193	+	338
novel.4208	NW_016539365.1	6920	8572	+	286
novel.4209	NW_016539365.1	41069	42956	+	252
novel.4200	NW_016539336.1	10021	16231	-	1421
novel.4201	NW_016539336.1	191889	192461	-	358
novel.4202	NW_016539342.1	5913	6508	+	596
novel.4203	NW_016539342.1	1885	8233	-	3375
novel.4204	NW_016539347.1	6859	7599	-	617
novel.4205	NW_016539352.1	6602	43332	-	311
novel.4206	NW_016539356.1	1	10734	-	655
novel.4207	NW_016539357.1	17007	23582	-	287
novel.8770	NW_016606648.1	5512	6488	+	977
novel.8771	NW_016606680.1	300	1839	+	229
novel.8772	NW_016606727.1	22	2198	+	1521
novel.8773	NW_016606727.1	19	6688	-	6670
novel.8774	NW_016606767.1	3211	6120	+	232
novel.8775	NW_016606780.1	1465	5918	-	316
novel.8776	NW_016606782.1	334	1400	-	1067
novel.8777	NW_016606826.1	1167	6358	-	529
novel.8778	NW_016606879.1	1573	3289	+	425
novel.8779	NW_016606903.1	5196	7071	+	1581
novel.3780	NW_016537760.1	228906	230143	-	1238
novel.3781	NW_016537764.1	80757	81109	+	353
novel.3782	NW_016537766.1	115906	116348	-	391
novel.3783	NW_016537772.1	26567	29191	-	282
novel.3968	NW_016538383.1	219721	220039	-	231
novel.3969	NW_016538387.1	75341	102470	+	5534
novel.3786	NW_016537777.1	41114	98497	-	327
novel.3787	NW_016537779.1	3523	4344	+	788
novel.3964	NW_016538371.1	299084	300709	-	877
novel.3965	NW_016538383.1	5208	5629	-	255
novel.3966	NW_016538383.1	217800	220119	-	2320
novel.3967	NW_016538383.1	218301	219661	-	1078
novel.3960	NW_016538369.1	1715	2466	+	710
novel.3961	NW_016538371.1	90828	92420	-	1423
novel.3962	NW_016538371.1	111053	114458	-	1859
novel.3963	NW_016538371.1	298569	298991	-	423
novel.5807	NW_016547412.1	2651	11636	-	678
novel.2789	NW_016534323.1	274603	276252	+	1599
novel.2788	NW_016534323.1	86578	102685	+	538
novel.2787	NW_016534323.1	23279	25705	+	2374
novel.2786	NW_016534319.1	22420	23211	-	590

novel.2785	NW_016534319.1	345584	363662	+	743
novel.2784	NW_016534313.1	134307	134668	+	226
novel.2783	NW_016534309.1	120209	122612	+	238
novel.2782	NW_016534307.1	114293	121783	-	4810
novel.2781	NW_016534300.1	437861	443291	-	3232
novel.2780	NW_016534300.1	432747	434801	-	635
novel.6603	NW_016553650.1	159752	161514	-	1575
novel.2711	NW_016534147.1	5879	6613	-	636
novel.3508	NW_016536899.1	995	9118	-	1420
novel.2563	NW_016533739.1	357863	362327	-	1549
novel.2562	NW_016533735.1	2630	5792	-	1887
novel.2561	NW_016533731.1	17806	20972	-	1121
novel.2560	NW_016533729.1	336	15133	+	2350
novel.2567	NW_016533757.1	56853	59687	-	217
novel.2566	NW_016533742.1	45006	45728	-	447
novel.2565	NW_016533742.1	199975	200179	+	205
novel.2564	NW_016533742.1	181812	184098	+	867
novel.2569	NW_016533765.1	4569	5036	+	426
novel.2568	NW_016533763.1	17	783	-	619
novel.3502	NW_016536883.1	94476	95217	+	348
novel.3503	NW_016536883.1	314853	315264	+	273
novel.3500	NW_016536852.1	10566	10890	-	269
novel.5806	NW_016547408.1	242457	248645	-	970
novel.3501	NW_016536867.1	175337	183880	+	2556
novel.3506	NW_016536888.1	7987	16486	-	370
novel.3507	NW_016536889.1	33153	37213	-	1266
novel.3504	NW_016536883.1	313728	314147	-	316
novel.3505	NW_016536888.1	6964	7799	-	836
novel.3657	NW_016537382.1	127199	130502	+	1691
novel.6142	NW_016549786.1	27038	28253	-	255
novel.4129	NW_016539067.1	100580	102922	+	1956
novel.6145	NW_016549817.1	64069	68395	-	4276
novel.4128	NW_016539061.1	23191	23452	+	219
novel.1140	NW_016529851.1	25763	26567	-	466
novel.1141	NW_016529851.1	27836	29562	-	1624
novel.1142	NW_016529851.1	29662	30743	-	1016
novel.1143	NW_016529855.1	800006	805987	+	5982
novel.1144	NW_016529855.1	1436583	1446657	+	1645
novel.1145	NW_016529855.1	1636689	1638204	+	477
novel.1146	NW_016529855.1	1655431	1666084	+	3948
novel.1147	NW_016529855.1	257909	259889	-	399
novel.1148	NW_016529855.1	304958	350433	-	242
novel.1149	NW_016529855.1	357621	357946	-	326
novel.6144	NW_016549817.1	63112	63831	-	720

novel.4437	NW_016540353.1	50562	58877	+	1583
novel.4436	NW_016540353.1	3462	5434	+	1917
novel.4435	NW_016540350.1	26903	30510	-	687
novel.4434	NW_016540350.1	27405	28096	+	692
novel.4433	NW_016540344.1	241077	248943	-	518
novel.4432	NW_016540344.1	241077	248943	+	686
novel.4431	NW_016540341.1	184240	185898	-	497
novel.4120	NW_016538990.1	104276	104539	+	264
novel.4439	NW_016540357.1	97157	98220	-	1029
novel.4438	NW_016540353.1	3462	5318	-	1810
novel.6691	NW_016554579.1	103330	111384	-	1036
novel.6690	NW_016554569.1	87168	87448	+	281
novel.6693	NW_016554590.1	285	1270	+	678
novel.6692	NW_016554582.1	2	604	+	557
novel.6695	NW_016554619.1	17991	18598	+	551
novel.6694	NW_016554606.1	6103	7108	+	318
novel.6697	NW_016554619.1	47837	49070	-	1130
novel.6696	NW_016554619.1	60911	61135	+	225
novel.6699	NW_016554642.1	251333	254718	-	1158
novel.6698	NW_016554629.1	1471	1792	+	268
novel.3651	NW_016537368.1	82574	83342	+	769
novel.566	NW_016528480.1	47191	48067	-	370
novel.567	NW_016528482.1	166889	167133	-	245
novel.564	NW_016528479.1	4863	5160	-	278
novel.565	NW_016528480.1	48142	49912	+	465
novel.562	NW_016528476.1	4687	5303	-	572
novel.563	NW_016528476.1	7052	9107	-	445
novel.560	NW_016528473.1	8587	9576	+	344
novel.561	NW_016528473.1	11757	13071	-	423
novel.568	NW_016528482.1	167277	167696	-	206
novel.569	NW_016528484.1	158699	181079	-	549
novel.8095	NW_016582079.1	1	1965	+	613
novel.8094	NW_016582067.1	1508	2344	-	788
novel.7016	NW_016557886.1	50880	57805	+	4665
novel.7017	NW_016557886.1	93561	95832	+	2272
novel.8091	NW_016581903.1	1	746	-	692
novel.8090	NW_016581900.1	26	5425	+	309
novel.8093	NW_016581972.1	642	1448	-	767
novel.8092	NW_016581945.1	25	968	-	340
novel.8099	NW_016582249.1	1	874	+	821
novel.8098	NW_016582221.1	514	1006	-	493
novel.3245	NW_016535932.1	7498	8688	+	562
novel.5346	NW_016544496.1	685792	686834	+	1043
novel.5347	NW_016544496.1	686191	688486	-	377

novel.5344	NW_016544495.1	61083	61538	+	456
novel.5345	NW_016544495.1	61608	62682	+	1043
novel.5342	NW_016544482.1	153	768	-	243
novel.5343	NW_016544493.1	3273	5901	-	206
novel.5340	NW_016544481.1	3784	4370	+	533
novel.5341	NW_016544481.1	3691	4370	-	623
novel.5348	NW_016544510.1	22374	23574	-	1155
novel.5349	NW_016544533.1	5181	5460	+	224
novel.8239	NW_016587007.1	16095	17181	+	218
novel.7279	NW_016562059.1	107891	113061	-	4972
novel.3244	NW_016535921.1	777516	778473	-	958
novel.8549	NW_016600498.1	697	1113	-	340
novel.8548	NW_016600486.1	11	1089	+	861
novel.8547	NW_016600429.1	1	1342	+	395
novel.8230	NW_016586846.1	284	2144	+	1821
novel.8233	NW_016586873.1	3298	7423	+	4072
novel.8232	NW_016586873.1	2621	3152	+	483
novel.8543	NW_016600324.1	74	806	-	427
novel.8234	NW_016586873.1	2621	3684	-	952
novel.8237	NW_016586884.1	3482	7477	-	3939
novel.8236	NW_016586884.1	4602	5409	+	753
novel.8046	NW_016580293.1	31355	32622	+	1268
novel.8047	NW_016580308.1	8795	9058	+	209
novel.8048	NW_016580353.1	1007	1395	-	389
novel.984	NW_016529524.1	87805	88470	-	318
novel.985	NW_016529528.1	44759	47147	-	2389
novel.986	NW_016529531.1	360010	368773	+	507
novel.7066	NW_016558385.1	4679	5177	-	442
novel.980	NW_016529515.1	25894	49609	+	618
novel.981	NW_016529515.1	6046	18942	-	642
novel.982	NW_016529515.1	26116	27449	-	952
novel.983	NW_016529524.1	32275	42677	+	1522
novel.988	NW_016529532.1	308415	311687	-	208
novel.989	NW_016529535.1	90677	91024	-	211
novel.1650	NW_016531064.1	31897	32212	+	316
novel.1651	NW_016531064.1	23509	32212	-	2466
novel.1652	NW_016531072.1	55283	56850	-	1326
novel.1653	NW_016531092.1	83123	84017	-	703
novel.6059	NW_016549290.1	43874	44160	+	287
novel.6058	NW_016549290.1	38122	38549	+	344
novel.3577	NW_016537135.1	10151	11874	-	1724
novel.3576	NW_016537133.1	237420	238826	-	1379
novel.3575	NW_016537133.1	237420	238464	+	1045
novel.6054	NW_016549284.1	79441	99861	+	444

novel.6053	NW_016549284.1	77851	93128	+	1100
novel.6052	NW_016549268.1	49189	62380	+	1608
novel.6051	NW_016549264.1	5657	6018	+	264
novel.3570	NW_016537131.1	145128	146355	-	944
novel.1656	NW_016531124.1	105529	106025	-	497
novel.1657	NW_016531126.1	78974	80105	+	309
novel.2992	NW_016535168.1	92106	92572	+	467
novel.2993	NW_016535169.1	475	872	+	398
novel.2990	NW_016535165.1	18211	20570	+	1217
novel.2991	NW_016535165.1	12966	13546	-	498
novel.2996	NW_016535169.1	81892	82322	-	403
novel.2997	NW_016535169.1	112466	115633	-	3062
novel.2994	NW_016535169.1	112989	115633	+	2595
novel.2995	NW_016535169.1	67360	67815	-	399
novel.2998	NW_016535184.1	25408	36324	-	1502
novel.2999	NW_016535187.1	338252	338750	+	445
novel.2198	NW_016532579.1	26188	28367	-	1975
novel.2199	NW_016532580.1	3682	62642	+	2337
novel.2192	NW_016532560.1	17348	17821	-	474
novel.2193	NW_016532561.1	149562	149878	-	282
novel.2190	NW_016532560.1	9336	10023	+	491
novel.2191	NW_016532560.1	16135	16665	-	476
novel.2196	NW_016532575.1	8287	9238	+	894
novel.2197	NW_016532575.1	9189	9446	-	233
novel.2194	NW_016532568.1	7464	8326	+	808
novel.2195	NW_016532572.1	1060	1587	+	335
novel.589	NW_016528543.1	14	236	+	223
novel.2228	NW_016532670.1	39171	39878	+	708
novel.2229	NW_016532678.1	6440	7384	+	893
novel.5504	NW_016545477.1	15872	16177	-	281
novel.5505	NW_016545496.1	12890	18233	+	214
novel.2226	NW_016532666.1	196298	214632	+	671
novel.5507	NW_016545496.1	7209	9598	-	2390
novel.5500	NW_016545453.1	65032	73897	-	8866
novel.5501	NW_016545457.1	5702	6124	+	423
novel.5502	NW_016545471.1	31645	32176	+	395
novel.2223	NW_016532658.1	15828	19112	-	339
novel.7982	NW_016578425.1	19	962	+	923
novel.1559	NW_016530765.1	514836	515219	-	345
novel.1558	NW_016530765.1	296356	299165	-	1708
novel.7983	NW_016578425.1	19	962	-	466
novel.1553	NW_016530762.1	143460	146785	+	3326
novel.1552	NW_016530761.1	180683	180972	-	237
novel.1551	NW_016530754.1	38254	38782	-	276

novel.1550	NW_016530753.1	4354	4713	-	221
novel.1557	NW_016530765.1	501301	514863	+	564
novel.1556	NW_016530765.1	255120	255498	+	379
novel.1555	NW_016530765.1	25259	26131	+	842
novel.1554	NW_016530762.1	146995	147642	+	252
novel.1085	NW_016529712.1	162552	162832	-	224
novel.1084	NW_016529708.1	410334	412863	-	829
novel.5649	NW_016546283.1	92165	93714	-	1493
novel.5648	NW_016546283.1	85819	86311	-	443
novel.1081	NW_016529700.1	179441	190211	-	227
novel.1080	NW_016529699.1	38013	38853	+	282
novel.1083	NW_016529703.1	67480	69902	+	858
novel.1082	NW_016529702.1	31220	58507	+	429
novel.5643	NW_016546240.1	557650	559300	-	1566
novel.5642	NW_016546240.1	538310	538607	-	245
novel.5641	NW_016546240.1	261185	263955	-	1560
novel.5640	NW_016546240.1	22111	24139	-	1980
novel.5647	NW_016546274.1	2232	5346	-	1029
novel.5646	NW_016546272.1	9727	10087	+	312
novel.5645	NW_016546249.1	15250	24736	-	332
novel.5644	NW_016546240.1	568954	574769	-	3728
novel.7984	NW_016578463.1	55601	56893	+	1244
novel.7985	NW_016578463.1	48543	51125	-	651
novel.7865	NW_016574333.1	24352	30553	-	3915
novel.7864	NW_016574333.1	16821	21827	-	5007
novel.7867	NW_016574377.1	3569	8110	-	2957
novel.7866	NW_016574333.1	61764	70737	-	848
novel.7861	NW_016574333.1	47073	51979	+	4351
novel.7860	NW_016574333.1	32797	36730	+	3824
novel.7863	NW_016574333.1	8	3614	-	3560
novel.7862	NW_016574333.1	56301	57544	+	448
novel.7869	NW_016574544.1	1	5315	-	2975
novel.7868	NW_016574544.1	1	2200	+	2143
novel.8594	NW_016602443.1	1	2339	-	1896
novel.1889	NW_016531686.1	703798	704089	+	214
novel.1888	NW_016531685.1	3076	4021	+	891
novel.1339	NW_016530248.1	12189	20168	-	1166
novel.1338	NW_016530248.1	29568	30564	+	488
novel.1885	NW_016531672.1	94969	95772	-	424
novel.1884	NW_016531672.1	17308	23345	-	5982
novel.1887	NW_016531685.1	201	2820	+	2567
novel.1886	NW_016531674.1	56652	72027	+	2275
novel.1333	NW_016530235.1	77945	78802	+	802
novel.7283	NW_016562174.1	3516	4781	+	1266

novel.1883	NW_016531672.1	48262	49058	-	378
novel.1882	NW_016531672.1	7196	7479	-	233
novel.7282	NW_016562125.1	4318	4864	+	458
novel.8590	NW_016602317.1	1	732	-	695
novel.8263	NW_016587498.1	11441	15398	+	544
novel.773	NW_016529012.1	137424	137712	+	289
novel.772	NW_016529011.1	96466	103318	-	372
novel.771	NW_016529000.1	64167	64366	+	200
novel.770	NW_016528996.1	295122	295701	-	281
novel.777	NW_016529038.1	69737	70014	-	231
novel.776	NW_016529036.1	174824	182678	-	213
novel.775	NW_016529031.1	439507	440854	+	1348
novel.774	NW_016529016.1	92986	94314	+	387
novel.779	NW_016529046.1	168009	169841	+	1798
novel.7286	NW_016562195.1	1	5483	-	476
novel.4598	NW_016540952.1	1	1674	+	1674
novel.4599	NW_016540952.1	306	1680	+	1345
novel.4590	NW_016540926.1	44124	46037	-	988
novel.4591	NW_016540934.1	62735	63755	+	1021
novel.4592	NW_016540934.1	78830	80030	-	358
novel.4593	NW_016540940.1	1617	1968	+	322
novel.4594	NW_016540940.1	113340	114565	-	1173
novel.4595	NW_016540940.1	126639	127178	-	249
novel.4596	NW_016540950.1	160495	171017	+	324
novel.4597	NW_016540951.1	14046	14413	+	276
novel.6738	NW_016554896.1	56268	57358	+	1008
novel.6739	NW_016554958.1	8853	9089	+	237
novel.6732	NW_016554800.1	16809	18184	-	1376
novel.6733	NW_016554800.1	20649	22374	-	1632
novel.6730	NW_016554798.1	23803	24482	+	218
novel.6731	NW_016554800.1	16508	18311	+	1657
novel.6736	NW_016554865.1	118573	118935	-	221
novel.6737	NW_016554886.1	6010	7311	-	521
novel.6734	NW_016554851.1	24330	42568	-	806
novel.6735	NW_016554865.1	114389	132047	-	1819
novel.3010	NW_016535245.1	105714	106086	+	373
novel.3011	NW_016535245.1	232853	233277	+	405
novel.3012	NW_016535245.1	438635	439330	+	696
novel.3013	NW_016535245.1	412360	412775	-	327
novel.3014	NW_016535252.1	20108	21172	-	210
novel.3015	NW_016535255.1	320295	329247	+	8904
novel.3016	NW_016535255.1	333652	365758	+	485
novel.3017	NW_016535255.1	334403	334729	+	232
novel.3018	NW_016535255.1	413844	414115	+	246

novel.3019	NW_016535264.1	23575	23910	-	336
novel.5444	NW_016545110.1	687156	689020	+	1131
novel.7487	NW_016565904.1	2630	15560	+	930
novel.7486	NW_016565898.1	90992	94611	+	2739
novel.7485	NW_016565887.1	33991	36181	+	2146
novel.7484	NW_016565887.1	69	19460	+	1444
novel.7483	NW_016565866.1	36582	36964	+	383
novel.7482	NW_016565744.1	13197	31980	-	526
novel.7481	NW_016565743.1	388	1005	+	586
novel.7480	NW_016565721.1	2149	3065	-	230
novel.7489	NW_016565904.1	11686	15560	-	432
novel.7488	NW_016565904.1	2630	9522	-	696
novel.3906	NW_016538186.1	36702	51743	+	432
novel.3907	NW_016538200.1	99728	101891	+	2109
novel.3904	NW_016538169.1	27240	30626	+	217
novel.3905	NW_016538169.1	46497	55834	+	482
novel.3902	NW_016538167.1	14629	15327	+	644
novel.3903	NW_016538167.1	13669	15216	-	1493
novel.3900	NW_016538163.1	172420	172765	+	289
novel.3901	NW_016538164.1	42998	46468	-	1801
novel.3908	NW_016538203.1	2337	55366	-	674
novel.3909	NW_016538209.1	132913	137919	-	1088
novel.5446	NW_016545110.1	740740	742309	+	987
novel.2761	NW_016534291.1	466682	467076	+	339
novel.2760	NW_016534290.1	52047	56734	+	1227
novel.2763	NW_016534291.1	392274	404961	-	257
novel.2762	NW_016534291.1	250725	251243	-	519
novel.2765	NW_016534292.1	327923	329471	+	665
novel.2764	NW_016534292.1	246921	247302	+	382
novel.2767	NW_016534292.1	116409	118928	-	649
novel.2766	NW_016534292.1	47002	51292	-	2765
novel.2769	NW_016534292.1	320263	323097	-	2672
novel.2768	NW_016534292.1	169713	184797	-	791
novel.2505	NW_016533580.1	40911	158706	-	515
novel.2504	NW_016533580.1	158564	159011	+	387
novel.2507	NW_016533608.1	61896	107080	+	257
novel.2506	NW_016533584.1	68612	93584	-	687
novel.2501	NW_016533566.1	3554	4113	-	508
novel.2500	NW_016533556.1	16791	17153	-	306
novel.2503	NW_016533575.1	28719	29810	-	631
novel.2502	NW_016533574.1	732	6184	+	379
novel.2509	NW_016533617.1	45517	100791	+	1197
novel.2508	NW_016533608.1	187181	191225	-	444
novel.5416	NW_016544965.1	12506	12773	+	212

novel.5417	NW_016544965.1	11688	12154	-	419
novel.2374	NW_016533184.1	6007	6568	-	392
novel.5415	NW_016544948.1	3784	4367	+	310
novel.5412	NW_016544943.1	26979	27514	-	536
novel.5413	NW_016544944.1	2798	3234	+	383
novel.5410	NW_016544936.1	5624	5976	+	300
novel.5411	NW_016544936.1	5827	7330	-	370
novel.3616	NW_016537251.1	188337	192617	+	1207
novel.3617	NW_016537251.1	205319	205567	+	249
novel.5418	NW_016544970.1	14844	66880	-	3181
novel.2379	NW_016533195.1	20103	20678	-	576
novel.6886	NW_016556545.1	29961	30740	+	742
novel.6501	NW_016552711.1	3	461	-	459
novel.6500	NW_016552681.1	155704	156043	+	252
novel.6503	NW_016552711.1	7304	11890	-	4508
novel.6502	NW_016552711.1	673	5336	-	520
novel.6505	NW_016552761.1	2381	3715	+	1159
novel.6504	NW_016552749.1	1156	6026	+	628
novel.6507	NW_016552779.1	6276	7133	-	704
novel.6506	NW_016552774.1	11634	11966	+	288
novel.6509	NW_016552786.1	5554	5861	+	267
novel.6508	NW_016552779.1	39070	40962	-	927
novel.1830	NW_016531541.1	28319	30582	+	2264
novel.1831	NW_016531541.1	339873	342816	+	2944
novel.1832	NW_016531541.1	110113	110667	-	303
novel.3291	NW_016536092.1	28059	28583	-	472
novel.1833	NW_016531543.1	519	7977	-	1389
novel.1834	NW_016531551.1	199627	219655	+	898
novel.1835	NW_016531552.1	46255	47288	+	221
novel.1836	NW_016531552.1	48665	49163	+	410
novel.1837	NW_016531552.1	45435	46395	-	324
novel.7078	NW_016558620.1	41237	42445	-	987
novel.7079	NW_016558620.1	50435	51767	-	771
novel.7076	NW_016558619.1	15169	15641	+	359
novel.7077	NW_016558620.1	123208	124405	+	1141
novel.7074	NW_016558611.1	12137	12403	-	243
novel.7075	NW_016558619.1	7309	9931	+	290
novel.7072	NW_016558552.1	7879	8703	-	825
novel.7073	NW_016558557.1	1	5258	-	283
novel.7070	NW_016558526.1	833	1903	-	651
novel.7071	NW_016558526.1	19481	53598	-	245
novel.3296	NW_016536100.1	27510	27804	-	240
novel.3297	NW_016536119.1	158151	158443	-	293
novel.3922	NW_016538269.1	73091	78717	+	675

novel.5368	NW_016544660.1	1	534	-	534
novel.5369	NW_016544673.1	42685	43415	-	695
novel.3295	NW_016536100.1	22864	23915	-	935
novel.5360	NW_016544608.1	28225	53396	-	902
novel.5361	NW_016544608.1	67781	68549	-	769
novel.5362	NW_016544608.1	73742	79063	-	5322
novel.5363	NW_016544621.1	9815	16229	+	647
novel.5364	NW_016544627.1	223005	223328	-	251
novel.5365	NW_016544629.1	128950	130950	+	1669
novel.5366	NW_016544629.1	131911	133549	-	1639
novel.5367	NW_016544643.1	9482	9741	-	227
novel.238	NW_016527767.1	133619	133920	+	302
novel.239	NW_016527774.1	384632	385189	+	558
novel.548	NW_016528457.1	46271	47571	-	1169
novel.549	NW_016528458.1	47617	53344	+	491
novel.232	NW_016527738.1	110514	141736	-	1464
novel.545	NW_016528454.1	16343	16887	+	545
novel.230	NW_016527737.1	66331	67706	-	1106
novel.547	NW_016528457.1	4111	5540	-	379
novel.540	NW_016528448.1	222011	222426	+	360
novel.541	NW_016528448.1	222511	223437	-	560
novel.234	NW_016527746.1	122683	123059	+	377
novel.235	NW_016527753.1	255162	259135	+	274
novel.7292	NW_016562271.1	4251	5109	-	838
novel.7293	NW_016562306.1	59263	61383	-	2121
novel.7290	NW_016562233.1	3359	3935	+	522
novel.7291	NW_016562233.1	3077	3766	-	646
novel.7296	NW_016562324.1	75834	78142	-	1895
novel.7297	NW_016562328.1	2003	3222	-	1196
novel.7294	NW_016562310.1	2465	6945	+	989
novel.7295	NW_016562319.1	1	1800	-	233
novel.7298	NW_016562345.1	29764	30244	+	447
novel.7299	NW_016562350.1	6208	6790	+	526
novel.4019	NW_016538529.1	5034	5452	+	419
novel.4018	NW_016538513.1	190569	193894	-	3228
novel.4011	NW_016538502.1	48796	49494	-	699
novel.4010	NW_016538502.1	13292	14140	-	794
novel.4013	NW_016538507.1	74397	74930	+	485
novel.4012	NW_016538507.1	64161	66187	+	1543
novel.4015	NW_016538507.1	95727	99950	-	1370
novel.4014	NW_016538507.1	64161	66187	-	1601
novel.4017	NW_016538513.1	220155	220672	+	518
novel.4016	NW_016538513.1	157462	158904	+	650
novel.7160	NW_016559733.1	163488	167028	-	1349

novel.7161	NW_016559738.1	9575	12561	+	2898
novel.7162	NW_016559738.1	6280	7320	-	1041
novel.3591	NW_016537153.1	16577	19959	+	244
novel.3590	NW_016537152.1	15369	22113	-	3505
novel.3593	NW_016537159.1	1	1198	-	1147
novel.3592	NW_016537156.1	5677	8609	-	1694
novel.3595	NW_016537163.1	192339	192577	-	218
novel.3594	NW_016537163.1	192382	193360	+	723
novel.3597	NW_016537167.1	25284	25565	-	227
novel.3596	NW_016537165.1	68470	69037	+	454
novel.3599	NW_016537183.1	8524	12152	+	335
novel.3598	NW_016537168.1	284655	288417	+	2336
novel.5553	NW_016545683.1	256994	257355	+	285
novel.7165	NW_016559779.1	1	563	-	507
novel.7166	NW_016559807.1	1	571	+	358
novel.7167	NW_016559811.1	46912	47694	-	460
novel.7168	NW_016559811.1	50789	51212	-	250
novel.7169	NW_016559812.1	149161	150967	+	1763
novel.2672	NW_016534043.1	90557	90925	-	369
novel.353	NW_016528038.1	75324	75592	+	217
novel.2671	NW_016534041.1	1134	1690	+	506
novel.352	NW_016528038.1	73268	73492	+	225
novel.5566	NW_016545790.1	29942	32321	-	452
novel.5567	NW_016545806.1	75600	75856	+	257
novel.2244	NW_016532733.1	96582	97710	-	667
novel.2245	NW_016532740.1	120664	123354	+	2591
novel.5562	NW_016545768.1	104588	129326	+	517
novel.5563	NW_016545768.1	1	872	-	872
novel.5560	NW_016545764.1	13333	13734	-	361
novel.5561	NW_016545768.1	102807	111142	+	316
novel.2248	NW_016532753.1	170761	171079	+	221
novel.5569	NW_016545843.1	2162	3950	+	422
novel.1539	NW_016530732.1	453296	461939	+	1674
novel.1538	NW_016530727.1	62881	64756	-	1117
novel.1535	NW_016530713.1	466209	467093	-	885
novel.1534	NW_016530713.1	458045	459080	-	376
novel.1537	NW_016530725.1	72802	74207	-	252
novel.1536	NW_016530713.1	467139	471687	-	2387
novel.1531	NW_016530713.1	10395	11033	+	263
novel.1530	NW_016530706.1	166312	198360	+	1089
novel.1533	NW_016530713.1	670947	674303	+	1114
novel.1532	NW_016530713.1	525986	536389	+	1107
novel.5629	NW_016546211.1	4060	6209	-	2046
novel.5628	NW_016546211.1	2853	8769	+	5656

novel.5625	NW_016546201.1	206500	208387	-	599
novel.5624	NW_016546195.1	197628	197897	-	270
novel.5627	NW_016546206.1	12	18551	+	328
novel.5626	NW_016546201.1	225145	235730	-	995
novel.5621	NW_016546186.1	116334	123367	+	939
novel.5620	NW_016546179.1	4655	28765	+	1034
novel.5623	NW_016546189.1	15011	18604	+	3594
novel.5622	NW_016546186.1	110370	112845	-	295
novel.7849	NW_016574099.1	534	2120	+	346
novel.7848	NW_016574086.1	11032	13289	+	361
novel.7847	NW_016574029.1	32914	48738	+	1113
novel.7846	NW_016574015.1	17966	18583	-	562
novel.7845	NW_016573963.1	76	875	-	800
novel.7844	NW_016573839.1	1	2658	-	224
novel.7843	NW_016573804.1	1176	1515	-	284
novel.59	NW_016527375.1	138917	139417	+	445
novel.7841	NW_016573779.1	1	1361	+	1316
novel.7840	NW_016573741.1	12595	17414	+	4343
novel.5079	NW_016542931.1	144000	144739	+	448
novel.8837	NW_016607825.1	1	4357	+	4357
novel.2674	NW_016534052.1	2541	26984	+	302
novel.5064	NW_016542862.1	101804	108925	+	201
novel.8384	NW_016591733.1	2096	3874	-	1724
novel.8387	NW_016591816.1	221	5428	-	300
novel.8834	NW_016607750.1	2757	15254	-	256
novel.8833	NW_016607707.1	5491	9865	-	342
novel.8380	NW_016591719.1	397	3735	+	3339
novel.5197	NW_016543632.1	182903	222057	+	1368
novel.5196	NW_016543626.1	19232	19655	+	424
novel.5195	NW_016543617.1	56126	58147	-	1283
novel.5194	NW_016543616.1	260071	294930	+	592
novel.5193	NW_016543612.1	54781	55998	-	1218
novel.5192	NW_016543612.1	2047	3827	-	1724
novel.5191	NW_016543611.1	5819	6535	-	717
novel.5190	NW_016543610.1	8009	8524	+	516
novel.8382	NW_016591729.1	1	4529	-	2652
novel.5199	NW_016543632.1	220346	222064	-	1689
novel.5198	NW_016543632.1	202369	203344	+	976
novel.3650	NW_016537360.1	3565	27943	-	413
novel.5069	NW_016542877.1	667288	668169	-	753
novel.3841	NW_016537937.1	206130	206836	+	537
novel.5068	NW_016542877.1	621517	622404	-	888
novel.4572	NW_016540880.1	227966	304040	+	221
novel.4573	NW_016540885.1	12888	13818	+	931

novel.4570	NW_016540858.1	79973	83010	+	1917
novel.4571	NW_016540868.1	1946	2226	+	227
novel.4576	NW_016540891.1	7147	7757	+	309
novel.4577	NW_016540891.1	7887	10865	+	2979
novel.4574	NW_016540885.1	41067	72536	+	1203
novel.4575	NW_016540888.1	1505	3636	-	2132
novel.4578	NW_016540891.1	497	2481	-	878
novel.4579	NW_016540892.1	10466	24105	+	545
novel.6718	NW_016554721.1	8905	12674	-	3713
novel.6719	NW_016554722.1	34731	35251	+	327
novel.6714	NW_016554713.1	5449	8547	-	2861
novel.6715	NW_016554713.1	12035	13235	-	1145
novel.6716	NW_016554721.1	1586	12674	+	2770
novel.6717	NW_016554721.1	1498	1750	-	220
novel.6710	NW_016554687.1	1517	2734	+	449
novel.6711	NW_016554713.1	5449	8168	+	2617
novel.6712	NW_016554713.1	3935	4371	-	391
novel.6713	NW_016554713.1	4443	4749	-	258
novel.6995	NW_016557696.1	620	870	-	227
novel.3438	NW_016536638.1	479722	482021	+	2056
novel.3439	NW_016536638.1	286653	288014	-	355
novel.6152	NW_016549869.1	503	1974	+	1427
novel.6153	NW_016549869.1	33295	38614	+	1458
novel.6150	NW_016549859.1	8102	10498	-	646
novel.2749	NW_016534219.1	17815	93714	-	1127
novel.2748	NW_016534218.1	6646	11389	+	4591
novel.2743	NW_016534212.1	276008	277324	+	373
novel.2742	NW_016534209.1	130269	134483	-	4215
novel.2741	NW_016534209.1	104363	104624	-	208
novel.2740	NW_016534209.1	91771	92123	+	301
novel.2747	NW_016534217.1	266897	267611	+	244
novel.2746	NW_016534216.1	39799	41506	-	298
novel.2745	NW_016534215.1	228368	229152	-	785
novel.2744	NW_016534215.1	134941	135242	+	302
novel.8668	NW_016604698.1	2110	3514	+	999
novel.8669	NW_016604703.1	1058	2344	+	1287
novel.3434	NW_016536636.1	218830	285039	+	383
novel.8662	NW_016604590.1	2729	3427	-	699
novel.8663	NW_016604627.1	2628	3455	+	768
novel.8660	NW_016604534.1	81	1501	+	379
novel.8661	NW_016604590.1	2729	3427	+	699
novel.8666	NW_016604672.1	92	2126	+	218
novel.8667	NW_016604683.1	1279	3125	+	313
novel.8664	NW_016604627.1	2478	3455	-	978

novel.8665	NW_016604655.1	1943	3445	-	805
novel.3928	NW_016538282.1	33350	33829	-	480
novel.3929	NW_016538291.1	93686	98648	-	4963
novel.3298	NW_016536122.1	37999	38864	-	866
novel.3299	NW_016536125.1	220104	220848	+	745
novel.3920	NW_016538260.1	17375	39546	+	218
novel.3921	NW_016538268.1	237162	240683	-	1084
novel.3294	NW_016536100.1	20643	21795	-	455
novel.3923	NW_016538270.1	151064	156807	+	236
novel.3292	NW_016536096.1	1025	1267	+	201
novel.3293	NW_016536100.1	23058	24105	+	1028
novel.3290	NW_016536080.1	87937	107503	-	1447
novel.3927	NW_016538280.1	30698	65352	-	281
novel.2529	NW_016533653.1	109723	110298	-	326
novel.2528	NW_016533652.1	155211	160198	+	1255
novel.2527	NW_016533650.1	5352	16237	+	416
novel.2526	NW_016533647.1	1608	2037	-	385
novel.2525	NW_016533647.1	71505	78540	+	842
novel.2524	NW_016533646.1	215542	215809	-	268
novel.2523	NW_016533644.1	31757	33169	-	544
novel.2522	NW_016533642.1	53910	55484	+	805
novel.2521	NW_016533637.1	45911	53069	-	203
novel.2520	NW_016533630.1	17837	18592	-	756
novel.1351	NW_016530274.1	269901	270158	+	258
novel.6158	NW_016549878.1	3030	4928	+	1117
novel.6159	NW_016549929.1	4857	5376	+	340
novel.3432	NW_016536626.1	7531	8811	+	1162
novel.3433	NW_016536626.1	42921	43710	+	515
novel.3430	NW_016536614.1	222517	223050	+	381
novel.3431	NW_016536624.1	20459	20915	+	401
novel.6156	NW_016549873.1	101723	114431	+	488
novel.6157	NW_016549873.1	36551	37149	-	390
novel.6154	NW_016549869.1	503	840	-	338
novel.3435	NW_016536636.1	291944	297345	+	200
novel.4802	NW_016541767.1	52008	52931	+	888
novel.4803	NW_016541777.1	744	1502	+	759
novel.4800	NW_016541764.1	2169	7111	-	1053
novel.4801	NW_016541767.1	16249	19656	+	1430
novel.4806	NW_016541779.1	9949	10866	-	269
novel.4807	NW_016541785.1	100308	101397	+	600
novel.4804	NW_016541777.1	737	1502	-	766
novel.4805	NW_016541779.1	139649	140269	+	227
novel.4808	NW_016541786.1	73244	75079	-	498
novel.4809	NW_016541797.1	54072	54910	+	839

novel.7280	NW_016562079.1	18300	19711	+	429
novel.6529	NW_016553100.1	103442	104846	+	1198
novel.6528	NW_016553100.1	46376	52675	+	605
novel.6523	NW_016552990.1	7878	11338	-	350
novel.6522	NW_016552976.1	1034	1842	+	503
novel.6521	NW_016552939.1	22544	23325	+	727
novel.6520	NW_016552927.1	68457	73068	-	297
novel.6527	NW_016553059.1	11193	12592	+	1400
novel.6526	NW_016553017.1	1	1277	-	1218
novel.6525	NW_016553017.1	1	1217	+	1193
novel.6524	NW_016553001.1	10345	12135	+	1063
novel.2608	NW_016533845.1	32509	32776	-	268
novel.2609	NW_016533848.1	208234	208834	+	295
novel.199	NW_016527663.1	295936	296452	-	222
novel.2606	NW_016533842.1	225612	227692	+	2081
novel.198	NW_016527663.1	100185	105088	-	559
novel.5927	NW_016548285.1	5857	10301	-	694
novel.2604	NW_016533837.1	361	758	+	378
novel.2255	NW_016532816.1	31524	60162	-	456
novel.2605	NW_016533837.1	5294	5722	-	391
novel.2254	NW_016532808.1	9569	46252	-	1559
novel.2602	NW_016533828.1	46966	47357	-	368
novel.2257	NW_016532826.1	68947	69358	-	369
novel.2603	NW_016533837.1	2	5576	+	376
novel.2256	NW_016532819.1	25766	68504	+	3132
novel.2600	NW_016533828.1	121202	123457	+	2176
novel.5571	NW_016545846.1	57869	59980	+	810
novel.2601	NW_016533828.1	45678	46902	-	1225
novel.5570	NW_016545843.1	17932	19592	+	1604
novel.5573	NW_016545861.1	82782	88281	+	230
novel.197	NW_016527663.1	29249	33967	+	1797
novel.5572	NW_016545851.1	56030	58647	+	2618
novel.196	NW_016527662.1	630270	652462	+	297
novel.7284	NW_016562174.1	3221	4761	-	1492
novel.5579	NW_016545897.1	4972	5911	-	940
novel.2258	NW_016532836.1	142066	142440	-	227
novel.7212	NW_016560689.1	6570	6964	+	247
novel.1278	NW_016530103.1	24592	25318	+	589
novel.1279	NW_016530106.1	271391	273566	+	2176
novel.7213	NW_016560720.1	10553	14079	-	3527
novel.7287	NW_016562198.1	11907	20694	+	535
novel.1273	NW_016530096.1	65248	67488	-	2241
novel.1270	NW_016530093.1	45583	48731	-	779
novel.1271	NW_016530095.1	191680	193508	-	999

novel.1276	NW_016530102.1	495766	496125	+	309
novel.1277	NW_016530103.1	29523	32177	+	2552
novel.1274	NW_016530101.1	53970	70105	+	844
novel.1275	NW_016530102.1	455903	456701	+	799
novel.8522	NW_016599351.1	1	678	-	392
novel.8525	NW_016599383.1	61	1653	-	466
novel.8524	NW_016599377.1	1	1652	+	204
novel.1126	NW_016529792.1	115686	117919	-	897
novel.8527	NW_016599430.1	1	1660	-	837
novel.7215	NW_016560723.1	1306	3376	-	2040
novel.7337	NW_016562957.1	15819	16389	-	571
novel.8051	NW_016580534.1	529	1396	-	868
novel.7051	NW_016558195.1	6563	8256	+	1694
novel.8053	NW_016580747.1	441	794	-	307
novel.8052	NW_016580699.1	5689	5897	-	209
novel.8055	NW_016580878.1	2350	3153	-	776
novel.8054	NW_016580878.1	2350	3215	+	762
novel.8057	NW_016580991.1	2138	2469	-	275
novel.8056	NW_016580955.1	1640	2399	-	760
novel.7058	NW_016558259.1	1559	2168	-	610
novel.7059	NW_016558298.1	10402	11690	+	1235
novel.7728	NW_016570455.1	50669	54794	-	4126
novel.7729	NW_016570513.1	7963	8275	+	259
novel.1127	NW_016529798.1	75243	93210	-	1265
novel.5382	NW_016544719.1	9936	10340	+	350
novel.5383	NW_016544724.1	132346	134451	-	591
novel.5380	NW_016544714.1	49892	52340	-	297
novel.5381	NW_016544716.1	115166	150186	+	2808
novel.5386	NW_016544729.1	7825	9050	+	1175
novel.5387	NW_016544729.1	16972	17663	-	671
novel.5384	NW_016544725.1	6105	8033	-	762
novel.5385	NW_016544726.1	65782	66302	-	398
novel.5388	NW_016544730.1	13189	50509	-	1443
novel.5389	NW_016544735.1	2404	3181	+	356
novel.210	NW_016527690.1	17634	39171	-	230
novel.211	NW_016527704.1	10839	25748	-	503
novel.212	NW_016527706.1	30744	31014	-	216
novel.213	NW_016527707.1	7208	13033	+	468
novel.214	NW_016527708.1	133061	136032	+	475
novel.215	NW_016527709.1	43921	45177	+	400
novel.216	NW_016527709.1	42810	43934	-	975
novel.217	NW_016527712.1	7101	8220	-	416
novel.218	NW_016527714.1	220562	228069	+	264
novel.219	NW_016527714.1	214983	215779	-	753

novel.8275	NW_016587772.1	5941	6405	+	422
novel.1500	NW_016530644.1	18011	18611	-	546
novel.8277	NW_016587782.1	1161	1746	-	540
novel.8276	NW_016587772.1	1	1094	-	1094
novel.8271	NW_016587693.1	7996	8668	-	673
novel.8270	NW_016587666.1	440	1127	+	661
novel.8273	NW_016587729.1	20633	30453	+	9821
novel.1501	NW_016530649.1	175716	176248	-	533
novel.8279	NW_016587904.1	394	985	+	535
novel.1050	NW_016529637.1	78698	102294	-	620
novel.1401	NW_016530376.1	11849	26280	-	307
novel.1503	NW_016530655.1	29927	63801	+	638
novel.1400	NW_016530376.1	27747	28012	+	266
novel.1504	NW_016530655.1	86208	86414	+	207
novel.1403	NW_016530384.1	14778	52783	-	381
novel.1505	NW_016530658.1	13040	17436	+	337
novel.1402	NW_016530379.1	56520	56724	+	205
novel.1506	NW_016530658.1	94062	95429	+	1244
novel.1405	NW_016530385.1	13089	22658	-	464
novel.1055	NW_016529640.1	244530	245171	-	489
novel.1404	NW_016530385.1	119781	120513	+	578
novel.4389	NW_016540173.1	8200	8675	-	366
novel.4388	NW_016540173.1	49536	60186	+	575
novel.1407	NW_016530389.1	9838	10803	-	878
novel.4381	NW_016540153.1	7387	8228	-	283
novel.4380	NW_016540151.1	7495	9236	+	1254
novel.4383	NW_016540155.1	211465	215528	+	3839
novel.4382	NW_016540154.1	3418	6159	-	2646
novel.4385	NW_016540155.1	226479	227198	-	665
novel.4384	NW_016540155.1	213525	242911	-	1804
novel.4387	NW_016540168.1	52500	56029	-	2052
novel.4386	NW_016540166.1	5321	5704	-	384
novel.3601	NW_016537192.1	28333	28890	-	530
novel.3600	NW_016537184.1	57984	60920	-	562
novel.3603	NW_016537204.1	117268	118114	+	795
novel.3602	NW_016537204.1	116665	117266	+	552
novel.3605	NW_016537204.1	117268	117936	-	632
novel.3604	NW_016537204.1	115156	115911	-	727
novel.3607	NW_016537208.1	23469	23737	-	269
novel.3606	NW_016537206.1	75764	76771	+	982
novel.3609	NW_016537219.1	89329	91286	-	1928
novel.3608	NW_016537219.1	171527	171830	+	268
novel.6899	NW_016556734.1	27599	28543	-	889
novel.6898	NW_016556734.1	241690	242278	+	449

novel.5527	NW_016545566.1	410383	410822	+	332
novel.4033	NW_016538611.1	11797	39800	+	480
novel.4032	NW_016538609.1	48	1001	+	911
novel.8589	NW_016602306.1	1	2288	-	2288
novel.4030	NW_016538581.1	91988	92535	-	320
novel.4037	NW_016538625.1	38877	39642	-	766
novel.4036	NW_016538625.1	34454	38559	-	268
novel.4035	NW_016538621.1	175491	197264	-	2690
novel.4034	NW_016538611.1	9939	37587	-	209
novel.8583	NW_016602036.1	285	1423	+	214
novel.8582	NW_016601954.1	1	1958	+	503
novel.4039	NW_016538638.1	1790	2218	+	376
novel.4038	NW_016538632.1	268036	268381	-	309
novel.8587	NW_016602183.1	1	2213	-	675
novel.8586	NW_016602173.1	66	2252	-	2187
novel.8585	NW_016602156.1	1	1437	-	609
novel.8584	NW_016602083.1	125	1163	-	313
novel.6099	NW_016549532.1	444300	450429	+	901
novel.6098	NW_016549530.1	236068	236791	+	695
novel.6093	NW_016549498.1	1600	2148	-	374
novel.6092	NW_016549484.1	16463	26265	+	1160
novel.6091	NW_016549462.1	114839	115619	-	496
novel.6090	NW_016549459.1	62949	66828	-	3880
novel.6097	NW_016549523.1	1	4393	-	317
novel.6096	NW_016549512.1	49387	50530	-	1144
novel.6095	NW_016549512.1	68472	68982	+	462
novel.6094	NW_016549512.1	67691	67999	+	261
novel.8612	NW_016602798.1	1149	2435	+	538
novel.2268	NW_016532849.1	43211	49888	-	2074
novel.2269	NW_016532849.1	110211	111624	-	834
novel.5818	NW_016547480.1	67566	69780	-	2215
novel.5819	NW_016547486.1	100101	100524	+	424
novel.5812	NW_016547453.1	66398	67886	+	951
novel.2261	NW_016532844.1	204908	205258	-	351
novel.5810	NW_016547418.1	148	413	+	212
novel.5811	NW_016547440.1	1	1194	+	1139
novel.5816	NW_016547480.1	229616	233438	+	3398
novel.2265	NW_016532849.1	441312	444959	+	1709
novel.5814	NW_016547469.1	109284	109978	-	581
novel.5815	NW_016547478.1	1276	5073	+	3557
novel.858	NW_016529238.1	115707	117383	-	1677
novel.859	NW_016529242.1	15716	16248	+	323
novel.852	NW_016529233.1	39081	39774	+	498
novel.853	NW_016529233.1	303119	305147	-	236

novel.850	NW_016529232.1	18235	18980	-	721
novel.851	NW_016529232.1	56226	61721	-	2227
novel.856	NW_016529238.1	101306	102811	+	425
novel.857	NW_016529238.1	20909	21845	-	465
novel.854	NW_016529235.1	76645	183078	-	1798
novel.855	NW_016529237.1	205081	205877	-	745
novel.1041	NW_016529622.1	47445	89502	-	418
novel.1040	NW_016529620.1	69256	69672	-	362
novel.1043	NW_016529633.1	13842	14183	+	217
novel.1042	NW_016529626.1	161811	165733	+	517
novel.1045	NW_016529633.1	1029280	1055644	+	1375
novel.1044	NW_016529633.1	280844	281301	+	436
novel.1047	NW_016529633.1	311844	314490	-	2647
novel.1046	NW_016529633.1	1143935	1162558	+	516
novel.1049	NW_016529637.1	193672	194232	+	479
novel.1048	NW_016529637.1	71597	72350	+	332
novel.1519	NW_016530683.1	80972	82137	+	1110
novel.1518	NW_016530674.1	227885	229844	-	1928
novel.5607	NW_016546080.1	507	6235	+	1241
novel.5606	NW_016546059.1	1	834	+	808
novel.5605	NW_016546054.1	50352	83611	-	3256
novel.5604	NW_016546048.1	12504	33682	+	298
novel.5603	NW_016546037.1	1	1775	-	616
novel.5602	NW_016546037.1	1	437	+	382
novel.5601	NW_016546021.1	38556	39255	+	700
novel.5600	NW_016546019.1	16495	19011	+	1252
novel.5609	NW_016546092.1	3088	3586	+	499
novel.5608	NW_016546081.1	62949	63634	-	662
novel.1206	NW_016529950.1	117	407	+	253
novel.8550	NW_016600524.1	560	1771	+	1212
novel.4639	NW_016541109.1	199097	200988	+	1212
novel.4638	NW_016541105.1	9345	9744	+	346
novel.4631	NW_016541063.1	1	833	+	789
novel.4630	NW_016541062.1	127234	128535	+	1252
novel.4633	NW_016541064.1	28624	29027	+	265
novel.4632	NW_016541063.1	897	1849	+	931
novel.4635	NW_016541089.1	59214	59733	-	465
novel.4634	NW_016541084.1	75986	76596	-	337
novel.4637	NW_016541097.1	12492	12817	+	269
novel.4636	NW_016541095.1	109609	110637	-	813
novel.8552	NW_016600642.1	29	1873	+	721
novel.8337	NW_016590555.1	5387	5727	-	284
novel.4554	NW_016540750.1	82904	89578	-	224
novel.4555	NW_016540751.1	186565	187054	-	332

novel.4556	NW_016540757.1	102231	102439	+	209
novel.4557	NW_016540757.1	1404	4174	-	1722
novel.4550	NW_016540745.1	8093	16401	-	681
novel.4551	NW_016540746.1	235169	235390	+	222
novel.4552	NW_016540746.1	155914	165040	-	332
novel.4553	NW_016540748.1	88442	89620	+	1094
novel.4558	NW_016540757.1	17225	20114	-	1028
novel.4559	NW_016540765.1	10551	10878	-	231
novel.6776	NW_016555317.1	73456	81057	+	510
novel.6777	NW_016555317.1	144291	145277	+	210
novel.6774	NW_016555313.1	1665	10332	+	1704
novel.6775	NW_016555316.1	4340	5359	-	1020
novel.6772	NW_016555302.1	227810	229517	+	1103
novel.6773	NW_016555306.1	23533	28918	+	528
novel.6770	NW_016555279.1	351972	357682	+	1400
novel.6771	NW_016555293.1	13395	14957	+	1563
novel.6778	NW_016555329.1	98246	100514	+	470
novel.6779	NW_016555345.1	160640	161476	+	741
novel.669	NW_016528742.1	1960	4274	-	437
novel.668	NW_016528737.1	101316	103650	-	2174
novel.661	NW_016528716.1	215105	217256	+	2152
novel.660	NW_016528716.1	118595	120045	+	1413
novel.663	NW_016528716.1	284828	286072	-	595
novel.662	NW_016528716.1	118701	120045	-	1345
novel.665	NW_016528718.1	51121	51403	-	283
novel.664	NW_016528718.1	43492	44842	-	701
novel.667	NW_016528734.1	37554	37810	+	210
novel.666	NW_016528723.1	298553	304834	-	783
novel.8759	NW_016606358.1	327	4388	-	289
novel.2729	NW_016534183.1	3929	26327	-	727
novel.2728	NW_016534181.1	504	6995	-	1041
novel.2899	NW_016534788.1	15372	20370	-	315
novel.2898	NW_016534785.1	255255	256085	+	774
novel.2897	NW_016534785.1	144916	150613	+	5493
novel.2896	NW_016534775.1	6433	6889	-	431
novel.2895	NW_016534768.1	145412	148544	+	3076
novel.2894	NW_016534768.1	17390	20205	+	243
novel.2893	NW_016534763.1	250585	256556	+	5972
novel.2892	NW_016534759.1	125920	127804	-	1885
novel.2723	NW_016534168.1	21389	22990	+	1585
novel.2722	NW_016534166.1	4945	15147	+	260
novel.7559	NW_016567272.1	3405	3986	+	528
novel.7558	NW_016567153.1	110109	110415	+	307
novel.8648	NW_016604072.1	8	2892	+	2885

novel.8649	NW_016604079.1	642	3077	-	2436
novel.7551	NW_016567041.1	28592	28956	+	255
novel.7550	NW_016567037.1	5358	10182	-	359
novel.7553	NW_016567118.1	912	2116	+	218
novel.8647	NW_016604022.1	1420	2823	-	341
novel.8640	NW_016603864.1	1	2955	+	407
novel.8641	NW_016603929.1	1513	2987	-	1003
novel.7557	NW_016567153.1	48241	58977	+	295
novel.7556	NW_016567153.1	45228	63116	+	1327
novel.2097	NW_016532262.1	12308	12778	-	276
novel.6039	NW_016549118.1	760	1013	+	221
novel.2095	NW_016532259.1	364554	364987	-	434
novel.2094	NW_016532238.1	187076	196246	+	487
novel.2093	NW_016532232.1	24398	32543	-	461
novel.2092	NW_016532229.1	207143	207844	-	702
novel.2091	NW_016532229.1	52616	54807	-	2138
novel.3518	NW_016536974.1	28808	45653	+	404
novel.2099	NW_016532263.1	409138	425483	-	608
novel.2098	NW_016532263.1	409138	412382	+	3245
novel.6031	NW_016549086.1	80793	94642	+	829
novel.6030	NW_016549086.1	51271	56113	+	951
novel.6033	NW_016549090.1	162790	166030	+	2439
novel.6032	NW_016549090.1	54782	58250	+	2557
novel.6035	NW_016549090.1	48399	49080	-	441
novel.6034	NW_016549090.1	4002	4474	-	356
novel.6037	NW_016549100.1	17598	25464	+	1661
novel.6036	NW_016549090.1	229066	236904	-	901
novel.2275	NW_016532859.1	174716	178437	-	758
novel.3711	NW_016537551.1	7003	9013	+	1955
novel.3418	NW_016536545.1	62748	73077	+	209
novel.3419	NW_016536545.1	143011	143499	+	461
novel.6174	NW_016550004.1	79846	80078	+	233
novel.6175	NW_016550028.1	6	5445	+	373
novel.6176	NW_016550030.1	3323	3920	+	544
novel.3417	NW_016536538.1	113787	119990	+	1085
novel.3410	NW_016536509.1	463524	487286	+	1297
novel.3411	NW_016536510.1	241801	242322	+	522
novel.3412	NW_016536524.1	17414	21009	-	284
novel.3413	NW_016536532.1	53517	54542	+	976
novel.4824	NW_016541843.1	65458	65936	-	479
novel.4825	NW_016541845.1	15877	20938	+	230
novel.4826	NW_016541845.1	27258	64431	+	509
novel.4827	NW_016541846.1	14022	14305	+	263
novel.4820	NW_016541840.1	174537	175425	+	330

novel.4821	NW_016541840.1	50973	51273	-	244
novel.4822	NW_016541841.1	80590	81107	+	330
novel.4823	NW_016541842.1	77	882	-	451
novel.4828	NW_016541846.1	13045	14064	-	278
novel.4829	NW_016541846.1	14188	26059	-	418
novel.1272	NW_016530096.1	65248	67488	+	2241
novel.6549	NW_016553215.1	72853	74323	-	1423
novel.6548	NW_016553207.1	81243	86675	-	2386
novel.6545	NW_016553168.1	8022	8841	-	765
novel.6544	NW_016553168.1	1619	8822	+	1390
novel.6547	NW_016553172.1	4241	5138	+	898
novel.6546	NW_016553172.1	2047	2697	+	594
novel.6541	NW_016553132.1	6350	8644	+	1080
novel.6540	NW_016553124.1	3514	5947	+	358
novel.6543	NW_016553154.1	15016	15870	+	557
novel.6542	NW_016553154.1	1	1525	+	1472
novel.107	NW_016527479.1	72843	73245	+	241
novel.2813	NW_016534435.1	120325	153849	+	2329
novel.7641	NW_016568897.1	1818	2271	+	276
novel.2812	NW_016534427.1	72424	119952	+	394
novel.6866	NW_016556332.1	3093	3699	+	586
novel.6867	NW_016556333.1	20640	20936	+	262
novel.7640	NW_016568876.1	9587	10072	+	463
novel.6864	NW_016556328.1	120	1682	+	1563
novel.6865	NW_016556328.1	1773	6213	+	4222
novel.6862	NW_016556213.1	2615	2958	-	287
novel.6863	NW_016556265.1	25566	27943	+	2378
novel.6860	NW_016556210.1	33838	37296	+	369
novel.7643	NW_016568913.1	879	1722	-	844
novel.6861	NW_016556213.1	3101	4763	+	1552
novel.1702	NW_016531249.1	37055	52852	+	1128
novel.1703	NW_016531249.1	14298	14974	-	677
novel.1700	NW_016531248.1	162536	165223	-	800
novel.1701	NW_016531248.1	317041	317969	-	721
novel.1706	NW_016531253.1	36983	37241	+	204
novel.1707	NW_016531255.1	103395	108340	+	4789
novel.1704	NW_016531249.1	15072	16421	-	904
novel.1705	NW_016531249.1	37055	37945	-	891
novel.1254	NW_016530069.1	720765	721776	+	978
novel.1255	NW_016530078.1	490204	498090	+	1567
novel.1708	NW_016531255.1	41164	41555	-	221
novel.1257	NW_016530078.1	559369	561966	+	868
novel.1250	NW_016530068.1	1100045	1100390	+	291
novel.1251	NW_016530068.1	128307	130847	-	250

novel.1252	NW_016530068.1	561287	561660	-	246
novel.1253	NW_016530069.1	11563	11897	+	296
novel.5012	NW_016542616.1	29570	31705	-	991
novel.5013	NW_016542619.1	34269	47319	+	327
novel.5010	NW_016542603.1	8252	20166	-	1757
novel.5011	NW_016542603.1	38587	39417	-	831
novel.5016	NW_016542630.1	21649	34653	-	265
novel.5017	NW_016542632.1	204	6300	-	1668
novel.5014	NW_016542622.1	120162	120824	+	376
novel.5015	NW_016542624.1	5386	20950	-	541
novel.5018	NW_016542633.1	31645	31965	+	264
novel.5019	NW_016542641.1	29862	77773	+	447
novel.7645	NW_016568956.1	4533	5626	+	1060
novel.7704	NW_016570161.1	73085	75772	+	2688
novel.7705	NW_016570177.1	12537	23510	-	2248
novel.7706	NW_016570183.1	10562	14364	-	1275
novel.7707	NW_016570191.1	209670	210302	+	388
novel.7700	NW_016570088.1	655	1468	-	779
novel.7701	NW_016570098.1	437	881	+	346
novel.7702	NW_016570134.1	2532	2836	+	305
novel.7703	NW_016570152.1	636	1091	-	229
novel.7708	NW_016570199.1	3805	4375	+	371
novel.7709	NW_016570199.1	4445	5636	+	1192
novel.7644	NW_016568953.1	1	1004	+	911
novel.3926	NW_016538273.1	137467	139601	-	660
novel.588	NW_016528542.1	98067	99192	-	1077
novel.3366	NW_016536393.1	11446	11793	+	294
novel.3495	NW_016536845.1	45586	46021	+	436
novel.580	NW_016528502.1	231113	234186	-	3074
novel.581	NW_016528517.1	312748	315798	-	1100
novel.582	NW_016528519.1	55418	60239	-	435
novel.583	NW_016528522.1	75	430	+	356
novel.584	NW_016528522.1	640607	641066	+	321
novel.585	NW_016528522.1	646	1514	-	699
novel.586	NW_016528533.1	4677	4948	+	236
novel.587	NW_016528542.1	165012	178070	+	639
novel.276	NW_016527901.1	228869	233096	-	418
novel.277	NW_016527902.1	81	1876	-	247
novel.274	NW_016527900.1	295011	298221	-	639
novel.3365	NW_016536392.1	279203	279981	+	779
novel.272	NW_016527900.1	353523	377144	+	960
novel.273	NW_016527900.1	1259	1629	-	236
novel.270	NW_016527897.1	20207	20547	-	288
novel.271	NW_016527899.1	54947	55915	-	348

novel.3362	NW_016536376.1	24494	45583	+	736
novel.278	NW_016527907.1	226508	227531	+	222
novel.279	NW_016527907.1	281388	282458	+	1071
novel.7050	NW_016558180.1	12	1237	-	610
novel.3492	NW_016536832.1	8839	9412	-	519
novel.7723	NW_016570349.1	14348	15634	-	1287
novel.3493	NW_016536838.1	55324	55589	+	266
novel.7052	NW_016558195.1	8939	9308	-	339
novel.7053	NW_016558201.1	36989	37222	+	234
novel.7054	NW_016558201.1	37432	40162	+	250
novel.7055	NW_016558201.1	1	4975	-	2359
novel.7056	NW_016558259.1	82314	95346	+	4581
novel.7057	NW_016558259.1	95473	96119	+	593
novel.3499	NW_016536852.1	9540	10399	-	780
novel.8059	NW_016581009.1	255	559	+	275
novel.8079	NW_016581743.1	2520	3780	+	420
novel.8078	NW_016581739.1	12730	19575	-	751
novel.169	NW_016527599.1	82758	84615	-	1786
novel.8073	NW_016581607.1	7943	8335	+	337
novel.8072	NW_016581539.1	8118	8663	+	324
novel.8071	NW_016581527.1	4	5900	+	700
novel.8070	NW_016581518.1	1	2348	-	2282
novel.8077	NW_016581739.1	18853	19575	+	650
novel.8076	NW_016581714.1	1136	1367	-	232
novel.8075	NW_016581693.1	1288	1770	-	382
novel.8074	NW_016581626.1	27465	28618	-	1134
novel.3629	NW_016537283.1	115417	120323	-	420
novel.3628	NW_016537283.1	223623	224427	+	488
novel.3623	NW_016537265.1	10876	11166	-	237
novel.3622	NW_016537265.1	36771	44387	+	201
novel.3621	NW_016537262.1	122903	123369	-	467
novel.3620	NW_016537256.1	63628	69587	-	2822
novel.3627	NW_016537283.1	169326	171348	+	1969
novel.3626	NW_016537278.1	13567	95851	-	492
novel.3625	NW_016537277.1	23976	24335	-	360
novel.3624	NW_016537265.1	16683	21867	-	235
novel.4055	NW_016538704.1	82247	84747	+	270
novel.4054	NW_016538695.1	13612	16382	+	301
novel.4057	NW_016538715.1	155170	162026	-	559
novel.4056	NW_016538715.1	18937	35736	-	660
novel.7722	NW_016570349.1	14348	16407	+	2012
novel.4050	NW_016538658.1	467587	468282	+	696
novel.4053	NW_016538673.1	389988	399026	+	215
novel.4052	NW_016538665.1	55285	56537	+	1197

novel.8257	NW_016587318.1	787	1097	-	256
novel.8256	NW_016587299.1	20	4559	-	2714
novel.8255	NW_016587267.1	1333	6718	+	1521
novel.8254	NW_016587217.1	11340	11801	-	462
novel.4059	NW_016538726.1	10359	10775	-	293
novel.4058	NW_016538716.1	15632	16885	-	1201
novel.8251	NW_016587126.1	214470	215639	-	1170
novel.8250	NW_016587126.1	197021	197611	-	224
novel.1647	NW_016531045.1	24620	66827	-	3206
novel.1646	NW_016531035.1	207636	246445	-	902
novel.1645	NW_016531035.1	207096	207579	-	484
novel.1644	NW_016531035.1	199735	204536	-	837
novel.1643	NW_016531035.1	157532	164896	-	257
novel.1642	NW_016531032.1	37099	38532	-	1034
novel.1641	NW_016531025.1	72008	109489	+	3644
novel.1640	NW_016531021.1	4	471	+	421
novel.7720	NW_016570306.1	316	590	-	239
novel.1138	NW_016529844.1	109696	110029	-	334
novel.7721	NW_016570336.1	4927	8069	-	424
novel.7726	NW_016570447.1	23874	24830	-	906
novel.7727	NW_016570454.1	327	578	-	207
novel.2282	NW_016532908.1	147290	147921	+	632
novel.2283	NW_016532908.1	148128	148757	+	397
novel.2280	NW_016532884.1	53237	53562	+	276
novel.2281	NW_016532884.1	12979	13295	-	317
novel.2286	NW_016532917.1	91663	92073	-	247
novel.2287	NW_016532926.1	98157	98468	+	246
novel.2284	NW_016532908.1	105014	107998	-	2985
novel.2285	NW_016532915.1	42803	43136	+	297
novel.5834	NW_016547565.1	314132	315105	-	974
novel.5835	NW_016547569.1	51031	51596	+	529
novel.2288	NW_016532930.1	183275	185497	+	2223
novel.2289	NW_016532930.1	129664	141950	-	434
novel.5830	NW_016547560.1	32875	51640	+	1819
novel.5831	NW_016547565.1	69482	69826	-	270
novel.5832	NW_016547565.1	161752	167107	-	4065
novel.5833	NW_016547565.1	212454	213228	-	262
novel.870	NW_016529257.1	140438	140867	+	430
novel.871	NW_016529257.1	234254	241568	+	2542
novel.872	NW_016529257.1	139520	140296	-	716
novel.873	NW_016529257.1	160084	161214	-	1131
novel.874	NW_016529261.1	183807	184034	+	228
novel.875	NW_016529267.1	395360	395660	+	301
novel.876	NW_016529267.1	249807	250208	-	352

novel.877	NW_016529273.1	241094	242087	-	785
novel.7724	NW_016570390.1	1	669	+	622
novel.879	NW_016529274.1	1379087	1379946	-	860
novel.7725	NW_016570440.1	6337	8859	+	611
novel.7889	NW_016575368.1	459	1328	-	813
novel.7888	NW_016575368.1	43173	46992	+	3297
novel.7883	NW_016575060.1	87952	88376	+	261
novel.7882	NW_016575044.1	2305	2968	-	623
novel.7881	NW_016574986.1	4816	5115	-	247
novel.7880	NW_016574981.1	488	1867	-	370
novel.7887	NW_016575368.1	459	1839	+	988
novel.7886	NW_016575363.1	398	999	+	545
novel.7885	NW_016575282.1	7476	12439	-	4964
novel.7884	NW_016575282.1	7476	12439	+	4964
novel.4047	NW_016538657.1	104381	104772	+	337
novel.2011	NW_016532056.1	328296	328863	+	402
novel.8274	NW_016587749.1	1	1488	-	297
novel.4613	NW_016540988.1	108488	113934	+	1196
novel.4612	NW_016540981.1	13082	26401	+	566
novel.4611	NW_016540978.1	643794	655977	-	1895
novel.4610	NW_016540978.1	383639	519901	-	258
novel.4617	NW_016540991.1	27348	34209	-	2552
novel.4616	NW_016540990.1	10319	10659	-	312
novel.4615	NW_016540988.1	258262	258523	-	210
novel.4614	NW_016540988.1	111979	112717	+	528
novel.4619	NW_016541002.1	129972	130433	+	261
novel.4618	NW_016540991.1	54661	55267	-	260
novel.6286	NW_016550687.1	544749	545507	+	707
novel.6287	NW_016550703.1	7353	8692	+	309
novel.6284	NW_016550643.1	381136	381646	+	457
novel.1068	NW_016529666.1	783376	786106	-	2731
novel.6282	NW_016550632.1	8805	9064	+	233
novel.6283	NW_016550640.1	3857	4116	-	260
novel.6280	NW_016550610.1	86650	88229	+	910
novel.6281	NW_016550631.1	19134	19786	+	653
novel.1063	NW_016529666.1	380179	382717	+	2539
novel.1062	NW_016529665.1	92857	101683	-	307
novel.1061	NW_016529665.1	443515	449208	+	1402
novel.1060	NW_016529665.1	415899	417602	+	263
novel.1067	NW_016529666.1	678256	683894	-	4161
novel.1066	NW_016529666.1	587001	598049	-	299
novel.1065	NW_016529666.1	384725	385212	-	226
novel.1064	NW_016529666.1	992058	999497	+	2725
novel.8278	NW_016587840.1	204	476	+	217

novel.4538	NW_016540697.1	19979	22730	+	1483
novel.4539	NW_016540698.1	18996	60171	-	366
novel.4536	NW_016540695.1	17769	19031	-	1206
novel.4537	NW_016540697.1	5251	12785	+	2504
novel.4534	NW_016540693.1	115954	116249	-	241
novel.4535	NW_016540694.1	105179	106832	-	600
novel.4532	NW_016540693.1	115223	116141	+	590
novel.4533	NW_016540693.1	67306	67560	-	255
novel.4530	NW_016540675.1	109233	111563	-	341
novel.4531	NW_016540691.1	2031	39842	+	348
novel.6758	NW_016555173.1	25218	26567	-	1324
novel.6759	NW_016555178.1	14261	19663	+	551
novel.6750	NW_016555103.1	161411	164333	+	2923
novel.6751	NW_016555103.1	16320	20026	-	1081
novel.6752	NW_016555128.1	76181	76839	+	564
novel.6753	NW_016555142.1	4556	7018	+	2269
novel.6754	NW_016555142.1	2610	6772	-	661
novel.6755	NW_016555145.1	115103	115553	+	244
novel.6756	NW_016555153.1	132600	135326	-	2152
novel.6757	NW_016555172.1	6701	25932	+	333
novel.1399	NW_016530375.1	268175	269243	+	260
novel.1398	NW_016530368.1	27720	28465	+	746
novel.1395	NW_016530356.1	237439	241578	-	656
novel.1394	NW_016530356.1	26052	30425	-	3053
novel.1397	NW_016530359.1	73302	74050	-	496
novel.1396	NW_016530356.1	723459	723870	-	412
novel.1391	NW_016530356.1	340743	341775	+	581
novel.1390	NW_016530356.1	49621	53111	+	3277
novel.1393	NW_016530356.1	697770	701241	+	2113
novel.1392	NW_016530356.1	651295	653348	+	649
novel.2350	NW_016533127.1	683650	685302	-	335
novel.649	NW_016528685.1	8976	9220	-	222
novel.648	NW_016528685.1	20403	22895	+	936
novel.647	NW_016528683.1	4035	15583	-	624
novel.646	NW_016528682.1	303030	307071	+	2948
novel.645	NW_016528682.1	300472	301028	+	452
novel.644	NW_016528681.1	6672	13297	-	720
novel.643	NW_016528681.1	13027	14261	+	1161
novel.642	NW_016528680.1	4852	18755	-	1135
novel.641	NW_016528677.1	31738	32341	-	517
novel.640	NW_016528677.1	30413	30992	+	580
novel.4280	NW_016539705.1	41970	42339	-	314
novel.4281	NW_016539709.1	23323	23605	+	232
novel.4282	NW_016539709.1	1	2838	-	637

novel.4283	NW_016539728.1	14322	15233	+	819
novel.4284	NW_016539728.1	16841	17641	+	772
novel.4285	NW_016539728.1	14322	17418	-	1061
novel.4286	NW_016539738.1	67183	77573	+	389
novel.4287	NW_016539739.1	206649	214756	+	760
novel.4288	NW_016539748.1	22829	23298	-	470
novel.4289	NW_016539748.1	23436	25958	-	485
novel.7588	NW_016567776.1	664	1173	+	433
novel.8630	NW_016603444.1	1	1172	-	462
novel.7589	NW_016567776.1	664	1313	-	618
novel.7399	NW_016564021.1	4089	9094	+	263
novel.7398	NW_016563994.1	5910	6974	+	1042
novel.7397	NW_016563986.1	1084	1464	+	346
novel.7396	NW_016563986.1	471	1483	+	1013
novel.7395	NW_016563974.1	29155	30855	+	1670
novel.7394	NW_016563947.1	466	924	-	412
novel.7393	NW_016563947.1	466	924	+	403
novel.7392	NW_016563928.1	8294	8539	-	215
novel.7391	NW_016563919.1	1	687	+	687
novel.7390	NW_016563915.1	454	1456	+	1003
novel.1939	NW_016531835.1	256966	257614	+	649
novel.1938	NW_016531835.1	201794	203369	+	265
novel.1931	NW_016531815.1	163611	164665	-	507
novel.1930	NW_016531802.1	164652	177840	+	278
novel.1933	NW_016531824.1	49184	117087	-	801
novel.1932	NW_016531824.1	193456	194181	+	603
novel.1935	NW_016531831.1	16424	16664	-	211
novel.1934	NW_016531826.1	51328	51659	-	332
novel.1937	NW_016531835.1	109267	111507	+	319
novel.1936	NW_016531835.1	8929	10792	+	1712
novel.5229	NW_016543794.1	7005	10087	+	3083
novel.5228	NW_016543776.1	142066	145534	-	3469
novel.5221	NW_016543734.1	107829	110366	+	417
novel.5220	NW_016543725.1	40776	45321	+	390
novel.5223	NW_016543752.1	202408	202855	+	397
novel.5222	NW_016543752.1	963	1359	+	350
novel.5225	NW_016543752.1	248756	249723	-	946
novel.5224	NW_016543752.1	203937	204351	+	204
novel.5227	NW_016543776.1	206965	213938	+	1123
novel.5226	NW_016543775.1	59920	64691	+	730
novel.2707	NW_016534133.1	70969	71272	+	304
novel.2706	NW_016534132.1	51094	61132	-	2298
novel.2705	NW_016534132.1	7344	7803	-	312
novel.2704	NW_016534132.1	61514	63208	+	1695

novel.2703	NW_016534132.1	54466	56216	+	1751
novel.2702	NW_016534130.1	23745	25094	+	1205
novel.2701	NW_016534130.1	22427	23653	+	1227
novel.2700	NW_016534124.1	35508	35809	-	258
novel.2879	NW_016534711.1	44653	45753	+	1044
novel.2878	NW_016534710.1	62982	87338	+	751
novel.2709	NW_016534146.1	23243	50918	+	568
novel.2708	NW_016534134.1	5034	5355	-	284
novel.7573	NW_016567544.1	60172	60471	-	300
novel.7572	NW_016567543.1	409	2735	+	2327
novel.7571	NW_016567540.1	210	462	+	206
novel.7570	NW_016567515.1	6330	7006	+	677
novel.7577	NW_016567590.1	818	1272	-	411
novel.7576	NW_016567573.1	114213	114726	-	380
novel.7575	NW_016567573.1	113803	115578	+	1482
novel.7574	NW_016567568.1	74479	75291	+	662
novel.7579	NW_016567641.1	3766	6068	+	1334
novel.7578	NW_016567611.1	60192	60596	+	315
novel.8628	NW_016603381.1	1453	2711	-	737
novel.8629	NW_016603418.1	1650	2626	+	294
novel.3559	NW_016537124.1	5219	12775	+	5594
novel.3558	NW_016537121.1	37904	38205	+	257
novel.7405	NW_016564071.1	366	2434	-	468
novel.7582	NW_016567646.1	5361	7043	-	1683
novel.7528	NW_016566668.1	4379	5500	-	1069
novel.7404	NW_016564041.1	1	919	-	919
novel.7583	NW_016567670.1	5882	7137	+	516
novel.6116	NW_016549619.1	84753	85014	-	262
novel.6117	NW_016549619.1	85129	86337	-	821
novel.6114	NW_016549619.1	143283	147112	+	3089
novel.6115	NW_016549619.1	227218	229759	+	2199
novel.6112	NW_016549610.1	22491	25729	+	377
novel.6113	NW_016549615.1	9597	14128	-	2419
novel.6110	NW_016549605.1	6688	12939	-	411
novel.6111	NW_016549606.1	468	811	-	287
novel.3478	NW_016536757.1	38918	40681	-	627
novel.6119	NW_016549619.1	143283	146270	-	2561
novel.2880	NW_016534729.1	280681	294507	+	3329
novel.2881	NW_016534740.1	58488	59336	+	432
novel.2882	NW_016534741.1	34728	82981	-	248
novel.2365	NW_016533160.1	240643	241016	+	257
novel.2883	NW_016534743.1	28424	30076	+	1606
novel.4848	NW_016541939.1	74071	74323	+	253
novel.5404	NW_016544889.1	184256	184837	+	582

novel.4846	NW_016541928.1	186344	226160	-	512
novel.2884	NW_016534744.1	3415	5018	+	1548
novel.4844	NW_016541928.1	9813	53024	+	7300
novel.4845	NW_016541928.1	21882	22277	-	396
novel.4842	NW_016541906.1	11700	12035	-	336
novel.4843	NW_016541915.1	49320	49826	+	507
novel.4840	NW_016541892.1	2916	30088	+	301
novel.2885	NW_016534744.1	3415	5539	-	984
novel.5406	NW_016544889.1	222864	243480	+	1477
novel.2886	NW_016534745.1	186560	194253	+	231
novel.2361	NW_016533154.1	84481	86653	-	200
novel.2887	NW_016534749.1	151	821	+	631
novel.2360	NW_016533148.1	281128	282537	-	938
novel.6893	NW_016556594.1	2779	14380	+	354
novel.7598	NW_016568031.1	23226	27264	+	620
novel.2363	NW_016533156.1	1961	4013	+	1708
novel.6892	NW_016556584.1	24179	27918	+	515
novel.2362	NW_016533156.1	1	1665	+	1665
novel.6891	NW_016556582.1	19734	21824	-	2061
novel.7400	NW_016564039.1	97066	105040	+	2565
novel.6890	NW_016556582.1	7719	12688	-	2580
novel.970	NW_016529462.1	115895	116268	-	210
novel.6897	NW_016556734.1	27493	31260	+	624
novel.6896	NW_016556633.1	1089	4259	+	3171
novel.6895	NW_016556631.1	52297	52794	-	441
novel.2369	NW_016533169.1	12116	13333	-	304
novel.6894	NW_016556631.1	28766	29821	+	848
novel.2368	NW_016533169.1	101063	105938	+	603
novel.6567	NW_016553335.1	641	1140	-	359
novel.6566	NW_016553333.1	3168	4454	-	1190
novel.6565	NW_016553333.1	4458	4739	+	230
novel.6564	NW_016553292.1	10799	13591	-	2793
novel.6563	NW_016553292.1	10434	13591	+	3158
novel.6562	NW_016553284.1	191569	191867	-	243
novel.6561	NW_016553284.1	209091	209969	+	879
novel.6560	NW_016553284.1	208623	223991	+	1150
novel.976	NW_016529485.1	41520	45186	-	340
novel.8673	NW_016604771.1	1705	3563	+	549
novel.6569	NW_016553374.1	60286	61340	-	369
novel.6568	NW_016553342.1	100707	102508	+	1378
novel.8672	NW_016604761.1	1	2677	-	2355
novel.8675	NW_016604822.1	903	2956	-	394
novel.8674	NW_016604815.1	1	3389	+	325
novel.8677	NW_016604840.1	1246	3611	-	409

novel.8676	NW_016604830.1	1	3624	-	1053
novel.6826	NW_016555835.1	15764	17185	-	1422
novel.7520	NW_016566478.1	4891	5118	+	207
novel.6827	NW_016555835.1	16638	17185	-	548
novel.7521	NW_016566482.1	1123	2748	+	1580
novel.1236	NW_016530032.1	66946	67859	-	864
novel.1237	NW_016530032.1	223122	235114	-	1098
novel.1234	NW_016530032.1	175319	179904	+	425
novel.1235	NW_016530032.1	180701	183252	+	1183
novel.1232	NW_016530032.1	66946	68031	+	1086
novel.1233	NW_016530032.1	163947	175190	+	1497
novel.1230	NW_016530028.1	93731	102111	-	6612
novel.1231	NW_016530029.1	436604	437057	+	418
novel.1728	NW_016531309.1	51142	61106	+	939
novel.1825	NW_016531532.1	41195	190118	-	703
novel.1238	NW_016530037.1	174657	180691	-	342
novel.1239	NW_016530039.1	344964	345314	+	281
novel.5034	NW_016542708.1	30660	30933	-	274
novel.5035	NW_016542716.1	3174	10161	+	1994
novel.5036	NW_016542716.1	67321	68899	+	1579
novel.1352	NW_016530274.1	295185	296802	+	366
novel.5030	NW_016542700.1	64924	71609	-	3098
novel.5031	NW_016542702.1	30103	31170	-	548
novel.5032	NW_016542702.1	176884	177476	-	367
novel.5033	NW_016542708.1	27708	28336	+	574
novel.1355	NW_016530278.1	77232	86507	+	914
novel.5038	NW_016542716.1	46887	47634	-	463
novel.4031	NW_016538604.1	562	7275	+	222
novel.7523	NW_016566552.1	4665	5742	-	508
novel.1354	NW_016530276.1	3544	6393	+	522
novel.8588	NW_016602306.1	1	2288	+	2288
novel.1357	NW_016530287.1	227559	228673	-	1065
novel.1356	NW_016530282.1	67694	69514	+	685
novel.4193	NW_016539320.1	309050	309952	-	480
novel.8097	NW_016582221.1	514	1006	+	493
novel.7524	NW_016566599.1	44091	44675	+	543
novel.3285	NW_016536067.1	538989	539759	-	273
novel.4467	NW_016540462.1	221079	232142	-	1258
novel.3936	NW_016538321.1	192379	201300	+	8871
novel.8581	NW_016601873.1	1	2167	+	2167
novel.8580	NW_016601861.1	814	2102	+	498
novel.258	NW_016527842.1	241611	267636	+	1487
novel.5947	NW_016548399.1	124736	125143	+	366
novel.254	NW_016527838.1	152309	152854	+	546

novel.255	NW_016527838.1	83310	95268	-	331
novel.7525	NW_016566599.1	40578	42936	-	826
novel.257	NW_016527842.1	148996	160432	+	466
novel.250	NW_016527824.1	214670	214960	+	291
novel.6279	NW_016550602.1	2979	24337	-	764
novel.252	NW_016527824.1	326926	329575	-	2650
novel.253	NW_016527828.1	13353	13616	-	209
novel.6278	NW_016550601.1	192739	193624	-	695
novel.3931	NW_016538302.1	7660	13236	+	1408
novel.3930	NW_016538294.1	11914	38327	+	236
novel.7526	NW_016566635.1	15048	15408	-	281
novel.4469	NW_016540477.1	10387	10659	+	220
novel.7527	NW_016566647.1	1	1128	+	1128
novel.6277	NW_016550600.1	137354	140733	-	2495
novel.6276	NW_016550600.1	93080	97640	-	2108
novel.8019	NW_016579365.1	10076	10316	+	241
novel.8018	NW_016579365.1	2483	3235	+	696
novel.7768	NW_016571870.1	1	1526	-	535
novel.7769	NW_016571975.1	2760	5860	+	551
novel.8015	NW_016579231.1	72904	73222	+	297
novel.8014	NW_016579226.1	24849	27610	-	2077
novel.8017	NW_016579264.1	3502	3969	-	427
novel.8016	NW_016579231.1	71619	72126	-	454
novel.8011	NW_016579074.1	742	1536	+	738
novel.8010	NW_016579004.1	43979	46719	+	1687
novel.8013	NW_016579122.1	755	7279	-	6525
novel.7761	NW_016571581.1	1	3172	-	468
novel.3869	NW_016538058.1	86834	87491	+	625
novel.3868	NW_016538058.1	56836	62806	+	5934
novel.3649	NW_016537358.1	25708	29434	-	1978
novel.3648	NW_016537349.1	11140	35206	+	291
novel.3645	NW_016537336.1	747	1039	-	259
novel.3644	NW_016537330.1	57085	57986	+	590
novel.3647	NW_016537348.1	94115	139841	+	450
novel.3646	NW_016537337.1	143653	143910	-	258
novel.3641	NW_016537327.1	51578	52844	+	1232
novel.3640	NW_016537318.1	383032	383693	-	662
novel.3643	NW_016537329.1	60248	83847	+	903
novel.3642	NW_016537327.1	2198	4040	-	205
novel.4198	NW_016539336.1	184276	188305	+	543
novel.4079	NW_016538813.1	413771	416112	-	833
novel.4078	NW_016538813.1	260518	288805	-	459
novel.4077	NW_016538813.1	175279	180709	-	468
novel.4076	NW_016538802.1	526709	528405	-	308

novel.4075	NW_016538802.1	1894	16132	+	1031
novel.4074	NW_016538792.1	3104	9378	-	803
novel.4073	NW_016538788.1	1215	1737	+	480
novel.4072	NW_016538782.1	457748	458238	+	491
novel.4071	NW_016538778.1	31178	31645	+	427
novel.4070	NW_016538778.1	19689	20293	+	559
novel.4199	NW_016539336.1	9239	9963	-	725
novel.8734	NW_016605845.1	4391	4813	-	423
novel.8735	NW_016605873.1	967	1493	-	266
novel.8736	NW_016605877.1	1268	4873	+	1752
novel.8737	NW_016605883.1	1670	3021	+	279
novel.8730	NW_016605801.1	80	1455	+	584
novel.8731	NW_016605804.1	1	2310	-	1822
novel.7188	NW_016560192.1	18806	19645	+	786
novel.7189	NW_016560193.1	8655	12763	-	1464
novel.8732	NW_016605834.1	3096	4788	-	1693
novel.7182	NW_016559931.1	734	1914	+	1134
novel.7183	NW_016560074.1	19684	24654	-	1707
novel.7180	NW_016559915.1	105	648	-	521
novel.7170	NW_016559812.1	149176	152080	-	412
novel.7186	NW_016560161.1	171062	175676	-	751
novel.7187	NW_016560163.1	6848	8546	+	298
novel.7184	NW_016560106.1	17888	64967	+	346
novel.7185	NW_016560161.1	174705	175676	+	972
novel.2112	NW_016532271.1	19074	61659	+	859
novel.2113	NW_016532272.1	568451	568704	+	217
novel.2110	NW_016532267.1	43201	43434	+	205
novel.2111	NW_016532269.1	21785	26696	+	4658
novel.1087	NW_016529719.1	120873	127572	+	6700
novel.2117	NW_016532280.1	293810	294299	-	366
novel.2114	NW_016532274.1	349639	350324	+	337
novel.2115	NW_016532274.1	356017	375349	+	2005
novel.2118	NW_016532282.1	143903	144181	-	233
novel.2119	NW_016532296.1	18141	18677	+	310
novel.5856	NW_016547740.1	15808	23064	-	1430
novel.5857	NW_016547742.1	8352	9401	-	966
novel.5854	NW_016547732.1	21568	24650	+	1211
novel.5855	NW_016547732.1	31080	33347	+	234
novel.5852	NW_016547671.1	184223	185123	-	380
novel.2259	NW_016532839.1	4982	6816	-	255
novel.5850	NW_016547671.1	184235	185079	+	845
novel.5851	NW_016547671.1	833	1323	-	491
novel.5588	NW_016545968.1	3280	29049	-	519
novel.5589	NW_016545969.1	18042	29409	-	3031

novel.5858	NW_016547754.1	71618	76304	-	288
novel.5859	NW_016547760.1	7294	36239	+	603
novel.816	NW_016529145.1	9701	9977	+	257
novel.817	NW_016529146.1	21479	22657	-	419
novel.814	NW_016529136.1	76009	77455	-	709
novel.815	NW_016529139.1	20418	45038	-	450
novel.812	NW_016529136.1	5338	9404	-	3118
novel.813	NW_016529136.1	83378	84041	-	474
novel.810	NW_016529136.1	5338	8667	+	1688
novel.811	NW_016529136.1	121514	123728	+	382
novel.818	NW_016529150.1	3192	3986	-	574
novel.819	NW_016529152.1	11753	19951	-	565
novel.4983	NW_016542510.1	134632	137005	+	2374
novel.4982	NW_016542510.1	61309	61579	+	271
novel.4981	NW_016542507.1	41061	41393	-	283
novel.4980	NW_016542507.1	3582	5408	-	491
novel.4987	NW_016542534.1	35111	36099	-	470
novel.4986	NW_016542530.1	182370	182833	+	232
novel.4985	NW_016542528.1	1377580	1392891	+	355
novel.4984	NW_016542510.1	213587	214971	-	1328
novel.4989	NW_016542554.1	836	1111	+	225
novel.4988	NW_016542536.1	2278	2610	-	280
novel.66	NW_016527381.1	564748	583890	+	599
novel.4675	NW_016541286.1	156690	158890	+	424
novel.4674	NW_016541284.1	11481	35966	-	266
novel.4677	NW_016541286.1	263238	265008	-	308
novel.4676	NW_016541286.1	255341	255997	+	512
novel.4671	NW_016541259.1	504117	505240	-	1124
novel.4670	NW_016541259.1	590633	601997	+	736
novel.4673	NW_016541281.1	7152	10444	+	3129
novel.4672	NW_016541261.1	51682	59579	+	569
novel.4679	NW_016541291.1	116418	118873	+	2400
novel.4678	NW_016541288.1	53184	65367	-	719
novel.1009	NW_016529569.1	38638	41681	-	2803
novel.1008	NW_016529567.1	139234	140574	-	1312
novel.1005	NW_016529567.1	73690	101342	+	1646
novel.1004	NW_016529563.1	105573	106120	-	501
novel.1007	NW_016529567.1	363044	368320	+	2464
novel.1006	NW_016529567.1	134451	138600	+	259
novel.1001	NW_016529559.1	151815	162144	-	208
novel.1000	NW_016529555.1	44977	52078	+	5944
novel.1003	NW_016529561.1	147899	200968	+	277
novel.1002	NW_016529559.1	252646	254759	-	247
novel.4510	NW_016540604.1	29233	29890	-	614

novel.4511	NW_016540605.1	160170	291536	-	822
novel.4512	NW_016540611.1	161745	164249	+	2505
novel.4513	NW_016540627.1	175504	176805	+	1245
novel.4514	NW_016540627.1	180688	180998	+	272
novel.4515	NW_016540627.1	174891	175741	-	798
novel.4516	NW_016540629.1	161831	170760	+	3848
novel.4517	NW_016540629.1	131985	135528	-	3490
novel.4518	NW_016540629.1	135698	136819	-	355
novel.4519	NW_016540629.1	142936	147143	-	3761
novel.625	NW_016528633.1	275787	285723	+	4855
novel.624	NW_016528633.1	133703	134344	+	467
novel.627	NW_016528634.1	5019	5414	+	342
novel.626	NW_016528634.1	1395	1631	+	237
novel.621	NW_016528630.1	271596	275412	-	349
novel.620	NW_016528630.1	231962	233521	-	1560
novel.623	NW_016528633.1	132220	134883	+	577
novel.622	NW_016528632.1	3076	4562	-	336
novel.629	NW_016528645.1	106649	107191	-	498
novel.628	NW_016528637.1	8073	47251	+	801
novel.6149	NW_016549859.1	21640	23381	+	1707
novel.6148	NW_016549859.1	14716	15542	+	392
novel.7371	NW_016563503.1	1	2665	+	2665
novel.7370	NW_016563475.1	1749	9369	-	350
novel.7373	NW_016563557.1	77007	79606	+	2600
novel.7372	NW_016563503.1	1	7062	-	2344
novel.7375	NW_016563624.1	64933	66638	+	701
novel.7374	NW_016563582.1	6026	8140	-	298
novel.7377	NW_016563624.1	69291	73008	+	1207
novel.7376	NW_016563624.1	67487	68465	+	979
novel.7379	NW_016563638.1	5038	15244	+	818
novel.7378	NW_016563624.1	55842	56486	-	427
novel.1913	NW_016531747.1	194755	197929	+	362
novel.1912	NW_016531746.1	57875	127569	+	245
novel.1911	NW_016531738.1	20935	21697	-	710
novel.1910	NW_016531738.1	97323	97639	+	263
novel.1917	NW_016531767.1	14009	30229	-	4925
novel.1916	NW_016531767.1	47293	48858	+	1512
novel.1915	NW_016531760.1	251129	267789	+	216
novel.1914	NW_016531751.1	194436	195624	+	1189
novel.1919	NW_016531767.1	81485	81824	-	317
novel.1918	NW_016531767.1	62093	75393	-	1431
novel.5203	NW_016543639.1	2778	3141	-	307
novel.3421	NW_016536554.1	296461	313700	-	436
novel.5201	NW_016543635.1	9872	10863	-	938

novel.5200	NW_016543635.1	8964	10247	+	1202
novel.5207	NW_016543659.1	57806	128878	+	375
novel.5206	NW_016543656.1	2619	3337	-	667
novel.5205	NW_016543650.1	2507	3947	-	1386
novel.3420	NW_016536550.1	28799	35506	-	231
novel.5209	NW_016543676.1	6427	16492	-	271
novel.5208	NW_016543664.1	8540	25745	+	313
novel.6143	NW_016549793.1	395729	396237	-	456
novel.8608	NW_016602748.1	1	2442	+	2442
novel.8609	NW_016602748.1	1	2442	-	2442
novel.8358	NW_016591244.1	13	4600	-	265
novel.8359	NW_016591247.1	1672	5713	-	1026
novel.8600	NW_016602618.1	1227	2103	-	516
novel.8353	NW_016591146.1	30479	32133	-	1292
novel.8602	NW_016602645.1	1146	2409	+	1044
novel.8603	NW_016602656.1	1	1547	-	1547
novel.8604	NW_016602696.1	1090	2094	+	267
novel.8605	NW_016602711.1	1	2411	+	2149
novel.8606	NW_016602726.1	1	2436	-	2436
novel.8355	NW_016591168.1	1301	1683	+	327
novel.7515	NW_016566382.1	105	581	-	477
novel.7514	NW_016566354.1	3587	4537	-	239
novel.7517	NW_016566456.1	912	1177	-	241
novel.7516	NW_016566398.1	123	5419	-	399
novel.7511	NW_016566211.1	260725	265456	+	4732
novel.7510	NW_016566201.1	25662	26455	-	526
novel.7513	NW_016566242.1	88445	90218	-	1351
novel.7512	NW_016566242.1	29313	31209	+	812
novel.6146	NW_016549827.1	6314	7083	-	713
novel.7519	NW_016566477.1	103702	130349	+	257
novel.7518	NW_016566467.1	1	1028	-	997
novel.3988	NW_016538461.1	16744	17137	+	394
novel.3989	NW_016538461.1	48807	119542	+	1296
novel.3986	NW_016538453.1	492	1424	+	701
novel.3987	NW_016538453.1	1	1424	-	1335
novel.3984	NW_016538432.1	43718	44324	+	607
novel.3985	NW_016538443.1	2244	20771	+	420
novel.3982	NW_016538428.1	135661	139743	+	577
novel.3983	NW_016538431.1	2362	3056	+	472
novel.3980	NW_016538422.1	6954	9709	+	901
novel.3981	NW_016538428.1	51329	52310	+	559
novel.3186	NW_016535741.1	5945	7903	+	1939
novel.3187	NW_016535741.1	26113	30214	-	3853
novel.3184	NW_016535736.1	48261	48673	+	393

novel.3185	NW_016535736.1	5365	6165	-	442
novel.3182	NW_016535735.1	19210	21044	-	498
novel.3183	NW_016535736.1	22800	26968	+	4143
novel.3180	NW_016535732.1	242912	243652	-	698
novel.3181	NW_016535733.1	85533	87793	+	225
novel.3188	NW_016535742.1	103637	104079	+	213
novel.3189	NW_016535743.1	61168	63833	-	2610
novel.2859	NW_016534647.1	253428	309407	-	691
novel.2858	NW_016534647.1	83821	88015	-	210
novel.2853	NW_016534626.1	9409	9740	-	281
novel.2852	NW_016534625.1	25871	26266	+	396
novel.2851	NW_016534622.1	8135	57937	-	736
novel.2850	NW_016534614.1	193241	219384	-	277
novel.2857	NW_016534647.1	68532	80194	-	602
novel.2856	NW_016534639.1	6804	7066	-	217
novel.2855	NW_016534639.1	13533	14164	+	632
novel.2854	NW_016534630.1	302414	306050	-	295
novel.6138	NW_016549765.1	66307	67761	+	402
novel.6139	NW_016549765.1	77062	81590	+	568
novel.3328	NW_016536243.1	71582	77643	-	5975
novel.3329	NW_016536246.1	195030	195525	-	439
novel.3322	NW_016536225.1	531897	532708	+	603
novel.3323	NW_016536225.1	529100	529472	-	339
novel.3320	NW_016536208.1	88487	98524	-	234
novel.3453	NW_016536661.1	66954	68149	-	1104
novel.3454	NW_016536661.1	79560	80659	-	923
novel.3455	NW_016536672.1	55488	58090	-	1566
novel.3456	NW_016536678.1	82962	85583	+	264
novel.3457	NW_016536678.1	90479	99111	+	3101
novel.8659	NW_016604519.1	35	3378	+	3344
novel.2589	NW_016533809.1	8537	8791	+	255
novel.2588	NW_016533808.1	23923	24414	-	245
novel.2585	NW_016533795.1	2238	10672	-	803
novel.2584	NW_016533795.1	28409	33350	+	1076
novel.2587	NW_016533807.1	117908	124387	-	5060
novel.2586	NW_016533805.1	414529	414765	+	237
novel.2581	NW_016533775.1	150891	151630	-	589
novel.2580	NW_016533775.1	343938	344769	+	832
novel.2583	NW_016533775.1	334401	349814	-	3188
novel.2582	NW_016533775.1	157604	158371	-	768
novel.4860	NW_016541994.1	154345	154931	+	543
novel.4861	NW_016541994.1	131352	134323	-	1399
novel.4862	NW_016541994.1	179433	180434	-	554
novel.4863	NW_016542010.1	23417	24014	+	545

novel.4864	NW_016542010.1	23417	24547	-	454
novel.4865	NW_016542021.1	750	1510	-	761
novel.4866	NW_016542025.1	225168	226572	+	1254
novel.4867	NW_016542025.1	226444	226872	-	234
novel.4868	NW_016542027.1	22226	22880	-	224
novel.4869	NW_016542028.1	17938	33585	-	307
novel.5548	NW_016545607.1	28247	29806	-	232
novel.5549	NW_016545607.1	54022	58161	-	564
novel.2260	NW_016532839.1	221629	299319	-	360
novel.5813	NW_016547467.1	304020	305705	-	448
novel.2619	NW_016533879.1	5748	9618	-	421
novel.2262	NW_016532844.1	205337	205753	-	246
novel.2618	NW_016533876.1	87277	93433	+	288
novel.5449	NW_016545122.1	11065	11420	-	301
novel.5448	NW_016545110.1	702065	703272	-	342
novel.2263	NW_016532846.1	4560	5165	-	606
novel.5441	NW_016545089.1	116185	117464	-	969
novel.2264	NW_016532848.1	58778	60230	+	883
novel.2323	NW_016533047.1	102431	104899	-	1147
novel.2322	NW_016533039.1	203976	204473	-	309
novel.2325	NW_016533052.1	229759	230006	+	248
novel.2324	NW_016533051.1	65493	150290	+	499
novel.2327	NW_016533055.1	30984	31882	+	791
novel.5817	NW_016547480.1	69865	70838	-	541
novel.2615	NW_016533870.1	39310	39878	+	362
novel.2266	NW_016532849.1	881817	905549	+	268
novel.2614	NW_016533861.1	422301	422765	+	430
novel.2267	NW_016532849.1	1	6990	-	1237
novel.2617	NW_016533873.1	203830	204456	+	575
novel.6456	NW_016552279.1	39120	39744	-	403
novel.2616	NW_016533872.1	44011	45100	-	880
novel.2611	NW_016533856.1	13170	13655	+	397
novel.2610	NW_016533856.1	2877	3833	+	905
novel.7902	NW_016575601.1	17595	17880	+	265
novel.2613	NW_016533861.1	193956	194278	+	224
novel.7903	NW_016575601.1	18845	19326	+	406
novel.2612	NW_016533856.1	2648	13076	-	1086
novel.7900	NW_016575598.1	312	902	+	536
novel.7901	NW_016575601.1	16909	18406	+	1498
novel.7906	NW_016575708.1	1263	1514	-	221
novel.7907	NW_016575754.1	955	2224	-	374
novel.7904	NW_016575614.1	19869	20182	+	267
novel.7905	NW_016575626.1	1525	2242	+	665
novel.1218	NW_016529957.1	312018	312387	-	314

novel.7300	NW_016562350.1	35833	36733	+	742
novel.1748	NW_016531371.1	131871	133636	+	1406
novel.7301	NW_016562350.1	8788	19038	-	1094
novel.1210	NW_016529952.1	13071	13495	-	425
novel.1211	NW_016529952.1	15897	44996	-	538
novel.1744	NW_016531360.1	3894	5411	+	616
novel.1745	NW_016531364.1	20522	20960	-	439
novel.1214	NW_016529955.1	16652	24067	-	809
novel.8163	NW_016584516.1	22528	22800	+	273
novel.1216	NW_016529955.1	74934	75938	-	948
novel.1741	NW_016531357.1	39190	39595	-	260
novel.7201	NW_016560470.1	4255	7321	-	3067
novel.7651	NW_016568958.1	23495	25959	-	1350
novel.5058	NW_016542855.1	60368	63482	-	376
novel.5059	NW_016542856.1	7892	9409	+	1427
novel.5056	NW_016542842.1	93866	100373	-	533
novel.7656	NW_016569002.1	1	325	-	325
novel.5054	NW_016542835.1	58453	62118	+	502
novel.5055	NW_016542842.1	366571	369689	+	271
novel.5052	NW_016542809.1	11030	12035	-	1006
novel.5053	NW_016542814.1	597094	605211	-	281
novel.5050	NW_016542809.1	20189	20565	+	323
novel.8164	NW_016584516.1	43342	43905	+	430
novel.7202	NW_016560503.1	3631	4908	+	1225
novel.7306	NW_016562469.1	2	336	+	335
novel.7205	NW_016560537.1	80190	80526	-	236
novel.7307	NW_016562473.1	8320	10062	+	1192
novel.7204	NW_016560507.1	9160	9467	-	258
novel.8169	NW_016584727.1	1	1073	-	1018
novel.7207	NW_016560555.1	13889	15553	+	1665
novel.8168	NW_016584712.1	846	1174	-	272
novel.7206	NW_016560538.1	101	1180	+	972
novel.7660	NW_016569071.1	466	1047	-	551
novel.1755	NW_016531376.1	9844	10673	+	810
novel.1517	NW_016530674.1	298713	300806	+	321
novel.1516	NW_016530673.1	29746	29990	-	209
novel.1515	NW_016530673.1	29339	29622	-	284
novel.1514	NW_016530672.1	204392	322129	+	288
novel.1513	NW_016530669.1	414307	414872	+	566
novel.1512	NW_016530668.1	24135	36584	-	273
novel.1511	NW_016530664.1	672383	686526	+	768
novel.7665	NW_016569214.1	1040	1540	-	477
novel.1510	NW_016530664.1	484800	489826	+	5027
novel.8037	NW_016580009.1	4233	5249	+	1017

novel.8036	NW_016580005.1	100	775	+	529
novel.8035	NW_016579977.1	6894	9232	+	834
novel.8034	NW_016579959.1	5422	5727	-	273
novel.8033	NW_016579920.1	1786	2057	+	245
novel.8032	NW_016579887.1	30252	32412	-	2161
novel.8031	NW_016579887.1	27770	28645	-	672
novel.8030	NW_016579887.1	27639	27908	+	228
novel.7748	NW_016571133.1	547	830	+	255
novel.7749	NW_016571133.1	1048	2243	+	1176
novel.8039	NW_016580017.1	636	958	-	267
novel.8038	NW_016580009.1	9555	14389	+	396
novel.3667	NW_016537429.1	13384	15624	+	2211
novel.3666	NW_016537429.1	987	1708	+	519
novel.3665	NW_016537426.1	135794	163460	-	926
novel.3840	NW_016537933.1	151102	151488	+	333
novel.3663	NW_016537405.1	55683	56097	+	360
novel.3662	NW_016537403.1	133378	135608	+	203
novel.6837	NW_016555951.1	13934	17253	-	439
novel.6836	NW_016555951.1	12583	16032	-	545
novel.6839	NW_016555954.1	191467	192146	+	659
novel.6838	NW_016555954.1	49572	50440	+	819
novel.3849	NW_016537972.1	221552	222687	+	1136
novel.3848	NW_016537970.1	21646	21975	+	278
novel.3669	NW_016537435.1	82737	82994	-	258
novel.3668	NW_016537435.1	140809	151827	+	824
novel.4091	NW_016538866.1	153646	154396	-	699
novel.4090	NW_016538866.1	138798	152085	+	3260
novel.8299	NW_016588978.1	2696	3198	-	341
novel.8298	NW_016588972.1	5196	6080	-	885
novel.4095	NW_016538874.1	407137	416654	-	469
novel.4094	NW_016538874.1	416772	424746	+	253
novel.4097	NW_016538895.1	97009	102529	+	1261
novel.4096	NW_016538886.1	14725	16008	+	1006
novel.8293	NW_016588824.1	3002	4913	+	622
novel.4098	NW_016538908.1	7055	7361	-	268
novel.8291	NW_016588720.1	136	690	+	501
novel.8290	NW_016588697.1	81934	83385	-	1207
novel.8297	NW_016588926.1	6185	6447	-	207
novel.8296	NW_016588899.1	1	841	+	765
novel.8295	NW_016588845.1	17817	18615	+	449
novel.8294	NW_016588824.1	12885	14187	+	769
novel.6557	NW_016553260.1	580	830	-	251
novel.6551	NW_016553220.1	297982	298275	+	294
novel.2928	NW_016534911.1	11437	11744	-	251

novel.2134	NW_016532352.1	105	28328	-	404
novel.2135	NW_016532354.1	16325	23520	+	391
novel.2136	NW_016532375.1	228	574	+	347
novel.2137	NW_016532378.1	120331	120634	+	256
novel.2130	NW_016532350.1	16064	17260	+	723
novel.2131	NW_016532350.1	17826	41141	+	544
novel.2132	NW_016532350.1	238	1009	-	632
novel.2133	NW_016532352.1	28923	29297	+	258
novel.2138	NW_016532378.1	3138	112153	-	1579
novel.2139	NW_016532385.1	85105	85339	-	209
novel.7505	NW_016566125.1	18092	19412	-	1321
novel.5878	NW_016547912.1	51308	52217	+	910
novel.5879	NW_016547959.1	339663	340788	-	1076
novel.5870	NW_016547846.1	194193	197432	-	1584
novel.5871	NW_016547848.1	17469	17786	-	261
novel.5872	NW_016547850.1	5309	6515	+	232
novel.5873	NW_016547871.1	103412	104554	-	209
novel.5874	NW_016547871.1	219492	244826	-	329
novel.5875	NW_016547875.1	68931	69250	+	267
novel.5876	NW_016547878.1	190992	191834	+	220
novel.5877	NW_016547899.1	33634	33951	-	268
novel.838	NW_016529191.1	41389	61220	+	644
novel.839	NW_016529192.1	167878	171743	+	465
novel.7502	NW_016566093.1	316974	336571	+	635
novel.835	NW_016529190.1	465937	466964	-	290
novel.836	NW_016529190.1	887938	889455	-	1078
novel.837	NW_016529191.1	15501	28648	+	4849
novel.830	NW_016529183.1	22590	23387	+	741
novel.831	NW_016529190.1	458170	458533	+	318
novel.832	NW_016529190.1	715864	717944	+	942
novel.833	NW_016529190.1	859743	870102	+	1076
novel.1897	NW_016531704.1	496419	496769	-	351
novel.8050	NW_016580488.1	4	1096	+	1093
novel.4659	NW_016541191.1	3251	3597	+	214
novel.4658	NW_016541171.1	68970	69522	-	553
novel.4657	NW_016541168.1	71880	142340	-	502
novel.4656	NW_016541160.1	62137	64373	-	975
novel.4655	NW_016541147.1	324550	325335	-	428
novel.4654	NW_016541147.1	323651	324126	-	422
novel.4653	NW_016541147.1	251923	253338	-	1317
novel.4652	NW_016541147.1	93099	116251	-	561
novel.4651	NW_016541147.1	325397	326365	+	921
novel.4650	NW_016541147.1	324294	324655	+	306
novel.6242	NW_016550402.1	343	855	-	378

novel.6243	NW_016550424.1	54913	56658	-	1537
novel.6240	NW_016550345.1	3002	7347	+	1872
novel.6241	NW_016550361.1	1612	2384	-	725
novel.6246	NW_016550448.1	37072	37998	-	927
novel.6247	NW_016550449.1	65933	67341	+	1179
novel.6244	NW_016550431.1	6002	18861	+	361
novel.6245	NW_016550448.1	124043	125394	+	1063
novel.6248	NW_016550449.1	69602	79720	-	1122
novel.6249	NW_016550450.1	13183	13820	+	335
novel.1027	NW_016529600.1	148023	150573	-	298
novel.1026	NW_016529600.1	41955	64899	-	479
novel.1025	NW_016529600.1	65617	150624	+	972
novel.1024	NW_016529600.1	58763	59516	+	754
novel.1023	NW_016529597.1	11311	12009	-	642
novel.1022	NW_016529596.1	156254	156675	+	347
novel.1021	NW_016529595.1	49273	55750	+	556
novel.1020	NW_016529593.1	433563	437618	+	1797
novel.1029	NW_016529603.1	250627	313851	-	2312
novel.1028	NW_016529603.1	414180	414706	+	321
novel.6794	NW_016555514.1	8524	9158	-	635
novel.6795	NW_016555565.1	1	305	+	305
novel.6796	NW_016555574.1	10186	11300	+	310
novel.6797	NW_016555606.1	77645	79914	+	529
novel.6790	NW_016555462.1	1725	2783	-	977
novel.6791	NW_016555491.1	22396	22920	+	317
novel.6792	NW_016555498.1	13	707	+	695
novel.6793	NW_016555514.1	8524	9207	+	556
novel.6798	NW_016555606.1	63705	78427	-	889
novel.6799	NW_016555608.1	77385	95055	-	274
novel.7221	NW_016560913.1	3463	4638	+	321
novel.1683	NW_016531206.1	71724	72238	-	335
novel.1682	NW_016531206.1	76664	79456	+	2320
novel.1681	NW_016531200.1	8706	11670	-	703
novel.1680	NW_016531198.1	22445	24496	-	1519
novel.1687	NW_016531209.1	69647	97974	-	875
novel.1686	NW_016531209.1	235724	238543	+	763
novel.1685	NW_016531207.1	318910	319374	+	292
novel.1684	NW_016531206.1	185861	187104	-	843
novel.1689	NW_016531224.1	64210	65492	-	293
novel.1688	NW_016531220.1	9732	10112	-	325
novel.8198	NW_016586006.1	1	1713	-	576
novel.8199	NW_016586097.1	82	1287	-	886
novel.7359	NW_016563317.1	5606	6491	+	886
novel.7358	NW_016563311.1	2779	4684	-	1722

novel.8190	NW_016585484.1	2830	5110	-	263
novel.8191	NW_016585623.1	257	1071	+	758
novel.8192	NW_016585639.1	877	1320	-	400
novel.8193	NW_016585705.1	1	203	-	203
novel.8194	NW_016585705.1	4903	5129	-	227
novel.8195	NW_016585782.1	10088	12914	-	2827
novel.8196	NW_016585875.1	16368	23622	+	324
novel.8197	NW_016585980.1	5004	8550	-	217
novel.7685	NW_016569592.1	21854	22381	+	422
novel.7684	NW_016569586.1	1	469	+	469
novel.7687	NW_016569617.1	264	1113	-	850
novel.7686	NW_016569594.1	23782	25630	+	393
novel.7681	NW_016569528.1	1	1225	+	1178
novel.7680	NW_016569493.1	4796	5404	-	556
novel.7683	NW_016569570.1	1	2313	-	2281
novel.7682	NW_016569531.1	2084	2539	-	456
novel.7689	NW_016569785.1	6908	7216	+	288
novel.7688	NW_016569660.1	16772	17389	-	213
novel.1975	NW_016531946.1	133371	133628	+	206
novel.1974	NW_016531945.1	4852	5286	+	378
novel.1977	NW_016531953.1	3595	4009	+	415
novel.1976	NW_016531946.1	133760	134253	+	494
novel.1971	NW_016531942.1	163126	164594	+	1432
novel.1970	NW_016531931.1	342385	344190	+	1806
novel.1973	NW_016531942.1	157372	164594	-	5659
novel.1972	NW_016531942.1	59772	60194	-	344
novel.1979	NW_016531955.1	169187	170719	+	1533
novel.1978	NW_016531955.1	13688	16522	+	1165
novel.359	NW_016528048.1	214518	216325	-	707
novel.5264	NW_016543970.1	390	850	+	404
novel.609	NW_016528580.1	579547	581433	-	1776
novel.608	NW_016528580.1	579554	581230	+	1677
novel.5261	NW_016543961.1	835	95721	-	669
novel.5260	NW_016543951.1	7944	8245	-	246
novel.5263	NW_016543962.1	22693	25795	-	639
novel.5262	NW_016543962.1	308797	309119	+	266
novel.351	NW_016528037.1	157777	158395	-	562
novel.350	NW_016528037.1	3059	3406	-	292
novel.601	NW_016528579.1	6757	14642	+	552
novel.600	NW_016528577.1	730	1723	-	606
novel.355	NW_016528039.1	297453	297827	+	375
novel.354	NW_016528038.1	72617	73052	-	387
novel.605	NW_016528579.1	201796	205617	+	1344
novel.604	NW_016528579.1	55172	70527	+	241

novel.8378	NW_016591698.1	18801	19523	-	667
novel.8379	NW_016591699.1	948	1697	-	273
novel.7539	NW_016566836.1	19345	23093	+	3728
novel.7538	NW_016566832.1	6025	6711	+	687
novel.7537	NW_016566813.1	2435	13483	+	412
novel.8375	NW_016591656.1	639	986	+	291
novel.8376	NW_016591665.1	1	28636	-	513
novel.8377	NW_016591698.1	527	1343	+	817
novel.8370	NW_016591563.1	9637	12126	-	1629
novel.8371	NW_016591600.1	3865	7790	+	3926
novel.8372	NW_016591635.1	570	844	-	275
novel.8373	NW_016591651.1	7	1198	+	1138
novel.1406	NW_016530389.1	2893	3779	+	887
novel.3528	NW_016537022.1	38277	42015	+	1300
novel.3529	NW_016537023.1	18780	22135	+	3300
novel.7311	NW_016562485.1	87	5896	-	1795
novel.3524	NW_016537001.1	5947	7930	-	1888
novel.4138	NW_016539104.1	3033	9625	+	1949
novel.4139	NW_016539104.1	245373	247575	+	1743
novel.2839	NW_016534578.1	9718	12046	-	2119
novel.2838	NW_016534577.1	43926	44237	+	258
novel.6005	NW_016548894.1	201122	202124	+	1003
novel.2835	NW_016534543.1	262159	262519	-	305
novel.2834	NW_016534543.1	33901	34399	+	499
novel.2837	NW_016534567.1	20117	42388	+	203
novel.4131	NW_016539069.1	59879	60297	+	363
novel.4136	NW_016539076.1	8753	9881	-	955
novel.2830	NW_016534510.1	129143	132745	+	463
novel.2833	NW_016534535.1	296	5583	+	446
novel.2832	NW_016534532.1	249298	250721	+	1076
novel.3527	NW_016537015.1	800982	801292	-	271
novel.3520	NW_016536978.1	7325	7628	-	252
novel.7310	NW_016562485.1	3993	5588	+	1562
novel.3521	NW_016536980.1	103037	103628	-	274
novel.7472	NW_016565639.1	230122	233601	-	2677
novel.5846	NW_016547658.1	44501	46937	-	1313
novel.3308	NW_016536170.1	66243	67036	+	760
novel.3309	NW_016536170.1	69048	69846	+	440
novel.7473	NW_016565651.1	16	3105	-	2928
novel.3304	NW_016536156.1	236209	239180	+	333
novel.3305	NW_016536157.1	18268	18621	-	354
novel.3306	NW_016536157.1	93054	94884	-	556
novel.3307	NW_016536170.1	56474	66191	+	374
novel.3300	NW_016536146.1	33449	52711	+	355

novel.3301	NW_016536146.1	168914	169628	+	236
novel.3302	NW_016536146.1	169705	170073	+	231
novel.3303	NW_016536155.1	4592	5197	+	565
novel.7471	NW_016565639.1	230122	232849	+	2075
novel.7476	NW_016565665.1	26094	26662	+	536
novel.7477	NW_016565681.1	407	1157	-	751
novel.7474	NW_016565653.1	6608	7050	+	402
novel.7475	NW_016565663.1	15562	36836	-	1423
novel.8671	NW_016604761.1	1	3277	+	2417
novel.8670	NW_016604703.1	1267	3418	-	1083
novel.5463	NW_016545255.1	4190	5913	-	1678
novel.2302	NW_016532990.1	1590	2005	-	371
novel.2301	NW_016532990.1	1689	2005	+	317
novel.2300	NW_016532973.1	16407	16747	-	294
novel.5467	NW_016545268.1	11468	11727	+	260
novel.5466	NW_016545267.1	8890	10194	-	1075
novel.5465	NW_016545261.1	7615	7989	+	263
novel.5464	NW_016545258.1	141627	155673	+	580
novel.5469	NW_016545291.1	7701	20218	+	436
novel.5468	NW_016545276.1	2648	14450	+	647
novel.4106	NW_016538926.1	13887	15976	+	2037
novel.3807	NW_016537812.1	192262	193325	-	314
novel.3806	NW_016537812.1	255416	257356	+	1896
novel.3805	NW_016537812.1	250587	255046	+	4460
novel.3804	NW_016537812.1	244264	245597	+	414
novel.3803	NW_016537812.1	91993	96386	+	1289
novel.3802	NW_016537808.1	113702	114455	+	754
novel.3801	NW_016537806.1	268209	268634	-	426
novel.3800	NW_016537806.1	249394	254997	-	468
novel.1760	NW_016531377.1	13267	14331	+	1065
novel.1761	NW_016531377.1	70361	82533	+	770
novel.1762	NW_016531382.1	489	9881	-	558
novel.1763	NW_016531390.1	3950	4825	+	204
novel.1764	NW_016531390.1	12788	16082	+	539
novel.1765	NW_016531392.1	30874	69390	+	974
novel.1766	NW_016531392.1	25342	30711	-	376
novel.1767	NW_016531393.1	55525	59016	+	502
novel.1768	NW_016531393.1	247865	249595	-	541
novel.1769	NW_016531398.1	63453	66604	+	280
novel.48	NW_016527355.1	22858	23155	-	298
novel.49	NW_016527358.1	316205	316659	-	418
novel.5072	NW_016542914.1	9987	11449	+	1277
novel.5073	NW_016542914.1	7925	24872	-	505
novel.5074	NW_016542914.1	10033	11449	-	822

novel.5075	NW_016542915.1	3464	3821	-	317
novel.5076	NW_016542921.1	69970	71315	+	1331
novel.5077	NW_016542921.1	4009	5914	-	1906
novel.5078	NW_016542921.1	71579	72039	-	252
novel.41	NW_016527350.1	200487	245893	+	392
novel.42	NW_016527351.1	1	2421	+	2238
novel.43	NW_016527351.1	349698	363537	+	8190
novel.44	NW_016527351.1	5381	7993	-	943
novel.45	NW_016527351.1	16580	19938	-	2360
novel.46	NW_016527351.1	387356	388691	-	667
novel.47	NW_016527353.1	193436	193709	-	227
novel.4159	NW_016539199.1	87428	89318	-	1891
novel.298	NW_016527945.1	337698	337962	+	208
novel.299	NW_016527946.1	380166	381199	+	225
novel.290	NW_016527922.1	57947	61271	-	398
novel.291	NW_016527925.1	2572	88953	-	484
novel.292	NW_016527931.1	129851	136119	+	1103
novel.293	NW_016527931.1	131073	138669	+	2446
novel.294	NW_016527931.1	136302	180006	-	920
novel.295	NW_016527934.1	750300	766407	+	537
novel.296	NW_016527936.1	776	1969	-	425
novel.5355	NW_016544579.1	12161	16207	-	546
novel.6479	NW_016552482.1	40436	46685	+	6250
novel.6478	NW_016552478.1	91521	91928	-	225
novel.6471	NW_016552420.1	100429	106091	+	3680
novel.6470	NW_016552420.1	61878	62479	+	217
novel.6473	NW_016552435.1	4590	5034	+	388
novel.6472	NW_016552433.1	45684	48120	+	287
novel.6475	NW_016552466.1	39458	40877	-	239
novel.6474	NW_016552456.1	42965	43425	-	300
novel.6477	NW_016552478.1	210125	211895	+	267
novel.6476	NW_016552467.1	159	993	+	495
novel.7047	NW_016558165.1	155587	192995	-	231
novel.7046	NW_016558165.1	122031	124086	+	2056
novel.8066	NW_016581384.1	1146	1379	-	212
novel.7044	NW_016558139.1	21154	21559	+	374
novel.8060	NW_016581030.1	2325	4779	+	247
novel.5357	NW_016544603.1	18748	19914	+	1114
novel.2314	NW_016533022.1	87501	92148	-	901
novel.8061	NW_016581038.1	1068	1358	+	250
novel.4301	NW_016539823.1	113421	113751	-	331
novel.4300	NW_016539823.1	50623	51127	-	449
novel.4303	NW_016539832.1	87480	89343	+	1751
novel.4302	NW_016539826.1	3666	4023	-	358

novel.4305	NW_016539833.1	7896	8287	+	337
novel.8062	NW_016581057.1	1	1216	+	898
novel.4307	NW_016539861.1	37048	37322	+	275
novel.4306	NW_016539833.1	11662	11936	-	227
novel.4309	NW_016539867.1	94345	94997	-	292
novel.4308	NW_016539867.1	1	791	+	612
novel.8063	NW_016581102.1	587	1157	+	571
novel.2315	NW_016533025.1	7914	8256	-	293
novel.3825	NW_016537873.1	1	9393	-	802
novel.3824	NW_016537867.1	49648	51618	-	1971
novel.3827	NW_016537880.1	27360	27865	+	449
novel.3826	NW_016537878.1	438181	441167	-	641
novel.3821	NW_016537857.1	646	1632	+	934
novel.3820	NW_016537856.1	8691	18178	+	1469
novel.3823	NW_016537867.1	6479	9662	-	635
novel.3822	NW_016537865.1	75115	80379	+	822
novel.3829	NW_016537880.1	27981	28914	-	934
novel.3828	NW_016537880.1	29080	29406	+	279
novel.2219	NW_016532642.1	26665	27995	+	1115
novel.7445	NW_016565026.1	223759	225037	-	208
novel.2218	NW_016532641.1	535638	564641	+	467
novel.7444	NW_016564953.1	12509	13177	-	624
novel.6819	NW_016555795.1	16771	17499	+	628
novel.6818	NW_016555795.1	6673	9700	+	406
novel.6813	NW_016555690.1	108130	108804	-	469
novel.6812	NW_016555672.1	1600	2002	-	403
novel.6811	NW_016555656.1	31395	33120	-	1282
novel.6810	NW_016555656.1	28745	31292	-	2287
novel.6817	NW_016555779.1	4118	4367	-	203
novel.6816	NW_016555774.1	22637	24542	-	889
novel.6815	NW_016555704.1	47883	49322	-	1392
novel.6814	NW_016555704.1	31573	31959	+	387
novel.2158	NW_016532473.1	3913	4582	-	557
novel.2159	NW_016532477.1	130909	131220	-	268
novel.2156	NW_016532458.1	149184	149742	-	431
novel.2157	NW_016532465.1	2478	11157	+	1171
novel.2154	NW_016532455.1	43533	46976	-	325
novel.2155	NW_016532458.1	383899	384185	+	230
novel.2152	NW_016532418.1	21620	26276	-	4545
novel.2153	NW_016532428.1	159731	191041	-	1437
novel.2150	NW_016532418.1	22834	26276	+	2926
novel.2151	NW_016532418.1	19359	20897	-	1160
novel.8419	NW_016593963.1	2	1112	-	1111
novel.8418	NW_016593961.1	1	390	+	284

novel.8411	NW_016592979.1	6	1054	+	1049
novel.8410	NW_016592876.1	1	1035	-	663
novel.8413	NW_016593112.1	1	1061	-	1061
novel.8412	NW_016593095.1	1	1060	+	509
novel.8415	NW_016593293.1	23	1072	-	890
novel.8414	NW_016593127.1	1	900	+	900
novel.8417	NW_016593887.1	1	1107	+	304
novel.8416	NW_016593330.1	1	687	+	332
novel.6438	NW_016552119.1	317986	319367	-	262
novel.5892	NW_016548009.1	30003	30319	+	271
novel.5893	NW_016548032.1	70837	86311	+	9322
novel.5890	NW_016548004.1	51054	51614	+	298
novel.5891	NW_016548004.1	51294	54028	-	631
novel.5896	NW_016548062.1	147186	147741	-	556
novel.5897	NW_016548073.1	123939	125210	-	358
novel.5894	NW_016548032.1	32757	35504	-	527
novel.5895	NW_016548058.1	2884	3223	-	315
novel.5898	NW_016548076.1	54919	55539	+	426
novel.5899	NW_016548076.1	635	7089	-	573
novel.265	NW_016527862.1	33755	34154	+	379
novel.264	NW_016527861.1	397897	403326	-	1975
novel.267	NW_016527886.1	4406	5151	-	746
novel.266	NW_016527865.1	47683	48440	+	588
novel.261	NW_016527847.1	58675	73054	+	1243
novel.260	NW_016527847.1	30078	30493	+	268
novel.263	NW_016527861.1	396882	399855	+	2974
novel.262	NW_016527847.1	40817	45437	-	623
novel.8058	NW_016581005.1	4847	34357	-	1460
novel.6264	NW_016550576.1	19536	21838	-	2262
novel.6265	NW_016550586.1	48363	48731	+	313
novel.6266	NW_016550590.1	60213	60969	+	707
novel.6267	NW_016550590.1	57564	57908	-	251
novel.6260	NW_016550507.1	8504	9435	-	686
novel.6261	NW_016550518.1	68954	73084	-	250
novel.6262	NW_016550576.1	7888	8471	+	536
novel.6263	NW_016550576.1	8661	8924	+	217
novel.6268	NW_016550590.1	60487	61538	-	1012
novel.6269	NW_016550597.1	11033	11647	-	615
novel.5795	NW_016547219.1	10970	13298	+	2329
novel.5794	NW_016547202.1	292632	354817	-	1101
novel.5797	NW_016547270.1	58530	59404	+	829
novel.5796	NW_016547238.1	71769	75259	+	3491
novel.5791	NW_016547194.1	3795	5667	-	1873
novel.5790	NW_016547194.1	3796	5292	+	1497

novel.5793	NW_016547202.1	318549	319525	+	921
novel.5792	NW_016547200.1	105310	107354	-	1587
novel.193	NW_016527660.1	43254	47859	+	1472
novel.192	NW_016527658.1	13742	45028	-	230
novel.191	NW_016527655.1	5370	5677	-	254
novel.190	NW_016527651.1	33299	33615	+	291
novel.5799	NW_016547349.1	26227	28550	-	783
novel.5798	NW_016547347.1	27880	36207	+	295
novel.195	NW_016527662.1	331944	333290	+	1296
novel.194	NW_016527662.1	282212	285002	+	231
novel.8172	NW_016584959.1	530	6061	+	368
novel.8173	NW_016584972.1	71	783	-	656
novel.8170	NW_016584752.1	4477	4799	+	301
novel.8171	NW_016584803.1	707	1390	-	239
novel.8176	NW_016585195.1	1501	2675	-	1118
novel.8177	NW_016585203.1	11438	11946	+	452
novel.8174	NW_016584977.1	25	283	-	225
novel.8175	NW_016585113.1	22	535	+	461
novel.7335	NW_016562884.1	17525	18716	-	917
novel.7334	NW_016562882.1	1	1165	-	742
novel.8178	NW_016585276.1	1780	2395	-	526
novel.8179	NW_016585345.1	1942	2300	+	203
novel.7331	NW_016562863.1	4465	4896	+	385
novel.7330	NW_016562835.1	4562	8009	+	649
novel.7333	NW_016562863.1	6005	6610	-	552
novel.7332	NW_016562863.1	2155	5141	-	513
novel.1409	NW_016530395.1	42652	42928	-	221
novel.1408	NW_016530390.1	178220	181736	+	803
novel.1959	NW_016531904.1	26563	29508	+	834
novel.1958	NW_016531902.1	16763	20435	-	3618
novel.1957	NW_016531902.1	16076	18506	+	2392
novel.1956	NW_016531900.1	104270	105822	-	1553
novel.1955	NW_016531900.1	89577	99307	-	3169
novel.1954	NW_016531900.1	45591	48017	-	852
novel.1953	NW_016531900.1	101698	105822	+	4069
novel.1952	NW_016531900.1	89577	96244	+	6621
novel.1951	NW_016531897.1	70219	72234	+	276
novel.1950	NW_016531889.1	10281	16838	-	807
novel.5249	NW_016543870.1	48078	48695	+	618
novel.5248	NW_016543869.1	16729	19898	-	1715
novel.5247	NW_016543868.1	13055	46800	-	1467
novel.5246	NW_016543858.1	13676	15780	-	459
novel.5245	NW_016543858.1	18318	18520	+	203
novel.5244	NW_016543846.1	910	2365	+	370

novel.5243	NW_016543844.1	61479	62575	+	665
novel.5242	NW_016543841.1	1200	1708	+	509
novel.5241	NW_016543841.1	1	434	+	434
novel.5240	NW_016543840.1	195	10113	+	259
novel.379	NW_016528087.1	71308	71990	+	628
novel.378	NW_016528086.1	1297	1612	+	280
novel.377	NW_016528082.1	37962	44546	-	365
novel.376	NW_016528080.1	132023	136682	-	493
novel.375	NW_016528080.1	39603	39904	-	256
novel.374	NW_016528080.1	3379	4128	-	750
novel.373	NW_016528080.1	38807	39134	+	302
novel.372	NW_016528080.1	37084	37803	+	669
novel.371	NW_016528080.1	35725	36914	+	1190
novel.370	NW_016528070.1	685043	730495	-	765
novel.8316	NW_016589777.1	488	5322	+	242
novel.8317	NW_016589833.1	10751	26404	+	922
novel.8314	NW_016589757.1	798	1215	+	263
novel.8315	NW_016589777.1	1	5107	+	304
novel.8312	NW_016589630.1	1	608	-	571
novel.8313	NW_016589638.1	2055	5557	-	201
novel.8310	NW_016589475.1	15511	15890	-	299
novel.8311	NW_016589576.1	25116	29972	-	390
novel.8318	NW_016589915.1	689	10103	+	208
novel.8319	NW_016589985.1	24	379	+	356
novel.3436	NW_016536636.1	314931	316197	+	226
novel.3437	NW_016536638.1	313795	329416	+	1324
novel.3142	NW_016535631.1	4580	9086	-	522
novel.3143	NW_016535631.1	5652	13896	-	812
novel.3140	NW_016535627.1	106669	107748	-	1060
novel.3141	NW_016535631.1	3780	12842	+	2318
novel.3146	NW_016535632.1	54722	57284	-	1335
novel.3147	NW_016535632.1	185373	186302	-	930
novel.3144	NW_016535631.1	12313	13124	-	707
novel.3145	NW_016535631.1	39916	40125	-	210
novel.3148	NW_016535632.1	186684	186940	-	209
novel.3149	NW_016535639.1	595	2064	+	268
novel.5438	NW_016545067.1	59869	70263	-	2941
novel.5439	NW_016545082.1	198742	199318	-	278
novel.2817	NW_016534450.1	53666	53991	-	292
novel.2816	NW_016534449.1	119280	119796	-	326
novel.2815	NW_016534449.1	112810	129812	+	2684
novel.2814	NW_016534447.1	1	728	-	452
novel.4118	NW_016538973.1	5129	7952	-	616
novel.4119	NW_016538978.1	9201	10489	+	529

novel.2811	NW_016534425.1	55994	56305	-	259
novel.2810	NW_016534422.1	424541	424875	+	300
novel.4114	NW_016538972.1	155149	169215	+	1817
novel.4115	NW_016538972.1	80728	84959	-	1518
novel.4116	NW_016538972.1	111875	113575	-	1611
novel.4117	NW_016538973.1	7249	7952	+	704
novel.4110	NW_016538933.1	11761	22908	+	412
novel.4111	NW_016538943.1	99838	102231	-	213
novel.4112	NW_016538946.1	1121	2602	-	304
novel.4113	NW_016538947.1	23230	37544	+	676
novel.3494	NW_016536839.1	96503	98390	-	406
novel.3367	NW_016536393.1	14342	15693	+	1248
novel.3496	NW_016536845.1	58972	59613	+	642
novel.3497	NW_016536846.1	52142	55953	-	1597
novel.3490	NW_016536811.1	109011	113290	-	637
novel.3491	NW_016536831.1	185821	186304	-	484
novel.3360	NW_016536355.1	69409	84270	+	1100
novel.3361	NW_016536371.1	4861	6184	+	385
novel.3498	NW_016536852.1	10227	10889	+	502
novel.2420	NW_016533319.1	457310	458172	-	249
novel.3368	NW_016536393.1	24895	25543	+	604
novel.3369	NW_016536393.1	49252	60927	+	379
novel.2353	NW_016533134.1	8802	15013	-	496
novel.2354	NW_016533138.1	63384	64328	-	945
novel.7338	NW_016563017.1	109	2597	+	985
novel.2355	NW_016533141.1	198668	199630	+	963
novel.2725	NW_016534168.1	370475	406377	-	975
novel.2356	NW_016533147.1	23242	28156	-	1651
novel.2724	NW_016534168.1	21493	25187	-	2947
novel.2425	NW_016533337.1	234595	235152	+	524
novel.2727	NW_016534181.1	2277	2913	+	583
novel.7200	NW_016560469.1	18298	18611	-	258
novel.2726	NW_016534171.1	29595	29984	-	258
novel.8391	NW_016591868.1	898	1288	+	339
novel.2721	NW_016534165.1	162268	181963	-	7210
novel.2720	NW_016534165.1	162268	167936	+	5669
novel.2891	NW_016534759.1	112532	120202	+	1537
novel.2890	NW_016534754.1	9650	12244	-	251
novel.7203	NW_016560507.1	8832	9118	-	287
novel.3843	NW_016537949.1	17456	19269	-	1758
novel.7834	NW_016573450.1	1	1834	-	1778
novel.3842	NW_016537949.1	1	520	-	520
novel.8644	NW_016603991.1	2212	2840	+	214
novel.8645	NW_016603991.1	1	1375	-	514

novel.8646	NW_016604017.1	1	3045	+	3045
novel.5405	NW_016544889.1	222203	222615	+	413
novel.2364	NW_016533157.1	223105	309332	+	289
novel.5407	NW_016544889.1	217349	218720	-	1293
novel.7552	NW_016567080.1	52	2677	+	1759
novel.5401	NW_016544831.1	266353	345672	+	2117
novel.5400	NW_016544828.1	168889	178911	-	7909
novel.5403	NW_016544889.1	16302	19812	+	409
novel.5402	NW_016544833.1	197907	198669	+	763
novel.971	NW_016529464.1	195182	252658	-	286
novel.7555	NW_016567150.1	73905	74173	-	200
novel.973	NW_016529475.1	414735	415391	+	590
novel.972	NW_016529470.1	154601	159971	-	243
novel.5409	NW_016544891.1	30852	36224	-	233
novel.5408	NW_016544889.1	236060	239922	-	495
novel.977	NW_016529486.1	120923	121695	-	495
novel.7554	NW_016567144.1	7531	9277	+	652
novel.8642	NW_016603982.1	255	3021	-	2767
novel.8643	NW_016603988.1	1	3025	+	2057
novel.5825	NW_016547550.1	11665	15449	+	3785
novel.3847	NW_016537970.1	2063	2381	+	319
novel.7788	NW_016572304.1	47996	48365	-	323
novel.7336	NW_016562895.1	2382	7491	-	522
novel.979	NW_016529498.1	43434	63183	-	1654
novel.5824	NW_016547549.1	114466	119682	-	2014
novel.3846	NW_016537966.1	27057	27331	-	229
novel.7789	NW_016572313.1	1669	2286	+	561
novel.978	NW_016529486.1	171095	174650	-	2105
novel.68	NW_016527382.1	39131	53874	-	399
novel.69	NW_016527385.1	17947	18638	+	692
novel.3845	NW_016537966.1	6054	6366	+	313
novel.67	NW_016527381.1	651750	663341	+	384
novel.64	NW_016527381.1	451811	458669	+	287
novel.65	NW_016527381.1	483979	501568	+	2553
novel.62	NW_016527380.1	8563	39344	-	690
novel.63	NW_016527381.1	266511	269366	+	215
novel.60	NW_016527375.1	137751	139417	-	764
novel.61	NW_016527380.1	359905	361259	+	385
novel.8248	NW_016587126.1	241907	248288	+	441
novel.4758	NW_016541525.1	105555	106313	+	705
novel.4759	NW_016541526.1	68939	78574	-	234
novel.8249	NW_016587126.1	83920	85473	-	1188
novel.4752	NW_016541522.1	4195	4718	+	524
novel.4753	NW_016541522.1	4864	5116	+	223

novel.4750	NW_016541514.1	6097	8367	+	2220
novel.4751	NW_016541519.1	300604	301160	+	508
novel.4756	NW_016541524.1	114908	122626	-	5298
novel.4757	NW_016541525.1	89934	94787	+	505
novel.4754	NW_016541522.1	24837	27188	+	2314
novel.4755	NW_016541522.1	24765	27188	-	1823
novel.3844	NW_016537952.1	18709	21450	-	258
novel.4692	NW_016541316.1	1044	1485	-	412
novel.2338	NW_016533086.1	3035	5519	-	578
novel.4041	NW_016538650.1	11807	12420	-	382
novel.6453	NW_016552251.1	287369	287684	-	316
novel.6452	NW_016552251.1	8912	9252	-	290
novel.6451	NW_016552251.1	287758	292201	+	4250
novel.6450	NW_016552244.1	140947	187764	+	452
novel.6457	NW_016552296.1	44679	56501	-	589
novel.8246	NW_016587126.1	123442	126354	+	316
novel.6455	NW_016552274.1	282	5308	+	274
novel.6454	NW_016552253.1	738	2044	-	392
novel.6459	NW_016552311.1	1243	1539	-	257
novel.8247	NW_016587126.1	202655	203032	+	304
novel.2096	NW_016532259.1	365170	365729	-	414
novel.4329	NW_016539946.1	46456	75891	+	822
novel.4328	NW_016539941.1	141328	142413	-	460
novel.4323	NW_016539932.1	64867	65586	-	302
novel.4322	NW_016539932.1	58804	61466	-	505
novel.4321	NW_016539926.1	22694	28576	+	2196
novel.4320	NW_016539924.1	49101	49921	-	821
novel.4327	NW_016539941.1	391327	403765	+	251
novel.4326	NW_016539940.1	41418	42089	-	272
novel.4325	NW_016539932.1	69978	72239	-	268
novel.4324	NW_016539932.1	67406	68294	-	245
novel.2090	NW_016532228.1	60468	66484	-	5815
novel.7787	NW_016572304.1	24311	25112	-	599
novel.418	NW_016528156.1	25384	28272	+	2725
novel.419	NW_016528156.1	279865	282638	-	965
novel.412	NW_016528148.1	8195	49490	-	243
novel.413	NW_016528150.1	58454	59120	+	616
novel.410	NW_016528147.1	102872	104473	+	459
novel.411	NW_016528147.1	153428	153838	+	382
novel.416	NW_016528154.1	232683	235530	+	2429
novel.417	NW_016528154.1	183638	231678	-	1121
novel.414	NW_016528151.1	1673	2014	-	342
novel.415	NW_016528153.1	150860	151859	-	1000
novel.4099	NW_016538908.1	128780	131813	-	1697

novel.8789	NW_016607063.1	2289	2730	+	442
novel.8788	NW_016607026.1	3378	7240	-	3863
novel.7478	NW_016565681.1	1306	1612	-	263
novel.7479	NW_016565721.1	14	423	+	217
novel.8781	NW_016606915.1	2203	7362	+	2992
novel.8780	NW_016606905.1	2784	7336	+	954
novel.8783	NW_016606935.1	30	7402	-	1948
novel.8782	NW_016606920.1	1333	3052	-	522
novel.8785	NW_016606990.1	1222	7195	+	2626
novel.8784	NW_016606985.1	1797	3477	+	1139
novel.8787	NW_016606993.1	6780	7818	-	856
novel.8786	NW_016606993.1	6224	7818	-	1595
novel.6879	NW_016556494.1	24218	24651	-	434
novel.6878	NW_016556494.1	23846	24788	+	891
novel.3809	NW_016537824.1	261679	262426	-	697
novel.3808	NW_016537812.1	250605	251724	-	1057
novel.6875	NW_016556489.1	39265	50090	+	1384
novel.6874	NW_016556449.1	487	2858	+	268
novel.6877	NW_016556489.1	17397	46413	-	363
novel.6876	NW_016556489.1	13056	50529	-	209
novel.6871	NW_016556374.1	1808	23775	-	602
novel.6870	NW_016556337.1	30465	37335	+	215
novel.6873	NW_016556434.1	8137	13484	+	381
novel.6872	NW_016556385.1	52325	60558	+	654
novel.2170	NW_016532503.1	23844	24113	-	235
novel.2171	NW_016532504.1	56866	57163	+	245
novel.2172	NW_016532509.1	768	1186	+	419
novel.2173	NW_016532509.1	26714	27140	-	427
novel.2174	NW_016532509.1	53181	55904	-	2724
novel.2175	NW_016532522.1	2524	2839	-	260
novel.2176	NW_016532522.1	56643	57899	-	1230
novel.2177	NW_016532536.1	247	929	+	657
novel.2178	NW_016532538.1	196169	198264	+	409
novel.2179	NW_016532541.1	50223	50493	+	219
novel.975	NW_016529483.1	31201	46045	-	288
novel.8433	NW_016594612.1	1	1155	-	1052
novel.8432	NW_016594575.1	1	1152	-	1152
novel.8431	NW_016594575.1	1	1152	+	1152
novel.8430	NW_016594568.1	356	1151	+	709
novel.8437	NW_016595033.1	1	1185	+	1185
novel.8436	NW_016595010.1	1	957	+	957
novel.8435	NW_016594993.1	779	1182	+	404
novel.8434	NW_016594826.1	1	1092	+	720
novel.8439	NW_016595289.1	1	733	-	313

novel.8438	NW_016595100.1	244	1094	-	851
novel.974	NW_016529479.1	14467	17670	-	3204
novel.2337	NW_016533086.1	913	1838	-	741
novel.6458	NW_016552311.1	1	1034	-	1034
novel.3779	NW_016537760.1	229562	230143	+	582
novel.3778	NW_016537759.1	491994	492433	+	216
novel.3771	NW_016537750.1	468869	469557	-	415
novel.3770	NW_016537750.1	532913	535618	+	2612
novel.3773	NW_016537751.1	76608	82561	+	447
novel.3772	NW_016537750.1	532913	539928	-	2773
novel.3775	NW_016537758.1	97545	97884	+	340
novel.3774	NW_016537751.1	64571	64873	-	303
novel.3777	NW_016537758.1	169723	170004	+	282
novel.3776	NW_016537758.1	167817	169302	+	1431
novel.4921	NW_016542277.1	64980	70531	-	2113
novel.4920	NW_016542276.1	29322	32201	-	2637
novel.4923	NW_016542288.1	99730	100483	-	248
novel.4922	NW_016542277.1	70610	71366	-	734
novel.4925	NW_016542296.1	30047	31664	-	225
novel.4924	NW_016542296.1	19423	25903	-	798
novel.4927	NW_016542296.1	201179	202089	-	911
novel.4926	NW_016542296.1	186984	200963	-	758
novel.4929	NW_016542305.1	40114	49570	+	465
novel.4928	NW_016542304.1	1	278	-	278
novel.4699	NW_016541329.1	66339	67438	+	932
novel.4698	NW_016541329.1	22322	22754	+	278
novel.4693	NW_016541316.1	2935	3776	-	748
novel.6147	NW_016549859.1	5420	5820	+	236
novel.4691	NW_016541316.1	2075	3780	+	1601
novel.4690	NW_016541315.1	6716	9439	-	2599
novel.4697	NW_016541327.1	3039	3422	+	337
novel.4696	NW_016541321.1	139091	141348	-	379
novel.4695	NW_016541321.1	108783	111540	-	554
novel.4694	NW_016541321.1	153112	174656	+	1690
novel.6208	NW_016550219.1	29643	30672	-	275
novel.6209	NW_016550225.1	60231	61259	-	281
novel.6206	NW_016550211.1	4652	10070	-	629
novel.6207	NW_016550219.1	29181	29425	-	245
novel.6204	NW_016550181.1	1	1282	+	1114
novel.6205	NW_016550181.1	1	1285	-	1242
novel.6202	NW_016550179.1	15297	15588	+	292
novel.6203	NW_016550179.1	15164	15588	-	254
novel.6200	NW_016550171.1	1698	2467	+	457
novel.6201	NW_016550177.1	114385	116864	-	367

novel.171	NW_016527599.1	292437	316674	-	764
novel.7918	NW_016575949.1	23117	26989	-	1684
novel.173	NW_016527611.1	230515	230802	+	254
novel.172	NW_016527604.1	557261	559531	+	247
novel.175	NW_016527611.1	398338	419365	+	413
novel.174	NW_016527611.1	356961	357329	+	331
novel.177	NW_016527614.1	86003	86751	+	617
novel.176	NW_016527611.1	577617	577898	+	215
novel.179	NW_016527618.1	52389	54449	+	1105
novel.178	NW_016527614.1	38467	40979	-	2266
novel.7913	NW_016575877.1	22	1040	+	998
novel.7912	NW_016575869.1	3276	4351	-	311
novel.7915	NW_016575932.1	4770	5786	+	1017
novel.7914	NW_016575880.1	1179	5772	+	395
novel.7917	NW_016575932.1	6011	6559	-	525
novel.7916	NW_016575932.1	4770	5786	-	1017
novel.8154	NW_016584276.1	732	1206	+	452
novel.8155	NW_016584276.1	627	1202	-	548
novel.8156	NW_016584320.1	4090	4500	-	390
novel.8157	NW_016584323.1	42837	50085	+	1289
novel.8150	NW_016584263.1	1	5488	-	392
novel.8151	NW_016584270.1	9104	13576	+	252
novel.8152	NW_016584270.1	8821	15553	-	1470
novel.8153	NW_016584271.1	346	940	-	228
novel.7649	NW_016568958.1	12143	13413	-	241
novel.7648	NW_016568958.1	5301	9630	-	4310
novel.8180	NW_016585345.1	1942	2301	-	316
novel.8158	NW_016584323.1	45390	45927	+	315
novel.8159	NW_016584339.1	2669	3021	+	327
novel.7319	NW_016562666.1	26015	27677	-	1663
novel.7318	NW_016562666.1	18846	20014	+	1169
novel.1423	NW_016530443.1	11481	20248	+	1453
novel.1422	NW_016530442.1	43875	53335	+	520
novel.1421	NW_016530442.1	42313	74090	+	1390
novel.1420	NW_016530430.1	315545	316903	+	1359
novel.1427	NW_016530448.1	88775	89449	-	675
novel.1426	NW_016530446.1	4220	4762	-	487
novel.1425	NW_016530446.1	2238	4518	+	605
novel.6618	NW_016553847.1	4758	5033	+	276
novel.6178	NW_016550053.1	248	754	+	450
novel.1429	NW_016530454.1	39547	42081	+	2485
novel.1428	NW_016530454.1	2807	3301	+	495
novel.6179	NW_016550053.1	124495	124854	+	303
novel.315	NW_016527960.1	922	1460	+	429

novel.3414	NW_016536532.1	119032	121806	+	1309
novel.317	NW_016527964.1	15754	43189	-	625
novel.316	NW_016527963.1	11706	12808	+	317
novel.311	NW_016527956.1	136867	138463	-	1597
novel.310	NW_016527956.1	32	1183	-	1152
novel.313	NW_016527958.1	22793	28478	+	226
novel.3415	NW_016536532.1	52007	52989	-	962
novel.319	NW_016527966.1	358683	392110	+	1142
novel.3416	NW_016536532.1	53044	54544	-	398
novel.6177	NW_016550033.1	5985	6489	+	484
novel.6170	NW_016550003.1	215538	215968	+	236
novel.6171	NW_016550003.1	22049	51981	-	589
novel.6172	NW_016550003.1	74057	74693	-	540
novel.6173	NW_016550004.1	73011	77338	+	609
novel.6996	NW_016557712.1	53531	69997	+	762
novel.3165	NW_016535683.1	116498	117809	+	719
novel.6994	NW_016557689.1	19771	20320	-	495
novel.3167	NW_016535685.1	6106	23275	-	305
novel.3160	NW_016535659.1	129465	136877	+	208
novel.3161	NW_016535664.1	1	527	-	474
novel.3162	NW_016535669.1	40758	43537	+	703
novel.3163	NW_016535669.1	35096	49274	-	217
novel.3168	NW_016535685.1	30016	30260	-	208
novel.3169	NW_016535687.1	37757	38466	+	710
novel.6998	NW_016557712.1	90347	98011	+	5379
novel.6999	NW_016557712.1	88849	94987	-	1667
novel.4176	NW_016539271.1	126915	128496	+	1582
novel.4177	NW_016539271.1	89519	90159	-	586
novel.4174	NW_016539250.1	52895	53339	+	268
novel.4175	NW_016539264.1	160114	162969	-	232
novel.4172	NW_016539237.1	15248	29847	-	369
novel.4173	NW_016539247.1	15592	17264	+	223
novel.4170	NW_016539231.1	54094	54949	-	258
novel.4171	NW_016539234.1	64225	69476	+	270
novel.8330	NW_016590180.1	6069	10162	-	291
novel.8331	NW_016590242.1	4036	4804	-	628
novel.8332	NW_016590289.1	755	1053	+	258
novel.8333	NW_016590439.1	345	682	+	308
novel.8334	NW_016590452.1	608	997	-	343
novel.8335	NW_016590532.1	67	328	+	239
novel.4178	NW_016539271.1	92586	98471	-	429
novel.8845	NW_016607852.1	10585	19839	-	553
novel.3348	NW_016536309.1	43690	47328	+	308
novel.3349	NW_016536309.1	405375	407227	+	815

novel.6198	NW_016550168.1	65145	71241	+	232
novel.6199	NW_016550169.1	60	3706	+	1504
novel.3340	NW_016536275.1	9477	16778	-	891
novel.3341	NW_016536275.1	10833	11125	-	238
novel.3342	NW_016536282.1	204247	212292	+	1629
novel.3343	NW_016536288.1	371	1215	-	566
novel.3344	NW_016536300.1	102580	112859	+	304
novel.3345	NW_016536306.1	38822	40578	+	549
novel.3346	NW_016536306.1	59598	60061	+	291
novel.3347	NW_016536306.1	39498	39919	-	422
novel.6887	NW_016556545.1	29961	30740	-	741
novel.4251	NW_016539566.1	17659	18028	-	241
novel.4250	NW_016539566.1	7482	7754	-	220
novel.6610	NW_016553722.1	22689	23817	-	1092
novel.6905	NW_016556763.1	28433	30773	-	652
novel.6904	NW_016556763.1	2572	25493	-	3482
novel.6907	NW_016556797.1	650	1590	+	449
novel.2695	NW_016534110.1	259589	283090	+	748
novel.2694	NW_016534108.1	115902	117599	-	1698
novel.2697	NW_016534114.1	38401	38939	+	501
novel.6906	NW_016556783.1	149540	150643	+	1104
novel.2691	NW_016534099.1	541040	541531	-	443
novel.2690	NW_016534099.1	193508	207038	-	340
novel.2693	NW_016534107.1	52305	63741	-	583
novel.2692	NW_016534100.1	9954	11969	-	1048
novel.6901	NW_016556735.1	74716	142012	+	360
novel.2699	NW_016534124.1	188050	190009	+	795
novel.2698	NW_016534118.1	23376	23586	+	211
novel.6900	NW_016556734.1	241694	242138	-	445
novel.6903	NW_016556753.1	1	201	-	201
novel.6902	NW_016556737.1	1	1078	-	1036
novel.5900	NW_016548076.1	55201	56524	-	1301
novel.5559	NW_016545745.1	21598	103249	+	1920
novel.2621	NW_016533880.1	26760	27109	-	300
novel.5558	NW_016545744.1	16524	17526	-	972
novel.5902	NW_016548103.1	15	3285	-	439
novel.2439	NW_016533373.1	3467	4270	+	624
novel.5557	NW_016545734.1	117108	117456	+	349
novel.5903	NW_016548109.1	39312	44066	-	2341
novel.5429	NW_016545026.1	4742	5544	-	389
novel.2348	NW_016533126.1	63637	64005	+	369
novel.5427	NW_016545025.1	114316	116343	+	1065
novel.5426	NW_016545023.1	59856	60424	-	486
novel.5425	NW_016545022.1	156393	158397	+	906

novel.2432	NW_016533358.1	5443	19215	+	444
novel.5423	NW_016545017.1	182527	183520	+	353
novel.2434	NW_016533366.1	163580	164179	-	334
novel.2437	NW_016533369.1	1	546	-	422
novel.5905	NW_016548154.1	10187	10457	-	225
novel.7919	NW_016575983.1	21504	22871	+	1311
novel.5554	NW_016545683.1	256152	257068	-	218
novel.5906	NW_016548177.1	118401	119395	+	228
novel.959	NW_016529432.1	482880	486910	-	3888
novel.170	NW_016527599.1	84722	85411	-	690
novel.957	NW_016529432.1	490646	502131	+	1528
novel.956	NW_016529430.1	858190	859428	-	1239
novel.955	NW_016529430.1	681869	682893	-	1025
novel.5907	NW_016548189.1	273254	296178	+	6569
novel.953	NW_016529430.1	624748	628090	-	3290
novel.952	NW_016529430.1	855300	856603	+	1304
novel.5497	NW_016545451.1	25297	25607	+	260
novel.950	NW_016529430.1	624708	630881	+	4266
novel.5908	NW_016548189.1	368877	372097	+	1455
novel.5551	NW_016545648.1	25394	26598	-	1205
novel.5909	NW_016548189.1	273896	282790	-	8644
novel.5550	NW_016545635.1	1962	2346	-	338
novel.6614	NW_016553806.1	44692	45164	+	473
novel.5494	NW_016545412.1	90230	91056	+	733
novel.7911	NW_016575774.1	7168	7683	-	354
novel.7910	NW_016575770.1	6008	7749	+	1711
novel.7353	NW_016563255.1	13185	18162	-	368
novel.5495	NW_016545412.1	238960	239333	-	374
novel.8507	NW_016598813.1	135	755	+	478
novel.8506	NW_016598704.1	1	373	+	373
novel.7317	NW_016562663.1	1202	6625	-	297
novel.7352	NW_016563237.1	33991	34270	+	280
novel.7316	NW_016562663.1	789	6625	+	702
novel.5492	NW_016545412.1	54657	55677	+	1021
novel.7315	NW_016562663.1	330	5938	+	482
novel.7314	NW_016562656.1	865	1834	-	922
novel.7313	NW_016562654.1	7340	7639	+	260
novel.8509	NW_016598928.1	718	1181	+	379
novel.7312	NW_016562511.1	7653	9348	+	224
novel.7351	NW_016563197.1	12874	18481	-	994
novel.8508	NW_016598902.1	1267	1586	-	320
novel.4778	NW_016541626.1	294589	296056	-	1216
novel.7647	NW_016568958.1	18104	21990	+	2164
novel.5493	NW_016545412.1	77636	81830	+	1111

novel.4774	NW_016541620.1	50028	143980	+	1229
novel.4775	NW_016541620.1	69684	69952	+	215
novel.4776	NW_016541623.1	1670	2638	+	916
novel.7646	NW_016568956.1	2223	2526	-	272
novel.4770	NW_016541599.1	418026	418658	-	431
novel.4771	NW_016541613.1	1302	1748	-	447
novel.4772	NW_016541616.1	139848	142356	+	1106
novel.4773	NW_016541618.1	60404	63109	+	604
novel.5490	NW_016545404.1	49531	50623	+	1016
novel.5801	NW_016547352.1	65281	68023	-	2052
novel.5491	NW_016545408.1	8836	9518	+	683
novel.6435	NW_016552100.1	3041	4249	-	1018
novel.6434	NW_016552100.1	3041	3653	+	613
novel.6437	NW_016552119.1	56813	57130	+	293
novel.6436	NW_016552109.1	4450	5093	+	330
novel.6431	NW_016552090.1	2892	3433	+	489
novel.6430	NW_016552079.1	1	3889	-	626
novel.6433	NW_016552091.1	105667	106109	+	443
novel.6432	NW_016552090.1	1382	4375	-	1307
novel.6439	NW_016552138.1	14400	14947	+	505
novel.4499	NW_016540577.1	29008	34227	-	402
novel.5800	NW_016547352.1	243750	244671	+	208
novel.788	NW_016529081.1	138102	138468	-	233
novel.789	NW_016529089.1	214963	221469	+	6065
novel.782	NW_016529048.1	72737	73580	-	503
novel.783	NW_016529053.1	146289	146773	+	322
novel.780	NW_016529046.1	470812	473395	+	2532
novel.1424	NW_016530445.1	9854	25165	-	632
novel.786	NW_016529067.1	541111	551058	+	2749
novel.787	NW_016529067.1	840609	842910	-	2096
novel.784	NW_016529066.1	3866	4142	-	220
novel.785	NW_016529067.1	1466	2509	+	734
novel.4349	NW_016540030.1	250137	251754	-	1561
novel.4348	NW_016540030.1	16593	57635	+	877
novel.4345	NW_016540010.1	78651	83850	-	285
novel.4344	NW_016540007.1	106722	114151	-	237
novel.4347	NW_016540022.1	3510	3808	-	255
novel.4346	NW_016540011.1	36980	37448	-	469
novel.4341	NW_016540005.1	792077	792512	+	436
novel.4340	NW_016540005.1	4888	110528	+	2589
novel.4343	NW_016540005.1	736834	775441	-	947
novel.4342	NW_016540005.1	219834	220884	-	205
novel.4355	NW_016540047.1	5890	6733	-	663
novel.5802	NW_016547359.1	107145	107375	-	231

novel.430	NW_016528185.1	39337	40290	+	920
novel.431	NW_016528185.1	90783	91378	+	554
novel.432	NW_016528190.1	186003	191470	+	320
novel.433	NW_016528201.1	171289	173979	-	368
novel.434	NW_016528202.1	16523	17916	+	442
novel.435	NW_016528205.1	59482	62236	+	636
novel.436	NW_016528205.1	62246	62879	-	384
novel.437	NW_016528207.1	23367	23903	+	334
novel.438	NW_016528208.1	410351	411761	-	1411
novel.439	NW_016528218.1	152347	166935	-	241
novel.3860	NW_016538048.1	10314	10763	-	430
novel.5805	NW_016547402.1	7358	9938	-	602
novel.1858	NW_016531613.1	6067	6306	+	240
novel.1859	NW_016531616.1	245830	246184	+	298
novel.1852	NW_016531596.1	2971	4498	+	716
novel.1853	NW_016531596.1	244837	245468	-	254
novel.1850	NW_016531594.1	466958	472972	+	488
novel.1851	NW_016531595.1	40579	41033	-	399
novel.3863	NW_016538055.1	2564	4570	+	1920
novel.1857	NW_016531610.1	66550	66836	+	235
novel.1854	NW_016531603.1	249978	251504	+	1527
novel.3924	NW_016538273.1	102905	127549	+	569
novel.5168	NW_016543454.1	5821	54245	+	730
novel.5169	NW_016543473.1	166328	182685	+	5053
novel.5804	NW_016547377.1	1	6958	+	919
novel.5162	NW_016543422.1	73284	74332	-	1049
novel.5163	NW_016543422.1	199495	199898	-	352
novel.5160	NW_016543389.1	61725	62367	-	252
novel.5161	NW_016543400.1	5479	17885	-	250
novel.5166	NW_016543443.1	156761	157137	+	339
novel.5167	NW_016543447.1	95549	96304	+	705
novel.5164	NW_016543423.1	36816	39019	+	262
novel.5165	NW_016543440.1	35	740	-	674
novel.2484	NW_016533517.1	1274	1559	+	253
novel.2485	NW_016533517.1	1	1154	-	591
novel.3862	NW_016538055.1	11	539	+	392
novel.3925	NW_016538273.1	4532	8505	-	785
novel.7102	NW_016559113.1	55977	279580	-	479
novel.7103	NW_016559116.1	18792	35032	-	1885
novel.7100	NW_016559099.1	55724	56727	-	971
novel.2487	NW_016533525.1	11319	41340	-	544
novel.7458	NW_016565342.1	5931	6369	-	382
novel.7107	NW_016559120.1	40577	45258	+	4597
novel.7104	NW_016559119.1	130744	132075	+	1287

novel.7105	NW_016559120.1	14931	15497	+	501
novel.7454	NW_016565319.1	10281	13188	+	559
novel.2480	NW_016533504.1	181432	181650	-	219
novel.7108	NW_016559120.1	152242	152733	+	373
novel.7109	NW_016559120.1	5003	6910	-	1812
novel.7450	NW_016565162.1	21068	23811	+	487
novel.7451	NW_016565222.1	1	5469	-	339
novel.7452	NW_016565234.1	2123	2625	+	463
novel.7453	NW_016565280.1	2709	3357	+	458
novel.2901	NW_016534792.1	133604	134382	-	723
novel.2482	NW_016533512.1	98	347	+	250
novel.314	NW_016527958.1	21729	22585	-	332
novel.3865	NW_016538055.1	11	539	-	481
novel.2951	NW_016534971.1	344531	346056	+	789
novel.6857	NW_016556200.1	1	1320	-	345
novel.6856	NW_016556195.1	9340	73808	-	467
novel.6855	NW_016556185.1	16948	17794	-	271
novel.6854	NW_016556180.1	26267	26711	-	445
novel.6853	NW_016556180.1	124106	124327	+	222
novel.6852	NW_016556160.1	1	1031	-	886
novel.6851	NW_016556160.1	1	844	+	844
novel.6850	NW_016556114.1	73769	77589	-	3766
novel.6859	NW_016556209.1	1846	2069	-	224
novel.6858	NW_016556207.1	29299	29977	-	274
novel.2900	NW_016534791.1	7786	8634	+	849
novel.3864	NW_016538055.1	9064	9833	+	730
novel.312	NW_016527956.1	211260	214369	-	260
novel.8455	NW_016596185.1	332	1201	+	366
novel.8454	NW_016596042.1	1	1264	-	856
novel.8457	NW_016596416.1	1	351	-	351
novel.8456	NW_016596291.1	1	1287	+	1287
novel.8451	NW_016595910.1	624	1252	-	629
novel.8450	NW_016595846.1	1	1238	+	595
novel.8453	NW_016596039.1	346	1263	-	345
novel.8452	NW_016595912.1	1	1168	-	1037
novel.8459	NW_016596448.1	1	1302	+	1040
novel.8458	NW_016596446.1	38	1186	+	509
novel.2903	NW_016534794.1	31395	32338	+	893
novel.318	NW_016527966.1	306546	307998	+	488
novel.2902	NW_016534794.1	7744	10234	+	2455
novel.898	NW_016529305.1	555331	560988	-	398
novel.899	NW_016529316.1	67067	71179	+	274
novel.896	NW_016529305.1	460943	461880	+	789
novel.897	NW_016529305.1	313605	333554	-	219

novel.894	NW_016529302.1	54107	54367	+	230
novel.895	NW_016529302.1	8352	13128	-	781
novel.892	NW_016529299.1	7217	8266	-	238
novel.893	NW_016529300.1	662557	684767	+	597
novel.890	NW_016529296.1	9452	9817	-	309
novel.891	NW_016529298.1	208119	208460	-	286
novel.2905	NW_016534801.1	169796	170170	+	375
novel.3753	NW_016537679.1	4231	4462	+	232
novel.3752	NW_016537674.1	27503	28581	-	1052
novel.3751	NW_016537674.1	27004	28581	+	634
novel.3750	NW_016537672.1	310547	327898	-	445
novel.3757	NW_016537687.1	173345	174979	+	1635
novel.3756	NW_016537687.1	3594	4889	+	1296
novel.3755	NW_016537683.1	12840	19147	-	720
novel.3754	NW_016537679.1	155171	155449	-	225
novel.3759	NW_016537689.1	48393	49416	+	718
novel.3758	NW_016537687.1	169314	172302	-	1617
novel.2904	NW_016534801.1	113081	114115	+	1008
novel.4909	NW_016542195.1	35206	38794	-	3460
novel.4908	NW_016542195.1	34502	38794	+	3631
novel.4903	NW_016542176.1	8035	8668	+	279
novel.4902	NW_016542169.1	176975	185677	+	2958
novel.4901	NW_016542164.1	5466	6470	+	241
novel.4900	NW_016542162.1	27575	55484	+	2989
novel.4907	NW_016542189.1	124605	124812	+	208
novel.4906	NW_016542183.1	9419	10644	-	396
novel.4905	NW_016542181.1	87474	87859	-	292
novel.4904	NW_016542178.1	54521	54729	+	209
novel.2907	NW_016534802.1	26342	34131	+	1267
novel.2906	NW_016534801.1	430411	434052	+	3580
novel.6220	NW_016550251.1	229610	233545	+	2242
novel.6221	NW_016550251.1	234577	234912	+	280
novel.6222	NW_016550251.1	265117	271381	+	678
novel.6223	NW_016550251.1	114905	117572	-	2668
novel.6224	NW_016550251.1	218060	219903	-	1144
novel.6225	NW_016550264.1	46223	47234	+	733
novel.6226	NW_016550276.1	3838	4631	+	794
novel.6227	NW_016550276.1	5599	43958	+	319
novel.6228	NW_016550276.1	42518	43159	+	585
novel.6229	NW_016550276.1	43098	43958	-	665
novel.1639	NW_016531017.1	660969	661271	+	303
novel.1193	NW_016529915.1	8867	9769	-	903
novel.1192	NW_016529915.1	67946	78290	+	3079
novel.1191	NW_016529915.1	16561	17572	+	957

novel.1190	NW_016529914.1	28726	39000	-	649
novel.1197	NW_016529922.1	124859	126205	+	701
novel.1196	NW_016529922.1	1443	2370	+	891
novel.1195	NW_016529918.1	7237	10500	-	366
novel.1194	NW_016529915.1	27819	28612	-	737
novel.1629	NW_016531013.1	47488	51799	+	833
novel.1628	NW_016531011.1	33392	34587	+	618
novel.1199	NW_016529932.1	113992	121783	-	507
novel.1198	NW_016529923.1	174254	176713	-	2460
novel.5751	NW_016546997.1	43977	45406	-	1219
novel.5750	NW_016546997.1	41990	42320	-	281
novel.5753	NW_016546999.1	193948	195502	+	1509
novel.5752	NW_016546999.1	161945	162827	+	384
novel.5755	NW_016547014.1	31849	33793	-	1945
novel.5754	NW_016547007.1	353473	651956	+	1353
novel.5757	NW_016547023.1	120839	122040	+	405
novel.5756	NW_016547023.1	20723	102806	+	539
novel.5759	NW_016547042.1	40883	41907	+	776
novel.5758	NW_016547023.1	60139	60968	-	784
novel.6155	NW_016549869.1	2273	2836	-	527
novel.7933	NW_016576165.1	9086	9679	-	461
novel.7932	NW_016576160.1	5750	7529	-	1724
novel.7931	NW_016576116.1	5034	7266	-	1160
novel.7930	NW_016576116.1	4648	4938	+	238
novel.7937	NW_016576361.1	1051	1562	+	463
novel.7936	NW_016576354.1	15882	16150	+	218
novel.7935	NW_016576316.1	4305	6294	-	1990
novel.7934	NW_016576195.1	452	1450	+	920
novel.7939	NW_016576381.1	9139	14672	+	479
novel.7938	NW_016576361.1	1668	2134	+	442
novel.159	NW_016527592.1	56747	57562	+	779
novel.158	NW_016527588.1	277172	277406	+	235
novel.1999	NW_016532025.1	134506	134976	-	421
novel.1998	NW_016532016.1	99647	101476	+	1773
novel.1993	NW_016531984.1	32825	33838	+	1014
novel.1992	NW_016531982.1	512544	564916	-	266
novel.1991	NW_016531982.1	77645	78790	+	657
novel.1990	NW_016531975.1	39627	41092	+	823
novel.1997	NW_016532014.1	156974	157510	-	430
novel.1996	NW_016532014.1	97658	109097	-	940
novel.1995	NW_016532014.1	156581	157062	+	435
novel.1994	NW_016531987.1	51256	366017	-	3722
novel.1445	NW_016530520.1	50567	53230	-	246
novel.1444	NW_016530520.1	55844	56504	+	432

novel.5289	NW_016544110.1	11133	11467	+	287
novel.5288	NW_016544099.1	350930	362178	+	538
novel.1441	NW_016530505.1	82389	83332	-	324
novel.1440	NW_016530503.1	88744	90088	-	1345
novel.1443	NW_016530516.1	381847	384332	+	2486
novel.1442	NW_016530507.1	152755	153458	-	523
novel.5283	NW_016544066.1	575107	580111	+	1958
novel.5282	NW_016544061.1	31466	58076	-	932
novel.5281	NW_016544061.1	116162	120444	+	580
novel.5280	NW_016544060.1	36689	46005	-	277
novel.5287	NW_016544097.1	11133	14700	-	1710
novel.5286	NW_016544092.1	1	427	-	400
novel.5285	NW_016544089.1	7205	18634	-	2185
novel.5284	NW_016544075.1	274	1274	-	1001
novel.339	NW_016528021.1	500732	518615	+	1074
novel.338	NW_016528019.1	79716	80365	-	542
novel.333	NW_016528003.1	174052	174638	+	587
novel.332	NW_016528003.1	168121	171730	+	866
novel.331	NW_016528002.1	14111	29347	-	302
novel.330	NW_016528000.1	40224	41664	-	240
novel.337	NW_016528008.1	235931	236537	+	550
novel.336	NW_016528006.1	19063	72047	+	321
novel.335	NW_016528005.1	28252	28834	+	583
novel.334	NW_016528004.1	61144	61791	+	222
novel.2000	NW_016532031.1	228982	229516	-	280
novel.2553	NW_016533721.1	404313	404664	+	352
novel.2550	NW_016533719.1	43473	43794	-	278
novel.2551	NW_016533720.1	9908	19206	+	1250
novel.2004	NW_016532035.1	447287	462203	+	393
novel.2557	NW_016533726.1	8171	10518	-	2348
novel.4486	NW_016540573.1	300756	304313	-	1104
novel.4487	NW_016540573.1	372498	373198	-	470
novel.7669	NW_016569292.1	5095	5608	+	407
novel.7668	NW_016569273.1	1	576	-	519
novel.4482	NW_016540573.1	383707	384563	+	823
novel.4483	NW_016540573.1	988056	993848	+	293
novel.8138	NW_016583708.1	91491	94080	+	2075
novel.8139	NW_016583754.1	817	3352	-	337
novel.8136	NW_016583648.1	7285	7491	+	207
novel.8137	NW_016583684.1	6507	6922	+	341
novel.8134	NW_016583597.1	754	2169	+	723
novel.8135	NW_016583613.1	75835	76906	-	930
novel.8132	NW_016583481.1	584	1364	-	781
novel.8133	NW_016583502.1	5082	7191	+	1111

novel.8130	NW_016583448.1	1	1354	-	208
novel.7664	NW_016569145.1	4717	12116	+	501
novel.6013	NW_016548949.1	444548	450895	-	3687
novel.3108	NW_016535558.1	383209	383994	+	744
novel.3109	NW_016535558.1	340799	346654	-	514
novel.3106	NW_016535557.1	6424	6924	+	450
novel.3107	NW_016535558.1	323006	323567	+	507
novel.3104	NW_016535547.1	727164	727643	+	451
novel.3105	NW_016535549.1	6411	7053	-	609
novel.3102	NW_016535547.1	141098	146412	+	720
novel.3103	NW_016535547.1	450061	454324	+	1572
novel.3100	NW_016535546.1	88324	88940	+	571
novel.3101	NW_016535546.1	5551	12056	-	760
novel.6010	NW_016548947.1	14472	15693	-	272
novel.6017	NW_016548961.1	190257	190788	+	427
novel.3536	NW_016537024.1	253305	253539	+	200
novel.4158	NW_016539199.1	9064	9507	-	402
novel.3164	NW_016535680.1	26590	28638	-	1085
novel.6015	NW_016548961.1	30932	31572	+	585
novel.6997	NW_016557712.1	55815	58013	+	361
novel.4150	NW_016539164.1	93794	95371	-	1524
novel.4151	NW_016539168.1	134952	136261	-	1279
novel.4152	NW_016539168.1	137246	140464	-	3219
novel.6014	NW_016548961.1	20992	22511	+	798
novel.4154	NW_016539172.1	1	2970	-	716
novel.3166	NW_016535683.1	112015	112788	-	774
novel.4156	NW_016539187.1	31777	33969	-	361
novel.4157	NW_016539190.1	283773	284624	+	692
novel.8680	NW_016604873.1	2151	3659	-	1509
novel.8681	NW_016604896.1	1	3645	-	3645
novel.8682	NW_016604912.1	1	2937	-	1692
novel.8683	NW_016604928.1	596	1746	-	456
novel.8684	NW_016604929.1	837	2702	-	1015
novel.8685	NW_016604954.1	3205	3754	+	550
novel.8686	NW_016604966.1	1839	3764	-	338
novel.8687	NW_016605033.1	1	2982	+	627
novel.8688	NW_016605042.1	450	1741	-	320
novel.7594	NW_016567850.1	2511	3185	+	545
novel.7597	NW_016567917.1	1203	2873	-	1022
novel.7596	NW_016567917.1	3345	3731	+	344
novel.7591	NW_016567799.1	5317	10562	-	1561
novel.7590	NW_016567784.1	22038	23537	-	976
novel.7593	NW_016567830.1	986	5058	+	672
novel.7592	NW_016567807.1	2059	2514	+	424

novel.3538	NW_016537049.1	266183	267509	+	1327
novel.6990	NW_016557673.1	22068	24844	+	2310
novel.6991	NW_016557673.1	22035	25776	-	2685
novel.7462	NW_016565453.1	40670	42393	-	1260
novel.5999	NW_016548838.1	7194	9851	-	519
novel.5998	NW_016548838.1	62027	62694	+	407
novel.5997	NW_016548838.1	9470	10012	+	382
novel.5996	NW_016548833.1	757	1184	+	372
novel.5995	NW_016548828.1	33995	54172	-	657
novel.5994	NW_016548828.1	45005	52046	+	6948
novel.5993	NW_016548816.1	229159	230003	-	815
novel.5992	NW_016548816.1	225378	230019	+	3899
novel.5991	NW_016548816.1	152287	154981	+	2695
novel.5990	NW_016548802.1	17847	18805	-	959
novel.7650	NW_016568958.1	13718	17702	-	3929
novel.1258	NW_016530082.1	122693	124152	+	1055
novel.1259	NW_016530082.1	198374	198576	-	203
novel.2413	NW_016533287.1	581157	593796	+	1266
novel.2412	NW_016533287.1	49185	49645	+	210
novel.2411	NW_016533276.1	104946	108247	+	3302
novel.2410	NW_016533274.1	71523	71882	-	360
novel.2417	NW_016533319.1	195634	196298	-	665
novel.2416	NW_016533319.1	644498	645011	+	301
novel.2415	NW_016533304.1	4535	5117	-	437
novel.2414	NW_016533304.1	34950	38382	+	713
novel.2419	NW_016533319.1	422871	425613	-	904
novel.2418	NW_016533319.1	196402	197853	-	904
novel.1256	NW_016530078.1	559005	559265	+	261
novel.935	NW_016529407.1	124848	125566	-	671
novel.8338	NW_016590558.1	70	384	-	261
novel.937	NW_016529408.1	129053	131081	-	975
novel.1709	NW_016531255.1	103395	104849	-	1309
novel.931	NW_016529400.1	228441	229046	-	429
novel.930	NW_016529400.1	1157913	1162646	+	407
novel.933	NW_016529403.1	292012	292449	+	215
novel.8339	NW_016590566.1	810	1061	-	200
novel.939	NW_016529410.1	399181	404945	-	245
novel.938	NW_016529409.1	407321	407591	+	271
novel.8849	NW_016607868.1	12721	17997	+	1374
novel.8842	NW_016607840.1	1406	8271	-	825
novel.8843	NW_016607844.1	6055	9699	-	3566
novel.8840	NW_016607828.1	5547	12637	-	479
novel.8841	NW_016607837.1	3377	4392	-	1016
novel.8846	NW_016607865.1	25609	27605	+	1484

novel.8847	NW_016607865.1	15820	17549	-	511
novel.8336	NW_016590537.1	267	739	-	433
novel.4179	NW_016539281.1	67773	68208	+	406
novel.28	NW_016527322.1	155363	156003	+	641
novel.29	NW_016527322.1	253477	257708	+	502
novel.22	NW_016527310.1	67212	68627	-	1310
novel.23	NW_016527315.1	147339	148817	+	528
novel.20	NW_016527301.1	704	18728	-	2937
novel.21	NW_016527310.1	116276	118516	+	2241
novel.26	NW_016527321.1	325749	327560	-	1812
novel.27	NW_016527321.1	331066	333552	-	829
novel.24	NW_016527315.1	513117	513796	-	251
novel.25	NW_016527321.1	322294	324614	-	2289
novel.4716	NW_016541387.1	27712	30427	+	602
novel.4717	NW_016541387.1	116702	117425	+	668
novel.4714	NW_016541347.1	59291	60730	+	1440
novel.4715	NW_016541354.1	363797	375945	-	903
novel.4712	NW_016541346.1	28570	30197	-	1229
novel.4713	NW_016541347.1	39780	40594	+	758
novel.4710	NW_016541344.1	1920	2241	+	274
novel.4711	NW_016541346.1	560486	560990	+	505
novel.4718	NW_016541387.1	122590	122829	-	218
novel.4719	NW_016541389.1	254714	255676	-	515
novel.6196	NW_016550166.1	17749	20415	-	622
novel.6197	NW_016550168.1	51461	64981	+	292
novel.6194	NW_016550129.1	52997	53355	-	304
novel.6195	NW_016550137.1	4874	5157	+	284
novel.6763	NW_016555200.1	137162	137722	+	561
novel.6192	NW_016550110.1	20570	21469	-	521
novel.6762	NW_016555198.1	10916	12724	-	364
novel.6193	NW_016550125.1	38485	48583	-	360
novel.6190	NW_016550105.1	12020	14445	-	2426
novel.6191	NW_016550110.1	20722	21085	+	364
novel.6419	NW_016551895.1	1	834	-	372
novel.6418	NW_016551862.1	13253	30289	-	332
novel.6417	NW_016551862.1	77804	92900	+	2165
novel.6769	NW_016555276.1	614	1492	+	218
novel.6415	NW_016551853.1	100804	101948	+	1145
novel.6414	NW_016551853.1	99822	100535	+	246
novel.6413	NW_016551853.1	87179	88545	+	905
novel.6412	NW_016551802.1	35	970	-	838
novel.6411	NW_016551802.1	934	2086	+	1099
novel.6768	NW_016555268.1	7890	9099	-	1210
novel.1290	NW_016530123.1	5845	6076	-	232

novel.1291	NW_016530123.1	7122	7968	-	795
novel.1292	NW_016530123.1	12626	13366	-	341
novel.1293	NW_016530126.1	711	1351	+	432
novel.1294	NW_016530126.1	1464	1681	+	218
novel.1295	NW_016530127.1	17329	24330	+	7002
novel.1296	NW_016530127.1	61283	65425	+	4143
novel.1297	NW_016530131.1	69864	73924	-	261
novel.1298	NW_016530134.1	263413	264560	-	231
novel.1299	NW_016530153.1	1728	2065	-	307
novel.8185	NW_016585412.1	4855	8154	+	356
novel.4367	NW_016540097.1	24995	25515	+	498
novel.4366	NW_016540095.1	926	1822	+	877
novel.4365	NW_016540080.1	55523	57615	-	242
novel.4364	NW_016540078.1	11825	14643	-	268
novel.4363	NW_016540076.1	39204	40406	+	458
novel.4362	NW_016540076.1	12115	19720	+	809
novel.4361	NW_016540074.1	1480	1800	+	264
novel.4360	NW_016540071.1	19481	30760	-	1048
novel.4369	NW_016540109.1	3137	4237	+	1068
novel.4368	NW_016540099.1	17013	18321	+	200
novel.456	NW_016528261.1	56646	56847	+	202
novel.457	NW_016528263.1	161926	177930	+	1046
novel.454	NW_016528260.1	139113	144804	-	2454
novel.455	NW_016528260.1	146868	149684	-	424
novel.452	NW_016528256.1	30277	30740	+	320
novel.453	NW_016528260.1	145104	147050	+	1835
novel.450	NW_016528240.1	166492	168455	-	1711
novel.451	NW_016528253.1	45040	45606	+	368
novel.458	NW_016528264.1	153541	153871	+	280
novel.459	NW_016528264.1	133881	134297	-	364
novel.1878	NW_016531671.1	651972	653084	+	1074
novel.1879	NW_016531671.1	648109	653084	-	4830
novel.1874	NW_016531669.1	168171	169083	-	913
novel.1875	NW_016531669.1	168673	169083	-	345
novel.1876	NW_016531671.1	276962	312900	+	755
novel.1877	NW_016531671.1	647384	651829	+	4058
novel.1870	NW_016531660.1	272483	276287	-	3748
novel.1871	NW_016531663.1	18240	27949	+	305
novel.1872	NW_016531663.1	27697	28008	-	271
novel.1873	NW_016531665.1	5117	33172	-	269
novel.5148	NW_016543310.1	26640	26913	+	217
novel.5149	NW_016543310.1	26732	29703	-	212
novel.5144	NW_016543274.1	678	1509	-	775
novel.5145	NW_016543281.1	64661	64985	+	268

novel.5146	NW_016543291.1	164644	173908	-	1743
novel.5147	NW_016543307.1	307050	308882	-	332
novel.5140	NW_016543263.1	4050	10832	+	6729
novel.5141	NW_016543263.1	17588	17905	-	282
novel.5142	NW_016543266.1	46715	51430	-	1392
novel.5143	NW_016543267.1	24986	32018	-	229
novel.7436	NW_016564714.1	1527	6093	-	1327
novel.7437	NW_016564725.1	1	1158	-	328
novel.7434	NW_016564691.1	65045	66123	-	263
novel.7435	NW_016564714.1	9283	12001	+	467
novel.7432	NW_016564653.1	12122	30310	+	586
novel.7433	NW_016564691.1	71862	72629	+	715
novel.7430	NW_016564635.1	33172	37853	-	4068
novel.7431	NW_016564637.1	106854	111166	-	3651
novel.7128	NW_016559324.1	4566	4923	+	323
novel.7129	NW_016559337.1	2309	2676	-	326
novel.7438	NW_016564742.1	9531	10328	-	272
novel.7439	NW_016564766.1	145870	157105	-	2640
novel.8479	NW_016597020.1	1	1354	-	1354
novel.8478	NW_016597019.1	1	1354	-	1354
novel.8477	NW_016597019.1	1	1354	+	1354
novel.8476	NW_016596985.1	1	1351	+	686
novel.8475	NW_016596966.1	1	1348	-	449
novel.8474	NW_016596963.1	487	1046	-	388
novel.8473	NW_016596955.1	837	1347	-	329
novel.8472	NW_016596955.1	1	1347	-	1347
novel.8471	NW_016596953.1	933	1347	-	415
novel.8470	NW_016596946.1	1	1347	+	1347
novel.5037	NW_016542716.1	6515	9067	-	801
novel.3735	NW_016537639.1	166822	167686	-	220
novel.3734	NW_016537639.1	465643	468176	+	1296
novel.3737	NW_016537645.1	2461	28007	-	1208
novel.3736	NW_016537639.1	301391	302397	-	915
novel.3731	NW_016537625.1	12417	15220	-	534
novel.3730	NW_016537612.1	26853	30202	+	3350
novel.3733	NW_016537639.1	164215	172078	+	797
novel.3732	NW_016537628.1	58143	69019	-	1981
novel.3739	NW_016537645.1	86616	89022	-	231
novel.3738	NW_016537645.1	86208	86438	-	231
novel.275	NW_016527901.1	209459	211651	+	225
novel.4969	NW_016542445.1	2142	3141	-	227
novel.4968	NW_016542445.1	117330	120429	+	506
novel.4965	NW_016542430.1	186570	187261	-	418
novel.4964	NW_016542430.1	109513	117514	-	762

novel.4967	NW_016542430.1	252076	252462	-	331
novel.4966	NW_016542430.1	206597	268134	-	2424
novel.4961	NW_016542428.1	328228	331900	+	3673
novel.4960	NW_016542428.1	184626	214580	+	222
novel.4963	NW_016542430.1	245148	245980	+	201
novel.4962	NW_016542430.1	201578	236875	+	1802
novel.2068	NW_016532189.1	55703	56034	+	204
novel.2069	NW_016532190.1	6024	6434	+	293
novel.2062	NW_016532187.1	43141	43516	+	282
novel.2063	NW_016532187.1	235050	235360	+	263
novel.2060	NW_016532184.1	27615	27969	-	355
novel.2061	NW_016532186.1	196678	197327	+	368
novel.2066	NW_016532187.1	242668	245049	-	2382
novel.2067	NW_016532187.1	387597	388142	-	489
novel.2064	NW_016532187.1	41744	43357	-	212
novel.2065	NW_016532187.1	234163	234882	-	720
novel.2696	NW_016534110.1	284545	288035	-	2483
novel.7655	NW_016569002.1	697	7495	+	1750
novel.1609	NW_016530931.1	35803	36132	+	284
novel.1608	NW_016530929.1	290250	293059	+	1081
novel.1603	NW_016530916.1	10539	10909	+	327
novel.1602	NW_016530908.1	61	4168	+	1048
novel.1601	NW_016530905.1	1468	2049	-	582
novel.1600	NW_016530903.1	62320	62785	+	357
novel.1607	NW_016530927.1	316403	317513	+	274
novel.1606	NW_016530922.1	80875	81967	-	489
novel.1605	NW_016530920.1	56531	80320	-	3366
novel.1604	NW_016530916.1	12625	12930	+	251
novel.5779	NW_016547129.1	192328	193728	+	713
novel.5778	NW_016547128.1	618	924	+	279
novel.5773	NW_016547096.1	12439	15078	+	2489
novel.5772	NW_016547096.1	910	6976	+	5652
novel.5771	NW_016547095.1	7395	16573	-	1533
novel.5770	NW_016547092.1	78256	78623	+	311
novel.5777	NW_016547125.1	1790	2714	+	482
novel.5776	NW_016547119.1	146438	153170	-	6676
novel.5775	NW_016547118.1	157283	160357	+	3075
novel.5774	NW_016547101.1	52785	53235	+	411
novel.7383	NW_016563773.1	33245	35145	-	803
novel.139	NW_016527548.1	48109	51057	+	2924
novel.138	NW_016527538.1	632058	632697	-	418
novel.135	NW_016527537.1	303112	303991	-	713
novel.134	NW_016527537.1	65348	67783	+	2436
novel.137	NW_016527538.1	155505	239808	-	3362

novel.136	NW_016527538.1	108408	111762	+	3355
novel.131	NW_016527524.1	126672	130769	-	1012
novel.130	NW_016527524.1	8510	9101	-	423
novel.133	NW_016527533.1	728	1477	-	704
novel.132	NW_016527533.1	102	1477	-	1376
novel.7955	NW_016577264.1	469	1121	-	653
novel.7954	NW_016577224.1	4262	5886	+	1601
novel.7957	NW_016577387.1	5495	9195	-	3701
novel.7956	NW_016577387.1	5495	12744	+	2068
novel.7951	NW_016576972.1	4379	5944	+	1196
novel.7950	NW_016576810.1	9	287	+	256
novel.7953	NW_016577162.1	23888	26270	-	717
novel.7952	NW_016577101.1	10821	13735	-	239
novel.7959	NW_016577484.1	14324	14740	+	417
novel.7958	NW_016577482.1	884	4772	+	453
novel.1469	NW_016530574.1	42756	44248	-	203
novel.1468	NW_016530571.1	254000	254783	-	452
novel.1467	NW_016530571.1	252632	253767	-	1136
novel.1466	NW_016530570.1	2688	3320	-	499
novel.1465	NW_016530570.1	1172	3181	+	1873
novel.1464	NW_016530569.1	145262	159157	-	473
novel.1463	NW_016530568.1	22975	23548	-	354
novel.1462	NW_016530563.1	100793	101393	-	321
novel.1461	NW_016530563.1	77625	78470	-	644
novel.1460	NW_016530563.1	77238	78484	-	1247
novel.8181	NW_016585378.1	39928	42643	+	1669
novel.683	NW_016528784.1	93901	94592	-	692
novel.682	NW_016528780.1	221239	221811	+	573
novel.681	NW_016528776.1	42769	43566	-	768
novel.680	NW_016528776.1	39530	42541	+	1409
novel.687	NW_016528797.1	54396	58925	-	3074
novel.686	NW_016528797.1	63761	64278	+	367
novel.685	NW_016528794.1	539833	540244	-	202
novel.684	NW_016528791.1	16459	35753	+	864
novel.689	NW_016528799.1	198987	216825	+	788
novel.688	NW_016528799.1	28887	30615	+	1578
novel.6395	NW_016551604.1	1	1383	-	1356
novel.8110	NW_016582868.1	1	1474	+	1474
novel.8111	NW_016582869.1	258	577	+	298
novel.8112	NW_016582919.1	1	1526	-	1495
novel.8113	NW_016582924.1	1931	2455	+	469
novel.8114	NW_016582956.1	14147	16544	+	2239
novel.8115	NW_016582956.1	6154	7710	-	1507
novel.8116	NW_016582988.1	6969	8153	+	1185

novel.8117	NW_016582988.1	11873	12321	+	395
novel.7605	NW_016568106.1	2700	3813	-	1058
novel.7604	NW_016568098.1	81004	91483	+	270
novel.7607	NW_016568232.1	186212	188440	+	1353
novel.7606	NW_016568106.1	4513	4821	-	287
novel.7601	NW_016568031.1	55891	56833	-	943
novel.7600	NW_016568031.1	42660	47116	-	4457
novel.7603	NW_016568098.1	41333	44668	+	437
novel.7602	NW_016568092.1	429	3651	+	1605
novel.6952	NW_016557119.1	1	296	-	265
novel.6953	NW_016557124.1	83499	83864	+	327
novel.6950	NW_016557107.1	14095	14518	-	215
novel.6951	NW_016557111.1	911	1417	+	450
novel.6956	NW_016557153.1	5808	6989	-	1138
novel.6957	NW_016557158.1	8358	8889	+	359
novel.6954	NW_016557133.1	2567	45363	-	626
novel.6955	NW_016557153.1	4624	4955	-	307
novel.3128	NW_016535602.1	1	5231	+	5203
novel.3129	NW_016535616.1	33702	36301	+	277
novel.6958	NW_016557177.1	11737	13474	+	293
novel.6959	NW_016557217.1	1	679	-	653
novel.2438	NW_016533370.1	29916	32880	-	335
novel.5688	NW_016546549.1	7638	7917	-	280
novel.8806	NW_016607365.1	6650	7441	-	206
novel.8807	NW_016607397.1	1724	3415	+	227
novel.8804	NW_016607320.1	1	9544	-	2545
novel.8805	NW_016607364.1	199	1248	+	223
novel.8802	NW_016607277.1	1	3328	+	3328
novel.8803	NW_016607319.1	1134	9858	+	453
novel.8800	NW_016607245.1	142	8134	+	1179
novel.8801	NW_016607250.1	4	2001	-	559
novel.8808	NW_016607428.1	825	3866	+	618
novel.8809	NW_016607428.1	3390	6587	-	400
novel.3384	NW_016536406.1	5674	8700	+	268
novel.2862	NW_016534649.1	28439	28668	+	230
novel.3386	NW_016536412.1	6060	6773	-	410
novel.3387	NW_016536414.1	27277	32660	+	3382
novel.3380	NW_016536404.1	139681	140098	-	361
novel.2349	NW_016533126.1	155135	158265	-	239
novel.3382	NW_016536405.1	73766	74514	-	396
novel.3383	NW_016536405.1	258812	259251	-	257
novel.5428	NW_016545025.1	114415	114862	-	448
novel.3388	NW_016536414.1	5513	11326	-	783
novel.2712	NW_016534149.1	79353	80061	+	680

novel.2347	NW_016533126.1	4973	51803	+	568
novel.2713	NW_016534149.1	86111	86818	-	708
novel.2346	NW_016533121.1	243359	249802	+	290
novel.2714	NW_016534156.1	133460	139167	+	3251
novel.2345	NW_016533121.1	105337	123248	+	355
novel.2715	NW_016534156.1	224063	240034	+	607
novel.6132	NW_016549718.1	9322	9615	+	242
novel.2344	NW_016533113.1	875	2652	-	202
novel.2716	NW_016534159.1	10073	31574	+	233
novel.2343	NW_016533110.1	2297	6117	-	323
novel.2717	NW_016534159.1	18283	19369	+	855
novel.5422	NW_016545010.1	75746	80512	-	327
novel.2341	NW_016533107.1	6281	6550	-	223
novel.5420	NW_016544974.1	1886	4969	+	607
novel.5971	NW_016548640.1	1	1045	+	1023
novel.5970	NW_016548598.1	64922	78119	-	910
novel.5973	NW_016548679.1	42423	48005	-	335
novel.5972	NW_016548678.1	18088	18611	+	273
novel.5975	NW_016548696.1	7950	9746	+	1368
novel.5974	NW_016548692.1	22991	24208	+	1198
novel.5977	NW_016548736.1	6088	7220	+	408
novel.5976	NW_016548728.1	23926	24743	+	792
novel.2659	NW_016533999.1	207437	208994	-	1365
novel.2658	NW_016533999.1	490345	493074	+	2196
novel.958	NW_016529432.1	480286	482274	-	1812
novel.8657	NW_016604483.1	1	3355	+	2704
novel.8656	NW_016604396.1	1107	3292	-	275
novel.2389	NW_016533230.1	188946	189310	-	318
novel.954	NW_016529430.1	629689	630881	-	1140
novel.2383	NW_016533207.1	165881	169275	-	485
novel.2382	NW_016533197.1	403411	403910	-	463
novel.2381	NW_016533196.1	4100	4427	-	273
novel.2380	NW_016533196.1	58513	62247	+	673
novel.2387	NW_016533220.1	338516	338910	+	220
novel.2386	NW_016533213.1	6725	7141	+	389
novel.2385	NW_016533209.1	227658	228391	-	666
novel.2384	NW_016533209.1	11443	31698	-	392
novel.2475	NW_016533481.1	186835	190188	-	2166
novel.2474	NW_016533478.1	6281	13986	+	317
novel.919	NW_016529388.1	50512	52407	+	721
novel.2476	NW_016533489.1	21107	26232	+	272
novel.2471	NW_016533471.1	32551	34107	+	482
novel.951	NW_016529430.1	628311	628577	+	211
novel.2473	NW_016533475.1	34626	68728	+	778

novel.2472	NW_016533474.1	49766	51046	+	520
novel.913	NW_016529367.1	271656	272913	-	1258
novel.912	NW_016529367.1	283802	285437	+	1636
novel.911	NW_016529367.1	270811	271624	+	766
novel.910	NW_016529361.1	254199	258701	-	501
novel.917	NW_016529386.1	272508	273207	-	572
novel.916	NW_016529380.1	20091	22009	-	1875
novel.915	NW_016529380.1	76516	77781	+	327
novel.914	NW_016529372.1	8612	9580	-	859
novel.8658	NW_016604515.1	2212	3377	-	470
novel.40	NW_016527349.1	121919	122275	-	357
novel.4738	NW_016541449.1	199	1419	+	1166
novel.4739	NW_016541449.1	1	1592	-	1535
novel.4730	NW_016541423.1	6097	11614	+	307
novel.4731	NW_016541423.1	6844	12493	-	433
novel.4732	NW_016541423.1	7245	12884	-	382
novel.4733	NW_016541424.1	266	53980	+	238
novel.4734	NW_016541425.1	44612	72114	+	839
novel.4735	NW_016541432.1	9508	24011	+	1092
novel.4736	NW_016541432.1	6599	6856	-	205
novel.4737	NW_016541445.1	25912	26808	-	723
novel.6389	NW_016551580.1	889	1862	+	923
novel.6388	NW_016551560.1	39582	46199	-	438
novel.6387	NW_016551553.1	5654	8618	+	936
novel.6386	NW_016551550.1	4295	6521	+	298
novel.6385	NW_016551511.1	5844	6305	+	462
novel.6384	NW_016551498.1	24	576	-	409
novel.6383	NW_016551497.1	8841	27761	-	1736
novel.6382	NW_016551497.1	25516	27910	+	2339
novel.6381	NW_016551457.1	48413	48753	+	341
novel.6380	NW_016551445.1	15470	17108	-	1549
novel.4051	NW_016538658.1	474829	478973	-	1491
novel.4134	NW_016539074.1	7019	8530	-	1512
novel.8259	NW_016587391.1	339	1433	-	1038
novel.8258	NW_016587340.1	29	5346	-	236
novel.4135	NW_016539074.1	8593	22313	-	443
novel.8253	NW_016587126.1	247716	248284	-	540
novel.8252	NW_016587126.1	215730	216504	-	479
novel.6662	NW_016554243.1	3693	3974	+	225
novel.1816	NW_016531515.1	25976	26780	-	805
novel.1817	NW_016531517.1	5169	5462	-	249
novel.1814	NW_016531496.1	514588	514902	-	272
novel.1815	NW_016531505.1	73215	128602	+	585
novel.1812	NW_016531494.1	74119	74519	+	401

novel.1813	NW_016531494.1	496851	497153	+	303
novel.1810	NW_016531484.1	74655	75751	-	424
novel.1811	NW_016531485.1	123526	134363	+	424
novel.1818	NW_016531519.1	63069	63485	+	267
novel.1819	NW_016531519.1	63595	64083	+	244
novel.5126	NW_016543150.1	186445	187386	-	886
novel.5127	NW_016543158.1	488026	490601	+	2522
novel.5124	NW_016543150.1	187021	187556	+	262
novel.5125	NW_016543150.1	48632	50328	-	400
novel.5122	NW_016543150.1	108078	108659	+	582
novel.5123	NW_016543150.1	185119	186875	+	1699
novel.5120	NW_016543143.1	5997	7524	+	840
novel.5121	NW_016543144.1	16088	17888	-	1801
novel.5128	NW_016543158.1	436347	438505	-	1304
novel.5129	NW_016543158.1	446609	448269	-	220
novel.7418	NW_016564230.1	195439	196562	+	640
novel.7419	NW_016564281.1	11679	11943	-	265
novel.7148	NW_016559651.1	494	1353	-	860
novel.7149	NW_016559686.1	46858	47844	+	888
novel.7410	NW_016564094.1	616	1235	-	620
novel.7411	NW_016564099.1	64637	70298	+	2109
novel.7412	NW_016564099.1	181929	182558	+	630
novel.7413	NW_016564114.1	56	872	-	792
novel.7414	NW_016564118.1	38607	40022	-	1360
novel.7415	NW_016564124.1	1	1626	-	406
novel.7416	NW_016564165.1	15330	43836	-	2349
novel.7417	NW_016564165.1	46716	47565	-	686
novel.478	NW_016528307.1	418471	419111	-	641
novel.479	NW_016528309.1	290522	293265	-	2744
novel.474	NW_016528303.1	70900	71585	+	431
novel.475	NW_016528306.1	49937	58318	-	1745
novel.476	NW_016528306.1	600515	606801	-	203
novel.477	NW_016528307.1	247464	247938	-	243
novel.470	NW_016528296.1	59018	63311	-	539
novel.471	NW_016528301.1	18838	32470	-	416
novel.472	NW_016528301.1	22853	23212	-	305
novel.473	NW_016528302.1	97345	98480	+	1136
novel.8491	NW_016597908.1	396	1455	-	858
novel.8490	NW_016597877.1	4	1452	-	889
novel.8493	NW_016597961.1	577	956	+	314
novel.8492	NW_016597953.1	1	1284	+	437
novel.8495	NW_016598056.1	315	1472	+	790
novel.8494	NW_016598028.1	1	1469	+	384
novel.8497	NW_016598159.1	1	1477	-	569

novel.8496	NW_016598092.1	620	1001	+	208
novel.8499	NW_016598245.1	359	1497	+	1139
novel.8498	NW_016598221.1	1	1361	-	659
novel.8396	NW_016591916.1	11918	12592	-	620
novel.3249	NW_016535938.1	65563	66054	+	455
novel.3248	NW_016535938.1	45791	48480	+	1554
novel.3719	NW_016537579.1	7841	8840	+	579
novel.3718	NW_016537578.1	296884	297232	+	349
novel.3241	NW_016535921.1	771897	772228	+	283
novel.3240	NW_016535921.1	524302	524867	+	510
novel.3243	NW_016535921.1	771293	772017	-	299
novel.3242	NW_016535921.1	784964	785304	+	287
novel.3713	NW_016537551.1	159129	160842	-	1630
novel.3712	NW_016537551.1	96026	102195	+	5517
novel.3247	NW_016535934.1	254918	255670	+	633
novel.3710	NW_016537549.1	67990	68666	-	624
novel.4947	NW_016542386.1	633258	634809	+	1116
novel.4946	NW_016542386.1	619326	620353	+	684
novel.4945	NW_016542386.1	553702	558509	+	1018
novel.4944	NW_016542381.1	38288	51963	-	378
novel.4943	NW_016542375.1	7318	25955	+	1562
novel.4942	NW_016542374.1	144975	153029	+	206
novel.4941	NW_016542374.1	126901	133108	+	1759
novel.4940	NW_016542356.1	20892	23279	-	779
novel.4949	NW_016542386.1	641881	644119	+	995
novel.4948	NW_016542386.1	638551	641321	+	2771
novel.8390	NW_016591843.1	12716	35420	+	1803
novel.2048	NW_016532154.1	21913	26926	-	1484
novel.2049	NW_016532157.1	10026	11087	+	782
novel.2044	NW_016532153.1	17995	18810	+	816
novel.2045	NW_016532153.1	1	2096	-	2004
novel.2046	NW_016532153.1	2605	3639	-	986
novel.2047	NW_016532154.1	22158	24206	+	2049
novel.2040	NW_016532141.1	69242	69891	+	536
novel.2041	NW_016532146.1	1	1283	+	517
novel.2042	NW_016532151.1	426130	427669	-	1355
novel.2043	NW_016532153.1	2605	3639	+	1035
novel.4779	NW_016541637.1	31704	35002	+	2728
novel.4777	NW_016541626.1	62395	75368	-	203
novel.1669	NW_016531156.1	238161	242784	+	971
novel.1668	NW_016531149.1	151197	151642	-	214
novel.1665	NW_016531148.1	57646	57902	+	257
novel.1664	NW_016531144.1	112764	131029	+	209
novel.1667	NW_016531149.1	149036	151581	+	2333

novel.1666	NW_016531148.1	90617	98386	-	306
novel.1661	NW_016531131.1	5966	6745	-	544
novel.1660	NW_016531131.1	3354	6745	+	1031
novel.1663	NW_016531133.1	18	304	+	257
novel.1662	NW_016531131.1	19225	23214	-	883
novel.8762	NW_016606497.1	1643	2048	-	219
novel.5719	NW_016546770.1	211662	213551	-	1890
novel.5718	NW_016546770.1	211593	211893	+	280
novel.5715	NW_016546743.1	4029	18799	-	4151
novel.5714	NW_016546728.1	290	2520	-	749
novel.5717	NW_016546767.1	1	760	+	712
novel.5716	NW_016546743.1	10852	11550	-	642
novel.5711	NW_016546702.1	49050	50718	+	976
novel.5710	NW_016546693.1	187639	191909	+	807
novel.5713	NW_016546717.1	66	371	+	252
novel.5712	NW_016546712.1	327107	329999	-	635
novel.113	NW_016527491.1	130964	132995	-	1570
novel.112	NW_016527491.1	40978	42146	-	444
novel.111	NW_016527485.1	630985	631339	-	301
novel.110	NW_016527483.1	10269	10736	+	241
novel.117	NW_016527503.1	1458868	1462775	+	400
novel.116	NW_016527503.1	1300212	1301199	+	322
novel.7979	NW_016578287.1	10420	11690	-	1271
novel.7978	NW_016578271.1	8754	11019	-	2238
novel.7977	NW_016578271.1	1579	3677	-	279
novel.7976	NW_016578257.1	24	1583	-	269
novel.7975	NW_016578248.1	1804	5192	+	2072
novel.7974	NW_016578234.1	244	4063	+	903
novel.7973	NW_016578228.1	1707	6852	-	642
novel.7972	NW_016578168.1	303	1891	-	1589
novel.7971	NW_016578165.1	284	976	-	638
novel.7970	NW_016578089.1	6558	7060	-	503
novel.8292	NW_016588749.1	1	461	-	424
novel.1481	NW_016530596.1	68027	78812	+	3775
novel.6163	NW_016549956.1	2549	5931	+	852
novel.1483	NW_016530597.1	6508	8120	+	1565
novel.1482	NW_016530596.1	73006	75487	-	1607
novel.1485	NW_016530597.1	274690	276555	+	325
novel.1484	NW_016530597.1	246294	278664	+	1058
novel.1487	NW_016530597.1	278754	280861	-	218
novel.6162	NW_016549956.1	1861	2340	+	456
novel.1489	NW_016530606.1	97425	98616	-	1171
novel.1488	NW_016530597.1	402525	406114	-	473
novel.6161	NW_016549952.1	45608	46101	-	439

novel.6160	NW_016549944.1	5707	8249	+	2486
novel.6167	NW_016549990.1	188006	189759	-	912
novel.6166	NW_016549990.1	115680	118088	-	344
novel.6165	NW_016549963.1	61467	61913	+	400
novel.6164	NW_016549963.1	59849	61189	+	1193
novel.6598	NW_016553598.1	4167	4621	-	250
novel.6599	NW_016553627.1	14645	15161	+	517
novel.6592	NW_016553553.1	9060	10976	-	1895
novel.6593	NW_016553553.1	11004	11990	-	930
novel.6590	NW_016553545.1	1	559	+	537
novel.6591	NW_016553553.1	9060	11990	+	2807
novel.6596	NW_016553587.1	18525	19094	-	521
novel.6597	NW_016553591.1	60256	61112	+	857
novel.6594	NW_016553571.1	100346	137593	+	1162
novel.6595	NW_016553571.1	245493	245781	-	261
novel.6168	NW_016550000.1	4616	6118	+	1458
novel.4442	NW_016540368.1	434779	435078	-	257
novel.4443	NW_016540371.1	178235	179791	+	295
novel.4440	NW_016540359.1	105765	106852	-	619
novel.4441	NW_016540368.1	230431	254027	+	983
novel.4446	NW_016540371.1	396282	397548	-	1050
novel.4447	NW_016540371.1	399956	402005	-	1012
novel.4444	NW_016540371.1	408097	427570	+	1357
novel.4445	NW_016540371.1	166108	167974	-	696
novel.4448	NW_016540373.1	37388	41953	+	1231
novel.4449	NW_016540373.1	382863	383332	+	414
novel.7627	NW_016568657.1	107	800	+	668
novel.7626	NW_016568651.1	354	1536	-	800
novel.7625	NW_016568606.1	1	308	-	251
novel.7624	NW_016568569.1	890	1155	-	227
novel.7623	NW_016568557.1	4907	5480	-	548
novel.7622	NW_016568551.1	5244	6480	-	324
novel.7621	NW_016568548.1	1	697	+	665
novel.7620	NW_016568542.1	8261	9090	-	460
novel.6624	NW_016553900.1	2122	4718	-	242
novel.6625	NW_016553903.1	81069	110906	+	281
novel.6626	NW_016553906.1	945	1656	+	302
novel.6627	NW_016553920.1	5902	9325	-	274
novel.6620	NW_016553858.1	18208	34236	+	633
novel.6621	NW_016553883.1	701	1791	-	684
novel.7629	NW_016568697.1	1	1835	-	1805
novel.7628	NW_016568697.1	1	1835	+	1796
novel.6974	NW_016557445.1	65144	105716	+	1864
novel.6975	NW_016557449.1	224	8295	-	315

novel.6976	NW_016557449.1	92972	96255	-	600
novel.6977	NW_016557470.1	42348	42595	-	248
novel.6970	NW_016557438.1	13881	14288	+	408
novel.6971	NW_016557438.1	82927	85427	+	385
novel.6972	NW_016557438.1	86500	87595	+	506
novel.6973	NW_016557438.1	97942	101492	+	998
novel.6978	NW_016557474.1	43055	45699	+	601
novel.6979	NW_016557475.1	16458	17331	-	367
novel.8238	NW_016586912.1	2771	3883	-	1113
novel.5979	NW_016548736.1	30176	31001	-	496
novel.8828	NW_016607646.1	1	8736	-	8736
novel.8829	NW_016607666.1	1	4143	+	3870
novel.4196	NW_016539336.1	4246	4895	+	593
novel.4197	NW_016539336.1	181086	182886	+	507
novel.4190	NW_016539310.1	561	793	+	203
novel.4191	NW_016539320.1	308441	309642	+	817
novel.8398	NW_016591922.1	69395	72977	+	821
novel.8399	NW_016591925.1	3767	5061	+	478
novel.8820	NW_016607527.1	11846	12283	-	290
novel.8821	NW_016607532.1	4393	7094	-	428
novel.8822	NW_016607536.1	1928	10743	-	5315
novel.8823	NW_016607539.1	195	10447	-	201
novel.8824	NW_016607569.1	1044	8079	+	2554
novel.8825	NW_016607574.1	10704	12862	+	481
novel.8826	NW_016607632.1	8951	10011	-	1061
novel.8827	NW_016607646.1	1	8672	+	8672
novel.1636	NW_016531014.1	16171	17354	-	1142
novel.5978	NW_016548736.1	17315	27345	+	684
novel.5556	NW_016545734.1	8774	9347	+	574
novel.2818	NW_016534477.1	25483	25792	+	310
novel.5555	NW_016545713.1	4880	16264	-	297
novel.1638	NW_016531014.1	45574	46058	-	354
novel.7083	NW_016558656.1	95512	95915	+	404
novel.7082	NW_016558653.1	30752	32382	-	1631
novel.7081	NW_016558638.1	3249	4390	-	1096
novel.7080	NW_016558620.1	122331	123126	-	370
novel.7087	NW_016558762.1	1479	1876	+	398
novel.7086	NW_016558737.1	1	269	+	269
novel.7085	NW_016558707.1	696	1095	-	361
novel.7084	NW_016558689.1	1828	6245	+	380
novel.7089	NW_016558780.1	14108	18830	+	765
novel.7088	NW_016558776.1	12373	28475	+	373
novel.5959	NW_016548505.1	124265	126938	-	688
novel.5958	NW_016548505.1	121691	123057	-	1367

novel.8758	NW_016606344.1	1	2121	+	1439
novel.5953	NW_016548505.1	16842	19372	+	1854
novel.5952	NW_016548494.1	8811	9394	+	555
novel.5951	NW_016548481.1	293189	293797	+	585
novel.5950	NW_016548481.1	57682	58624	+	674
novel.5957	NW_016548505.1	1899	4007	-	2035
novel.5956	NW_016548505.1	152364	153309	+	539
novel.5955	NW_016548505.1	149976	151885	+	1094
novel.5954	NW_016548505.1	123839	127312	+	1337
novel.5311	NW_016544301.1	27265	40709	+	869
novel.8538	NW_016600155.1	518	1780	+	721
novel.8539	NW_016600192.1	246	1786	-	1541
novel.5310	NW_016544298.1	84984	93815	+	2863
novel.8532	NW_016599700.1	1	1700	-	1700
novel.8533	NW_016599798.1	1	1717	-	941
novel.8530	NW_016599653.1	494	1688	-	1074
novel.8531	NW_016599682.1	1	693	+	693
novel.8536	NW_016599963.1	1	1744	-	1744
novel.8537	NW_016600152.1	1	835	-	229
novel.8534	NW_016599841.1	1	1724	+	277
novel.8535	NW_016599937.1	143	548	+	406
novel.5314	NW_016544306.1	2696	3170	+	409
novel.8750	NW_016606226.1	3525	5468	-	1419
novel.5552	NW_016545658.1	98666	99088	+	423
novel.5838	NW_016547589.1	80380	80774	-	339
novel.8751	NW_016606235.1	4181	5506	+	1157
novel.5839	NW_016547593.1	3583	6448	+	2330
novel.2909	NW_016534805.1	27794	28575	-	616
novel.2908	NW_016534802.1	10142	27475	-	2138
novel.5489	NW_016545398.1	54536	54839	+	251
novel.5488	NW_016545388.1	47976	51769	+	345
novel.2459	NW_016533426.1	272531	274047	+	1257
novel.2458	NW_016533426.1	23437	24560	+	1124
novel.5485	NW_016545368.1	59102	61512	-	2357
novel.2456	NW_016533421.1	44616	47388	-	804
novel.5487	NW_016545375.1	5677	7318	+	430
novel.5486	NW_016545372.1	4048	20842	-	870
novel.5481	NW_016545355.1	301081	301534	-	360
novel.5480	NW_016545355.1	63774	65812	-	1567
novel.2451	NW_016533406.1	257753	258061	-	309
novel.2450	NW_016533403.1	56710	57248	-	486
novel.2634	NW_016533943.1	135066	136840	-	1139
novel.2633	NW_016533943.1	115009	119281	-	1712
novel.5836	NW_016547574.1	1	7398	+	307

novel.2632	NW_016533943.1	102658	104583	+	1926
novel.2457	NW_016533421.1	79476	79945	-	284
novel.5837	NW_016547582.1	4465	5413	+	835
novel.2631	NW_016533938.1	45059	45365	+	262
novel.6130	NW_016549695.1	251	1194	+	889
novel.2630	NW_016533938.1	16985	20028	+	2600
novel.3230	NW_016535885.1	673	2951	-	238
novel.3231	NW_016535887.1	117529	135308	-	533
novel.7928	NW_016576116.1	1302	3797	+	266
novel.3232	NW_016535893.1	14570	16467	-	504
novel.7929	NW_016576116.1	4093	4551	+	459
novel.3233	NW_016535896.1	68597	73430	+	555
novel.6131	NW_016549697.1	44869	50484	-	488
novel.3234	NW_016535902.1	5043	6653	-	1501
novel.3235	NW_016535903.1	273888	274172	-	260
novel.3236	NW_016535905.1	104255	112739	-	296
novel.3237	NW_016535907.1	43859	45184	-	536
novel.7926	NW_016576045.1	369	1629	-	1233
novel.2455	NW_016533421.1	22245	22554	-	310
novel.7223	NW_016560962.1	5336	5760	-	240
novel.6028	NW_016549058.1	6319	7097	-	330
novel.6029	NW_016549070.1	9118	14100	+	471
novel.7222	NW_016560947.1	21252	21820	+	521
novel.6022	NW_016549042.1	26414	27392	+	567
novel.6023	NW_016549042.1	31400	33609	+	2005
novel.6020	NW_016549017.1	2715	3089	+	375
novel.6021	NW_016549041.1	17349	17851	+	503
novel.6026	NW_016549050.1	30435	31375	-	317
novel.6027	NW_016549052.1	133	2540	+	634
novel.6024	NW_016549042.1	35846	36541	+	642
novel.6025	NW_016549042.1	30288	31568	-	1197
novel.878	NW_016529274.1	829181	829422	-	206
novel.7220	NW_016560911.1	294	882	+	446
novel.7227	NW_016561046.1	15788	17309	+	1238
novel.7226	NW_016560991.1	22347	23039	+	489
novel.6133	NW_016549728.1	17842	40713	+	1642
novel.8345	NW_016590742.1	1205	1441	+	201
novel.7225	NW_016560991.1	14430	15093	+	664
novel.7224	NW_016560991.1	7432	7657	+	226
novel.2453	NW_016533417.1	40685	41017	-	277
novel.6134	NW_016549737.1	1136	2095	-	283
novel.6361	NW_016551298.1	6687	7039	+	304
novel.6360	NW_016551295.1	7917	8406	-	490
novel.6363	NW_016551316.1	12986	37818	+	699

novel.6362	NW_016551306.1	7674	22656	-	510
novel.6365	NW_016551316.1	17298	24485	+	544
novel.6364	NW_016551316.1	13808	16443	+	2580
novel.6367	NW_016551316.1	17447	18009	-	563
novel.6366	NW_016551316.1	13808	16443	-	2615
novel.6369	NW_016551336.1	95359	95723	-	279
novel.6368	NW_016551326.1	1	1341	-	1341
novel.2452	NW_016533417.1	35111	35361	+	251
novel.6135	NW_016549738.1	587	1048	+	214
novel.781	NW_016529048.1	66629	67001	+	228
novel.6136	NW_016549753.1	8295	9188	+	389
novel.1456	NW_016530550.1	80768	81228	-	461
novel.1457	NW_016530551.1	11357	16676	+	447
novel.1454	NW_016530546.1	65217	65590	+	374
novel.6043	NW_016549168.1	15065	30955	+	551
novel.6137	NW_016549758.1	1	8359	-	349
novel.1452	NW_016530533.1	45719	46746	-	979
novel.1981	NW_016531955.1	202765	203780	-	466
novel.1982	NW_016531956.1	572685	572999	+	315
novel.1983	NW_016531956.1	13059	20915	-	264
novel.8848	NW_016607865.1	21386	23922	-	1410
novel.8749	NW_016606220.1	1149	4651	+	1793
novel.1838	NW_016531553.1	6851	13759	+	205
novel.1839	NW_016531557.1	121972	124898	+	839
novel.1348	NW_016530268.1	856563	860200	-	3522
novel.1349	NW_016530271.1	150504	153928	+	206
novel.1342	NW_016530250.1	512422	513077	+	656
novel.1343	NW_016530250.1	494163	500494	-	6332
novel.1340	NW_016530248.1	13841	26976	-	298
novel.1341	NW_016530250.1	496830	500493	+	3664
novel.1346	NW_016530266.1	210559	251321	+	266
novel.1347	NW_016530268.1	451627	455440	+	3625
novel.1344	NW_016530258.1	299492	300225	-	711
novel.1345	NW_016530264.1	732	11206	+	237
novel.5108	NW_016543057.1	29150	35235	-	6086
novel.5109	NW_016543057.1	31357	35236	-	3823
novel.5100	NW_016543015.1	452345	456530	-	334
novel.5101	NW_016543033.1	453403	455409	+	1731
novel.5102	NW_016543033.1	353464	453214	-	2233
novel.5103	NW_016543035.1	292159	292633	+	314
novel.5104	NW_016543039.1	81825	82243	+	270
novel.5105	NW_016543055.1	526591	540734	+	322
novel.5106	NW_016543055.1	688793	693921	+	1930
novel.5107	NW_016543055.1	671949	673068	-	468

novel.8701	NW_016605360.1	2592	4186	-	1595
novel.8700	NW_016605359.1	930	2022	+	1093
novel.8703	NW_016605380.1	3372	4200	+	280
novel.8702	NW_016605367.1	330	3774	+	247
novel.8705	NW_016605403.1	1143	1905	-	763
novel.8704	NW_016605396.1	1324	2084	+	224
novel.8707	NW_016605419.1	10	3183	+	610
novel.8706	NW_016605412.1	1192	3118	+	1346
novel.8709	NW_016605443.1	979	4287	+	2925
novel.8708	NW_016605443.1	8	805	+	798
novel.2940	NW_016534937.1	407279	407677	-	399
novel.3887	NW_016538127.1	131538	133273	-	1684
novel.3886	NW_016538127.1	21539	22293	-	385
novel.3885	NW_016538126.1	1324	5802	-	755
novel.3884	NW_016538126.1	5175	5802	+	628
novel.3883	NW_016538124.1	997	33852	+	1617
novel.3882	NW_016538121.1	722	2791	-	2013
novel.3881	NW_016538121.1	722	1591	+	870
novel.3880	NW_016538108.1	20803	22175	+	522
novel.3889	NW_016538137.1	126046	129206	+	1005
novel.3888	NW_016538135.1	62385	62936	+	523
novel.492	NW_016528357.1	167639	174506	-	722
novel.493	NW_016528360.1	16877	18001	-	960
novel.490	NW_016528357.1	176055	177743	+	269
novel.491	NW_016528357.1	130230	131145	-	916
novel.496	NW_016528366.1	69235	72189	+	2898
novel.497	NW_016528366.1	360205	360584	+	380
novel.494	NW_016528362.1	5805	7285	+	1481
novel.495	NW_016528365.1	165892	213758	+	488
novel.498	NW_016528366.1	347821	349036	-	1216
novel.499	NW_016528366.1	408279	408877	-	297
novel.8272	NW_016587717.1	2406	3976	+	366
novel.3089	NW_016535526.1	397476	402482	+	270
novel.3088	NW_016535526.1	29993	31245	+	399
novel.3087	NW_016535525.1	18218	46799	-	1263
novel.3086	NW_016535522.1	169204	170106	+	652
novel.3085	NW_016535508.1	587	1782	+	1146
novel.3084	NW_016535505.1	231249	232233	-	928
novel.3083	NW_016535505.1	35084	37432	-	2036
novel.3082	NW_016535505.1	230960	233639	+	2525
novel.3081	NW_016535501.1	37013	53675	+	224
novel.3080	NW_016535498.1	11121	11705	+	415
novel.4279	NW_016539705.1	36268	42339	+	425
novel.4278	NW_016539692.1	109208	110312	+	1062

novel.4271	NW_016539676.1	1	4415	+	2897
novel.4270	NW_016539660.1	289729	290415	-	620
novel.4273	NW_016539676.1	17734	20875	+	3110
novel.4272	NW_016539676.1	8729	10933	+	2205
novel.4275	NW_016539676.1	10186	11435	-	1250
novel.4274	NW_016539676.1	1	4415	-	4106
novel.4277	NW_016539691.1	106	936	+	288
novel.4276	NW_016539685.1	25396	84552	-	773
novel.3263	NW_016535966.1	880	1569	+	690
novel.3262	NW_016535961.1	1820	29918	-	1017
novel.3261	NW_016535960.1	115279	116587	-	1257
novel.3260	NW_016535960.1	112467	112957	-	379
novel.3267	NW_016536002.1	11997	12387	+	284
novel.3266	NW_016536002.1	3474	11608	+	353
novel.3265	NW_016535998.1	122450	122794	-	292
novel.3264	NW_016535998.1	122450	122772	+	271
novel.3269	NW_016536006.1	125610	125981	-	255
novel.3268	NW_016536006.1	122222	126426	+	1533
novel.6830	NW_016555842.1	2904	3805	+	530
novel.225	NW_016527730.1	69926	70623	-	698
novel.8844	NW_016607849.1	2559	5767	+	1242
novel.2026	NW_016532105.1	161434	161733	-	248
novel.2027	NW_016532105.1	198305	198806	-	387
novel.2024	NW_016532097.1	1	383	+	349
novel.2025	NW_016532097.1	2139	2493	+	300
novel.2022	NW_016532082.1	5230	5645	+	360
novel.2023	NW_016532085.1	36183	70677	+	485
novel.2020	NW_016532071.1	281	1441	+	229
novel.2021	NW_016532078.1	69543	72415	-	617
novel.2028	NW_016532109.1	155415	156258	+	343
novel.2029	NW_016532115.1	23823	61616	+	4254
novel.1131	NW_016529808.1	185862	208044	+	2787
novel.1130	NW_016529805.1	83032	83684	-	604
novel.1133	NW_016529811.1	399319	403565	+	4247
novel.1132	NW_016529808.1	205336	208044	-	2709
novel.1135	NW_016529826.1	206054	209660	+	237
novel.1134	NW_016529823.1	20699	21372	+	625
novel.1137	NW_016529833.1	628	1280	-	653
novel.1136	NW_016529830.1	4220	4603	-	221
novel.1139	NW_016529846.1	158752	203328	-	638
novel.1856	NW_016531610.1	82	3519	+	3438
novel.1649	NW_016531060.1	507082	508937	+	1200
novel.1648	NW_016531050.1	168949	169474	+	316
novel.5737	NW_016546916.1	55782	60892	+	1607

novel.5736	NW_016546911.1	156036	156355	-	320
novel.5735	NW_016546906.1	3156	6234	-	340
novel.5734	NW_016546906.1	1844	2467	-	567
novel.5733	NW_016546876.1	107973	108284	+	312
novel.5732	NW_016546853.1	59	388	-	278
novel.5731	NW_016546844.1	414	2116	-	1646
novel.5730	NW_016546844.1	414	1904	+	1491
novel.1855	NW_016531605.1	375456	383514	-	476
novel.5739	NW_016546948.1	128861	139971	+	706
novel.5738	NW_016546948.1	24753	28637	+	841
novel.7991	NW_016578491.1	3920	5825	-	718
novel.7990	NW_016578487.1	8950	9942	-	945
novel.7993	NW_016578555.1	971	2015	+	274
novel.7992	NW_016578494.1	431	884	-	418
novel.7995	NW_016578596.1	1607	1917	-	255
novel.7994	NW_016578596.1	3720	4405	+	486
novel.7997	NW_016578619.1	12903	64037	-	1538
novel.7996	NW_016578619.1	61708	62674	+	967
novel.7999	NW_016578666.1	700	990	+	271
novel.7998	NW_016578627.1	5117	5806	+	690
novel.5432	NW_016545052.1	54688	55320	-	254
novel.399	NW_016528118.1	22102	22420	+	287
novel.398	NW_016528115.1	256047	278137	+	778
novel.395	NW_016528108.1	496489	499350	-	779
novel.394	NW_016528108.1	116362	119537	-	3176
novel.397	NW_016528111.1	369801	374749	-	286
novel.396	NW_016528111.1	102533	103698	-	1166
novel.391	NW_016528100.1	61670	62258	-	435
novel.390	NW_016528100.1	14157	15209	-	1023
novel.393	NW_016528105.1	48423	50517	+	1859
novel.392	NW_016528102.1	55079	56512	-	1434
novel.8231	NW_016586846.1	284	2144	-	1808
novel.5434	NW_016545053.1	86958	87332	-	321
novel.2017	NW_016532065.1	415642	416067	-	334
novel.2016	NW_016532062.1	51948	55472	+	273
novel.5435	NW_016545055.1	21551	23041	+	1491
novel.2015	NW_016532061.1	60824	70057	-	912
novel.4464	NW_016540462.1	268226	271818	+	279
novel.4465	NW_016540462.1	6552	10920	-	1025
novel.4466	NW_016540462.1	70785	71279	-	495
novel.2542	NW_016533699.1	73549	77242	+	420
novel.4460	NW_016540431.1	43191	44667	+	264
novel.4461	NW_016540448.1	33196	33552	+	269
novel.4462	NW_016540451.1	49588	49877	-	268

novel.4463	NW_016540462.1	9437	9815	+	323
novel.2013	NW_016532061.1	157	4955	-	1689
novel.4468	NW_016540462.1	289173	290333	-	589
novel.1069	NW_016529668.1	407236	408296	+	1061
novel.2012	NW_016532060.1	2136	2600	+	465
novel.6646	NW_016554103.1	9678	10244	-	533
novel.6647	NW_016554116.1	1	34689	-	1016
novel.6644	NW_016554101.1	46857	48179	-	201
novel.6645	NW_016554103.1	6255	6511	-	212
novel.6642	NW_016554090.1	79254	79723	+	413
novel.6643	NW_016554098.1	102983	103196	-	214
novel.6640	NW_016554077.1	45273	45517	-	207
novel.6641	NW_016554084.1	1053	1364	+	263
novel.5436	NW_016545062.1	5845	6053	+	209
novel.2010	NW_016532056.1	139994	146170	+	3544
novel.6648	NW_016554128.1	67997	68531	+	468
novel.6649	NW_016554128.1	67340	67645	-	269
novel.539	NW_016528448.1	43375	48080	+	470
novel.538	NW_016528440.1	142997	143343	-	347
novel.602	NW_016528579.1	28659	34503	+	480
novel.531	NW_016528425.1	48751	84190	+	1190
novel.530	NW_016528424.1	137544	138045	+	392
novel.533	NW_016528429.1	13725	15569	+	248
novel.532	NW_016528426.1	126405	127905	-	286
novel.535	NW_016528429.1	501038	502570	+	209
novel.534	NW_016528429.1	156595	161415	+	435
novel.537	NW_016528429.1	437085	438262	-	463
novel.536	NW_016528429.1	686570	779327	+	3268
novel.3545	NW_016537061.1	17360	17778	-	252
novel.5437	NW_016545063.1	47122	48058	+	231
novel.3542	NW_016537056.1	16305	17864	+	1524
novel.3543	NW_016537056.1	3632	3862	-	231
novel.2836	NW_016534545.1	3900	4434	+	465
novel.3540	NW_016537051.1	79550	80895	+	288
novel.3541	NW_016537056.1	5059	5674	+	616
novel.7101	NW_016559113.1	33786	34095	-	310
novel.6288	NW_016550725.1	11409	45836	-	2772
novel.7106	NW_016559120.1	20530	21776	+	346
novel.6289	NW_016550730.1	10145	11229	-	349
novel.7459	NW_016565347.1	3711	16384	+	243
novel.2831	NW_016534532.1	247136	262698	+	234
novel.3548	NW_016537074.1	56987	57477	-	257
novel.7455	NW_016565319.1	13980	23127	+	547
novel.3549	NW_016537074.1	103670	105290	-	1142

novel.7456	NW_016565319.1	20074	21994	+	841
novel.7457	NW_016565333.1	4466	4850	-	303
novel.6918	NW_016556848.1	3901	4290	+	358
novel.6919	NW_016556848.1	3694	4345	-	399
novel.6916	NW_016556829.1	157632	208180	-	501
novel.6917	NW_016556848.1	3242	3629	+	333
novel.6914	NW_016556799.1	3706	6468	-	2708
novel.6915	NW_016556824.1	2096	2550	-	207
novel.6912	NW_016556799.1	6894	8573	+	1575
novel.6913	NW_016556799.1	9900	15155	+	3114
novel.6910	NW_016556797.1	2825	4439	-	1615
novel.6911	NW_016556798.1	8497	11052	-	2556
novel.2638	NW_016533948.1	53550	54733	+	905
novel.5939	NW_016548340.1	3534	4974	-	465
novel.5938	NW_016548327.1	1146	1511	+	311
novel.5935	NW_016548322.1	329541	330424	-	884
novel.5934	NW_016548322.1	265259	265965	-	547
novel.5937	NW_016548324.1	44387	44700	+	263
novel.5936	NW_016548322.1	330597	331511	-	413
novel.5931	NW_016548322.1	108001	108478	-	312
novel.5930	NW_016548322.1	287854	289396	+	1543
novel.5933	NW_016548322.1	223554	230918	-	1003
novel.5932	NW_016548322.1	135105	136708	-	928
novel.7209	NW_016560639.1	104653	105033	-	325
novel.7208	NW_016560576.1	57392	59886	+	955
novel.8518	NW_016599194.1	550	1461	+	824
novel.8519	NW_016599266.1	1	1637	+	374
novel.8514	NW_016599087.1	1	318	+	318
novel.8515	NW_016599127.1	568	1391	+	565
novel.8516	NW_016599154.1	1	908	-	570
novel.8517	NW_016599159.1	1	1372	+	1372
novel.8510	NW_016598930.1	1	1589	+	1589
novel.8511	NW_016598962.1	1	1163	-	1163
novel.8512	NW_016598975.1	467	1596	-	1130
novel.8513	NW_016598983.1	2	1597	+	1596
novel.1711	NW_016531259.1	463318	472616	-	9299
novel.1710	NW_016531259.1	458253	458848	+	562
novel.1713	NW_016531264.1	17064	17387	-	324
novel.1712	NW_016531260.1	14275	20718	-	610
novel.1715	NW_016531278.1	214515	214828	+	285
novel.1714	NW_016531278.1	176792	177441	+	599
novel.2923	NW_016534884.1	469877	470255	-	311
novel.2922	NW_016534878.1	222734	226374	-	1098
novel.2921	NW_016534874.1	531479	539645	+	795

novel.2920	NW_016534869.1	5960	6413	+	400
novel.2927	NW_016534911.1	1	827	+	407
novel.2926	NW_016534908.1	357018	361855	-	245
novel.2925	NW_016534896.1	176443	177076	-	611
novel.2924	NW_016534894.1	85300	90295	+	324
novel.2929	NW_016534915.1	1	1082	+	1082
novel.1716	NW_016531278.1	2196	2852	-	298
novel.3698	NW_016537517.1	163947	164591	+	297
novel.3699	NW_016537525.1	3278	3606	+	290
novel.3692	NW_016537507.1	78984	79466	-	433
novel.3693	NW_016537508.1	51556	52513	-	958
novel.3690	NW_016537503.1	22851	113178	+	1913
novel.3691	NW_016537507.1	79446	81429	+	1935
novel.3696	NW_016537514.1	255297	255578	-	282
novel.3697	NW_016537514.1	267253	268009	-	732
novel.3694	NW_016537512.1	138303	139095	+	241
novel.3695	NW_016537514.1	94225	96776	-	2110
novel.8329	NW_016590180.1	53623	56178	+	1039
novel.4162	NW_016539214.1	13294	20852	-	3174
novel.8851	NW_016607898.1	303	8281	+	2150
novel.8850	NW_016607885.1	1	11808	+	577
novel.8325	NW_016590105.1	39426	39976	+	551
novel.8324	NW_016590105.1	2732	3848	+	676
novel.6008	NW_016548913.1	10445	11023	-	529
novel.6009	NW_016548941.1	89601	90792	+	406
novel.6004	NW_016548894.1	195678	195959	+	228
novel.4169	NW_016539231.1	50903	53285	-	970
novel.6006	NW_016548913.1	10226	10756	+	435
novel.6007	NW_016548913.1	46687	48024	+	1296
novel.6000	NW_016548838.1	15329	18035	-	413
novel.6001	NW_016548838.1	66472	72933	-	2950
novel.6002	NW_016548859.1	15732	16056	+	269
novel.6003	NW_016548860.1	1118	3763	-	309
novel.8321	NW_016589985.1	8881	9595	+	294
novel.8320	NW_016589985.1	412	1709	+	1245
novel.6349	NW_016551243.1	25481	26033	+	233
novel.6348	NW_016551243.1	19250	25078	+	254
novel.6343	NW_016551172.1	24781	25397	-	617
novel.6342	NW_016551172.1	24781	25397	+	302
novel.6341	NW_016551150.1	15420	39407	-	1601
novel.6340	NW_016551128.1	38544	40839	-	2296
novel.6347	NW_016551223.1	1	2199	+	2166
novel.6346	NW_016551210.1	21708	23287	+	1146
novel.6345	NW_016551206.1	4248	5914	-	1469

novel.6344	NW_016551204.1	1991	2413	-	376
novel.5575	NW_016545872.1	62	433	-	315
novel.6185	NW_016550069.1	525	1038	+	482
novel.6184	NW_016550053.1	179031	183600	-	4570
novel.6187	NW_016550081.1	126529	127444	-	444
novel.6186	NW_016550069.1	286	923	-	588
novel.6181	NW_016550053.1	179005	183600	+	4596
novel.5574	NW_016545872.1	110069	110707	+	639
novel.6180	NW_016550053.1	168689	171003	+	2315
novel.6183	NW_016550053.1	151685	151941	-	257
novel.6182	NW_016550053.1	31763	35700	-	835
novel.88	NW_016527431.1	68575	71830	+	1034
novel.89	NW_016527432.1	233244	233646	+	234
novel.84	NW_016527428.1	10	821	+	765
novel.85	NW_016527428.1	2666	3614	+	745
novel.86	NW_016527428.1	41857	43552	+	1042
novel.87	NW_016527428.1	2330	3008	-	577
novel.80	NW_016527420.1	427799	428131	-	276
novel.81	NW_016527421.1	60779	61502	-	506
novel.82	NW_016527426.1	41378	42106	-	729
novel.83	NW_016527427.1	83493	84048	-	226
novel.1588	NW_016530862.1	247247	249736	+	2490
novel.1589	NW_016530862.1	327930	328838	+	909
novel.1580	NW_016530832.1	172839	173433	-	595
novel.1581	NW_016530837.1	29013	29275	+	263
novel.1582	NW_016530837.1	31572	32107	+	488
novel.1583	NW_016530841.1	17351	17727	-	223
novel.1584	NW_016530842.1	2	723	-	697
novel.1585	NW_016530843.1	369833	378022	-	621
novel.1586	NW_016530843.1	393841	397338	-	815
novel.1587	NW_016530850.1	31584	52293	+	312
novel.5698	NW_016546634.1	14996	33893	+	221
novel.5699	NW_016546641.1	226210	230468	+	600
novel.5690	NW_016546582.1	3419	9601	-	342
novel.5691	NW_016546584.1	133335	133712	-	322
novel.5692	NW_016546588.1	108491	108882	+	392
novel.5693	NW_016546597.1	2682	2916	-	207
novel.5694	NW_016546600.1	5580	6644	+	228
novel.5695	NW_016546607.1	2545	5813	-	1072
novel.5696	NW_016546621.1	37	791	+	316
novel.5697	NW_016546627.1	9967	22285	+	638
novel.7838	NW_016573530.1	1023	3271	+	1575
novel.7839	NW_016573720.1	199731	200039	+	255
novel.7832	NW_016573358.1	832	1652	-	791

novel.7833	NW_016573441.1	8096	12401	+	592
novel.7830	NW_016573336.1	953	1387	+	435
novel.7831	NW_016573350.1	2076	2343	+	227
novel.7836	NW_016573491.1	10317	11181	-	812
novel.7837	NW_016573520.1	2198	3319	-	1122
novel.2819	NW_016534488.1	4943	31669	-	1208
novel.7835	NW_016573491.1	10627	11599	+	771
novel.1368	NW_016530311.1	4113	5754	-	1510
novel.1369	NW_016530312.1	117931	136835	+	505
novel.1364	NW_016530300.1	167431	169247	-	711
novel.1365	NW_016530304.1	37781	40549	-	890
novel.1366	NW_016530307.1	172361	173485	+	1125
novel.1367	NW_016530307.1	172374	173466	-	795
novel.1360	NW_016530294.1	347938	348406	+	418
novel.1361	NW_016530294.1	348492	348775	-	227
novel.1362	NW_016530295.1	68440	69239	+	761
novel.1363	NW_016530297.1	207251	301238	-	1432
novel.702	NW_016528825.1	731407	735508	+	3925
novel.703	NW_016528825.1	1124159	1212388	+	768
novel.700	NW_016528823.1	41530	41791	+	236
novel.701	NW_016528823.1	44504	44783	-	224
novel.706	NW_016528826.1	254719	255551	+	833
novel.707	NW_016528826.1	483839	487434	+	3342
novel.704	NW_016528825.1	1153348	1153995	+	478
novel.705	NW_016528825.1	731469	734544	-	3020
novel.708	NW_016528826.1	446526	486124	-	2192
novel.709	NW_016528828.1	11206	11593	+	262
novel.2477	NW_016533494.1	82020	82724	-	396
novel.3664	NW_016537405.1	137247	137812	-	510
novel.8217	NW_016586378.1	1387	2892	+	548
novel.3061	NW_016535434.1	179513	182420	+	489
novel.3060	NW_016535430.1	244563	275179	+	364
novel.3063	NW_016535434.1	179513	184350	-	2701
novel.3062	NW_016535434.1	183408	184021	+	266
novel.3065	NW_016535437.1	14505	14788	+	227
novel.3064	NW_016535437.1	2612	2873	+	215
novel.3067	NW_016535442.1	312402	312985	+	473
novel.3066	NW_016535439.1	37180	38844	+	885
novel.3069	NW_016535442.1	319384	322058	-	2467
novel.3068	NW_016535442.1	16665	17810	-	452
novel.8216	NW_016586363.1	1	532	-	477
novel.4253	NW_016539578.1	451	867	-	203
novel.4252	NW_016539578.1	55114	55832	+	662
novel.8729	NW_016605777.1	420	3021	+	889

novel.8728	NW_016605759.1	607	3362	-	356
novel.4257	NW_016539599.1	17781	18110	-	330
novel.4256	NW_016539591.1	7856	15987	+	302
novel.4255	NW_016539589.1	31165	31750	+	538
novel.4254	NW_016539578.1	32255	60587	-	290
novel.8723	NW_016605679.1	2088	4574	-	311
novel.8722	NW_016605666.1	6	2795	+	833
novel.8721	NW_016605665.1	1	1809	+	1310
novel.8720	NW_016605647.1	1	4534	+	4534
novel.8727	NW_016605752.1	3331	4676	+	306
novel.8726	NW_016605720.1	606	3597	+	2992
novel.8725	NW_016605716.1	1535	2900	+	623
novel.8724	NW_016605704.1	37	4575	+	4539
novel.8125	NW_016583288.1	7878	11414	+	572
novel.8617	NW_016603015.1	772	1530	+	210
novel.3205	NW_016535814.1	5985	6617	+	582
novel.3204	NW_016535812.1	14966	34275	+	271
novel.3207	NW_016535828.1	538211	538676	-	466
novel.3206	NW_016535823.1	259789	288679	+	349
novel.3201	NW_016535795.1	7214	7608	-	395
novel.3200	NW_016535795.1	16981	17988	+	1008
novel.3203	NW_016535806.1	71744	84739	-	10312
novel.3202	NW_016535805.1	93143	93459	+	267
novel.3209	NW_016535830.1	24069	28028	-	840
novel.3208	NW_016535828.1	711249	713962	-	284
novel.8214	NW_016586301.1	4751	5417	-	640
novel.8853	NW_016607924.1	344	20042	+	10012
novel.5578	NW_016545896.1	20011	20387	+	356
novel.5989	NW_016548802.1	10735	18805	+	474
novel.2008	NW_016532054.1	2382	2960	-	347
novel.2009	NW_016532055.1	174242	174885	-	538
novel.2558	NW_016533726.1	61006	62517	-	1512
novel.2559	NW_016533727.1	32319	32829	-	455
novel.2552	NW_016533720.1	10608	11056	-	306
novel.2001	NW_016532032.1	51922	52322	+	268
novel.2002	NW_016532032.1	51917	52340	-	292
novel.2003	NW_016532035.1	337777	338530	+	698
novel.8218	NW_016586392.1	1023	1851	+	772
novel.2005	NW_016532037.1	16140	86420	+	1859
novel.2006	NW_016532039.1	310462	314268	+	2785
novel.2007	NW_016532050.1	186769	187046	+	257
novel.8616	NW_016602979.1	1	1978	+	1978
novel.5985	NW_016548764.1	55787	64579	-	2708
novel.8852	NW_016607907.1	5370	13491	+	694

novel.6012	NW_016548949.1	243422	248646	-	395
novel.6011	NW_016548948.1	222102	222552	+	278
novel.6118	NW_016549619.1	92504	114242	-	278
novel.4899	NW_016542142.1	16352	16610	-	202
novel.4898	NW_016542129.1	11992	17931	-	1893
novel.4891	NW_016542096.1	46980	47310	+	285
novel.4890	NW_016542094.1	329582	329934	-	259
novel.4893	NW_016542107.1	52277	58178	+	341
novel.4892	NW_016542100.1	158144	158749	+	400
novel.4895	NW_016542124.1	50761	51272	+	487
novel.4894	NW_016542118.1	19771	51416	+	905
novel.4897	NW_016542129.1	11992	14692	+	2701
novel.4896	NW_016542124.1	98379	110060	+	841
novel.6016	NW_016548961.1	33520	34368	+	849
novel.1119	NW_016529778.1	10760	11338	+	363
novel.1118	NW_016529777.1	10712	35490	-	1817
novel.1113	NW_016529772.1	145852	149374	-	3467
novel.1112	NW_016529772.1	351766	352342	+	577
novel.1111	NW_016529772.1	342940	343559	+	620
novel.1110	NW_016529772.1	75508	76562	+	1029
novel.1117	NW_016529772.1	355134	356154	-	957
novel.1116	NW_016529772.1	351306	351665	-	316
novel.1115	NW_016529772.1	342940	343559	-	620
novel.1114	NW_016529772.1	223469	223916	-	379
novel.4408	NW_016540216.1	351408	351950	+	509
novel.4409	NW_016540216.1	393470	394505	+	990
novel.4406	NW_016540216.1	146905	151772	+	2629
novel.4407	NW_016540216.1	322191	339045	+	452
novel.4404	NW_016540197.1	232982	236766	-	3615
novel.4405	NW_016540212.1	92789	97820	+	4935
novel.4402	NW_016540197.1	221205	221704	-	444
novel.4403	NW_016540197.1	229222	239058	-	255
novel.4400	NW_016540195.1	58519	69771	-	269
novel.4401	NW_016540197.1	70606	72702	-	2097
novel.6668	NW_016554315.1	64168	65396	+	295
novel.6669	NW_016554315.1	78890	80009	+	1120
novel.6660	NW_016554222.1	24919	25891	+	973
novel.6661	NW_016554239.1	34080	34848	-	430
novel.3789	NW_016537779.1	3566	4591	-	997
novel.6663	NW_016554245.1	10386	10676	-	236
novel.6664	NW_016554278.1	801	2328	-	1528
novel.6665	NW_016554298.1	36921	37657	-	685
novel.6666	NW_016554298.1	75796	79066	-	738
novel.6667	NW_016554298.1	98141	98590	-	395

novel.7718	NW_016570306.1	316	585	+	233
novel.519	NW_016528404.1	1	666	+	666
novel.518	NW_016528403.1	5197	5597	+	362
novel.517	NW_016528400.1	76746	101063	+	1244
novel.516	NW_016528399.1	300596	301836	+	252
novel.515	NW_016528392.1	28149	34536	-	2220
novel.514	NW_016528392.1	440149	440915	+	712
novel.513	NW_016528392.1	34829	37472	+	2644
novel.512	NW_016528389.1	210338	211315	-	939
novel.511	NW_016528386.1	228633	232119	+	234
novel.510	NW_016528384.1	4967	5485	-	397
novel.8006	NW_016578879.1	10895	11715	-	821
novel.6019	NW_016548999.1	32626	33132	-	390
novel.8004	NW_016578802.1	30164	31227	+	757
novel.8005	NW_016578879.1	7627	8824	+	1155
novel.7339	NW_016563025.1	5098	56985	+	1071
novel.8002	NW_016578708.1	1	4218	+	4119
novel.8003	NW_016578758.1	655	2910	+	2256
novel.6018	NW_016548984.1	45339	46453	+	1062
novel.8001	NW_016578707.1	4123	4923	-	769
novel.2871	NW_016534675.1	162379	164669	+	1194
novel.2870	NW_016534672.1	455	1850	-	1286
novel.2873	NW_016534675.1	442457	442893	-	409
novel.2872	NW_016534675.1	163527	164669	-	772
novel.2875	NW_016534690.1	3494	8465	+	995
novel.2874	NW_016534687.1	375114	393845	-	373
novel.2877	NW_016534698.1	26386	27512	-	1127
novel.2876	NW_016534692.1	114394	114807	+	376
novel.6930	NW_016556926.1	32049	47875	-	6508
novel.3656	NW_016537382.1	47886	48437	+	471
novel.6932	NW_016556951.1	113236	124662	+	395
novel.6933	NW_016556963.1	15338	20322	+	2468
novel.6934	NW_016556963.1	21264	21529	+	266
novel.6935	NW_016556963.1	15195	15886	-	461
novel.6936	NW_016556980.1	96372	96993	+	538
novel.6937	NW_016556981.1	39234	42024	+	2215
novel.6938	NW_016556981.1	60679	62626	+	1022
novel.6939	NW_016556981.1	262243	264036	+	1743
novel.5313	NW_016544306.1	2159	2629	+	471
novel.5312	NW_016544303.1	31737	48976	+	4881
novel.5315	NW_016544309.1	11240	13762	+	382
novel.3654	NW_016537373.1	167	654	-	488
novel.5317	NW_016544309.1	11240	11653	-	364
novel.5316	NW_016544309.1	34285	35124	+	840

novel.5917	NW_016548213.1	150616	159296	+	1310
novel.5916	NW_016548212.1	4361	5059	-	670
novel.5915	NW_016548201.1	8358	15658	-	1052
novel.5914	NW_016548195.1	77518	78053	-	350
novel.5913	NW_016548195.1	106468	108656	+	2092
novel.5912	NW_016548189.1	360377	360787	-	249
novel.5911	NW_016548189.1	358276	359852	-	1196
novel.5910	NW_016548189.1	337746	350839	-	609
novel.3652	NW_016537368.1	95462	95747	+	286
novel.5919	NW_016548227.1	70257	76280	+	3271
novel.5918	NW_016548213.1	24863	25367	-	351
novel.8576	NW_016601573.1	216	2087	-	1872
novel.8577	NW_016601742.1	1	1323	+	1323
novel.8574	NW_016601513.1	6	2074	+	1251
novel.8575	NW_016601559.1	1	1886	-	1412
novel.8572	NW_016601481.1	1	882	+	396
novel.8573	NW_016601511.1	561	2073	+	582
novel.8570	NW_016601373.1	111	2039	+	285
novel.8571	NW_016601454.1	935	1354	-	259
novel.7229	NW_016561053.1	4288	5042	-	511
novel.7228	NW_016561046.1	15778	17006	-	1009
novel.8578	NW_016601774.1	1	2141	-	335
novel.8579	NW_016601836.1	1	1784	+	745
novel.8626	NW_016603243.1	1	2156	+	1630
novel.8627	NW_016603276.1	491	2291	+	314
novel.8624	NW_016603164.1	1844	2612	+	769
novel.8625	NW_016603201.1	2004	2627	+	348
novel.8622	NW_016603159.1	1	2611	+	2611
novel.8623	NW_016603159.1	10	2611	-	2602
novel.2945	NW_016534957.1	12418	12666	-	249
novel.2944	NW_016534957.1	62699	63246	+	499
novel.2947	NW_016534958.1	124117	137202	-	1538
novel.2946	NW_016534957.1	12795	13109	-	258
novel.2941	NW_016534955.1	20510	21479	+	817
novel.8620	NW_016603153.1	1	2608	-	2608
novel.2943	NW_016534957.1	62076	62537	+	462
novel.2942	NW_016534957.1	28944	65905	+	2374
novel.8621	NW_016603154.1	1	2608	-	2154
novel.2949	NW_016534971.1	304164	306031	+	1868
novel.2948	NW_016534971.1	291205	291839	+	517
novel.6066	NW_016549342.1	73955	74691	+	436
novel.6067	NW_016549354.1	112427	113474	-	583
novel.6064	NW_016549327.1	69874	71017	+	1089
novel.6065	NW_016549327.1	175089	175357	+	217

novel.6062	NW_016549290.1	36344	37663	-	1298
novel.6063	NW_016549305.1	1430	5197	+	331
novel.6060	NW_016549290.1	28795	29434	-	394
novel.6061	NW_016549290.1	33617	36072	-	2399
novel.6068	NW_016549369.1	124975	125778	-	804
novel.6069	NW_016549384.1	232634	275704	+	784
novel.2499	NW_016533552.1	399189	399483	-	295
novel.2498	NW_016533550.1	98688	129105	-	248
novel.4061	NW_016538748.1	43802	44368	-	207
novel.2493	NW_016533537.1	62279	63118	+	261
novel.2492	NW_016533530.1	73705	74959	+	1213
novel.2491	NW_016533529.1	1121787	1122096	+	255
novel.2490	NW_016533528.1	33807	40358	-	280
novel.2497	NW_016533550.1	170965	171289	+	270
novel.2496	NW_016533548.1	95861	96110	-	250
novel.2495	NW_016533539.1	32762	36189	+	325
novel.2494	NW_016533537.1	110035	112785	-	1822
novel.4798	NW_016541753.1	98856	100412	-	420
novel.4799	NW_016541756.1	5730	22654	+	246
novel.4796	NW_016541746.1	151767	152010	-	244
novel.4797	NW_016541750.1	7761	8380	-	236
novel.4794	NW_016541743.1	15452	16301	+	762
novel.4795	NW_016541746.1	149922	151123	-	1202
novel.4792	NW_016541737.1	34864	35547	+	652
novel.4793	NW_016541737.1	39709	40033	-	268
novel.4790	NW_016541726.1	10918	12874	+	1957
novel.4791	NW_016541726.1	7930	9622	-	724
novel.6329	NW_016551040.1	13061	13520	-	403
novel.6328	NW_016551040.1	11299	11548	+	217
novel.4414	NW_016540273.1	36987	37543	-	352
novel.6325	NW_016551018.1	3856	8502	-	218
novel.6324	NW_016551000.1	8087	8327	+	219
novel.6327	NW_016551040.1	11219	13268	+	293
novel.6326	NW_016551019.1	5213	6016	+	761
novel.6321	NW_016550975.1	1	322	-	322
novel.6320	NW_016550959.1	26109	27089	-	717
novel.6323	NW_016550993.1	77305	77561	+	233
novel.6322	NW_016550983.1	21177	21527	+	351
novel.5204	NW_016543648.1	14369	16091	-	568
novel.4412	NW_016540261.1	19244	19542	-	264
novel.6497	NW_016552669.1	8487	8904	-	245
novel.6496	NW_016552669.1	7999	8276	-	229
novel.6495	NW_016552669.1	4398	5324	-	688
novel.6494	NW_016552669.1	7490	7751	+	240

novel.6493	NW_016552669.1	4398	17409	+	2563
novel.6492	NW_016552651.1	3287	3899	-	613
novel.6491	NW_016552649.1	2186	6592	-	4407
novel.6490	NW_016552647.1	5580	5856	+	225
novel.6499	NW_016552678.1	370	777	+	316
novel.6498	NW_016552669.1	9260	10696	-	1380
novel.6674	NW_016554342.1	22135	36358	+	2059
novel.1562	NW_016530768.1	509886	511453	+	1535
novel.1563	NW_016530768.1	575419	575700	+	242
novel.1560	NW_016530768.1	506572	507217	+	616
novel.1561	NW_016530768.1	507961	509395	+	1394
novel.1566	NW_016530780.1	466397	466603	-	207
novel.1567	NW_016530788.1	73100	73562	+	408
novel.1564	NW_016530776.1	12822	18096	-	209
novel.1565	NW_016530777.1	68100	69439	-	471
novel.1568	NW_016530792.1	122116	122495	-	380
novel.1569	NW_016530794.1	70148	70763	+	271
novel.5672	NW_016546417.1	196125	198432	-	2252
novel.5673	NW_016546417.1	201992	202614	-	516
novel.5670	NW_016546417.1	194306	195930	+	1550
novel.5671	NW_016546417.1	196125	198432	+	2260
novel.5676	NW_016546449.1	267930	269805	+	1876
novel.5677	NW_016546449.1	267930	269805	-	1876
novel.5674	NW_016546438.1	12930	19658	-	377
novel.5675	NW_016546449.1	48558	50793	+	2040
novel.5678	NW_016546464.1	28200	41071	+	234
novel.5679	NW_016546473.1	1	3530	-	3449
novel.7818	NW_016573027.1	1	11091	-	276
novel.7819	NW_016573107.1	187	966	-	753
novel.7814	NW_016572938.1	1318	1707	-	358
novel.7815	NW_016572994.1	2232	2469	-	200
novel.7816	NW_016573001.1	350	659	+	267
novel.7817	NW_016573024.1	434	1118	+	645
novel.7810	NW_016572891.1	10315	10604	+	234
novel.7811	NW_016572891.1	7803	10125	-	2276
novel.7812	NW_016572893.1	2962	3337	-	236
novel.7813	NW_016572938.1	1259	1883	+	604
novel.1306	NW_016530165.1	78566	104449	+	214
novel.1307	NW_016530165.1	124697	125292	-	596
novel.1304	NW_016530164.1	20225	22958	+	722
novel.1305	NW_016530164.1	94239	119364	+	2216
novel.1302	NW_016530159.1	174865	175467	-	433
novel.1303	NW_016530163.1	136784	137410	+	627
novel.1300	NW_016530154.1	283504	298163	+	370

novel.1301	NW_016530159.1	3115	3813	+	303
novel.1308	NW_016530168.1	178921	270733	+	386
novel.1309	NW_016530168.1	165021	203037	-	210
novel.728	NW_016528859.1	88021	90695	+	1298
novel.729	NW_016528859.1	83118	88550	-	848
novel.720	NW_016528846.1	639848	674222	-	636
novel.721	NW_016528850.1	116228	116530	+	262
novel.722	NW_016528854.1	190245	207269	+	941
novel.723	NW_016528854.1	241224	244266	+	3043
novel.724	NW_016528854.1	70615	74332	-	3492
novel.725	NW_016528857.1	100332	107010	+	1098
novel.726	NW_016528859.1	46001	79888	+	613
novel.727	NW_016528859.1	63189	64317	+	383

gene_chr: The chromosome number of the new gene;

gene_start: The starting coordinates of a new gene on a chromosome;

gene_end: The terminal coordinates of a new gene on a chromosome;

gene_strand: Information on the positive and negative strands of a new gene on a chromosome;

gene_length: The length of new gene.

Supplementary table 2. DEGs between embryo-hair and adult-hair (embryo-hair vs adult-hair group)

Gene ID	log2FoldChange (embryo-hair vs adult-hair group)	padj	Gene name
108403753	27.54062924	5.29E-09	LOC108403753
108401670	25.18662056	1.49E-07	MYOG
108403755	25.17170744	1.51E-07	BPIFA1
108396706	13.49563006	9.98E-07	LOC108396706
novel.2691	13.10020009	1.91E-54	-
108386349	11.54344902	1.46E-12	DLK1
novel.6667	11.2831497	4.63E-39	-
108402994	10.2689854	0.000325	KRT4
novel.959	10.19190886	0.023908	-
108404578	10.17775676	0.048918	SMYD1
novel.3231	10.07444795	2.24E-12	-
108407212	9.756417969	0.030954	LOC108407212
108386766	9.388726104	7.41E-15	ALOX15
108410178	9.378527461	0.024508	RTL1
novel.7997	9.125561912	1.65E-07	-
108389267	9.078484438	1.14E-07	LOC108389267
108398407	8.901507456	1.19E-09	PEG10
108404657	8.698919164	2.07E-06	FEZF2
108386239	8.662831094	3.08E-17	OGN
novel.730	8.602709874	4.15E-06	-
108397708	8.493153618	1.26E-08	LOC108397708
108384371	8.482780901	5.06E-07	FIGF
108398405	8.378039098	9.52E-19	COL1A2
108385117	8.23145632	1.04E-07	TMEM92
novel.7996	8.215816721	2.61E-05	-
108388477	8.136937947	2.27E-05	LOC108388477
novel.7383	8.110198477	0.000138	-
novel.2921	8.109315319	0.000154	-
108392530	8.0968247	3.04E-15	COL11A1
108388713	8.094604528	9.30E-14	LOC108388713
108390042	8.077727423	1.27E-05	TTC6
108394651	8.048708676	1.96E-05	CARTPT
novel.7560	7.995605082	0.000381	-
108395905	7.917781959	0.000173	LOC108395905
108387740	7.917409336	0.000323	BHMT
novel.1920	7.896316771	7.30E-05	-
108396363	7.877183855	0.001	ATP1B4
108392838	7.827386725	0.000923	BRINP3
novel.1410	7.814357654	0.000166	-
108405369	7.803169258	0.000166	NEFM

108410258	7.801607608	8.32E-05	HBM
novel.3585	7.790795518	5.85E-10	-
108407689	7.787942071	2.76E-19	LOC108407689
108390991	7.723879438	0.000672	LOC108390991
108385587	7.647130331	0.000266	CLDN10
novel.8750	7.607101642	0.000206	-
108410180	7.590243553	2.39E-06	LOC108410180
108401111	7.585044326	3.08E-05	LOC108401111
108410003	7.576572641	0.001672	SLC4A1
108396540	7.571230204	0.023598	LOC108396540
novel.5386	7.571230204	0.023598	-
108396998	7.567923157	7.41E-06	OC90
108393609	7.544165728	9.76E-39	CLEC3B
novel.823	7.535950979	0.00087	-
novel.7163	7.532277923	0.009677	-
108406962	7.531346605	2.41E-13	ADAMTS19
108383849	7.479971813	0.00362	GDF3
108390412	7.479340061	0.000742	LOC108390412
108391037	7.471227269	2.36E-06	CASQ2
novel.4480	7.449338146	0.030239	-
108383801	7.399644338	0.001039	LOC108383801
novel.4802	7.396867471	0.033146	-
novel.1755	7.360795837	0.035261	-
novel.3853	7.356501346	0.035277	-
108392706	7.343056657	0.001819	LRFN2
108383750	7.324796997	5.80E-08	MEST
novel.5134	7.32043375	0.037843	-
novel.4554	7.304938995	0.039227	-
108392822	7.302056061	0.039214	LOC108392822
novel.3586	7.282315262	2.57E-05	-
108390537	7.280186356	0.00249	ISLR2
108400121	7.267279945	0.00017	CRMP1
novel.438	7.266462357	0.042008	-
108400617	7.244583715	0.010092	SLC6A3
novel.1465	7.22975259	0.000518	-
108385124	7.210867404	3.08E-17	COL1A1
108401433	7.205401246	5.22E-09	LOC108401433
108399390	7.190108402	0.009241	MAP3K7CL
108404602	7.165772641	0.000532	COL26A1
108401786	7.156786973	0.005855	ACTL9
108409895	7.143837305	0.023622	LOC108409895
108386709	7.1203422	0.004801	LOC108386709
novel.1489	7.106800328	0.00062	-
108409012	7.105173195	0.004972	OPALIN

novel.1235	7.100130649	0.019887	-
108390713	7.090966898	2.74E-22	NREP
108409018	7.04730959	0.008967	LOC108409018
108392652	7.016195948	1.93E-19	C1QTNF6
108385603	6.999981452	1.90E-06	LOC108385603
108405427	6.992352008	2.95E-07	TNNT2
108395289	6.975229632	0.038586	LOC108395289
novel.5056	6.958494513	0.001864	-
108399132	6.941175739	1.63E-12	LOC108399132
108409668	6.914342294	0.001437	LOC108409668
novel.6475	6.890838997	0.011557	-
108390209	6.876876011	0.027861	CDH12
108395566	6.872044182	0.000233	LOC108395566
108408911	6.841794242	1.87E-05	KRT24
novel.4717	6.836833035	0.034797	-
108402149	6.825962094	0.015764	ACAN
108392465	6.825715417	0.00015	HEPACAM2
108409350	6.821675735	0.005811	GRIA2
novel.4314	6.808509357	0.017688	-
108402535	6.806629429	0.023917	LOC108402535
108394997	6.793360424	0.015734	LOC108394997
108385916	6.7560134	0.002585	LOC108385916
108385699	6.74586049	0.020665	SYT2
novel.2703	6.744017034	0.011552	-
novel.1907	6.737959703	0.032966	-
108399559	6.726221695	0.002129	BRINP1
108386454	6.711374856	0.020387	LOC108386454
novel.6774	6.699800416	4.21E-08	-
108388181	6.693229052	0.034472	LOC108388181
108400922	6.692426484	0.037065	LOC108400922
108403769	6.686263286	4.09E-13	LOC108403769
novel.2668	6.68533785	0.004308	-
108384614	6.676556331	0.000468	KCNE5
108404660	6.668500273	0.000548	CADPS
108393325	6.653982087	3.75E-07	LOC108393325
novel.2221	6.631268778	0.005076	-
novel.495	6.616955181	0.027751	-
108386742	6.602168678	0.032994	LOC108386742
108393073	6.584339775	0.031553	LOC108393073
108407916	6.574395264	0.040403	HGFAC
novel.6318	6.565353368	0.000312	-
108388687	6.561802534	1.00E-12	SSC5D
108392167	6.548640091	1.59E-08	KCP
novel.2463	6.483628991	0.019345	-

novel.4999	6.478559726	0.009571	-
108388961	6.475005568	0.002514	LOC108388961
novel.2590	6.472050785	0.04344	-
108396089	6.406617456	2.86E-06	MYL4
108387760	6.397944059	0.048452	TLX3
108391665	6.380744343	4.68E-08	KERA
108409732	6.375765765	0.001469	CPNE4
108388448	6.36449368	4.03E-06	LOC108388448
108394841	6.333507966	8.86E-16	CD248
108384919	6.333316974	0.000259	LOC108384919
novel.3793	6.331058247	2.94E-18	-
108392716	6.330448773	7.68E-07	LOC108392716
novel.6931	6.328689743	0.015007	-
108404601	6.322484743	0.011117	DPPA3
108408481	6.292756488	4.98E-06	LOC108408481
novel.4964	6.288464399	0.033037	-
108401215	6.283200065	0.001144	ASGR1
108393085	6.260664473	2.25E-13	LOC108393085
108384932	6.249680684	3.08E-17	MATN4
108398658	6.246719132	0.012663	DBX2
108383795	6.170451034	0.00023	LOC108383795
novel.8041	6.165278837	0.000292	-
108394133	6.149958827	1.97E-06	TAGLN3
novel.839	6.142598571	0.018637	-
novel.3588	6.131384088	4.03E-07	-
108406631	6.129697462	2.01E-14	ATP8B3
108406972	6.120190225	0.000565	KCNMB2
novel.6193	6.11641928	6.90E-05	-
108388291	6.099542542	0.019679	BEND6
108397059	6.0950595	0.003132	EYA4
novel.3716	6.077157598	0.023053	-
108390545	6.033232988	2.52E-16	STRA6
novel.3140	5.98094209	0.026147	-
108396886	5.968735291	5.17E-08	MFAP5
108401437	5.962665532	0.034962	LOC108401437
108390632	5.937511377	1.87E-11	SFRP2
108407694	5.910529232	0.038377	LOC108407694
108389133	5.893610059	0.002678	LOC108389133
108410181	5.881477565	2.18E-05	LOC108410181
novel.4684	5.878322942	9.86E-05	-
novel.203	5.877414794	0.038703	-
108391666	5.869635735	1.34E-06	LUM
108395645	5.868955341	1.02E-10	LOC108395645
108403616	5.864806029	5.33E-11	SEMA6C

108406557	5.864169377	0.000586	HOXD3
108394038	5.845442499	4.74E-21	MMP16
108402966	5.839593929	2.17E-05	SOAT2
108408157	5.829709813	0.00048	APCDD1L
108386235	5.80303845	3.75E-08	ASPN
108395931	5.790685864	0.013709	LOC108395931
novel.455	5.78933575	0.034723	-
108386504	5.783733122	0.009548	NECAB2
108404021	5.777690916	7.34E-13	LRRC17
novel.6608	5.754083992	0.002643	-
108391073	5.735825495	1.20E-06	HOXB5
108400448	5.7156118	0.023423	LOC108400448
108398348	5.713745331	0.034246	IGSF5
108389827	5.680823265	0.015923	ZIM3
108409951	5.669492048	0.017929	ISL2
108407690	5.650368573	3.86E-12	LOC108407690
108384780	5.648814434	0.02912	ATCAY
108404394	5.648806194	0.006712	FAM131B
108395230	5.645684085	0.001242	GCGR
108386217	5.634389544	0.009423	SLC26A7
108402613	5.62379204	8.07E-07	LOC108402613
novel.5933	5.612092903	0.018761	-
108409567	5.607259874	2.90E-09	KAZALD1
novel.1431	5.60613727	0.000164	-
novel.6700	5.597665749	0.024255	-
108393474	5.584250232	0.0012	SKIDA1
108396562	5.584205941	0.000581	KIAA1024L
novel.2903	5.569871886	0.003003	-
108396711	5.5607167	0.002077	LMO3
108397922	5.557899109	8.56E-05	LRRC4B
108408525	5.542804458	7.74E-08	HOXC6
108394385	5.532793762	0.001349	APELA
novel.7633	5.525847415	0.003642	-
108384386	5.520846226	9.54E-05	LRFN5
108388795	5.513334693	0.00188	1-Mar
108403546	5.505947677	6.32E-08	GPR162
108398527	5.489133155	0.001153	LOC108398527
novel.2430	5.48571232	0.00757	-
108401773	5.479754216	0.000635	CNTNAP4
108384716	5.473589946	0.019283	CUNH19orf18
108390087	5.471252335	0.013889	KIF12
novel.5891	5.464568749	0.012826	-
novel.8074	5.461362834	0.006911	-
108397414	5.456197829	1.31E-06	CRABP1

108394368	5.45003158	6.01E-06	FREM1
108407896	5.449465527	0.000945	LOC108407896
108409441	5.430981304	7.22E-06	TMEFF2
novel.1008	5.424695349	0.035726	-
108399215	5.402758088	0.000838	LOC108399215
novel.2407	5.39107825	0.014512	-
108391734	5.388115307	6.08E-05	EPHA7
108391814	5.386397339	1.46E-07	SERPINF1
108407765	5.38413249	0.012663	FADS3
108389301	5.377796263	5.12E-06	LOC108389301
108386778	5.372948264	1.87E-06	GAS2
108398381	5.364395429	0.022052	TMEM8C
novel.2277	5.351181215	0.046666	-
108390795	5.340200754	0.008265	HMGCS2
novel.804	5.323672542	0.006629	-
108383276	5.30631807	1.98E-13	CRYM
108403402	5.298170042	4.17E-05	KCNQ4
novel.2042	5.29803262	0.015949	-
108400134	5.289339543	1.61E-05	LSAMP
108395218	5.288118027	8.80E-06	STAB2
108408139	5.278420858	0.007178	ATP2B2
108389911	5.27478203	3.40E-05	LOC108389911
108403263	5.273892938	0.000154	LOC108403263
108405505	5.269153798	0.005286	SERPINC1
108390604	5.268775465	0.000219	CDHR3
novel.3935	5.264440236	0.010677	-
108404448	5.250791296	0.012955	PPP1R3A
novel.2056	5.245302548	0.030706	-
novel.1364	5.24357194	0.036054	-
108398807	5.231666226	6.60E-05	NCAM2
novel.7946	5.205613339	0.023155	-
108383478	5.185224014	0.01554	PNCK
108393600	5.183195675	0.000287	CCL24
108408661	5.180692873	0.014011	LRRC66
108397792	5.156539128	1.02E-10	FGF10
novel.3554	5.141869895	0.002628	-
108387509	5.137203457	0.000271	LOC108387509
108390824	5.133876866	1.20E-08	POSTN
108383432	5.127491364	0.000207	ERN2
novel.7747	5.122400167	0.014114	-
novel.1432	5.116876581	0.00642	-
novel.3333	5.108082011	3.63E-05	-
novel.3693	5.10323085	0.002239	-
108393541	5.095780714	5.58E-05	MAGEL2

108393888	5.094058603	5.29E-11	MFAP2
108409493	5.090952052	2.88E-08	ADAM33
108386610	5.083300292	3.37E-12	CTHRC1
108409494	5.073605912	1.68E-14	LOC108409494
108410107	5.065970192	0.000405	LOC108410107
108399331	5.064052522	0.00102	KCNJ4
novel.2313	5.059724415	0.034208	-
108393919	5.059182902	0.028889	LOC108393919
108393189	5.058698476	0.043242	LGI3
108392835	5.04656611	1.35E-07	LOC108392835
novel.1142	5.039571383	0.032133	-
novel.3570	5.036274228	6.60E-06	-
108396670	5.031895999	4.23E-06	LRRC3B
108400788	5.028168293	0.000149	TMEM200A
108390243	5.022320974	0.032133	LOC108390243
novel.7398	5.015421486	0.035622	-
108399423	5.00468502	7.41E-06	SLC29A4
108409925	4.999973154	5.11E-08	FMOD
108387933	4.996895823	0.00102	CES1
108395675	4.99180029	1.82E-10	IGDCC4
novel.3146	4.985958587	0.03228	-
108405279	4.97408257	0.000166	UPK2
108390635	4.967011319	0.014614	FITM1
108408917	4.959873655	0.003236	OPRK1
108399290	4.95292176	0.010626	LOC108399290
108398432	4.947380039	7.09E-08	FGFR4
novel.7759	4.944601003	0.02747	-
108383948	4.942908577	0.008253	LOC108383948
108396530	4.932760845	0.000394	SLC7A3
108405398	4.929383425	4.64E-07	LOC108405398
novel.2461	4.927829215	0.001222	-
108388472	4.921853452	0.007177	LOC108388472
108407920	4.920612675	8.10E-16	OSR2
108387460	4.90656125	3.77E-10	AURKC
108399804	4.906031019	2.40E-05	GSC
novel.5788	4.900790636	0.049049	-
108398388	4.900687776	2.88E-08	ELN
novel.6024	4.880912782	0.0251	-
108403906	4.876912243	0.011956	LOC108403906
108399786	4.87200347	5.58E-05	LOC108399786
108391737	4.858316296	0.008055	C2CD4C
novel.2121	4.849115536	4.08E-05	-
108396486	4.848389723	3.66E-07	PTH1R
novel.1921	4.843828761	0.000791	-

108402158	4.82777867	4.24E-08	NTRK3
novel.3038	4.821616108	0.016525	-
108402965	4.818675406	6.10E-08	KRT18
108402608	4.809447263	0.000527	LOC108402608
108401920	4.798465372	0.036969	LOC108401920
108398707	4.797890905	0.00063	GRTP1
108397661	4.797125481	0.000235	LUZP2
108387414	4.789586751	0.040525	LOC108387414
108393438	4.78465609	0.000261	LOC108393438
108392548	4.764203702	4.69E-05	SPEG
108383481	4.758259959	2.73E-10	BGN
108405845	4.743028114	8.29E-05	NETO2
novel.2462	4.726905348	0.000219	-
108394122	4.707523261	0.002099	TMEM35A
108389883	4.694762312	4.54E-18	PI16
108402093	4.677640546	0.047202	GCNT3
108397868	4.677378453	1.20E-06	ARMCX4
108396666	4.661476682	0.025212	LOC108396666
108385897	4.637516021	0.000156	SVOP
108407292	4.63511676	1.46E-07	THBS3
108406128	4.631735571	1.65E-07	DACT3
108409383	4.62380119	1.62E-11	PCOLCE
novel.3657	4.623634869	0.01592	-
108387754	4.62207343	4.05E-05	NNAT
108397135	4.618015076	3.75E-07	LOC108397135
108400903	4.617996136	0.02107	ABCC6
novel.1429	4.607887185	0.003506	-
108402997	4.607336629	2.36E-09	COL5A1
108393953	4.602847577	1.31E-05	HPGDS
108401977	4.602362713	0.003036	LOC108401977
108406987	4.600817773	0.048297	NTN3
108404837	4.595528118	0.032986	HIPK4
108389962	4.595271766	0.001233	PTCH2
108403961	4.58721491	0.002175	FIGN
novel.2289	4.585353644	0.004821	-
108403912	4.583646819	0.00015	CAPN6
108390581	4.56183579	0.002129	DMBT1
novel.1945	4.550703744	0.02838	-
108398003	4.545988166	1.09E-05	ADAMTS14
novel.8667	4.544347524	0.019254	-
108400582	4.544123421	0.013889	LOC108400582
108393802	4.54005135	0.005553	AGER
108385348	4.526330116	1.32E-05	KCNA6
108395405	4.514463825	0.001335	PRAP1

108409116	4.510565222	4.68E-15	MFAP4
novel.3169	4.505939176	0.000895	-
108403634	4.504798433	0.030785	NXPH1
108389884	4.497477079	0.023877	LOC108389884
108407905	4.494365796	7.86E-06	SP5
108392913	4.489304334	0.000281	TNNC2
108404865	4.484824907	0.000128	USH1C
108401199	4.48140884	3.45E-08	ITGA11
108390599	4.479944824	0.000365	NOX4
108386237	4.472704171	9.51E-06	ECM2
108409684	4.470647497	0.000587	KRBA2
108401669	4.461580668	0.008212	ADORA1
108393307	4.450330697	0.015923	DUPD1
108399242	4.450216824	0.007301	PRND
108398629	4.447007211	0.031553	UBXN10
novel.5551	4.443436489	0.023571	-
novel.1141	4.441672857	0.001436	-
108389467	4.43428736	0.000302	LOC108389467
novel.283	4.434160796	0.003478	-
108398341	4.433129553	0.003195	CLDN3
108397095	4.431231096	0.000112	SPOCD1
108397591	4.407289767	1.42E-06	GFRA2
108384713	4.403539738	0.007814	DCHS2
108409300	4.401418758	0.001994	LOC108409300
108399338	4.398441418	0.0289	LOC108399338
108388230	4.393772022	0.020385	LOC108388230
108392701	4.390977172	0.008727	APOBEC2
108394280	4.385967456	4.67E-05	GNG4
108386505	4.384537363	6.79E-05	SLC38A8
108397366	4.381615793	2.67E-06	MGAT3
108400037	4.373376188	4.16E-05	ADCYAP1R1
108383937	4.371579015	0.001331	BRINP2
108406706	4.371339447	0.000728	TMEM178A
108384224	4.370360181	0.038042	LOC108384224
108391076	4.366956053	0.002286	HOXB6
108386056	4.364981556	2.71E-06	LOC108386056
108396364	4.363833656	7.43E-05	MAB21L1
108401245	4.363033341	0.000591	LOC108401245
novel.3675	4.3594991	0.042747	-
108408408	4.357898105	0.03032	HFM1
108389715	4.348752907	0.000429	LOC108389715
108399720	4.3396107	0.027721	LOC108399720
108383721	4.338335869	0.000109	CHRND
novel.536	4.333938594	6.22E-06	-

108385959	4.328107108	0.008078	LOC108385959
novel.3519	4.327096279	0.002465	-
novel.1562	4.323693244	0.025065	-
novel.636	4.322034305	0.004462	-
novel.1747	4.320997951	0.014614	-
108403559	4.320861624	0.042526	LOC108403559
108396737	4.310320428	3.40E-05	LOC108396737
108389850	4.294592193	0.000123	LOC108389850
108406007	4.288827751	0.002175	TMEM59L
108395367	4.284596397	0.038623	CHRNA1
108395684	4.282992531	0.014978	PTCHD1
108406475	4.276937379	1.74E-06	CPXM1
108395462	4.27514623	1.50E-05	ZNF469
108396960	4.272570379	0.001127	FAM92B
novel.6213	4.267380373	0.039227	-
108410030	4.267044303	3.87E-06	LOC108410030
novel.7486	4.263225152	0.012826	-
108399424	4.26306491	1.10E-06	DPT
108388519	4.257329938	1.71E-05	SRPX
108386921	4.252049801	3.69E-05	DTX1
108409793	4.250281631	3.29E-05	APLNR
108403516	4.249226743	9.26E-05	LOC108403516
108395234	4.246898577	6.87E-05	PPP1R27
108402371	4.243422053	0.002935	SLC12A8
108407873	4.239780424	0.031808	ACR
108392597	4.230710391	6.48E-12	FNDC1
108395435	4.230469813	9.00E-05	LOC108395435
108391656	4.228565127	0.000203	WDR93
108405396	4.218901249	2.95E-07	TBX5
108385601	4.199994431	0.003642	LOC108385601
108383723	4.19345909	0.026738	CHRNG
novel.4613	4.190495805	0.003195	-
novel.4241	4.183816679	0.022933	-
108406530	4.177109243	0.00515	LOC108406530
108390539	4.173141197	6.48E-10	ISLR
108396979	4.167574254	0.038302	SYT16
108409610	4.162880367	1.41E-12	FBN3
108389729	4.160970791	0.004561	LOC108389729
108399163	4.15969804	9.50E-06	COL9A3
108392268	4.158445108	1.95E-10	COL12A1
108392441	4.153431064	1.38E-05	LOC108392441
108400438	4.148297919	0.000467	LOC108400438
novel.4446	4.137494248	0.044494	-
108392711	4.134445921	0.046173	GRID2

108399525	4.134285533	0.006406	GCG
108393881	4.132100782	7.85E-06	SMTNL2
novel.6738	4.128260969	0.032489	-
108395368	4.12350186	0.001625	LOC108395368
108389163	4.118720605	0.000464	LOC108389163
novel.5200	4.11269043	0.038966	-
108394497	4.108283367	3.99E-06	CADM3
108391657	4.10754277	2.78E-06	ANPEP
108398045	4.104899275	0.006756	AMPD1
108395433	4.098251282	1.69E-05	COL21A1
novel.1957	4.096735625	0.043072	-
108408944	4.095034502	0.035019	LOC108408944
108410222	4.093943219	0.016288	LOC108410222
novel.8350	4.090351735	0.035206	-
novel.5804	4.089135422	0.041243	-
108395105	4.086195678	0.000348	NTF3
108400017	4.081263969	3.69E-10	SLC22A17
novel.164	4.066449228	0.006698	-
novel.2268	4.063446907	0.034852	-
108398271	4.055423627	0.00112	KRT7
108390536	4.054314231	1.16E-08	LOXL1
novel.4998	4.053021671	0.005076	-
108393437	4.052975158	0.005798	LOC108393437
novel.6358	4.050959678	0.000421	-
108389717	4.047063004	4.97E-05	LOC108389717
novel.2252	4.042976638	0.015829	-
108403551	4.042465367	0.003807	GNB3
108394435	4.041915065	0.032133	HMCN2
108385768	4.035582038	0.0321	RBFOX3
novel.7080	4.030945077	0.020007	-
108385788	4.026638118	6.18E-05	LOC108385788
108399563	4.026135822	0.025217	LOC108399563
novel.8203	4.024542094	2.53E-06	-
108408356	4.018612649	0.016275	NPPC
108395008	4.014452569	6.66E-08	FN3K
108391309	4.005488091	8.54E-05	VASH2
108404388	4.002096545	0.028294	CLCN1
novel.8067	4.001597302	0.035668	-
108406469	4.001068036	0.00171	SRL
108388324	4.000349757	0.000509	LOC108388324
108389213	3.999369295	0.004843	LOC108389213
108405167	3.993702403	0.043881	VWA3A
108399083	3.991304849	5.04E-05	TMCC2
108386554	3.990276932	9.36E-05	SDK2

108402724	3.987342183	0.000408	RXRG
108390980	3.981249498	0.041961	LOC108390980
108406806	3.980546963	1.40E-05	MDK
108386675	3.97941832	0.02912	SMCO3
108385558	3.978655897	0.022549	LOC108385558
108388076	3.975497796	0.046402	TRIM66
108383732	3.964976138	0.012131	MYBPC3
108383525	3.963045498	1.46E-10	ALDH1A3
108407367	3.952648227	0.037086	ILDR1
108402395	3.951224761	0.000941	MYOM1
108407388	3.942355723	2.13E-05	ADAM12
108389834	3.940801243	1.67E-09	CERCAM
108395029	3.930517319	1.32E-05	PLD5
108405718	3.928545904	9.23E-06	ADGRA2
108395572	3.926540858	0.001962	KIAA1324
108392070	3.926352684	5.47E-05	ADAMTSL5
novel.4067	3.913201431	0.032133	-
108394177	3.906909891	4.26E-06	RSPO3
novel.928	3.904944644	0.017743	-
108386099	3.903115907	2.89E-05	RHBDL1
108399225	3.899625262	0.000582	XPNPEP2
novel.5133	3.898412402	0.047299	-
108393998	3.897028718	0.000661	LOC108393998
108410108	3.893689599	0.023674	CCDC180
108398587	3.892896571	0.034723	BPIFB6
108393404	3.885136259	0.00151	USP35
108407007	3.881067956	0.02621	FGF14
108403954	3.878296211	8.16E-09	MYOM2
108383469	3.877793604	0.00042	GPIHBP1
novel.4351	3.874649676	1.87E-06	-
novel.522	3.874050061	0.004995	-
novel.2982	3.871603303	0.00362	-
108406808	3.861375088	8.48E-08	CREB3L1
novel.8786	3.859942876	0.009785	-
108391956	3.852658426	0.024978	TSPYL5
108396034	3.844030154	0.025103	LOC108396034
108408970	3.842306632	0.000395	MUC4
108389061	3.839309146	0.022677	LOC108389061
108394197	3.837582339	0.000509	PCDH18
108394015	3.836802135	0.00877	PCP4
novel.7166	3.833191846	0.018697	-
108402992	3.826336182	0.002628	KRT8
108387585	3.823090215	0.003955	BAIAP3
108399607	3.820675682	0.032886	LOC108399607

108383279	3.815198855	0.01433	LOC108383279
108386630	3.810703076	0.002029	NAPEPLD
novel.1233	3.808169371	0.008673	-
108402571	3.805010595	0.001273	GYG2
108402092	3.798558243	0.024835	KCNB2
108408427	3.796302683	8.80E-07	CUNH14orf132
novel.4712	3.788867269	0.03371	-
108407357	3.788755089	5.84E-05	SLC15A2
108400043	3.787957377	0.00023	MAP1A
108385615	3.786762367	0.012827	PABPN1L
108396496	3.779139028	0.000173	NACAD
108403512	3.776909289	0.000468	ROBO2
108401818	3.776642784	0.006883	FNDC5
108401942	3.773851142	0.012131	LOC108401942
108388530	3.760127213	0.0018	SEC16B
108387217	3.758881314	2.03E-07	FSTL1
108397070	3.756005292	3.15E-05	SLC4A3
novel.3408	3.755742149	0.022321	-
108404668	3.738522424	0.043072	LRRN3
novel.7637	3.73842985	0.018545	-
108394943	3.732551381	0.027727	LOC108394943
108389525	3.731290862	0.008572	LOC108389525
108402838	3.731208962	0.000869	RHPN1
novel.5174	3.718222356	0.002657	-
108408683	3.713542123	0.000975	CDKN1C
108396379	3.708692393	0.000219	GPHA2
108395532	3.708439044	0.008117	PLPPR3
108397929	3.708402362	4.51E-05	CLEC11A
108398174	3.706603264	2.24E-08	MMP23B
108408788	3.705796733	0.000634	PIEZO2
108399171	3.691546676	6.87E-05	SLC17A9
108405883	3.689800522	0.00131	LOC108405883
108404109	3.687611312	0.006602	LOC108404109
novel.4396	3.686729069	0.001588	-
novel.8128	3.686707549	0.026013	-
novel.3179	3.683523554	0.039343	-
novel.3063	3.675357639	0.015336	-
108389696	3.673438755	0.001787	CDHR4
108383872	3.673347499	0.018583	LOC108383872
108407470	3.672906591	0.031561	AVPR2
108408269	3.664958948	0.030904	SEZ6
108397652	3.664521142	0.001986	COL24A1
novel.8199	3.66200854	0.011144	-
108393693	3.659603686	0.041473	LOC108393693

108406835	3.656168285	0.007949	FUT7
108394104	3.655850307	9.05E-06	COL14A1
108407924	3.650292096	9.78E-08	PKDCC
108385120	3.648245733	4.19E-07	SAMD14
novel.3700	3.641877977	7.33E-06	-
novel.5641	3.637143208	0.019887	-
108405873	3.63228164	0.000645	DCLK3
novel.2581	3.630517085	0.002349	-
108404006	3.629773394	0.026225	MSANTD1
novel.8089	3.625401182	0.008526	-
108389942	3.624452072	0.022823	ALAS2
108386753	3.624038931	0.000806	CCDC8
108405049	3.621583908	0.032953	COL13A1
108387546	3.619962073	0.008967	PIK3R6
108397558	3.603387752	0.009941	CA14
108397175	3.602958134	0.019288	MPO
108387985	3.598466942	0.046258	CACNA2D3
novel.918	3.5980918	0.012601	-
novel.7954	3.596788349	0.011097	-
108387168	3.591913166	0.002261	LOC108387168
108408125	3.590049638	0.048163	LOC108408125
108402816	3.588725647	0.001055	OLFML2B
108387541	3.587874333	1.24E-05	CPE
24019677	3.58524853	0.012498	ND4L
108383412	3.584661304	0.001972	PCDH9
108400595	3.583161095	0.029258	LOC108400595
108384375	3.58313812	0.046895	ART3
108407264	3.575787078	0.02599	MUC1
108396548	3.57498471	0.020493	BMPER
108385774	3.574232893	0.007216	LOC108385774
108386667	3.573167436	0.003388	GRIN3A
108408527	3.572637958	0.00923	HOXC5
novel.4658	3.561094001	0.033297	-
108410116	3.556201178	0.002572	MPPED2
108404966	3.554610454	0.039842	WDR86
108395911	3.552934916	0.00094	RSPO1
108399396	3.549549012	0.022003	LOC108399396
108395853	3.544548151	0.034586	GDF10
108386077	3.540620235	0.000524	WNK4
novel.2893	3.54049256	0.023853	-
novel.7935	3.540206599	0.005897	-
108396555	3.538869617	1.21E-06	LOC108396555
108402852	3.538770189	2.39E-07	SEMA3B
108400436	3.534042273	0.000173	SOX12

108407788	3.53148004	0.0456	CUNH17orf99
108383303	3.530352356	2.28E-05	NES
108403904	3.529975876	0.00196	HHIP
108394754	3.515967915	0.046228	ALX4
108390600	3.514967051	0.025813	GRIA3
novel.5176	3.514948431	0.040038	-
novel.2820	3.513191836	0.027413	-
novel.1549	3.51116345	7.66E-05	-
108384141	3.503350785	0.001777	SPAG17
108395878	3.501148537	0.007315	LOC108395878
novel.3047	3.498203397	0.013439	-
108385605	3.497003571	0.000821	CBFA2T3
novel.7595	3.496934549	0.042218	-
108384774	3.489754856	0.000137	ZFR2
novel.3866	3.487290165	0.032133	-
108383696	3.484285601	4.67E-05	CACNB1
108402439	3.482118637	0.030058	FGF16
108393806	3.479529559	2.69E-05	ZNF536
108409816	3.477688374	0.00216	CHGA
108405911	3.477171676	0.00014	DMRT2
108394734	3.471057072	0.005206	AFAP1L1
novel.2323	3.468401785	4.80E-06	-
108409121	3.464091623	0.000327	LOC108409121
108402776	3.463103508	0.044905	CACNA1G
108407698	3.457257253	0.003693	CUNH2orf40
108402373	3.456070464	0.000154	FRAS1
108395489	3.45604522	0.005395	COL5A3
24019678	3.445762186	0.013402	ND4
novel.5494	3.444104078	1.95E-08	-
108394147	3.436161872	0.026793	POPDC2
novel.8181	3.435630946	0.031823	-
108397098	3.434228169	0.043072	LOC108397098
108401399	3.428527823	0.042534	RIMS2
108393114	3.425340218	0.002129	ANGPTL1
108394476	3.423435594	0.023788	EFHC1
108387521	3.422154502	0.004718	LOC108387521
108400983	3.419918229	0.008159	MYO15A
108390293	3.418429688	0.000493	LOX
108406904	3.41384077	0.000895	RASL11B
108409890	3.411913757	0.000611	LOC108409890
108396072	3.408638235	0.003162	GPC3
108399067	3.406478008	0.032623	OGDHL
novel.3069	3.405138535	0.002051	-
108392880	3.400523498	7.65E-06	CCDC80

108385283	3.399407979	0.032133	LOC108385283
108392649	3.396319454	9.58E-06	BAIAP2L2
novel.3036	3.393110035	0.028422	-
108384156	3.387767154	0.000436	BEGAIN
108406547	3.38550983	0.004364	HOXD4
108410148	3.384235788	3.52E-06	LOC108410148
108397281	3.381774968	0.003642	ABCC11
108387258	3.378113822	0.024299	LOC108387258
108387846	3.37746938	0.00018	LOC108387846
108388629	3.376654943	0.002219	LOC108388629
108405378	3.375513238	0.000206	LOC108405378
108393345	3.374728761	0.001526	BSN
novel.2388	3.374149962	0.019277	-
108406478	3.3734225	0.005327	CLDN7
novel.2739	3.37093506	0.01041	-
108408542	3.367912687	0.005265	LOC108408542
novel.2443	3.367742616	2.89E-05	-
108392677	3.359999963	3.11E-05	LGALS1
novel.2075	3.359153141	1.01E-05	-
108400615	3.356563338	0.00113	NRIP2
108395040	3.356250416	7.76E-07	ADAMTS2
108394259	3.353845687	4.56E-05	PLCB4
108399577	3.351970668	2.03E-07	FKBP10
108404025	3.350828666	9.51E-05	ACKR4
108406849	3.349788777	7.87E-05	VCAN
novel.4493	3.347858857	0.01285	-
108392687	3.347396752	0.032133	SOX10
108399878	3.346680028	0.005248	ACACB
108407441	3.346600441	0.001233	TMEM121
108398530	3.3459898	2.42E-05	LOC108398530
108399643	3.341370554	0.041441	CACNA1A
108388090	3.340938214	0.006146	LOC108388090
108396615	3.337393644	0.014084	ENTPD8
108403914	3.335303291	0.02107	IGSF23
108408293	3.331471391	0.046225	CCDC81
novel.1731	3.329010129	0.018163	-
108409490	3.326870474	0.001467	HSPA12B
108390474	3.323886371	0.007318	LOC108390474
108402023	3.320389982	0.000117	NOVA1
108409212	3.315653561	0.047059	NTNG2
novel.5087	3.311742543	0.028749	-
108406324	3.31018739	0.006201	MYH7B
108401830	3.309637217	0.00037	SFRP4
108391872	3.308859601	0.000822	NCALD

108400326	3.306607394	8.11E-07	RCN3
108383785	3.306256079	0.007315	RASA4B
108397863	3.305080402	1.17E-05	DDAH2
108385987	3.304117648	0.040897	LOC108385987
108394005	3.301952329	0.000808	LOC108394005
108394379	3.301945317	0.003765	SCG5
108395474	3.301938353	0.002777	LOC108395474
novel.5640	3.296774124	0.031541	-
108408230	3.291364381	0.026016	MYT1
108388123	3.289070934	0.001124	CD34
novel.706	3.284760445	0.003003	-
108392160	3.283854247	1.98E-06	GXYLT2
108394945	3.282827539	0.00015	CST6
108396489	3.28236605	0.01364	LOC108396489
108387572	3.281129121	0.003984	LOC108387572
108393221	3.279869679	0.000149	DTL
novel.5521	3.277500027	0.011417	-
108402586	3.277253633	0.010375	LOC108402586
108406152	3.277008161	0.002678	CAPRIN2
108403081	3.275690003	0.001598	CARD11
108401792	3.275496026	0.00123	ADAMTS10
108393212	3.273101136	0.000197	LOXL2
108400434	3.271945888	3.31E-05	CUNH20orf96
108392837	3.262620544	0.03371	ADAMTS15
108388174	3.262227466	0.004832	LOC108388174
108407625	3.260583424	0.011857	TPM2
108383997	3.254805382	0.003207	RGS22
novel.3126	3.246858457	0.000473	-
108384787	3.246016617	6.41E-05	HTRA3
108409995	3.242542637	0.001877	FBXL22
novel.3245	3.239578012	0.043341	-
108401393	3.235378924	0.002461	LOC108401393
108400708	3.235314504	0.004831	LOC108400708
108385581	3.234920983	0.001757	SPACA6
novel.7256	3.23389491	0.029044	-
108384429	3.232724912	0.013439	DTX4
108405124	3.230964274	0.000645	PRICKLE1
novel.1029	3.230418942	0.015224	-
108397913	3.230086201	0.000266	LOC108397913
novel.6804	3.227179454	0.002555	-
108384473	3.224651983	0.02107	STARD9
novel.2622	3.223237383	0.010566	-
108407520	3.222663421	0.013845	LOC108407520
108390540	3.218789845	0.007481	LOC108390540

108393295	3.215061696	0.001386	CDH7
108391653	3.207642472	0.011417	MESP2
novel.8169	3.206595148	0.019672	-
108388732	3.205961406	0.034404	AVIL
108403667	3.203163393	2.95E-07	FZD2
novel.6288	3.201562896	0.01243	-
108390152	3.199136931	0.013799	SPHKAP
108401872	3.193968494	0.01694	LOC108401872
novel.2902	3.193011207	0.004129	-
novel.1670	3.19240483	0.04146	-
novel.5994	3.189357699	0.007469	-
108409792	3.18453402	0.001242	SLC43A1
108394772	3.183439027	0.028919	NPFF
108385258	3.182164541	0.000524	ZNF835
108398385	3.177158325	0.041623	ADAMTS13
108402127	3.176634889	0.004129	HTR3A
novel.6933	3.175656518	0.002461	-
108384076	3.169355667	0.006249	AFF3
108386039	3.163950404	0.013885	PLXND1
108387691	3.163412736	0.002372	ZNF500
108387577	3.159037514	0.008943	ATP10A
108408851	3.154951677	0.016897	LOC108408851
108401674	3.154824202	0.004129	LOC108401674
novel.1521	3.153567395	0.009286	-
108392941	3.148296769	5.89E-05	SEMA3D
108394407	3.141998996	0.036909	LOC108394407
108386340	3.140338004	0.039227	LOC108386340
108384235	3.137578424	0.001875	DCHS1
108394106	3.136986352	0.000813	LRRN2
108390607	3.133827355	0.022569	LOC108390607
novel.3949	3.133231092	0.028502	-
108392108	3.13132058	2.69E-05	RADIL
108409109	3.127757344	0.030175	RTN1
108401982	3.12650224	0.001819	TRIM45
108402431	3.124431034	0.015923	LOC108402431
24019680	3.123841976	0.024517	ND6
108402878	3.122956816	0.000334	CUNH3orf18
108385485	3.122357154	0.000591	FRMD5
novel.8085	3.121407715	0.022024	-
108385243	3.115708889	0.018499	NRCAM
108393272	3.115242245	0.001903	TBXA2R
108384685	3.114784489	0.000511	DNM1
novel.135	3.113113264	0.001919	-
108399915	3.108814135	0.032634	GPR39

novel.658	3.106749588	0.002549	-
108383324	3.105080131	0.01498	PEAR1
108395606	3.102508964	2.52E-05	LOC108395606
novel.5733	3.10210448	0.035831	-
108387956	3.101455954	0.019389	LOC108387956
108407408	3.099278961	0.002157	LOC108407408
108395981	3.098208575	0.005592	MAST2
108391906	3.096510679	0.000407	NR2F1
108397452	3.094495612	0.039635	ATP6V1B1
108389482	3.089399758	0.000169	ARMCX6
108407243	3.087853103	7.41E-06	TWIST2
108388271	3.087077229	0.001511	ARHGEF17
108404289	3.085448214	0.013799	LOC108404289
108400234	3.084960609	0.001526	LOC108400234
108393146	3.084944488	0.00018	CARNS1
novel.6530	3.084858518	0.021361	-
108403632	3.076819505	0.005119	LOC108403632
108388461	3.069180846	6.25E-06	ANGPTL2
novel.5834	3.065288132	0.020051	-
108404936	3.064167027	0.014224	LOC108404936
108385922	3.062193835	0.023205	LOC108385922
108398950	3.059509629	0.012072	FXYD7
novel.8342	3.059170633	0.047663	-
108396554	3.055150317	0.009785	DNM3
108405368	3.054816418	0.012513	ESPL1
108402687	3.054764393	0.033624	ZBED8
108391384	3.05352419	0.002376	PTPRS
108405949	3.052086385	0.038223	LOC108405949
108407906	3.050774103	0.043604	MYO3B
108395772	3.048452139	0.011105	LOC108395772
108398021	3.044250287	0.028889	FAM19A5
108406900	3.039240221	1.90E-06	PLAT
108398326	3.038481359	0.0163	VPS37D
108400830	3.03829365	0.013072	CILP
108391081	3.030542852	0.009898	HOXB4
novel.4050	3.030205635	0.000409	-
108389537	3.026478953	0.009898	LOC108389537
novel.7400	3.025075143	0.01554	-
novel.263	3.023463054	0.041288	-
108400518	3.011491556	0.034929	LOC108400518
108406673	3.006272487	9.18E-05	COL6A6
108408468	3.006265323	0.033053	KCNH3
novel.7675	3.005471901	0.02154	-
108393428	3.002194169	0.048452	LECT1

108386108	3.000565338	0.004268	WDR90
108410310	2.998022819	7.45E-06	PDZRN3
108407486	2.997872232	0.004247	NDNF
108390749	2.997870188	0.001829	VASH1
108399919	2.996329514	0.000126	SORCS2
108397091	2.990257701	0.000421	COL16A1
novel.2658	2.989876548	0.020985	-
108402381	2.989524125	0.000468	LGR5
novel.6141	2.988687	0.006484	-
108388011	2.988463852	0.007736	MAP1S
108402365	2.983782711	0.00131	NEIL2
108394722	2.982263087	0.002409	CDH11
24019679	2.981421769	0.042008	ND5
108398417	2.980954823	0.027698	DMGDH
108392073	2.978509373	0.014821	PCSK4
108392119	2.978117492	0.009677	DND1
novel.7214	2.975736625	0.030175	-
108395215	2.971805116	0.003034	GLT8D2
108401325	2.968823833	0.046917	TDRD12
108396485	2.967186584	0.000467	RFTN2
108395169	2.966452178	0.008839	FRMPD3
108390342	2.962184987	0.004797	HSF4
108386456	2.961424403	0.006336	AGT
novel.77	2.961300358	0.027844	-
108386997	2.953648866	0.036801	SPATA32
108393143	2.953164754	0.00079	CPEB1
108409756	2.95038094	0.026761	PRRT2
108409754	2.949064848	0.016808	MVP
108386776	2.945683794	0.000272	LOXL4
108385509	2.940773588	0.011557	NAP1L3
108409560	2.940394277	0.000707	COL6A1
108386514	2.938048045	0.00193	ADGRD1
108393431	2.93722453	0.027413	LOC108393431
novel.4879	2.93319833	0.019013	-
108389940	2.928049212	0.001835	BOC
108408407	2.924981753	0.009774	LOC108408407
108406052	2.923573239	0.007704	COL28A1
108398837	2.916733135	0.011652	RGN
108404683	2.908754213	0.000508	PTGFR
108394940	2.906055724	0.0163	CELF3
108403705	2.902893535	0.02371	WDR97
108383267	2.9005041	0.004606	CRISPLD1
108389716	2.897277413	0.001672	LOC108389716
108407058	2.893253029	0.009368	FN1

108397006	2.893125723	0.025699	LOC108397006
108401425	2.892713783	0.014614	LOC108401425
108401004	2.891756247	0.018211	ARHGEF9
108403823	2.891735852	4.68E-05	OSR1
108384717	2.890257068	0.002531	ZNF606
108403594	2.888044657	0.004001	TLE2
108406821	2.879569502	0.007736	ABCA2
novel.3066	2.874229047	0.021893	-
108402983	2.87216449	0.033214	TNS2
108403547	2.868580052	1.02E-05	P3H3
108404359	2.868508201	3.69E-05	GSTA4
novel.4844	2.864900023	0.023423	-
108397951	2.864337652	0.005928	CCDC141
108390122	2.863842411	0.001039	OLFML3
108383243	2.863162223	0.008778	AMT
108390983	2.862915367	0.031633	TENM3
108400601	2.86277746	0.00951	LOC108400601
108410202	2.855449183	0.006517	NFATC4
108398721	2.854172167	0.00356	ZNF182
108404478	2.853207103	0.016072	SRGAP1
108410002	2.84728917	9.50E-06	FAM171A2
108402584	2.844538712	0.003693	ARHGEF16
novel.5503	2.84384589	0.038391	-
108390645	2.842567369	0.007159	TSHZ3
108392445	2.840813777	0.017497	TRPV4
24019681	2.839168669	0.022915	CYTB
108384164	2.835209389	0.000115	VSTM2A
novel.5218	2.833083804	0.006255	-
108406499	2.830905834	0.008079	NLGN2
108396951	2.829664805	0.0026	RNF212
108398346	2.828338073	0.002326	LIMK1
108387235	2.824647379	0.029531	MPP3
108390801	2.824412737	0.014443	LOC108390801
108409844	2.824315533	0.034256	LOC108409844
108383990	2.824020222	0.002424	S100A4
108388282	2.822921196	0.009265	WISP1
108394675	2.818576676	0.017929	LOC108394675
108387826	2.818292432	0.004308	CUNH22orf15
108397230	2.817527153	0.039964	NPR1
novel.6568	2.816583065	0.042543	-
108393792	2.816085831	0.000174	LOC108393792
108386869	2.815465563	0.031454	CUNH16orf96
108398220	2.81432771	0.000858	GRIP2
108403538	2.813979421	0.004026	PALM3

108406245	2.807905635	0.024786	ZFP69B
108394818	2.807122862	0.004268	KLHL13
108409492	2.806855452	0.049668	SIGLEC1
novel.2942	2.806596942	0.01772	-
108398244	2.800190426	0.045998	KCNK15
108402862	2.799180029	0.017497	LSMEM2
108393964	2.795519876	0.001651	ZC4H2
108399055	2.79524127	0.000266	ZNF671
108391158	2.795151003	0.013794	GPC6
108385938	2.793741363	0.008486	KIAA1549L
108402589	2.791268999	0.018761	LEFTY1
108386240	2.790587012	0.039564	LOC108386240
108384892	2.789604588	0.043072	LOC108384892
108397422	2.785870061	0.014742	ADAMTS7
108398930	2.781756421	0.046272	LOC108398930
108395365	2.781748721	0.016463	CHN1
108385482	2.760801477	0.025103	STX1B
108386940	2.760602496	0.007387	AHRR
108410042	2.760189834	0.007267	PXDN
108392368	2.757890043	0.016808	TRIL
108406707	2.75001473	0.006422	LOC108406707
108407787	2.749787581	0.008267	TK1
novel.3299	2.74863779	0.030126	-
108403319	2.744666018	0.00489	ZMYM3
108395048	2.740923869	0.00409	CUL7
108402654	2.734898796	0.008826	NME6
108388980	2.732612733	0.009423	LOC108388980
108402185	2.731058492	0.034586	LOC108402185
108400480	2.729447829	0.003928	TMEM132C
108404180	2.728933014	0.011796	PRRG3
108395551	2.728822846	2.07E-06	GALT
108386407	2.728041567	0.000272	SMTN
108400796	2.725177349	0.00015	EVA1B
108397351	2.71207088	0.016275	LRRC4C
108392832	2.711992249	0.014508	ADAMTS8
108409564	2.711091056	0.007537	COL6A2
108399921	2.710836884	0.024156	LRRN1
108405831	2.71024865	0.041751	LOC108405831
108391754	2.708000305	0.028614	LOC108391754
108409314	2.707208367	0.00204	THY1
108384636	2.706675378	0.004285	YPEL1
108401779	2.706597403	0.001452	DENND2A
108393179	2.706555019	0.001981	BMP1
108387844	2.7023121	3.11E-05	MRC2

novel.7978	2.701616907	0.036803	-
108396411	2.700034409	3.55E-05	OBSL1
108385864	2.699136212	0.000616	FAT4
108384676	2.698080724	0.026169	TNFRSF25
108397852	2.697231892	0.023553	LY6G6C
108384403	2.697212945	0.004395	CHAD
24019670	2.696120186	0.034833	ND2
108397090	2.696075224	0.03162	ADGRB2
108403553	2.694826049	0.047527	CDCA3
108385714	2.694230391	0.00791	LOC108385714
108390252	2.691603079	0.005076	ARHGEF40
108385771	2.691032184	0.011354	CEP131
108395933	2.690570361	0.037789	PMEL
108397299	2.687513005	0.011305	LOC108397299
108399061	2.686373511	0.012736	RWDD2A
108390470	2.684461343	0.000817	LOC108390470
108399260	2.683871625	0.037789	LOC108399260
novel.2911	2.683827748	0.021317	-
108397956	2.68352452	0.020294	FKBP7
108400672	2.683456054	0.008229	AEBP1
108397902	2.681569813	0.003506	ARMCX1
108406211	2.67094586	0.00675	ENPP6
108384385	2.670166005	0.001962	RIMS1
108387468	2.667676992	0.002293	SCARA5
108398704	2.663614343	0.038536	F7
108403657	2.661952975	0.043062	LOC108403657
108394817	2.661507074	0.000811	EML1
108404172	2.661343507	0.018423	MTMR11
108392545	2.660017798	0.000208	TMOD2
108389341	2.651937485	0.000335	SLC2A4RG
108404581	2.650506356	0.00923	LOC108404581
108393172	2.648653469	0.007097	RNF207
108409497	2.648588537	0.034211	SPEF1
108394778	2.643772372	0.047262	IL17B
108390341	2.643588338	0.005016	B3GNT9
novel.3154	2.643019489	0.00411	-
108399318	2.641721529	0.006478	IRX1
108390888	2.640852023	0.005911	LOC108390888
108389705	2.639758424	0.00114	FAM69B
108408523	2.632534235	0.000966	HOXC4
108395924	2.632366273	6.20E-05	LOC108395924
108396267	2.631887924	0.013448	LOC108396267
novel.8369	2.628538104	0.046416	-
108402441	2.62699049	0.008117	SPON2

108400221	2.626602362	0.044133	SLC4A11
108405186	2.626131511	0.014011	TTLL11
108409832	2.626097466	0.024333	ABCC5
108406639	2.622120228	0.001088	LOC108406639
108387234	2.619737149	0.017948	ETV4
108383794	2.617580274	4.73E-05	PRDM11
108394480	2.617232501	0.005213	LOC108394480
novel.145	2.616943543	0.019195	-
108408524	2.61237305	0.013439	HOXC9
108401694	2.609242506	0.012164	SH3GL3
108405137	2.606665683	0.033728	MAP2K6
108385092	2.604276487	0.00465	SERPINH1
novel.7055	2.601457666	0.025824	-
108400034	2.599991059	0.017124	CDH24
108385829	2.598399726	0.001327	GPX7
108383360	2.59740285	0.004715	CCDC65
108405877	2.597169108	0.013298	TRANK1
108390939	2.597072417	0.013799	PMEPA1
108383784	2.59316196	0.001012	LOC108383784
108391599	2.590272039	1.23E-05	TGFBR3
108404678	2.59025923	0.015764	WTIP
novel.1393	2.587708037	0.037576	-
108394386	2.587343934	0.004526	PLPP7
108385169	2.584300437	0.044494	PHLDB1
108388835	2.583839969	0.037065	LOC108388835
108407678	2.58063195	0.002326	BMP4
108408793	2.578762266	0.049538	GNAO1
108385825	2.578568471	0.032634	MYO1A
108410113	2.577215407	0.016666	AKAP6
novel.690	2.577164701	0.002678	-
108404419	2.57503787	0.008826	DNAH1
108403218	2.574205273	0.011953	PARVB
108386779	2.572961648	0.023487	EMP3
108390357	2.572674477	0.010581	ZDHHC1
108402511	2.569760225	0.01498	SGCG
108383994	2.568690197	0.004695	S100A6
novel.5595	2.56194376	0.048248	-
108398489	2.561448683	0.031541	STX1A
108392090	2.560017734	0.005788	SIX5
novel.33	2.559831402	0.001044	-
108383392	2.558196883	0.001209	KCNAB3
108387107	2.551526818	0.033426	TNFRSF13C
108399333	2.544036195	0.005247	KDELR3
108396733	2.540329605	0.000467	LOC108396733

108390517	2.539170598	0.010442	LOC108390517
108388785	2.538784889	0.010738	ABCA7
108409110	2.536991561	0.01429	RNF112
novel.548	2.525726091	0.044412	-
108386230	2.525691256	0.031522	LOC108386230
108402354	2.516344748	0.032743	CEP152
108392815	2.514678182	3.94E-05	PLSCR4
108398563	2.511360905	0.00185	MOXD1
108402892	2.50751713	0.049913	LOC108402892
108396082	2.505656298	0.035277	LOC108396082
108396649	2.505160729	0.001447	COPZ2
108402090	2.504194349	0.030818	PDE3A
108399742	2.50410541	0.015829	LOC108399742
108383898	2.502099192	0.030948	WHSC1
108385912	2.501846221	0.000996	CPNE5
108389888	2.501309625	0.042534	CAND2
108406785	2.501075714	0.019287	COL6A3
108406060	2.486774169	0.01647	CDK3
108395335	2.486578316	0.000445	FILIP1L
novel.7942	2.485206755	0.032013	-
108391822	2.485130106	0.003422	GALNT16
novel.6402	2.482280879	0.0012	-
108394239	2.481586206	0.002498	BEND5
108388709	2.480858405	0.009718	KMT5C
novel.79	2.479606943	0.02283	-
novel.8421	2.473234314	0.039353	-
108396026	2.472181474	0.032133	NID2
108385255	2.471425477	0.043072	LOC108385255
108406553	2.470255257	0.012313	HOXD9
108395567	2.470192291	0.033878	LOC108395567
108391809	2.467690414	0.03379	RTN4RL1
108383354	2.465138114	0.001552	PTOV1
108397343	2.463821976	0.014014	PTDSS2
108389570	2.463071675	0.005911	ZNF350
108398235	2.461624739	0.015923	LOC108398235
108405426	2.461587317	0.046416	LOC108405426
108388617	2.460905165	0.033348	LOC108388617
108391146	2.45801115	0.003003	PHF19
108390543	2.456634828	0.002141	PML
108391763	2.452935314	0.024242	LOC108391763
108392098	2.451364215	0.031541	CAB39L
108405786	2.450677261	0.007612	LOC108405786
108400415	2.447354652	0.032585	TXLNB
108393193	2.4431049	0.001794	CA11

novel.4913	2.435828198	0.00532	-
108399613	2.435796228	0.000371	BIN1
108408753	2.435629929	0.02599	SYDE1
novel.4861	2.434744909	0.006204	-
novel.8042	2.42909374	0.00585	-
108406641	2.429079939	0.045229	ABHD15
108394849	2.426901142	0.0123	CNIH2
novel.3187	2.426080276	0.030228	-
108399959	2.424299294	0.017367	DCUN1D2
108400207	2.422942428	0.012131	CUNH20orf194
108394346	2.420549097	0.010567	CNNM2
108407340	2.414884842	0.017227	TBX15
108402612	2.408929454	0.025183	IGF2
108383974	2.408664361	0.033962	FGF18
108395922	2.407790908	0.011011	MMP19
108390890	2.406893082	0.030647	LOC108390890
novel.1667	2.396100845	0.005247	-
108393184	2.387566893	0.015639	LOC108393184
108391501	2.38656089	0.012164	VEGFC
108400136	2.383145265	0.006415	GAP43
novel.3755	2.382388943	0.019009	-
108404432	2.381891096	0.02944	STAB1
108389680	2.37971131	0.01772	LOC108389680
108384051	2.379298743	0.01364	EHD2
108400293	2.376818475	0.000183	FCGRT
novel.3658	2.375956136	0.030319	-
108402490	2.375044404	0.010775	RASL10A
108394212	2.371848372	0.01269	PODXL2
108394465	2.36656624	0.019381	DEPDC5
novel.8706	2.364467901	0.000323	-
108399679	2.36291407	0.007267	LYL1
108400558	2.359246224	0.036117	CUX2
novel.3385	2.357562749	0.000277	-
novel.680	2.354336788	0.02914	-
108405770	2.351293049	0.002748	WNT11
108391806	2.349686353	0.006519	HIC1
108402355	2.349509264	0.015196	CACNA1D
108394752	2.348725572	0.002142	TP53I11
108400749	2.34583805	0.009932	INTS12
108393168	2.341679296	0.014977	GPR153
108384467	2.338101433	0.035668	LOC108384467
108395604	2.337862465	0.013495	LMF2
novel.8429	2.334963074	0.034083	-
108385313	2.334330584	0.022558	TPCN2

novel.4594	2.332221956	0.007313	-
108395463	2.32990282	0.040042	ZFPM1
108394751	2.320840066	0.034145	TSPAN18
novel.7411	2.320287813	0.020223	-
108397435	2.315469608	0.030656	LOC108397435
108402736	2.313776736	0.016349	MAGED1
108401817	2.312822869	0.034649	LOC108401817
108403618	2.310858768	0.031705	TMOD4
108407478	2.309847728	0.002579	CCDC102A
108399435	2.308901453	0.001777	GAMT
108402867	2.305757532	0.02599	ZMYND10
108405776	2.305172712	0.035394	LOC108405776
108409883	2.304196905	0.008004	SMO
108392625	2.300464997	0.0329	TMEM119
novel.1901	2.298378565	0.035277	-
108400335	2.297712346	0.030785	CCDC155
novel.1590	2.297488752	0.028721	-
108390354	2.291231438	0.04084	SLC9A5
108390740	2.289955763	0.046677	LAYN
108408098	2.283814537	0.023053	TRIM3
108407763	2.2831252	0.024406	FADS2
108400979	2.282845884	0.001895	ALX3
108388663	2.281448659	0.014036	MIIP
108396660	2.280928837	0.027496	DPYSL4
108407662	2.279342509	0.001585	PTP4A3
108407091	2.279028392	0.000421	MEIS1
108392389	2.278614408	0.035269	DAGLA
108396877	2.275133792	0.030175	RNF215
108391205	2.274595398	0.009898	EMILIN1
108392364	2.269060538	0.026016	GPX8
108409220	2.268013025	0.043062	CAPS
108393692	2.267869327	0.004666	PGBD1
108404417	2.263257584	0.004165	NT5DC2
108392089	2.26320788	0.015639	DMPK
108407122	2.2624463	0.011956	ROR2
108398953	2.25653559	0.023065	HPN
108389121	2.255262642	0.039458	LOC108389121
108389378	2.24369306	0.03094	LOC108389378
108395150	2.243046456	0.016003	FBLN5
108388471	2.240870104	0.000661	RAB30
108398976	2.239964135	0.005962	HIF3A
108401655	2.238853458	0.000438	BMF
108386659	2.237415395	0.037223	LOC108386659
108406185	2.236476124	0.048248	EDN3

108392765	2.236019009	0.002547	GGA2
108391340	2.234739339	0.007706	PCBP4
108408780	2.228761527	0.038391	LOC108408780
108404904	2.227903673	0.041651	CNTLN
108397909	2.227163124	0.026424	SLF1
108395238	2.226967375	0.043431	NPB
108398202	2.226873802	0.026169	SPC24
108401315	2.225397299	0.022265	CDKL1
108405095	2.224657444	0.044387	INPPL1
108384329	2.219100024	0.002157	LRP3
108404135	2.218857493	0.025568	EGFLAM
108385471	2.216008287	0.042862	OLFM2
108401243	2.215725479	0.037855	TDRP
108406538	2.210145528	0.040403	NPR2
108403482	2.207805686	0.008069	ZNF713
108393898	2.205296111	0.007538	ATP13A2
108385741	2.201791505	0.00085	PDGFRA
108387667	2.198668115	0.045532	OLFM1
108403498	2.193385944	0.019602	ZNF275
novel.6684	2.192315712	0.003824	-
108409683	2.191694601	0.041374	HES7
novel.4611	2.188805128	0.034484	-
108402873	2.186539228	0.034833	SLC38A3
108403374	2.186296048	0.036027	FRMD4A
108406102	2.185619415	0.007464	MMP15
108406777	2.184873444	0.000321	THBS2
108403394	2.183916619	0.014224	NDN
108391651	2.183626061	0.030255	KIF7
108388066	2.183476519	0.033284	HOXA3
108409982	2.18144263	0.009898	ARHGEF25
108393388	2.181302043	0.035157	GRIK5
108407047	2.179376778	0.031297	CKMT2
108386944	2.173967228	0.026555	TPPP
108387279	2.172802439	0.013076	LOC108387279
108401638	2.171604676	0.005013	RCN1
108384516	2.156468547	0.003506	ARHGAP24
108402981	2.156221367	0.009677	IGFBP6
108395637	2.155869131	0.026883	DTNA
108385483	2.154863637	0.023418	PYROXD2
108410203	2.149239895	0.049485	RIPK3
108395523	2.147397462	0.006629	PALM
108395046	2.145217934	0.000558	PTK7
108384413	2.144314092	0.002544	SMARCA1
108395319	2.141877332	0.018178	ZKSCAN2

108406638	2.139937957	0.012072	ANKRD13B
108398404	2.138345311	0.001276	SGCE
108407191	2.136653842	0.011417	IER5L
108386057	2.135068393	0.009567	PLEKHH3
108391390	2.134761916	0.020922	LOC108391390
108400264	2.133926931	0.010092	RGMB
108387410	2.129548685	0.003689	PRKAR1B
108396056	2.128885012	0.016666	RGS10
108395804	2.127482598	0.012453	NAT16
108386884	2.126447255	0.001565	RTN2
108385655	2.124320943	0.001934	LOC108385655
108395270	2.118127598	0.032013	CLCN2
108401071	2.116061231	0.037065	XRCC3
108400433	2.113876	0.003642	ZCCHC3
108410246	2.113011899	0.009056	SLC26A11
108400016	2.112671135	0.039343	CUNH14orf93
novel.995	2.10607575	0.034211	-
108395992	2.104661538	0.046172	SLC1A3
108389876	2.102030589	0.027727	LOC108389876
108400552	2.099159901	0.045532	HVCN1
108396805	2.097526467	0.032133	DAAM2
108397253	2.097426016	0.014862	FARP1
108405154	2.097376438	0.024201	LOC108405154
108400638	2.093999123	0.030175	MYO15B
108390408	2.093235308	0.004336	ABCG2
novel.7575	2.089592989	0.017532	-
108410266	2.089043474	0.041834	PPP1R26
108400284	2.073472803	0.021115	MAMSTR
108390074	2.070141998	0.032133	TGFB1I1
108410378	2.070007839	0.013214	P3H1
108396822	2.066638553	0.036593	INPP5A
108395073	2.064787093	0.031513	FRS3
108410271	2.06319637	0.040038	SIX4
108385217	2.060950343	0.011757	SEMA6A
108393959	2.057612181	0.049877	WNT2
108397436	2.055941453	0.034246	LOC108397436
108383414	2.055884437	0.048734	LOC108383414
108392463	2.052162515	0.03182	OSBPL6
108389587	2.050832811	0.04851	MLYCD
108385656	2.046915958	0.033053	LOC108385656
108395594	2.043844232	0.008159	CCL21
108388639	2.043594558	0.010222	ENTPD6
108403985	2.04328051	0.016003	CENPV
108397239	2.037677539	0.013441	SLC27A3

novel.3098	2.036419682	0.009253	-
108391745	2.036224026	0.047623	SHC2
novel.131	2.02942962	0.038586	-
108387247	2.017373725	0.0118	AP3B2
108397579	2.014967422	0.006272	RARRES2
108407859	2.01104532	0.001972	ADD3
108399569	2.004332637	0.001777	P3H4
108395012	2.001381273	0.009898	TBCD
108399164	1.998243611	0.025595	RBBP8NL
108403420	1.997393421	0.033739	RCBTB2
108395453	1.994856513	0.040863	FBXO17
108391716	1.992563078	0.01554	ANXA6
108386918	1.992561881	0.005169	CDC42EP5
108395778	1.992047347	0.047224	LOC108395778
108385854	1.986865798	0.037246	LOC108385854
108407850	1.982595835	0.039317	SH3BGR
108392905	1.980264046	0.030562	ZSWIM3
108402283	1.977146317	0.035207	RTKN
108394470	1.9735348	0.041832	PI15
108402792	1.973338311	0.011085	RHOBTB3
108387645	1.968775188	0.029193	TTLL10
108396354	1.967806151	0.02255	LOC108396354
108410074	1.966002308	0.032634	TSPAN7
108393092	1.964042856	0.038854	DUSP15
108401163	1.962860801	0.024299	LOC108401163
108391348	1.961059496	0.006858	ABHD14A
108400230	1.958236886	0.032743	TMEM263
novel.1192	1.956414347	0.030785	-
108397667	1.950039468	0.035019	KANK4
108400712	1.949419232	0.013086	FBXL7
108409757	1.94822381	0.022786	PAGR1
108398333	1.938813137	0.030319	SPATA2L
108407013	1.931463763	0.021115	BICD1
108397018	1.925226865	0.019598	CUNH11orf74
108401808	1.917303877	0.031823	ZNF362
108384232	1.911169299	0.04369	ILK
108387712	1.910740618	0.02944	ARHGAP20
108384416	1.905754251	0.002465	RBP1
108406471	1.899214795	0.043062	EBF4
108401135	1.897015219	0.045291	PRKCDBP
108387302	1.895866973	0.020956	DAPK1
108386221	1.894750155	0.018637	TTYH2
108390344	1.893948138	0.041441	FBXL8
108408272	1.889818937	0.019679	ORC1

108384072	1.887690119	0.004773	RAB34
108395796	1.878991218	0.003587	POFUT2
108386749	1.87671796	0.005076	ANKRD29
108398433	1.873683184	0.034723	ZNF346
108391752	1.869591942	0.014224	MAGEH1
108388677	1.859990839	0.034246	NAT14
108403789	1.858893366	0.018761	ABI2
108390326	1.858064202	0.009947	CMTM3
108402860	1.854565552	0.025212	HYAL3
108407399	1.854307226	0.028721	LOC108407399
108402759	1.848255725	0.014614	FXYD6
108404255	1.846188196	0.032013	RNF169
108383501	1.840965963	0.046869	CACNB3
108393575	1.8406467	0.018697	FAXC
108408612	1.839540153	0.026793	SLC12A7
108406642	1.838097106	0.024786	TP53I13
108401939	1.838053947	0.01933	IGFBP5
108408638	1.826896443	0.041832	CRACR2B
108396369	1.822939704	0.029153	NRXN2
108393370	1.817887397	0.030818	GLRB
108399660	1.817648144	0.044339	SYCE2
108394081	1.806725907	0.018272	TCEA2
108402629	1.798664885	0.040038	LTBP3
108395512	1.791977244	0.040622	ENHO
108409266	1.788439111	0.025065	IP6K2
108402690	1.786871685	0.03985	C1QTNF2
novel.1773	1.785970616	0.035697	-
108386412	1.784908822	0.043547	LOC108386412
108408096	1.783766656	0.030617	APBB1
novel.3851	1.781017465	0.04891	-
108404795	1.778215609	0.017936	ABLIM2
108409802	1.777305709	0.042008	LOC108409802
108385819	1.770937558	0.00891	NAB2
108396183	1.767357604	0.025358	GMPPA
108399213	1.76637332	0.009056	LOC108399213
108388626	1.75864891	0.03536	LOC108388626
108397882	1.757742108	0.027496	ATP6V1G2
108390819	1.749091062	0.043597	MYO7A
108409913	1.748950959	0.027721	SNRPN
novel.4146	1.745639783	0.025197	-
108389711	1.738743411	0.00891	LOC108389711
108385513	1.72861434	0.046013	CHST10
108393213	1.728265035	0.013889	R3HCC1
108409591	1.721444089	0.041374	SFXN3

108400632	1.718400997	0.023889	GAL3ST4
108402491	1.714668995	0.046917	LOC108402491
108384521	1.706156973	0.048163	CCDC167
108387837	1.705296194	0.025027	LOC108387837
108395994	1.69518297	0.018778	TMEM9
108395990	1.690314489	0.042526	DNPEP
108399800	1.688376649	0.020076	VDAC3
108406367	1.676927803	0.0412	MXD3
108391812	1.673998182	0.029901	PTPRD
108398952	1.652808087	0.049775	ZNF792
108393268	1.645823008	0.021227	TJP3
108383864	1.64096837	0.026777	LOC108383864
108384789	1.636598619	0.033154	TMSB10
108408985	1.625118053	0.01554	MZT2B
108397791	1.621981087	0.025065	LOC108397791
novel.4340	1.597521867	0.046228	-
108396407	1.593609496	0.037246	LOC108396407
108408439	1.568968384	0.032966	GLT8D1
108391043	1.554615981	0.026451	FAM117A
108408687	1.546143452	0.036397	MPDZ
108385650	1.526246799	0.038703	LOC108385650
108408904	1.524716483	0.03988	LOC108408904
108409864	1.513004519	0.025727	TCAIM
108389813	1.496289078	0.048476	ZBTB8A
108399386	1.478309341	0.036027	FMO4
108403239	1.477025756	0.045433	GOLPH3L
108387424	-1.319094082	0.046674	ABCE1
108404952	-1.418439706	0.044641	EFCAB14
108399574	-1.419476516	0.02838	KRT14
108397315	-1.424842301	0.031561	AGFG1
108402106	-1.427595852	0.04112	BNIP2
108391770	-1.435882724	0.041071	FCHO2
108396757	-1.436287904	0.049485	LOC108396757
108383828	-1.438063961	0.039716	PCYT1A
108401192	-1.441292181	0.028135	CANX
108398160	-1.445336559	0.046685	PICALM
108393756	-1.459626487	0.031795	SUSD6
108389795	-1.464212049	0.047865	LOC108389795
108392571	-1.473918327	0.038536	GPD2
108392982	-1.47527804	0.04505	BICD2
108397739	-1.478275323	0.045532	CUNH16orf72
108407928	-1.478582556	0.03275	GTF2A1
108402775	-1.503012617	0.03429	SPAG9
108396812	-1.503519219	0.034723	NUFIP2

novel.1418	-1.503588488	0.024508	-
108394898	-1.526586595	0.028131	ABCD4
108407730	-1.544836364	0.038623	PTBP3
108392849	-1.550441381	0.026467	LOC108392849
108397036	-1.553268219	0.030904	TRAFD1
108402820	-1.555942262	0.04052	LOC108402820
108395867	-1.556622633	0.042543	TCP11L1
108403663	-1.559760325	0.048245	ARNTL
108406501	-1.566929544	0.041202	KCTD11
novel.5586	-1.572488839	0.031055	-
108394203	-1.573499178	0.031797	CEBPG
108396460	-1.575508708	0.020836	LOC108396460
108396993	-1.579633993	0.027868	PNPLA8
108390925	-1.579992001	0.034833	SUDS3
108398478	-1.586046089	0.008673	UHMK1
108407907	-1.586513265	0.036787	SMNDC1
108396063	-1.586961527	0.036983	ARHGEF12
108385181	-1.590910969	0.017662	FAM8A1
108391606	-1.608231242	0.024034	SMC5
108392350	-1.608639288	0.025103	AFF4
108404707	-1.624047771	0.043062	IL1R1
108405136	-1.626074436	0.029666	ABCA5
108392317	-1.632812997	0.019602	TP63
108396005	-1.638402671	0.046175	SOCS4
108407422	-1.639287932	0.030904	SLC41A1
108406202	-1.641288899	0.019803	DENND1C
108402057	-1.641860385	0.015923	BAZ1A
108387061	-1.6504004	0.018326	HACE1
108385528	-1.659197884	0.046416	DCUN1D3
108385950	-1.659878808	0.041571	WRN
108400388	-1.66212592	0.020693	PIK3CB
108385742	-1.672026997	0.034649	CHIC2
novel.595	-1.673794937	0.048403	-
108389972	-1.680529986	0.009929	MCTP2
108407259	-1.681070087	0.028344	LNX2
108394689	-1.681773396	0.038505	SAMD4B
108409064	-1.686318885	0.026511	ATP8B1
108388787	-1.687444021	0.035207	GPX4
108400916	-1.689700679	0.033214	MAOB
108390457	-1.69102092	0.02595	NR1D2
108397387	-1.69103813	0.01403	APPL1
108404565	-1.706668139	0.016663	ZMPSTE24
108405624	-1.708547371	0.01157	PPP1R14C
108404911	-1.70911042	0.041202	CPOX

108396362	-1.710528585	0.027294	LOC108396362
108406974	-1.711359166	0.027727	ZMAT3
108409957	-1.715283177	0.019389	CCNL1
108386211	-1.719221272	0.02685	STX3
108391689	-1.72476103	0.042862	DNAJB14
108392750	-1.724903877	0.021184	UBE2H
108392322	-1.725013221	0.019381	BCL6
108395133	-1.725289799	0.041243	HK1
108384181	-1.731200466	0.021325	WASL
108397186	-1.731900885	0.010985	UGCG
108404267	-1.732284055	0.019377	EIF4A1
108394837	-1.73402375	0.018761	PSTPIP2
108399693	-1.740256973	0.022823	VAV3
108394108	-1.741047777	0.02224	HPSE
108407725	-1.74145615	0.043084	NIPAL4
108385984	-1.749817613	0.020086	LOC108385984
108386806	-1.751554119	0.011284	ATAD1
108407842	-1.752605631	0.025824	SNX11
108408568	-1.753769377	0.02136	EIF4E
108399243	-1.756020004	0.037065	SMOX
108403399	-1.7593531	0.023853	KLRG2
108390575	-1.76075114	0.044323	IKZF5
108384891	-1.763356682	0.00633	LOC108384891
108398513	-1.76378437	0.024303	TMEM165
108388208	-1.764228822	0.024044	ARMC12
108393848	-1.766214313	0.031541	KLF6
108404424	-1.769987699	0.009988	NDUFAF4
108397544	-1.770845263	0.037392	ARL8B
108387198	-1.771579335	0.030034	LOC108387198
108406396	-1.771679736	0.042006	CPEB4
108391706	-1.776939056	0.018697	NFKB1
108408424	-1.780956935	0.023609	SBNO1
108405705	-1.781511484	0.025212	PELI1
108394871	-1.781935992	0.048403	DSC1
108389465	-1.792882562	0.043062	ZRANB1
108398502	-1.804678869	0.029993	VMP1
108405690	-1.805085331	0.039873	OSTM1
108392022	-1.809202435	0.015777	ZBTB21
108409431	-1.809685593	0.003928	ABCA1
108404149	-1.811688873	0.045777	SHOC2
108387488	-1.817188856	0.021843	PARD6B
108392756	-1.820345939	0.016912	PQLC1
108400015	-1.826454468	0.023267	AJUBA
108396117	-1.827351206	0.031553	M6PR

108393396	-1.829072852	0.0165	ADAM10
108409482	-1.836434024	0.005404	ZBTB43
108398424	-1.840767124	0.004648	CPNE8
108408000	-1.841031949	0.049668	CSGALNACT2
108404704	-1.847061512	0.004483	SCNN1B
108393044	-1.854641215	0.046917	LOC108393044
108390973	-1.860096099	0.011462	ATP1A1
108407429	-1.860676445	0.018832	RAB29
108395157	-1.863529563	0.003436	DDX18
108385913	-1.865180945	0.047262	CDKN1A
108408937	-1.865417682	0.005121	SLC16A7
novel.2781	-1.869443416	0.014224	-
novel.3011	-1.869920705	0.028889	-
108386759	-1.872269284	0.044714	MOSPD2
108396994	-1.878382039	0.007537	EFR3A
108408056	-1.878409245	0.012381	TTPAL
108390163	-1.881514133	0.002639	MXD1
108399565	-1.887979649	0.00699	AVL9
108391105	-1.88892326	0.037112	FOXP2
108391396	-1.894281135	0.004268	TICAM1
108392797	-1.896572043	0.025262	CHD7
108397797	-1.900197607	0.006866	ADSS
108394160	-1.902916208	0.014443	UBE2D1
108384826	-1.906925541	0.042526	C1S
108389820	-1.907089285	0.04511	FAM76A
108406328	-1.909867628	0.037789	PROCR
108391001	-1.910038105	0.017532	UBL3
108391339	-1.919058929	0.049133	TIGAR
108386274	-1.920842823	0.006629	MFHAS1
108409078	-1.922734249	0.016881	SLC24A4
108390605	-1.923096957	0.002219	SYPL1
108408795	-1.930735888	0.00791	NUDT21
108396898	-1.933607287	0.003693	PLEKHA1
108398395	-1.93780218	0.034081	SLC2A6
108395941	-1.938669809	0.022549	E2F7
108395304	-1.941742634	0.036895	LOC108395304
108396380	-1.942083127	0.036739	BATF2
108386446	-1.943673103	0.031984	LOC108386446
108398317	-1.945278198	0.008035	ARAP2
108388818	-1.945633866	0.004641	SAR1B
108393458	-1.946327685	0.004972	LOC108393458
108407158	-1.948638194	0.010147	MAP7D1
108397984	-1.950648692	0.044766	STON2
108400048	-1.95296558	0.008773	CKMT1A

108408055	-1.95436682	0.048452	SERINC3
108405995	-1.956671597	0.036054	AK4
108385611	-1.957918677	0.034211	PRSS8
108409555	-1.959008444	0.006502	CACUL1
108402782	-1.963564777	0.04787	TOB1
108383611	-1.96467153	0.028721	ANKRD9
108403795	-1.970658724	0.024421	TRMO
novel.7601	-1.977477572	0.02247	-
108393946	-1.979114254	0.004488	IL6R
108410302	-1.980757872	0.045857	IL20RA
108404052	-1.987282687	0.001777	CPD
108403280	-1.989034269	0.018142	IGF1R
108408987	-1.994686924	0.043509	SNAP29
108387475	-1.99863805	0.043834	LOC108387475
108392617	-2.003431157	0.016808	RET
108397518	-2.007355361	0.013405	ABCG1
108400398	-2.009616128	0.020086	LOC108400398
108400010	-2.01062359	0.007972	LOC108400010
108393510	-2.013820571	0.03182	LOC108393510
108395742	-2.016618627	0.018853	CREBRF
108388158	-2.024379164	0.011861	IFRD1
108400883	-2.025247417	0.047224	LOC108400883
108391114	-2.028071679	0.034929	LMBRD2
108390191	-2.034511318	0.04181	THBS4
108398007	-2.036859064	0.012275	SGPL1
108405346	-2.043483797	0.01791	ANTXR2
108397144	-2.045401365	0.012281	ANKRD13C
108405299	-2.049141824	0.005617	NIP7
108401119	-2.054047346	0.001487	RAB27A
108393487	-2.060201358	0.025824	ADGRF2
108404217	-2.064678677	0.026375	AASS
108409986	-2.065493308	0.024508	OTUD6B
108402368	-2.065799107	0.000421	LOC108402368
108407915	-2.066683601	0.03332	LOC108407915
108407892	-2.070452451	0.046258	LOC108407892
108408666	-2.07381778	0.003308	PPP1R15B
108393904	-2.07655037	0.001399	PIK3C2A
108386644	-2.078275367	0.005928	PFKFB2
108403151	-2.079595738	0.018637	TGFB2
108386320	-2.088411524	0.022537	UBXN2A
108390969	-2.089995291	0.028502	PANK3
108400241	-2.090293353	0.044494	SQRDL
108394031	-2.091305916	0.03988	SGK1
108391330	-2.094596136	0.000282	TRIB1

108401571	-2.096617133	0.013402	OSMR
108404078	-2.101722731	0.000402	XDH
108391856	-2.106048228	0.036054	KCNK12
108390664	-2.110488338	0.014224	GMCL1
108400155	-2.110660999	0.017093	RHBDL2
novel.8783	-2.11116386	0.00323	-
108401945	-2.111363057	0.019369	SRXN1
108387001	-2.112531435	0.024283	ETS2
108391499	-2.118430821	0.035182	ITGB8
108398766	-2.122188718	0.047224	PLAU
108404836	-2.122355726	0.02685	SERTAD1
108405201	-2.123545212	0.037213	LOC108405201
108392107	-2.125961269	0.037062	LOC108392107
108396014	-2.133152778	0.002748	ABCA12
108396120	-2.136421485	0.0012	CBR3
108408102	-2.138967026	0.005197	RAP2B
108386533	-2.140553232	0.049538	LOC108386533
108387747	-2.145437019	0.02497	LOC108387747
108395052	-2.154657818	0.018489	IER5
108403285	-2.156552695	0.039954	SYNM
novel.8694	-2.164187459	0.002579	-
108398029	-2.167601613	0.009047	DDX21
108394451	-2.169942249	0.034847	FMR1
novel.5127	-2.171262338	0.037062	-
108385188	-2.176100799	0.019689	CERS6
108396680	-2.177724692	0.039964	TSNAX
108394869	-2.180427301	0.001225	DSC3
108392867	-2.183674229	0.001653	FAS
108390852	-2.185688043	0.036593	MICALCL
108398314	-2.187935767	0.026225	HSPA5
novel.8288	-2.188074382	0.032212	-
108387606	-2.188572933	0.049097	SCNN1A
108390379	-2.190108327	0.00585	LOC108390379
108402801	-2.197831947	0.001529	FHL2
108394306	-2.199844071	0.02599	MAP2
108403343	-2.203683499	0.010092	FOXN3
108389486	-2.204103647	0.035601	LOC108389486
108409763	-2.208463654	0.013545	NQO2
108394552	-2.209527193	0.010107	DYRK3
108396775	-2.221235769	0.028889	RORC
108393697	-2.224316229	0.028889	LRRC8B
108407758	-2.225592338	0.003422	SGK3
108396648	-2.234273112	0.020387	SPOPL
novel.4720	-2.239181526	0.004098	-

108383514	-2.239278668	0.00855	FAM46B
108406227	-2.239288344	0.035261	PDK4
108408006	-2.24098709	0.00311	KCNF1
108396428	-2.243375136	0.011876	P2RY1
108394115	-2.244502693	0.001526	GPAT3
108386468	-2.250723673	0.000858	SLC36A1
108404987	-2.253043235	0.032761	DST
108395847	-2.253565148	0.046771	FZD5
108386888	-2.257632369	0.047352	LOC108386888
108395068	-2.260267165	0.012683	SRF
108398232	-2.261457556	0.032986	ETF1
108393229	-2.262829796	0.013086	CMTM8
108399749	-2.264331842	0.010707	TEAD4
108403071	-2.266101165	0.015116	MITF
108386068	-2.268561005	0.000321	STAT3
108401024	-2.277928662	0.033403	EHF
108403627	-2.280481565	0.000863	PCGF5
108402523	-2.281410055	0.032966	NFKBIA
108404736	-2.282681644	0.038305	LOC108404736
108406456	-2.282692624	0.001345	ABHD5
108409106	-2.28596277	0.007301	AREG
108386310	-2.286617568	0.034004	ATAD2B
108391269	-2.2902528	0.000534	ADAMTS5
108399591	-2.299820403	0.041571	SOX9
108409437	-2.300106588	0.037576	PLN
108409103	-2.301964342	0.008767	PQLC3
108392428	-2.303964383	0.005642	SIK1
108401057	-2.304118225	0.038305	TCTEX1D1
108396658	-2.308431302	0.022175	DDR2
108390534	-2.309070438	0.002077	AHCYL2
108397145	-2.312997057	0.011417	LOC108397145
108408594	-2.315746516	0.001225	CPEB2
108403497	-2.317301345	0.042547	TREX2
108407501	-2.317456212	0.031078	FHAD1
108397262	-2.321695196	0.013739	PAQR3
108385876	-2.322335316	0.030175	FADS6
108405298	-2.323552409	0.034586	TMED6
108384576	-2.324174926	0.022749	HIGD1A
108406403	-2.325592719	0.022265	EPAS1
108391607	-2.33241497	0.009516	KLF9
108405197	-2.334508213	5.35E-05	KDM7A
novel.8165	-2.335003213	0.010738	-
novel.668	-2.337081904	0.002144	-
108402547	-2.341381221	0.035829	ELOVL7

108387504	-2.341890541	0.002511	ARHGEF5
108387620	-2.346348124	0.000453	LPAR5
108390562	-2.346838265	0.004871	DENND5B
novel.5907	-2.346872901	0.032922	-
108396236	-2.349320665	0.04022	LOC108396236
108389977	-2.352607527	0.009872	HK2
108410364	-2.354667446	0.041849	NLRC5
108408790	-2.35770186	0.00033	TAOK1
108408945	-2.362692787	0.025883	LOC108408945
108387633	-2.373207265	0.003765	LOC108387633
108383728	-2.385814795	0.030409	SLC36A2
108392816	-2.393118009	0.015923	LOC108392816
108403187	-2.393916982	0.009484	LNX1
108399405	-2.397091282	0.011557	LRCH1
108391796	-2.397170174	0.010162	GPX3
novel.2256	-2.39728957	0.023294	-
108393228	-2.400266307	0.017511	IL6ST
108384925	-2.40076892	0.014232	SDC4
108397156	-2.40672532	0.026169	GPX2
108397491	-2.4095231	0.035888	NEDD9
108400958	-2.4147806	0.006853	LOC108400958
108408853	-2.415055219	0.027407	ETS1
108386839	-2.424658732	0.001672	LOC108386839
108402128	-2.427216853	0.005909	ZBTB16
108405577	-2.427640834	0.044133	LOC108405577
108387604	-2.431186772	0.030228	PGM2
108408574	-2.431310377	0.032115	SNX20
novel.7738	-2.43550142	0.000135	-
novel.136	-2.436199836	0.002775	-
novel.1567	-2.43951136	0.041313	-
108390458	-2.441226591	0.001646	THRB
novel.8386	-2.441314685	0.044607	-
108396351	-2.442786421	0.020387	LOC108396351
108385837	-2.450079944	0.007217	CALM1
108405891	-2.456725122	0.018423	MAT2A
108394461	-2.45982135	0.00047	IL13RA1
108410142	-2.461012233	0.027248	DEFB1
108398768	-2.46720604	0.021644	LOC108398768
108387384	-2.467733512	0.020076	LOC108387384
108384616	-2.468936846	0.027727	ACSL4
108387804	-2.471205198	0.00598	HECTD1
108403236	-2.473485856	0.002008	MCL1
108385056	-2.482262859	0.011557	MPZL3
novel.2176	-2.485987864	0.025212	-

108383955	-2.487413775	0.017544	SPINK7
108390802	-2.488878163	0.049144	TIPARP
108402693	-2.489006388	0.032634	ZNF699
108386371	-2.489857716	0.011268	S100A9
108402822	-2.490468851	0.040595	ARSJ
108383576	-2.493135188	0.007315	TCP11L2
108399719	-2.496451213	0.046013	TNFSF13B
108406197	-2.497066152	0.032789	ACER1
108387859	-2.499740273	0.018761	LOC108387859
108392346	-2.50370952	0.003642	DDX3X
108397942	-2.513012196	0.002775	CUNH6orf141
108400897	-2.519480312	0.000622	NDE1
108383701	-2.519592387	0.025065	LOC108383701
108387481	-2.52037197	0.041288	SRGN
108403510	-2.520516511	0.012219	IGFBP3
108395279	-2.522574369	0.000512	MICAL2
108402472	-2.537529548	0.007175	GPT2
108399300	-2.543279205	0.001882	IL36RN
108396450	-2.544267751	0.000461	PRDM1
108389922	-2.544930618	0.038102	TMEM2
108392697	-2.545426552	0.005855	TRPV3
108384134	-2.548514891	0.008967	LOC108384134
108402244	-2.550862633	0.002772	SLC5A10
108403053	-2.553149398	0.02143	ADAM9
108405121	-2.55759218	0.000293	STOM
108387980	-2.564759608	0.01772	EGLN3
108393744	-2.566226695	0.005713	RARRES1
108401294	-2.566934551	0.013158	SERPINB1
108389583	-2.567711746	0.013909	TMEM86A
108404752	-2.570579089	0.00898	PTGER4
108390201	-2.573485079	0.002424	RASSF5
108402999	-2.585206618	0.001447	TTPA
108408672	-2.585649161	0.018464	RGS2
108389149	-2.601396134	0.024156	GCNT4
108399406	-2.603544907	0.015639	LOC108399406
108395454	-2.604300693	0.008053	NCCRP1
108388405	-2.606910991	0.004049	LOC108388405
novel.3690	-2.626538356	0.01523	-
108391152	-2.629754888	0.003132	ERRFI1
108389692	-2.633876969	0.015521	LOC108389692
108402794	-2.636506849	0.004974	GLRX
108392259	-2.637938994	0.020627	LOC108392259
novel.4382	-2.646703191	0.025566	-
108405984	-2.662240483	0.008529	SPSB1

108408023	-2.667265014	0.008839	LOC108408023
108390806	-2.667593066	0.002157	IFFO2
novel.1119	-2.667754584	0.03371	-
108397630	-2.668165711	0.013287	PTGER3
novel.8854	-2.674378804	0.01554	-
108391353	-2.674951742	0.042862	LOC108391353
108388907	-2.676738201	0.026223	LOC108388907
108391159	-2.677866554	0.000644	SOAT1
108388281	-2.680262242	0.012836	NDRG1
108405567	-2.683292691	0.03373	LOC108405567
108391579	-2.684993362	5.33E-05	AOX1
novel.8657	-2.685960182	0.005182	-
108409946	-2.688901902	0.041439	LOC108409946
novel.2520	-2.692478191	0.006873	-
108386363	-2.694974221	0.01729	S100A12
novel.4389	-2.697002649	0.005693	-
108403232	-2.700663221	0.000539	LOC108403232
novel.7319	-2.703038633	0.009768	-
108387872	-2.712467553	0.000119	SCUBE2
novel.3671	-2.717300939	0.018761	-
108386933	-2.721598136	0.043431	LOC108386933
novel.4273	-2.721922324	0.020943	-
novel.3929	-2.728530244	2.15E-05	-
108393354	-2.729322726	0.012017	LOC108393354
108392348	-2.729635776	0.025784	LOC108392348
108405130	-2.73061121	0.030175	GLUL
novel.8722	-2.735075865	0.041571	-
108399224	-2.740146417	0.003689	ZDHHC9
108399792	-2.745297721	0.01922	CUNH2orf54
108401267	-2.74594929	0.005352	MASP1
108392237	-2.753432098	0.000666	MBP
108398928	-2.756333233	0.01958	IL7
108386982	-2.763132954	4.87E-06	CTH
108386298	-2.763168546	0.03371	XKRX
108407430	-2.771325081	0.039684	ASB2
108393425	-2.772057099	0.003133	CD79A
108396532	-2.781487682	0.042862	LOC108396532
108395255	-2.781819966	0.000508	MAPK6
108386365	-2.785060203	0.004466	S100A8
108396761	-2.790952528	0.006891	RORA
108395700	-2.800738334	0.017743	THSD7A
108399810	-2.805482791	0.003215	PRSS22
108392181	-2.807119627	2.38E-05	MAP3K8
108397481	-2.820020134	0.030966	LOC108397481

108393646	-2.822091019	0.000675	SKIL
novel.3620	-2.83076533	0.025313	-
108384933	-2.835389865	0.000198	CXADR
108401965	-2.836298767	0.004142	KSR2
108389699	-2.840674567	0.030126	LOC108389699
108403088	-2.844409243	0.003162	NQO1
108392655	-2.84490806	0.009484	CSF2RB
108389446	-2.846270895	0.041042	LOC108389446
108404983	-2.855682713	0.020212	TMPPE
108398292	-2.856492573	0.004956	LOC108398292
108385953	-2.85790372	0.0163	LOC108385953
108390232	-2.858342255	0.003922	PMAIP1
108393129	-2.872291892	0.000608	LOC108393129
108385228	-2.873580717	0.045532	LOC108385228
108393019	-2.881185193	0.00102	FAM134B
108407180	-2.883174089	0.001462	LOC108407180
108401588	-2.884085094	0.002657	LIN7C
108385785	-2.885921319	0.01131	SERPINB10
108398231	-2.892534897	0.00451	EGR1
108398225	-2.894072574	0.045073	PI3
108401174	-2.900897012	0.002591	SGMS2
108391351	-2.901996056	0.000175	LOC108391351
108401051	-2.908871195	5.04E-07	HSPB8
novel.137	-2.915572008	0.027727	-
novel.723	-2.923390265	0.004071	-
108390102	-2.929439863	0.003293	GALNT3
108386824	-2.934260788	0.0032	KLK5
108406858	-2.940511001	0.028721	IL7R
108386879	-2.966077884	0.021808	FOSB
108395587	-2.9681675	0.001819	DUSP14
108405258	-2.975007737	1.09E-05	NFKBIZ
108391793	-2.975059495	0.006645	RASL12
108402243	-2.980168086	0.001523	FAM83G
108400185	-2.983455884	0.000325	LOC108400185
108396122	-2.984847294	0.000155	LOC108396122
108404851	-2.989631358	0.000445	CX3CL1
novel.134	-2.991365278	0.02255	-
108384161	-3.003063638	0.00082	NT5E
novel.7151	-3.006747606	0.005969	-
108387165	-3.007048732	0.003915	F2RL1
108384291	-3.045842626	0.004049	SERPINE2
108391708	-3.059298402	0.000214	SLC39A8
108398810	-3.065012776	0.004099	PACSIN1
108398929	-3.068278168	8.59E-08	MSX1

108392747	-3.075385044	0.000203	FAM65B
108391566	-3.093962681	0.000852	HSPA2
108404594	-3.097628802	0.000128	ACSS3
108387873	-3.104644798	0.000858	NRIP3
108383649	-3.104667626	0.005568	PSORS1C2
108409313	-3.105653922	8.39E-06	USP2
108385365	-3.11257048	0.000196	HS3ST3B1
108395906	-3.118841461	0.000168	ZC3H12A
108391984	-3.130653545	0.003832	B4GALT5
108410040	-3.13090382	0.002102	LAMC2
108409105	-3.137468051	0.001088	EREG
108384750	-3.140746367	9.35E-07	EVA1C
novel.5626	-3.142210061	0.000372	-
108389484	-3.142455132	0.000321	SLC25A33
108391102	-3.148059436	0.038586	HTR1D
108385275	-3.155685012	0.000243	RTP4
108386364	-3.156935611	0.001124	LOC108386364
novel.2204	-3.166985484	0.011428	-
108389109	-3.168496001	0.007267	LOC108389109
108387306	-3.170477127	0.034047	RNF183
108397323	-3.174646575	0.036593	LIPG
108407151	-3.179698737	0.005265	AGO3
108407996	-3.184731839	0.002543	CRISPLD2
108390496	-3.190730255	0.004268	LOC108390496
108387278	-3.191261808	0.018219	LOC108387278
108392186	-3.193393092	0.016768	CUNH1orf162
108383745	-3.205990288	0.003697	CPA4
108385059	-3.206147534	0.02486	PLCXD2
108404710	-3.207956888	0.043134	IL1R2
108387224	-3.22630445	0.007534	LOC108387224
novel.8002	-3.240151953	0.003128	-
108400147	-3.240227646	0.031553	THBS1
108406746	-3.241053103	2.62E-06	LOC108406746
108393758	-3.249153252	0.040247	PLEKHD1
108401231	-3.253581772	0.044494	LOC108401231
108389727	-3.26392893	0.036054	LOC108389727
108404824	-3.274524254	0.000596	STEAP1
108400250	-3.280513676	0.000338	LOC108400250
108402218	-3.286991059	0.003422	MLANA
108386768	-3.290592231	0.03092	LOC108386768
108399296	-3.290948691	0.002067	PAX8
108386976	-3.292090081	0.002409	LOC108386976
novel.3969	-3.293679991	0.011417	-
108402643	-3.29504395	0.001972	CRCT1

108390221	-3.297147023	0.015082	PECAM1
108397672	-3.303108259	0.000296	EPGN
108407130	-3.303287194	0.001859	DHRS9
108386645	-3.305113562	1.52E-05	YOD1
108395779	-3.322617368	0.031092	SERPINE1
108386821	-3.328985227	0.00262	KLK9
108399939	-3.337386998	0.000163	IL33
108402797	-3.341396229	0.002547	CNST
novel.7501	-3.346690184	0.026121	-
108407802	-3.351544971	0.002772	SOCS3
108408065	-3.358873787	0.00023	RASEF
108383541	-3.360561797	0.004972	TMEM221
108400512	-3.370965802	0.048734	ARC
108405714	-3.374275757	0.027322	LOC108405714
108405324	-3.377102018	5.32E-05	LOC108405324
108402467	-3.378056493	1.20E-06	LOC108402467
novel.6890	-3.384711826	9.24E-06	-
novel.1313	-3.388623098	0.002979	-
108402417	-3.397196892	0.048075	SLC22A7
108403859	-3.400098207	0.012313	C7
108410343	-3.401943595	0.001829	SLC10A6
108389096	-3.401979835	0.038102	CDHR1
108391526	-3.41007114	0.015181	ALPL
novel.2748	-3.4298104	0.003107	-
108389114	-3.432223369	0.024275	LOC108389114
108388537	-3.432908398	4.12E-06	LOC108388537
108400399	-3.434607307	0.034586	LOC108400399
108392522	-3.439362089	0.001379	MAK
108398048	-3.440446856	0.007006	IDE
108396020	-3.440454236	9.51E-05	FNIP2
108389072	-3.460231294	0.005228	LOC108389072
108407237	-3.460490699	0.002997	LOC108407237
108406161	-3.464997704	0.018637	LEPR
108404939	-3.46849658	0.01157	LOC108404939
108405438	-3.470822199	0.000607	GPNMB
108404717	-3.481902969	0.005911	LOC108404717
108383461	-3.484610513	0.008803	SPTLC3
novel.5839	-3.486940092	0.001078	-
108392821	-3.489596154	0.02995	LOC108392821
108407904	-3.489610287	0.004014	LOC108407904
108403269	-3.490180851	0.013444	MS4A7
108407559	-3.497679525	1.01E-05	SLC6A6
108384252	-3.505261797	0.031392	LOC108384252
108404282	-3.508769469	0.002198	LOC108404282

108386367	-3.527577415	0.034586	LOC108386367
108407609	-3.528990238	0.00634	LOC108407609
108409104	-3.529401693	0.013495	CUNH2orf50
108400872	-3.530627611	0.002049	LOC108400872
108396130	-3.542810276	0.028889	SLC10A7
novel.4272	-3.543897668	0.028889	-
108393034	-3.555968798	0.004813	IL1B
novel.4993	-3.561814614	0.008259	-
108392503	-3.566285742	0.024381	CHAC1
108403475	-3.566717795	7.53E-06	GABRP
novel.8655	-3.573016208	1.77E-06	-
108384684	-3.580119188	0.016808	LCN2
108396321	-3.596084349	1.70E-05	RCAN1
108399444	-3.603522842	0.000326	GPR68
108396774	-3.616300191	0.000675	LINGO4
108404646	-3.625410043	0.030175	LOC108404646
novel.5169	-3.629015846	0.000338	-
novel.7511	-3.633955151	0.000135	-
108405108	-3.635093647	0.036056	TMEM173
novel.7181	-3.639984832	0.018873	-
108399668	-3.657627626	0.000266	BEST2
108407108	-3.658532282	0.004206	LOC108407108
novel.3598	-3.662403961	0.004001	-
108383259	-3.665455365	0.003697	CA4
108383755	-3.676862959	0.020855	CCL4
108401017	-3.677828953	0.001337	TNFSF15
108389172	-3.683335473	0.025784	LOC108389172
108399575	-3.689918191	0.000112	KRT16
108401873	-3.706201578	0.000606	TIMP1
108391202	-3.712852178	0.024533	TCF23
novel.6967	-3.714947111	0.032133	-
108394994	-3.715102644	0.012596	ATP12A
108386941	-3.719256119	3.48E-07	SLC9A3
108399809	-3.72098575	5.42E-05	PRSS27
108404185	-3.731889991	2.13E-05	LOC108404185
108394173	-3.73776894	6.93E-08	LIPN
108402160	-3.743773621	0.004376	ISG20
novel.7863	-3.760519312	0.005642	-
108385203	-3.7639822	0.000348	S100P
108408086	-3.766896929	0.016242	LOC108408086
108390269	-3.767467825	0.018343	LOC108390269
novel.8603	-3.777814318	0.001962	-
108410135	-3.799485773	1.70E-05	LOC108410135
108393355	-3.804813735	1.26E-05	LOC108393355

novel.18	-3.807976862	0.023155	-
108388152	-3.809735809	2.91E-09	LOC108388152
108396933	-3.810695653	0.042355	LOC108396933
108406725	-3.813863374	0.001824	PKDREJ
108384456	-3.814441368	0.014297	LOC108384456
novel.7119	-3.854121694	0.034941	-
108400809	-3.865516847	0.015923	LOC108400809
108401684	-3.870384291	0.018655	LOC108401684
108395455	-3.87751279	0.002231	SYCN
108392757	-3.882161311	0.001419	LOC108392757
108393322	-3.882723801	0.01554	LOC108393322
108383423	-3.883917196	9.66E-05	LVRN
108386180	-3.889971038	0.015764	LOC108386180
108390295	-3.890001841	0.002129	LOC108390295
108400435	-3.892842041	1.70E-05	TRIB3
108407446	-3.89710162	1.01E-05	LOC108407446
108395198	-3.922283965	0.015303	LOC108395198
108390085	-3.930538427	0.013151	LOC108390085
108383388	-3.936048063	9.00E-05	ALOX15B
108407560	-3.946726132	2.24E-07	LOC108407560
108394952	-3.950807797	6.91E-05	FOSL1
novel.7061	-3.953018507	0.024283	-
108407434	-3.954020504	0.000396	OTUB2
novel.5366	-3.957513599	0.014548	-
108396613	-3.961331027	0.006502	LOC108396613
108405328	-3.963875289	0.005324	KPRP
108398497	-3.96529537	0.006349	PADI3
108398031	-3.965703936	0.043547	LOC108398031
108402134	-3.96574475	0.02111	LOC108402134
108396749	-3.974992058	7.41E-06	HSD11B1
108401780	-3.98673855	9.19E-05	C3
108385697	-3.988083903	4.45E-09	NEU2
108408511	-3.995037635	0.001242	HOXC10
108409762	-4.001295038	2.47E-05	LOC108409762
108405113	-4.005602849	0.001552	MZB1
108386822	-4.017113543	0.000177	KLK6
novel.669	-4.030854822	0.003824	-
108409356	-4.037542385	4.85E-12	NAMPT
108395127	-4.059478644	0.03332	LYG1
108383995	-4.068912791	0.000338	S100A3
108394334	-4.071558948	6.93E-05	LOC108394334
novel.3441	-4.089478667	0.018483	-
108409024	-4.095673202	0.002709	SELP
108408021	-4.097782206	3.30E-05	LOC108408021

108388007	-4.09818847	9.57E-05	SLC5A5
108397063	-4.101709112	0.01895	LOC108397063
108401902	-4.113934105	1.81E-06	FAM83D
novel.4694	-4.121361181	0.004092	-
novel.1690	-4.132849001	1.71E-05	-
108401368	-4.133156913	1.31E-05	CILP2
108407665	-4.139843001	0.019287	ADGRV1
novel.4274	-4.153747811	0.001276	-
novel.1067	-4.154598054	2.34E-07	-
108391441	-4.161439145	7.39E-05	HPGD
108393599	-4.170962227	0.000164	CCL26
novel.6891	-4.172277574	0.000852	-
novel.6455	-4.202261767	0.011417	-
108399581	-4.202689153	0.046666	LOC108399581
108393520	-4.206047515	0.004498	LOC108393520
108399084	-4.229202231	0.003822	KLHDC8A
108408074	-4.235855919	1.49E-05	LOC108408074
108383655	-4.252268707	3.74E-06	CDSN
novel.1174	-4.270051345	0.025212	-
novel.5662	-4.292564281	0.000277	-
108405327	-4.293874324	0.000845	CUNH1orf68
108403845	-4.301871537	8.25E-11	AQP9
108399294	-4.302824146	4.47E-05	LOC108399294
108392827	-4.310606298	4.65E-06	EDNRB
novel.7910	-4.362054479	0.020343	-
108388266	-4.365877448	0.001819	FCGR2B
novel.611	-4.377891254	9.58E-06	-
108387915	-4.382799917	0.001341	LOC108387915
108387501	-4.384988083	4.03E-05	TGM3
108388080	-4.420110411	0.024303	CLEC7A
novel.6277	-4.423192571	0.000219	-
108409046	-4.429258692	3.60E-13	SCNN1D
novel.1571	-4.447614188	0.000283	-
108409509	-4.457740703	0.006484	CXCL5
108392820	-4.458359771	0.002754	LOC108392820
108392288	-4.46137459	0.00622	LOC108392288
novel.1308	-4.462604565	0.043072	-
108389053	-4.467337609	8.78E-06	BIRC3
108395366	-4.497330974	1.50E-08	CCL19
108386141	-4.519864514	0.039018	LOC108386141
108406790	-4.527829114	1.89E-07	NABP1
108384675	-4.527973206	0.014019	LOC108384675
novel.5977	-4.533878207	6.55E-06	-
108403781	-4.536850963	0.04505	LOC108403781

108387358	-4.566064481	0.006201	LOC108387358
108385644	-4.566499103	0.00262	LOC108385644
108407481	-4.585991839	0.000972	TNIP3
novel.4486	-4.587357985	0.003832	-
108400202	-4.620726136	3.24E-06	CHPT1
novel.6756	-4.623870247	0.00015	-
novel.6231	-4.62627798	2.47E-06	-
novel.6458	-4.638358785	0.049765	-
108398693	-4.643697152	0.000382	AK7
108392273	-4.660585614	0.027924	LOC108392273
108383752	-4.675740048	1.50E-05	LOC108383752
108402681	-4.676895898	6.96E-05	CA6
novel.5146	-4.676977666	1.43E-06	-
novel.7865	-4.693026138	0.038703	-
novel.6067	-4.694612899	0.00262	-
108390301	-4.725563476	0.001278	LOC108390301
novel.2106	-4.741963711	0.001799	-
novel.2963	-4.745342313	4.57E-08	-
108406350	-4.750920726	0.041313	POU2AF1
novel.2898	-4.791058319	1.49E-05	-
108383956	-4.802279598	0.000164	CUNH5orf46
108385678	-4.831586503	8.34E-06	KNG1
108394008	-4.895295919	0.005592	LOC108394008
108384166	-4.913616195	0.000539	LOC108384166
108389082	-4.930115863	0.010626	LOC108389082
108393195	-4.93495133	6.03E-12	SLC39A14
108387314	-4.943610236	0.035782	LOC108387314
novel.6137	-4.94916062	0.019854	-
novel.1651	-4.958031998	0.000986	-
108391632	-5.006738416	2.95E-07	SMPD3
novel.5978	-5.060386779	0.007851	-
108398159	-5.083443958	8.24E-06	KCTD4
108383363	-5.085846914	2.81E-05	HSD17B3
108399388	-5.104919629	1.41E-05	LOC108399388
108404321	-5.107263226	0.016666	LOC108404321
novel.45	-5.126706738	0.000204	-
novel.2782	-5.149586372	8.52E-05	-
108390150	-5.159720802	3.36E-06	CCL20
novel.1146	-5.166811188	0.000112	-
108389161	-5.170364389	0.009183	LOC108389161
108401147	-5.170745873	0.00277	LOC108401147
108396559	-5.177690062	0.000703	LOC108396559
108407235	-5.179543409	0.000437	LOC108407235
novel.6272	-5.23264325	1.53E-07	-

novel.6941	-5.235031549	0.002964	-
novel.5743	-5.251814862	0.005076	-
108400596	-5.252609052	1.53E-06	LOC108400596
108388083	-5.27168581	0.008082	TMEM52B
108401676	-5.274921092	0.000579	CHI3L1
108388227	-5.343972498	0.000184	LOC108388227
108383629	-5.350503647	7.10E-06	LOC108383629
108402130	-5.381616828	3.84E-15	NNMT
novel.7564	-5.449229819	2.02E-05	-
108408910	-5.470731801	9.71E-05	KRT27
108395397	-5.555238237	8.06E-11	LOC108395397
108388560	-5.645419286	0.042862	LOC108388560
novel.6075	-5.646980347	0.028583	-
108405889	-5.782094369	0.004235	GNLY
108390481	-5.794449492	4.16E-05	GPRC5D
108393320	-5.801744099	0.000339	KRT32
novel.3680	-5.84252062	0.015764	-
108397153	-5.891836905	4.16E-05	LOC108397153
108385402	-5.892877277	0.00033	LOC108385402
108384163	-5.897795233	0.014088	LOC108384163
108389651	-5.900370347	3.17E-06	LOC108389651
novel.7078	-5.935486443	0.020223	-
108399738	-5.979512648	2.48E-05	LRRC15
novel.6368	-5.997031756	6.23E-06	-
novel.2571	-6.020181447	3.08E-05	-
novel.7860	-6.04028817	0.018823	-
108402973	-6.042462821	1.69E-07	KRT71
108389953	-6.101553416	1.08E-13	LOC108389953
novel.5418	-6.106536808	6.19E-05	-
108397154	-6.109406673	2.40E-05	LOC108397154
108384557	-6.127330955	0.041202	LOC108384557
108399571	-6.153767023	3.52E-06	LOC108399571
108398582	-6.173618555	0.015424	LOC108398582
108394235	-6.186370041	8.07E-06	JCHAIN
108405481	-6.200376369	1.41E-07	STEAP4
108399823	-6.223992271	0.044641	LOC108399823
108397718	-6.231095562	0.043509	CUNH1orf94
novel.7403	-6.258773489	5.64E-09	-
108389952	-6.309392424	9.55E-09	LOC108389952
108406440	-6.330076181	7.30E-05	CXCL8
novel.8847	-6.338297439	0.02056	-
novel.7861	-6.341651549	0.026487	-
novel.4065	-6.347845153	0.029967	-
108403650	-6.358991076	0.045229	SPINK2

novel.2653	-6.514349115	0.00012	-
108398533	-6.518537445	0.020493	LOC108398533
108385034	-6.569158253	1.20E-06	ANGPTL5
108383630	-6.571041224	0.004955	LOC108383630
108384175	-6.589529802	3.46E-11	LY6D
novel.3457	-6.600706513	2.66E-07	-
novel.3467	-6.621450012	0.014224	-
novel.702	-6.653168851	0.002293	-
108391723	-6.666469103	0.040743	CLDN17
novel.5926	-6.705792951	2.80E-05	-
novel.1885	-6.713063717	0.023487	-
novel.2421	-6.733501301	0.031077	-
novel.379	-6.745390698	0.016768	-
108390594	-6.753909363	0.049313	LOC108390594
novel.4573	-6.753986784	0.033246	-
novel.1050	-6.774977011	0.040687	-
novel.415	-6.782321415	0.002514	-
novel.1318	-6.797213542	0.009718	-
novel.6866	-6.819231326	0.036202	-
novel.5363	-6.830571272	0.038703	-
novel.6211	-6.857340649	0.017089	-
108408912	-6.870997792	6.86E-07	KRT25
108408510	-6.872348179	0.002547	HOXC11
108390127	-6.875090374	0.04383	CUNH6orf222
108393711	-6.887333539	0.016808	LOC108393711
novel.8551	-6.889046082	0.005076	-
108400568	-6.966024027	1.59E-21	SOD2
108408372	-7.004072732	0.038955	LOC108408372
novel.127	-7.030224108	0.016666	-
novel.8072	-7.083743479	0.042395	-
novel.8378	-7.131996198	0.041849	-
108395398	-7.1608527	0.003036	LOC108395398
108391725	-7.163597139	0.012198	LOC108391725
108388163	-7.16564856	5.44E-08	SLC6A15
108404201	-7.212880121	0.006058	FEZF1
108402088	-7.224197343	0.013909	ARG1
108408024	-7.228091639	0.009631	LOC108408024
108393319	-7.234185829	1.79E-08	LOC108393319
108408630	-7.255496734	0.003132	CDHR5
novel.2015	-7.263786898	0.005855	-
108383508	-7.280345422	0.002662	STMND1
novel.5383	-7.300601754	0.009991	-
108406072	-7.344680784	0.014773	LOC108406072
novel.2107	-7.349643108	0.002465	-

novel.398	-7.378779573	0.003944	-
108396465	-7.395655733	0.002333	LOC108396465
108388106	-7.41903288	6.78E-08	LOC108388106
108397657	-7.430919172	0.013041	LOC108397657
108399439	-7.450665283	0.001062	TRNAF-GAA
novel.5910	-7.461381305	0.022052	-
novel.2871	-7.485778115	0.00329	-
novel.6304	-7.5006908	0.006891	-
novel.7859	-7.500887563	0.001263	-
108405399	-7.528545034	0.001187	LOC108405399
novel.5444	-7.533874485	0.004247	-
novel.5927	-7.554427425	0.001644	-
108398673	-7.589603834	1.07E-11	SERPINA12
novel.6960	-7.655236639	0.001368	-
108397853	-7.661596477	0.000243	LY6G6D
108395690	-7.665725259	2.49E-06	TRH
108392823	-7.669396665	4.95E-09	LOC108392823
novel.1694	-7.693560371	0.000173	-
108385153	-7.72949108	0.005272	CSF3
108383626	-7.746794948	0.002231	LOC108383626
novel.492	-7.766910085	0.005538	-
novel.236	-7.848146115	0.003642	-
108383627	-7.850710462	0.000825	LOC108383627
108406627	-7.937171599	0.000279	GPR50
108391945	-7.951328184	0.013405	LOC108391945
108408091	-8.098640215	0.002511	LOC108408091
108388622	-8.149438819	0.000121	LOC108388622
novel.2910	-8.1944753	0.000148	-
novel.1363	-8.291494257	0.00151	-
108408282	-8.415047574	0.000309	LOC108408282
108403607	-8.440887085	3.11E-05	LOC108403607
novel.7489	-8.499378096	7.42E-05	-
novel.6751	-8.536977277	0.000396	-
108404212	-8.542889566	0.000574	LOC108404212
108403606	-8.602910413	3.42E-05	LOC108403606
novel.4225	-8.604839888	0.00023	-
108405469	-8.646614321	0.000276	NLRP3
novel.7487	-8.743784392	5.46E-05	-
novel.1173	-8.756157076	0.000321	-
novel.7183	-8.86338055	0.000101	-
108384948	-8.976449002	3.12E-05	CCDC60
novel.8114	-8.995372213	2.13E-05	-
108396601	-8.999151255	8.78E-05	LOC108396601
108407711	-9.037532145	2.89E-06	CUNH8orf46

108391726	-9.168594331	0.000292	LOC108391726
novel.7239	-9.191618935	1.85E-05	-
108409550	-9.458779746	0.036425	LOC108409550
108408464	-9.599148213	2.22E-06	LOC108408464
108398247	-10.19911641	2.76E-06	LOC108398247
108392719	-10.22139949	6.66E-08	LOC108392719
108389176	-10.7385479	7.15E-08	LOC108389176
108385898	-11.21865411	6.46E-20	LOC108385898

Supplementary Table 3 Significant enrich terms in GO enrichment analysis with up-regulated DEGs in adult-hair group (embryo-hair vs adult-hair group)

GO ID	Description	p-value	padj	Gene ID	Gene Name	Count
GO:0006954	inflammatory response	3.27E-06	0.001320413	108399294/108399300/108393034/108389446/108389056/108405149/108399293	LOC108399294/IL36RN/IL1B/LOC108389446/LOC108389056/IL4R/IL1F10	7
GO:0006955	immune response	5.98E-05	0.012078287	108390150/108399294/108406440/108391351/108404851/108401017/108399300/108393034/108385153/108409509/108398928/108389446/108399719/108392461/108407514/108389056/108405685/108396370/108406189/108399293	CCL20/LOC108399294/CXCL8/LOC108391351/CX3CL1/TNFSF15/IL36RN/IL1B/CSF3/CXCL5/IL7/LOC108389446/TNFSF13B/ENDOU/CCL16/LOC108389056/GCSAML/LOC108396370/TNFSF9/IL1F10	20
GO:0002376	immune system process	9.76E-05	0.013137402	108390150/108399294/108406440/108391351/108404851/108401017/108399300/108393034/108385153/108409509/108398928/108389446/108399719/108392461/108407514/108389056/108405685/108396370/108406189/108399293	CCL20/LOC108399294/CXCL8/LOC108391351/CX3CL1/TNFSF15/IL36RN/IL1B/CSF3/CXCL5/IL7/LOC108389446/TNFSF13B/ENDOU/CCL16/LOC108389056/GCSAML/LOC108396370/TNFSF9/IL1F10	20
GO:0006952	defense response	0.000309111	0.03122024	108399294/108399300/108393034/108389446/108389056/108405149/108399293	LOC108399294/IL36RN/IL1B/LOC108389446/LOC108389056/IL4R/IL1F10	7
GO:0090066	regulation of anatomical structure size	0.000468551	0.03785893	108408339/108392546/108384264/108407971/108394598/108408243/108386352	EDN1/TMOD3/EDN2/ARPC4/ARPC5L/AP1AR/CAPZA1	7
GO:0006508	proteolysis	0.000766426	0.041418671	108385898/108409313/108399809/108386822/108391269/108387873/108404052/108386821/108386824/108399810/108383745/108401267/108409555/108404939/108400809/108404565/108403053/108385611/108384826/108398766/	LOC108385898/USP2/PRSS27/KLK6/ADAMTS5/NRIP3/CPD/KLK9/KLK5/PRSS22/CPA4/MASP1/CACUL1/LOC108404939/LOC108400809/ZMPSTE24/ADAM9/PRSS8/C1S/PLAU/MMP17/LAP3/USP38/LOC108390504/C2/LOC1083	53

				108399376/108400146/108402345/108390504/108393991/108395086/108395378/108393577/108390716/108387497/108390924/108383254/108404280/108394492/108397532/108391133/108392608/108402778/108386448/108393217/108399339/108384976/108392400/108386194/108403950/108393107/108386820/108402528/108400905/108397466/108402364/108385790/108395511	95086/PSEN1/USP45/LOC108390716/CPA6/PCSK1/USP32/SENP3/CPM/CFI/MMP13/LOC108392608/LOC108402778/LOC108386448/ADAMDEC1/JOSD1/TMPRSS4/UCHL3/LOC108386194/USP53/CTSC/KLK7/PSMA6/LOC108400905/LOC108397466/CTSB/FURIN/USP12	
GO:0018149	peptide cross-linking	0.000820172	0.041418671	108387501/108389727/108388714/108390032/108386620	TGM3/LOC108389727/TGM7/TGM5/TGM1	5
GO:0051130	positive regulation of cellular component organization	0.000820172	0.041418671	108395327/108407971/108394598/108408243/108406498	LOC108395327/ARPC4/ARPC5L/AP1AR/EIF5A	5
GO:0007015	actin filament organization	0.000979673	0.043976415	108392259/108384181/108392546/108407971/108394598/108408243/108386352	LOC108392259/WASL/TMOD3/ARPC4/ARPC5L/AP1AR/CAPZA1	7
GO:0005882	intermediate filament	1.53E-11	7.21E-10	108392823/108393319/108402973/108408912/108399571/108383629/108408910/108399575/108393320/108383627/108390806/108383626/108392820/108393322/108408086/108399574/108392821/108403285/novel.8378/108408918/108408913/108393327/108401984/108408088/novel.8569/108402986/108383628/novel.8422/108388248/108401985	LOC108392823/LOC108393319/KRT71/KRT25/LOC108399571/LOC108383629/KRT27/KRT16/KRT32/LOC108383627/IFFO2/LOC108383626/LOC108392820/LOC108393322/LOC108408086/KRT14/LOC108392821/SYNM/-/LOC108408918/KRT39/LOC108393327/KRT82/LOC108408088/-/LOC108402986/LOC108383628/-/LOC108388248/KRT84	30

GO:0045111	intermediate filament cytoskeleton	1.53E-11	7.21E-10	108392823/108393319/108402973/108408912/108399571/108383629/108408910/108399575/108393320/108383627/108390806/108383626/108392820/108393322/108408086/108399574/108392821/108403285/novel.8378/108408918/108408913/108393327/108401984/108408088/novel.8569/108402986/108383628/novel.8422/108388248/108401985	LOC108392823/LOC108393319/KRT71/KRT25/LOC108399571/LOC108383629/KRT27/KRT16/KRT32/LOC108383627/IFFO2/LOC108383626/LOC108392820/LOC108393322/LOC108408086/KRT14/LOC108392821/SYNM/-/LOC108408918/KRT39/LOC108393327/KRT82/LOC108408088/-/LOC108402986/LOC108383628/-/LOC108388248/KRT84	30
GO:0099513	polymeric cytoskeletal fiber	4.22E-11	1.32E-09	108392823/108393319/108402973/108408912/108399571/108383629/108408910/108399575/108393320/108383627/108390806/108383626/108392820/108393322/108408086/108399574/108392821/108403285/novel.8378/108408918/108408913/108393327/108401984/108408088/novel.8569/108402986/108383628/novel.8422/108388248/108401985	LOC108392823/LOC108393319/KRT71/KRT25/LOC108399571/LOC108383629/KRT27/KRT16/KRT32/LOC108383627/IFFO2/LOC108383626/LOC108392820/LOC108393322/LOC108408086/KRT14/LOC108392821/SYNM/-/LOC108408918/KRT39/LOC108393327/KRT82/LOC108408088/-/LOC108402986/LOC108383628/-/LOC108388248/KRT84	30
GO:0099080	supramolecular complex	4.91E-10	7.70E-09	108392823/108393319/108402973/108408912/108399571/108383629/108408910/108399575/108393320/108383627/108390806/108383626/108392820/108393322/108408086/108399574/108392821/108403285/novel.8378/108408918/108408913/108393327/108401984/108408088/novel.8569/108402986/108383628/novel.8422/108388248/108401985	LOC108392823/LOC108393319/KRT71/KRT25/LOC108399571/LOC108383629/KRT27/KRT16/KRT32/LOC108383627/IFFO2/LOC108383626/LOC108392820/LOC108393322/LOC108408086/KRT14/LOC108392821/SYNM/-/LOC108408918/KRT39/LOC108393327/KRT82/LOC108408088/-	30

					/LOC108402986/LOC108383628/-/LOC108388248/KRT84	
GO:0099081	supramolecular polymer	4.91E-10	7.70E-09	108392823/108393319/108402973/108408912/108399571/108383629/108408910/108399575/108393320/108383627/108390806/108383626/108392820/108393322/108408086/108399574/108392821/108403285/novel.8378/108408918/108408913/108393327/108401984/108408088/novel.8569/108402986/108383628/novel.8422/108388248/108401985	LOC108392823/LOC108393319/KRT71/KRT25/LOC108399571/LOC108383629/KRT27/KRT16/KRT32/LOC108383627/IFFO2/LOC108383626/LOC108392820/LOC108393322/LOC108408086/KRT14/LOC108392821/SYNM/-/LOC108408918/KRT39/LOC108393327/KRT82/LOC108408088/-/LOC108402986/LOC108383628/-/LOC108388248/KRT84	30
GO:0099512	supramolecular fiber	4.91E-10	7.70E-09	108392823/108393319/108402973/108408912/108399571/108383629/108408910/108399575/108393320/108383627/108390806/108383626/108392820/108393322/108408086/108399574/108392821/108403285/novel.8378/108408918/108408913/108393327/108401984/108408088/novel.8569/108402986/108383628/novel.8422/108388248/108401985	LOC108392823/LOC108393319/KRT71/KRT25/LOC108399571/LOC108383629/KRT27/KRT16/KRT32/LOC108383627/IFFO2/LOC108383626/LOC108392820/LOC108393322/LOC108408086/KRT14/LOC108392821/SYNM/-/LOC108408918/KRT39/LOC108393327/KRT82/LOC108408088/-/LOC108402986/LOC108383628/-/LOC108388248/KRT84	30
GO:0045095	keratin filament	4.70E-06	6.32E-05	108383629/108383627/108383626/novel.8378/108408088/108383628/108388248	LOC108383629/LOC108383627/LOC108383626/-/LOC108408088/LOC108383628/LOC108388248	7
GO:0044430	cytoskeletal part	1.57E-05	0.000184077	108392823/108393319/108402973/108408912/108399571/108383629/108408910/108399575/	LOC108392823/LOC108393319/KRT71/KRT25/LOC108399571/LOC108383629/KRT27/KRT1	37

				108393320/108383627/108390806/108383626/108392820/108393322/108408086/108399574/108392821/108403285/novel.8378/108408918/108408913/108393327/108401984/108408088/108398660/novel.8569/108394709/108402986/108383628/novel.8422/108400222/108386095/108388248/108407971/108394598/108401985/108386352	6/KRT32/LOC108383627/IFFO2/LOC108383626/LOC108392820/LOC108393322/LOC108408086/KRT14/LOC108392821/SYNM/-/LOC108408918/KRT39/LOC108393327/KRT82/LOC108408088/MYO1E/-/MYO1D/LOC108402986/LOC108383628/-/MYO5B/LOC108386095/LOC108388248/ARPC4/ARPC5L/KRT84/CAPZA1	
GO:0005856	cytoskeleton	2.86E-05	0.00029914	108392823/108393319/108402973/108408912/108399571/108383629/108408910/108399575/108393320/108383627/108390806/108383626/108392820/108407158/108393322/108408086/108399574/108392821/108404987/108403285/novel.8378/108408918/108408913/108393327/108401984/108408088/108398660/novel.8569/108394709/108402986/108383628/novel.8422/108400222/108386095/108388248/108407971/108394598/108401985/108386352	LOC108392823/LOC108393319/KRT71/KRT25/LOC108399571/LOC108383629/KRT27/KRT16/KRT32/LOC108383627/IFFO2/LOC108383626/LOC108392820/MAP7D1/LOC108393322/LOC108408086/KRT14/LOC108392821/DST/SYNM/-/LOC108408918/KRT39/LOC108393327/KRT82/LOC108408088/MYO1E/-/MYO1D/LOC108402986/LOC108383628/-/MYO5B/LOC108386095/LOC108388248/ARPC4/ARPC5L/KRT84/CAPZA1	39
GO:0005615	extracellular space	0.002116162	0.019891926	108399294/108401780/108399300/108393034/108389446/108393997/108406298/108405990/108399293	LOC108399294/C3/IL36RN/IL1B/LOC108389446/C4A/FGA/SOGA1/IL1F10	9
GO:0070011	peptidase activity, acting on L-amino acid peptides	0.000109996	0.030358783	108385898/108409313/108399809/108383423/108386822/108391269/108387873/108404052/108386821/108386824/108399810/108383745/108401267/108404939/108400809/108404565/	LOC108385898/USP2/PRSS27/LVRN/KLK6/ADAMTS5/NRIP3/CPD/KLK9/KLK5/PRSS22/CPA4/MASP1/LOC108404939/LOC108400809/ZMPSTE24/RHBDL2/ADAM9/PRSS8/C1S/PLA	57

				108400155/108403053/108385611/108384826/108398766/108399376/108400146/108402345/108385032/108386011/108390504/108393991/108395086/108395378/108393577/108390716/108387497/108390924/108383254/108404280/108394492/108397532/108391133/108392608/108402778/108386448/108393217/108399339/108384976/108392400/108386194/108403950/108393107/108386820/108402528/108400905/108397466/108402364/108390493/108385790/108395511	U/MMP17/LAP3/USP38/LOC108385032/ERAP1/LOC108390504/C2/LOC108395086/PSEN1/USP45/LOC108390716/CPA6/PCSK1/USP32/SENP3/CPM/CFI/MMP13/LOC108392608/LOC108402778/LOC108386448/ADAMDEC1/JOSD1/TMPRSS4/UCHL3/LOC108386194/USP53/CTSC/KLK7/PSMA6/LOC108400905/LOC108397466/CTSB/DERL1/FURIN/USP12	
GO:0008233	peptidase activity	0.000244772	0.033778557	108385898/108409313/108399809/108383423/108386822/108391269/108387873/108404052/108386821/108386824/108399810/108383745/108401267/108404939/108400809/108404565/108400155/108403053/108385611/108384826/108398766/108399376/108400146/108402345/108385032/108386011/108390504/108393991/108395086/108395378/108393577/108390716/108387497/108390924/108383254/108404280/108394492/108397532/108391133/108392608/108402778/108386448/108393217/108399339/108384976/108392400/108386194/108403950/108393107/108386820/108402528/108400905/108397466/108402364/108390493/108385790/108395511	LOC108385898/USP2/PRSS27/LVRN/KLK6/ADAMTS5/NRIP3/CPD/KLK9/KLK5/PRSS22/CPA4/MASP1/LOC108404939/LOC108400809/ZMPSTE24/RHBDL2/ADAM9/PRSS8/C1S/PLAU/MMP17/LAP3/USP38/LOC108385032/ERAP1/LOC108390504/C2/LOC108395086/PSEN1/USP45/LOC108390716/CPA6/PCSK1/USP32/SENP3/CPM/CFI/MMP13/LOC108392608/LOC108402778/LOC108386448/ADAMDEC1/JOSD1/TMPRSS4/UCHL3/LOC108386194/USP53/CTSC/KLK7/PSMA6/LOC108400905/LOC108397466/CTSB/DERL1/FURIN/USP12	57

GO:0005125	cytokine activity	0.000425371	0.039134095	108390150/108399294/108406440/108399939/108404851/108399300/108393034/108385153/108409509/108389446/108407514/108399293	CCL20/LOC108399294/CXCL8/IL33/CX3CL1/IL36RN/IL1B/CSF3/CXCL5/LOC108389446/CCL16/IL1F10	12

Supplementary Table 4 Significant enriched terms in GO enrichment analysis with up-regulated DEGs in embryo-hair group (embryo-hair vs adult-hair group)

GO ID	Description	p-value	padj	Gene ID	Gene Name	Count
GO:0005216	ion channel activity	4.46E-06	0.000344904	108385348/108403402/108383696/108383721/108389467/108406972/108399331/108401977/108386667/108402127/108409350/108390801/108402355/108392445/108385313/108402092/108390600/108383723/108404388/108400595/108393370/108395270/108408468/108393388/108395367/108399643/108402776/108400552/108392711/108383501/108402892/108386781/108404928/108404345/108404003/108386925/108384738/108398963/108394236/108386788/108395465/108394575/108398149/108400101/108404982/108405924/108403895/108402184/108399367/108395859/108397611/108392845	KCNA6/KCNQ4/CACNB1/CHRND/LOC108389467/KCNMB2/KCNJ4/LOC108401977/GRIN3A/HTR3A/GRIA2/LOC108390801/CACNA1D/TRPV4/TPCN2/KCNB2/GRIA3/CHRNG/CLCN1/LOC108400595/GLRB/CLCN2/KCNH3/GRIK5/CHRNA1/CACNA1A/CACNA1G/HVCN1/GRID2/CACNB3/LOC108402892/GRIN2D/TMEM266/GLRA4/TMEM38A/TPCN1/GABRR1/FXYD1/LOC108394236/KCNJ14/GRID1/GRIA1/GRIA4/TRPC3/KCNH8/ZACN/CATSPER2/KCNMB4/TMEM37/TRPC1/LOC108397611/LOC108392845	52
GO:0015267	channel activity	4.46E-06	0.000344904	108385348/108403402/108383696/108383721/108389467/108406972/108399331/108401977/108386667/108402127/108409350/108390801/108402355/108392445/108385313/108402092/108390600/108383723/108404388/108400595/108393370/108395270/108408468/108393388/108395367/108399643/108402776/108400552/108392711/108383501/108402892/108386781/108404928/108404345/108404003/108386925/108384738/108398963/108394236/108386788/108395465/108394575/108398149/108400101/108404982/10	KCNA6/KCNQ4/CACNB1/CHRND/LOC108389467/KCNMB2/KCNJ4/LOC108401977/GRIN3A/HTR3A/GRIA2/LOC108390801/CACNA1D/TRPV4/TPCN2/KCNB2/GRIA3/CHRNG/CLCN1/LOC108400595/GLRB/CLCN2/KCNH3/GRIK5/CHRNA1/CACNA1A/CACNA1G/HVCN1/GRID2/CACNB3/LOC108402892/GRIN2D/TMEM266/GLRA4/TMEM38A/TPCN1/GABRR1/FXYD1/LOC108394236/KCNJ14/GRID1/GRIA1/GRIA4/TRPC3/KCNH8/ZACN/CATSPER2/KCNMB	52

				8405924/108403895/108402184/108399367/108395859/108397611/108392845	4/TMEM37/TRPC1/LOC108397611/LOC108392845	
GO:0022803	passive transmembrane transporter activity	4.46E-06	0.000344904	108385348/108403402/108383696/108383721/108389467/108406972/108399331/108401977/108386667/108402127/108409350/108390801/108402355/108392445/108385313/108402092/108390600/108383723/108404388/108400595/108393370/108395270/108408468/108393388/108395367/108399643/108402776/108400552/108392711/108383501/108402892/108386781/108404928/108404345/108404003/108386925/108384738/108398963/108394236/108386788/108395465/108394575/108398149/108400101/108404982/108405924/108403895/108402184/108399367/108395859/108397611/108392845	KCNA6/KCNQ4/CACNB1/CHRND/LOC108389467/KCNMB2/KCNJ4/LOC108401977/GRIN3A/HTR3A/GRIA2/LOC108390801/CACNA1D/TRPV4/TPCN2/KCNB2/GRIA3/CHRNG/CLCN1/LOC108400595/GLRB/CLCN2/KCNH3/GRIK5/CHRNA1/CACNA1A/CACNA1G/HVCN1/GRID2/CACNB3/LOC108402892/GRIN2D/TMEM266/GLRA4/TMEM38A/TPCN1/GABRR1/FXYD1/LOC108394236/KCNJ14/GRID1/GRIA1/GRIA4/TRPC3/KCNH8/ZACN/CATSPER2/KCNMB4/TMEM37/TRPC1/LOC108397611/LOC108392845	52
GO:0022838	substrate-specific channel activity	4.46E-06	0.000344904	108385348/108403402/108383696/108383721/108389467/108406972/108399331/108401977/108386667/108402127/108409350/108390801/108402355/108392445/108385313/108402092/108390600/108383723/108404388/108400595/108393370/108395270/108408468/108393388/108395367/108399643/108402776/108400552/108392711/108383501/108402892/108386781/108404928/108404345/108404003/108386925/108384738/108398963/108394236/108386788/108395465/108394575/108398149/108400101/108404982/10	KCNA6/KCNQ4/CACNB1/CHRND/LOC108389467/KCNMB2/KCNJ4/LOC108401977/GRIN3A/HTR3A/GRIA2/LOC108390801/CACNA1D/TRPV4/TPCN2/KCNB2/GRIA3/CHRNG/CLCN1/LOC108400595/GLRB/CLCN2/KCNH3/GRIK5/CHRNA1/CACNA1A/CACNA1G/HVCN1/GRID2/CACNB3/LOC108402892/GRIN2D/TMEM266/GLRA4/TMEM38A/TPCN1/GABRR1/FXYD1/LOC108394236/KCNJ14/GRID1/GRIA1/GRIA4/TRPC3/KCNH8/ZACN/CATSPER2/KCNMB	52

				8405924/108403895/108402184/108399367/108395859/108397611/108392845	4/TMEM37/TRPC1/LOC108397611/LOC108392845	
GO:0005234	extracellular-glutamate-gated ion channel activity	1.03E-05	0.000617786	108386667/108409350/108390600/108400595/108393388/108392711/108386781/108395465/108394575/108398149	GRIN3A/GRIA2/GRIA3/LOC108400595/GRIK5/GRID2/GRIN2D/GRID1/GRIA1/GRIA4	10
GO:0004970	ionotropic glutamate receptor activity	2.04E-05	0.000617786	108386667/108409350/108390600/108400595/108393388/108392711/108386781/108395465/108394575/108398149	GRIN3A/GRIA2/GRIA3/LOC108400595/GRIK5/GRID2/GRIN2D/GRID1/GRIA1/GRIA4	10
GO:0008066	glutamate receptor activity	2.04E-05	0.000617786	108386667/108409350/108390600/108400595/108393388/108392711/108386781/108395465/108394575/108398149	GRIN3A/GRIA2/GRIA3/LOC108400595/GRIK5/GRID2/GRIN2D/GRID1/GRIA1/GRIA4	10
GO:0022824	transmitter-gated ion channel activity	2.04E-05	0.000617786	108386667/108409350/108390600/108400595/108393388/108392711/108386781/108395465/108394575/108398149	GRIN3A/GRIA2/GRIA3/LOC108400595/GRIK5/GRID2/GRIN2D/GRID1/GRIA1/GRIA4	10
GO:0022835	transmitter-gated channel activity	2.04E-05	0.000617786	108386667/108409350/108390600/108400595/108393388/108392711/108386781/108395465/108394575/108398149	GRIN3A/GRIA2/GRIA3/LOC108400595/GRIK5/GRID2/GRIN2D/GRID1/GRIA1/GRIA4	10
GO:0030594	neurotransmitter receptor activity	2.04E-05	0.000617786	108386667/108409350/108390600/108400595/108393388/108392711/108386781/108395465/108394575/108398149	GRIN3A/GRIA2/GRIA3/LOC108400595/GRIK5/GRID2/GRIN2D/GRID1/GRIA1/GRIA4	10
GO:0022836	gated channel activity	2.20E-05	0.000617786	108383696/108383721/108406972/108399331/108401977/108386667/108402127/108409350/108402092/108390600/108383723/108404388/108400595/108393370/108395270/108393388/108395367/108392711/108383501/108386781/10840	CACNB1/CHRND/KCNMB2/KCNJ4/LOC108401977/GRIN3A/HTR3A/GRIA2/KCNB2/GRIA3/CHRNG/CLCN1/LOC108400595/GLRB/CLCN2/GRIK5/CHRNA1/GRID2/CACNB3/GRIN2D/GLRA4/GABRR1/KCNJ14/	31

				4345/108384738/108386788/108395465/108394575/108398149/108405924/108402184/108399367/108397611/108392845	GRID1/GRIA1/GRIA4/ZACN/KCNMB4/TMEM37/LOC108397611/LOC108392845	
GO:0005201	extracellular matrix structural constituent	0.000158039	0.004069495	108398405/108385124/108392530/108402997/108397135/108397652/108395489/108390086/108392454	COL1A2/COL1A1/COL11A1/COL5A1/LOC108397135/COL24A1/COL5A3/COL27A1/COL2A1	9
GO:0005230	extracellular ligand-gated ion channel activity	0.000308855	0.006062541	108383721/108386667/108402127/108409350/108390600/108383723/108400595/108393370/108393388/108395367/108392711/108386781/108404345/108384738/108395465/108394575/108398149/108405924	CHRND/GRIN3A/HTR3A/GRIA2/GRIA3/CHRNG/LOC108400595/GLRB/GRIK5/CHRNA1/GRID2/GRIN2D/GLRA4/GABRR1/GRID1/GRIA1/GRIA4/ZACN	18
GO:0015276	ligand-gated ion channel activity	0.000309166	0.006062541	108383721/108399331/108401977/108386667/108402127/108409350/108390600/108383723/108400595/108393370/108393388/108395367/108392711/108386781/108404345/108384738/108386788/108395465/108394575/108398149/108405924	CHRND/KCNJ4/LOC108401977/GRIN3A/HTR3A/GRIA2/GRIA3/CHRNG/LOC108400595/GLRB/GRIK5/CHRNA1/GRID2/GRIN2D/GLRA4/GABRR1/KCNJ14/GRID1/GRIA1/GRIA4/ZACN	21
GO:0022834	ligand-gated channel activity	0.000309166	0.006062541	108383721/108399331/108401977/108386667/108402127/108409350/108390600/108383723/108400595/108393370/108393388/108395367/108392711/108386781/108404345/108384738/108386788/108395465/108394575/108398149/108405924	CHRND/KCNJ4/LOC108401977/GRIN3A/HTR3A/GRIA2/GRIA3/CHRNG/LOC108400595/GLRB/GRIK5/CHRNA1/GRID2/GRIN2D/GLRA4/GABRR1/KCNJ14/GRID1/GRIA1/GRIA4/ZACN	21
GO:0008083	growth factor activity	0.000313918	0.006062541	108397792/108384371/108406806/108407678/108383849/108391501/108402589/108407007/108402439/108383974/108395853/108406024/108	FGF10/FIGF/MDK/BMP4/GDF3/VEGFC/LEFTY1/FGF14/FGF16/FGF18/GDF10/GDF	19

				397360/108391645/108385350/108406200/108393235/108386597/108396772	1/PDGFB/PTN/MSTN/PSPN/AMH/FGF22/INHA	
GO:0005231	excitatory extracellular ligand-gated ion channel activity	0.00043847	0.007969843	108386667/108409350/108390600/108400595/108393388/108392711/108386781/108395465/108394575/108398149	GRIN3A/GRIA2/GRIA3/LOC108400595/GRIK5/GRID2/GRIN2D/GRID1/GRIA1/GRIA4	10
GO:0022857	transmembrane transporter activity	0.001052802	0.018073102	108400017/108399423/108385348/108397070/108403402/108383696/108383721/108385897/108389467/108396530/108406972/108399331/108410003/108401977/108386667/108397281/108402127/108387521/108409350/108408139/108410246/108386217/108400617/108390801/108402355/108392445/108400903/108385313/108409832/108402092/108390600/108383723/108397882/108404388/108400595/108393370/108395270/108408468/108393388/108395367/108390354/108409591/108399643/108402776/108400552/108395992/108392711/108383501/108402892/108394129/108386781/108404861/108383479/108404928/108404345/108408258/108404003/108402411/108396743/108386925/108384738/108394404/108398963/108409670/108394236/108386788/108395465/108394575/108398149/108405144/108400101/108404982/108405924/108391843/108403895/108387375/108402184/108397058/1	SLC22A17/SLC29A4/KCNA6/SLC4A3/KCNQ4/CACNB1/CHRND/SVOP/LOC108389467/SLC7A3/KCNMB2/KCNJ4/SLC4A1/LOC108401977/GRIN3A/ABCC11/HTR3A/LOC108387521/GRIA2/ATP2B2/SLC26A11/SLC26A7/SLC6A3/LOC108390801/CACNA1D/TRPV4/ABCC6/TPCN2/ABCC5/KCNB2/GRIA3/CHRNG/ATP6V1G2/CLCN1/LOC108400595/GLRB/CLCN2/KCNH3/GRIK5/CHRNA1/SLC9A5/SFXN3/CACNA1A/CACNA1G/HVCN1/SLC1A3/GRID2/CACNB3/LOC108402892/SLC6A1/GRIN2D/ABCC8/ATP2B3/TMEM266/GLRA4/ABCB9/TMEM38A/ABCD1/LOC108396743/TPCN1/GABRR1/SIDT1/FXYD1/SLC41A3/LOC108394236/KCNJ14/GRID1/GRIA1/GRIA4/SLC2A10/TRPC3/KCNH8/ZACN/SLC4A2/CATSPER2/SLC1A4/KCNMB4/NIPA1/TMEM37/TRPC1/LOC108397611/SLC7A10/LOC108392845	83

				08399367/108395859/108397611/108384331/108392845		
GO:0022891	substrate-specific transmembrane transporter activity	0.001191927	0.019384501	108399423/108385348/108397070/108403402/108383696/108383721/108389467/108396530/108406972/108399331/108410003/108401977/108386667/108402127/108387521/108409350/108408139/108410246/108386217/108400617/108390801/108402355/108392445/108385313/108402092/108390600/108383723/108404388/108400595/108393370/108395270/108408468/108393388/108395367/108390354/108409591/108399643/108402776/108400552/108392711/108383501/108402892/108394129/108386781/108383479/108404928/108404345/108404003/108396743/108386925/108384738/108394404/108398963/108409670/108394236/108386788/108395465/108394575/108398149/108400101/108404982/108405924/108391843/108403895/108402184/108397058/108399367/108395859/108397611/108384331/108392845	SLC29A4/KCNA6/SLC4A3/KCNQ4/CACNB1/CHRND/LOC108389467/SLC7A3/KCNMB2/KCNJ4/SLC4A1/LOC108401977/GRIN3A/HTR3A/LOC108387521/GRIA2/ATP2B2/SLC26A11/SLC26A7/SLC6A3/LOC108390801/CACNA1D/TRPV4/TPCN2/KCNB2/GRIA3/CHRNG/CLCN1/LOC108400595/GLRB/CLCN2/KCNH3/GRIK5/CHRNA1/SLC9A5/SFXN3/CACNA1A/CACNA1G/HVCN1/GRID2/CACNB3/LOC108402892/SLC6A1/GRIN2D/ATP2B3/TMEM266/GLRA4/TMEM38A/LOC108396743/TPCN1/GABRR1/SIDT1/FXYD1/SLC41A3/LOC108394236/KCNJ14/GRID1/GRIA1/GRIA4/TRPC3/KCNH8/ZACN/SLC4A2/CATSPER2/KCNMB4/NIPA1/TMEM37/TRPC1/LOC108397611/SLC7A10/LOC108392845	71
GO:0015075	ion transmembrane transporter activity	0.00183006	0.028274423	108385348/108397070/108403402/108383696/108383721/108389467/108396530/108406972/108399331/108410003/108401977/108386667/108402127/108387521/108409350/108408139/108410246/108386217/108400617/108390801/108402355/108392445/108385313/108402092/108390	KCNA6/SLC4A3/KCNQ4/CACNB1/CHRND/LOC108389467/SLC7A3/KCNMB2/KCNJ4/SLC4A1/LOC108401977/GRIN3A/HTR3A/LOC108387521/GRIA2/ATP2B2/SLC26A11/SLC26A7/SLC6A3/LOC108390801/CACNA1D/TRPV4/TPCN2/KCNB2/GRIA3/C	69

				600/108383723/108404388/108400595/108393370/108395270/108408468/108393388/108395367/108390354/108409591/108399643/108402776/108400552/108392711/108383501/108402892/108394129/108386781/108383479/108404928/108404345/108404003/108396743/108386925/108384738/108398963/108409670/108394236/108386788/108395465/108394575/108398149/108400101/108404982/108405924/108391843/108403895/108402184/108397058/108399367/108395859/108397611/108384331/108392845	HRNG/CLCN1/LOC108400595/GLRB/CLCN2/KCNH3/GRIK5/CHRNA1/SLC9A5/SFXN3/CACNA1A/CACNA1G/HVCN1/GRID2/CACNB3/LOC108402892/SLC6A1/GRIN2D/ATP2B3/TMEM266/GLRA4/TMEM38A/LOC108396743/TPCN1/GABRR1/FXYD1/SLC41A3/LOC108394236/KCNJ14/GRID1/GRIA1/GRIA4/TRPC3/KCNH8/ZACN/SLC4A2/CATSPER2/KCNMB4/NIPA1/TMEM37/TRPC1/LOC108397611/SLC7A10/LOC108392845	
GO:0003774	motor activity	0.002800722	0.041210631	108401433/108389267/108392716/108407478/108406324/108400983/108404419/108390087/108384473/108400638/108391651/108385825/108390819/108408814/108389935/108384379/108385171/108410322/108393941/108410288/108409980/108408765/108401580/108388322/108386166/108397071/108384321	LOC108401433/LOC108389267/LOC108392716/CCDC102A/MYH7B/MYO15A/DNAH1/KIF12/STARD9/MYO15B/KIF7/MYO1A/MYO7A/KIF26A/LOC108389935/KIF9/LOC108385171/KIF20A/KIFC1/LOC108410288/KIF5A/LOC108408765/LOC108401580/LOC108388322/KIF19/DNAH7/LOC108384321	27
GO:0004222	metalloendopeptidase activity	0.003246242	0.045594946	108394038/108406962/108398174/108409493/108395040/108398003/108407388/108401792/108393179/108406102/108395922/108395772/108392832/108392837/108409423/108388899/108399663/108400678/108397137/108407511	MMP16/ADAMTS19/MMP23B/ADAM33/ADAMTS2/ADAMTS14/ADAM12/ADAMTS10/BMP1/MMP15/MMP19/LOC108395772/ADAMTS8/ADAMTS15/MME/ADAM7/LOC108399663/MMP27/ADAMTS16/MMP28	20

Supplementary Table 5. DEGs (embryo-hair vs adult-hair group) common to both the transcriptome and proteome

Protein ID	UP-/ DOWN-regulated in embryo-hair group	Gene ID	log2FoldChange (embryo-hair vs adult-hair group)	padj	Gene name
XP_017521509.1	down	108400568	-6.966024	1.59E-21	SOD2
XP_017499006.1	down	108385898	-11.218654	6.46E-20	LOC108385898
XP_017512162.1	up	108394841	6.33350797	8.86E-16	CD248
XP_017508327.1	up	108392530	8.0968247	3.04E-15	COL11A1
XP_017502735.1	up	108388713	8.09460453	9.30E-14	LOC108388713
XP_017518346.1	down	108398673	-7.5896038	1.07E-11	SERPINA12
XP_017505592.1	up	108390824	5.13387687	1.20E-08	POSTN
XP_017503284.1	up	108389267	9.07848444	1.14E-07	LOC108389267
XP_017516313.1	up	108397414	5.45619783	1.31E-06	CRABP1
XP_017495741.1	down	108383655	-4.2522687	3.74E-06	CDSN
XP_017502029.1	up	108388123	3.28907093	0.00112387	CD34
XP_017498905.1	up	108385829	2.59839973	0.0013268	GPX7
XP_017520965.1	up	108400234	3.08496061	0.00152631	LOC108400234
XP_017519712.1	up	108399435	2.30890145	0.00177738	GAMT
XP_017502194.1	down	108388266	-4.3658774	0.001819	FCGR2B
XP_017535294.1	up	108409314	2.70720837	0.00203977	THY1
XP_017536061.1	up	108409816	3.47768837	0.00216028	CHGA
XP_017512788.1	up	108395215	2.97180512	0.00303384	GLT8D2
XP_017513844.1	up	108395796	1.87899122	0.00358708	POFUT2
XP_017496674.1	down	108384291	-3.0458426	0.00404898	SERPINE2
XP_017509691.1	up	108393437	4.05297516	0.0057978	LOC108393437
XP_017517270.1	up	108398029	-2.1676016	0.00904686	DDX21
XP_017499696.1	down	108386371	-2.4898577	0.01126826	S100A9
XP_017498833.1	down	108385785	-2.8859213	0.01131029	SERPINB10
XP_017522635.1	down	108401294	-2.5669346	0.01315755	SERPINB1
XP_017504458.1	up	108390221	-3.297147	0.01508195	PECAM1
XP_017506968.1	up	108391716	1.99256308	0.01553987	ANXA6
XP_017499673.1	down	108386363	-2.6949742	0.01729007	S100A12
XP_017517694.1	up	108398314	-2.1879358	0.02622472	HSPA5
XP_017530308.1	up	108406227	-2.2392883	0.03526084	PDK4
XP_017532764.1	up	108407730	-1.5448364	0.03862281	PTBP3
XP_017501173.1	up	108387424	-1.3190941	0.04667409	ABCE1
XP_017512203.1	down	108394871	-1.781936	0.04840318	DSC1
XP_017532291.1	down	108407438	-2.4589578	0.05803667	CSTA

Supplementary Table 6. DEGs (embryo-scale vs. adult-scale group) common to both the transcriptome and proteome.

Protein ID	UP-/DOWN-regulated in embryo-scale group	Gene ID	log2FoldChange(embryo-scale vs. adult-scale group)	padj	Gene name
XP_017513209.1	up	108395433	5.24496759	1.08E-10	COL21A1
XP_017509555.1	up	108393323	11.5983024	2.69E-10	LOC108393323
XP_017517849.1	up	108398388	4.51993605	1.77E-06	ELN
XP_017506867.1	up	108391665	7.08565145	2.26E-06	KERA
XP_017516313.1	up	108397414	7.20488382	1.52E-05	CRABP1
XP_017512535.1	up	108395057	5.46804255	0.00016667	GNMT
XP_017526319.1	up	108403604	6.51144694	0.00032971	LOC108403604
XP_017515805.1	up	108397091	3.07568529	0.00038596	COL16A1
XP_017520914.1	up	108400195	-2.6232486	0.00176417	MAP1LC3B
XP_017533455.1	up	108408165	-2.9267555	0.00737728	RAB21
XP_017506953.1	up	108391727	5.02876673	0.00755271	LOC108391727
XP_017512463.1	up	108395012	2.35361236	0.00880083	TBCD
XP_017524748.1	up	108402612	2.54050279	0.01369294	IGF2
XP_017507985.1	up	108392338	-2.007472	0.01532875	ACTR2
XP_017507425.1	up	108392007	-1.9158616	0.01836743	PTGES3
XP_017514340.1	up	108396117	-1.9674562	0.03167314	M6PR
XP_017504095.1	up	108390026	-1.9798241	0.0499269	MAPRE1

Supplementary Table 7. DEGs between adult-hair and adult-scale (adult-hair vs adult-scale group)

Gene ID	log2FoldChange (MJH-vs MJS-group)	padj	Gene name	Gene biotype
108403509	-8.85817039	1.01E-12	IGFBP1	protein_coding
108390635	-7.18914713	1.01E-12	FITM1	protein_coding
108383880	-6.13766555	3.26E-12	CDH2	protein_coding
108394021	-5.24552008	3.26E-12	DUSP26	protein_coding
108398764	-6.1599075	4.51E-12	LOC108398764	protein_coding
108385125	-3.99221883	4.51E-12	SGCA	protein_coding
108395637	-5.5029254	4.53E-12	DTNA	protein_coding
108405476	-6.1465675	4.82E-11	LOC108405476	protein_coding
108395367	-8.14808654	6.76E-11	CHRNA1	protein_coding
108396505	-5.75829849	9.24E-11	CRYAB	protein_coding
novel.1822	-8.412147	1.57E-10	-	-
novel.2845	-8.89796553	4.26E-10	-	-
108386302	-7.03423953	4.34E-10	KLHL30	protein_coding
108397059	-9.50871363	4.34E-10	EYA4	protein_coding
108388701	-5.22801955	6.95E-10	WIPF3	protein_coding
108398347	-5.84009577	7.55E-10	CDH15	protein_coding
novel.8371	-4.92140168	7.55E-10	-	-
108406640	-4.70657649	7.55E-10	CORO6	protein_coding
108401165	-6.93932744	1.10E-09	SCN4A	protein_coding
novel.4789	-4.73172446	2.34E-09	-	-
108392169	-6.58493021	4.34E-09	FLNC	protein_coding
108398008	-2.97863249	4.85E-09	PCBD1	protein_coding
108406345	-4.40247863	5.51E-09	ARPP21	protein_coding
108407340	-3.88703466	5.64E-09	TBX15	protein_coding
108399997	-5.47875111	8.25E-09	ART5	protein_coding
108387025	-7.00626634	9.34E-09	LOC108387025	protein_coding
108400415	-5.62846693	1.11E-08	TXLNB	protein_coding
108397159	-5.40989129	1.16E-08	SPTB	protein_coding
108394386	-3.95991169	1.18E-08	PLPP7	protein_coding
108410273	-5.75495524	1.26E-08	SIX1	protein_coding
novel.7867	-6.13392118	1.33E-08	-	-
108392089	-4.58317565	1.40E-08	DMPK	protein_coding
108406917	-5.24007642	2.01E-08	CX3CR1	protein_coding
108407937	-4.37637727	2.55E-08	FBXO10	protein_coding
108404863	-6.71748753	3.81E-08	KCNJ11	protein_coding
108404304	-6.30801405	4.76E-08	MURC	protein_coding
108384506	-5.7592248	4.76E-08	BVES	protein_coding
108403601	-5.81575846	4.76E-08	MYO18B	protein_coding
108409628	-5.62765642	5.81E-08	FBXO32	protein_coding
108383367	-6.0525856	6.35E-08	LOC108383367	protein_coding
108399995	-6.22835352	6.51E-08	ART1	protein_coding

108405270	-4.98624756	6.85E-08	LOC108405270	protein_coding
108399498	-5.10965954	6.85E-08	LOC108399498	protein_coding
108399878	-4.4744571	7.06E-08	ACACB	protein_coding
108397377	-5.44415881	7.06E-08	ETNPPL	protein_coding
108386693	-4.18131133	7.09E-08	SESN1	protein_coding
108399800	-2.50237327	7.58E-08	VDAC3	protein_coding
108392811	-4.96530604	7.58E-08	FAT3	protein_coding
108393126	-8.38247185	8.38E-08	LOC108393126	protein_coding
108405414	-4.32497964	9.41E-08	SHISA4	protein_coding
108386982	-4.10438717	1.02E-07	CTH	protein_coding
108391030	-4.36491879	1.11E-07	FGF1	protein_coding
108410063	-6.79838757	1.12E-07	STOML3	protein_coding
108386637	-6.15847026	1.12E-07	CAPN3	protein_coding
108387731	-4.39924891	1.34E-07	AMPD3	protein_coding
108384375	-5.54951914	1.34E-07	ART3	protein_coding
novel.1408	-6.88173084	1.36E-07	-	-
108410160	-4.706626	1.46E-07	SLC25A4	protein_coding
novel.450	-6.2944883	1.46E-07	-	-
108383730	-3.11896013	1.55E-07	RAPSN	protein_coding
108406225	-6.89300759	1.55E-07	PON1	protein_coding
108397235	-3.23580695	1.87E-07	S100A1	protein_coding
novel.5775	-4.76016629	2.20E-07	-	-
108389888	-5.11365081	2.48E-07	CAND2	protein_coding
108398929	1.932054515	2.49E-07	MSX1	protein_coding
108398735	-4.08553145	2.68E-07	SNX8	protein_coding
108384643	-4.03935118	2.87E-07	LPIN1	protein_coding
novel.6341	-7.20702909	2.93E-07	-	-
108399956	-6.04045749	2.94E-07	LOC108399956	protein_coding
108405137	-4.35420016	3.00E-07	MAP2K6	protein_coding
108402058	-3.86800007	3.00E-07	CFL2	protein_coding
novel.3328	-3.58034113	3.19E-07	-	-
108403285	-4.73917421	3.20E-07	SYNM	protein_coding
108398776	-5.36314667	3.24E-07	LOC108398776	protein_coding
108390533	-4.61545294	3.52E-07	STRIP2	protein_coding
108397849	-3.30696983	3.61E-07	KIFAP3	protein_coding
108393015	-5.70481442	3.64E-07	DNAJC22	protein_coding
novel.7860	-4.83330366	3.71E-07	-	-
108390120	-3.88729031	3.85E-07	GATSL2	protein_coding
108390751	-4.27376718	4.03E-07	LOC108390751	protein_coding
novel.3880	-8.41991658	4.19E-07	-	-
108392701	-4.43324548	4.37E-07	APOBEC2	protein_coding
108384271	-4.40543726	4.37E-07	LOC108384271	lncRNA
108394543	-4.87396976	4.46E-07	SLC43A2	protein_coding
108405763	-4.81354655	4.50E-07	HRASLS	protein_coding

108396218	-4.23210013	4.50E-07	COQ8A	protein_coding
108397658	-5.06729646	4.93E-07	LOC108397658	protein_coding
108408501	-7.9500848	5.33E-07	LBX1	protein_coding
108403949	-6.1304955	5.78E-07	MYOZ2	protein_coding
novel.2468	-5.9458017	5.91E-07	-	-
novel.6214	-4.91322405	5.91E-07	-	-
108400323	-2.92216636	6.79E-07	HRC	protein_coding
108396286	-3.67197085	6.86E-07	FHL1	protein_coding
108394115	-3.73834031	6.87E-07	GPAT3	protein_coding
108409032	-3.44966381	7.35E-07	CLIC5	protein_coding
novel.5128	-5.60314692	7.37E-07	-	-
108407392	-6.02395741	7.98E-07	MRLN	protein_coding
108400093	-5.30453238	8.92E-07	TMEM155	protein_coding
108394344	-2.54655519	8.98E-07	ABCB6	protein_coding
108397094	-6.38412855	8.98E-07	HCRTR1	protein_coding
108393245	-6.34585262	8.99E-07	JSRP1	protein_coding
novel.8306	-6.26733786	9.79E-07	-	-
108400389	-4.65961041	1.05E-06	ITGA10	protein_coding
108386884	-3.00813147	1.08E-06	RTN2	protein_coding
108396331	-5.98377412	1.12E-06	TNNT1	protein_coding
108394334	4.437416016	1.12E-06	LOC108394334	lncRNA
108401866	2.677735011	1.42E-06	CA2	protein_coding
novel.2029	-5.8073454	1.67E-06	-	-
108387963	-5.44778038	1.69E-06	GRAMD1B	protein_coding
108393093	-6.68249189	1.72E-06	MYLK2	protein_coding
108404359	-2.80541258	1.80E-06	GSTA4	protein_coding
108390001	-7.7521461	1.80E-06	PNMT	protein_coding
108394427	-4.8494875	1.85E-06	TMOD1	protein_coding
novel.8773	-3.83812772	1.90E-06	-	-
novel.2778	-7.72186123	2.10E-06	-	-
108410271	-3.8129854	2.16E-06	SIX4	protein_coding
108391635	-3.1136266	2.31E-06	EPM2A	protein_coding
108409998	-5.99930465	2.33E-06	LOC108409998	protein_coding
108384557	-4.36564361	2.45E-06	LOC108384557	lncRNA
novel.8123	-4.96062645	2.47E-06	-	-
108407550	-5.13747072	2.55E-06	MYPN	protein_coding
108405387	2.669477146	3.18E-06	NPNT	protein_coding
108391850	-3.83553567	3.32E-06	ASB10	protein_coding
novel.3280	-7.16654528	3.39E-06	-	-
108389836	-4.04379123	3.41E-06	RBM38	protein_coding
108384735	-3.29470395	3.52E-06	PM20D2	protein_coding
108404409	-3.09497565	3.71E-06	DNAJB5	protein_coding
108395397	2.844373113	3.71E-06	LOC108395397	lncRNA
108402808	-5.97476354	3.74E-06	ASB5	protein_coding

108390965	2.96328948	3.74E-06	TENM2	protein_coding
108397268	-6.89707072	3.79E-06	CMYA5	protein_coding
108398767	-5.702381	3.94E-06	SYNPO2L	protein_coding
novel.4548	-7.7449254	3.98E-06	-	-
108386337	-4.10308987	3.99E-06	LOC108386337	lncRNA
108401670	-8.15391129	3.99E-06	MYOG	protein_coding
novel.2595	-4.15649589	4.10E-06	-	-
108403512	-4.71734027	4.66E-06	ROBO2	protein_coding
108395512	-3.32802836	4.98E-06	ENHO	protein_coding
108387771	3.142484377	4.98E-06	LOC108387771	protein_coding
108404600	-6.66834553	5.14E-06	PTPRQ	protein_coding
108388252	-5.17119281	5.19E-06	LOC108388252	protein_coding
108387246	-5.77475798	5.42E-06	FSD2	protein_coding
108386124	-4.22617662	5.49E-06	GPRC5C	protein_coding
108385121	-2.95100962	5.49E-06	PDK2	protein_coding
108396215	-2.90958664	5.69E-06	PSEN2	protein_coding
108391033	-5.19304511	6.10E-06	LRRC2	protein_coding
108388172	-3.50009849	6.32E-06	LOC108388172	protein_coding
108401509	-5.05982061	7.19E-06	HSPB6	protein_coding
108384367	-6.53313937	7.37E-06	NRAP	protein_coding
108400756	-4.68028119	7.37E-06	USP13	protein_coding
108409192	-4.67373139	7.40E-06	PLCL2	protein_coding
108394207	-5.92588397	7.40E-06	KBTBD12	protein_coding
108383506	-7.07199703	7.40E-06	RBM24	protein_coding
108388099	-7.41605894	8.37E-06	EYA1	protein_coding
108407707	-3.75997128	8.37E-06	KLF15	protein_coding
108409792	-3.0243194	8.90E-06	SLC43A1	protein_coding
108394548	-2.63548383	9.29E-06	RPH3AL	protein_coding
108387691	-3.83068624	9.55E-06	ZNF500	protein_coding
108393106	-3.59966207	9.64E-06	INSIG1	protein_coding
novel.1390	-3.85869332	1.01E-05	-	-
108402131	-3.12662672	1.02E-05	USP28	protein_coding
108406485	-3.27928187	1.04E-05	CHRNB1	protein_coding
novel.5266	-7.9254434	1.13E-05	-	-
108404428	-6.14908923	1.16E-05	TNNC1	protein_coding
108403218	-2.97708281	1.17E-05	PARVB	protein_coding
novel.6528	-6.59376083	1.19E-05	-	-
108386416	-2.25653536	1.28E-05	PIK3IP1	protein_coding
novel.8010	-6.22247017	1.33E-05	-	-
108396504	-3.40122934	1.40E-05	HSPB2	protein_coding
108391423	-4.83909977	1.46E-05	LOC108391423	protein_coding
108407910	-5.12881711	1.46E-05	DDIT4L	protein_coding
novel.5425	-7.24747672	1.52E-05	-	-
108397688	-2.68410595	1.52E-05	ACADS	protein_coding

novel.2775	-4.49652744	1.53E-05	-	-
novel.6405	-4.83341135	1.58E-05	-	-
108403008	-4.811697	1.58E-05	PFKM	protein_coding
novel.5386	-7.68557937	1.63E-05	-	-
108400088	-3.88185258	1.74E-05	NEXN	protein_coding
novel.5538	-7.38106385	1.79E-05	-	-
108401681	-2.71648774	2.01E-05	RILPL1	protein_coding
108389779	-2.47912424	2.04E-05	LOC108389779	protein_coding
108409025	-4.67387029	2.04E-05	LOC108409025	lncRNA
108397416	-3.01147505	2.15E-05	DNAJA4	protein_coding
108388558	4.572602503	2.16E-05	LOC108388558	protein_coding
108403648	-5.72554835	2.18E-05	LOC108403648	protein_coding
novel.7975	-3.02225003	2.22E-05	-	-
108388330	-4.28750637	2.24E-05	LOC108388330	protein_coding
108403632	-2.98354621	2.24E-05	LOC108403632	protein_coding
108398853	-3.31878262	2.31E-05	ACYP2	protein_coding
108396108	-5.28288513	2.34E-05	DES	protein_coding
108389926	2.586521936	2.40E-05	LOC108389926	protein_coding
108383696	-3.07911968	2.52E-05	CACNB1	protein_coding
108402310	-5.92910976	2.59E-05	SMTNL1	protein_coding
108404516	-2.81435625	2.62E-05	DYRK1B	protein_coding
novel.480	-5.88574497	2.77E-05	-	-
novel.5494	-2.37442509	2.77E-05	-	-
108392319	-4.42962308	2.77E-05	LOC108392319	lncRNA
108402571	-3.86101729	2.93E-05	GYG2	protein_coding
108404865	-4.58670926	3.18E-05	USH1C	protein_coding
108398581	-1.9179246	3.18E-05	SOD1	protein_coding
108391716	-2.52284644	3.28E-05	ANXA6	protein_coding
novel.5728	-6.52918074	3.43E-05	-	-
108400198	-4.26408226	3.45E-05	LOC108400198	lncRNA
108398647	-3.78489657	3.50E-05	MAPT	protein_coding
108399159	-2.22419486	3.66E-05	ADRM1	protein_coding
novel.7494	-7.5910774	3.69E-05	-	-
108383824	-7.99171153	3.70E-05	LHX8	protein_coding
novel.7199	-5.99369996	3.70E-05	-	-
108396363	-6.71835073	3.70E-05	ATP1B4	protein_coding
108390904	-2.37380682	3.70E-05	LOC108390904	protein_coding
108389843	2.994825716	3.72E-05	LOC108389843	protein_coding
108394676	2.478603584	3.82E-05	CDH3	protein_coding
108393308	-4.80883456	3.92E-05	DUSP13	protein_coding
novel.3815	-6.12656008	3.93E-05	-	-
novel.8804	-3.73300426	4.26E-05	-	-
108386683	-6.19054785	4.26E-05	ABRA	protein_coding
108392327	-3.28875446	4.53E-05	GMPR	protein_coding

108404215	-2.86951424	4.60E-05	PPP1R3F	protein_coding
108399296	-2.59981276	4.64E-05	PAX8	protein_coding
108406145	-3.33080589	4.68E-05	SYNPO	protein_coding
novel.5400	-5.64003554	4.74E-05	-	-
108404146	2.684446373	4.87E-05	LOC108404146	protein_coding
novel.882	4.952074797	4.87E-05	-	-
108399199	-6.12727961	5.02E-05	LOC108399199	lncRNA
108399381	3.761060481	5.03E-05	LOC108399381	protein_coding
108387386	3.214259633	5.63E-05	IRX4	protein_coding
108404892	-3.56633437	5.68E-05	GADD45A	protein_coding
108397975	-2.76130606	6.12E-05	ST6GALNAC6	protein_coding
novel.5186	-4.0941815	6.12E-05	-	-
108399955	-5.66658965	6.12E-05	ADPRHL1	protein_coding
novel.6292	-5.21826915	6.12E-05	-	-
108406634	-3.57982438	6.15E-05	SSH2	protein_coding
108395234	-3.69174822	6.17E-05	PPP1R27	protein_coding
108404580	3.293958611	6.30E-05	LOC108404580	protein_coding
108386353	-2.94780314	6.31E-05	PPM1J	protein_coding
108404596	-7.546398	6.58E-05	MYF5	protein_coding
108407336	-2.63491823	6.59E-05	EIF4E3	protein_coding
108391154	-4.15904677	6.81E-05	LOC108391154	lncRNA
108389868	-5.4884332	7.14E-05	LOC108389868	protein_coding
108400043	-3.0845479	7.20E-05	MAP1A	protein_coding
108399994	-5.25177466	7.42E-05	CHRNA10	protein_coding
108404795	-3.62175526	7.42E-05	ABLIM2	protein_coding
novel.4900	-5.96261824	7.98E-05	-	-
108400692	-5.53052412	8.02E-05	TRIM72	protein_coding
108391749	-2.32470632	8.34E-05	LOC108391749	protein_coding
108405111	-3.57517639	8.35E-05	PROB1	protein_coding
108393690	-2.38554937	8.78E-05	LMBR1	protein_coding
novel.1886	-6.29284488	9.08E-05	-	-
108384226	-6.68009912	9.14E-05	CUNH2orf88	protein_coding
108407197	-1.97098893	9.22E-05	CRAT	protein_coding
108409141	-2.20830807	9.55E-05	EIF4EBP1	protein_coding
novel.6076	-5.91149591	0.000100498	-	-
novel.1000	-3.75695459	0.000100725	-	-
108386301	-4.00105625	0.000106375	ESPNL	protein_coding
108390648	-3.61253805	0.000106375	LOC108390648	lncRNA
108402624	-3.40522425	0.000110175	TIGD3	protein_coding
108408464	4.128597505	0.000114952	LOC108408464	lncRNA
108406553	-2.24373498	0.000114952	HOXD9	protein_coding
108406064	-6.31953683	0.0001198	LOC108406064	protein_coding
108407391	-3.0564271	0.000121769	CCDC6	protein_coding
108385720	-2.8813698	0.000121769	AGTRAP	protein_coding

novel.5531	2.316765923	0.000121769	-	-
novel.6727	-3.21429933	0.000123278	-	-
108403980	-2.70162111	0.000128482	ECHDC2	protein_coding
108386776	-3.55571332	0.000133266	LOXL4	protein_coding
novel.8144	-2.82291259	0.000138341	-	-
108392348	3.002761535	0.000138341	LOC108392348	protein_coding
108396508	-5.30257429	0.000138378	LOC108396508	lncRNA
novel.4777	-5.90652813	0.000138616	-	-
108405193	-3.56472493	0.000144066	HIPK2	protein_coding
novel.3616	-7.04919639	0.000144543	-	-
108408150	-3.64702353	0.000145376	LOC108408150	protein_coding
108391194	-2.98880552	0.000146561	TRIM54	protein_coding
108405497	-2.65100684	0.000146561	DIP2C	protein_coding
108397575	-2.89575727	0.000148047	ATP6V0E2	protein_coding
novel.5806	-5.20588258	0.000148119	-	-
108399500	-2.6498861	0.000148599	LOC108399500	protein_coding
novel.6773	-5.64335024	0.000150051	-	-
novel.2370	-6.45383977	0.000152969	-	-
novel.4405	-3.60303544	0.000153081	-	-
108402582	-4.3834753	0.000153446	LOC108402582	lncRNA
108399613	-2.81250035	0.000153446	BIN1	protein_coding
108408132	-2.36729471	0.000153803	CRELD1	protein_coding
108387667	-2.77105532	0.000166634	OLFM1	protein_coding
108390009	-4.43927342	0.000166634	TCAP	protein_coding
108387484	-3.05710514	0.000172439	SLC16A10	protein_coding
108393326	6.087368026	0.000175746	LOC108393326	protein_coding
novel.5893	-6.02532865	0.000177123	-	-
108393005	-5.78313396	0.000181591	SLC9A2	protein_coding
novel.8165	-3.74522932	0.0001891	-	-
novel.4391	-6.27532433	0.000196274	-	-
108393400	-5.45733688	0.000196936	TNNT3	protein_coding
108384179	-6.71086132	0.000198293	ASB15	protein_coding
novel.7198	-5.43458621	0.000200119	-	-
108398024	-4.59614137	0.000200185	LOC108398024	protein_coding
108390047	2.578572413	0.00020253	RASSF9	protein_coding
108387774	3.637907373	0.00020253	IL22RA1	protein_coding
108402804	-2.81600275	0.000205228	LOC108402804	lncRNA
108395737	-4.74438469	0.000212423	CACNG6	protein_coding
novel.5969	-7.1453883	0.000212423	-	-
108388523	-4.18352217	0.000216772	ARNT2	protein_coding
108398702	-2.24115804	0.000219753	CUL4A	protein_coding
108396706	-7.18706921	0.000219753	LOC108396706	protein_coding
108386093	-3.20827828	0.000220112	LOC108386093	protein_coding
108383556	-2.31514621	0.000233836	KEAP1	protein_coding

108387862	-2.3777268	0.000236244	TYSND1	protein_coding
108405309	-2.41399077	0.000243008	HTATIP2	protein_coding
108385934	-4.14242923	0.000252258	LOC108385934	protein_coding
108387577	-2.43623841	0.000256355	ATP10A	protein_coding
108396001	-5.47888655	0.000257062	CACNA1S	protein_coding
108388681	-6.50219189	0.000266042	SBK3	protein_coding
108386500	-3.55586694	0.000268864	OSGIN1	protein_coding
108384774	-2.3415958	0.000274075	ZFR2	protein_coding
108398780	-3.58908786	0.000278165	PDE4D	protein_coding
108406863	-3.13042045	0.000282312	CLTCL1	protein_coding
108398708	-2.17743938	0.000283845	HDAC4	protein_coding
108408541	-7.06995313	0.000284194	LOC108408541	pseudogene
108390322	-2.08767766	0.000284194	NOL3	protein_coding
novel.8814	-5.17047776	0.000284194	-	-
108394413	-2.43448727	0.000287802	ATG9A	protein_coding
108409556	3.264096025	0.000291669	ZBED2	protein_coding
108401639	-2.04031164	0.000291669	LOC108401639	protein_coding
108408944	-5.81699765	0.000309194	LOC108408944	protein_coding
108389528	-6.59822784	0.000310127	LOC108389528	protein_coding
novel.5102	-3.97461895	0.000310577	-	-
novel.6038	-5.05187887	0.000311771	-	-
108402511	-2.68699743	0.000319133	SGCG	protein_coding
108407229	-2.48408471	0.000334205	GKAP1	protein_coding
108404866	-4.64236024	0.000334205	KCNC1	protein_coding
108396429	-2.0328267	0.00033678	TPRA1	protein_coding
108394661	-3.20828213	0.000349096	ZFAND2A	protein_coding
108392381	3.353743447	0.000358779	VWCE	protein_coding
108386909	-3.97400162	0.000369502	LOC108386909	protein_coding
108404578	-6.98885383	0.000377634	SMYD1	protein_coding
108397573	-4.92636308	0.000380675	RBFOX1	protein_coding
108402208	-2.04423541	0.000380675	TSPAN17	protein_coding
novel.7016	-4.47585764	0.000384295	-	-
108401212	-6.66461812	0.000393144	NT5C1A	protein_coding
108398841	-5.02213387	0.000393144	KLHL6	protein_coding
novel.5867	-4.22170136	0.000401596	-	-
novel.679	-5.16498526	0.000408246	-	-
108397761	-2.3085205	0.000418614	LOC108397761	lncRNA
108398531	-3.75126465	0.000432245	B3GALT1	protein_coding
novel.5864	-6.32297772	0.000433501	-	-
108400482	-2.28327656	0.000435297	SLC15A4	protein_coding
108400825	-1.99501592	0.000439541	SLC10A3	protein_coding
108394817	-2.10173487	0.000454238	EML1	protein_coding
novel.1011	-6.6308409	0.000454238	-	-
108403071	-2.00666392	0.000454238	MITF	protein_coding

novel.5611	-4.39848406	0.000455907	-	-
108407826	2.07265408	0.000469621	CA9	protein_coding
108383617	-1.91600483	0.000469621	PLEKHO1	protein_coding
108395817	-4.87693223	0.000473062	ST8SIA2	protein_coding
108406879	-2.76332405	0.000477914	TANGO2	protein_coding
novel.2100	2.27965992	0.000478213	-	-
108407876	-5.35109719	0.000503932	CPT1B	protein_coding
108408683	-2.91919481	0.000503932	CDKN1C	protein_coding
108386869	-2.60832041	0.00050465	CUNH16orf96	protein_coding
108405982	1.722249137	0.000517805	LOC108405982	protein_coding
108384080	-4.99485629	0.000532396	LOC108384080	lncRNA
108409626	-6.34630294	0.000536904	LOC108409626	pseudogene
108408419	-1.6873601	0.000537655	CCDC86	protein_coding
108409631	-4.39699907	0.000542467	ANXA13	protein_coding
108401336	-2.2122308	0.000543236	ACTN3	protein_coding
novel.1541	4.358290507	0.000543236	-	-
novel.593	-5.02705344	0.000543236	-	-
novel.1152	-2.43287905	0.000543236	-	-
108407481	3.997610159	0.000543236	TNIP3	protein_coding
108407606	1.907444732	0.000559143	LOC108407606	protein_coding
108403027	-2.41443064	0.00057273	ZXDC	protein_coding
108407839	-2.13941779	0.000585872	NFE2L1	protein_coding
108390976	-4.11516158	0.000588831	CD2	protein_coding
108395941	2.521244507	0.000590997	E2F7	protein_coding
108402253	-3.85351962	0.000590997	DYSF	protein_coding
108402873	-2.77578721	0.000595835	SLC38A3	protein_coding
108402307	-2.020695	0.000603756	LOC108402307	protein_coding
novel.7861	-4.90837091	0.000667132	-	-
108409552	-1.91890708	0.000673456	SIK2	protein_coding
108407785	-6.45725575	0.00067353	KCNT1	protein_coding
novel.2892	-3.60993208	0.00067353	-	-
novel.2670	-3.65203678	0.000674478	-	-
108404926	-4.14266916	0.000686218	SVIL	protein_coding
108399888	-2.32378753	0.000690767	FRAT1	protein_coding
108407764	-2.55346177	0.000697536	FTH1	protein_coding
108383529	-1.42062048	0.000705522	VIMP	protein_coding
novel.3001	-3.20852308	0.000705522	-	-
108384569	-6.61153008	0.000705522	HHATL	protein_coding
108406223	-7.33555862	0.000709146	ASB4	protein_coding
108388274	-2.57156679	0.000709146	P2RY2	protein_coding
108406256	-6.88264594	0.00072032	YIPF7	protein_coding
108399390	-6.43409117	0.000724353	MAP3K7CL	protein_coding
108394035	-3.98195582	0.000724353	LOC108394035	protein_coding
108397950	-7.31464631	0.000754054	TTN	protein_coding

108386314	-4.49912814	0.000754054	CCDC27	protein_coding
novel.4945	-3.72105026	0.000762971	-	-
108388334	-6.37719096	0.000765143	HCRT	protein_coding
108400992	-2.3514166	0.000765143	AMIGO1	protein_coding
108384500	2.677758326	0.000765143	CAMK1G	protein_coding
108388744	-3.36791464	0.000772133	TULP1	protein_coding
novel.7102	-5.4798264	0.0007755	-	-
108398751	-4.74650262	0.000781319	MYOZ1	protein_coding
108405619	-3.01388733	0.000781319	FAM13A	protein_coding
108390124	-2.60736204	0.000782323	SNTA1	protein_coding
novel.4813	-3.5837465	0.000788423	-	-
108406443	2.911187143	0.000789918	RASSF6	protein_coding
108408976	-5.30883203	0.00079828	P2RX6	protein_coding
108398110	2.816974123	0.00079828	CAPS2	protein_coding
108398530	-2.81845763	0.000799233	LOC108398530	protein_coding
108407625	-4.28339965	0.000809654	TPM2	protein_coding
108390055	-2.1120708	0.000817899	CGRRF1	protein_coding
108396139	-2.43303428	0.000834373	PHKB	protein_coding
108396899	-1.8700148	0.000834373	TACC2	protein_coding
108384566	-2.29427698	0.000835995	ZBTB47	protein_coding
108406641	-3.27758326	0.000838325	ABHD15	protein_coding
108397802	-2.28944946	0.000846841	TTC34	protein_coding
108404867	-6.30935428	0.000851879	MYOD1	protein_coding
108409473	2.015415982	0.00085689	SLC16A14	protein_coding
novel.8713	-4.46232383	0.000861455	-	-
108404987	2.225914278	0.000874195	DST	protein_coding
108408505	-4.80863374	0.000876849	PPP1R1A	protein_coding
108396193	-2.605383	0.000876849	LOC108396193	protein_coding
108405974	2.141722155	0.000882229	KLF5	protein_coding
108410180	-6.77726507	0.000890626	LOC108410180	protein_coding
novel.1481	-9.50368901	0.000890626	-	-
novel.8138	-3.90530661	0.00089796	-	-
108403654	-3.93601858	0.000901827	CRHR2	protein_coding
108403887	-6.22095549	0.000906344	ZIC1	protein_coding
108400238	-6.69774213	0.000906344	CALCR	protein_coding
108389972	1.431000327	0.000907074	MCTP2	protein_coding
108399881	-2.17859809	0.000942078	RRP12	protein_coding
108388597	-2.9769262	0.000942078	GYG1	protein_coding
108386060	-2.67632072	0.000942078	ATP6V0A1	protein_coding
108391572	-4.97894279	0.000942378	AQP4	protein_coding
108387374	-3.85084398	0.00096237	CEP68	protein_coding
108402395	-4.05255927	0.001009034	MYOM1	protein_coding
108405900	-2.6695235	0.001029672	ATOH8	protein_coding
novel.829	-5.81035277	0.001035935	-	-

novel.8553	-2.3445956	0.001040886	-	-
108385421	-2.87562588	0.001042023	ZFAND5	protein_coding
108406788	1.09580692	0.001042023	YEATS2	protein_coding
novel.4485	-1.5353679	0.001042023	-	-
108387314	6.227001801	0.001042023	LOC108387314	lncRNA
108397204	-2.38942978	0.001042023	PNMA1	protein_coding
novel.6030	-6.06196062	0.001042023	-	-
novel.1592	-6.40600696	0.001042023	-	-
108385304	-3.859583	0.001053026	GPR137C	protein_coding
108386348	2.249212391	0.001058139	LOC108386348	protein_coding
108393070	-2.91969221	0.001079068	HEY2	protein_coding
108390213	-2.42334347	0.00108177	ME3	protein_coding
novel.5247	2.906784219	0.001110219	-	-
108390058	-5.20222467	0.0011248	LOC108390058	lncRNA
108388844	-2.30720748	0.0011248	LOC108388844	protein_coding
108393837	-3.92479948	0.001157252	GADD45G	protein_coding
108402088	3.738372639	0.001157366	ARG1	protein_coding
108397357	-4.69633016	0.001158625	LOC108397357	lncRNA
108397193	2.790589375	0.001158749	COL17A1	protein_coding
108400633	1.772193945	0.001193918	GPC2	protein_coding
108394106	-3.14902902	0.001204529	LRRN2	protein_coding
108383473	1.941113797	0.001232532	FAM110C	protein_coding
108386313	-2.31748534	0.001242759	LRRC47	protein_coding
novel.2044	-6.63719868	0.001247415	-	-
novel.3215	-3.90930403	0.001264391	-	-
108406100	2.141021547	0.001265845	COL4A5	protein_coding
108405653	-2.08138228	0.001304873	NATD1	protein_coding
108402625	-3.34142503	0.001328369	SLC25A45	protein_coding
novel.8502	-2.13423555	0.001345352	-	-
108402048	-2.53618017	0.001346105	LOC108402048	protein_coding
novel.5658	-6.55173153	0.001346105	-	-
108399718	-6.93508751	0.001358181	RIMBP2	protein_coding
108390936	-3.46571447	0.001366917	PCK1	protein_coding
108385453	-5.3810243	0.001366917	LRRC30	protein_coding
108393455	-5.14409972	0.00137218	PAX3	protein_coding
novel.2908	-1.75702504	0.001373034	-	-
108390132	-2.49707224	0.001380024	ETV7	protein_coding
108405400	2.339711197	0.00139775	DDIAS	protein_coding
novel.7265	-2.44004603	0.001397753	-	-
108406465	-1.67492554	0.001413543	DNAJA3	protein_coding
novel.1068	-4.69508347	0.001413629	-	-
108409073	-7.00677799	0.001419726	LOC108409073	protein_coding
108407924	-1.86938181	0.001419726	PKDCC	protein_coding
108395642	1.64491513	0.001436651	PLEKHA5	protein_coding

108393367	-2.27683874	0.001436651	SIK3	protein_coding
108387629	-3.65647214	0.001439482	LOC108387629	lncRNA
108399928	-4.02351695	0.001439482	KCNC4	protein_coding
novel.5185	-3.83140781	0.001441278	-	-
novel.3000	-3.46681911	0.001441278	-	-
108408450	-1.20027711	0.001441278	XBP1	protein_coding
108383554	-1.58378002	0.001441278	ATG4D	protein_coding
108396692	-5.55895164	0.001441278	LOC108396692	protein_coding
108390355	3.411435361	0.001450931	LOC108390355	lncRNA
novel.4321	-3.2274994	0.001459008	-	-
108384856	-2.76755298	0.001472512	YBX3	protein_coding
108402021	-4.00064237	0.001476054	DPF3	protein_coding
novel.8843	-4.26149932	0.001527568	-	-
108396319	1.13128487	0.001575719	KDM5B	protein_coding
108409058	-3.82142992	0.001584004	NCAM1	protein_coding
108393204	-6.56477534	0.001612482	PEBP4	protein_coding
108405278	-4.87659816	0.001612482	CXCR5	protein_coding
108398016	2.273666202	0.001617885	DSG3	protein_coding
novel.5708	-5.97897652	0.001621486	-	-
108384606	-2.33582236	0.001625969	TFDP2	protein_coding
108383286	3.274806268	0.001625969	BNC1	protein_coding
108383767	-1.7489245	0.001626106	ZNF583	protein_coding
108392789	-4.59445747	0.001649718	UCP3	protein_coding
108409008	-3.42627728	0.001658221	CACNA2D1	protein_coding
108385209	-2.11165138	0.001662368	ZNF688	protein_coding
108391037	-4.67123762	0.001707166	CASQ2	protein_coding
108390684	3.395863335	0.001716488	ITGB6	protein_coding
108398466	-1.46927421	0.001716585	ALDOA	protein_coding
108400534	-4.3687898	0.001716799	CLCNKA	protein_coding
108404861	-2.80473136	0.001721742	ABCC8	protein_coding
108393510	2.505563329	0.001772383	LOC108393510	protein_coding
novel.5962	-6.70292113	0.00179481	-	-
108383798	2.267531422	0.001809281	KIF20B	protein_coding
108397061	-2.97022043	0.001810017	KSR1	protein_coding
108390952	1.552072195	0.001810017	LOC108390952	pseudogene
108401173	-2.45733134	0.001810017	LOC108401173	protein_coding
108407239	-2.27116969	0.001810017	ASB1	protein_coding
108389225	2.302613896	0.001820866	LOC108389225	protein_coding
108394870	2.172020577	0.001821031	DSC2	protein_coding
108388005	-2.698191	0.001827141	ARRDC2	protein_coding
108408074	-3.45982348	0.001827141	LOC108408074	lncRNA
108387637	-2.56525565	0.00184885	LOC108387637	lncRNA
108384166	3.116621275	0.001874469	LOC108384166	lncRNA
108392282	-3.60121822	0.001899038	FILIP1	protein_coding

novel.4138	-5.85691759	0.00191639	-	-
108402607	-6.57717518	0.001924715	TECRL	protein_coding
108396127	-1.73395058	0.001934091	LOC108396127	protein_coding
108400197	-2.24906767	0.001953745	FBXO31	protein_coding
108405346	-2.24736142	0.001982291	ANTXR2	protein_coding
108393946	-1.87007961	0.001994664	IL6R	protein_coding
108409156	-2.53747277	0.002013167	LOC108409156	lncRNA
108395479	-2.73364246	0.002027279	SHMT1	protein_coding
novel.7780	-6.12574438	0.002035897	-	-
novel.8659	-5.38510307	0.002072913	-	-
108390163	1.461784144	0.002094681	MXD1	protein_coding
108396797	2.200943984	0.002103089	EVA1A	protein_coding
108383370	-6.36829754	0.002126618	LOC108383370	lncRNA
108392623	-2.57020854	0.00212927	SMYD2	protein_coding
108396507	-4.43439159	0.002160115	LOC108396507	lncRNA
108398402	-3.36812408	0.00223457	LOC108398402	protein_coding
108393196	-1.95443216	0.002264591	PPP3CC	protein_coding
108405106	-3.12190955	0.002264591	LOC108405106	protein_coding
novel.6417	-6.91317847	0.002264591	-	-
108393886	3.162272851	0.002277429	PADI1	protein_coding
108410017	-1.69569419	0.002281995	TMUB2	protein_coding
108386055	-2.25823706	0.002318931	TUBG1	protein_coding
novel.4612	-5.58071719	0.002342603	-	-
108399360	-2.32141851	0.002358134	DCAF6	protein_coding
108399315	-2.40425139	0.002367943	TTC7B	protein_coding
108399236	-3.03613029	0.002367943	ST3GAL1	protein_coding
108402903	-2.50447811	0.002372369	LOC108402903	lncRNA
novel.1558	-5.85376096	0.002378661	-	-
108397949	-4.59219874	0.00237873	LOC108397949	protein_coding
108401193	-1.60705674	0.002388749	SQSTM1	protein_coding
108408142	-1.46001351	0.002452385	EMC3	protein_coding
novel.2199	-1.69598281	0.002464648	-	-
novel.6404	-5.55519799	0.002464648	-	-
108405582	-5.30840397	0.002467483	CGA	protein_coding
108390963	-3.06783491	0.002471061	LOC108390963	protein_coding
108398608	-2.38501847	0.002477495	PHKA1	protein_coding
108393401	-4.26382731	0.002478265	LOC108393401	lncRNA
108393751	-4.00151978	0.00248112	SLC8A3	protein_coding
108384568	-7.14472789	0.002502343	KLHL40	protein_coding
108392790	-5.18348951	0.002507176	LOC108392790	pseudogene
108395858	2.309052973	0.002507176	DEPDC7	protein_coding
108384177	-6.52068862	0.002507176	LMOD2	protein_coding
108388387	3.877849594	0.002547911	GREB1	protein_coding
108394869	2.129622061	0.00255092	DSC3	protein_coding

108384381	3.61477424	0.00255092	LOC108384381	protein_coding
108389885	-2.46660378	0.00255092	LOC108389885	protein_coding
108407917	-2.51783254	0.002559662	DOK7	protein_coding
108397301	2.706075919	0.002559662	NMU	protein_coding
108391679	-1.75574102	0.002604671	LRRC20	protein_coding
108404747	-1.61979247	0.00263214	LOC108404747	protein_coding
108389155	-6.53262998	0.002650824	LOC108389155	lncRNA
108404503	-6.43266081	0.00265209	MYLPF	protein_coding
108402402	3.621397901	0.002652633	OTX1	protein_coding
108409532	-3.98109634	0.002654944	LOC108409532	protein_coding
108400667	2.700436029	0.002656621	PTPRZ1	protein_coding
108399574	2.236039419	0.002658659	KRT14	protein_coding
108386147	-2.26039979	0.002684519	TMEM52	protein_coding
108385019	-2.95305828	0.002688429	JPH1	protein_coding
108393562	2.843850942	0.002699999	E2F8	protein_coding
108406548	-1.74044344	0.002713359	LOC108406548	protein_coding
108387212	-2.68321156	0.002778475	RERE	protein_coding
108383929	-2.25527577	0.002778475	RCSD1	protein_coding
108402648	2.279668513	0.002778475	PLXNB1	protein_coding
108399123	1.191097626	0.002778475	LOC108399123	lncRNA
novel.2770	-3.58681966	0.00285224	-	-
108393167	2.67240912	0.002863234	CHD5	protein_coding
108406614	-2.00055133	0.002980101	LOC108406614	protein_coding
108392203	-2.23063997	0.002980101	LOC108392203	protein_coding
108399659	-1.79012311	0.003015106	GCDH	protein_coding
108396055	-2.8525993	0.00302345	BAG3	protein_coding
novel.2307	-6.48788986	0.003040303	-	-
108401616	-5.35166783	0.003040303	METTL21C	protein_coding
108384558	-2.32974058	0.003064274	ALDH1L1	protein_coding
108395507	1.935868809	0.003066965	STK26	protein_coding
novel.7650	-5.95536523	0.003080472	-	-
108399101	-1.16257486	0.003080472	CCDC127	protein_coding
108385644	4.100808669	0.003090546	LOC108385644	lncRNA
108398789	-6.30024261	0.003098108	TRIM63	protein_coding
108389986	3.351304698	0.003098108	LOC108389986	protein_coding
108391861	1.501506726	0.003103875	MSH2	protein_coding
108392322	1.55341248	0.00311022	BCL6	protein_coding
108410206	2.116541315	0.00311022	LTB4R	protein_coding
108385515	-3.40350725	0.00311022	LOC108385515	protein_coding
108384270	-2.62617488	0.003128973	SYNPO2	protein_coding
108405932	-1.97648381	0.003152091	KCNIP2	protein_coding
108390549	-4.28539225	0.003270365	UBE2QL1	protein_coding
108392022	1.485549712	0.00327326	ZBTB21	protein_coding
108391211	-1.8206525	0.003313953	MAPRE3	protein_coding

108408096	-2.05682693	0.003462977	APBB1	protein_coding
108400665	-6.8656947	0.003466952	TPH2	protein_coding
108394126	-4.19712562	0.003490181	LOC108394126	lncRNA
108406664	-4.57913237	0.003538496	SEC14L5	protein_coding
108406801	-2.36693714	0.003538496	RAPGEF1	protein_coding
108402822	2.571516784	0.003559067	ARSJ	protein_coding
108405989	2.481749618	0.003575278	DSN1	protein_coding
108387729	-2.49879164	0.003622756	RAB11FIP5	protein_coding
108394753	1.541790017	0.003622756	CD82	protein_coding
108389754	-2.33791987	0.003629537	LOC108389754	protein_coding
novel.1641	-8.33595852	0.003659945	-	-
108405741	-1.93001872	0.003675067	BTBD6	protein_coding
108408816	2.328129404	0.003675067	ASPG	protein_coding
108402292	1.833061238	0.00376914	JAG1	protein_coding
108383351	-1.96236221	0.003777727	TBC1D17	protein_coding
108393407	-1.79403549	0.003777992	GAB2	protein_coding
108404272	-1.57825334	0.003796256	FXR2	protein_coding
108399558	-6.86163989	0.003821416	ANKRD1	protein_coding
108390499	-4.26827562	0.003821416	HSPB3	protein_coding
108407035	-2.18420961	0.003879415	HSPBAP1	protein_coding
108405736	-7.6936806	0.003887379	CUNH10orf71	protein_coding
108391067	-1.90089445	0.003906939	PHOSPHO1	protein_coding
108405742	-1.88969584	0.003944997	PACS2	protein_coding
108389943	-2.1782127	0.004010587	PGM1	protein_coding
108388443	-1.9885597	0.004045213	PCTP	protein_coding
108402848	-2.93215356	0.004045213	CAMKV	protein_coding
108393919	-5.49483983	0.004050094	LOC108393919	protein_coding
108407141	-1.8600271	0.0040523	MRPL14	protein_coding
108392197	2.539239996	0.0040523	TOP2A	protein_coding
108405992	-2.35190791	0.004104612	SAMHD1	protein_coding
108392317	1.845157099	0.004171185	TP63	protein_coding
108402384	-2.20491924	0.00420557	LARP6	protein_coding
108386251	-1.82014997	0.004220365	LOC108386251	protein_coding
108392310	-1.27561965	0.004295627	NDUFB9	protein_coding
108399596	2.385815863	0.004306794	LOC108399596	lncRNA
108396428	2.173799642	0.004318189	P2RY1	protein_coding
108387454	-4.37702951	0.004318189	ADAM23	protein_coding
108388696	-3.96346644	0.004323464	JAZF1	protein_coding
novel.5907	2.843549521	0.004378378	-	-
108391970	-3.46715409	0.004378378	MLIP	protein_coding
108409264	2.146893145	0.004397305	COL7A1	protein_coding
novel.8290	-6.36306423	0.004397305	-	-
108395648	-1.83360689	0.004414266	CMTM7	protein_coding
108409638	-2.64088563	0.00445719	FOXO1	protein_coding

novel.7266	-1.71445718	0.004468903	-	-
108399062	-3.40795809	0.004477298	LOC108399062	protein_coding
novel.936	-2.49689717	0.004519298	-	-
108407765	-5.14931938	0.004551444	FADS3	protein_coding
108402934	-7.03854698	0.004602242	IP6K3	protein_coding
108394747	3.257900338	0.004640202	TMEM169	protein_coding
108389787	-2.12397808	0.004649234	LOC108389787	lncRNA
108389270	-1.69603693	0.004796061	SNX18	protein_coding
novel.7797	-4.72792345	0.004838343	-	-
108407182	-5.97373629	0.004879045	ACTN2	protein_coding
108408183	-4.22847076	0.004918361	CD38	protein_coding
108402408	-3.03324813	0.004921166	SRPK3	protein_coding
108388589	-2.40939162	0.004990347	GCLM	protein_coding
novel.415	3.359683847	0.005112899	-	-
108404440	1.890218556	0.005112899	MARCKSL1	protein_coding
108393797	-1.77554892	0.005112899	AGPAT1	protein_coding
108394450	2.656913623	0.005112899	KCNK10	protein_coding
novel.3630	-6.49444919	0.005129178	-	-
108399914	2.807469766	0.005129178	NCKAP5	protein_coding
108403009	-1.57838539	0.005130487	ASB8	protein_coding
108391484	-1.35783546	0.005130487	UBC	protein_coding
108386694	1.252766817	0.005147122	LOC108386694	protein_coding
108398970	1.201406234	0.005170538	LSR	protein_coding
108409619	2.859298267	0.00518648	LRRC8E	protein_coding
novel.7400	-3.07650749	0.005247519	-	-
108395929	-3.17888672	0.005314632	METTL7B	protein_coding
108409046	2.015970128	0.005332365	SCNN1D	protein_coding
108399958	-3.43315237	0.005359248	LOC108399958	protein_coding
108399805	-4.03369731	0.005437476	MUSK	protein_coding
108386423	-1.63034672	0.005455804	CUNH1orf216	protein_coding
108399769	-1.80607458	0.005455804	AIFM1	protein_coding
108405433	-2.48283365	0.005490628	PHLDA3	protein_coding
108405940	2.45975767	0.005507161	LOC108405940	protein_coding
108390285	1.774528517	0.005513619	IKZF2	protein_coding
108395846	-1.94504694	0.005515826	IFT22	protein_coding
108384284	-2.08467531	0.005526616	LOC108384284	protein_coding
108398815	-2.25359861	0.005526616	WNT9A	protein_coding
108407237	3.027156828	0.005526616	LOC108407237	protein_coding
108405559	-1.14087584	0.005526616	DEXI	protein_coding
108387011	-2.47630825	0.005608378	LOC108387011	protein_coding
108399138	2.856242552	0.005705163	UNC5C	protein_coding
108390787	-1.33080062	0.005744249	LOC108390787	protein_coding
novel.1070	-5.3794886	0.005744249	-	-
108406989	-1.9973131	0.005744249	ABCA3	protein_coding

108388939	-5.86529157	0.00577392	LOC108388939	protein_coding
novel.8285	-4.04889182	0.005840167	-	-
108408308	-3.01411763	0.005932599	LOC108408308	lncRNA
novel.7274	-5.70562876	0.006021241	-	-
novel.3712	2.844721417	0.006115427	-	-
108387400	-5.77692353	0.006115901	LOC108387400	protein_coding
108406496	-2.11451481	0.006125628	YBX2	protein_coding
108387050	2.132149832	0.006163153	SEMA4B	protein_coding
108386862	-1.97311068	0.006197745	THTPA	protein_coding
108384505	-6.15222683	0.006215522	POPDC3	protein_coding
108405537	-1.59193546	0.006277791	DHRS7	protein_coding
108402939	-1.41683002	0.006279853	LOC108402939	protein_coding
108401180	2.908952686	0.006339702	HJURP	protein_coding
novel.3549	-5.21842749	0.00641959	-	-
108407135	1.863370988	0.00641959	VEGFA	protein_coding
108391902	2.789719505	0.00644496	SERTAD4	protein_coding
108392394	-2.58334527	0.006457174	FADS1	protein_coding
108404380	2.058304291	0.006489952	PERM1	protein_coding
novel.131	-1.7949339	0.006598498	-	-
108385598	-1.59826475	0.006665299	BCL2L13	protein_coding
108401595	-3.07969251	0.006692049	RPRD2	protein_coding
108404357	3.856925144	0.006699361	LOC108404357	protein_coding
108409286	2.275092361	0.006699361	B3GNT7	protein_coding
108405345	-2.08923579	0.006715692	LOC108405345	protein_coding
108406701	-1.82508663	0.006721605	LOC108406701	protein_coding
108402151	-1.14842277	0.006733987	MFGE8	protein_coding
108387228	2.154969152	0.00678077	ARL4D	protein_coding
108403687	-4.22171531	0.006785872	ARX	protein_coding
108396175	-1.95438561	0.006852111	LOC108396175	lncRNA
108402129	-3.03700052	0.006872584	LOC108402129	lncRNA
108407290	-2.29277588	0.006890292	KCNN3	protein_coding
108395241	-4.61647958	0.006890292	MYADML2	protein_coding
novel.7864	-3.57080105	0.006930536	-	-
108406381	-6.53156826	0.006966179	NEB	protein_coding
108403638	-5.16485617	0.00699712	LOC108403638	protein_coding
108399431	-1.55864718	0.00699712	LOC108399431	protein_coding
108409553	-1.88141984	0.00699712	MLLT11	protein_coding
108398360	-3.01763875	0.007012547	ABCC9	protein_coding
108391654	3.066734193	0.007020225	TICRR	protein_coding
108395260	1.149847946	0.007031375	MYO5A	protein_coding
108401262	2.006028104	0.007048033	TMEM51	protein_coding
novel.7741	-2.32987265	0.007048033	-	-
108397052	-2.04515008	0.007107032	SLC3A2	protein_coding
108389545	1.817924759	0.007135479	SOX7	protein_coding

108401945	-2.01599764	0.007166526	SRXN1	protein_coding
108389096	3.226962959	0.007166526	CDHR1	protein_coding
108403048	2.572544398	0.007367801	IGSF9	protein_coding
108387907	2.954641206	0.007389722	LOC108387907	protein_coding
108403011	-2.24084981	0.007389722	LOC108403011	protein_coding
108386559	-1.66890299	0.007416424	SLC27A1	protein_coding
108384537	-1.85661667	0.007417211	ZNF287	protein_coding
108390469	3.95459824	0.007426367	PCDH20	protein_coding
108389320	-2.86756427	0.007432447	LOC108389320	lncRNA
108384036	-3.40802515	0.007436924	LOC108384036	protein_coding
108406637	2.178144988	0.007436924	DLK2	protein_coding
108396972	-2.6353541	0.00755868	NEURL1	protein_coding
108392910	-1.6549317	0.007611943	NEURL2	protein_coding
108401692	-1.36338538	0.007633713	LOC108401692	protein_coding
novel.2756	-4.56590104	0.007654588	-	-
108400950	-4.12641195	0.007654863	LOC108400950	lncRNA
108391973	-4.27357944	0.007693365	KLHL31	protein_coding
108403954	-1.83933807	0.007693365	MYOM2	protein_coding
108402603	-1.8195149	0.007693365	PLA2G15	protein_coding
108386845	1.479338399	0.00769652	CTTNBP2NL	protein_coding
108384778	-1.26071413	0.007763519	RAX2	protein_coding
108397018	-2.15374519	0.007832706	CUNH11orf74	protein_coding
108403076	-5.91122715	0.007857763	MYOT	protein_coding
108387362	-1.17109112	0.007958942	PNKD	protein_coding
108384654	-2.38741484	0.008008656	CUNH15orf65	protein_coding
108402057	1.611391477	0.008139881	BAZ1A	protein_coding
108398490	-2.81452433	0.008139881	ZFP37	protein_coding
108405410	-3.88417276	0.008139881	PPP1R12B	protein_coding
novel.820	-3.40120567	0.008139881	-	-
108405479	-4.30087682	0.008174623	TRNAN-GUU	tRNA
108405920	1.622881061	0.008190914	NPM3	protein_coding
108400311	-2.03319073	0.008190914	GYS1	protein_coding
108393282	-1.9306435	0.008190914	ZDHHC2	protein_coding
108399895	-2.33957351	0.008194417	LARGE1	protein_coding
108392405	-1.91782236	0.008204818	SLC16A6	protein_coding
108410056	-1.27479336	0.008221839	LOC108410056	protein_coding
108394099	-2.24660491	0.008221839	PTPN21	protein_coding
108401663	-3.94076238	0.008221839	PALM2	protein_coding
108397270	-3.24167581	0.008265361	LOC108397270	lncRNA
108395203	-1.105761	0.008308763	PEBP1	protein_coding
108393608	1.949031142	0.008308763	CDCP1	protein_coding
108393284	-2.35788694	0.008310737	SLC7A2	protein_coding
108386946	2.355377715	0.008394772	KCNJ15	protein_coding
108389073	-2.50998767	0.008400681	LOC108389073	pseudogene

108392620	-1.78164592	0.008400681	PROX1	protein_coding
108406772	-3.0800612	0.008469039	LOC108406772	protein_coding
108409021	-2.73012958	0.008514403	F5	protein_coding
108410350	-2.50574323	0.008532486	LOC108410350	protein_coding
108385992	1.935651017	0.008572904	CKAP2	protein_coding
108397359	-1.65632936	0.008815094	CBX7	protein_coding
108387280	2.772703397	0.008983419	LOC108387280	protein_coding
108391152	-2.58894949	0.008992557	ERRFI1	protein_coding
108402950	2.680514185	0.009062785	VANGL2	protein_coding
108390057	-2.17588133	0.009089424	SAMD4A	protein_coding
108410139	-1.74272711	0.009122704	MCPH1	protein_coding
108403636	2.419419277	0.009122704	GDPD2	protein_coding
108391636	-2.45735832	0.009144723	FBXO30	protein_coding
108397497	-1.54944406	0.00919593	GRHPR	protein_coding
novel.5156	-6.14479235	0.00919593	-	-
novel.3524	-1.95518267	0.009296568	-	-
108408324	-2.15333411	0.009306291	LOC108408324	protein_coding
108385309	2.335966213	0.009306291	MAEL	protein_coding
108396360	2.54812367	0.00934369	PCDH7	protein_coding
novel.5909	3.76598443	0.009375357	-	-
108405063	1.713871013	0.009395803	LOC108405063	protein_coding
108403094	-2.60993346	0.009426649	ALPK2	protein_coding
108395930	-0.96385569	0.009449275	BLOC1S1	protein_coding
108391803	-3.50237588	0.009511966	LOC108391803	protein_coding
108406010	-2.37472585	0.009514798	LOC108406010	lncRNA
108407386	-8.23606642	0.009579399	LOC108407386	protein_coding
108390743	-1.37588487	0.009579399	LOC108390743	lncRNA
108407731	-2.95194045	0.009581367	SUSD1	protein_coding
108390550	1.52812787	0.009619244	PDZD2	protein_coding
108393890	2.042943181	0.009628793	EPHA2	protein_coding
108398139	-1.67498211	0.009706913	MYL6B	protein_coding
108391340	-1.60263993	0.009719408	PCBP4	protein_coding
108393826	-2.75621632	0.009719408	MPV17L	protein_coding
108408611	2.492907454	0.009785899	TERT	protein_coding
108396713	-6.2535254	0.009785899	TRIM55	protein_coding
108405588	1.225333042	0.009796249	MTHFD1L	protein_coding
108398373	2.445898711	0.009990158	PROKR2	protein_coding
108404207	-1.66647749	0.01000581	AKIRIN2	protein_coding
novel.5823	-3.70263083	0.01002983	-	-
108386426	-2.20974812	0.01002983	LOC108386426	protein_coding
108397951	-2.61277533	0.010062991	CCDC141	protein_coding
108404778	-2.15345578	0.01007058	SMAD3	protein_coding
108383253	-2.65738829	0.010221092	LOC108383253	protein_coding
108393479	-5.40120487	0.010271003	LOC108393479	lncRNA

novel.2766	-1.67294533	0.010339045	-	-
108405431	-1.93766181	0.010339045	ARL8A	protein_coding
108402363	-1.41979316	0.010339045	FDFT1	protein_coding
108403323	-1.69213801	0.010339045	FOXO4	protein_coding
108386146	1.697820614	0.010339755	LOC108386146	lncRNA
108396514	2.581842956	0.010339755	LOC108396514	protein_coding
108406226	-2.37456846	0.010407915	LOC108406226	protein_coding
108389629	-6.12789645	0.010442331	LOC108389629	protein_coding
108407562	-1.44984694	0.010442331	SOBP	protein_coding
108389256	-2.35703367	0.010442331	LOC108389256	protein_coding
108391421	-2.7992645	0.010443609	LRRC3	protein_coding
108384927	-1.39212942	0.010455128	PIGT	protein_coding
108405655	-2.04106687	0.010482615	LOC108405655	lncRNA
108398962	2.31304661	0.010546761	FXYD3	protein_coding
108406888	-1.00618784	0.01055538	MRPS36	protein_coding
108408345	-2.68401067	0.01072558	TTLL7	protein_coding
108409265	1.700903347	0.010870216	LOC108409265	protein_coding
108391187	3.027509583	0.01092987	DEPDC1	protein_coding
108388483	-1.69671123	0.010989804	LOC108388483	protein_coding
108394537	2.363396843	0.011027556	AIM2	protein_coding
108407508	-2.70703101	0.011035679	RASL10B	protein_coding
novel.5152	-3.38555816	0.011090445	-	-
novel.7423	-5.62882036	0.011090445	-	-
108384619	-2.15434708	0.01111034	LOC108384619	protein_coding
108399033	-3.6106339	0.011111634	PRG4	protein_coding
novel.4127	-4.61612482	0.011162416	-	-
108406529	1.782127314	0.011175539	MXRA5	protein_coding
novel.7061	3.314861184	0.011195278	-	-
108405139	-2.87748091	0.011300306	RASD1	protein_coding
108396372	-5.46425779	0.011301091	PYGM	protein_coding
108386792	-2.69688517	0.011316838	SORBS1	protein_coding
novel.2749	-3.66986881	0.011316838	-	-
108396091	-2.08958867	0.011334287	MVB12B	protein_coding
novel.4631	-4.0759333	0.011444106	-	-
108404176	-0.91731884	0.01148023	MPC1	protein_coding
108395051	-1.37508631	0.01148023	PPP2R5D	protein_coding
108393964	-2.48584593	0.011490776	ZC4H2	protein_coding
108386904	-1.86242953	0.011556577	LOC108386904	protein_coding
108392112	-1.93921693	0.011568466	FOXK1	protein_coding
108392977	-3.12178421	0.011654757	WNK2	protein_coding
108408582	-1.79078619	0.01167138	TMEM140	protein_coding
108392595	2.044498276	0.01167138	FAM78B	protein_coding
108394066	-1.53891275	0.011695923	PPDPF	protein_coding
108407507	-3.13000699	0.011695923	GAS2L2	protein_coding

novel.4488	-2.57881038	0.011695923	-	-
108409539	-1.3138559	0.011822082	NENF	protein_coding
108396003	2.09624709	0.011857127	CUNH1orf106	protein_coding
108408392	-3.82474532	0.011993225	ITIH3	protein_coding
novel.7032	-5.44144212	0.012002693	-	-
108397857	-1.6658637	0.012062135	VARS	protein_coding
108393464	-1.63868255	0.012137679	FLRT2	protein_coding
108395832	-1.78339602	0.012188443	VEZT	protein_coding
novel.2893	-3.71300157	0.012267667	-	-
novel.2776	-4.42072698	0.012314976	-	-
108406205	-1.91131847	0.012369789	SLC25A23	protein_coding
novel.7891	-4.97402178	0.012369789	-	-
108388422	-2.36935836	0.012486552	FHL3	protein_coding
novel.3079	-6.47808007	0.012496455	-	-
108409834	-5.27023598	0.012571416	LOC108409834	protein_coding
108394519	-2.37627528	0.012571416	LOC108394519	protein_coding
108392199	2.076461491	0.012654161	TNS4	protein_coding
novel.8205	-3.43902561	0.012662134	-	-
108406230	2.145022154	0.012695938	LOC108406230	protein_coding
novel.3971	-5.06307453	0.012731746	-	-
108406534	1.454561316	0.012779813	JARID2	protein_coding
108403095	1.376759795	0.012896212	MALT1	protein_coding
108395426	2.198559049	0.012896212	MBNL3	protein_coding
108405397	1.591088417	0.012896212	CSAD	protein_coding
novel.6760	1.921340297	0.012896212	-	-
108386839	1.568906732	0.012907305	LOC108386839	protein_coding
108399716	-1.74296067	0.012907305	STX2	protein_coding
108389989	1.880285952	0.012907305	NUSAP1	protein_coding
108389605	-1.60323314	0.012907305	LOC108389605	protein_coding
novel.2793	-3.47003309	0.01293529	-	-
108395771	-1.20048622	0.012951533	ENDOG	protein_coding
108386438	-1.67018365	0.012965	CDKN2B	protein_coding
108393750	-3.68302919	0.013005381	LOC108393750	protein_coding
108395268	-1.58644462	0.013233366	PSMD2	protein_coding
108408071	1.919164331	0.013244653	ROBO1	protein_coding
108405373	2.142668255	0.013279872	CDCA2	protein_coding
108403293	1.238225642	0.013334516	ITPR2	protein_coding
108388345	-1.95619297	0.013586752	GHDC	protein_coding
108387549	-2.1511132	0.013640642	MFSD6L	protein_coding
108400370	2.640857101	0.013649951	LOC108400370	lncRNA
novel.6222	-6.04014207	0.013687007	-	-
108387236	-1.458194	0.013695572	DUSP3	protein_coding
108393019	-2.45897834	0.013728816	FAM134B	protein_coding
108406715	2.047526629	0.013728816	SLC2A1	protein_coding

novel.136	-2.31458874	0.013790767	-	-
108397984	1.740139574	0.013821902	STON2	protein_coding
108397972	-1.67081813	0.01390167	FPGS	protein_coding
108403694	-1.76073891	0.01420257	ZER1	protein_coding
108383352	-1.4598718	0.01420257	AKT1S1	protein_coding
108406625	2.886617033	0.01420257	LOC108406625	protein_coding
108401159	-1.31454753	0.014279261	PIGH	protein_coding
novel.3758	-5.02716619	0.014423411	-	-
108387375	-2.03448217	0.01449764	SLC1A4	protein_coding
novel.1533	2.140139023	0.014552923	-	-
108402437	2.926242599	0.01458024	MYOCD	protein_coding
108393605	-1.49155886	0.01460685	TMEM120A	protein_coding
108395172	-1.66846852	0.014656628	TSC22D3	protein_coding
108402407	2.045466231	0.014922346	PLXNB3	protein_coding
108397701	2.849827193	0.015009513	CABP1	protein_coding
108408184	3.469333957	0.015029868	CEP55	protein_coding
108390225	1.757541252	0.015029868	LOC108390225	protein_coding
108409629	-6.09481534	0.015029868	KLHL38	protein_coding
108408564	1.155963585	0.015029868	ZNF184	protein_coding
108399615	2.015586014	0.015072964	LOC108399615	protein_coding
108400715	-4.42255436	0.015112786	LOC108400715	protein_coding
novel.5151	-4.99889334	0.015160319	-	-
108385174	-1.67664079	0.01521954	ALDH6A1	protein_coding
108400569	-2.08831832	0.015239464	TCP1	protein_coding
108394989	1.994656676	0.015239464	RNF17	protein_coding
108397333	1.092921743	0.01529585	TET2	protein_coding
108406549	-1.71639389	0.015299847	HOXD8	protein_coding
108396053	-1.63445588	0.015368842	PRDX3	protein_coding
108393146	-2.0690028	0.01541926	CARNS1	protein_coding
108391227	-1.92540976	0.015440008	ALPK3	protein_coding
108384714	1.682553013	0.015474113	P3H2	protein_coding
108383538	-1.503615	0.015501067	LOC108383538	lncRNA
108395598	-2.17800742	0.015529215	MAPK12	protein_coding
108409412	-1.1593367	0.015655725	NOL7	protein_coding
108407088	1.316857897	0.015767738	PTPRK	protein_coding
108390176	2.186723602	0.015975005	FAM92A1	protein_coding
108403327	-1.86332338	0.016122852	PRKAB2	protein_coding
108395182	1.496436906	0.016122852	COL4A6	protein_coding
108391398	-1.59130817	0.016122852	FEM1A	protein_coding
108387477	-5.91805791	0.016122852	TMEM130	protein_coding
108394268	1.657467123	0.016122852	RABGAP1L	protein_coding
108395861	-1.55262824	0.016122852	LOC108395861	lncRNA
108387536	-2.56690813	0.016192584	DGKD	protein_coding
108410224	2.241232016	0.016192584	LOC108410224	lncRNA

108400586	-2.77458827	0.016192584	LOC108400586	protein_coding
108386747	-2.31218002	0.016192584	ANO5	protein_coding
novel.7648	-3.87083648	0.016192584	-	-
108399114	-6.05534696	0.016192584	AGBL1	protein_coding
108392492	-6.38946987	0.016387228	CACNG1	protein_coding
108390380	2.294109192	0.016393118	KNTC1	protein_coding
108397920	-2.72538125	0.016393118	MYBPC2	protein_coding
108389565	-3.13232709	0.016393118	LOC108389565	protein_coding
novel.5435	-2.88501136	0.016394341	-	-
108396510	-1.70109415	0.016417598	LOC108396510	protein_coding
108396820	-1.78244242	0.016504624	DNAJC28	protein_coding
108386636	-2.23964161	0.016639028	LOC108386636	protein_coding
108396015	1.129882298	0.016639028	LOC108396015	protein_coding
108401680	2.071104343	0.016639028	LOC108401680	protein_coding
108401719	2.231606508	0.016692182	SLC4A8	protein_coding
108396449	-1.78608098	0.016692182	LOC108396449	lncRNA
108409101	-2.65083219	0.016692182	ROCK2	protein_coding
108400328	-1.28404173	0.016806789	BCAT2	protein_coding
108390630	-1.47595708	0.016840381	NUDT16	protein_coding
108405914	-1.79774411	0.016840381	RNF157	protein_coding
novel.1979	2.030842703	0.016910705	-	-
novel.2805	-1.66716828	0.017005588	-	-
108392152	2.458498986	0.017059716	SLITRK6	protein_coding
novel.1699	-3.16920395	0.017059716	-	-
novel.7133	-1.52491211	0.017059716	-	-
108408407	-2.23794774	0.017059716	LOC108408407	protein_coding
108401579	1.235192554	0.017198851	DBF4	protein_coding
108388011	-1.78522141	0.017242099	MAP1S	protein_coding
108399111	-2.31460008	0.017333767	TMEM170B	protein_coding
108385381	-1.23255873	0.017333767	DRG1	protein_coding
108400639	2.016016986	0.017466009	ITGB4	protein_coding
108390465	1.83876979	0.017466009	LRIG3	protein_coding
108389048	1.368560551	0.017473859	LOC108389048	protein_coding
108387213	-2.33841217	0.017501643	SLC45A1	protein_coding
108392661	-3.14863548	0.017501643	P2RX5	protein_coding
108394639	1.647212577	0.017623251	ALKBH8	protein_coding
novel.1482	-5.71462735	0.017684698	-	-
108404758	-2.84216963	0.017924235	APC2	protein_coding
108401106	2.196474486	0.017937476	PRSS16	protein_coding
108392548	-2.81734596	0.018017488	SPEG	protein_coding
108383476	-1.67723229	0.018086322	POMGNT2	protein_coding
108408749	1.630446881	0.018086322	NOTCH3	protein_coding
108397462	-2.58555039	0.01808832	PAIP2B	protein_coding
108400789	-2.48261917	0.018219758	LOC108400789	lncRNA

novel.722	-5.31064215	0.018270351	-	-
novel.982	-1.70984409	0.0182827	-	-
108393442	-2.57589772	0.018344483	ST8SIA4	protein_coding
108397843	2.121430111	0.018344739	ANLN	protein_coding
novel.7355	-1.73949194	0.018373581	-	-
108401987	-1.82539546	0.01849569	CCPG1	protein_coding
108399136	-1.4899466	0.018521761	ABCC4	protein_coding
108409995	-2.59467719	0.018521761	FBXL22	protein_coding
108389281	-1.56038821	0.018785458	LOC108389281	lncRNA
novel.2404	-3.15902225	0.018785458	-	-
108385159	-1.55093846	0.018790658	PSMD3	protein_coding
108405251	-2.58009329	0.018802267	LOC108405251	lncRNA
108397027	-1.53512613	0.018872558	LYRM9	protein_coding
108393449	0.994671147	0.018922995	HAS3	protein_coding
108400564	-2.13713734	0.019014087	MRPL18	protein_coding
novel.8024	2.019836975	0.019014087	-	-
108397336	-2.13530127	0.019191544	ANO6	protein_coding
108385826	1.095332744	0.019202968	SHMT2	protein_coding
108398963	-1.62545789	0.019235077	FXYD1	protein_coding
108385892	2.359047499	0.019299379	LOC108385892	lncRNA
108385043	2.158609913	0.019340482	JAML	protein_coding
108398991	1.335229527	0.019360768	PLPPR2	protein_coding
108399247	-2.83186504	0.019639359	PTPRM	protein_coding
108402164	-3.26933622	0.019664152	LMCD1	protein_coding
108388159	-4.9143873	0.019699757	LSMEM1	protein_coding
108392055	2.445933356	0.019710442	ARHGAP11A	protein_coding
108404410	-4.84888135	0.019768765	PHF24	protein_coding
108401022	-5.27260134	0.019768765	LOC108401022	protein_coding
108405258	1.373029794	0.019769982	NFKBIZ	protein_coding
108384598	-2.11366618	0.019769982	CAMKK2	protein_coding
108393339	-1.56978048	0.019796969	RNF123	protein_coding
108396542	-5.44239022	0.019824767	SMPX	protein_coding
108404313	-1.45749036	0.019849734	PSMC1	protein_coding
108386104	-1.55636972	0.019849734	LMF1	protein_coding
108401625	1.460992029	0.019895758	ARHGAP39	protein_coding
108404210	1.549744344	0.019913022	PRICKLE3	protein_coding
novel.8652	-3.31200125	0.019946001	-	-
108402359	-1.01790079	0.019962071	LOC108402359	protein_coding
108405008	-1.99413585	0.020051112	PRUNE2	protein_coding
novel.4273	2.836870472	0.020051112	-	-
108396276	-5.799179	0.020051112	LOC108396276	lncRNA
108384652	-2.04675001	0.020136552	LOC108384652	protein_coding
108387167	-1.54280425	0.020295249	LOC108387167	protein_coding
108398823	-1.26989876	0.020295249	SNAP47	protein_coding

108395057	1.840889806	0.020387679	GNMT	protein_coding
novel.4035	2.379986203	0.020466231	-	-
108394723	1.952269829	0.020466231	EXPH5	protein_coding
108405413	-2.24323031	0.020466231	LMOD1	protein_coding
novel.8388	3.187989544	0.020466231	-	-
108408160	-1.05448234	0.020547771	VAPB	protein_coding
novel.4745	-1.94176703	0.020572594	-	-
108395829	-2.02657933	0.020583509	NCDN	protein_coding
108400201	-5.7838122	0.020739174	LOC108400201	protein_coding
108400029	-1.80724014	0.020739174	SLC7A8	protein_coding
108388503	-5.8447758	0.02075444	LOC108388503	protein_coding
108386186	2.119509517	0.02079403	B4GALNT3	protein_coding
novel.8558	-5.85154478	0.020958036	-	-
108395340	5.16010541	0.020958036	SCIMP	protein_coding
108398365	-1.51489112	0.020958036	CMAS	protein_coding
108402992	3.260657165	0.020958036	KRT8	protein_coding
108396064	2.129240741	0.020964456	NECTIN1	protein_coding
108399444	1.947861526	0.021079978	GPR68	protein_coding
novel.3705	-2.56595833	0.021155309	-	-
108390096	2.338662358	0.02116485	ATAD2	protein_coding
108387655	-1.69153445	0.021244025	INPP5E	protein_coding
108396767	1.454722749	0.021246372	CORO2A	protein_coding
108398707	-3.55912975	0.021267004	GRTP1	protein_coding
novel.4632	-4.3269735	0.021276466	-	-
108402986	1.93401376	0.021315872	LOC108402986	protein_coding
108384933	1.624947743	0.021357171	CXADR	protein_coding
108390100	-1.87387322	0.021357486	LOC108390100	protein_coding
108390177	2.526833115	0.021357486	RAD54B	protein_coding
108394650	1.539678374	0.021449136	TBC1D32	protein_coding
108387330	-1.67936448	0.021499399	LOC108387330	protein_coding
108403399	2.198484624	0.021516673	KLRG2	protein_coding
108387725	-1.38813609	0.021522176	PRADC1	protein_coding
108391250	-1.8683547	0.02154381	LOC108391250	protein_coding
108390125	-3.56161136	0.02154381	LOC108390125	lncRNA
108406597	-2.24959128	0.021609542	LOC108406597	protein_coding
108391676	-1.73260248	0.021609542	ZBED1	protein_coding
108387706	1.337622734	0.022046759	ZNF180	protein_coding
novel.3198	2.127510102	0.022076308	-	-
novel.1984	-2.09755155	0.022076308	-	-
novel.7865	-3.35277118	0.022128903	-	-
108405835	2.097880004	0.022184553	ALDH3B1	protein_coding
108409076	0.924967406	0.022221694	IER3	protein_coding
novel.8778	-3.16032645	0.022221694	-	-
108404488	-2.12852784	0.022221694	LOC108404488	lncRNA

108385653	-4.26739261	0.022255556	MARCO	protein_coding
108393194	-1.49123118	0.022255556	POLR3D	protein_coding
108409366	2.550067153	0.022255556	ZAN	protein_coding
novel.500	-3.76541444	0.022303506	-	-
108384382	-2.49539845	0.022303506	LOC108384382	lncRNA
novel.6399	-4.01088125	0.022303506	-	-
108393073	-5.32793613	0.022315001	LOC108393073	protein_coding
108400787	-1.43445484	0.022384654	PELO	protein_coding
108409284	-1.75026368	0.02243633	PSMD1	protein_coding
108405771	2.319388094	0.022439103	ESR1	protein_coding
108405640	2.950087142	0.022447886	KIAA0101	protein_coding
108402850	1.931166307	0.022452195	MST1R	protein_coding
108407047	-3.09507454	0.022583262	CKMT2	protein_coding
108397493	1.864248887	0.022661018	LOC108397493	lncRNA
108387650	1.368540813	0.022661018	NOTCH1	protein_coding
108385274	-1.45936112	0.022706296	PHYH	protein_coding
108396674	-2.37923052	0.022706296	SGMS1	protein_coding
novel.2651	1.79110069	0.022808309	-	-
108384750	1.506351997	0.022863665	EVA1C	protein_coding
108399989	-8.08921612	0.022966147	LOC108399989	lncRNA
108402158	-2.70456341	0.022985845	NTRK3	protein_coding
108399461	0.993695841	0.023001896	LYST	protein_coding
108401736	-3.10129309	0.023096454	LOC108401736	protein_coding
novel.5008	1.113736566	0.023129262	-	-
108403272	1.8296984	0.023140012	CCNB1IP1	protein_coding
108409311	-3.64565919	0.023247595	LOC108409311	lncRNA
108399165	-1.30451224	0.023247595	CABLES2	protein_coding
108388067	1.359269557	0.023358374	HOXA1	protein_coding
108384684	2.841315253	0.023377194	LCN2	protein_coding
108408083	-5.6660959	0.023377194	ACTA1	protein_coding
novel.3756	-1.51703527	0.023412702	-	-
108387288	2.428691513	0.023433404	SRCIN1	protein_coding
108395616	-1.97623124	0.02344598	REPS2	protein_coding
108393838	1.897486244	0.023522209	CKS2	protein_coding
108385408	2.042472572	0.023537597	SPTBN5	protein_coding
108402665	-1.57981692	0.023543306	TMEM53	protein_coding
108383459	1.98622522	0.023630934	MAP3K9	protein_coding
novel.5162	-3.69832	0.023686397	-	-
108388079	-2.11730849	0.023686397	GABARAPL1	protein_coding
108396506	-2.53660116	0.023686397	DIXDC1	protein_coding
108389090	-4.05227114	0.023703619	LOC108389090	protein_coding
108383513	1.12727098	0.023703619	SLC9A1	protein_coding
novel.2031	-4.05742632	0.02373713	-	-
108409892	2.908807687	0.02373713	LOC108409892	protein_coding

108387137	-1.74461642	0.02373713	BHLHE41	protein_coding
novel.3103	1.587829372	0.02373713	-	-
108395741	1.645938278	0.02386777	DLL1	protein_coding
108403083	-2.06118867	0.023941499	LOC108403083	protein_coding
108394754	-4.40783202	0.024126628	ALX4	protein_coding
108391815	-1.84584379	0.024204864	LOC108391815	protein_coding
108402517	-1.73750815	0.024232951	MTMR1	protein_coding
108401043	1.094915458	0.024421545	CCDC15	protein_coding
108400537	-2.15860491	0.024421545	ATP2A2	protein_coding
108385882	2.198738682	0.024421545	LOC108385882	protein_coding
novel.6096	-1.67833449	0.024421545	-	-
108386154	-1.35672076	0.024423705	FAAP20	protein_coding
108388115	1.73854833	0.024464881	NCAPG	protein_coding
108396599	-1.46934443	0.024589761	WDYHV1	protein_coding
108392463	-2.12239896	0.024589761	OSBPL6	protein_coding
108393027	-2.36392527	0.024589761	MEF2C	protein_coding
108395358	-1.76179145	0.024589761	LOC108395358	protein_coding
108383404	-4.06373745	0.024589761	TSPAN8	protein_coding
108401740	3.582631274	0.024589761	DNA2	protein_coding
108400195	-1.34769709	0.024659965	MAP1LC3B	protein_coding
108385366	1.99036602	0.024687711	LOC108385366	lncRNA
novel.3521	1.955307508	0.024700512	-	-
108404500	-1.34632733	0.024773688	ZNF48	protein_coding
108383769	-1.96680649	0.024837877	ANK2	protein_coding
108392048	1.263339886	0.024862455	LOC108392048	protein_coding
108385287	2.80185559	0.025007501	LOC108385287	protein_coding
novel.2166	-2.15690791	0.025007501	-	-
108408629	2.48708696	0.025026602	LMNTD2	protein_coding
108393786	-1.62423427	0.025042094	PSMA1	protein_coding
108400698	-1.46380502	0.025042094	LOC108400698	protein_coding
108395835	-0.96079295	0.025042094	FIS1	protein_coding
108408433	1.475758374	0.025042094	CD44	protein_coding
108386836	-2.30015945	0.02509773	LOC108386836	protein_coding
108393575	-1.74704347	0.025162714	FAXC	protein_coding
108399692	-1.46274575	0.025218141	ZNF358	protein_coding
108398220	-1.80129789	0.025318149	GRIP2	protein_coding
108392372	1.743709195	0.025540275	LOC108392372	protein_coding
108406892	-1.25794933	0.025540275	SMIM20	protein_coding
108398738	-1.55090393	0.025600593	EIF3B	protein_coding
108399779	-2.20932025	0.0256836	MORN4	protein_coding
novel.6815	1.774912536	0.0256836	-	-
108404843	-1.46903072	0.0256836	CUNH19orf47	protein_coding
108393659	-1.90962565	0.025871195	FUT4	protein_coding
novel.1277	-1.71177407	0.026110233	-	-

108409082	1.49468066	0.026110233	LOC108409082	protein_coding
108390359	-2.28680477	0.026135221	TPPP3	protein_coding
108395907	1.754665206	0.026195726	LOC108395907	lncRNA
108401772	-5.30343903	0.026333645	CAV3	protein_coding
108405911	-1.88116255	0.026343406	DMRT2	protein_coding
108393707	1.600946013	0.026343406	TMC7	protein_coding
108402520	-1.43481881	0.026371367	LOC108402520	protein_coding
108401631	-2.75909114	0.026470379	LOC108401631	lncRNA
108405998	-2.37877202	0.026602626	LOC108405998	protein_coding
108401702	2.529587728	0.026607036	GALNT6	protein_coding
108406032	1.59605132	0.026673966	LOC108406032	protein_coding
novel.7231	-6.26989704	0.026742857	-	-
108402376	-1.32402732	0.026819492	PYGL	protein_coding
108397873	-1.551283	0.026999007	BAG6	protein_coding
108404294	-2.74503435	0.027073315	LOC108404294	protein_coding
novel.14	-1.67736615	0.027073315	-	-
novel.2050	-3.04904272	0.027077581	-	-
novel.4888	-3.59849925	0.027160444	-	-
108408282	-3.35393642	0.027167554	LOC108408282	lncRNA
108401682	-1.31832773	0.027167554	SNRNP35	protein_coding
108407082	-4.72795428	0.027211452	LOC108407082	protein_coding
novel.1605	-1.47133791	0.02726906	-	-
108402812	-1.82082005	0.027276655	TTC17	protein_coding
108384239	-2.85317644	0.027276655	ADGRA3	protein_coding
108389037	2.478534056	0.027323256	SLC34A3	protein_coding
108398984	1.327036418	0.027392287	CCDC151	protein_coding
novel.4146	-1.42392565	0.02749754	-	-
108393277	-1.49909137	0.027534919	NFIC	protein_coding
108391017	-5.23279811	0.027534919	CKM	protein_coding
108384627	-1.66967119	0.027534919	ZFYVE28	protein_coding
108395820	-2.57361515	0.027534919	FAM19A4	protein_coding
108392024	-1.04238761	0.02768176	COPS6	protein_coding
108390358	1.492124705	0.027706077	SMPDL3B	protein_coding
108409758	-1.64477116	0.027872972	WFS1	protein_coding
108401559	-5.01806475	0.027872972	LOC108401559	lncRNA
108393118	-1.29941556	0.027919435	KTI12	protein_coding
108406235	-3.22960897	0.028019286	PGAM2	protein_coding
108403513	1.367172144	0.0282023	RAPGEF5	protein_coding
108395919	0.976117202	0.028286119	DGKA	protein_coding
108408453	1.886167091	0.02828792	CHEK2	protein_coding
108393255	-1.126203	0.02835485	OAZ1	protein_coding
108390873	-5.54915126	0.02836701	PDILT	protein_coding
novel.3537	-3.91318828	0.02836701	-	-
novel.5288	-3.45324449	0.028383186	-	-

108409022	-2.2835782	0.02840578	LOC108409022	lncRNA
108402271	3.810946457	0.028494049	DQX1	protein_coding
novel.2509	1.459449877	0.028538579	-	-
108407841	-1.95444749	0.028664559	LOC108407841	pseudogene
novel.8630	-3.12615804	0.028688056	-	-
108409957	1.266695941	0.028764582	CCNL1	protein_coding
108399124	-5.96727236	0.028764582	VGLL2	protein_coding
108399689	2.155762139	0.028775728	PCP2	protein_coding
108393775	-2.80115389	0.028805335	SLC35G2	protein_coding
108395341	-2.80130736	0.028805713	LOC108395341	protein_coding
108384917	0.975448582	0.02890418	ATL2	protein_coding
108389970	1.65149696	0.029164388	PUS10	protein_coding
108385949	-1.84124877	0.029215334	PM20D1	protein_coding
novel.668	2.049757718	0.029232375	-	-
108385009	-1.03830737	0.029408579	TMEM161A	protein_coding
108406324	-2.46542939	0.029481786	MYH7B	protein_coding
108389579	-3.99236995	0.02952166	LOC108389579	protein_coding
108401655	-1.87120521	0.029595374	BMF	protein_coding
novel.7296	-2.82367895	0.029600867	-	-
108385511	2.607622176	0.029653644	DLGAP5	protein_coding
108400299	1.928419742	0.029653644	LOC108400299	protein_coding
108394736	-1.30441746	0.029653644	LOC108394736	protein_coding
108405766	-2.51034969	0.029778112	GPR150	protein_coding
108394042	-2.45982041	0.029778112	GRB14	protein_coding
novel.991	-3.3394364	0.029846159	-	-
108397467	1.406410851	0.0299998	LOC108397467	lncRNA
108388007	2.642854052	0.0299998	SLC5A5	protein_coding
108400844	1.901822126	0.0299998	LOC108400844	protein_coding
108405384	1.842216142	0.0299998	ITGB7	protein_coding
108397708	-4.71133023	0.0299998	LOC108397708	protein_coding
108404952	-1.82683343	0.030027912	EFCAB14	protein_coding
108398643	-2.46595339	0.030259396	VWA5B1	protein_coding
108407762	-1.67396537	0.030269626	RAB3IL1	protein_coding
108402128	-2.11520216	0.030269626	ZBTB16	protein_coding
108392841	-2.34266946	0.030316535	LDHD	protein_coding
novel.2913	-1.5804485	0.03033375	-	-
novel.4830	-5.96460474	0.030352556	-	-
108383861	-1.13439794	0.03036596	MPC2	protein_coding
108400357	-1.6844509	0.03044893	RRAGD	protein_coding
108388515	-1.54500563	0.03044893	GRB10	protein_coding
108405635	0.974067428	0.030463022	10-Sep	protein_coding
108391088	1.739050011	0.030730892	LOC108391088	protein_coding
108401630	1.826123806	0.03075002	PPP1R16A	protein_coding
108392765	-1.27486591	0.030915335	GGA2	protein_coding

108407259	1.061374096	0.031240745	LNX2	protein_coding
108390806	1.922310079	0.031240745	IFFO2	protein_coding
108383493	1.230832075	0.031256911	CACHD1	protein_coding
novel.6309	-2.19552204	0.031256911	-	-
108397574	-1.86243618	0.031260116	ZNF862	protein_coding
novel.6451	1.830679331	0.031260116	-	-
108389879	1.594694528	0.031281807	LOC108389879	protein_coding
108404277	-1.16901061	0.031473261	LOC108404277	protein_coding
novel.7676	2.091725646	0.031506533	-	-
108387603	1.009584243	0.031506533	CLASP2	protein_coding
108393522	-1.33248311	0.031514256	CNOT11	protein_coding
108403596	-1.33720703	0.031514256	GNA11	protein_coding
108391771	-2.53293586	0.031519658	LOC108391771	protein_coding
108393490	-1.87129523	0.03154769	LOC108393490	protein_coding
108407433	-4.32369576	0.031589151	UNC79	protein_coding
novel.4427	-1.52841734	0.031590799	-	-
108393647	2.744472034	0.031668276	LOC108393647	lncRNA
novel.852	-5.13847766	0.031788231	-	-
108404424	-1.18485261	0.031936309	NDUFAF4	protein_coding
108392473	-3.80320311	0.032027022	CACNB2	protein_coding
108409888	1.32328304	0.032085626	DDR1	protein_coding
108407577	2.464885264	0.032085626	TEX40	protein_coding
novel.7121	-2.33841327	0.032088724	-	-
108398292	-2.26426274	0.032546009	LOC108398292	protein_coding
108393158	-1.17806401	0.032630258	POLD4	protein_coding
108396122	2.048962648	0.032630258	LOC108396122	lncRNA
108398311	0.965830346	0.032630258	INTS2	protein_coding
108395707	2.508633644	0.03282867	HEPHL1	protein_coding
108404283	1.807155679	0.032893555	LOC108404283	protein_coding
108398714	-1.79277229	0.032952745	UHRF1BP1	protein_coding
108398389	2.533686193	0.033040676	SARDH	protein_coding
108394495	1.102321556	0.033040676	RASA1	protein_coding
108392396	-3.65850896	0.033040676	LOC108392396	protein_coding
108384677	-1.47266945	0.033073667	KLHL21	protein_coding
novel.4946	-1.94896771	0.033101286	-	-
108399379	-1.6962059	0.033250338	ULK1	protein_coding
108394728	-1.16514273	0.033250338	FGF13	protein_coding
novel.8000	-1.80791001	0.033250338	-	-
108388118	-1.09627482	0.033250338	TOP3B	protein_coding
novel.5986	-2.51487008	0.033407964	-	-
108393344	-1.18722121	0.033425862	APEH	protein_coding
108399118	2.941226518	0.033425862	LOC108399118	lncRNA
108397099	2.88987717	0.033425862	CENPE	protein_coding
108407283	-1.36752504	0.03353139	SCAMP3	protein_coding

108400003	-5.91251469	0.033625133	LOC108400003	protein_coding
108400720	-1.81106199	0.033625133	KIF1B	protein_coding
108406793	-2.68331334	0.03368025	LOC108406793	lncRNA
108407171	1.196409413	0.03368025	F11R	protein_coding
108390273	1.576239126	0.03368025	CUNH3orf58	protein_coding
novel.6530	-2.66813158	0.033695655	-	-
108400033	-1.17748921	0.033710633	PSMB5	protein_coding
novel.744	-4.8220724	0.033710633	-	-
108384223	1.950089806	0.033710633	GJA1	protein_coding
108386209	2.535811428	0.033834556	TCN1	protein_coding
novel.4702	-3.61000439	0.034018364	-	-
novel.1368	1.483588742	0.034018364	-	-
108394342	-1.25775178	0.034097669	MKRN2	protein_coding
novel.6224	-5.94095842	0.034105523	-	-
108385230	-1.07729122	0.034174297	CUNH19orf12	protein_coding
108383792	1.637087143	0.034174297	CEP170B	protein_coding
108391390	-1.4657541	0.034174297	LOC108391390	protein_coding
novel.779	-5.23978653	0.034200281	-	-
108394240	-2.62688147	0.034229205	LOC108394240	protein_coding
108395525	1.833091598	0.034289494	LOC108395525	lncRNA
108393565	-5.5511637	0.034617142	CSRP3	protein_coding
108388713	-2.84739998	0.034617142	LOC108388713	protein_coding
108392642	3.461514513	0.034712778	TMPRSS11A	protein_coding
108408808	-1.78239872	0.034784101	PACSIN3	protein_coding
108391224	-5.30570415	0.034794247	BIK	protein_coding
108397886	-1.51837847	0.034954226	LOC108397886	protein_coding
108392867	1.36701453	0.034960352	FAS	protein_coding
novel.4961	-3.17724939	0.034986208	-	-
108393700	1.510865297	0.034991864	LRRC8D	protein_coding
108399693	1.82692013	0.034991864	VAV3	protein_coding
108402225	-1.01542004	0.034991864	COQ10A	protein_coding
108401346	1.944098713	0.035320731	FERMT1	protein_coding
108410223	1.709345538	0.035327498	SULT4A1	protein_coding
108395526	-1.17620574	0.035367885	MED16	protein_coding
108392880	-1.59454759	0.035367885	CCDC80	protein_coding
108396498	-1.12276498	0.035369108	CCM2	protein_coding
108398183	-1.33761063	0.035507717	ABHD2	protein_coding
novel.8684	-3.10953337	0.035536845	-	-
108386975	-2.22025313	0.035536845	LOC108386975	lncRNA
novel.7888	-5.53427493	0.035634734	-	-
108399906	-3.65272842	0.035655209	SLIT1	protein_coding
novel.1126	4.526398145	0.035743576	-	-
108404732	-1.65341099	0.035909882	RHOBTB1	protein_coding
108393807	1.612567208	0.036006718	PARP8	protein_coding

108392599	-1.78167611	0.036146535	TMTC1	protein_coding
108387864	-1.5782677	0.036218669	AIFM2	protein_coding
108409210	-1.61059494	0.036459671	TBCEL	protein_coding
108408473	-1.47012665	0.036459671	PRPF40B	protein_coding
108385815	1.49813476	0.036479859	NDUFA4L2	protein_coding
108403012	-5.6296137	0.036514255	DNAH11	protein_coding
108404683	-1.63002857	0.036519612	PTGFR	protein_coding
108400340	-4.73806877	0.036623904	KCNA7	protein_coding
108393496	-1.06869416	0.036736655	ANXA11	protein_coding
108383746	2.534573447	0.03678216	CPA5	protein_coding
novel.7738	-1.67818422	0.036829665	-	-
108386745	-2.6679341	0.036840785	SOX6	protein_coding
108402597	-6.98227558	0.037068681	LOC108402597	protein_coding
108386424	2.797252819	0.037127107	CLSPN	protein_coding
108383487	-2.17751563	0.037127107	LOC108383487	lncRNA
108405791	-3.84289366	0.037184623	EFCAB5	protein_coding
novel.8540	-2.07865706	0.037184623	-	-
108384329	-1.23368445	0.037294715	LRP3	protein_coding
108397166	-2.02390694	0.037294715	KCNQ5	protein_coding
108397755	-1.17344992	0.037294715	TXN2	protein_coding
108410130	-1.3400921	0.037294715	LOC108410130	protein_coding
novel.3737	2.777977676	0.037363643	-	-
108388639	-1.04723826	0.037363643	ENTPD6	protein_coding
108386437	-3.4210139	0.037363643	NRN1L	protein_coding
108385602	-3.14729595	0.037367198	LOC108385602	protein_coding
108409643	-6.00928176	0.037471925	DUSP27	protein_coding
108403530	-1.54557647	0.037471925	LOC108403530	protein_coding
108393792	-1.30161996	0.037471925	LOC108393792	protein_coding
108398374	-1.65548255	0.037683241	CDS2	protein_coding
108401362	2.686848361	0.037683241	IL31RA	protein_coding
108394561	-3.20348562	0.037777303	LOC108394561	lncRNA
108385969	-2.74562994	0.037848781	FRZB	protein_coding
108409855	-1.30970344	0.037848781	KAT2B	protein_coding
108401224	1.394028843	0.037848781	ALOX12	protein_coding
108389054	-2.6002297	0.037980273	LOC108389054	protein_coding
108392535	1.557882968	0.038101033	CDK6	protein_coding
novel.580	-7.51317456	0.038114108	-	-
108406227	-2.56237833	0.038144715	PDK4	protein_coding
108395990	-1.02400132	0.038202986	DNPEP	protein_coding
108401273	3.194648982	0.03821304	HMMR	protein_coding
novel.492	4.216024553	0.03821304	-	-
108402140	-5.12850763	0.038311293	LOC108402140	lncRNA
108401902	2.28204617	0.038336006	FAM83D	protein_coding
108407682	-1.09199098	0.038336006	HMCES	protein_coding

108401314	1.198433751	0.038420833	INTS6L	protein_coding
108407586	-1.14804208	0.038427697	TRPT1	protein_coding
108397343	-1.05318103	0.038509854	PTDSS2	protein_coding
108401853	3.466648767	0.038773065	RPS6KA6	protein_coding
108384129	1.629901157	0.038891332	TOR4A	protein_coding
108402700	-1.79727603	0.038999157	LOC108402700	pseudogene
108389568	-1.64655049	0.039048253	LOC108389568	lncRNA
108406864	1.74448958	0.039048253	TBX1	protein_coding
108385731	-2.21404158	0.039074644	LOC108385731	protein_coding
108395374	1.942878825	0.039357529	PAPLN	protein_coding
108392402	-1.71035323	0.039357529	WIPI1	protein_coding
108391021	-1.31768024	0.039357529	CD3EAP	protein_coding
108392308	-1.2567353	0.039357529	TATDN1	protein_coding
108409802	-1.05949627	0.039357529	LOC108409802	protein_coding
novel.1672	-2.2858761	0.039386973	-	-
108396783	-1.1004921	0.03940035	STARD7	protein_coding
108400223	-1.21588509	0.039420075	ACAA2	protein_coding
108407662	-1.78464864	0.039429709	PTP4A3	protein_coding
108386120	1.232951095	0.039477989	HAGHL	protein_coding
108395551	-1.07158921	0.039522348	GALT	protein_coding
novel.4272	3.368108127	0.039628372	-	-
108395915	1.633838482	0.039628372	LOC108395915	protein_coding
108392080	-1.64352575	0.039642596	DMWD	protein_coding
108406499	1.340177415	0.039642596	NLGN2	protein_coding
108406732	-3.38705869	0.039642596	LOC108406732	protein_coding
novel.2092	2.79106306	0.039823949	-	-
novel.7651	-3.59797902	0.039913932	-	-
108409316	-1.57822358	0.039929391	SLC1A7	protein_coding
108407538	-1.54971293	0.039929391	ADGRG2	protein_coding
108398672	-1.44124576	0.039955575	UBE2O	protein_coding
108390981	-1.91031834	0.039955575	LOC108390981	protein_coding
108389971	1.281713683	0.040082612	REL	protein_coding
108406537	-1.05458249	0.040082612	LOC108406537	protein_coding
108395862	-1.88195791	0.040195384	PCOLCE2	protein_coding
108398787	2.428301818	0.040200541	CATSPER4	protein_coding
108402519	-1.75883031	0.040291235	LOC108402519	pseudogene
108403639	2.411634438	0.04061186	LOC108403639	protein_coding
108388216	-1.6940201	0.040678056	SSPN	protein_coding
novel.100	-3.03282198	0.040678056	-	-
108395675	-2.03890376	0.040691395	IGDCC4	protein_coding
108395211	-1.63734697	0.040714487	TXNRD1	protein_coding
novel.8224	-4.16749553	0.040724046	-	-
108390753	-5.97326515	0.040757985	ESRRB	protein_coding
108387338	-2.23038878	0.040794396	P2RY12	protein_coding

108396812	0.956378387	0.040839967	NUFIP2	protein_coding
108391406	1.318410669	0.040915471	SH3GL1	protein_coding
108393747	-1.21743873	0.041000555	SDCCAG8	protein_coding
novel.4968	-2.94692211	0.041070979	-	-
108391551	-1.26139666	0.04109617	COPS3	protein_coding
108384494	2.224022163	0.041229395	LOC108384494	lncRNA
108396834	-3.60628616	0.041540682	LOC108396834	lncRNA
108393706	-2.91885339	0.041662146	TMC5	protein_coding
108406519	1.352797894	0.041662146	MAP3K6	protein_coding
108400006	1.181308696	0.042018513	RBM23	protein_coding
108407637	-5.22845468	0.042018513	SLN	protein_coding
108402037	-2.30246274	0.042021357	SLCO5A1	protein_coding
108389328	-1.35133886	0.042021852	LOC108389328	lncRNA
108406927	1.303062178	0.042021852	SMC4	protein_coding
108398596	-1.42004201	0.042021852	BFAR	protein_coding
108400456	1.406904468	0.042021852	NBEAL2	protein_coding
108386898	-2.27520037	0.042021852	LOC108386898	lncRNA
108401191	1.614176021	0.042053865	LTC4S	protein_coding
108390603	1.845329971	0.042111144	PPARGC1B	protein_coding
108388208	-1.08027294	0.042182077	ARMC12	protein_coding
novel.8591	4.670639126	0.042182077	-	-
108409276	-1.68611607	0.042207658	ABCB4	protein_coding
novel.8628	2.09414181	0.042347644	-	-
108407476	-1.10098641	0.04245922	COQ9	protein_coding
108400106	1.001884282	0.042467229	CUNH15orf59	protein_coding
108404730	-0.91547793	0.042485347	FTSJ1	protein_coding
108389547	3.980534401	0.042485347	LOC108389547	protein_coding
108395596	1.356234914	0.042490593	PLXNB2	protein_coding
108409583	-0.82762052	0.042571713	SEC31B	protein_coding
108405521	1.293082588	0.042847228	FRS2	protein_coding
108385344	1.443356029	0.042854283	LOC108385344	lncRNA
novel.7765	4.569533898	0.043096968	-	-
108390379	2.310688097	0.043260661	LOC108390379	protein_coding
108408098	-1.2197461	0.043508773	TRIM3	protein_coding
24019681	-1.52650483	0.043508773	CYTB	protein_coding
novel.8770	2.34794235	0.043517783	-	-
108391338	1.256494154	0.043518909	LOC108391338	protein_coding
108410344	1.506127996	0.043797974	PTPN13	protein_coding
108402112	-1.74812517	0.043856411	ORC6	protein_coding
novel.658	-2.05255855	0.043924797	-	-
novel.2871	2.821284711	0.043924797	-	-
108388622	2.388480781	0.043924797	LOC108388622	lncRNA
108395230	-3.63986502	0.043924797	GCGR	protein_coding
108400295	-1.71418981	0.043924797	RRAS	protein_coding

108393193	1.21492126	0.043972667	CA11	protein_coding
108401296	1.14113632	0.04399132	TTL	protein_coding
108397522	1.405492042	0.04399132	TNK2	protein_coding
novel.3676	-1.79613113	0.04399132	-	-
108407590	-1.36281626	0.044164376	ACCS	protein_coding
108394846	-1.28355019	0.044184009	KLC2	protein_coding
108397999	-1.33615947	0.044193675	FARSB	protein_coding
108403780	1.782389159	0.044312826	SDC1	protein_coding
108389806	2.407375014	0.044476488	LOC108389806	protein_coding
108383736	-1.02994127	0.044559706	PSMC3	protein_coding
108400146	-1.79219017	0.044559706	LAP3	protein_coding
108383589	1.82230047	0.044559706	ZNF711	protein_coding
108398417	-2.73724019	0.044593539	DMGDH	protein_coding
108384976	4.983262788	0.04477766	TMPRSS4	protein_coding
novel.4944	5.023526279	0.04477766	-	-
novel.4861	1.563047035	0.045005937	-	-
novel.7462	-2.15965734	0.045005937	-	-
novel.2176	2.709824706	0.045005937	-	-
108395520	2.060909095	0.045180653	FGFR2	protein_coding
novel.1167	-2.15170197	0.045197197	-	-
108408777	-1.13214147	0.045214558	BCL2A1	protein_coding
novel.2859	-3.92215744	0.045255572	-	-
108405984	-2.36501593	0.045255572	SPSB1	protein_coding
108398505	-0.87775236	0.045255572	WBSCR22	protein_coding
108388553	1.848240232	0.04556987	LOC108388553	lncRNA
108403230	-1.63143058	0.04556987	LOC108403230	protein_coding
108395906	1.90394122	0.045676864	ZC3H12A	protein_coding
108406586	-1.17250802	0.045752449	KCMF1	protein_coding
108392172	2.092611625	0.045914093	SLC22A15	protein_coding
108402401	3.635287761	0.045953244	LOC108402401	lncRNA
108401928	-0.80140908	0.045953244	UBE2E3	protein_coding
108384900	-6.41681095	0.045953244	LOC108384900	protein_coding
108386730	-5.70112014	0.045953244	LOC108386730	protein_coding
108402774	-0.99267612	0.046047586	ANKRD40	protein_coding
108393922	-2.15057246	0.046117775	PLA2G16	protein_coding
108408538	4.432701099	0.046117775	SORCS3	protein_coding
108403409	-1.32929552	0.046117775	SCMH1	protein_coding
108410005	2.930251508	0.046140032	CUNH17orf53	protein_coding
108389844	2.434600246	0.046140032	LOC108389844	protein_coding
108390353	-1.54200078	0.046175871	FHOD1	protein_coding
108398239	1.844446426	0.046358734	LOC108398239	lncRNA
novel.6331	-5.67262313	0.046358734	-	-
108397483	1.687131482	0.046358734	LOC108397483	protein_coding
108402781	3.73981304	0.046432474	LOC108402781	lncRNA

108397558	-2.97184566	0.046476537	CA14	protein_coding
108399276	-1.11367052	0.046621807	ATPIF1	protein_coding
108397195	-1.22393503	0.046801396	RHBDD1	protein_coding
108400882	-1.64909283	0.047103204	LOC108400882	protein_coding
108406895	-2.13126678	0.047156928	SEL1L3	protein_coding
108391184	1.751686614	0.047193538	DRC1	protein_coding
108384083	1.154386912	0.047193538	SLC2A9	protein_coding
108405995	1.191806284	0.047210061	AK4	protein_coding
108390191	-2.53904303	0.047210061	THBS4	protein_coding
108389266	-0.85882363	0.047247326	NDUFB10	protein_coding
108391320	2.831938342	0.047247326	CENPF	protein_coding
novel.6904	-2.59802656	0.047457045	-	-
108394788	-1.08932439	0.047684394	CUNH9orf78	protein_coding
108396045	1.462974541	0.047837905	PLK4	protein_coding
108400191	-0.85552887	0.047837905	MORF4L1	protein_coding
108406417	1.082793426	0.04788134	PPP2R3A	protein_coding
108388361	-1.4758195	0.047962773	LOC108388361	protein_coding
108398040	2.427428279	0.048005386	KIF11	protein_coding
108395449	-1.81586599	0.048005386	FAM219A	protein_coding
108386069	-1.35435448	0.048005386	EZH1	protein_coding
novel.800	-3.08028418	0.048005386	-	-
108400475	-1.18762957	0.048008765	PSMA5	protein_coding
108386064	-1.23983319	0.048008765	MLX	protein_coding
108404103	-1.20380497	0.048008765	LOC108404103	lncRNA
108403257	-2.11784201	0.048119098	LOC108403257	protein_coding
108395517	-1.53868116	0.048153046	SDC2	protein_coding
108405693	1.270062562	0.048177391	AGRN	protein_coding
108397699	-1.32343631	0.048256707	RNF10	protein_coding
108402834	-4.63524798	0.04835098	LOC108402834	protein_coding
108402273	-1.27559344	0.048442929	DCTN1	protein_coding
108388581	1.331425249	0.048442929	SH3BP2	protein_coding
108400773	-1.29240699	0.048633848	EVL	protein_coding
108389651	2.297715473	0.048912927	LOC108389651	protein_coding
novel.4441	-3.61592907	0.048970729	-	-
108407572	-1.53069131	0.048970729	LOC108407572	lncRNA
108393891	2.409826987	0.049171183	ARHGEF19	protein_coding
108383523	-1.16216643	0.049171183	PFDN2	protein_coding
108409729	-1.75141849	0.049283305	SPATA18	protein_coding
108385727	1.778636327	0.049283305	CLCN6	protein_coding
108390393	-1.22733275	0.049283305	WDR66	protein_coding
108406112	0.953144397	0.04945949	SRSF7	protein_coding
108401734	-1.59382533	0.049462347	LOC108401734	pseudogene
108395189	-2.37516976	0.049594479	ESM1	protein_coding
108393302	-1.65880636	0.049764855	HSPB1	protein_coding

novel.5908	2.682482974	0.049788477	-	-
108409078	2.111671778	0.049788477	SLC24A4	protein_coding
108410284	-1.36577648	0.049935464	SLC25A28	protein_coding
108388878	-5.84325842	0.049994332	NMRK2	protein_coding

Supplementary Table 8. DEGs (adult-hair vs adult-scale group) enriched in KEGG signaling pathways related to skin appendage differentiation

KEGG ID	KEGG pathway	padj	Count	Gene Name
mmu04010	MAPK signaling pathway	0.19	64	FLNC, FGF1, MAP2K6, CACNB1, CACNG6, GADD45A, CACNA1S, CACNG1, MAPT, GADD45G, CACNA2D1, EPHA2, DUSP3, RASA1, FAS, VEGFA, RRAS, MAP3K6, AKT3, MEF2C, RPS6KA6, MAP3K5, LOC108397962, FGF22, MAP3K1, TGFB2, BRAF, MAP3K8, LOC108389056, NFATC1, RPS6KA3, LOC108407494, TGFB3, PGF, FGFR2, STK4, ANGPT2, JUN, LOC108402853, FGF10, DUSP7, HSPB1, MAPK12, PLA2G4D, FGF5, MAPKAPK2, MRAS, FOS, DUSP2, NTF4, LOC108387329, NGFR, MAPK11, PLA2G4F, AREG, MET, PRKACA, MAP2K2, FGFR4, RAF1, NFKB1, DUSP6, VEGFB
mmu04014	Ras signaling pathway	0.35	45	FGF1, EPHA2, RASA1, VEGFA, RRAS, LOC108392372, REL, GAB2, PLA2G16, AKT3, FOXO4, LOC108409892, LOC108397962, FGF22, RIN1, GNG4, LOC108407494, PGF, FGFR2, STK4, PLD2, ANGPT2, GNB5, PIK3R1, FGF10, PLA2G2E, PLA2G4D, PIK3R2, FGF5, MRAS, NTF4, NGFR, PLA2G4F, KSR2, MET, PRKACA, GNG2, MAP2K2, RAPGEF5, FGFR4, RAF1, PLD1, NFKB1, VEGFB, GNG11
mmu04015	Rap1 signaling pathway	0.34	44	FGF1, MAP2K6, P2RY1, EPHA2, VEGFA, RRAS, RAPGEF1, LOC108392372, LOC108406701, EVL, PRKCI, AKT3, SIPA1L2, LOC108397962, PRKD2, FGF22, SIPA1L3, BRAF, CDH1, LPAR3, RAPGEF3, PGF, FGFR2, ANGPT2, MAGI3, PIK3R1, FGF10, MAPK12, ADCY6, PIK3R2, FGF5, MRAS, NGFR, PARD6B, MAPK11, MET, RGS14, SRC, LPAR5, MAP2K2, RAPGEF5, FGFR4, RAF1, VEGFB
mmu04510	Focal adhesion	0.50	40	FLNC, MYLK2, ITGA10, MYLPF, PARVB, ITGB6, CAV3, MYL2, ITGB7, VEGFA, RAPGEF1, THBS4, AKT3, ROCK2, LOC108409892, LOC108397962, COL4A5, LOC108401680, BRAF, CCND1, PGF, BIRC3, JUN, BCL2, ITGB4, PIK3R1, LOC108385698, PIK3R2, ARHGAP5, LOC108405577, SPP1, ITGA3, ITGB8, TNN, LOC108389457, MET, SRC, RAF1, VEGFB, LOC108406127
mmu04068	FoxO signaling pathway	0.02	36	FBXO32, GADD45A, BCL6, GADD45G, SMAD3, FOXO1, PRKAG3, AKT3, GABARAPL1, CCNB1, FOXO4, PRKAB2, LOC108397962, TGFB2, BRAF, TGFB3, CCND1, STK4, PLK3, LOC108390100, IL7R, PIK3R1, MAPK12, IRS1, PIK3R2, TNFSF10, PLK4, CDK2, LOC108403230, LOC108401022, SETD7, MAPK11, SLC2A4, CDKN2B, MAP2K2, RAF1
mmu04530	Tight junction	0.43	33	BVES, SYNPO, YBX3, MYL2, MYL6B, LOC108392372, PRKAG3, PRKCI, F11R, LOC108401434, CLDN14, ROCK2, PRKAB2, MAP3K5, MAP3K1, MARVELD2, LOC108401433, MPDZ, MICALL2, MYH7B, CCND1, LOC108400752, JUN, TJP2,

				PPP2R2A, LOC108388016, DLG3, PARD6B, LOC108408766, TJP3, SRC, PRKACA, WHAMM
mmu04390	Hippo signaling pathway	0.74	28	FGF1, RASSF6, SMAD3, PRKCI, WNT9A, TGFB2, CDH1, TGFB3, CCND1, WNT4, LOC108400752, BMP7, WNT11, PPP2R2A, APC2, LOC108394467, LOC108388016, DLG3, TCF7, FZD4, PARD6B, TEAD2, AREG, AJUBA, BMP4, SMAD1, YAP1, WNT9B
mmu04110	Cell cycle	0.39	27	CDKN1C, GADD45A, GADD45G, TFDP2, SMAD3, CDK6, CHEK2, MCM3, CCNB1, DBF4, CDK7, TGFB2, CCNA2, TGFB3, CCND1, CDC7, CDC14A, LOC108384777, LOC108401648, CHEK1, ORC6, ESPL1, LOC108394467, CDK2, CCNE1, CDKN2B, LOC108400585
mmu04210	Apoptosis	0.59	26	GADD45A, GADD45G, ENDOG, FAS, AIFM1, AKT3, MAP3K5, PTPN13, CTSZ, BCL2A1, BAK1, BIRC3, JUN, BCL2, PIK3R1, LOC108387640, CTSS, PIK3R2, TNFSF10, FOS, PARP2, LOC108404249, NFKBIA, MAP2K2, RAF1, NFKB1
mmu04310	Wnt signaling pathway	0.99	24	SMAD3, PRICKLE3, LOC108394519, VANGL2, FRAT1, WNT9A, ROCK2, NFATC1, CCND1, WNT4, JUN, WNT11, CXXC4, APC2, CAMK2B, TCF7, FZD4, RSPO3, DAAM1, PRKACA, CHD8, CTBP1, WNT9B, CAMK2G
mmu04380	Osteoclast differentiation	0.97	19	MAP2K6, MITF, CALCR, GAB2, AKT3, SQSTM1, IFNGR2, TGFB2, LOC108389056, NFATC1, JUN, PIK3R1, MAPK12, PIK3R2, FOS, MAPK11, NFKBIA, SOCS1, NFKB1
mmu04512	ECM-receptor interaction	0.57	19	ITGA10, ITGB6, NPNT, CD44, ITGB7, THBS4, HMMR, SDC1, COL4A5, LOC108401680, AGRN, ITGB4, LOC108405577, SPP1, LOC108385602, ITGA3, ITGB8, TNN, LOC108389457
mmu04115	p53 signaling pathway	0.27	18	SESN1, GADD45A, GADD45G, FAS, CDK6, AIFM2, CHEK2, CCNB1, RRM2, CCND1, LOC108390234, BCL2, CHEK1, MDM4, LOC108387640, CDK2, CCNE1
mmu04658	Th1 and Th2 cell differentiation	0.64	17	LOC108388330, JAG1, DLL1, NOTCH3, IFNGR2, NOTCH2, JAG2, NFATC1, NOTCH1, JUN, MAPK12, FOS, NFKBIB, MAPK11, NFKBIA, NFKB1, MAF
mmu04659	Th17 cell differentiation	0.99	15	LOC108388330, SMAD3, IL6R, IFNGR2, AHR, NFATC1, JUN, MAPK12, FOS, MTOR, HIF1A, NFKBIB, MAPK11, NFKBIA, NFKB1
mmu04370	VEGF signaling pathway	0.40	14	VEGFA, AKT3, PIK3R1, HSPB1, MAPK12, PLA2G4D, PIK3R2, MAPKAPK2, MAPK11, PLA2G4F, SRC, NOS3, MAP2K2, RAF1
mmu04350	TGF-beta signaling pathway	0.99	14	SMAD3, ACVR1B, TGFB2, TGIF2, TGFB3, BMP7, LEFTY1, SMURF2, CDKN2B, BMP4, SMAD9, ACVR2A, SMAD1, LOC108400585
mmu04330	Notch signaling pathway	0.73	11	PSEN2, JAG1, DLL1, HEY2, NOTCH3, KAT2B, NOTCH2, JAG2, HES1, NOTCH1, CTBP1
mmu03320	PPAR signaling pathway	0.99	10	CPT1B, ME3, LOC108407606, SLC27A1, SORBS1, NR1H3, CPT1C, ACSL4, DBI, ACSBG1

mmu04340	Hedgehog signaling pathway	0.99	7	CCND1, BOC, BCL2, CDON, SMURF2, PRKACA, KIF3A
